# An integrated genetic-epigenetic analysis of schizophrenia: evidence for co-localization of genetic associations and differential DNA methylation

**DOI:** 10.1186/s13059-016-1041-x

**Published:** 2016-08-30

**Authors:** Eilis Hannon, Emma Dempster, Joana Viana, Joe Burrage, Adam R. Smith, Ruby Macdonald, David St Clair, Colette Mustard, Gerome Breen, Sebastian Therman, Jaakko Kaprio, Timothea Toulopoulou, Hilleke E. Hulshoff Pol, Marc M. Bohlken, Rene S. Kahn, Igor Nenadic, Christina M. Hultman, Robin M. Murray, David A. Collier, Nick Bass, Hugh Gurling, Andrew McQuillin, Leonard Schalkwyk, Jonathan Mill

**Affiliations:** 1 University of Exeter Medical School, University of Exeter, Exeter, UK; 2 The Institute of Medical Sciences, Aberdeen University, Aberdeen, UK; 3 University of the Highlands and Islands, Inverness, UK; 4 Institute of Psychiatry, Psychology & Neuroscience (IoPPN), King’s College London, London, UK; 5 National Institute for Health and Welfare, Helsinki, Finland; 6 Institute for Molecular Medicine, University of Helsinki, Helsinki, Finland; 7 Department of Public Health, University of Helsinki, Helsinki, Finland; 8 Department of Psychology, The University of Hong Kong, Pokfulam, Hong Kong; 9 Department of Psychiatry, Brain Center Rudolf Magnus, University Medical Center Utrecht, Utrecht, The Netherlands; 10 Department of Psychiatry and Psychotherapy, Jena University Hospital, Jena, Germany; 11 Department of Medical Epidemiology and Biostatistics, Karolinska Institutet, Solna, Sweden; 12 Eli Lilly and Company Ltd, Windlesham, UK; 13 Division of Psychiatry, University College London, London, UK; 14 School of Biological Sciences, University of Essex, Colchester, UK; 15 Royal Devon & Exeter Hospital, RILD Building, Level 4, Barrack Rd, Exeter, EX2 5DW UK

**Keywords:** Schizophrenia, DNA methylation, Epigenetics, Genetics, Polygenic risk score (PRS), Genome-wide association study (GWAS), Epigenome-wide association study (EWAS)

## Abstract

**Background:**

Schizophrenia is a highly heritable, neuropsychiatric disorder characterized by episodic psychosis and altered cognitive function. Despite success in identifying genetic variants associated with schizophrenia, there remains uncertainty about the causal genes involved in disease pathogenesis and how their function is regulated.

**Results:**

We performed a multi-stage epigenome-wide association study, quantifying genome-wide patterns of DNA methylation in a total of 1714 individuals from three independent sample cohorts. We have identified multiple differentially methylated positions and regions consistently associated with schizophrenia across the three cohorts; these effects are independent of important confounders such as smoking. We also show that epigenetic variation at multiple loci across the genome contributes to the polygenic nature of schizophrenia. Finally, we show how DNA methylation quantitative trait loci in combination with Bayesian co-localization analyses can be used to annotate extended genomic regions nominated by studies of schizophrenia, and to identify potential regulatory variation causally involved in disease.

**Conclusions:**

This study represents the first systematic integrated analysis of genetic and epigenetic variation in schizophrenia, introducing a methodological approach that can be used to inform epigenome-wide association study analyses of other complex traits and diseases. We demonstrate the utility of using a polygenic risk score to identify molecular variation associated with etiological variation, and of using DNA methylation quantitative trait loci to refine the functional and regulatory variation associated with schizophrenia risk variants. Finally, we present strong evidence for the co-localization of genetic associations for schizophrenia and differential DNA methylation.

**Electronic supplementary material:**

The online version of this article (doi:10.1186/s13059-016-1041-x) contains supplementary material, which is available to authorized users.

## Background

Schizophrenia is a severe, highly heritable, neuropsychiatric disorder characterized by episodic psychosis and altered cognitive function. With a lifetime prevalence rate of ~1 %, schizophrenia contributes significantly to the global burden of disease, ranking amongst the top 10 causes of disability in developed countries worldwide [1]. Schizophrenia has a highly complex etiology, aggregating in families but not segregating in a Mendelian manner. Recent approaches to understanding the causes of schizophrenia have focused on describing the genetic contribution to the disorder; the advent of large-scale genome-wide association studies (GWAS) and exome sequencing has enabled a systematic, hypothesis-free exploration of genetic risk factors. These “forward-genetics” approaches have been highly successful; a recent large-scale GWAS meta-analysis by the Psychiatric Genomics Consortium (PGC) identified 108 independent genomic loci exhibiting a genome-wide significant association with schizophrenia (*P* < 5 × 10^−8^), with evidence for a substantial polygenic component in signals that fall below this stringent level of significance [2].

Despite success in identifying genetic variants associated with schizophrenia, however, there remains uncertainty about the causal genes involved in disease pathogenesis, and how their function is regulated. Many GWAS variants reside in large regions of strong linkage disequilibrium (LD) and do not directly index coding changes affecting protein structure [3]; instead, they are hypothesized to influence gene regulation, a hypothesis supported by the observation that common variants associated with disease are enriched in regulatory domains, including enhancers and regions of open chromatin [4, 5]. Insights into the functional complexity of the genome have also focused attention on the probable role of non-sequence-based genomic variation in health and disease. Of particular interest are epigenetic processes that regulate gene expression via modifications to DNA, histone proteins, and chromatin. DNA methylation is the best-characterized epigenetic modification, stably influencing gene expression via disruption of transcription factor binding and recruitment of methyl-binding proteins that initiate chromatin compaction and gene silencing. Despite being traditionally regarded as a mechanism of transcriptional repression, DNA methylation is actually associated with both increased and decreased gene expression [6], and other genomic functions including alternative splicing and promoter usage [7]. The availability of high-throughput profiling methods for quantifying DNA methylation across the genome at single-base resolution in large numbers of samples has enabled researchers to perform epigenome-wide association studies (EWAS) aimed at identifying methylomic variation associated with environmental exposure and disease [8]; however, these studies are inherently more complex to design and interpret than GWAS [9–11]. The dynamic nature of epigenetic processes means that unlike in genetic epidemiology a range of potentially important confounding factors need to be considered, including tissue or cell type, age, sex, lifestyle exposures, and reverse causation [9]. In recent years there has been a growing interest in the role of developmentally regulated epigenetic variation in the molecular etiology of schizophrenia, supported by data from recent analyses of DNA methylation in co-twins from disease-discordant monozygotic twin pairs [12], clinical sample cohorts [13, 14], and post-mortem brain tissue [15–17].

A better understanding of the molecular mechanisms underlying disease phenotypes is best achieved using an integrated functional genomics strategy, although few studies have attempted to systematically integrate genetic and epigenetic epidemiological approaches. For example, we previously demonstrated how DNA methylation is under local genetic control, identifying an enrichment of DNA methylation quantitative trait loci (mQTL) amongst genomic regions associated with schizophrenia, and highlighting how mQTLs can be used to refine GWAS loci by identifying discrete sites of regulatory variation associated with schizophrenia risk variants [18]. There is also potential for using polygenic risk scores (PRS) – defined as the sum of trait-associated alleles across many genetic loci, weighted by effect sizes estimated by GWAS analyses – as disease biomarkers, although their utility for exploring the molecular genomic mechanisms involved in disease pathogenesis is largely unexplored. For example, PRS-associated epigenetic variation is potentially less affected by factors associated with the disease itself (e.g., medication exposure, stress, and smoking), which can confound case–control analyses.

In this study we present a methodological framework for large EWAS and report widespread differences in DNA methylation between schizophrenia patients and controls in the largest analysis yet undertaken. Leveraging on previous investments in GWAS analyses in schizophrenia, we assessed genome-wide patterns of DNA methylation in a total of 1714 individuals from three independent sample cohorts to identify molecular biomarkers of the disease. Using genetic data from the same individuals, we performed an integrated genetic-epigenetic study to further our functional understanding of common variants associated with schizophrenia etiology. We demonstrate the utility of using PRS for identifying molecular variation associated with etiological variation, and mQTLs for refining the functional/regulatory variation associated with schizophrenia risk variants. Finally, we present strong evidence for the co-localization of genetic associations for schizophrenia and differential DNA methylation.

## Results and discussion

### Methodological overview

We performed a multi-stage EWAS of (1) schizophrenia and (2) schizophrenia PRS, quantifying genome-wide patterns of DNA methylation using the Illumina Infinium HumanMethylation450 BeadChip (“450 K array”) (Illumina Inc., San Diego, CA, USA) in DNA samples isolated from whole blood. After implementing a stringent quality control pipeline (see Methods), our “discovery cohort” (phase 1) included 675 individuals (353 schizophrenia cases and 322 non-psychiatric controls). Schizophrenia-associated differentially methylated positions (DMPs) were subsequently tested in an independent “replication cohort” (phase 2) of 847 individuals (414 schizophrenia cases and 433 non-psychiatric controls; phase 2) and 96 monozygotic (MZ) twin pairs (phase 3). We tested for a significant enrichment of schizophrenia-associated DMPs in regulatory regions, gene ontology (GO) pathways, and genomic regions identified in the recent PGC GWAS of schizophrenia [2]. Finally, we integrated our genetic and epigenetic data to interrogate mQTLs across robust schizophrenia-associated GWAS regions, utilizing Bayesian co-localization analyses to identify genetic variants associated with both schizophrenia and methylomic variation. An overview of our methodological approach is presented in Additional file 1: Figure S1.

### Controlling for confounders in epigenetic epidemiology: smoking as an important covariate for schizophrenia EWAS

**Fig. 1:**
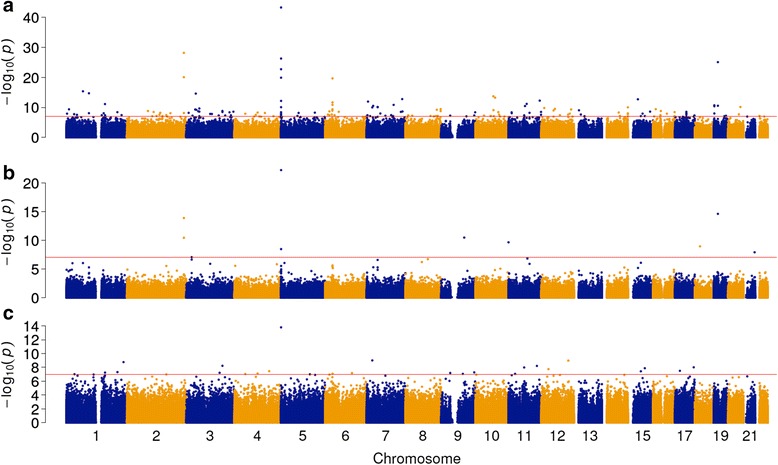
It is critical to control for smoking in an epigenome-wide association study (EWAS) of schizophrenia. Manhattan plots comparing association *P*-values (*y*-axis, −log10 scale) against genomic location (*x*-axis) for (**a**) an EWAS of schizophrenia case–control status without inclusion of a smoking covariate, (**b**) a published EWAS of tobacco smoking (never versus current) [22], and (**c**) an EWAS of schizophrenia case–control status including a DNA methylation-derived smoking score as a covariate. There is considerable overlap between the associated loci in panels a and b (e.g., on chromosomes 2, 5, 6, and 19), implying that the elevated rate and intensity of smoking in patients with schizophrenia [25–27] is a severe confounder in this analysis. In contrast, there is no overlap in the associated loci shown in panels b and c, suggesting that the derived smoking score effectively removes these false positives

Our initial analysis of the phase 1 cohort included covariates for sex and experimental batch, in addition to age and cell composition measures derived from the DNA methylation data [19–21]. We identified 160 schizophrenia-associated DMPs at a stringent experiment-wide significance threshold (*P* < 1 × 10^−7^) representing a 5 % family-wise error-rate estimated from 5000 permutations (see Methods). The top-ranked DMPs were annotated to *AHRR* (cg05575921, cg21161138, cg26529655, cg25648203), *F2RL3* (cg03636183), *GFI1* (cg09935388), and *MYO1G* (cg12803068, cg22132788), in addition to intergenic regions on chromosome 6p21.33 (cg06126421, cg14753356) and 2q37.1 (cg01940273, cg05951221) (Additional file 2: Table S1). Altered DNA methylation at each of these DMPs has been previously associated with cigarette smoking [22–24] (Fig. 1), consistent with epidemiological data highlighting elevated smoking rates and intensity in patients with schizophrenia [25–27]. Because detailed smoking information was not available for every individual in the phase 1 cohort, we derived a proxy variable using DNA methylation values for sites on the 450 K array previously associated with smoking [22, 23]. The resulting smoking scores were consistent with actual smoking status for samples with available smoking data, with current smokers having higher scores than non-smokers (Additional file 1: Figure S2). Across the full sample, DNA methylation-derived smoking scores were significantly higher in patients with schizophrenia compared to controls (Mann–Whitney *P* = 1.51 × 10^−41^; Additional file 1: Figure S3), consistent with epidemiological data [25–27] and the results of our initial EWAS. We next repeated our schizophrenia EWAS analysis using these derived smoking scores as covariates; smoking-associated probes (*P* < 1 × 10^−7^ in [22]) were no longer differentially methylated in the patients with schizophrenia (*P* > 1 × 10^−7^; Additional file 1: Figure S4 and Additional file 2: Table S2) with the exception of cg05575921 (annotated to *AHRR*; *P* = 1.62 × 10^−14^). Although it is possible that DNA methylation at this locus is associated with schizophrenia beyond the confounding effects of smoking, we took the final step of removing all smoking-associated probes from subsequent analyses to enable a clear interpretation of our data.

### DMPs associated with schizophrenia are robust to additional confounding

In total we identified 25 DMPs associated with schizophrenia passing our stringent experiment-wide significance threshold (*P* < 1 × 10^−7^) when controlling for age, sex, experimental batch, and derived estimates of cell composition and smoking (Table 1, Additional file 1: Figure S5). An additional 1223 DMPs were identified at a more relaxed “discovery” threshold of *P* < 5 × 10^−5^ (Additional file 2: Table S3). Because it is likely that other unmeasured environmental exposures also confound our case–control analysis of methylomic variation associated with schizophrenia, we investigated the impact of additional surrogate variables capturing variation in DNA methylation on the association statistics for schizophrenia-associated DMPs. We first compared each of the first 10 principal components (PCs) derived from the DNA methylation data to the available phenotype data to identify potential sources of additional variation between samples (Additional file 1: Figure S6). For example, we observed strong correlations (*r* > 0.5) between the first three PCs and estimated blood cell composition measures, reflecting the likely effect of epigenetic differences between cell types. Age was moderately correlated (*r* > 0.2) with PCs 3, 8, and 9; sex was moderately correlated (*r* > 0.2) with PCs 6 and 7; and smoking was weakly correlated (*r* < 0.2) with each of the top 10 PCs. Although PCs are routinely included in GWAS analyses to control for population stratification, they have not been widely used in DNA methylation studies. We compared the regression coefficients from our initial analysis model (controlling for age, sex, batch, cell composition, and derived smoking score) to sequential models iteratively including up to 10 PCs. We observed a strong positive correlation for schizophrenia-associated DNA methylation differences between analyses (Spearman’s *r* = 0.629 to 0.820), with even stronger similarities observed for the top 1223 discovery schizophrenia-associated DMPs (Spearman’s *r* = 0.952 to 0.983) (Additional file 1: Figure S7). Although additional (unmeasured) confounders are likely to exist in our dataset (e.g., medication exposures, drugs of abuse and stress), our sensitivity analyses showed that the identified schizophrenia-associated DMPs were relatively robust to the major PCs associated with methylomic variance in this dataset.

**Table 1:** Schizophrenia-associated differentially methylated positions

Probe ID	DNA methylation difference (%)	*P* value	Chromosome	Base position	Gene annotation	
cg10311104	0.80	9.75E−10	7	23053899	FAM126A	TSS200
cg08752433	2.78	1.05E−09	12	111016566	PPTC7	Body
cg26314722	1.85	1.73E−09	1	234867300		
cg24054898	1.64	5.91E−09	3	148721868	GYG1	Body
cg23684410	2.40	6.13E−09	11	116897558	SIK3	Body
cg00945209	1.91	9.66E−09	17	76801579	USP36	Body
cg18518074	2.29	1.03E−08	11	64642316	EHD1	Body
cg09706133	1.23	1.31E−08	15	68659758	ITGA11	Body
cg21522988	2.06	1.80E−08	12	29376872	FAR2	5′UTR
cg02656560	1.69	3.14E−08	17	19967600		
cg11418177	2.03	3.50E−08	4	142636072	IL15	5′UTR
cg06736148	2.14	3.68E−08	15	52416833	GNB5	Body
cg08655071	1.74	4.74E−08	1	209928895	TRAF3IP3	TSS1500
cg00829438	−1.02	5.26E−08	9	136213035	MED22	Body
cg27541604	1.65	5.57E−08	1	159046451	AIM2	5′UTR;1stExon
cg03149593	−1.24	5.98E−08	3	136988095		
cg14178364	1.44	6.43E−08	9	37529128	FBXO10	Body
cg14038731	1.42	6.70E−08	6	110732536	DDO	Body
cg20737259	−3.09	7.79E−08	4	95038723		
cg09470958	1.20	8.35E−08	6	31055471		
cg13803727	1.43	8.65E−08	9	89445247		
cg03402926	1.72	8.77E−08	11	27340767		
cg07326387	−2.10	9.16E−08	4	44543613		
cg00092992	1.80	9.69E−08	1	33596100		
cg03665078	1.58	9.98E−08	5	118689961	TNFAIP8	Body

Listed are all differentially methylated positions (DMPs) associated with schizophrenia (*P* < 1 × 10^−7^) with the corresponding *P* values and regression coefficients from the phase 1 discovery cohort. All DMPs with *P* < 5 × 10^−5^ are listed in Additional file 2: Table S3 with the corresponding *P* values and regression coefficients for the two independent replication cohorts

### Evidence of coordinated differential DNA methylation associated with schizophrenia across genomic regions

We next sought to identify extended regions characterized by schizophrenia-associated DNA methylation differences spanning multiple Illumina 450 K probes, implementing two methodological approaches to define differentially methylated regions (DMRs). First, we employed the *comb-p* algorithm that corrects the DMP *P* values for auto-correlation between probes and then scans the genome for peaks of association around a seed signal (set to *P* < 5 × 10^−5^) [28]. For each region it calculates the Stouffer–Liptak corrected *P* value, which is then adjusted for multiple testing using Šidák’s correction. This approach identified 12 significant schizophrenia-associated DMRs (Šidák-corrected *P* < 0.05) spanning between 2 and 20 DNA methylation sites (Additional file 2: Table S4). The top-ranked DMR identified using this approach spanned 20 CpG sites overlapping the major histocompatibility complex (MHC) on chromosome 6, noteworthy as it is the most robustly associated locus in schizophrenia GWAS [2, 29–32]. Second, in order to identify groups of sites that may not contain highly significant individual DMPs but are instead characterized by an extended region of contiguous differential DNA methylation associated with schizophrenia, we used a sliding window approach (see Methods) [33], using permutations to establish an appropriate multiple testing threshold (set at *P* < 3 × 10^−7^ for 5 % family-wise error). We identified 531 schizophrenia-associated regions, which were filtered to a set of 76 non-overlapping regions (Additional file 2: Table S5). Three of the 12 DMRs identified by *comb-p* were also identified as DMRs using this sliding window approach. The 76 DMRs contained between 2 and 120 probes (median 8.5), with the DMR *P* value not biased by the number of probes within each region (Additional file 1: Figure S8). Of note, 30 (36 %) of these genomic regions were not implicated by the probe-wise analysis, and for the majority (96 %) of regions, the DMR *P* values were more significant than the best individual probe *P* value, suggesting that there might be multiple semi-independent DMPs in these regions (Additional file 1: Figure S9). The top DMR (*P* = 1.87 × 10^−14^) identified using this approach spanned three probes within *GYG1* on chromosome 3 (Additional file 1: Figure S10), a gene previously shown to be differentially expressed in prefrontal pyramidal neurons from patients with schizophrenia [34].

### Schizophrenia-associated DMPs are enriched in transcription factor binding sites and in the vicinity of genes involved in immune-related pathways

We investigated whether the 1223 phase 1 discovery DMPs (*P* < 5 × 10^−5^) are enriched in specific regulatory domains identified in the ENCODE project [35, 36]. We found no significant enrichment of DMPs within DNAse I hypersensitivity sites (DHS) (Additional file 2: Table S6; *P* > 0.05) and a significant depletion of DMPs in the broad set of transcription factor binding sites [odds ratio (OR) = 0.852, *P* = 0.00542]. We identified a significant enrichment (*P* < 0.00338; corrected for 148 transcription factors) in certain specific transcription factor binding motifs including BATF (OR = 4.90, *P* = 5.04 × 10^−16^), BCL11A (OR = 3.48, *P* = 2.08 × 10^−7^), IRF4 (M-17) (OR = 2.20, *P* = 1.71 × 10^−5^), and MEF2A (OR = 2.13, *P* = 3.04 × 10^−5^), and a significant depletion in HA-E2F1 binding-sites (OR = 0.701, *P* = 0.000319). In order to investigate functional relationships between the 955 genes annotated to the 1223 phase 1 discovery DMPs, we tested for an over-representation of ontological categories and pathways using a method that controls for the number of probes annotated to each gene on the 450 K array (see Methods). Given the hierarchical structure of the ontological annotations, many of the significant terms are not independent and are associated by virtue of their overlapping membership; we therefore sought to group terms where the significant enrichment was explained by the overlap with a more significant term (see Methods), identifying 153 groups of related GO categories (Additional file 2: Table S7). The top-ranked group of pathways were related to immune function, consistent with findings from genetic [37], transcriptomic [38, 39], and epidemiological data [40, 41]. The second-ranked group of pathways were related to neuronal proliferation and brain development, an interesting observation given the hypothesized neurodevelopmental origins of schizophrenia [42].

### Replication of schizophrenia-associated DMPs in two independent cohorts

**Fig. 2:**
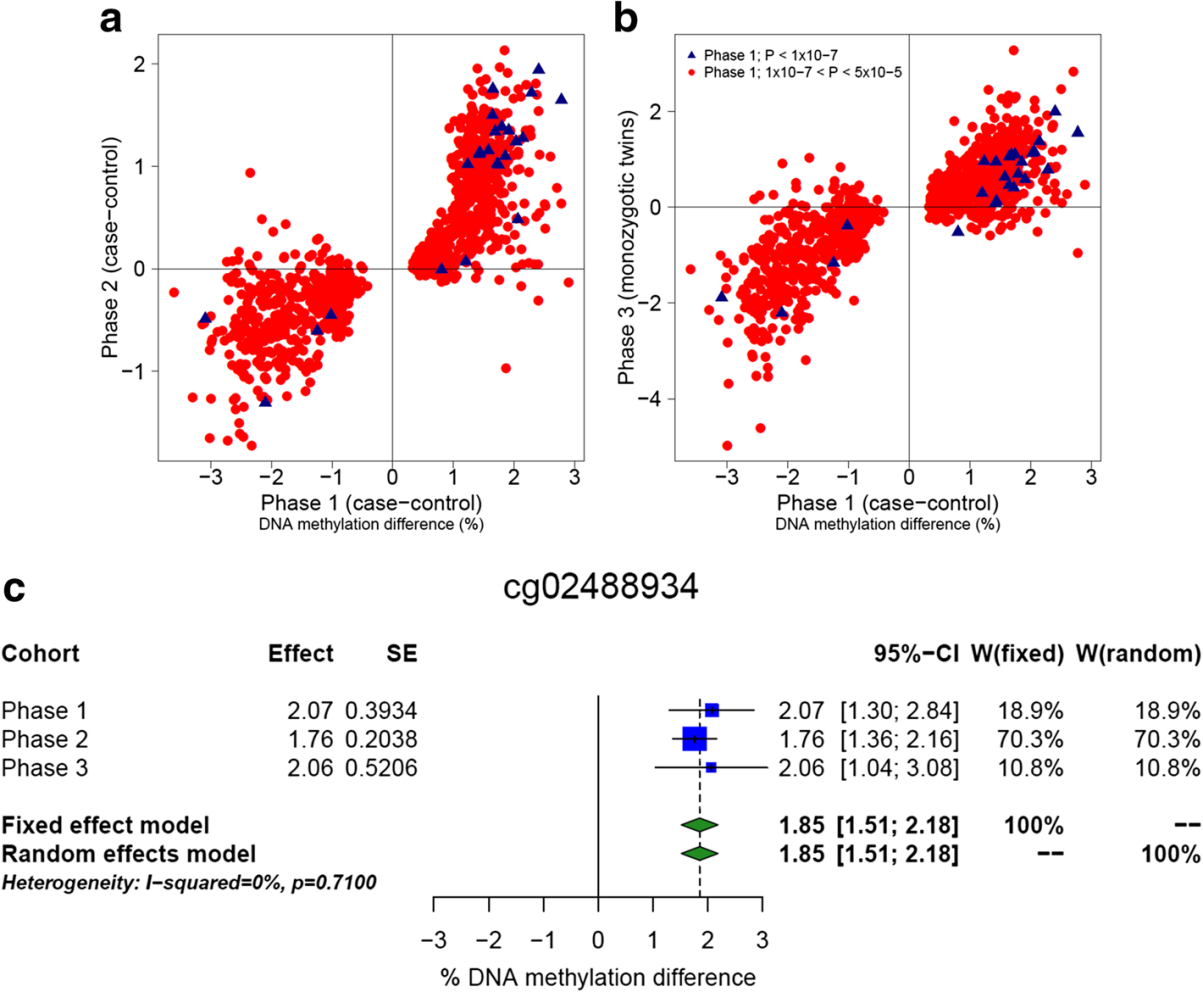
Replication of schizophrenia-associated DNA methylation differences identified in the phase 1 analysis in two independent cohorts. Shown are scatterplots demonstrating the concordance in effect size [schizophrenia-associated DNA methylation difference (%)] between the phase 1 (case–control) cohort (*n* = 675) (*x*-axis) and either the (**a**) phase 2 (case–control) cohort (*n* = 847) or (**b**) phase 3 (*n* = 96 monozygotic twin pairs) cohort for probes associated with schizophrenia at both the experiment-wide (*P* < 1 × 10^−7^; *red circles*) and discovery (*P* < 5 × 10^−5^; *blue triangles*) *P*-value thresholds. Many of these individual DNA methylation sites are significantly associated with schizophrenia in the replication cohorts (Table 1 and Additional file 2: Table S3). Meta-analyses across the three independent cohorts identified many additional sites significantly associated with schizophrenia at *P* < 1 × 10^−7^. (**c**) A forest plot of the top ranked probe from the meta-analysis (cg02488934), with the effect size and standard error (*SE*) from each individual cohort and the pooled effect from the meta-analysis. *CI* confidence interval

We next sought to confirm the identified schizophrenia-associated differences in an independent replication sample (phase 2) by generating 450 K array data from an additional 414 patients with schizophrenia and 433 non-psychiatric controls. As with the phase 1 cohort, patients with schizophrenia were characterized by a significantly higher smoking score derived from Illumina 450 K array DNA methylation data (Mann–Whitney *P* = 1.15 × 10^−22^; Additional file 1: Figure S3). We therefore employed an analysis model controlling for age, sex, batch, cell composition, and smoking to identify schizophrenia-associated differences at nominated DMPs. The 25 experiment-wide significant (*P* < 1 × 10^−7^) DMPs identified in phase 1 were characterized by highly consistent schizophrenia-associated differences in the same direction in the phase 2 dataset (sign test *P* = 7.75 × 10^−7^) (Fig. 2a); 14 of these DMPs were characterized by experiment-wide significant (*P* < 1 × 10^-7^) differences in the same direction in phase 2, with five additional DMPs significant at *P* < 5 × 10^−5^. The phase 1 discovery DMPs (*P* < 5 × 10^−5^) were also characterized by highly consistent schizophrenia-associated differences in phase 2, with 1159 (94.8 %) having a consistent direction of effect (sign test *P* = 4.25 × 10^−261^). Of the phase 1 DMPs, 137 (11.2 %), 249 (20.4 %), and 245 (20.0 %) were associated at *P* < 1 × 10^−7^, *P* < 5 × 10^−5^, and *P* < 4.09 × 10^−5^ (correcting for 1223 DMPs) respectively (Additional file 2: Table S3).

We next tested the schizophrenia-associated DMPs from phase 1 in a sample of 96 monozygotic twin pairs (phase 3); the analysis of MZ twins is a powerful tool in epigenetic epidemiology, as they do not differ for many of the confounders that can influence case–control analyses (e.g., age, sex, genotype) [9]. Although none of the top-ranked phase 1 DMPs (*P* < 1 × 10^−7^) reached experiment-wide significance in the twin dataset (minimum *P* = 1.11 × 10^−4^; Additional file 2: Table S3), this is not surprising given the relatively small number of twin pairs, and schizophrenia-associated differences were highly correlated with those identified in phase 1. Strikingly, 24 out of 25 (96 %) experiment-wide significant DMPs (sign test *P* = 7.75 × 10^−7^) and 1113 out of 1216 (91.5 %) phase 1 discovery DMPs (Fig. 2b; sign test *P* = 6.47 × 10^−215^) were characterized by schizophrenia-associated differences in the same direction, demonstrating that these effects were not confounded by factors such as genotype and sex that are perfectly matched between genetically identical twins. Finally, a meta-analysis across the three independent datasets demonstrated that 22 out of 25 (88 %) of the phase 1 experiment-wide significant DMPs were characterized by an experiment-wide significant association across all cohorts, with an additional 343 experiment-wide DMPs (*P* < 1 × 10^−7^) identified in the combined meta-analysis (Fig. 2c, Additional file 1: Figure S11, Additional file 2: Table S8, and Additional file 3).

### Differential DNA methylation associated with polygenic burden for schizophrenia

**Fig. 3:**
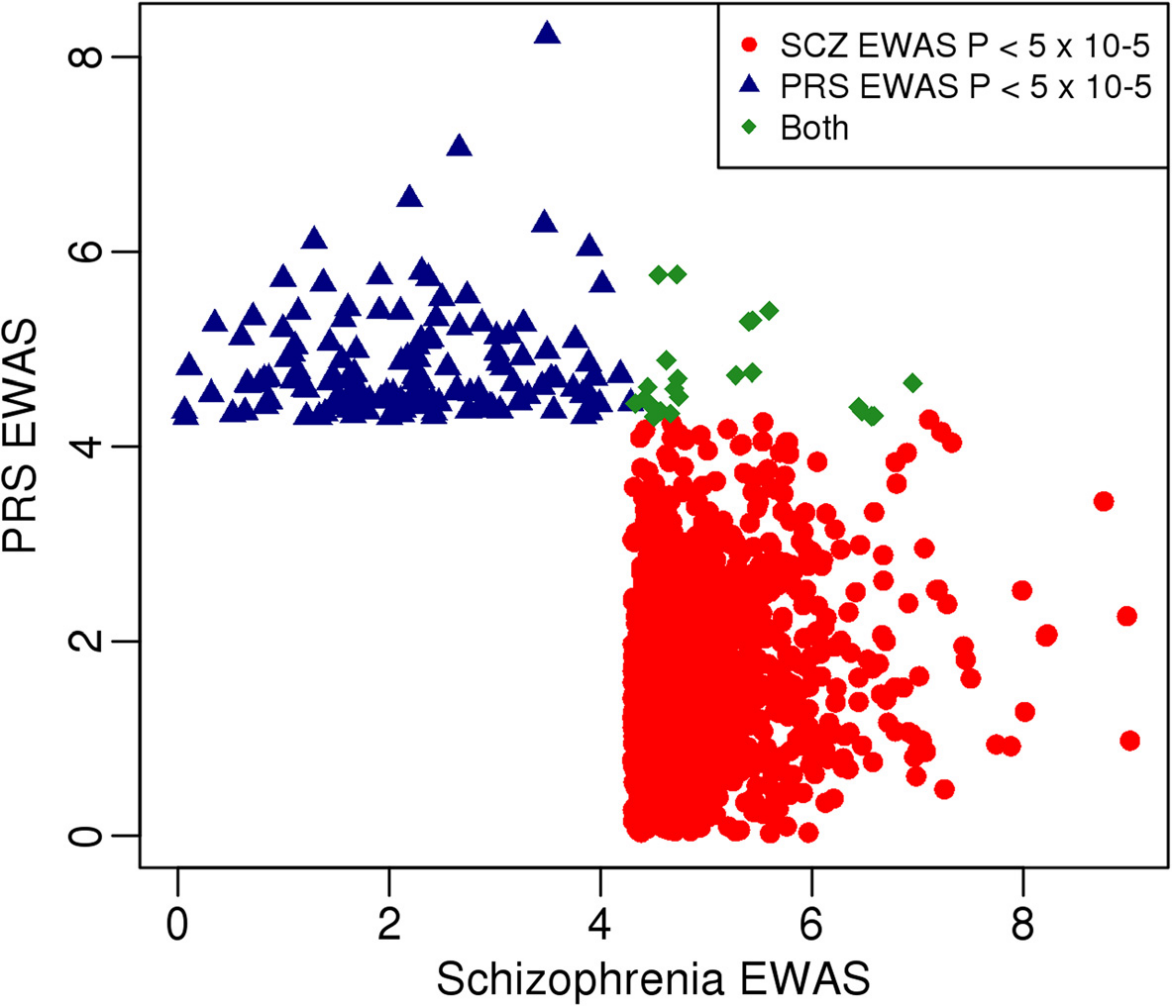
There is minimal overlap between significant schizophrenia-associated differentially methylated positions and those associated with the schizophrenia polygenic risk score (*PRS*). Shown is a scatterplot comparing probe-wise significance in the epigenome-wide association study (*EWAS*) of schizophrenia case status (*x*-axis) and PRS (*y*-axis). Data are presented for probes identified as significant (P < 5 × 10^−5^) in the schizophrenia EWAS (*red circles*), PRS EWAS (*blue triangles*), or both (*green diamonds*)

Many common DNA sequence variants, each conferring a small effect on susceptibility, mediate risk for schizophrenia [2, 29, 30, 43]. Beyond the specific genome-wide significant loci identified in GWAS, an individual’s accumulated genetic burden can be quantified to define an overall PRS – that is, the sum of trait-associated alleles across many genetic loci, weighted by effect sizes estimated by GWAS analyses [43]. It has been suggested that an individual’s PRS may function as a less confounded phenotype for molecular epidemiology, quantitatively indexing underlying neurobiological phenotypes associated with susceptibility. Schizophrenia PRSs were calculated for 639 samples in the phase 1 cohort based on genetic association data from the recent large PGC GWAS analysis of schizophrenia [2] (see Methods). As expected, patients with schizophrenia had a significantly higher PRS than control samples (*P* = 3.34 × 10^−27^; Additional file 1: Figure S12), confirming a higher polygenic burden of common risk variants in this group. We next performed an EWAS of the schizophrenia PRS, using a linear model controlling for the covariates of age, sex, and cell counts derived from the DNA methylation data, but not smoking status (see Methods). Unlike the analysis of schizophrenia diagnosis, we did not see an enrichment of smoking-associated DMPs (Additional file 1: Figures S13 and S14), indicating that increased smoking rates in schizophrenia might not result from the underlying common polygenic architecture of the disease. Performing a sensitivity analysis using PCs derived from the DNA methylation data iteratively (as described above) highlighted similar strong correlations (*r* = 0.963–0.980) with the effects identified in the initial EWAS (Additional file 1: Figure S15). Two DMPs were associated with schizophrenia PRS at our experiment-wide significance threshold (*P* < 1 × 10^–7^), with 156 DMPs identified at the more relaxed discovery threshold of *P* < 5 × 10^−5^ (Additional file 2: Table S9). Of note, the top-ranked disease- and PRS-associated DMPs are distinct, with no site reaching experiment-wide significance in both analyses (Fig. 3). Given our previous finding that there is an enrichment of mQTLs amongst SNPs associated with schizophrenia [18], we investigated whether any of the PRS-associated DMPs resulted directly from such genetic associations. Performing an mQTL analysis for all genetic variants incorporated in the PRS, we identified no overlap with DNA methylation sites associated with PRS. We next investigated PRS-associated DMPs in samples from our phase 2 replication cohort for whom genotype data were available (*n* = 843), in which patients with schizophrenia were again characterized by a significantly higher schizophrenia PRS than controls (*P* = 2.09 × 10^−31^; Additional file 1: Figure S12). Although none of the 156 DMPs reached experiment-wide significance in the phase 2 dataset (minimum *P* = 0.000121; Additional file 2: Table S9), effect sizes were again strongly correlated and there was a significant excess of consistent changes across both cohorts (123/156 sign test *P* = 1.05 × 10^−13^; Additional file 1: Figure S16).

### Differentially methylated sites overlap schizophrenia GWAS loci

**Fig. 4:**
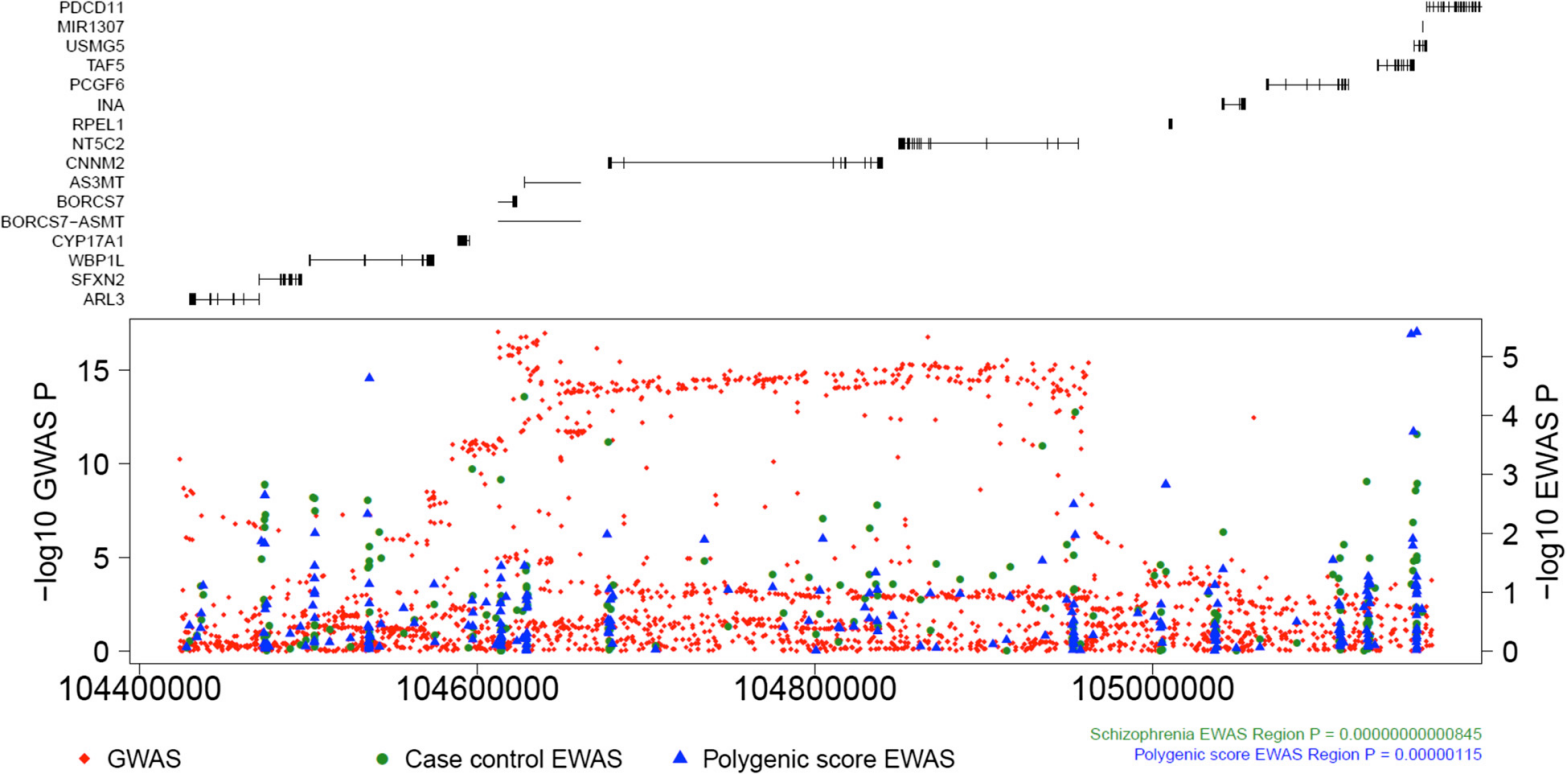
Evidence for schizophrenia-associated differential DNA methylation within genome-wide association study (*GWAS*)-nominated genomic regions. A Manhattan plot of an example genomic region (chr10:104423800-105165583) identified in the recent GWAS of schizophrenia [2] illustrates the location (*x*-axis) of genetic variants and Illumina 450 K probes against their significance with schizophrenia (*y*-axis; −log10 *P* value). Gene locations (exons and introns) are depicted above the Manhattan plot. *Red diamonds* depict GWAS results, *green circles* depict results from our phase 1 schizophrenia EWAS, and *blue triangles* depict results from our schizophrenia polygenic risk score EWAS

We next examined whether there is any overlap between the location of DMPs identified in this study and the 105 autosomal genomic regions nominated by the recent GWAS of schizophrenia [2]. These regions were derived by the PGC by “clumping” the GWAS *P* values so that multiple non-independent associations were collapsed into a single associated loci. Briefly, we generated a combined differential methylation *P* value from the individual probes, taking into account the correlation structure between them [33] (see Methods); 76 of the GWAS regions contained more than one 450 K array probe (median = 35, range = 2–504) and were appropriate for generating a combined *P* value. From these, we identified 27 regions (35.5 %) that demonstrated significant differences in DNA methylation (Bonferroni corrected threshold *P* < 0.000658; Additional file 2: Table S10) in schizophrenia samples compared to controls, of which nine were also characterized by a significant combined PRS EWAS *P* value. The top region is plotted in Fig. 4, highlighting multiple sites of differential DNA methylation across the whole LD block. Because these regions were larger than those considered for the sliding window approach, we generated empirical *P* values (see Methods) to confirm significant associations across 25 of the 27 schizophrenia-associated regions and four of nine PRS-associated regions. In all of these DMRs, the combined *P* value was more significant than the best DMP *P* value (Additional file 1: Figure S17), suggesting that there might be multiple semi-independently associated differentially methylated sites across these regions. Taken together, these results support previous findings that schizophrenia-associated DNA methylation differences overlap with genetic susceptibility loci [44, 45].

### Evidence that schizophrenia GWAS signals co-localize with mQTLs

**Fig. 5:**
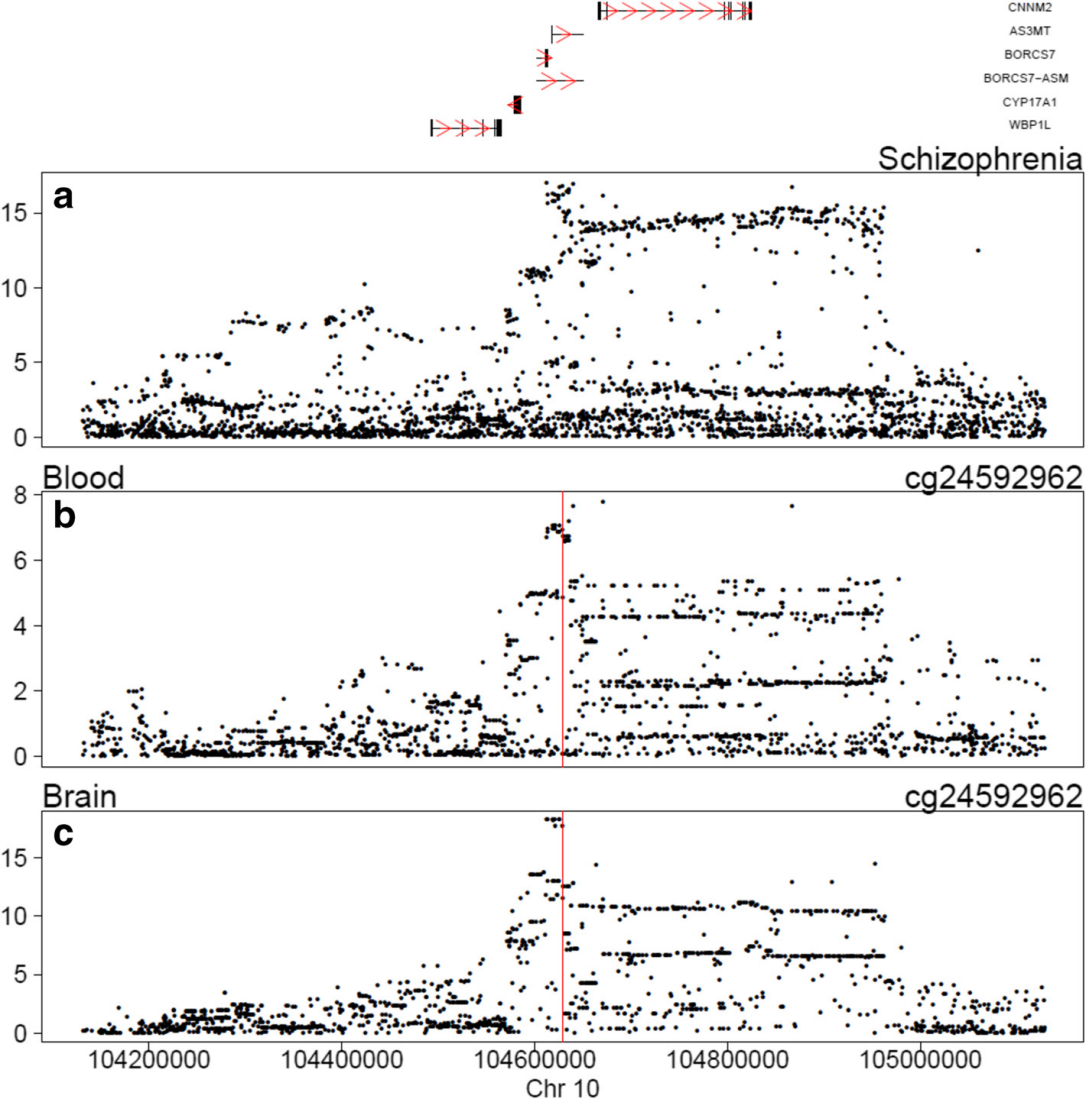
Co-localization of genome-wide association study (GWAS) signals associated with both schizophrenia and DNA methylation in blood and brain. Manhattan plots illustrate the location of genetic variants (*x*-axis) and their significance (*y*-axis; −log10 scale) in GWAS of schizophrenia (**a**), and DNA methylation at cg24592962 in blood (**b**) and brain (**c**). The *solid red line* indicates the location of the DNA methylation site (cg24592962). Comparing the pattern of these results for the mQTL and schizophrenia is supportive of the same causal variant being associated with both. Other examples are presented in Additional file 4

Although an enrichment of schizophrenia-associated DMPs in regions identified in GWAS is consistent with DNA methylation mediating the relationship between common risk variants and pathogenesis, this association does not establish a direct causal link. Motivated by this, we performed a Bayesian co-localization analysis [46] in phase 1 samples for which both genetic and DNA methylation data were available (*n* = 639). Briefly, this approach compares the pattern of association results from two independent GWAS (i.e. of schizophrenia and DNA methylation) to see if they are indexing an association with the same causal variant. We considered mQTL data for 23,649 unique Illumina 450 K probes located within 500 kb of the 105 autosomal GWAS-nominated regions defined by the PGC [2]. Because some probes were located in more than one GWAS region, we assessed a total of 23,918 potential mQTL pairs with schizophrenia. The posterior probabilities for 80 regions, involving DNA methylation sites in 1375 mQTL pairs, are supportive of a co-localized association signal for both schizophrenia and DNA methylation in that region (PP_3_ + PP_4_ > 0.99; Additional file 2: Table S11). Of these pairs, 127 (covering 39 regions associated with schizophrenia) had a higher posterior probability for both schizophrenia and DNA methylation, being associated with the same causal variant (PP_4_/PP_3_ > 1), with 66 (over 27 regions) of these having sufficient support for them to be considered as “convincing” (PP_4_/PP_3_ > 5) according to the criteria of Guo and colleagues [47]. We next compared these results to a similar analysis performed in a smaller sample of post-mortem brains [18]. Of 16 convincing pairs identified in brain, nine also had evidence of a co-localized association signal (PP_3_ + PP_4_ > 0.99) for both schizophrenia and DNA methylation in blood, seven (44 %) of which were also classed as demonstrating convincing co-localization with blood mQTLs (Table 2). One such example of this is shown in Fig. 5, highlighting a similar profile of GWAS *P* values across the region for schizophrenia and the mQTL in both blood and brain (additional examples are presented in Additional file 4).

**Table 2:** Convincing co-localization of schizophrenia and DNA methylation genome-wide association study signals in blood and brain

Schizophrenia GWAS region	Probe ID	Chr	Base position	Gene annotation	Bayesian co-localization					
					mQTL in blood			mQTL in brain		
					nsnps	PP3 + PP4	PP4/PP3	nsnps	PP3 + PP4	PP4/PP3
108	cg00585072	5	140186983	PCDHA2;PCDHA1;PCDHA4;PCDHA3	1595	0.9982	5.340	1260	0.9983	6.058
7	cg02951883	7	2050386	MAD1L1	1808	1.0000	14.596	2144	0.9992	11.820
3	cg08772003	10	104629869	AS3MT	1994	1.0000	51.075	1316	1.0000	36.981
3	cg11784071	10	104629166	AS3MT	1994	0.9999	8.046	1315	1.0000	39.298
3	cg24592962	10	104629151	AS3MT	1994	0.9986	36.510	1315	1.0000	55.055
99	cg14258853	12	29935411	TMTC1	3551	0.9995	46.932	2598	0.9989	10.723
47	cg26732615	19	19648335	CILP2;YJEFN3	1644	1.0000	8.196	1285	0.9906	5.514

Listed are all instances where the GWAS results indicate that the same causal variant is associated with schizophrenia and DNA methylation at a specific site in both blood and brain. Bayesian co-localization analysis compared the GWAS results evaluating the evidence for five hypotheses (see Methods); convincing co-localization signs were defined as PP3 + PP4 > 0.99 and PP4/PP3 > 5. The full set of results for all genetic loci associated with schizophrenia can be found in Additional file 2: Table S12.

*Chr* chromosome, *GWAS* genome-wide association study; *mQTL* DNA methylation quantitative trait loci; *nsnps *number of SNPs

## Conclusions

This study is the first systematic integrated analysis of genetic and epigenetic variation in schizophrenia, introducing a methodological pipeline that can be used to inform EWAS analyses of other traits and diseases. We have identified multiple DMPs and DMRs associated with schizophrenia, independently of important confounders such as smoking, with striking levels of replication in independent sample cohorts. We also show that polygenic burden for schizophrenia is associated with epigenetic variation across the genome, independently of loci implicated in the analysis of diagnosed schizophrenia. Finally, we have used mQTL analyses to annotate the extended genomic regions nominated by GWAS analyses of schizophrenia, using co-localization analyses to highlight potential regulatory variation causally involved in disease.

## Methods

All experimental methods were in accordance with the Helsinki declaration.

### Cohort description: phase 1- University College London

The University College London case–control sample has been described elsewhere [48] but briefly comprises of unrelated ancestrally matched cases and controls from the UK. Case participants were recruited from UK National Health Service (NHS) mental health services with a clinical International Classification of Diseases 10th edition (ICD-10) diagnosis of schizophrenia. All case participants were interviewed with the Schedule for Affective Disorders and Schizophrenia-Lifetime Version (SADS-L) [49] to confirm Research Diagnostic Criteria (RDC) diagnosis. A control sample screened for an absence of mental health problems was recruited. Each control subject was interviewed to confirm that they did not have a personal history of an RDC-defined mental disorder or a family history of schizophrenia, bipolar disorder, or alcohol dependence. UK NHS multicenter and local research ethics approval was obtained and all participants signed an approved consent form after reading an information sheet.

### Cohort description: phase 2 – Aberdeen

The Aberdeen case–control sample has been described elsewhere [50] but briefly contains patients with schizophrenia and controls who have self-identified as born in the British Isles (95 % in Scotland). All cases met the Diagnostic and Statistical Manual for Mental Disorders fourth edition (DSM-IV) and ICD-10 criteria for schizophrenia. Diagnosis was made by Operational Criteria Checklist (OPCRIT). All case participants were outpatients or stable inpatients. Detailed medical and psychiatric histories were collected. A clinical interview using the Structured Clinical Interview for DSM-IV (SCID) was also performed on schizophrenia cases. Controls were volunteers recruited through general practices in Scotland. Practice lists were screened for potentially suitable volunteers by age and sex and by exclusion of individuals with major mental illness or use of neuroleptic medication. Volunteers who replied to a written invitation were interviewed using a short questionnaire to exclude major mental illness in the individual themselves and their first-degree relatives. All cases and controls gave informed consent. The study was approved by both local and multiregional academic ethical committees.

### Cohort description: phase 3 – monozygotic twins

The MZ twin cohort is a multi-center collaborative project aimed at identifying DNA methylation differences in MZ twin pairs discordant for schizophrenia. We identified 96 informative twin-pairs (*n* = 192 individuals) from European twin studies based in Utrecht (The Netherlands), Helsinki (Finland), London (UK), Stockholm (Sweden), and Jena (Germany). Of the MZ twin pairs utilized in the analysis, 75 were discordant for diagnosed schizophrenia, six were concordant for schizophrenia, and 15 twin pairs were free of any psychiatric disease. In this analysis we tested specific DNA methylation probes nominated from our case–control analysis; a more detailed description of the cohort along with more in-depth analysis is currently under preparation (Dempster et al., in preparation).

### Genome-wide quantification of DNA methylation

The EZ-96 DNA Methylation kit (Zymo Research, CA, USA) was used to treat 500 ng of DNA from each sample with sodium bisulfite in duplicate. DNA methylation was quantified using the Illumina Infinium HumanMethylation450 BeadChip (Illumina Inc.) run on an Illumina iScan System (Illumina) using the manufacturers’ standard protocol. Samples were randomly assigned to chips and plates to ensure equal distribution of cases and controls across arrays and to minimize batch effects. In addition, a fully methylated control (CpG Methylated HeLa Genomic DNA; New England BioLabs, MA, USA) was included in a random position on each plate.

Signal intensities were imported in the R programming environment using the *methylumIDAT*() function in the methylumi package [51]. Our stringent quality control pipeline included the following steps: (1) checking methylated and unmethylated signal intensities, excluding samples where this was <2500; (2) using the 10 control probes to ensure the bisulfite conversion was successful, excluding any samples with median <90; (3) identifying the fully methylated control sample was in the correct location; (4) all tissues predicted as of blood origin using the tissue prediction from the Epigenetic Clock software (https://dnamage.genetics.ucla.edu/) [21]; (5) multidimensional scaling of sites on X and Y chromosomes separately to confirm reported gender; (6) comparison of genotype data for up to 65 single nucleotide polymorphism (SNP) probes on 450 K array; and (7) use of the *pfilter*() function from wateRmelon package [52] to exclude samples with >1 % of probes with detection *P* value > 0.05 and probes with >1 % of samples with detection *P* value > 0.05. PCs were used (calculated across all probes) to identify outliers, samples >2 standard deviations from the mean for both PC1 and PC2 were removed. Finally, we checked the correlation (*r* = 0.927) of reported age with that predicted by the Epigenetic Clock. Normalization of the DNA methylation data was performed used the *dasen*() function in the *wateRmelon* package [52]. Due to a different experimental design, the phase 3 cohort was performed so that both members of each MZ twin pair were run on the same chip. Data processing followed a similar pipeline with an additional step using the 65 SNP probes to confirm that twins were genetically identical.

### Genotyping

Genotyping was performed using the Affymetrix Mapping 500 K Array and the Genomewide Human SNP Array 5.0 or 6.0 (Affymetrix, CA, USA). Genotypes were called from raw intensity data using the Birdseed component of the Birdsuite algorithm [53, 54]. Samples were genotyped by the Genetic Analysis Platform at The Broad Institute of Harvard and MIT according to standard protocols.

### Imputation

Prior to imputation, PLINK [55] was used to remove samples with >5 % missing data. We also excluded SNPs characterized by >5 % missing values, a Hardy–Weinberg equilibrium *P* value < 0.001 and a minor allele frequency of <5 %. Imputation was performed using ChunkChromosome (http://genome.sph.umich.edu/wiki/ChunkChromosome) and Minimac2 [56, 57] with the 1000 Genomes reference panel of European samples (phase 1, version 3). Imputed genotypes were then converted back in the PLINK format files using fcgene [58] only including variants with Rsq > 0.3. SNPs were then refiltered with PLINK such that they satified the criteria: <1 % missing values, Hardy–Weinburg equilibrium *P*-value < 0.001, and a minor allele frequency of >5 %. Subsequently, SNPs were also filtered so that each of the three genotype groups with zero, one, or two minor alleles (or two genotype groups in the case of rare SNPs with zero or one minor allele) had a minimum of five observations.

### DNA methylation smoking score

As smoking status information was not present for all samples, we estimated a proxy based on the DNA methylation profile at sites known to be associated with smoking status following the approach in [22]. This methodology produces a weighted score across 183 DNA methylation sites, where the weights were taken from the smoking EWAS in [23].

### Polygenic risk scores

As samples from both phase 1 and phase 2 were included in the PGC GWAS of schizophrenia, we obtained the PRS scores from these analyses calculated as part of the leave one out validation experiment, where the training dataset (to derive weights for associated scores) was based on all samples bar one source dataset in which the PRSs were calculated [2]. In this analysis we used the scores calculated across an independent set of variants with *P* value < 0.05.

### Statistical analysis

Probes previously identified as containing a common SNP (allele frequency > 5 % in European populations) within 10 base pairs (bp) of the single base extension position [59] or potentially cross-hybridizing to multiple genomic locations [59, 60] were removed prior to analysis. A linear regression model was used to test for differentially methylated sites associated with schizophrenia. DNA methylation values for each probe were regressed against case–control status with covariates for age, gender, and cell composition. As cell count data were not available for these DNA samples, these were estimated from the DNA methylation data using both the Epigenetic Clock software [21] and Houseman algorithm [19, 20], including the seven variables recommended in the documentation for the Epigenetic Clock in the regression analysis. Additional regression models including smoking score and principal components, also derived from DNA methylation, were also performed. For the twins a linear model was used to generate regression coefficients, but clustered standard errors using the plm package [61] – recognizing individuals from the same twin pair – were used to calculate *P*-values. DMP results are annotated with their genomic location and gene annotation taken from the annotation files provided by Illumina. In addition, transcription factor binding site and DHS site annotation were taken from the supplementary files provided by Slieker and colleagues [36].

### Multiple testing threshold

To establish the multiple testing significance threshold, 5000 permutations were performed repeating the linear regression model for randomly selected groups of cases and controls to match the numbers in the phase 1 data. For each permutation, *P* values from the EWAS were saved and the minimum identified. Across all permutations the fifth percentile was calculated to generate the 5 % alpha significance threshold.

### Regional analysis

Two different region approaches were used. First, the results for every probe were converted into a BED file (containing genomic location and EWAS *P* value) and run through the *comb-p* [28] pipeline with a seed of 5 × 10^−5^ and distance parameter set to 500 bp. Briefly, *comb-p* generates DMRs by (1) calculating the auto-correlation between probes to adjust the input DMP *P*-values using the Stouffer–Liptak–Kechris correction, (2) running a peak finding algorithm over these adjusted *P* values to identify enriched regions around a seed signal, (3) calculating the region *P* value using the Stouffer–Liptak correction, and (4) correcting for multiple testing with the one-step Šidák correction. Significant regions were identified as those with at least two probes and a corrected *P* value < 0.05.

Second, we implemented a sliding window approach with multiple window sizes. Previous EWAS have reported DMRs that span either a few hundred or a few thousand base pairs [62]. As is it unlikely that every DMR will contain exactly the same number of probes or have the same genomic span, partly due to the irregular distribution of 450 K probes across the genome [63], multiple window sizes were used (100, 200, 500, 1000, 2000, 5000 bp). For each window a combined *P* value was calculated from the individual DMP *P* values contained, taking into account the correlation between probes [33]. Each probe on the 450 K array was considered and all probes within the window extended in both directions were collated. The correlation coefficients between each pair of probes in the window and *P* values from the EWAS were combined using Brown’s method for combining non-independent test statistics [33]. To derive an appropriate multiple testing threshold (based on 5 % family-wise error), we repeated this procedure on the results of the randomly permuted EWASs separately for each sized window, identified the minimum region *P* value for each permutation, and calculated the fifth percentile. The set of significant regions was then reduced into the best non-overlapping set by ranking all regions by their *P* value, retaining the most significant, and removing any that overlapped (defined as both regions containing any common probes), before moving to the next most significant region, until the bottom of the list was reached.

### Enrichment of regulatory regions

Published 450 K array probe annotations [36] were used to identify probes located in transcription factor binding sites or DHSs based on data made publically available as part of the ENCODE project [3, 35]. The overlap between regulatory features and DMPs was tested for enrichment using a two-sided Fisher’s 2 × 2 exact test. The significance level for enrichment of overlap with transcription factor binding sites was calculated using a Bonferroni correction for the 148 different transcription factor binding sites tested.

### Gene ontology analysis

Illumina UCSC gene annotation, which is derived from the genomic overlap of probes with RefSeq genes or up to 1500 bp of the transcription start site of a gene, was used to create a test gene list from the DMPs for pathway analysis. Where probes were not annotated to any gene (i.e. in the case of intergenic locations), they were omitted from this analysis; where probes were annotated to multiple genes, all were included. A logistic regression approach was used to test if genes in this list predicted pathway membership, while controlling for the number of probes that passed quality control (i.e., were tested) annotated to each gene. Pathways were downloaded from the GO website (http://geneontology.org/) and mapped to genes, including all parent ontology terms. All genes with at least one 450 K probe annotated and mapped to at least one GO pathway were considered. Pathways were filtered to those containing between 10 and 2000 genes. After applying this method to all pathways, the list of significant pathways (*P* < 0.05) was refined by grouping to control for the effect of overlapping genes. This was achieved by taking the most significant pathway, and retesting all remaining significant pathways while controlling additionally for the best term. If the test genes no longer predicted the pathway, the term was said to be explained by the more significant pathway, and hence these pathways were grouped together. This algorithm was repeated, taking the next most significant term, until all pathways were considered as the most significant or found to be explained by a more significant term.

### Meta-analysis

All probes with *P* value < 5 × 10^−5^ in the phase 1 EWAS were considered for a meta-analysis with phase 2 and phase 3 for case–control analysis only. This was performed using the *metagen*() function in the R package meta [64], providing the regression coefficients and standard errors from each individual cohort to calculate weighted pooled estimates and to test for significance. Results from both fixed and random effects models are reported in Additional file 2: Tables S8 and S10; however, we only considered those from the fixed effect model because, with only two or three cohorts, estimates of heterogeneity are poor.

### Overlap with schizophrenia GWAS loci

The GWAS regions were defined by the PGC in their original manuscript [2] and are available for download from the PGC website (https://www.med.unc.edu/pgc/results-and-downloads). Briefly, these were identified by performing a “clumping” procedure on the GWAS *P* values to collapse multiple correlated signals (due to LD) surrounding the index SNP (i.e., with the smallest *P* value) into a single associated region. To define 108 physically distinct loci, those within 250 kb of each other were subsequently merged to obtain the final set of GWAS regions. The outermost SNPs of each associated region define the start and stop parameters of the region. Using the set of 105 autosomal schizophrenia-associated genomic loci we used Brown’s method [33] to calculate a combined *P* value across all 450 K probes located within each region. This used the *P* values from both the case–control and PRS EWAS and correlation coefficients between all pairs of probes calculated from the DNA methylation values. This methodology was repeated with the 5000 random permutations we generated. Empirical *P* values for each region were calculated by counting how many of the permutations had more significant *P* values than the true combined *P* value and dividing by the total number of permutations performed.

### Co-localization analyses

Schizophrenia-associated genomic loci were taken as the 105 autosomal regions published as part of the PGC mega-analysis [2]. Given our definition of *cis* mQTLs (i.e., associations between SNPs and DNA methylation probes within 500 kb), all DNA methylation sites located within 500 kb of these regions were identified and *cis* mQTL analysis was performed using MatrixEQTL [65]. An additive linear model was fitted to test if the number of alleles (coded 0, 1, or 2) predicted DNA methylation (beta value 0–100) at each site, including covariates for age, sex, and the first two PCs from the genotype data.

Co-localization analysis was performed as previously described [46] using the R coloc package (http://cran.r-project.org/web/packages/coloc) for each DNA methylation site within each region. From both the PGC schizophrenia GWAS data and our mQTL results we inputted the regression coefficients, their variances, and the SNP minor allele frequencies, and the prior probabilities were left as their default values. This methodology quantifies the support across the results of each GWAS for five hypotheses by calculating the posterior probabilities, denoted as *PPi* for hypothesis *Hi*:

- *H*_*0*_*: there exist no causal variants for either trait;*
- *H*_*1*_*: there exists a causal variant for one trait only, schizophrenia;*
- *H*_*2*_*: there exists a causal variant for one trait only, DNA methylation;*
- *H*_*3*_*: there exist two distinct causal variants, one for each trait;*
- *H*_*4*_*: there exists a single causal variant common to both traits.*

## Abbreviations

450K array, Illumina Infinium HumanMethylation450 BeadChip; bp, Base pair; DHS, DNAse I hypersensitivity site; DMP, Differentially methylated positions; DMR, Differentially methylated region; DSM-IV, Diagnostic and Statistical Manual for Mental Disorders fourth edition; EWAS, Epigenome-wide association study; GO, Gene Ontology; GWAS, Genome-wide association study; ICD-10, International Classification of Diseases 10th edition; LD, linkage disequilibrium; MHC, Major histocompatibility complex; mQTL, DNA methylation quantitative trait loci; MZ, Monozygotic; OPCRIT, Operational Criteria Checklist; OR, Odds ratio; PC, Principal component; PGC, Psychiatric Genomics Consortium; PRS, Polygenic risk score; RDC, Research diagnostic criteria; SADS-L, Schedule for Affective Disorders and Schizophrenia-Lifetime Version; SCID, Structured Clinical Interview for DSM-IV; SNP, single nucleotide polymorphism

## Additional files

Additional file 1: Figures S1–S17. (PDF 1469 kb) Additional file 2: Supplementary Tables S1-S11. (XLSX 6.83 mb) Additional file 3: Forest plots from case–control meta-analyses. (PDF 462 kb) Additional file 4: Examples of co-localization between variants associated with schizophrenia and DNA methylation. (PDF 335 kb) Additional file 5: The DNA methylation values for the specific probes used in the MZ twin replication analysis. (XLSX 3313 kb) Additional file 6: The phenotype data for the samples used in the MZ twin replication analysis. (XLSX 16 kb)
