# Supplementary material for: An integrated genetic-epigenetic analysis of schizophrenia: evidence for co-localization of genetic associations and differential DNA methylation
Source: Genome Biol. 2016 Aug 30;17(1):176. doi: 10.1186/s13059-016-1041-x (PMC5004279; doi:10.1186/s13059-016-1041-x)
Supplement: Additional file 1: Figures S1–S17. — (PDF 1469 kb) [file 13059_2016_1041_MOESM1_ESM.pdf]

Additional File 1

Figure S1 Overview of the methodological approach used in this study. Blue boxes indicate analyses performed as part of this study; red boxes identify results taken from publicly available databases. Abbreviations: DMP – differentially methylated position, DMR – differentially methylated region, PRS – polygenic risk score, GWAS – genome-wide association study, EWAS – epigenome-wide association study.

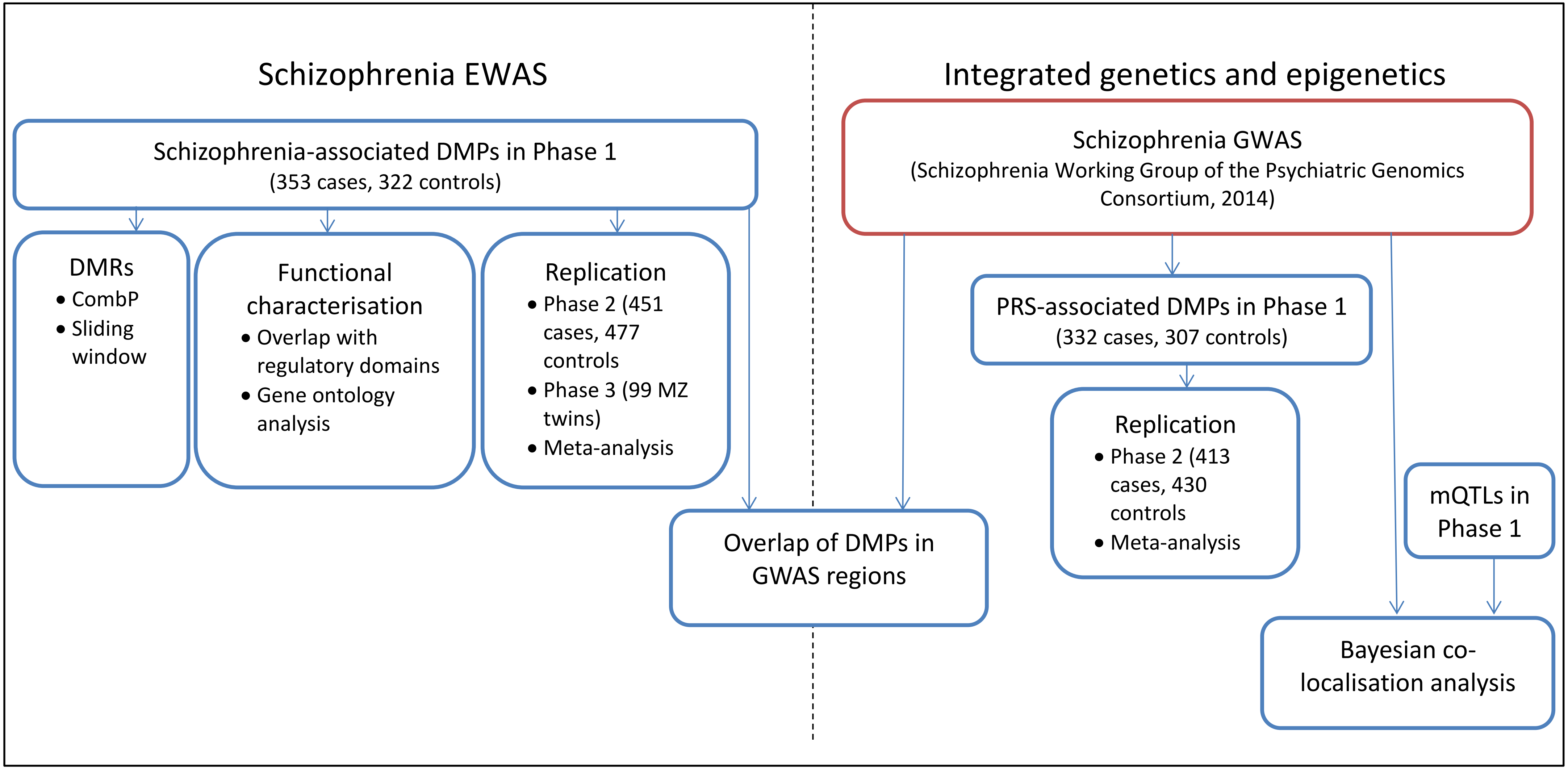

Figure S2 Validation of smoking score derived from DNA methylation data. Actual current smoking status data was only available for a small number of individuals in phase 1 (non-smoker: n = 4; smoker: n = 14), but confirmed the validity of the proxy smoking score generated from the results previously published EWAS of cigarette smoking [1, 2].

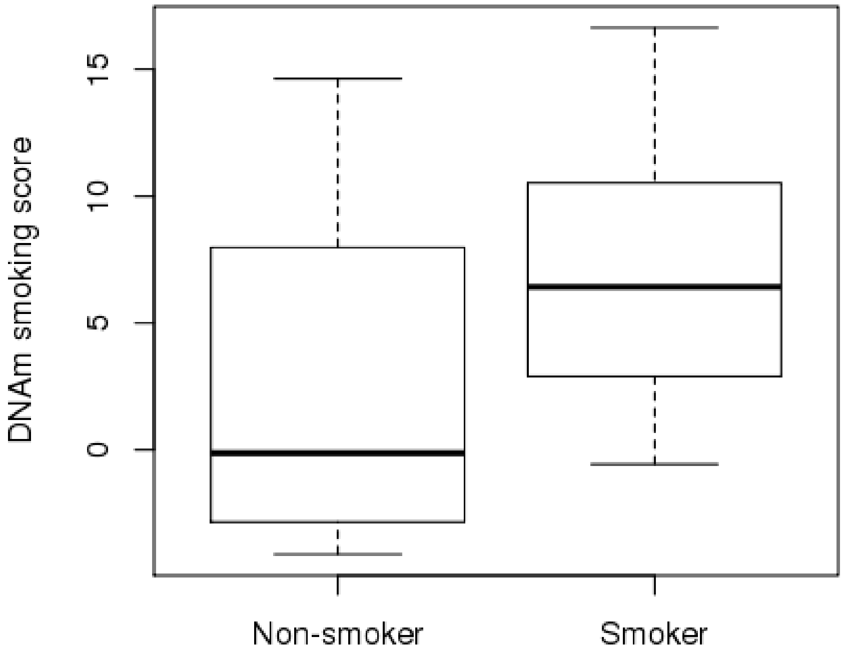

Figure S3 Density plot of DNA methylation smoking score split by case status. Using the previously published results from an EWAS of cigarette smoking [1, 2], we calculated a weighted score from the DNA methylation data that captures smoking behavior. This figure shows the distribution of these scores split into cases and controls for samples from a) phase 1 (353 cases, 322 controls) and b) phase 2 (451 cases, 477 controls). The profiles are consistent with epidemiological reports of elevated smoking amongst schizophrenia patients (phase 1: Mann-Whitney P = 1.51×10^{-41}; phase 2: Mann-Whitney P = 1.15×10^{-22}).

a)

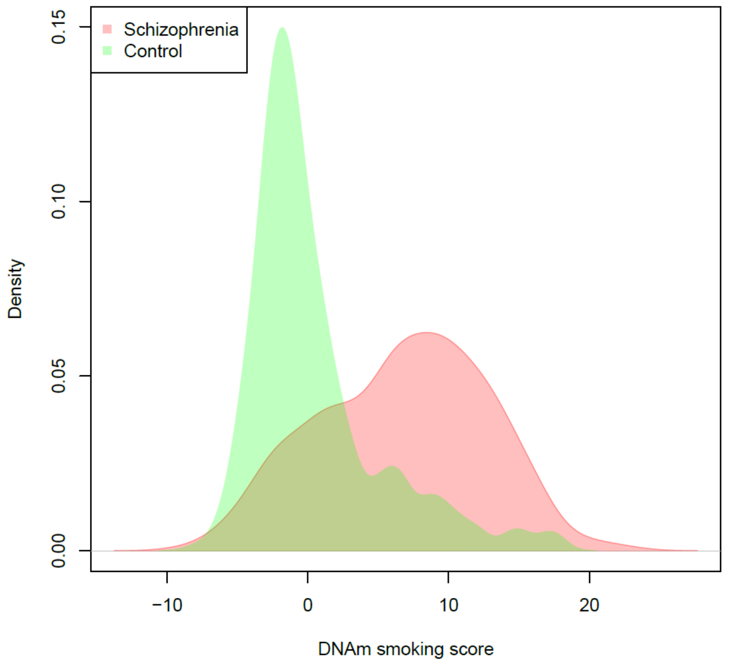

b)

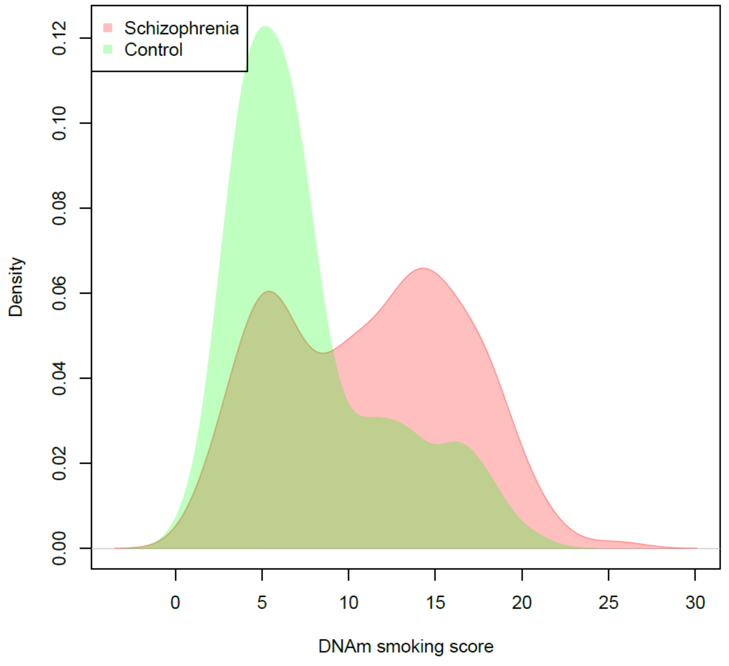

Figure S4 The confounding effects of smoking in EWAS analyses of schizophrenia can be controlled for using a smoking score derived from DNA methylation data. Scatterplots of probes significantly associated with schizophrenia a) not controlling for derived smoking status (160 probes with $P < 1x10^{-7}$) and b) controlling for derived smoking status. Presented for each probe is the signed log10 P value from the EWAS of schizophrenia (x-axis) and corresponding signed log10 P values from a published EWAS of smoking (y-axis; current vs never) [1].

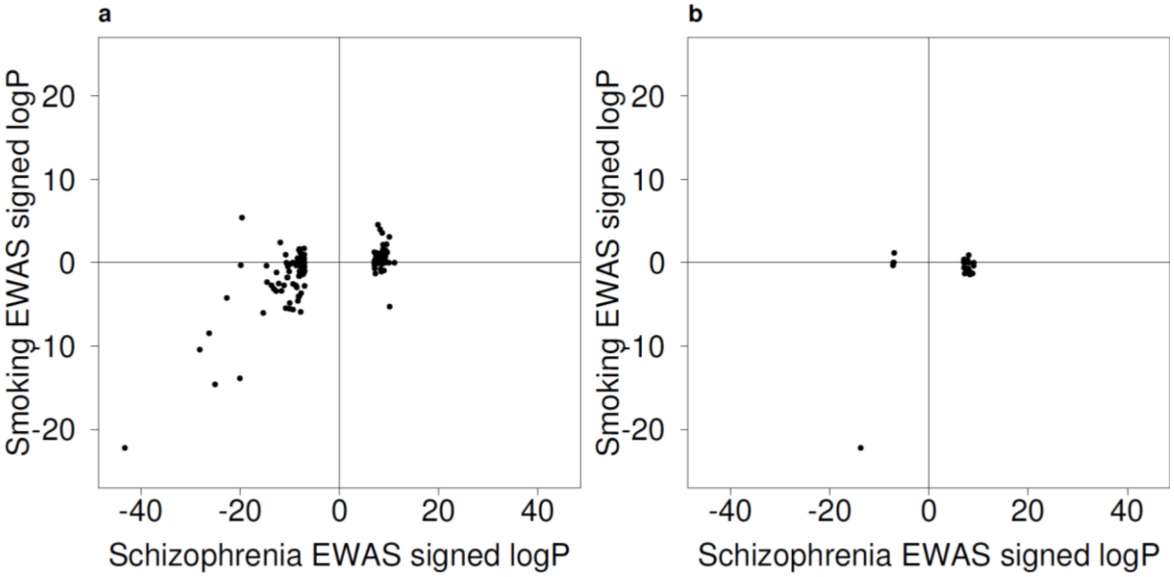

Supplementary Figure S5: Box-plots showing differences in DNA methylation between schizophrenia cases (n = 353) and non-psychiatric controls (n = 322) at the 10 top-ranked DMPs.

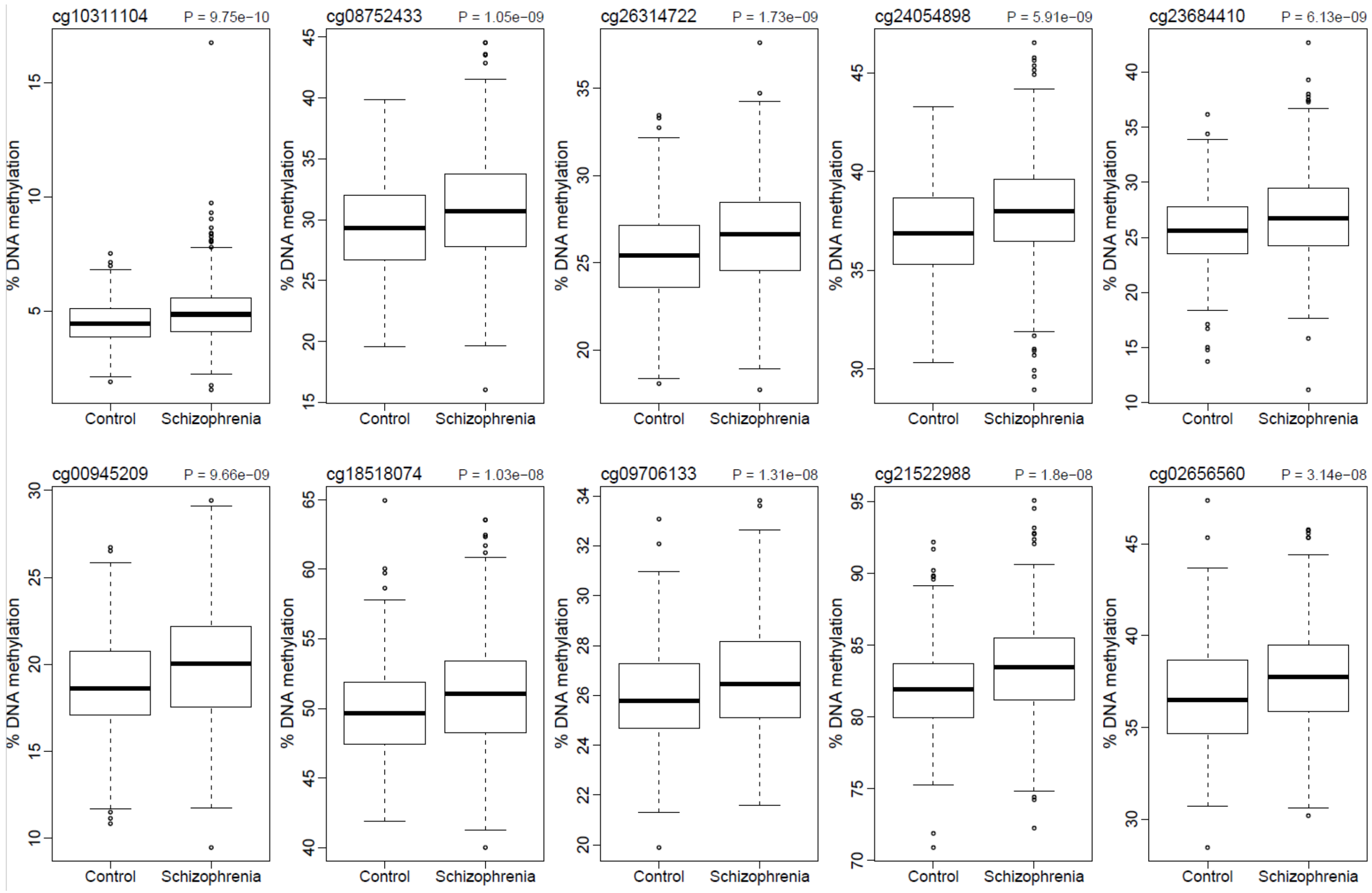

Supplementary Figure S6: Relationship of the top 10 principal components (PCs) derived from DNA methylation data with available and derived covariates. Heat-map of correlations between each PC (1-10) and the available phenotype information (SCZ – schizophrenia status; sex) and variables derived from the DNA methylation data (DNAmAge – age estimated from DNA methylation data; DNAm Smoking - smoking score estimated from DNA methylation data; PlasmaBlast, CD8pCD28nCD45RAn, CD8.naive, CD4.naive - cellular abundance estimates from epigenetic clock software[3]; CD8T, CD4T, NK, Bcell, Mono, Gran - cellular proportion estimates from Houseman algorithm[4, 5]).

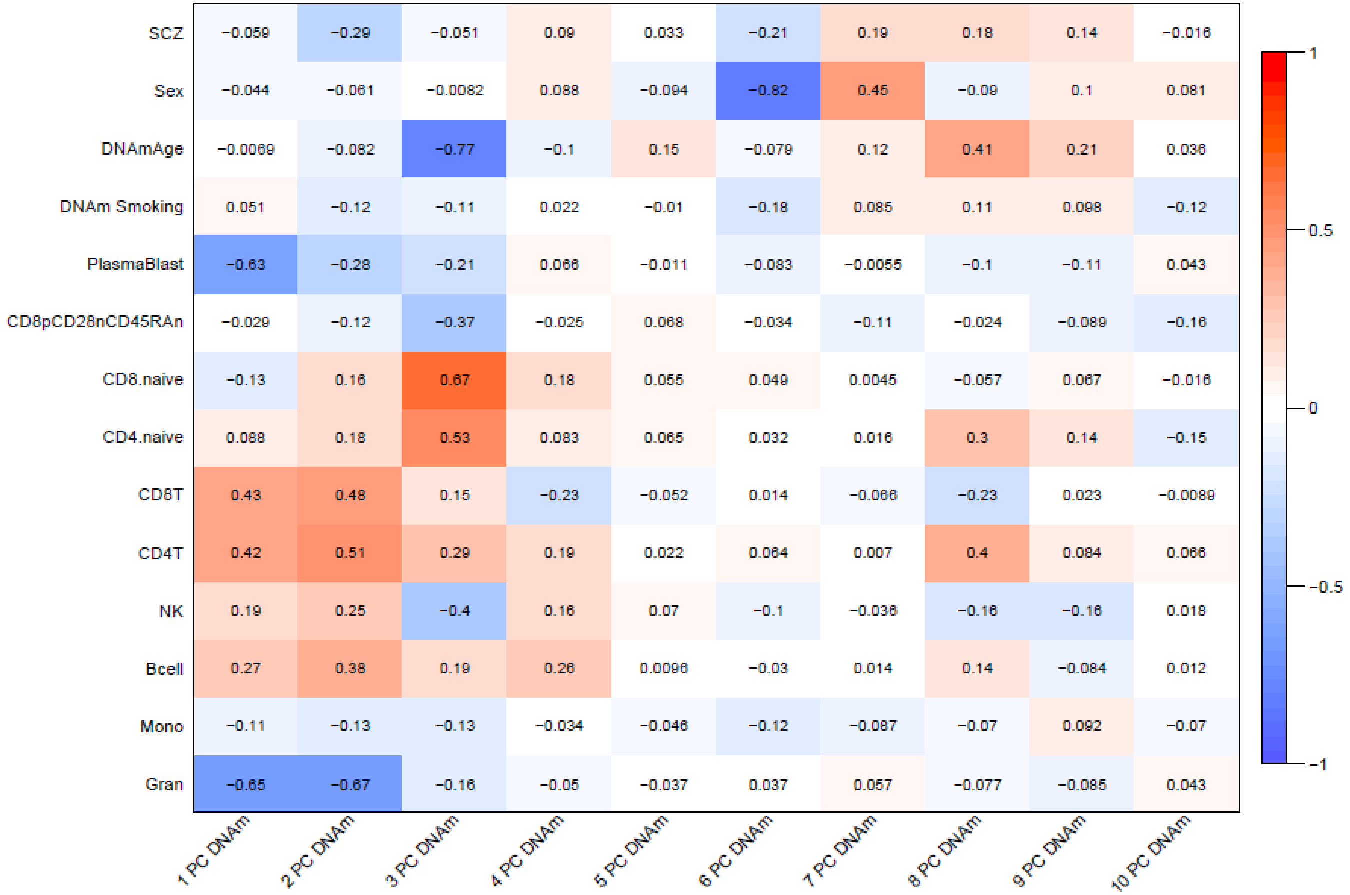

Supplementary Figure S7: Schizophrenia-associated DNA methylation differences are robust to the addition of PCs capturing variation in DNA methylation data. Shown for schizophrenia-associated DMPs ($P < 5x10^{-5}$) are DNA methylation differences between cases and controls in EWAS unadjusted for PCs (x-axis) against EWAS iteratively including additional PCs (y-axis). Points are colored by their significance in the original EWAS.

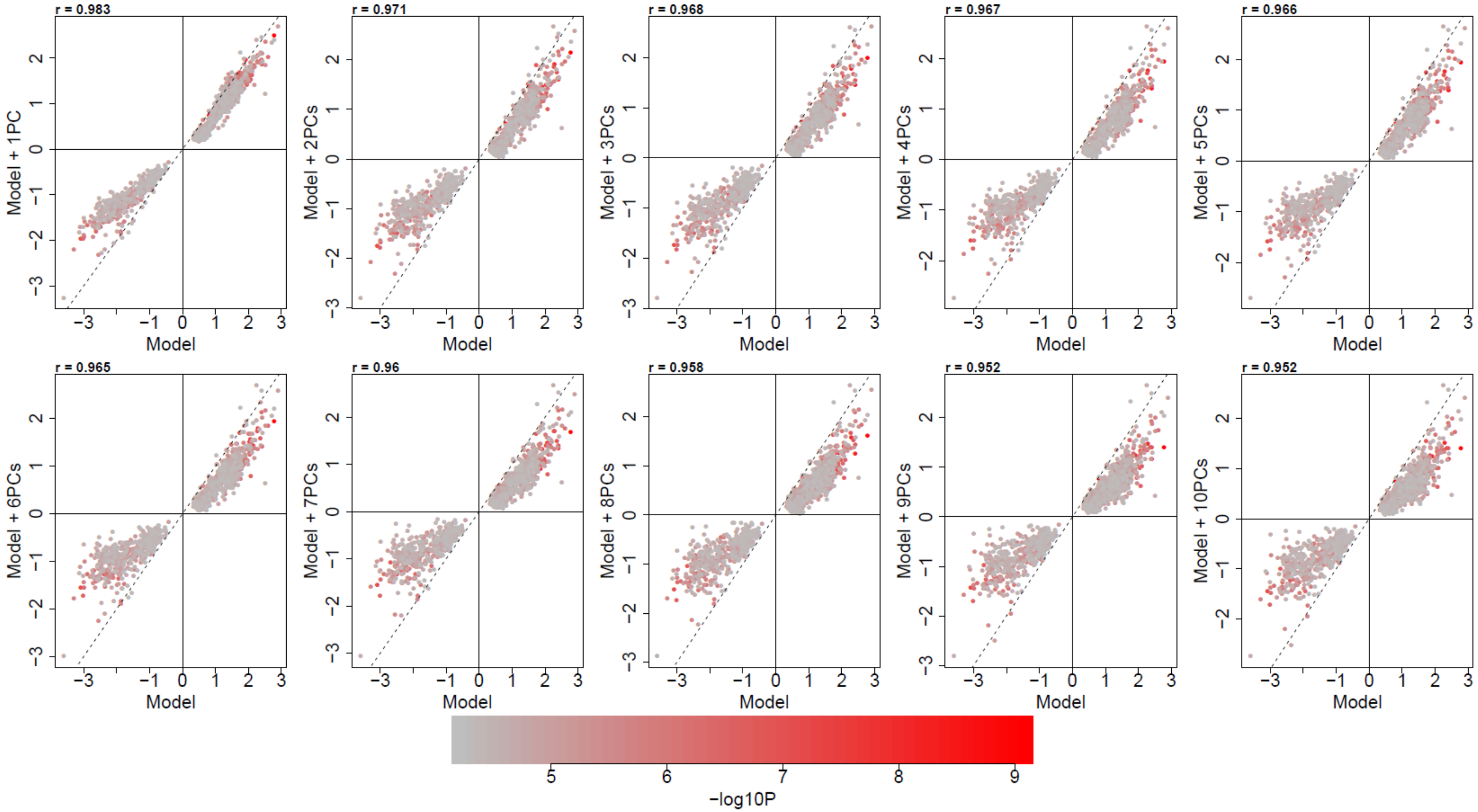

Figure S8: P values for differentially methylated regions are not biased by the number of probes located within them. Shown for 6 different sized sliding windows is the relationship between the number of DNA methylation sites within each tested region (x-axis) and –log10 P value (y-axis).

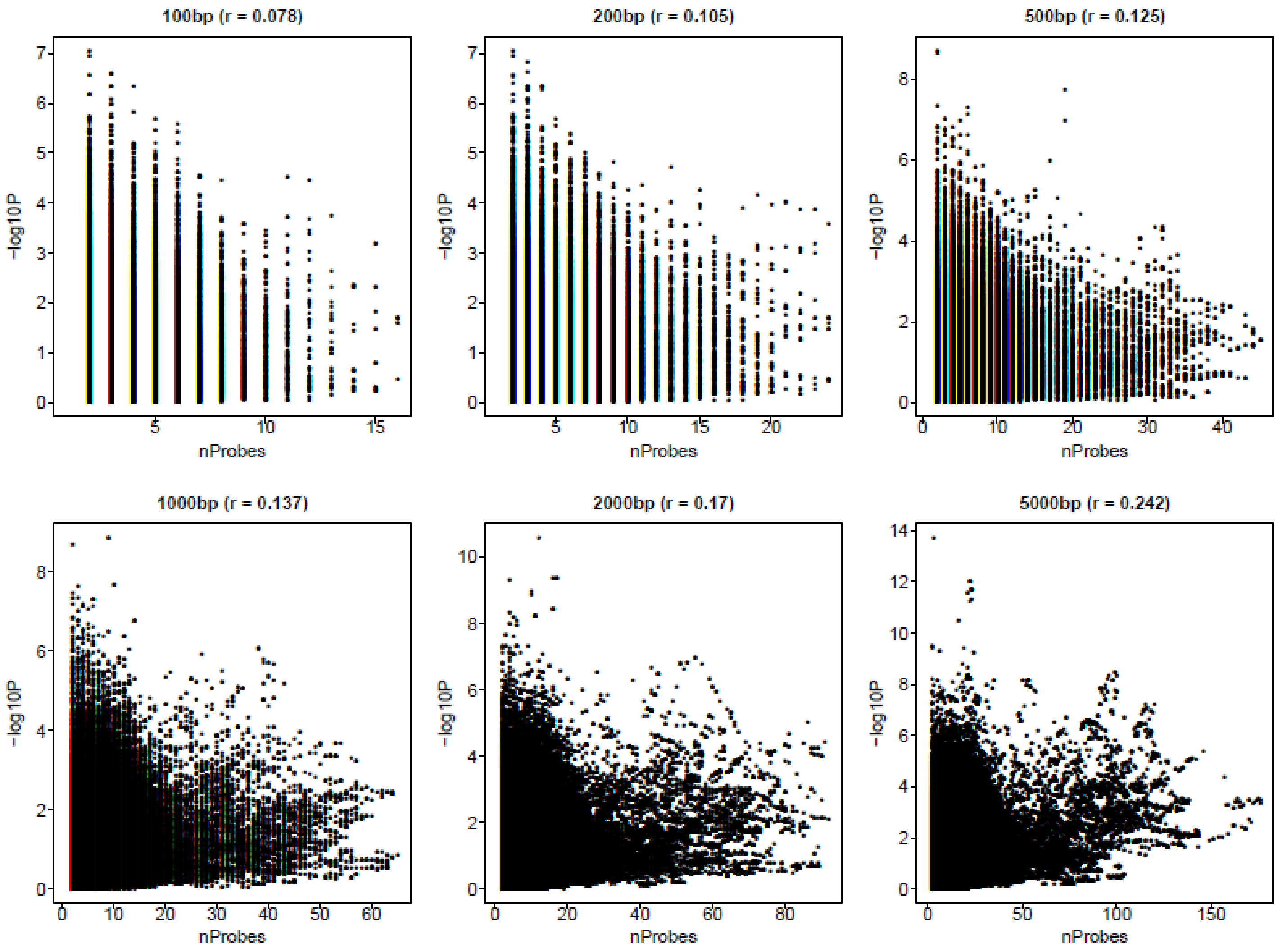

Figure S9. Schizophrenia-associated regions characterized by multiple independent signals. Scatterplot of best DMP –log10 P value (x-axis) against combined region –log10 P value (y-axis) for each differentially methylated region (DMR). Points above the solid black line represent regions where the combined region P value is more significant than any of the individual DMPs. The dashed black lines demonstrated array-wide significance for the probe level analysis (vertical) and regional analyses (horizontal).

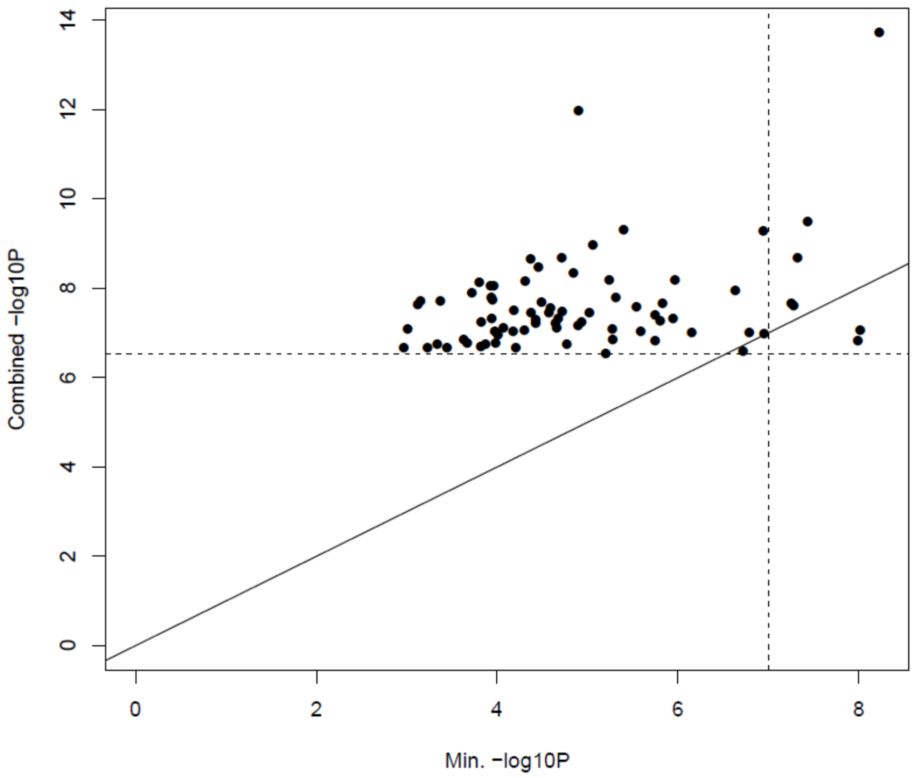

Figure S10 The top-ranked schizophrenia associated differentially methylated region resides within *GYG1* on chromosome 3. This figure depicts the a) gene track of this region, b) the EWAS –log10(p value) for each probe located with this region and c) the mean difference in DNA methylation (%) between schizophrenia cases and controls (blue dot) and standard error (solid black line) associated with this effect.

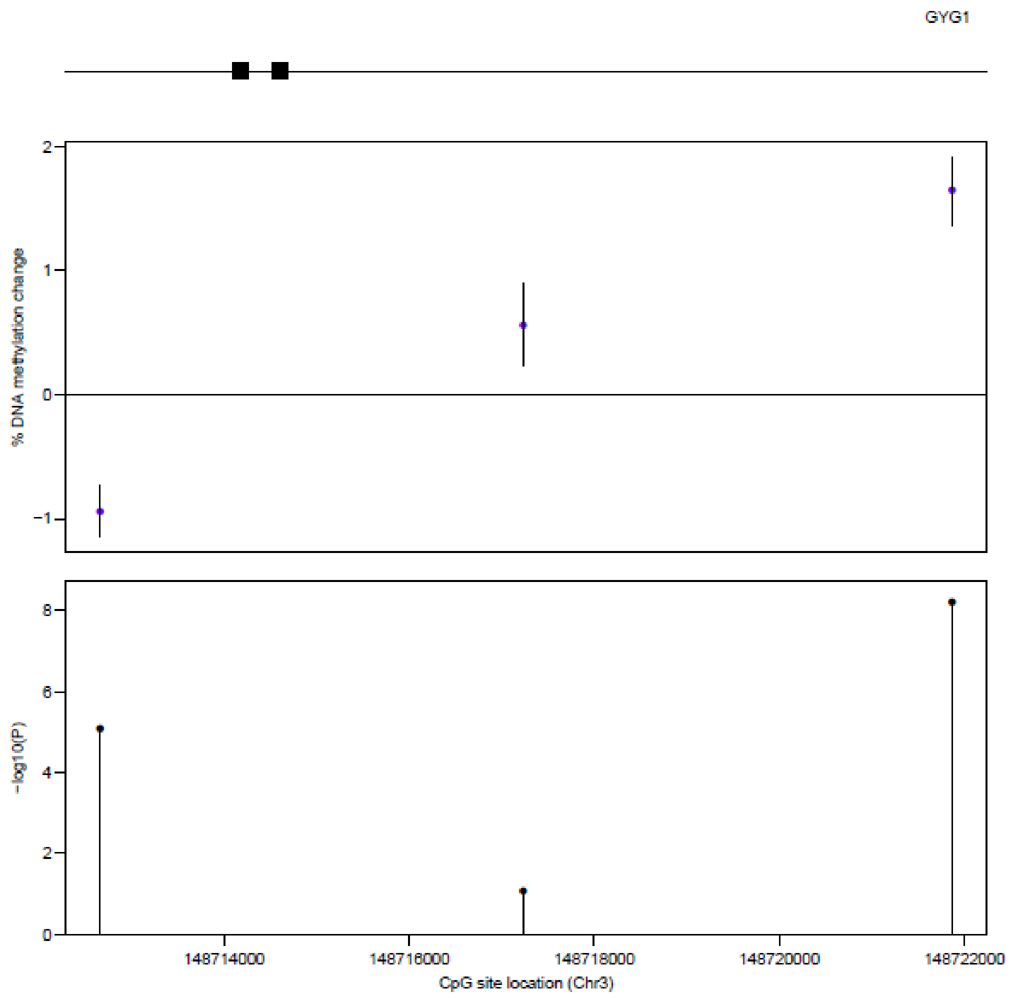

Supplementary Figure S11: Meta-analysis identifies DMPs with consistent changes associated with schizophrenia across three independent datasets. Heat-map comparing DNA methylation differences associated with schizophrenia across the three cohorts for all DMPs identified as significant ($P < 1 \times 10^{-7}$) in the meta-analysis. For each DMP (rows) in each cohort (columns) the color represents the DNA methylation difference associated with schizophrenia status.

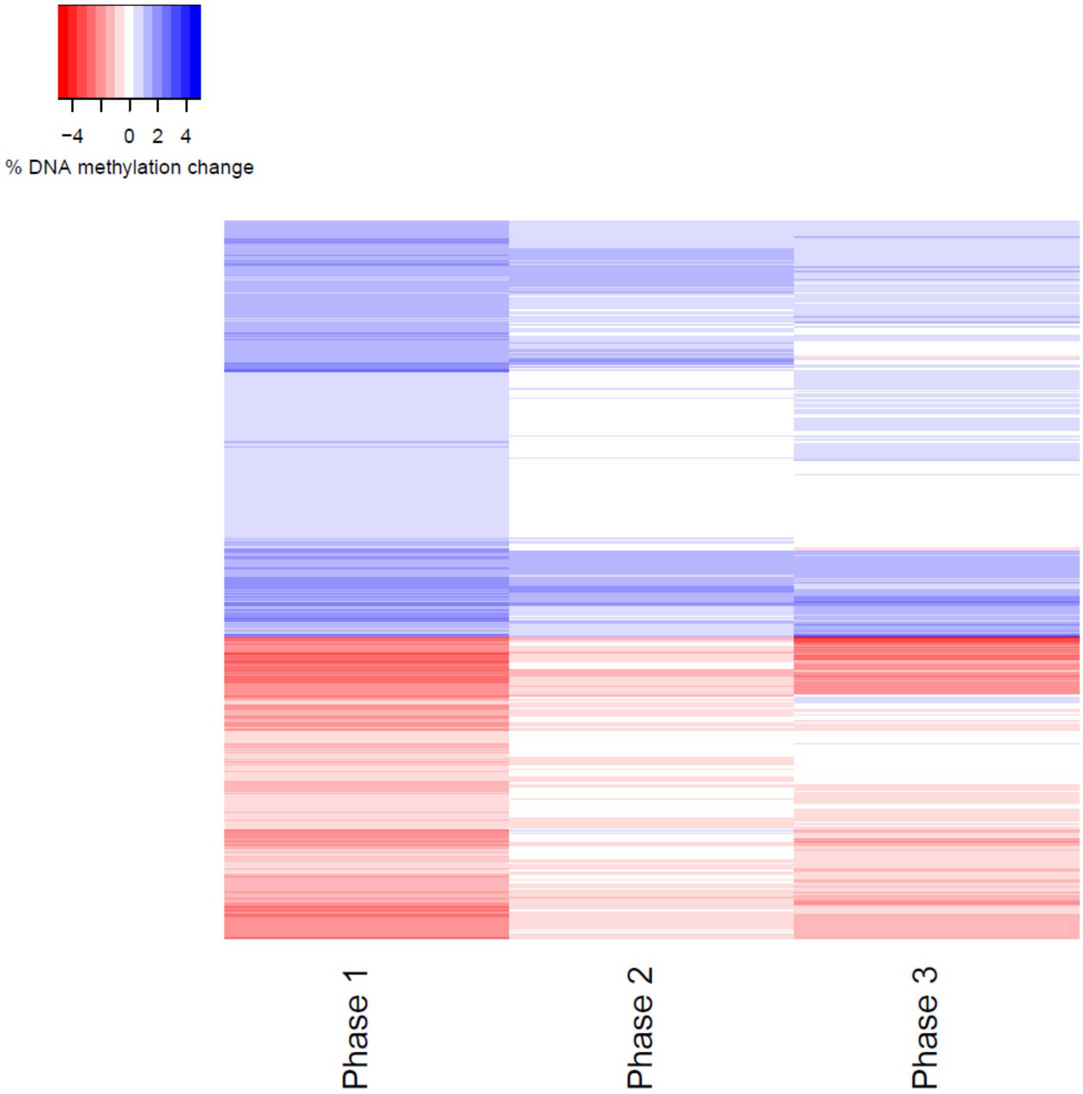

Supplementary Figure S12: The schizophrenia polygenic risk score (PRS) is significantly higher in cases compared to controls. Boxplot of distribution of PRS split into schizophrenia cases and controls for samples from a) phase 1 (332 cases, 307 controls; P = 3.34×10^{-27}) and b) phase 2 (451 cases, 477 controls; P = 2.09×10^{-31}).

a)

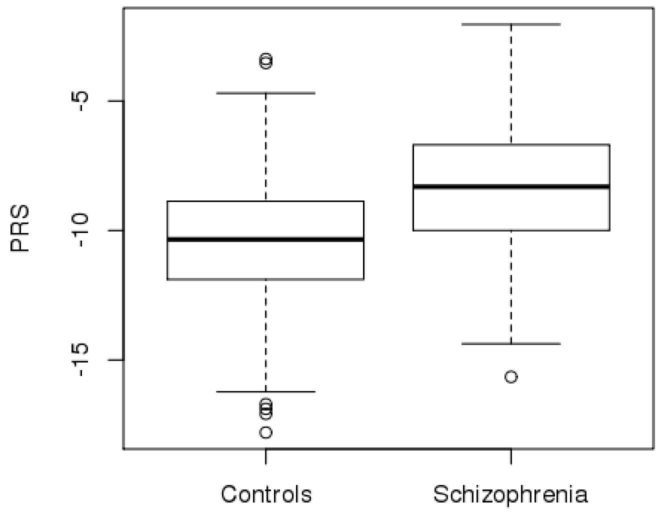

b)

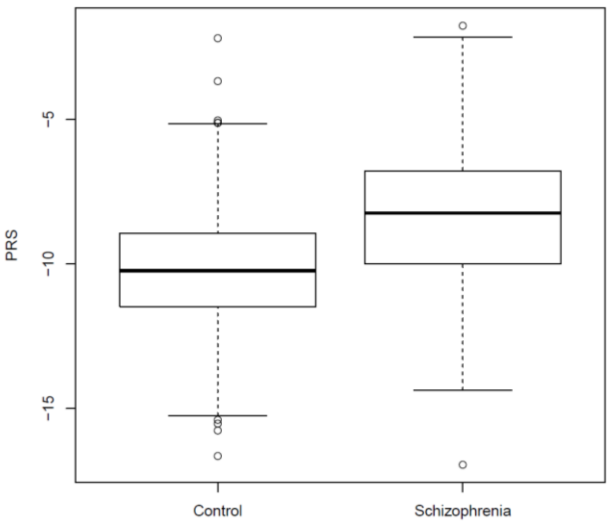

Supplementary Figure S13: Smoking status does not confound the EWAS of schizophrenia PRS. Manhattan plots comparing the a) PRS EWAS not controlling for smoking, b) smoking EWAS and c) EWAS controlling for smoking. Unlike the case-control EWAS (see **Figure 1**) there is minimal overlap with the results of the smoking EWAS.

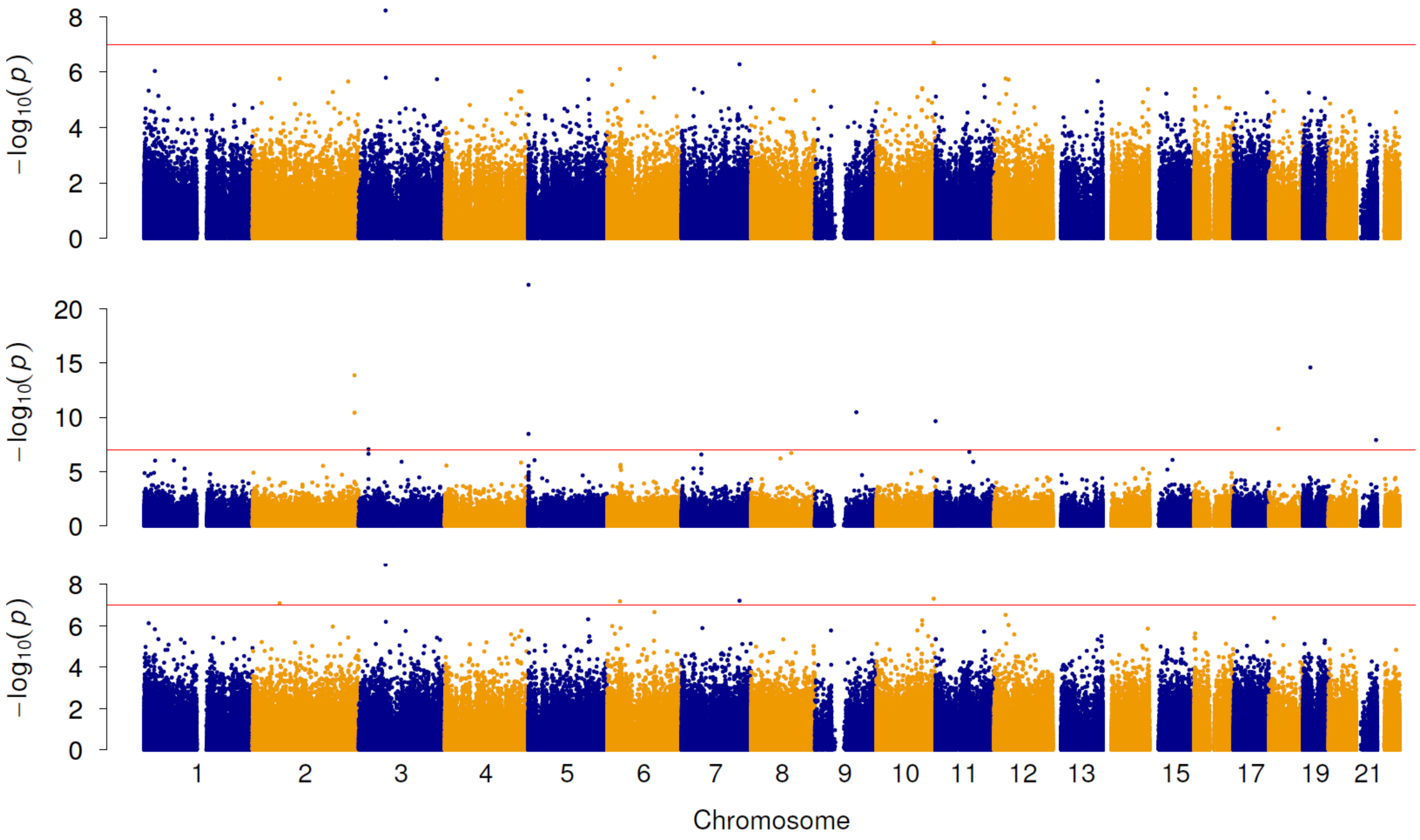

Figure S14 Addition of a smoking covariate does not influence schizophrenia PRS EWAS results. Scatterplots comparing EWAS of PRS with (y-axis) and without smoking covariate (x-axis). The plot on the left compares –log10(P value) and the plot on the right compares regression coefficients.

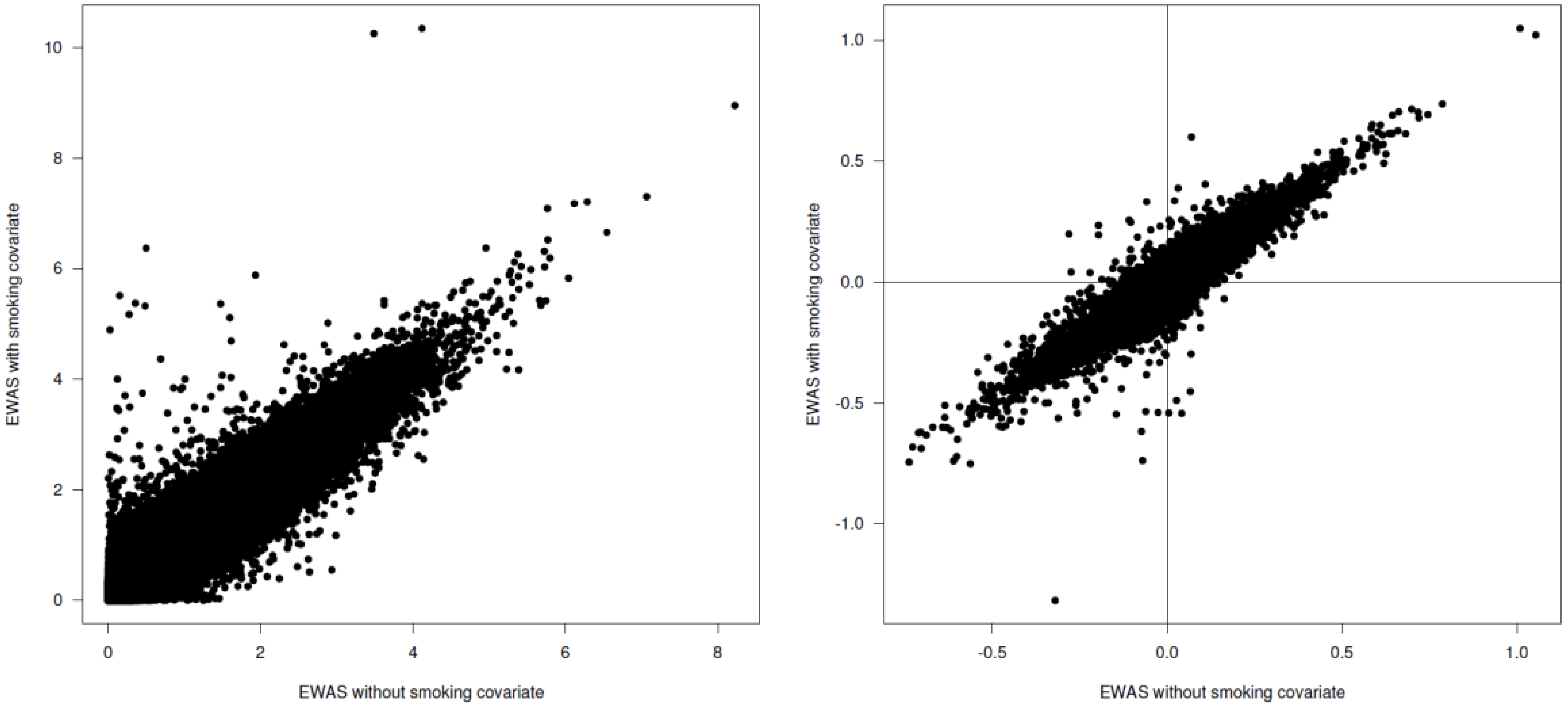

Supplementary Figure S15: PRS-associated DNA methylation differences are robust to the addition of PCs capturing variation in DNA methylation data. Shown for 156 PRS-associated DMPs ($P < 5 \times 10^{-5}$) are DNA methylation effect sizes in the EWAS unadjusted for PCs (x-axis) and the EWAS iteratively including additional PCs (y-axis). Points are coloured by their significance in the original EWAS.

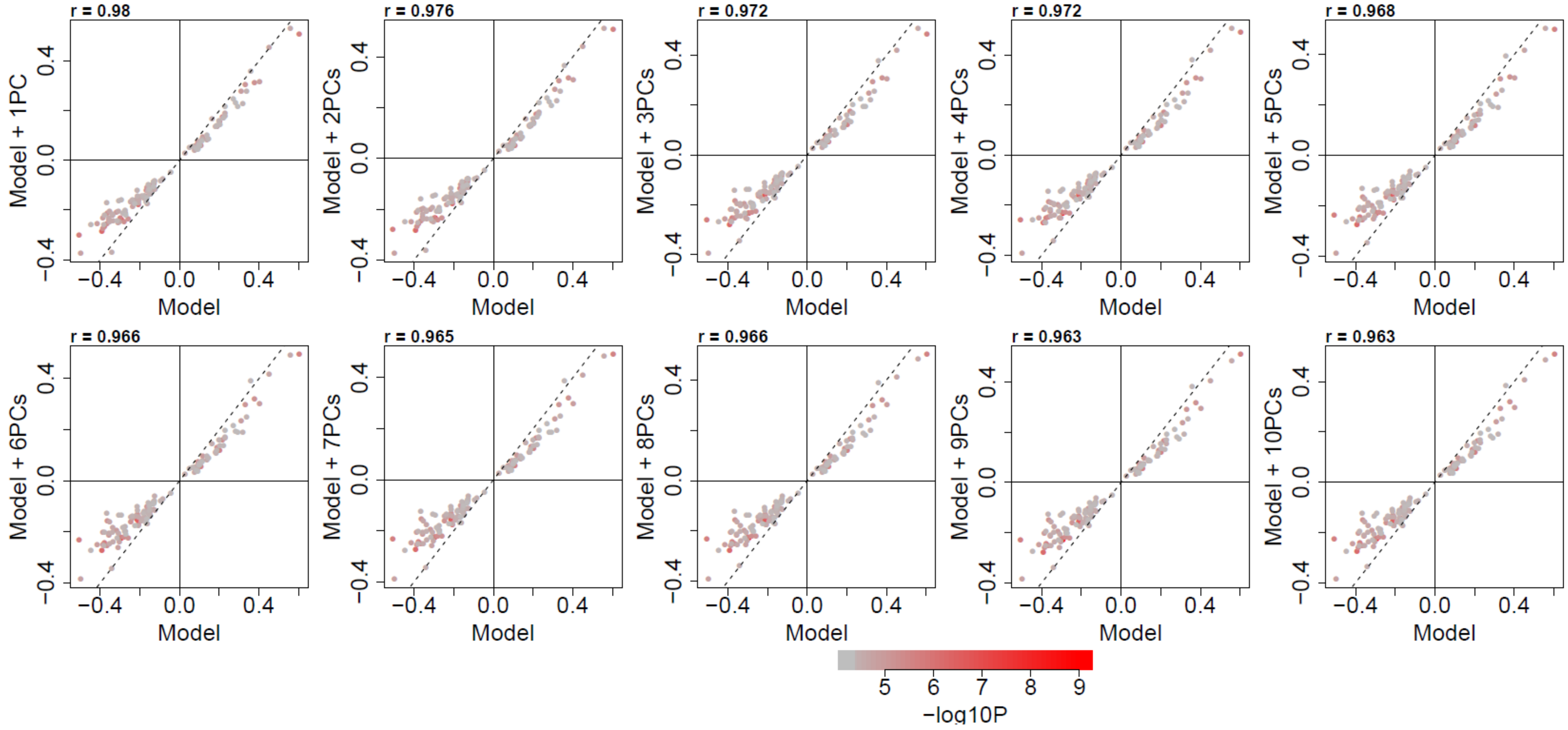

Supplementary Figure S16: PRS-associated DMPs show consistent associations with DNA methylation in the phase 2 replication cohort. Scatterplot demonstrating the concordance in effect sizes between the phase 1 (x-axis) and phase 2 for DMPs ($P < 5 \times 10^{-5}$) associated with schizophrenia PRS.

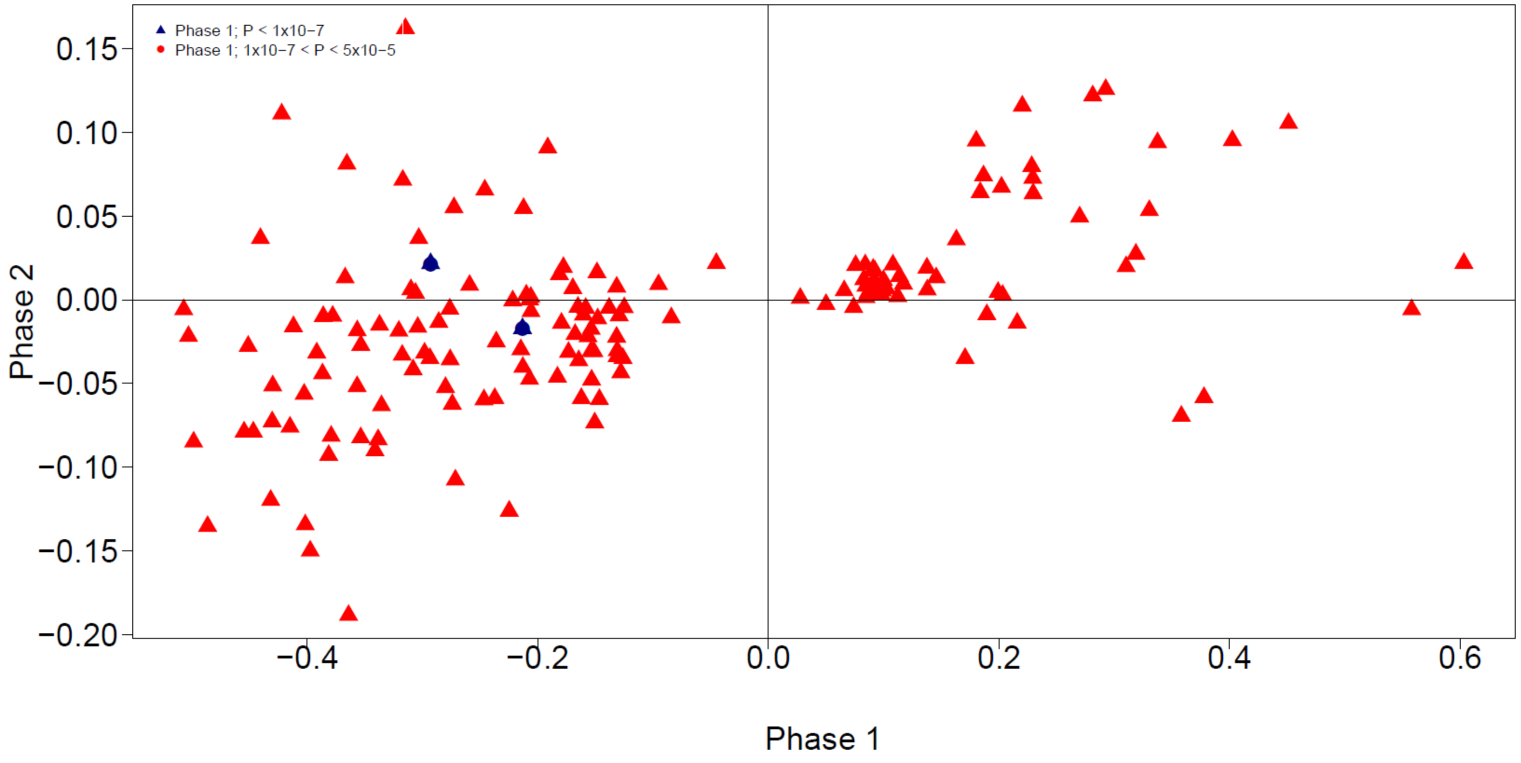

Supplementary Figure S17: Evidence for multiple independent signals across genomic regions associated with schizophrenia in GWAS. Scatterplot of best DMP –log10 P value (x-axis) against combined region –log10 P value (y-axis) for each GWAS-nominated genomic region in a) the case control EWAS and b) the PRS EWAS. Points above the solid black line represent regions where the combined region P value is more significant than any of the individual DMPs. The dashed black lines demonstrated array-wide significance for the probe level analysis (vertical) and GWAS region analyses (horizontal) (P < 0. 000658).

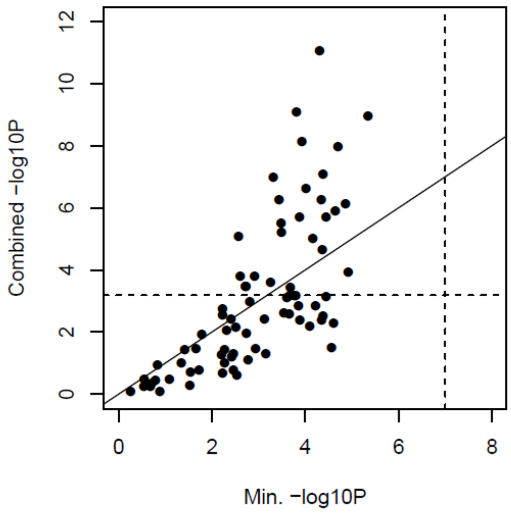

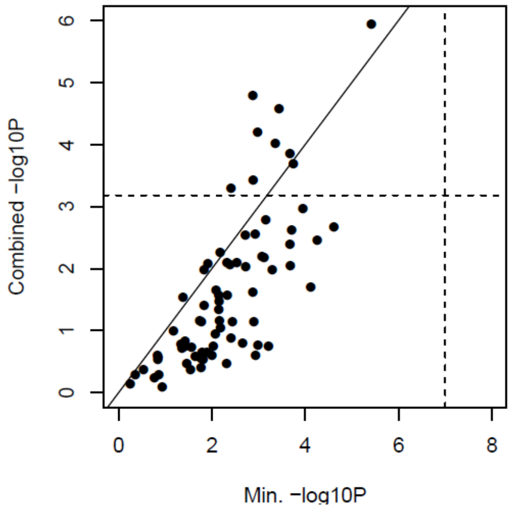

References

1. Elliott HR, Tillin T, McArdle WL, Ho K, Duggirala A, Frayling TM, Davey Smith G, Hughes AD, Chaturvedi N, Relton CL: **Differences in smoking associated DNA methylation patterns in South Asians and Europeans.** *Clin Epigenetics* 2014, **6:**4.
2. Zeilinger S, Kühnel B, Klopp N, Baurecht H, Kleinschmidt A, Gieger C, Weidinger S, Lattka E, Adamski J, Peters A, et al: **Tobacco smoking leads to extensive genome-wide changes in DNA methylation.** *PLoS One* 2013, **8:**e63812.
3. Horvath S: **DNA methylation age of human tissues and cell types.** *Genome Biol* 2013, **14:**R115.
4. Houseman EA, Accomando WP, Koestler DC, Christensen BC, Marsit CJ, Nelson HH, Wiencke JK, Kelsey KT: **DNA methylation arrays as surrogate measures of cell mixture distribution.** *BMC Bioinformatics* 2012, **13:**86.
5. Koestler DC, Christensen B, Karagas MR, Marsit CJ, Langevin SM, Kelsey KT, Wiencke JK, Houseman EA: **Blood-based profiles of DNA methylation predict the underlying distribution of cell types: a validation analysis.** *Epigenetics* 2013, **8:**816-826.
6. Hannon E, Lunnon K, Schalkwyk L, Mill J: **Interindividual methylomic variation across blood, cortex, and cerebellum: implications for epigenetic studies of neurological and neuropsychiatric phenotypes.** *Epigenetics* 2015, **10:**1024-1032.
