# Supplementary material for: An integrated genetic-epigenetic analysis of schizophrenia: evidence for co-localization of genetic associations and differential DNA methylation
Source: Genome Biol. 2016 Aug 30;17(1):176. doi: 10.1186/s13059-016-1041-x (PMC5004279; doi:10.1186/s13059-016-1041-x)
Supplement: Additional file 3: — Forest plots from case–control meta-analyses. (PDF 462 kb) [file 13059_2016_1041_MOESM3_ESM.pdf]

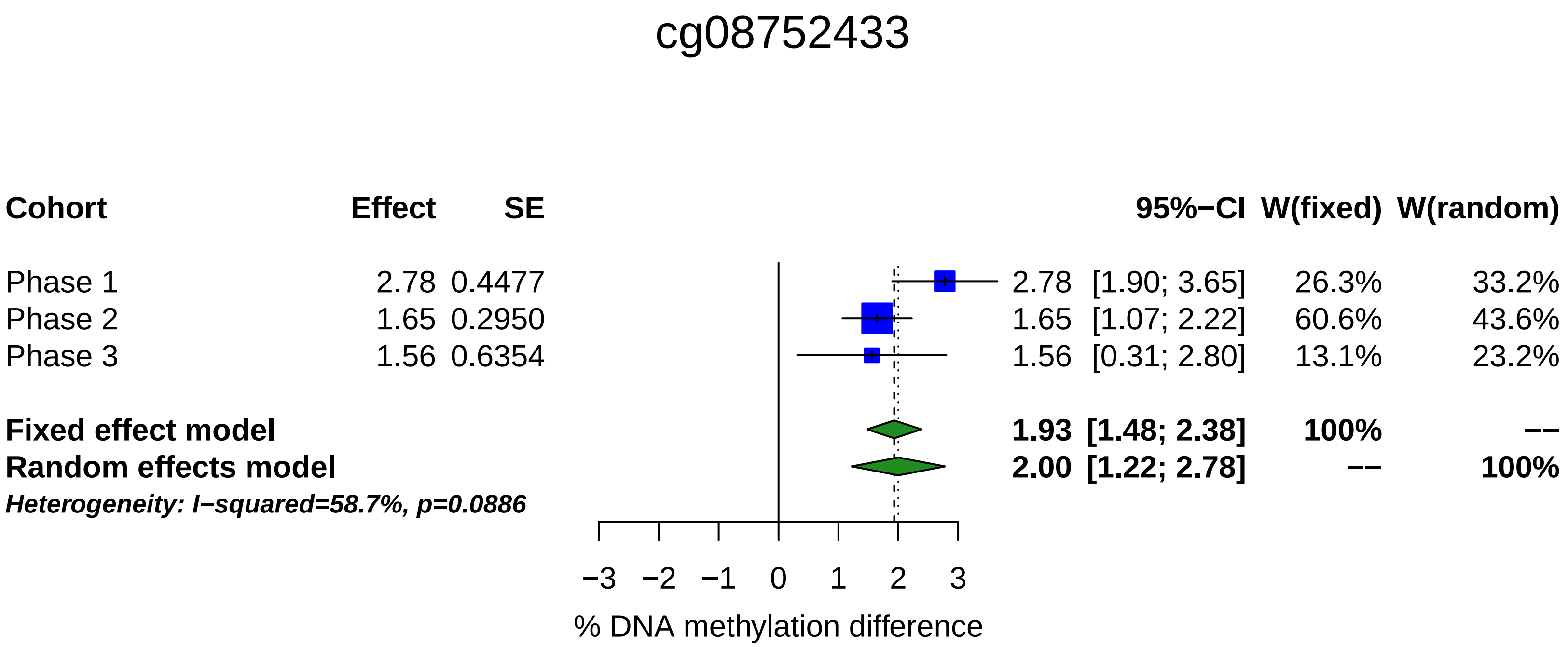
cg08752433
Cohort
Effect
SE
95%−CI
W(fixed)
W(random)
Phase 1 2.78 0.4477 2.78 [1.90; 3.65] 26.3% 33.2%
Phase 2 1.65 0.2950 1.65 [1.07; 2.22] 60.6% 43.6%
Phase 3 1.56 0.6354 1.56 [0.31; 2.80] 13.1% 23.2%
Fixed effect model 1.93 [1.48; 2.38] 100% −−
Random effects model 2.00 [1.22; 2.78] −− 100%
Heterogeneity: I−squared=58.7%, p=0.0886
−3 −2 −1 0 1 2 3
% DNA methylation difference

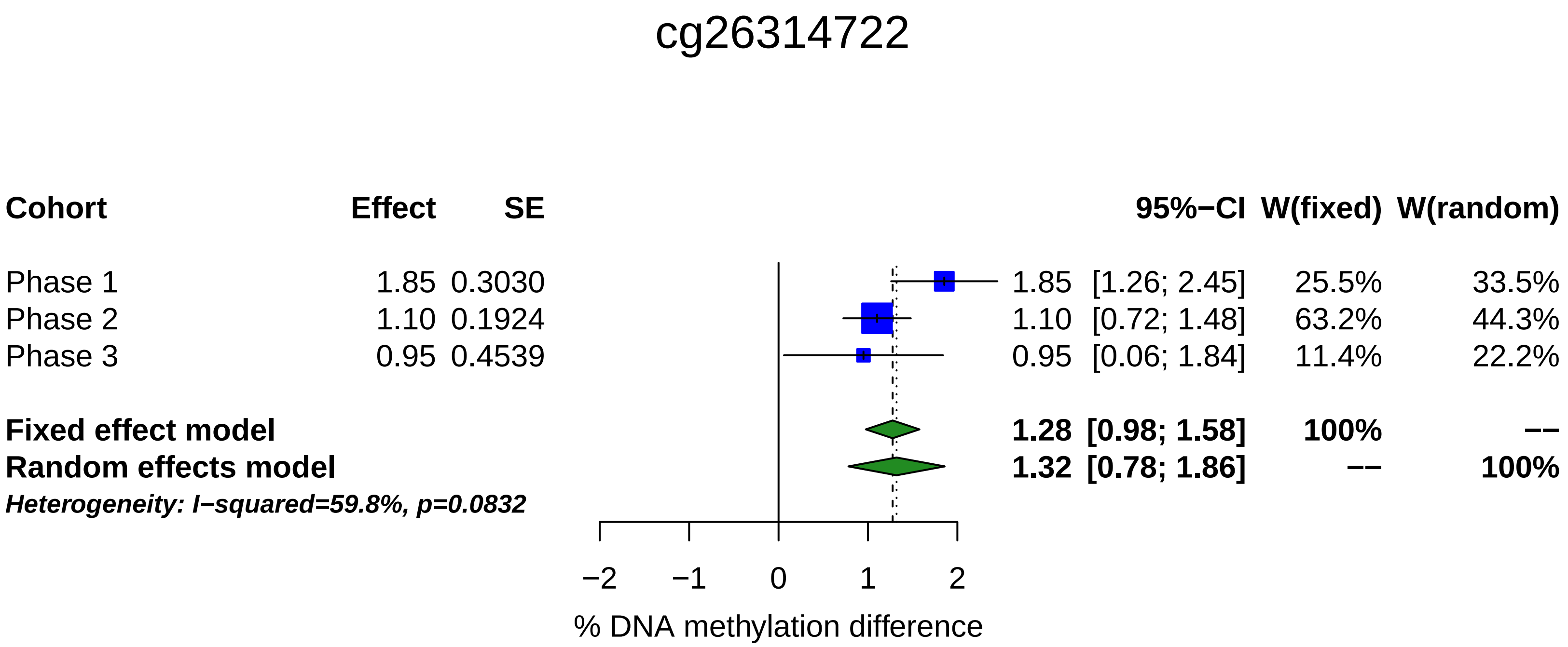

cg26314722
Cohort
Effect
SE
95%−CI
W(fixed)
W(random)
Phase 1 1.85 0.3030 1.85 [1.26; 2.45] 25.5% 33.5%
Phase 2 1.10 0.1924 1.10 [0.72; 1.48] 63.2% 44.3%
Phase 3 0.95 0.4539 0.95 [0.06; 1.84] 11.4% 22.2%
Fixed effect model 1.28 [0.98; 1.58] 100% −−
Random effects model 1.32 [0.78; 1.86] −− 100%
Heterogeneity: I−squared=59.8%, p=0.0832
−2
−1
0
1
2
% DNA methylation difference

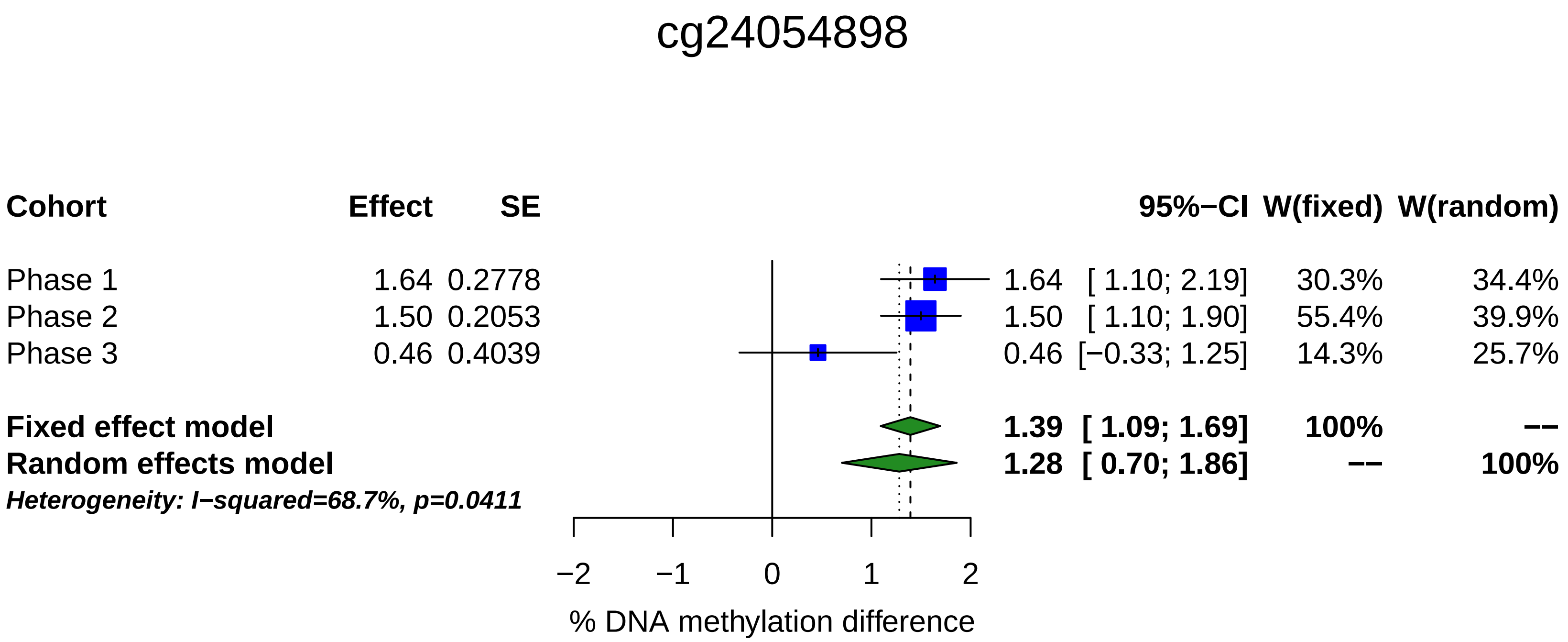
cg24054898
Cohort
Effect
SE
95%−CI
W(fixed)
W(random)
Phase 1 1.64 0.2778 1.64 [1.10; 2.19] 30.3% 34.4%
Phase 2 1.50 0.2053 1.50 [1.10; 1.90] 55.4% 39.9%
Phase 3 0.46 0.4039 0.46 [−0.33; 1.25] 14.3% 25.7%
Fixed effect model 1.39 [1.09; 1.69] 100% −−
Random effects model 1.28 [0.70; 1.86] −− 100%
Heterogeneity: I−squared=68.7%, p=0.0411
−2
−1
0
1
2
% DNA methylation difference

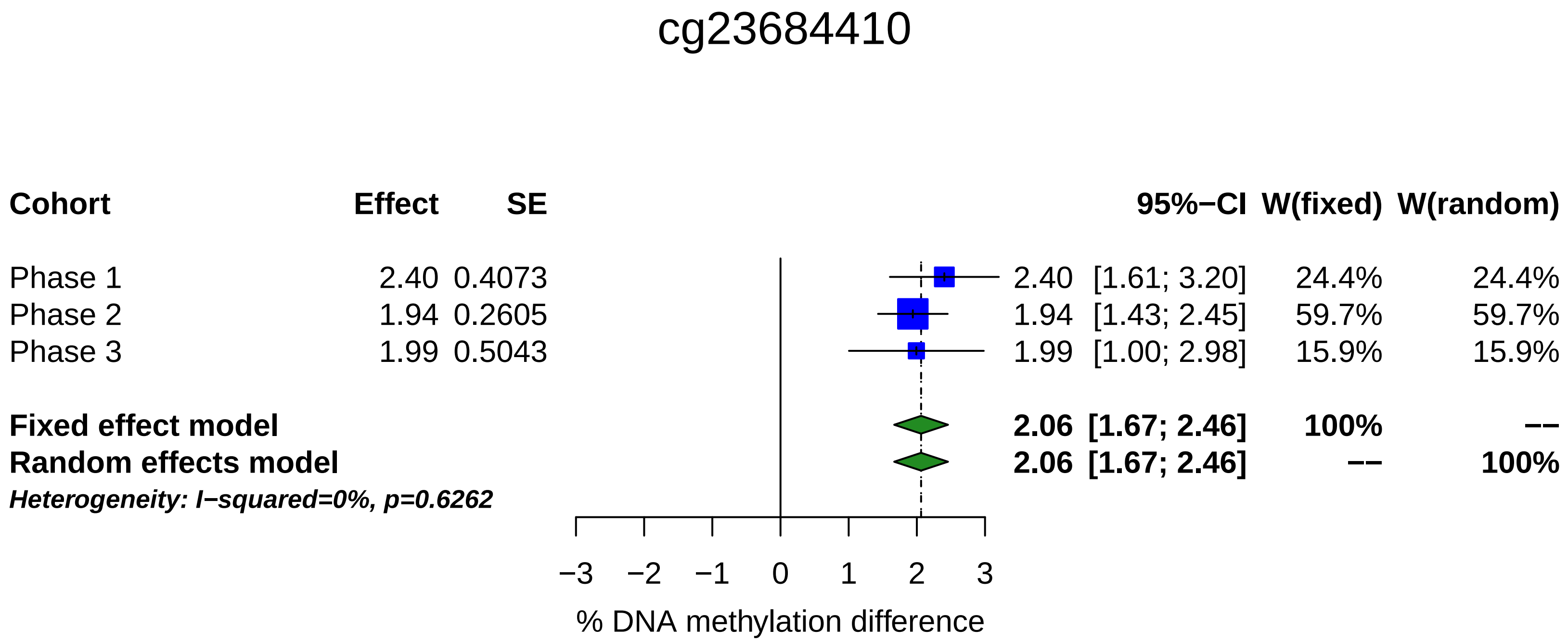

cg23684410
Cohort
Effect
SE
95%−CI
W(fixed)
W(random)
Phase 1 2.40 0.4073 2.40 [1.61; 3.20] 24.4% 24.4%
Phase 2 1.94 0.2605 1.94 [1.43; 2.45] 59.7% 59.7%
Phase 3 1.99 0.5043 1.99 [1.00; 2.98] 15.9% 15.9%
Fixed effect model 2.06 [1.67; 2.46] 100% −−
Random effects model 2.06 [1.67; 2.46] −− 100%
Heterogeneity: I−squared=0%, p=0.6262
−3 −2 −1 0 1 2 3
% DNA methylation difference

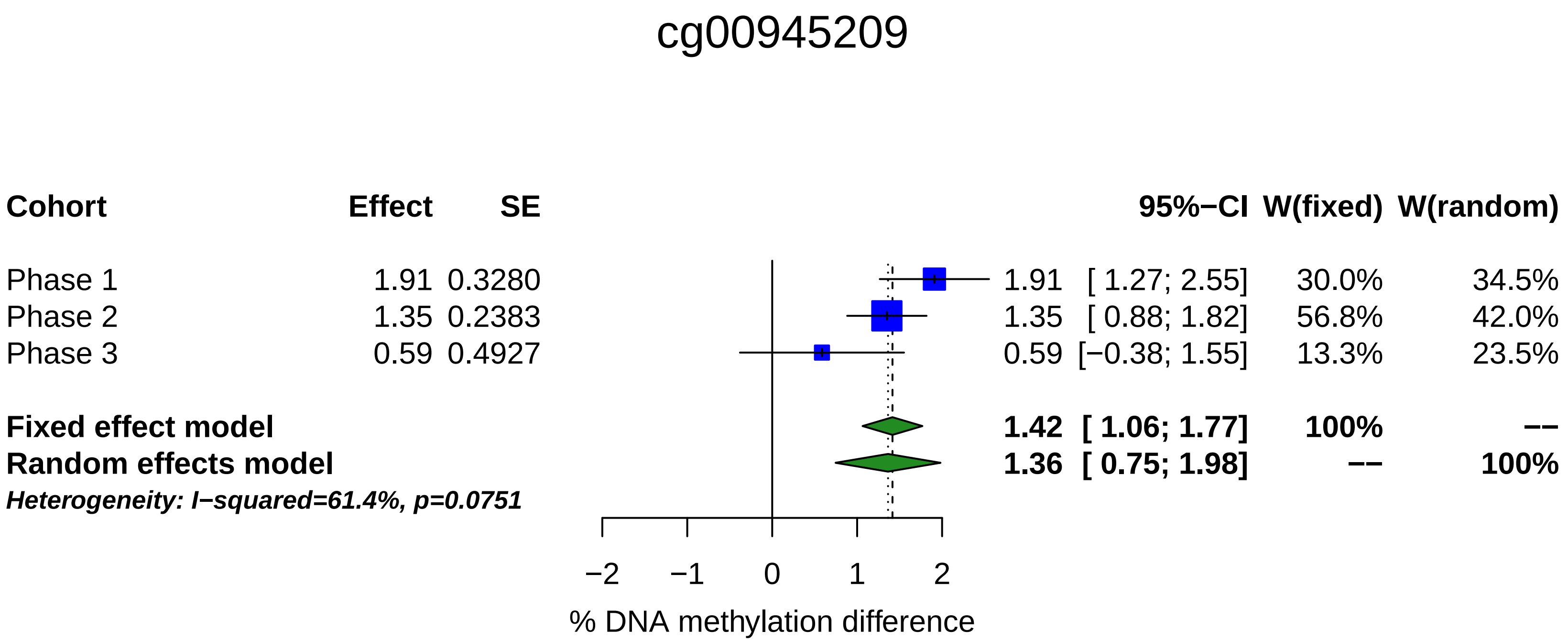
cg00945209
Cohort
Effect
SE
95%−CI
W(fixed)
W(random)
Phase 1
1.91
0.3280
1.91 [1.27; 2.55]
30.0%
34.5%
Phase 2
1.35
0.2383
1.35 [0.88; 1.82]
56.8%
42.0%
Phase 3
0.59
0.4927
0.59 [−0.38; 1.55]
13.3%
23.5%
Fixed effect model
1.42 [1.06; 1.77]
100%
−−
Random effects model
1.36 [0.75; 1.98]
−−
100%
Heterogeneity: I−squared=61.4%, p=0.0751
−2
−1
0
1
2
% DNA methylation difference

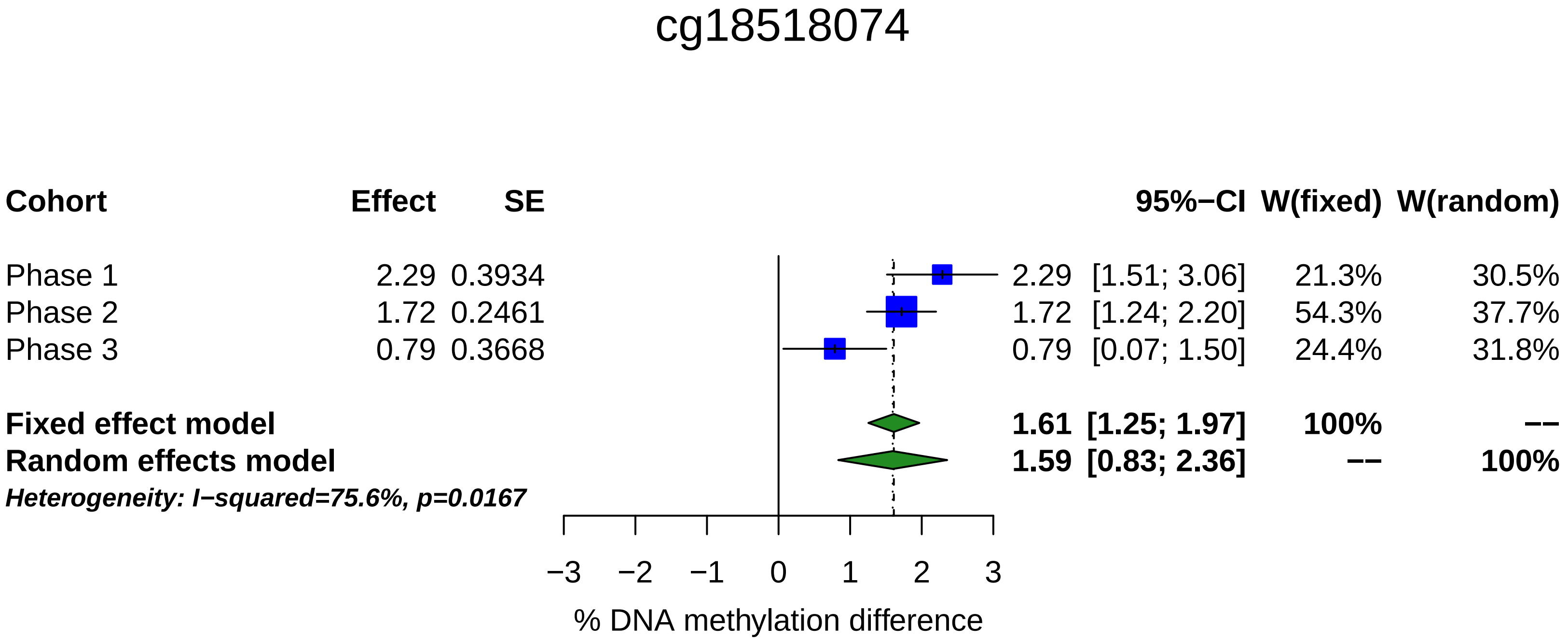
cg18518074
Cohort
Effect
SE
95%−CI
W(fixed)
W(random)
Phase 1 2.29 0.3934 2.29 [1.51; 3.06] 21.3% 30.5%
Phase 2 1.72 0.2461 1.72 [1.24; 2.20] 54.3% 37.7%
Phase 3 0.79 0.3668 0.79 [0.07; 1.50] 24.4% 31.8%
Fixed effect model 1.61 [1.25; 1.97] 100% −−
Random effects model 1.59 [0.83; 2.36] −− 100%
Heterogeneity: I−squared=75.6%, p=0.0167
−3
−2
−1
0
1
2
3
% DNA methylation difference

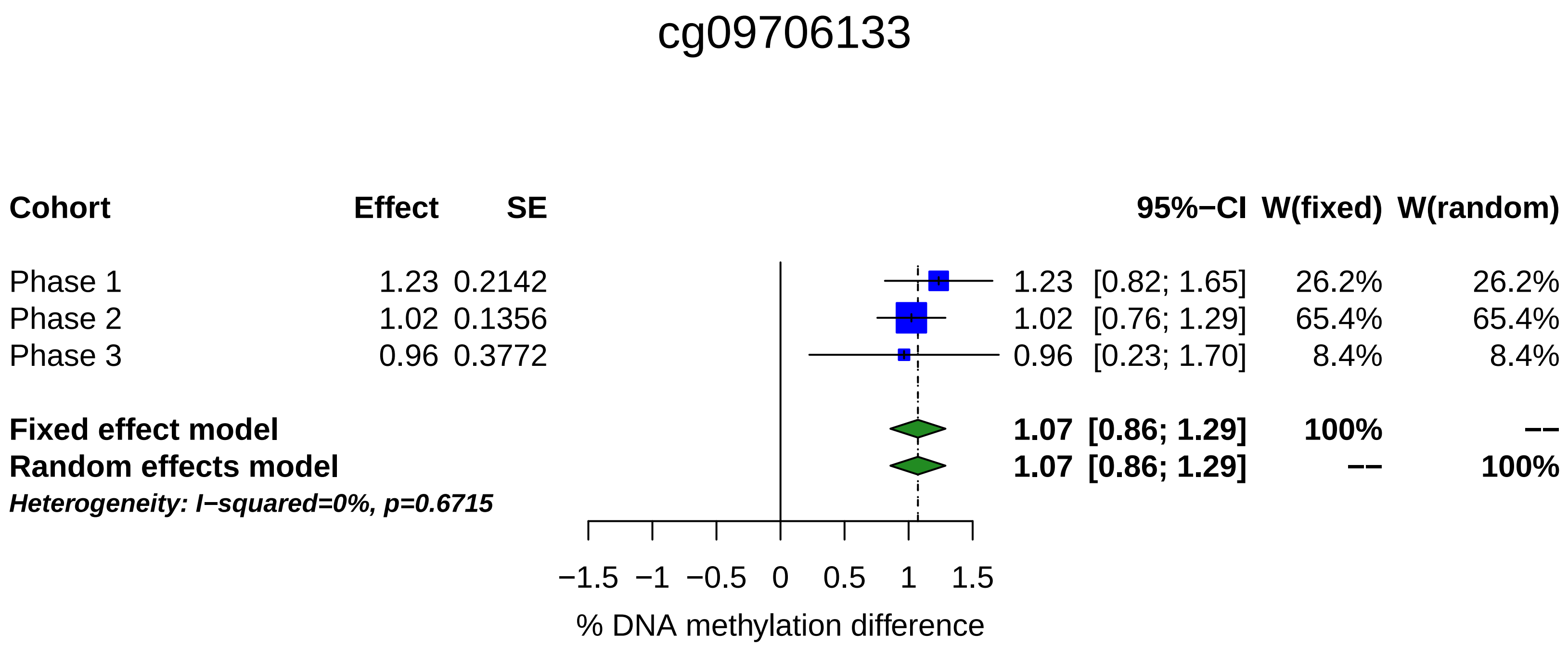
cg09706133
Cohort
Effect
SE
95%−CI
W(fixed)
W(random)
Phase 1 1.23 0.2142 1.23 [0.82; 1.65] 26.2% 26.2%
Phase 2 1.02 0.1356 1.02 [0.76; 1.29] 65.4% 65.4%
Phase 3 0.96 0.3772 0.96 [0.23; 1.70] 8.4% 8.4%
Fixed effect model 1.07 [0.86; 1.29] 100% −−
Random effects model 1.07 [0.86; 1.29] −− 100%
Heterogeneity: I−squared=0%, p=0.6715
−1.5 −1 −0.5 0 0.5 1 1.5
% DNA methylation difference

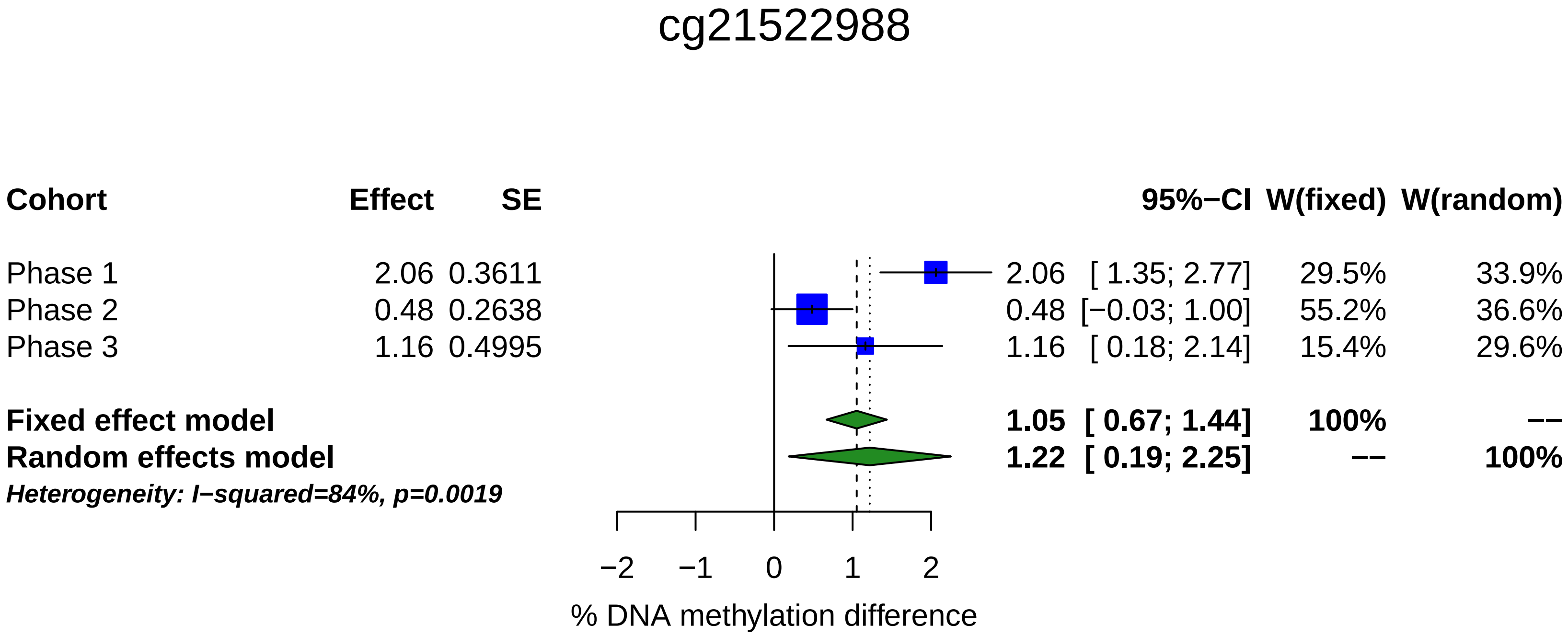
cg21522988
Cohort
Effect
SE
95%−CI
W(fixed)
W(random)
Phase 1 2.06 0.3611 2.06 [1.35; 2.77] 29.5% 33.9%
Phase 2 0.48 0.2638 0.48 [−0.03; 1.00] 55.2% 36.6%
Phase 3 1.16 0.4995 1.16 [0.18; 2.14] 15.4% 29.6%
Fixed effect model 1.05 [0.67; 1.44] 100% −−
Random effects model 1.22 [0.19; 2.25] −− 100%
Heterogeneity: I−squared=84%, p=0.0019
−2
−1
0
1
2
% DNA methylation difference

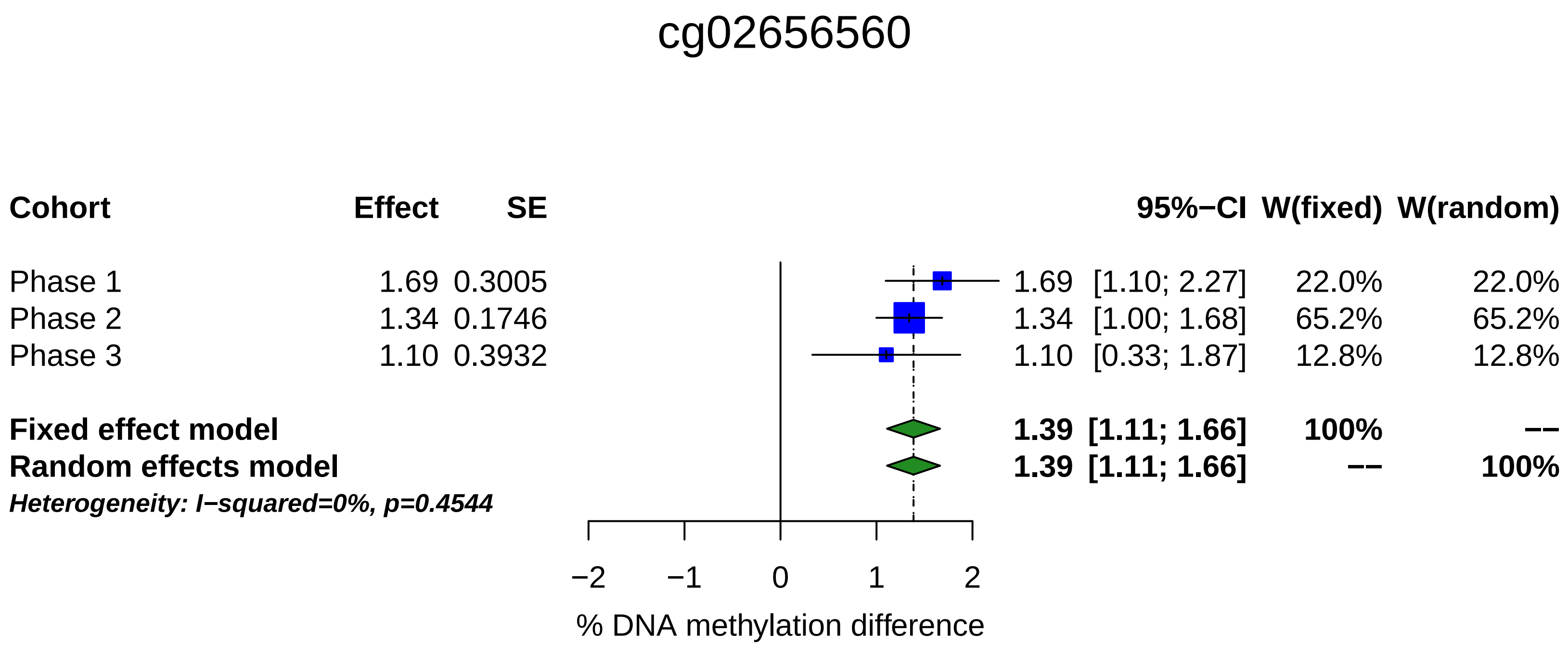
cg02656560
Cohort
Effect
SE
95%−CI
W(fixed)
W(random)
Phase 1 1.69 0.3005 1.69 [1.10; 2.27] 22.0% 22.0%
Phase 2 1.34 0.1746 1.34 [1.00; 1.68] 65.2% 65.2%
Phase 3 1.10 0.3932 1.10 [0.33; 1.87] 12.8% 12.8%
Fixed effect model 1.39 [1.11; 1.66] 100% −−
Random effects model 1.39 [1.11; 1.66] −− 100%
Heterogeneity: I−squared=0%, p=0.4544
−2
−1
0
1
2
% DNA methylation difference

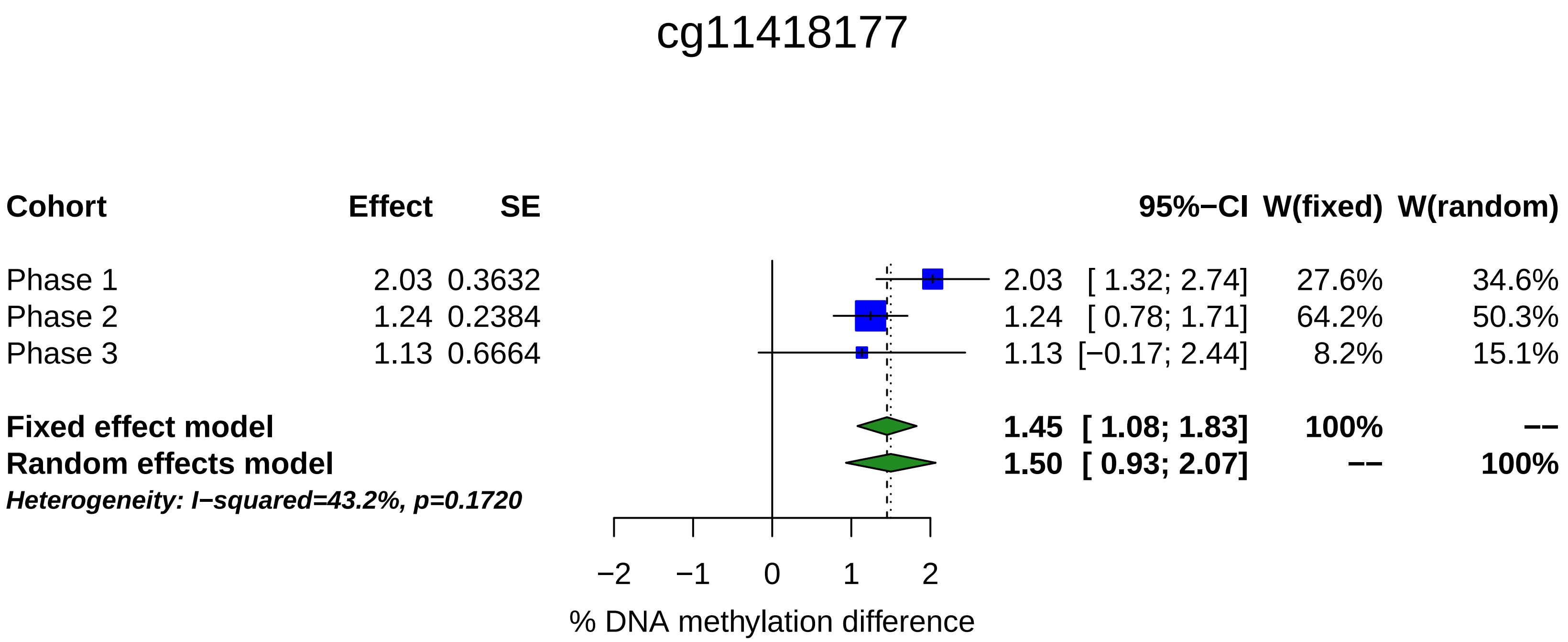
cg11418177
Cohort
Effect
SE
95%−CI
W(fixed)
W(random)
Phase 1
2.03
0.3632
2.03 [1.32; 2.74]
27.6%
34.6%
Phase 2
1.24
0.2384
1.24 [0.78; 1.71]
64.2%
50.3%
Phase 3
1.13
0.6664
1.13 [−0.17; 2.44]
8.2%
15.1%
Fixed effect model
1.45 [1.08; 1.83]
100%
−−
Random effects model
1.50 [0.93; 2.07]
−−
100%
Heterogeneity: I−squared=43.2%, p=0.1720
−2
−1
0
1
2
% DNA methylation difference

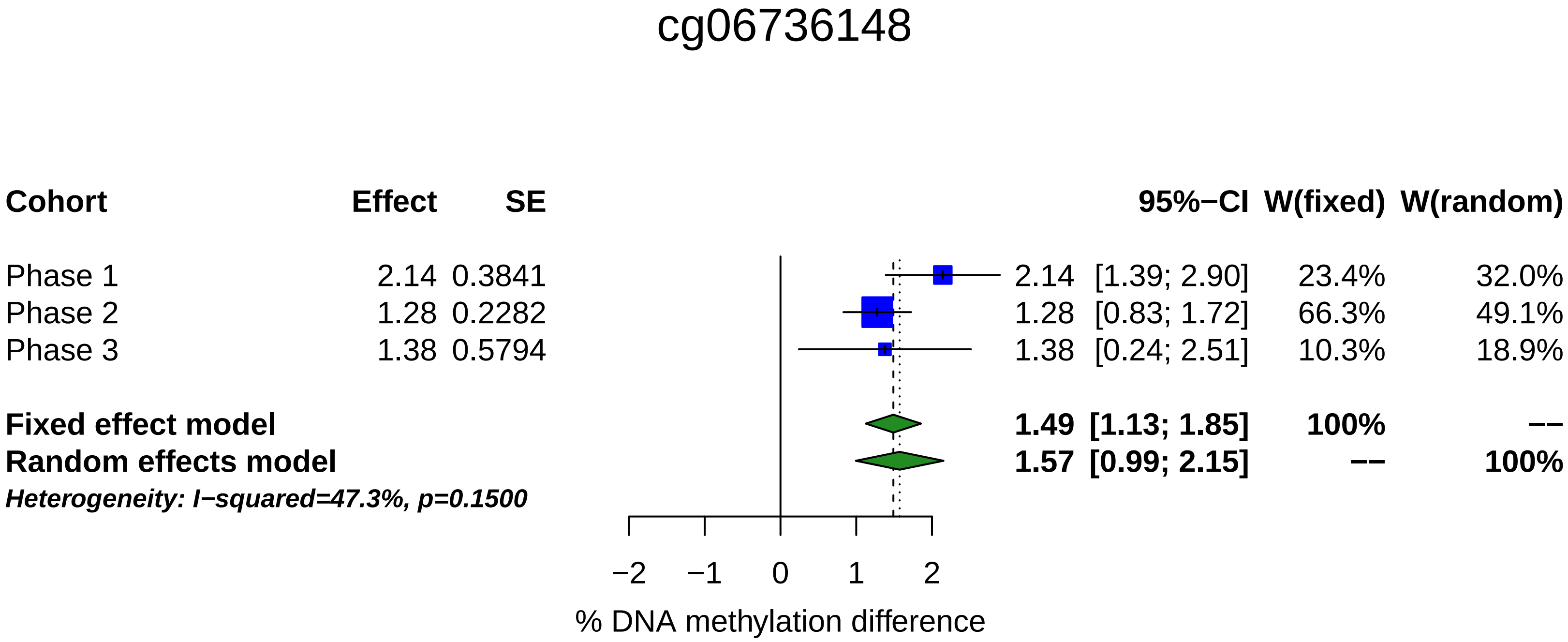
cg06736148
Cohort
Effect
SE
95%−CI
W(fixed)
W(random)
Phase 1 2.14 0.3841 2.14 [1.39; 2.90] 23.4% 32.0%
Phase 2 1.28 0.2282 1.28 [0.83; 1.72] 66.3% 49.1%
Phase 3 1.38 0.5794 1.38 [0.24; 2.51] 10.3% 18.9%
Fixed effect model 1.49 [1.13; 1.85] 100% −−
Random effects model 1.57 [0.99; 2.15] −− 100%
Heterogeneity: I−squared=47.3%, p=0.1500
−2
−1
0
1
2
% DNA methylation difference

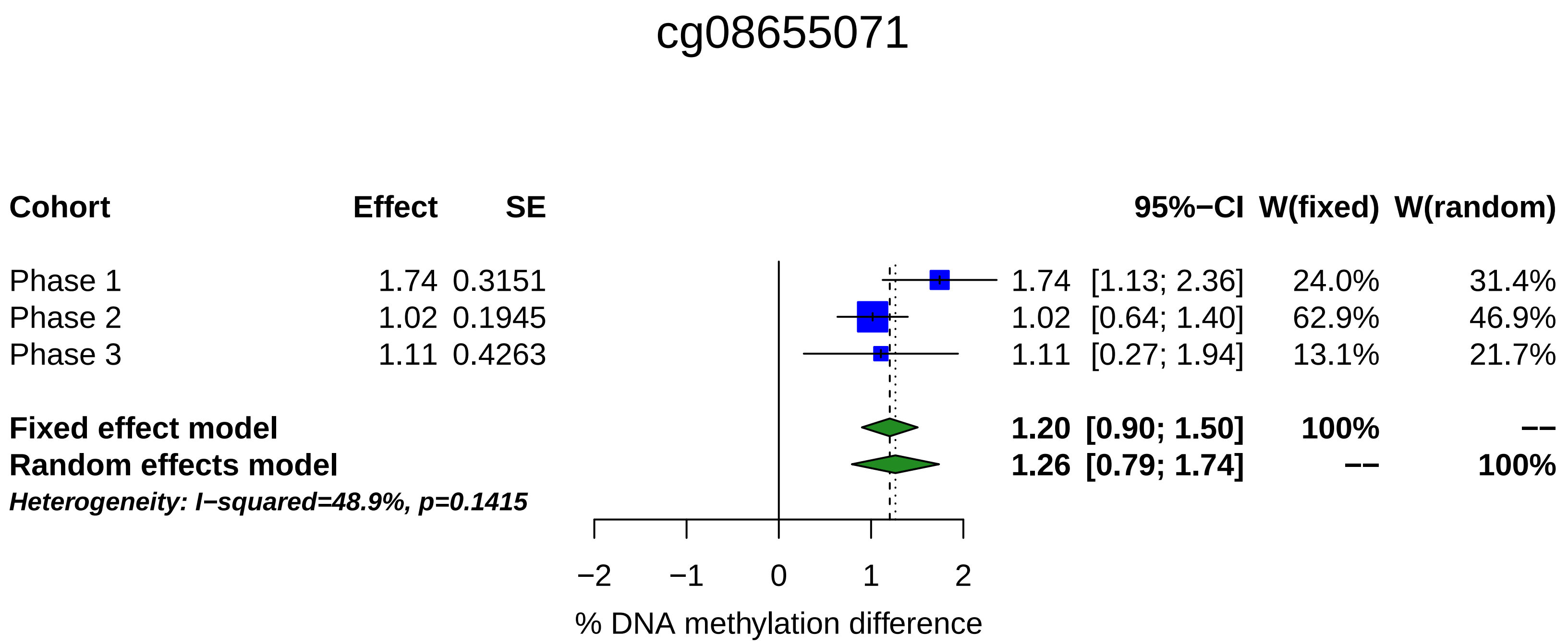

cg08655071
Cohort
Effect
SE
95%−CI
W(fixed)
W(random)
Phase 1 1.74 0.3151 1.74 [1.13; 2.36] 24.0% 31.4%
Phase 2 1.02 0.1945 1.02 [0.64; 1.40] 62.9% 46.9%
Phase 3 1.11 0.4263 1.11 [0.27; 1.94] 13.1% 21.7%
Fixed effect model 1.20 [0.90; 1.50] 100% −−
Random effects model 1.26 [0.79; 1.74] −− 100%
Heterogeneity: I−squared=48.9%, p=0.1415
−2
−1
0
1
2
% DNA methylation difference

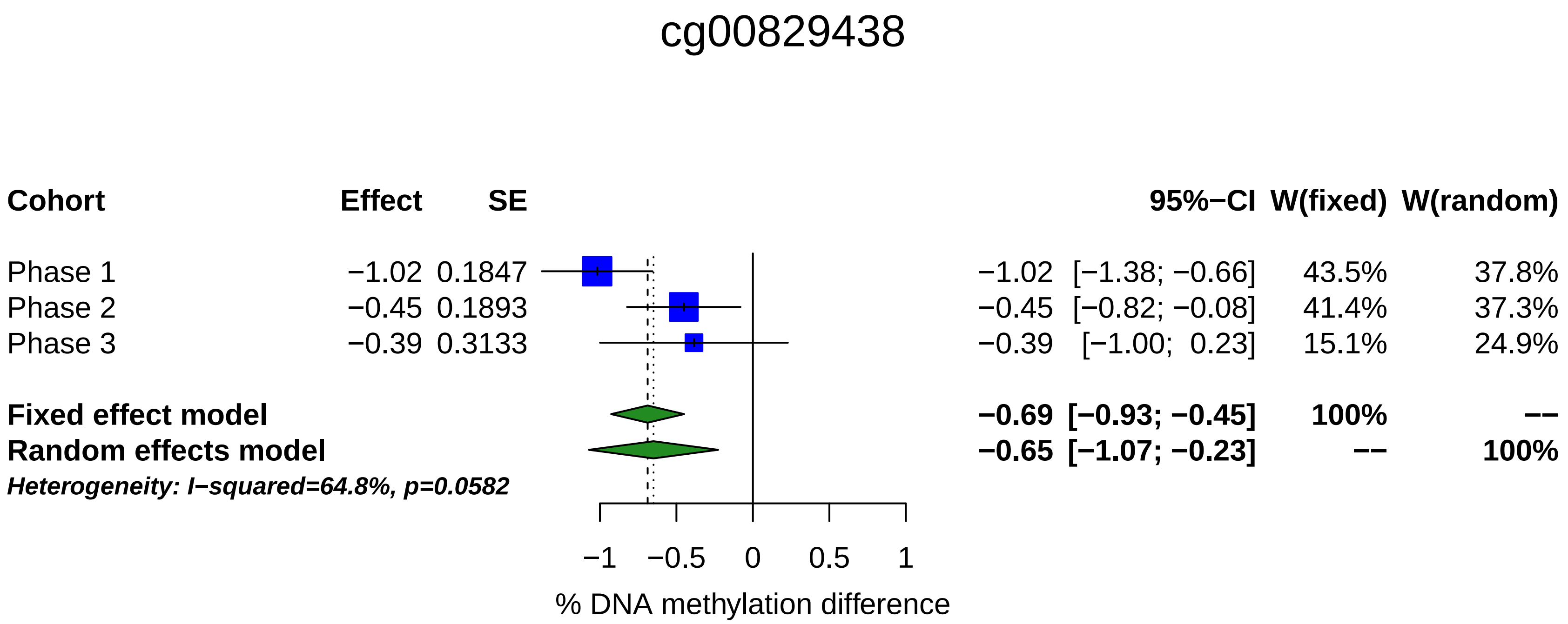
cg00829438
Cohort
Effect
SE
95%−CI
W(fixed)
W(random)
Phase 1 −1.02 0.1847 −1.02 [−1.38; −0.66] 43.5% 37.8%
Phase 2 −0.45 0.1893 −0.45 [−0.82; −0.08] 41.4% 37.3%
Phase 3 −0.39 0.3133 −0.39 [−1.00; 0.23] 15.1% 24.9%
Fixed effect model −0.69 [−0.93; −0.45] 100% −−
Random effects model −0.65 [−1.07; −0.23] −− 100%
Heterogeneity: I−squared=64.8%, p=0.0582
−1
−0.5
0
0.5
1
% DNA methylation difference

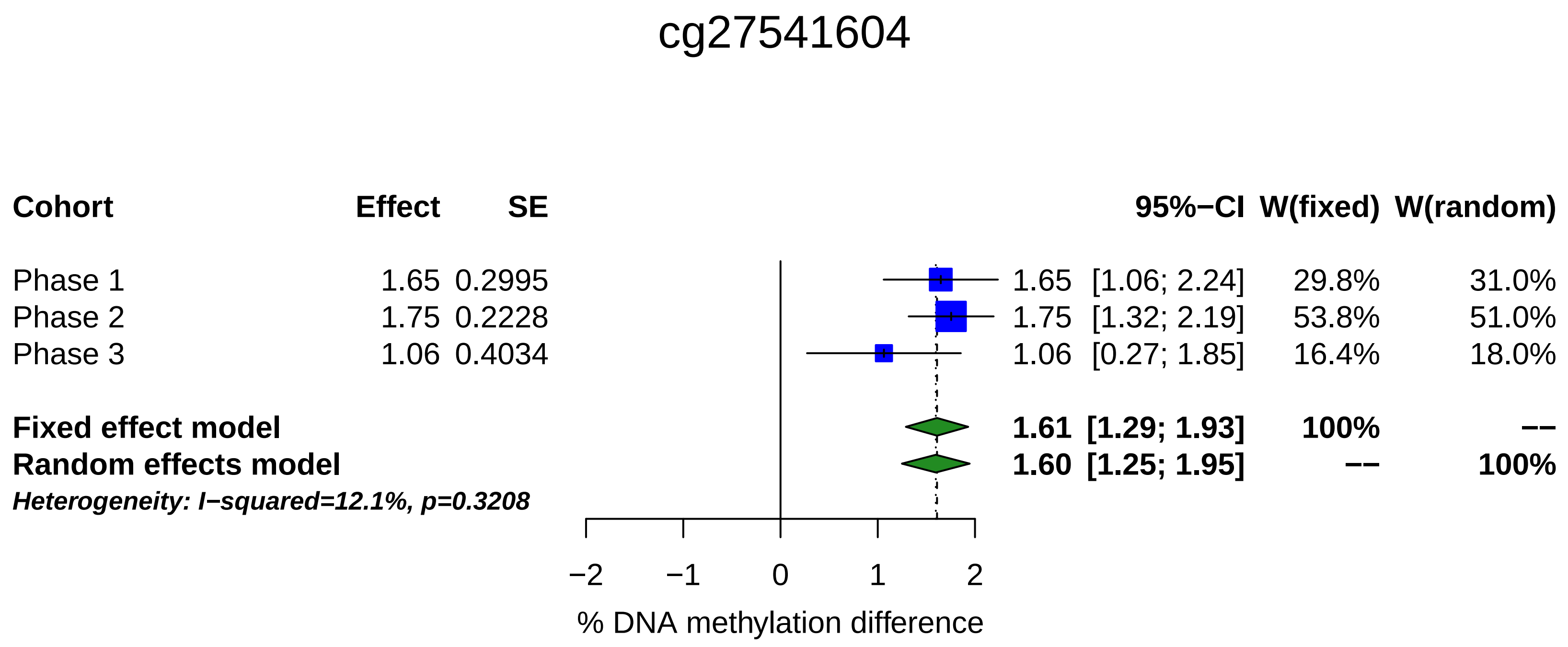
cg27541604
Cohort
Effect
SE
95%−CI
W(fixed)
W(random)
Phase 1 1.65 0.2995 1.65 [1.06; 2.24] 29.8% 31.0%
Phase 2 1.75 0.2228 1.75 [1.32; 2.19] 53.8% 51.0%
Phase 3 1.06 0.4034 1.06 [0.27; 1.85] 16.4% 18.0%
Fixed effect model 1.61 [1.29; 1.93] 100% −−
Random effects model 1.60 [1.25; 1.95] −− 100%
Heterogeneity: I−squared=12.1%, p=0.3208
−2
−1
0
1
2
% DNA methylation difference

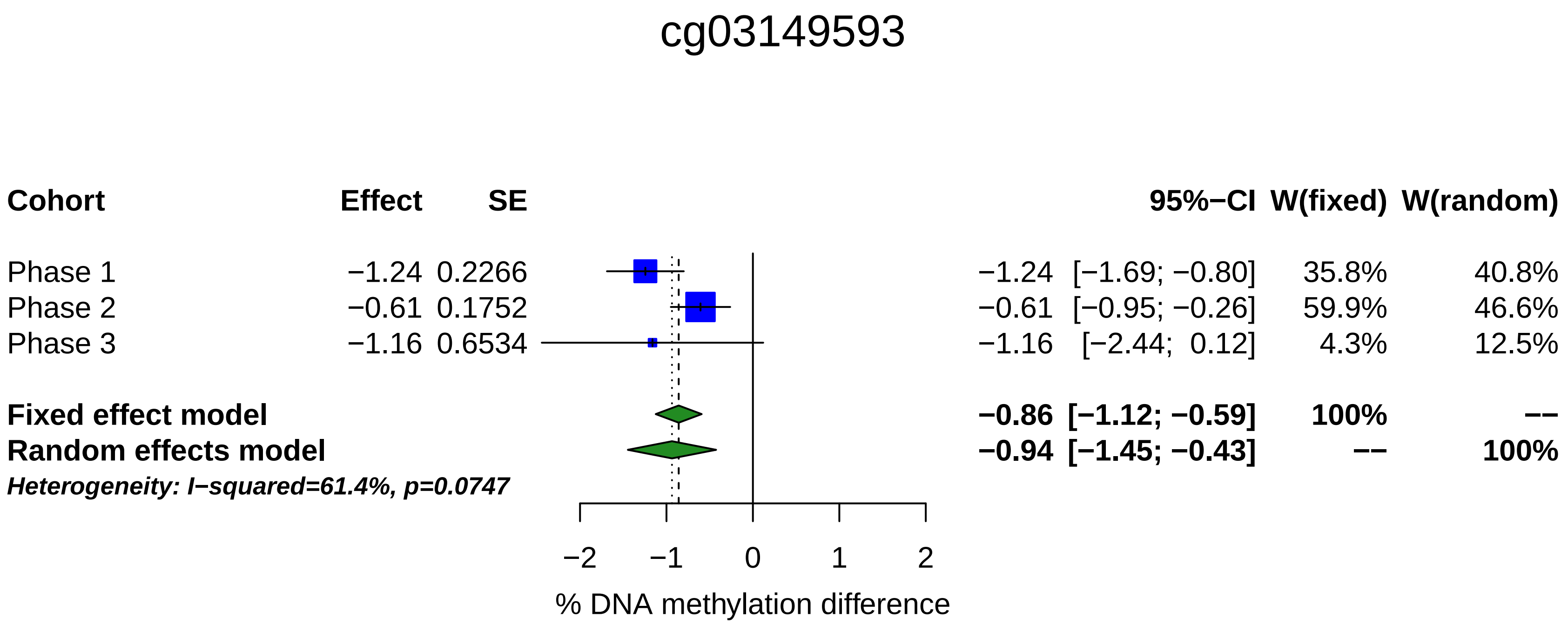
cg03149593
Cohort
Effect
SE
95%−CI
W(fixed)
W(random)
Phase 1 −1.24 0.2266 −1.24 [−1.69; −0.80] 35.8% 40.8%
Phase 2 −0.61 0.1752 −0.61 [−0.95; −0.26] 59.9% 46.6%
Phase 3 −1.16 0.6534 −1.16 [−2.44; 0.12] 4.3% 12.5%
Fixed effect model −0.86 [−1.12; −0.59] 100% −−
Random effects model −0.94 [−1.45; −0.43] −− 100%
Heterogeneity: I−squared=61.4%, p=0.0747
−2
−1
0
1
2
% DNA methylation difference

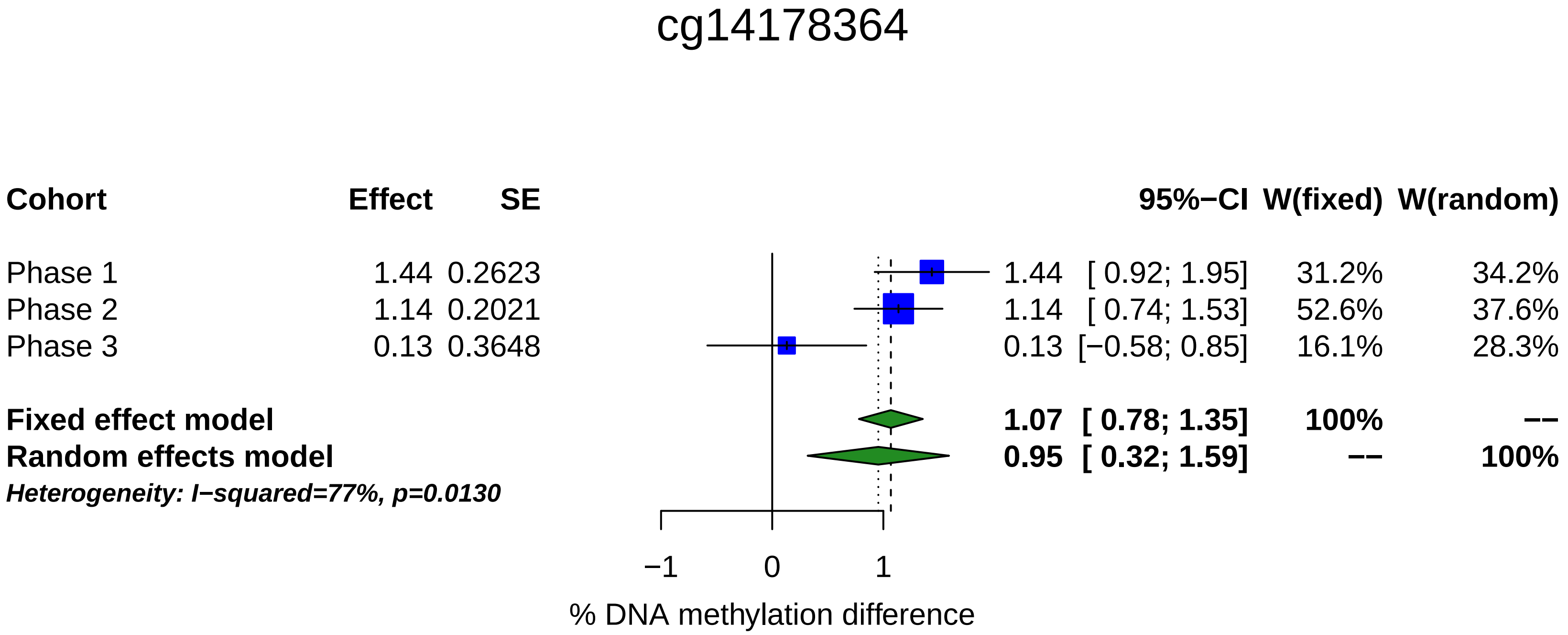
cg14178364
Cohort
Effect
SE
95%−CI
W(fixed)
W(random)
Phase 1 1.44 0.2623 1.44 [0.92; 1.95] 31.2% 34.2%
Phase 2 1.14 0.2021 1.14 [0.74; 1.53] 52.6% 37.6%
Phase 3 0.13 0.3648 0.13 [−0.58; 0.85] 16.1% 28.3%
Fixed effect model 1.07 [0.78; 1.35] 100% −−
Random effects model 0.95 [0.32; 1.59] −− 100%
Heterogeneity: I−squared=77%, p=0.0130
−1
0
1
% DNA methylation difference

cg14038731

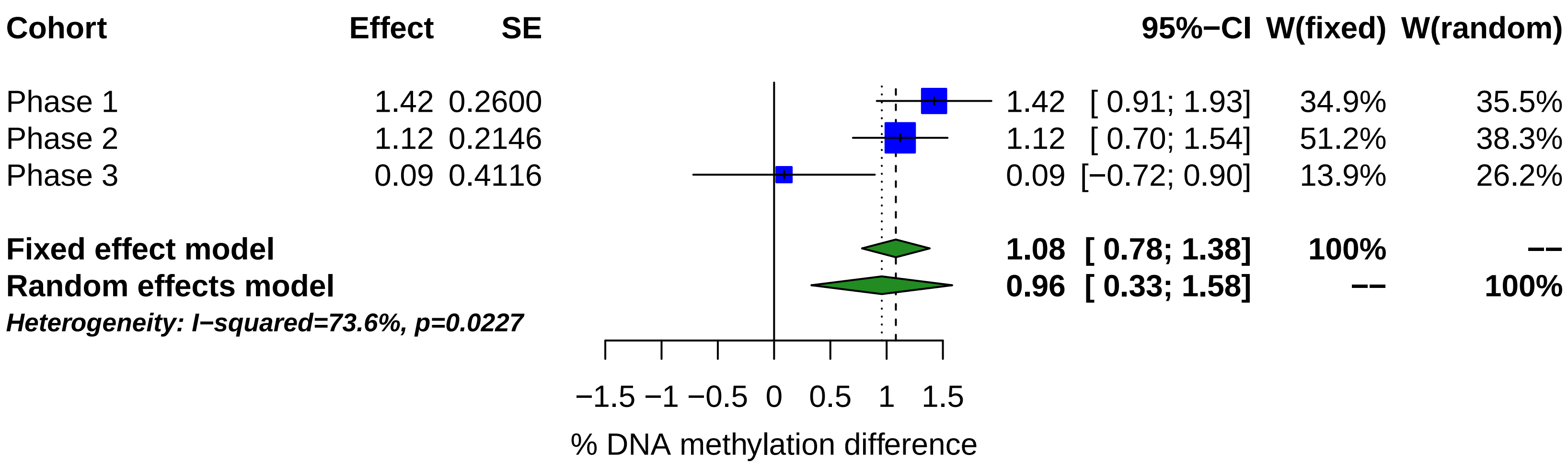

Cohort
Effect
SE
95%−CI
W(fixed)
W(random)
Phase 1 1.42 0.2600 1.42 [0.91; 1.93] 34.9% 35.5%
Phase 2 1.12 0.2146 1.12 [0.70; 1.54] 51.2% 38.3%
Phase 3 0.09 0.4116 0.09 [−0.72; 0.90] 13.9% 26.2%
Fixed effect model 1.08 [0.78; 1.38] 100% −−
Random effects model 0.96 [0.33; 1.58] −− 100%
Heterogeneity: I−squared=73.6%, p=0.0227
−1.5 −1 −0.5 0 0.5 1 1.5
% DNA methylation difference

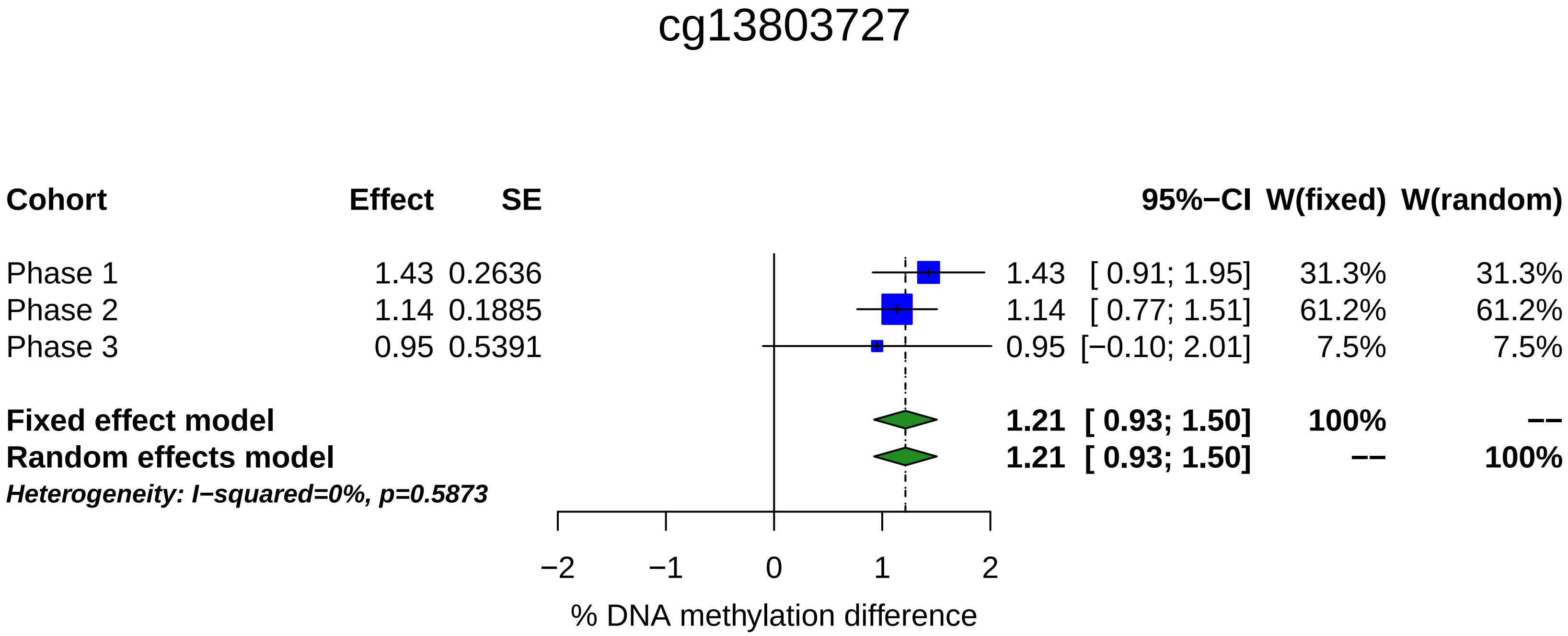
cg13803727
Cohort
Effect
SE
95%−CI
W(fixed)
W(random)
Phase 1 1.43 0.2636 1.43 [0.91; 1.95] 31.3% 31.3%
Phase 2 1.14 0.1885 1.14 [0.77; 1.51] 61.2% 61.2%
Phase 3 0.95 0.5391 0.95 [−0.10; 2.01] 7.5% 7.5%
Fixed effect model 1.21 [0.93; 1.50] 100% −−
Random effects model 1.21 [0.93; 1.50] −− 100%
Heterogeneity: I−squared=0%, p=0.5873
−2
−1
0
1
2
% DNA methylation difference

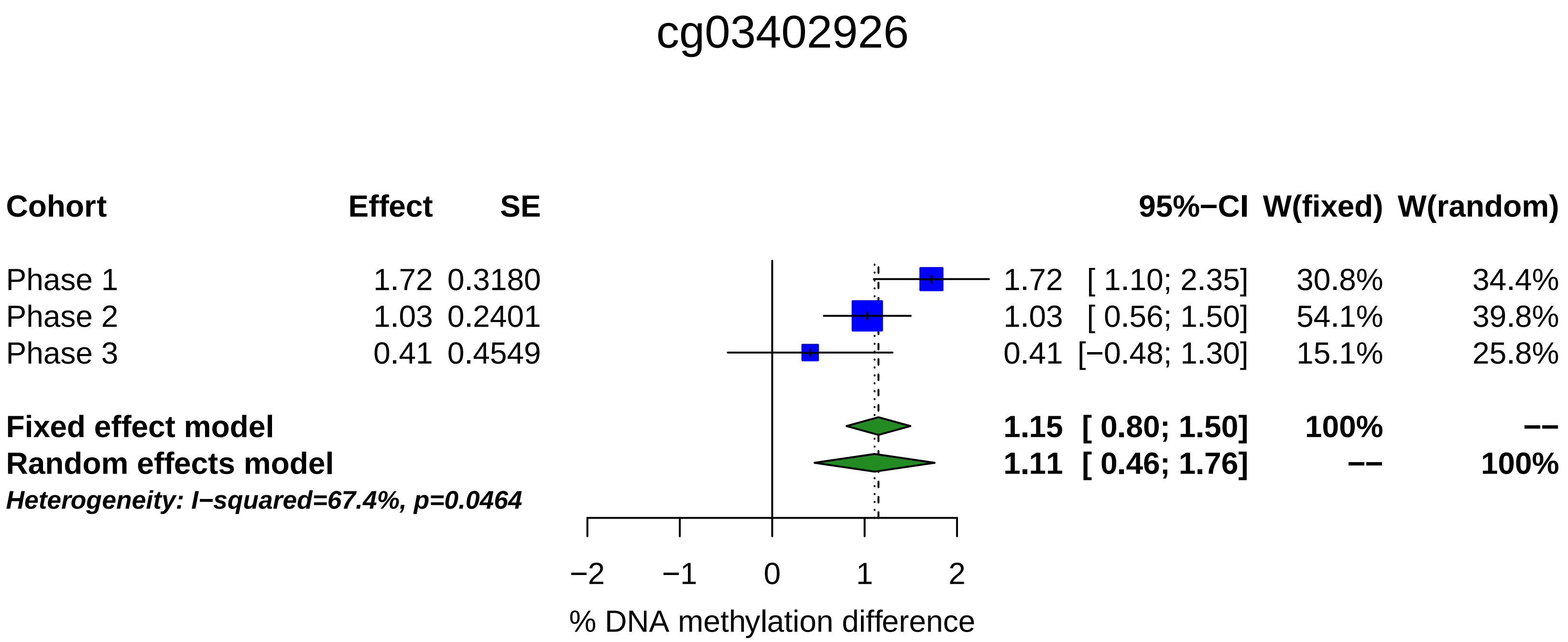
cg03402926
Cohort
Effect
SE
95%−CI
W(fixed)
W(random)
Phase 1 1.72 0.3180 1.72 [1.10; 2.35] 30.8% 34.4%
Phase 2 1.03 0.2401 1.03 [0.56; 1.50] 54.1% 39.8%
Phase 3 0.41 0.4549 0.41 [−0.48; 1.30] 15.1% 25.8%
Fixed effect model 1.15 [0.80; 1.50] 100% −−
Random effects model 1.11 [0.46; 1.76] −− 100%
Heterogeneity: I−squared=67.4%, p=0.0464
−2
−1
0
1
2
% DNA methylation difference

cg07326387

Cohort	Effect	SE	95%−CI	W(fixed)	W(random)
Phase 1	−2.10	0.3878	−2.10 [−2.86; −1.34]	28.6%	35.8%
Phase 2	−1.31	0.2580	−1.31 [−1.81; −0.80]	64.6%	50.8%
Phase 3	−2.21	0.7941	−2.21 [−3.77; −0.65]	6.8%	13.3%
**Fixed effect model**			**−1.59 [−2.00; −1.19]**	**100%**	**−−**
**Random effects model**			**−1.71 [−2.34; −1.09]**	**−−**	**100%**

Heterogeneity: I−squared=43.5%, p=0.1702

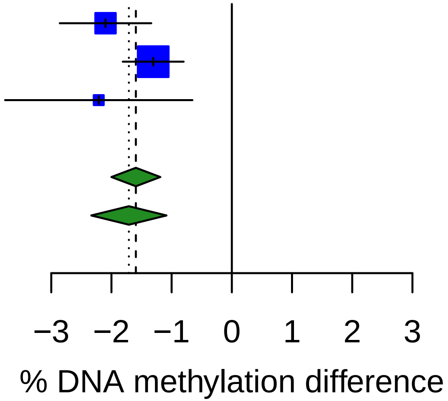

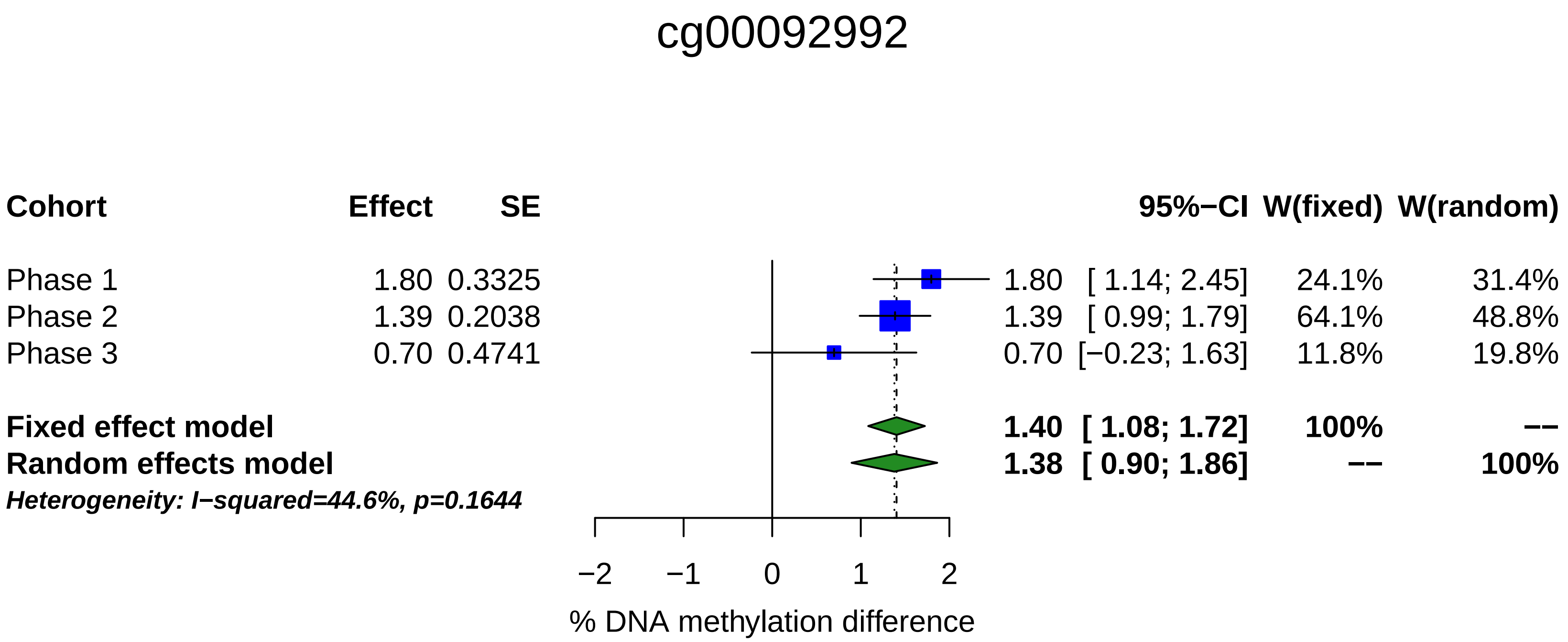
cg00092992
Cohort
Effect
SE
95%−CI
W(fixed)
W(random)
Phase 1 1.80 0.3325 1.80 [1.14; 2.45] 24.1% 31.4%
Phase 2 1.39 0.2038 1.39 [0.99; 1.79] 64.1% 48.8%
Phase 3 0.70 0.4741 0.70 [−0.23; 1.63] 11.8% 19.8%
Fixed effect model 1.40 [1.08; 1.72] 100% −−
Random effects model 1.38 [0.90; 1.86] −− 100%
Heterogeneity: I−squared=44.6%, p=0.1644
−2
−1
0
1
2
% DNA methylation difference

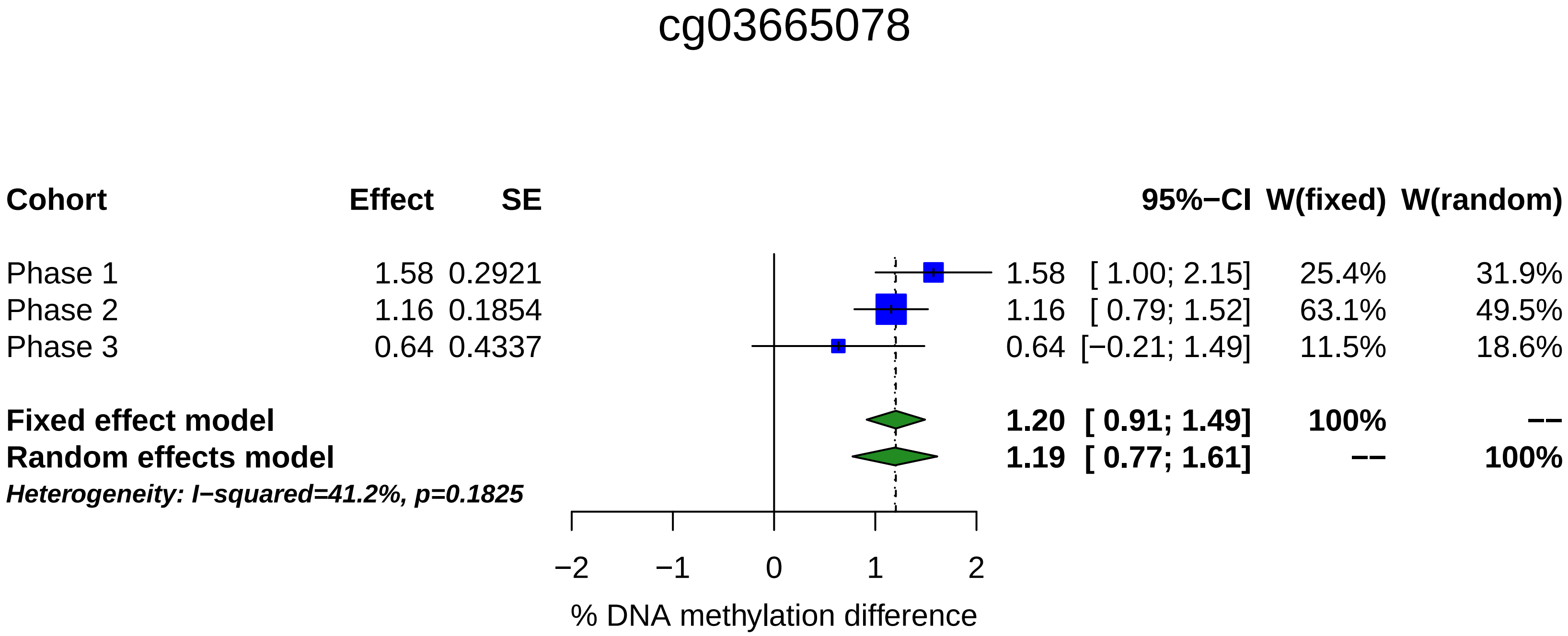
cg03665078
Cohort
Effect
SE
95%−CI
W(fixed)
W(random)
Phase 1 1.58 0.2921 1.58 [1.00; 2.15] 25.4% 31.9%
Phase 2 1.16 0.1854 1.16 [0.79; 1.52] 63.1% 49.5%
Phase 3 0.64 0.4337 0.64 [−0.21; 1.49] 11.5% 18.6%
Fixed effect model 1.20 [0.91; 1.49] 100% −−
Random effects model 1.19 [0.77; 1.61] −− 100%
Heterogeneity: I−squared=41.2%, p=0.1825
−2
−1
0
1
2
% DNA methylation difference

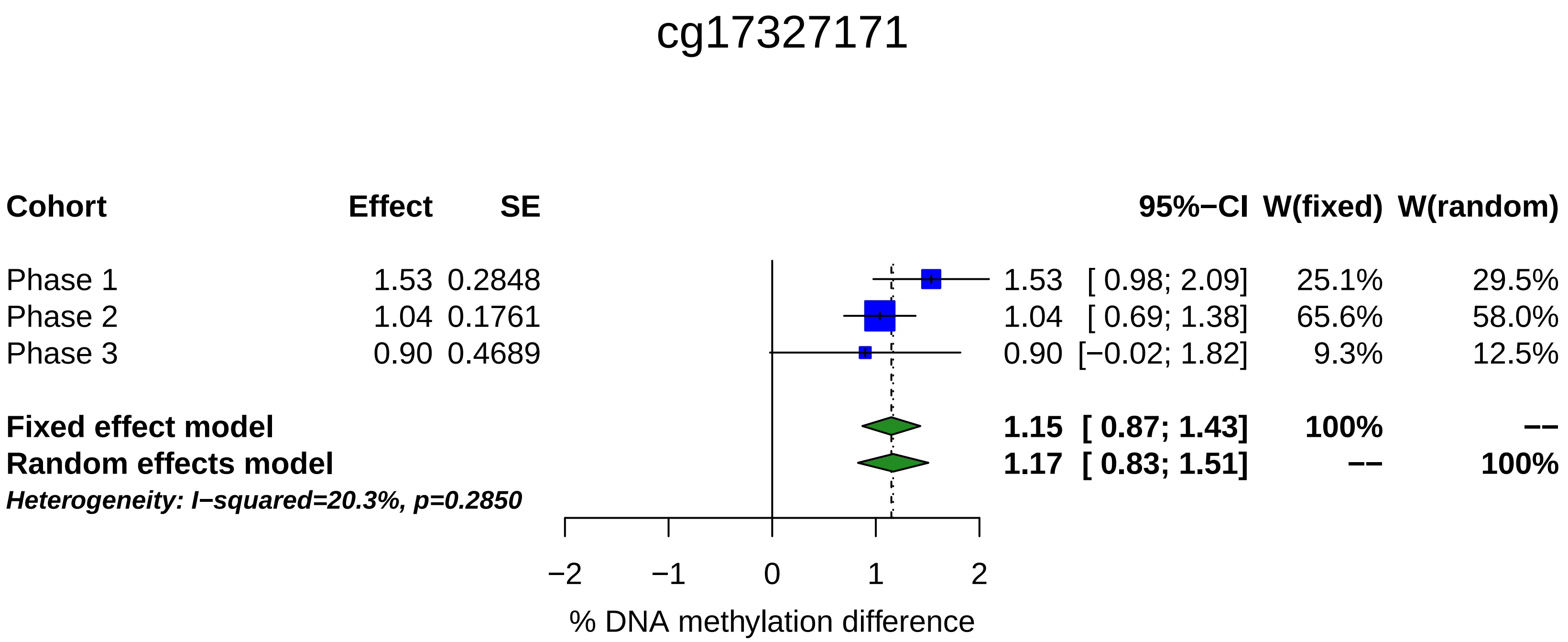

cg17327171
Cohort
Effect
SE
95%−CI
W(fixed)
W(random)
Phase 1 1.53 0.2848 1.53 [0.98; 2.09] 25.1% 29.5%
Phase 2 1.04 0.1761 1.04 [0.69; 1.38] 65.6% 58.0%
Phase 3 0.90 0.4689 0.90 [−0.02; 1.82] 9.3% 12.5%
Fixed effect model 1.15 [0.87; 1.43] 100% −−
Random effects model 1.17 [0.83; 1.51] −− 100%
Heterogeneity: I−squared=20.3%, p=0.2850
−2
−1
0
1
2
% DNA methylation difference

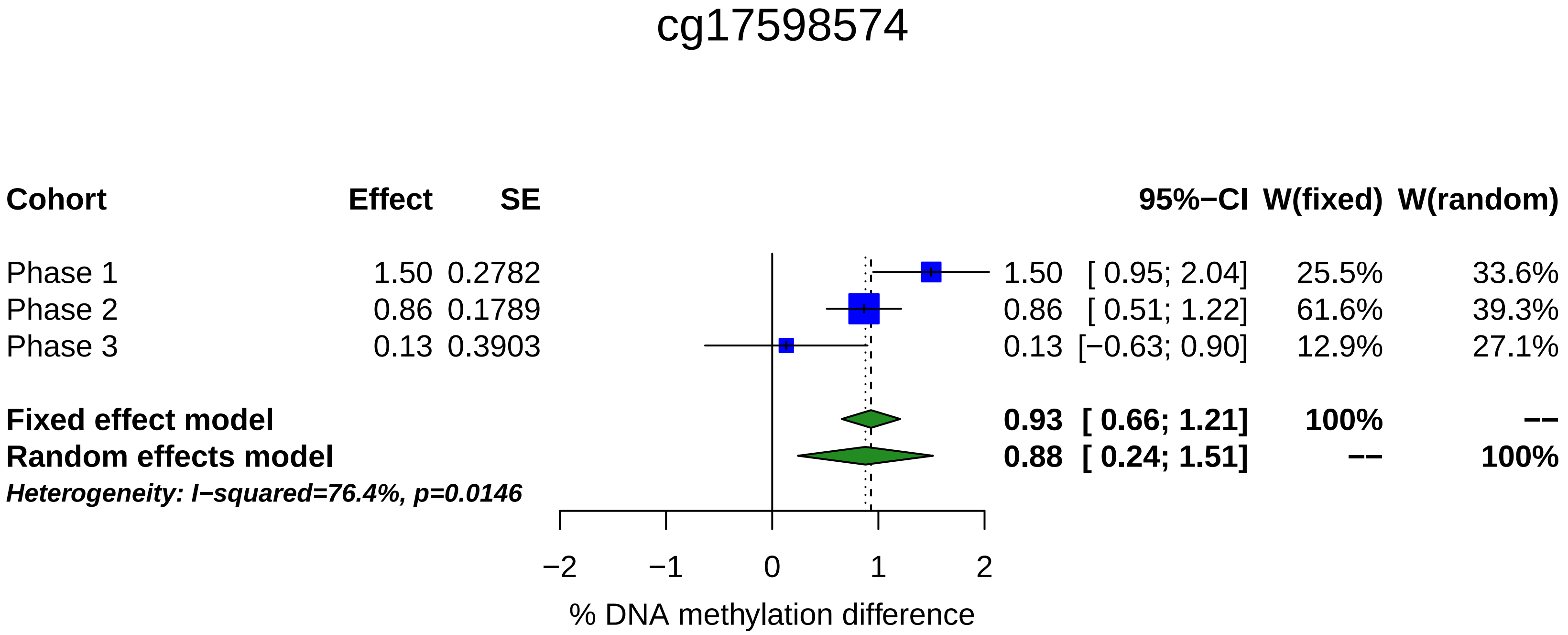
cg17598574
Cohort
Effect
SE
95%−CI
W(fixed)
W(random)
Phase 1 1.50 0.2782 1.50 [0.95; 2.04] 25.5% 33.6%
Phase 2 0.86 0.1789 0.86 [0.51; 1.22] 61.6% 39.3%
Phase 3 0.13 0.3903 0.13 [−0.63; 0.90] 12.9% 27.1%
Fixed effect model 0.93 [0.66; 1.21] 100% −−
Random effects model 0.88 [0.24; 1.51] −− 100%
Heterogeneity: I−squared=76.4%, p=0.0146
−2
−1
0
1
2
% DNA methylation difference

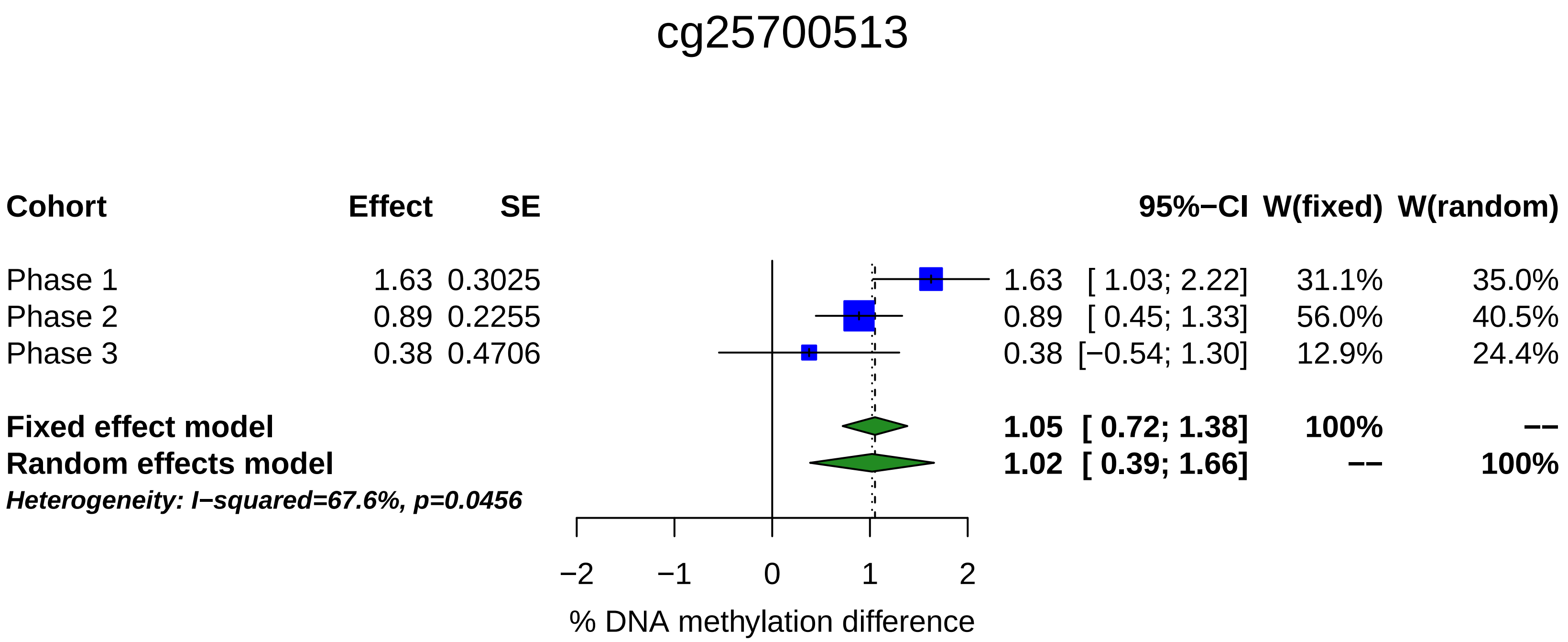
cg25700513
Cohort
Effect
SE
95%−CI
W(fixed)
W(random)
Phase 1
1.63
0.3025
1.63 [1.03; 2.22]
31.1%
35.0%
Phase 2
0.89
0.2255
0.89 [0.45; 1.33]
56.0%
40.5%
Phase 3
0.38
0.4706
0.38 [−0.54; 1.30]
12.9%
24.4%
Fixed effect model
1.05 [0.72; 1.38]
100%
−−
Random effects model
1.02 [0.39; 1.66]
−−
100%
Heterogeneity: I−squared=67.6%, p=0.0456
−2
−1
0
1
2
% DNA methylation difference

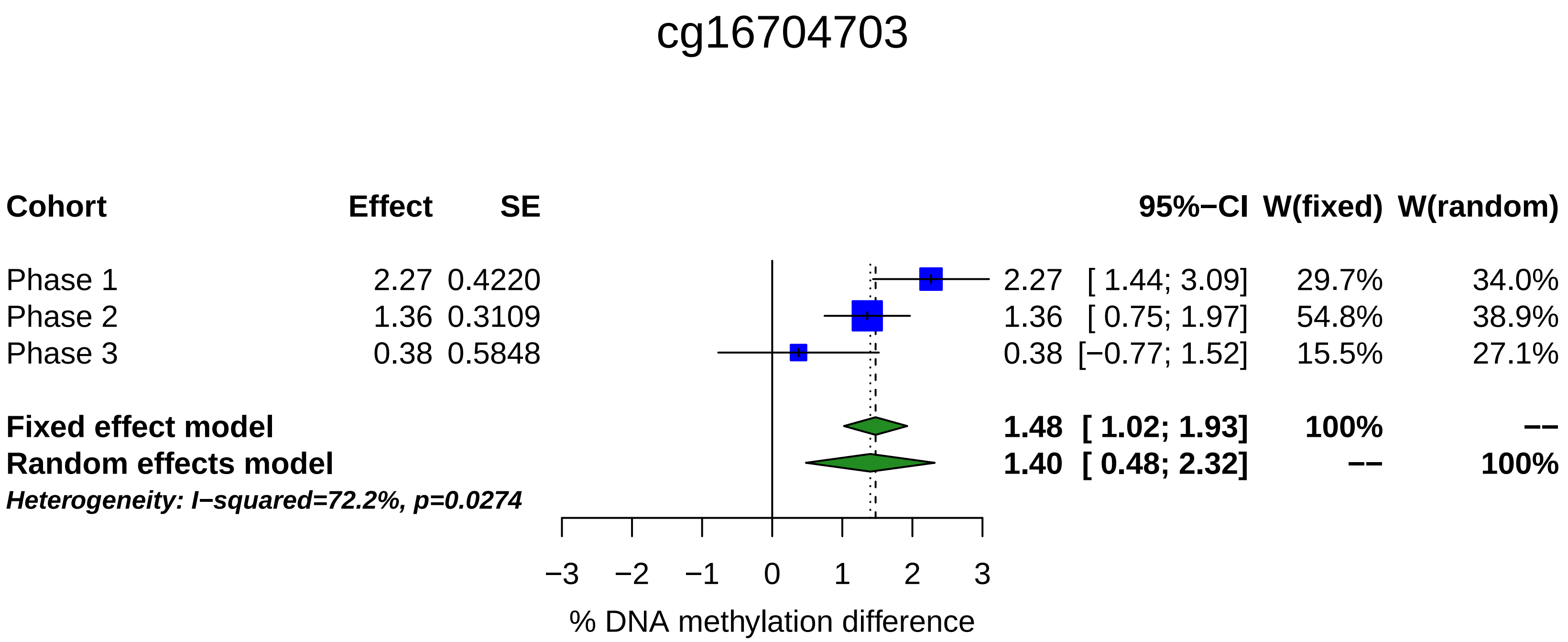
cg16704703
Cohort
Effect
SE
95%−CI
W(fixed)
W(random)
Phase 1 2.27 0.4220 2.27 [1.44; 3.09] 29.7% 34.0%
Phase 2 1.36 0.3109 1.36 [0.75; 1.97] 54.8% 38.9%
Phase 3 0.38 0.5848 0.38 [−0.77; 1.52] 15.5% 27.1%
Fixed effect model 1.48 [1.02; 1.93] 100% −−
Random effects model 1.40 [0.48; 2.32] −− 100%
Heterogeneity: I−squared=72.2%, p=0.0274
−3
−2
−1
0
1
2
3
% DNA methylation difference

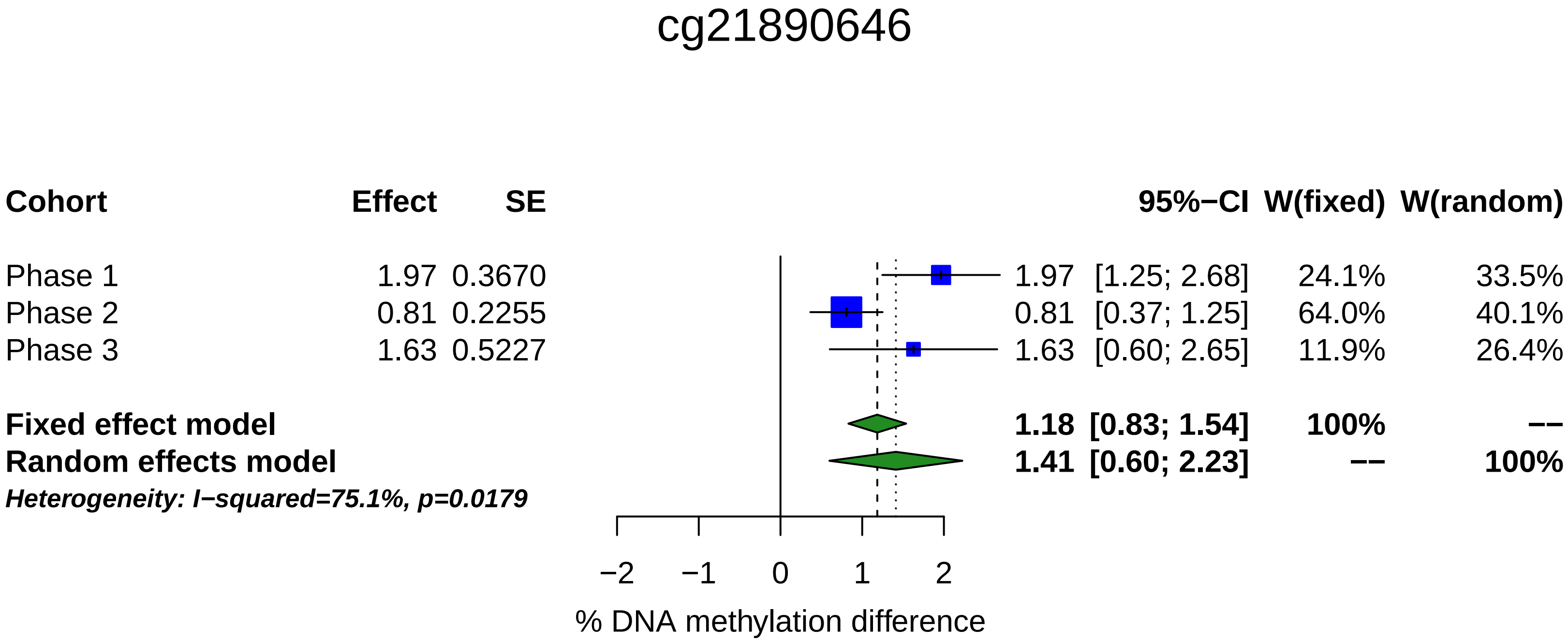
cg21890646
Cohort
Effect
SE
95%−CI
W(fixed)
W(random)
Phase 1 1.97 0.3670 1.97 [1.25; 2.68] 24.1% 33.5%
Phase 2 0.81 0.2255 0.81 [0.37; 1.25] 64.0% 40.1%
Phase 3 1.63 0.5227 1.63 [0.60; 2.65] 11.9% 26.4%
Fixed effect model 1.18 [0.83; 1.54] 100% −−
Random effects model 1.41 [0.60; 2.23] −− 100%
Heterogeneity: I−squared=75.1%, p=0.0179
−2
−1
0
1
2
% DNA methylation difference

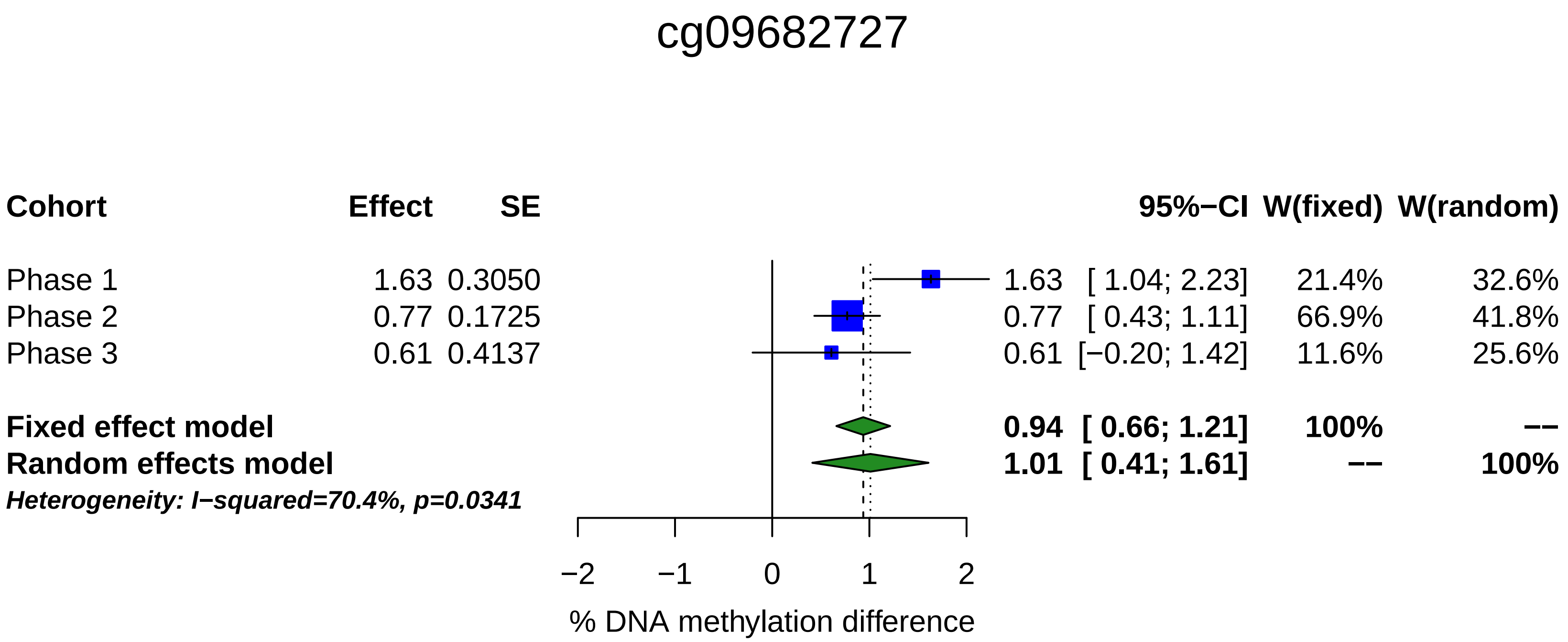
cg09682727
Cohort
Effect
SE
95%−CI
W(fixed)
W(random)
Phase 1 1.63 0.3050 1.63 [1.04; 2.23] 21.4% 32.6%
Phase 2 0.77 0.1725 0.77 [0.43; 1.11] 66.9% 41.8%
Phase 3 0.61 0.4137 0.61 [−0.20; 1.42] 11.6% 25.6%
Fixed effect model 0.94 [0.66; 1.21] 100% −−
Random effects model 1.01 [0.41; 1.61] −− 100%
Heterogeneity: I−squared=70.4%, p=0.0341
−2
−1
0
1
2
% DNA methylation difference

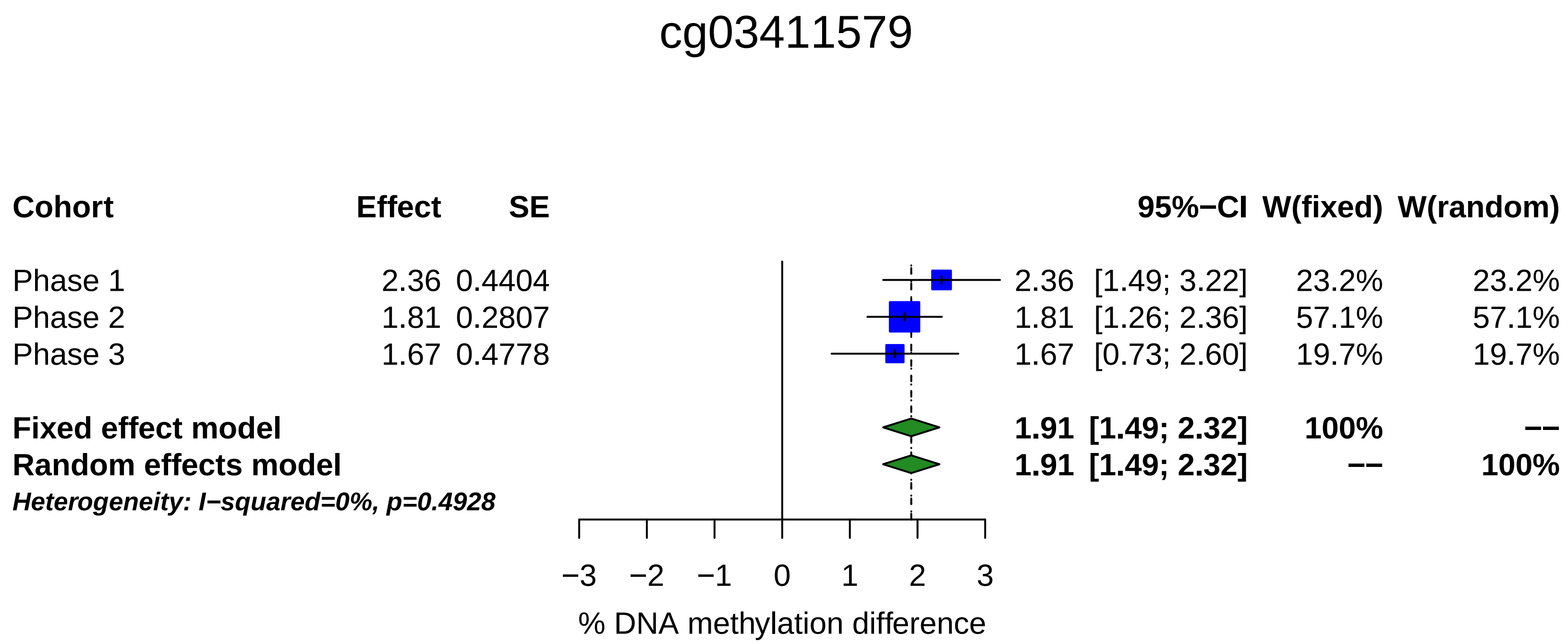
cg03411579
Cohort
Effect
SE
95%−CI
W(fixed)
W(random)
Phase 1
2.36
0.4404
2.36
[1.49; 3.22]
23.2%
23.2%
Phase 2
1.81
0.2807
1.81
[1.26; 2.36]
57.1%
57.1%
Phase 3
1.67
0.4778
1.67
[0.73; 2.60]
19.7%
19.7%
Fixed effect model
1.91
[1.49; 2.32]
100%
−−
Random effects model
1.91
[1.49; 2.32]
−−
100%
Heterogeneity: I−squared=0%, p=0.4928
−3
−2
−1
0
1
2
3
% DNA methylation difference

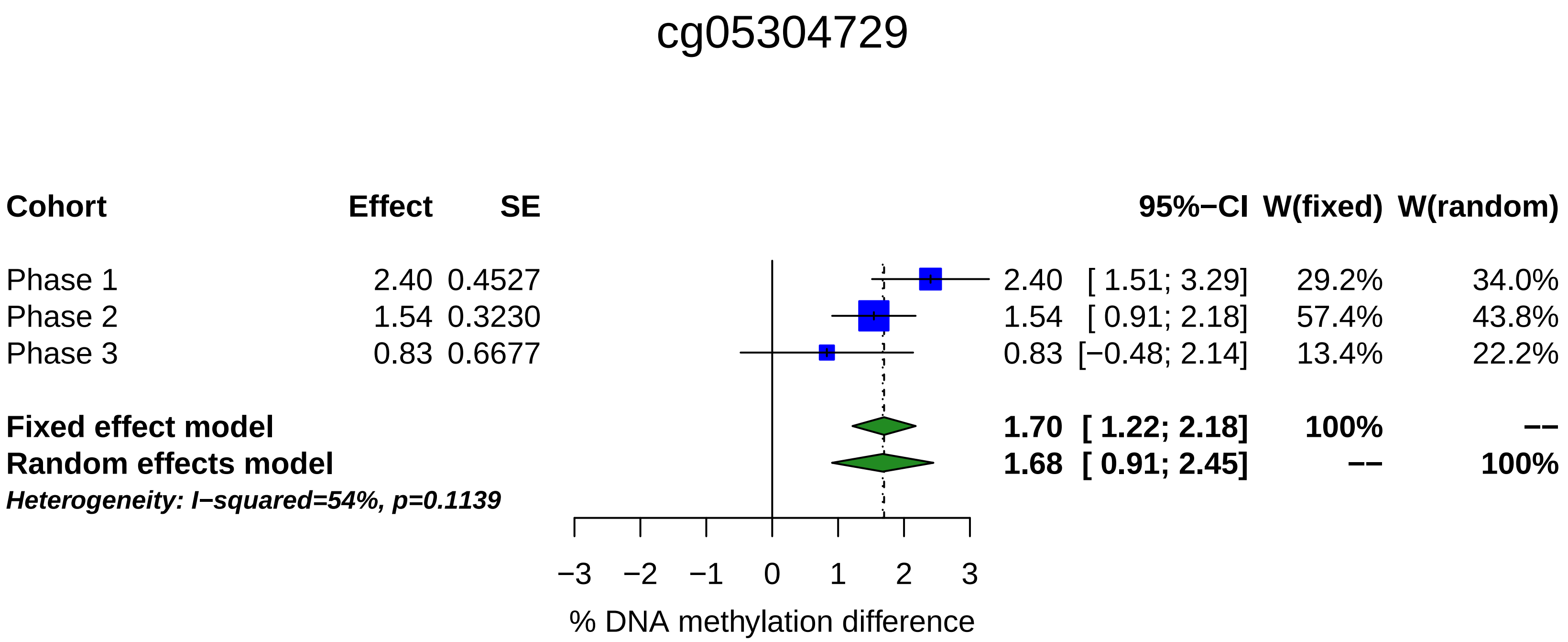
cg05304729
Cohort
Effect
SE
95%−CI
W(fixed)
W(random)
Phase 1 2.40 0.4527 2.40 [1.51; 3.29] 29.2% 34.0%
Phase 2 1.54 0.3230 1.54 [0.91; 2.18] 57.4% 43.8%
Phase 3 0.83 0.6677 0.83 [−0.48; 2.14] 13.4% 22.2%
Fixed effect model 1.70 [1.22; 2.18] 100% −−
Random effects model 1.68 [0.91; 2.45] −− 100%
Heterogeneity: I−squared=54%, p=0.1139
−3
−2
−1
0
1
2
3
% DNA methylation difference

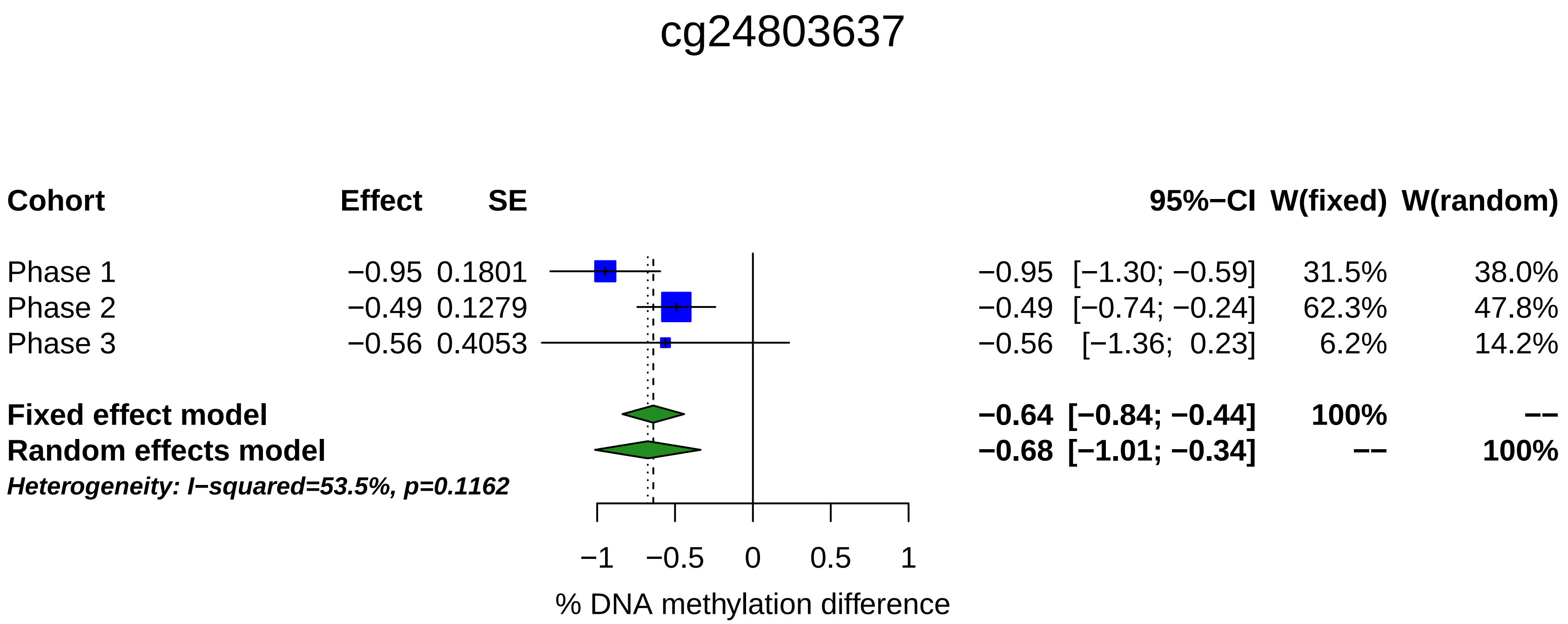

cg24803637
Cohort
Effect
SE
95%−CI
W(fixed)
W(random)
Phase 1 −0.95 0.1801 −0.95 [−1.30; −0.59] 31.5% 38.0%
Phase 2 −0.49 0.1279 −0.49 [−0.74; −0.24] 62.3% 47.8%
Phase 3 −0.56 0.4053 −0.56 [−1.36; 0.23] 6.2% 14.2%
Fixed effect model −0.64 [−0.84; −0.44] 100% --
Random effects model −0.68 [−1.01; −0.34] -- 100%
Heterogeneity: I−squared=53.5%, p=0.1162
−1
−0.5
0
0.5
1
% DNA methylation difference

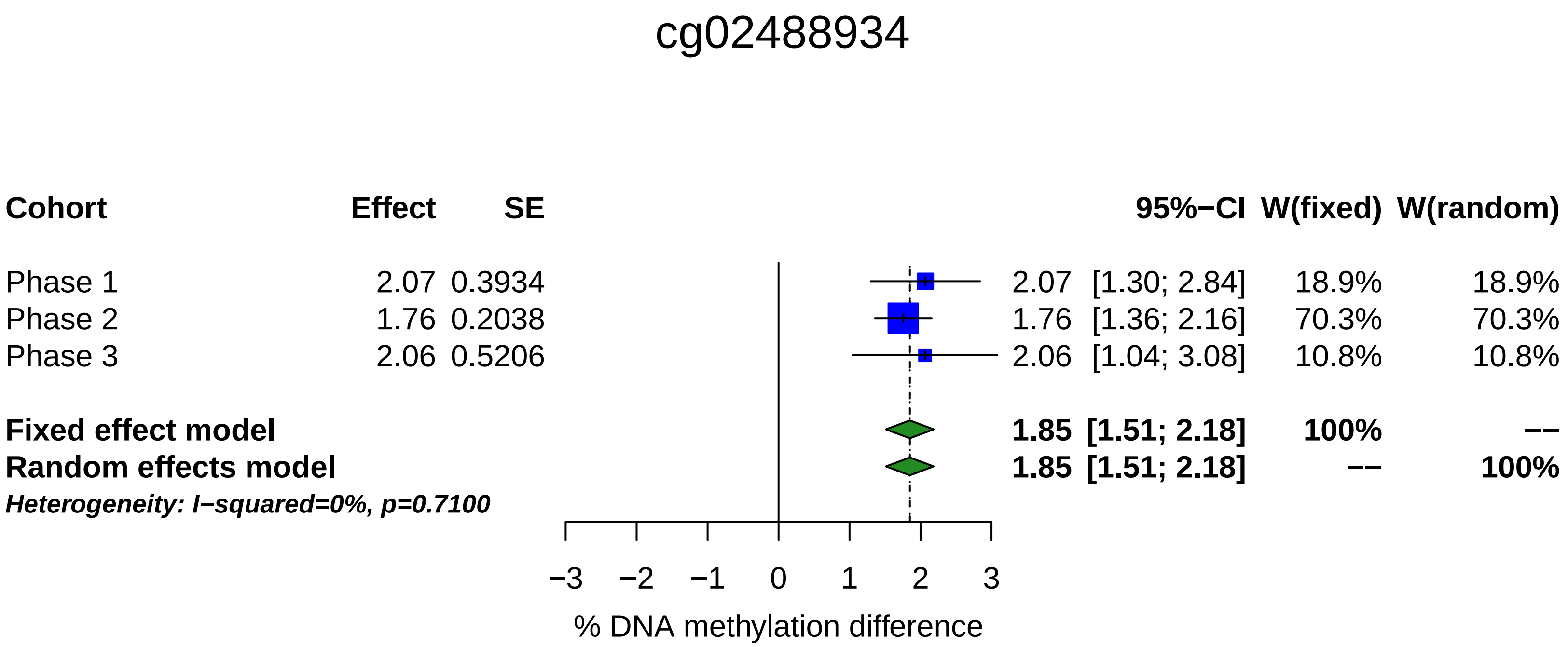
cg02488934
Cohort
Effect
SE
95%−CI
W(fixed)
W(random)
Phase 1 2.07 0.3934 2.07 [1.30; 2.84] 18.9% 18.9%
Phase 2 1.76 0.2038 1.76 [1.36; 2.16] 70.3% 70.3%
Phase 3 2.06 0.5206 2.06 [1.04; 3.08] 10.8% 10.8%
Fixed effect model 1.85 [1.51; 2.18] 100% −−
Random effects model 1.85 [1.51; 2.18] −− 100%
Heterogeneity: I−squared=0%, p=0.7100
−3 −2 −1 0 1 2 3
% DNA methylation difference

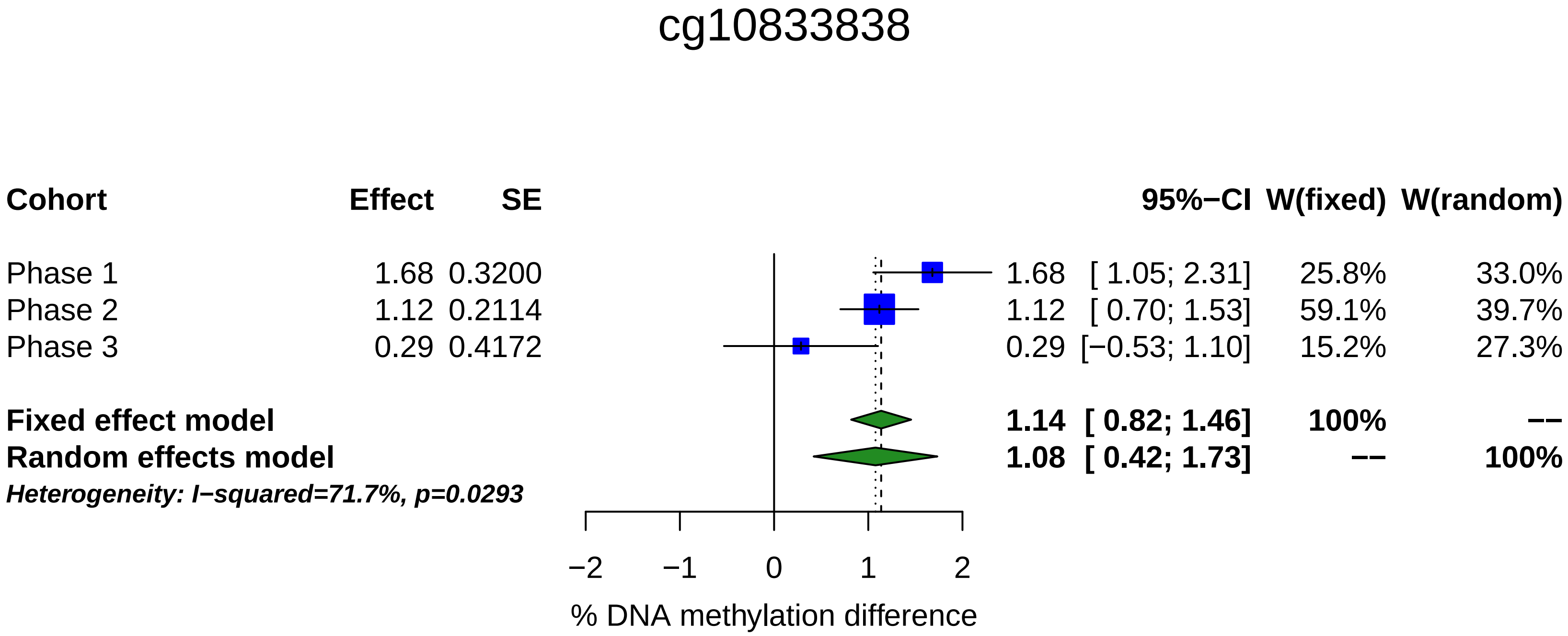
cg10833838
Cohort
Effect
SE
95%−CI
W(fixed)
W(random)
Phase 1 1.68 0.3200 1.68 [1.05; 2.31] 25.8% 33.0%
Phase 2 1.12 0.2114 1.12 [0.70; 1.53] 59.1% 39.7%
Phase 3 0.29 0.4172 0.29 [−0.53; 1.10] 15.2% 27.3%
Fixed effect model 1.14 [0.82; 1.46] 100% −−
Random effects model 1.08 [0.42; 1.73] −− 100%
Heterogeneity: I−squared=71.7%, p=0.0293
−2
−1
0
1
2
% DNA methylation difference

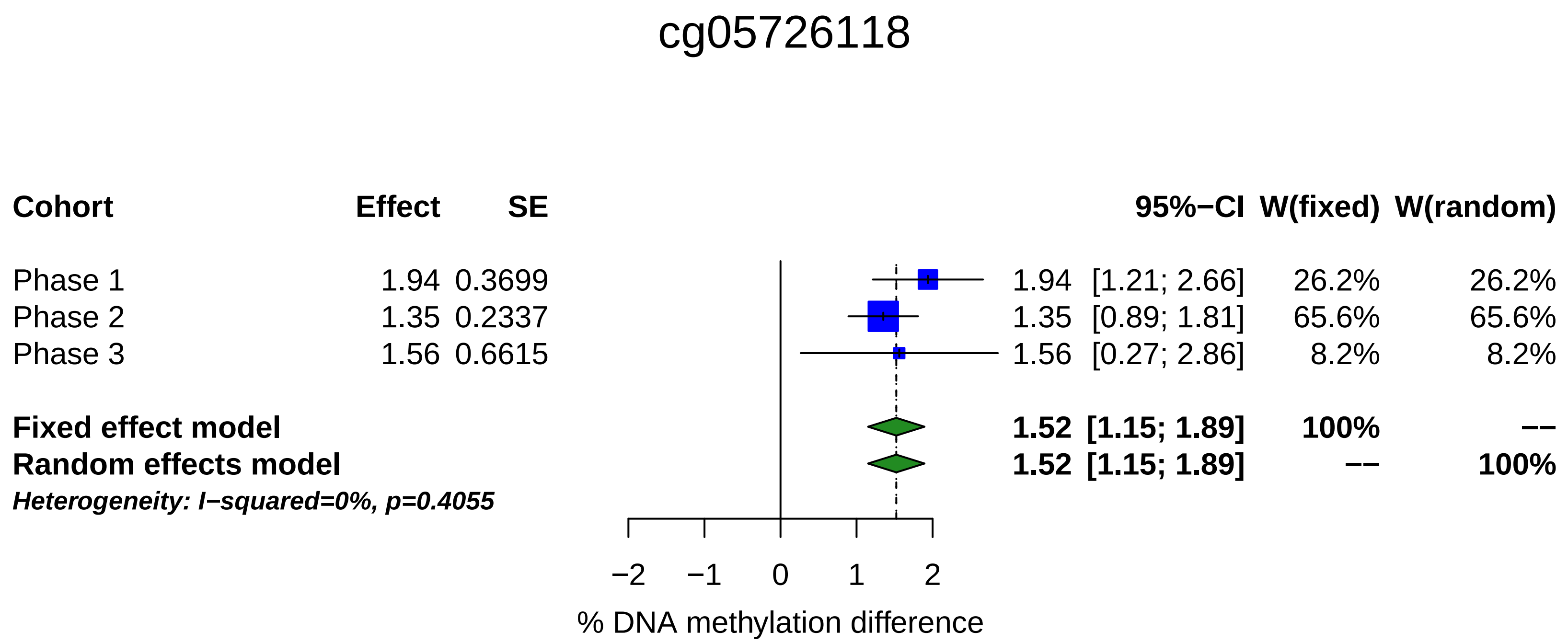
cg05726118
Cohort
Effect
SE
95%−CI
W(fixed)
W(random)
Phase 1
1.94
0.3699
1.94 [1.21; 2.66]
26.2%
26.2%
Phase 2
1.35
0.2337
1.35 [0.89; 1.81]
65.6%
65.6%
Phase 3
1.56
0.6615
1.56 [0.27; 2.86]
8.2%
8.2%
Fixed effect model
1.52 [1.15; 1.89]
100%
−−
Random effects model
1.52 [1.15; 1.89]
−−
100%
Heterogeneity: I−squared=0%, p=0.4055
−2
−1
0
1
2
% DNA methylation difference

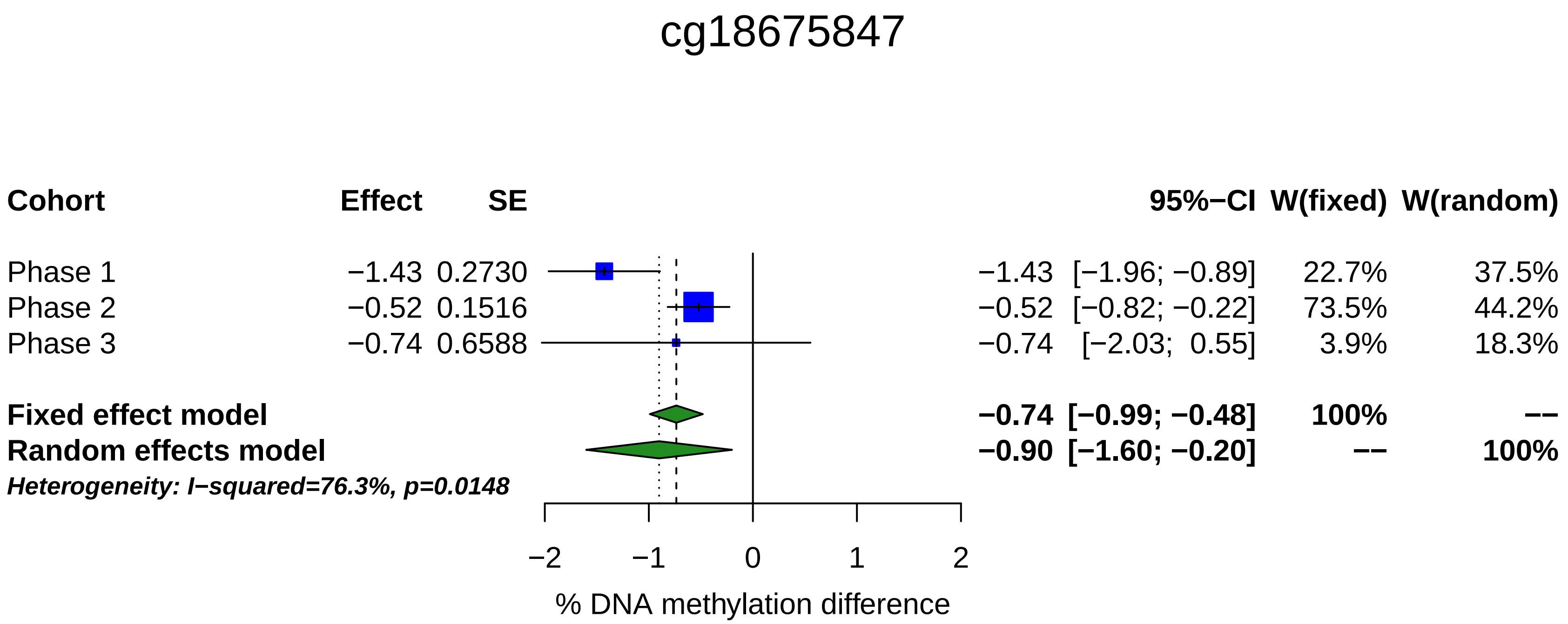
cg18675847
Cohort
Effect
SE
95%−CI
W(fixed)
W(random)
Phase 1 −1.43 0.2730 −1.43 [−1.96; −0.89] 22.7% 37.5%
Phase 2 −0.52 0.1516 −0.52 [−0.82; −0.22] 73.5% 44.2%
Phase 3 −0.74 0.6588 −0.74 [−2.03; 0.55] 3.9% 18.3%
Fixed effect model −0.74 [−0.99; −0.48] 100% −−
Random effects model −0.90 [−1.60; −0.20] −− 100%
Heterogeneity: I−squared=76.3%, p=0.0148
−2
−1
0
1
2
% DNA methylation difference

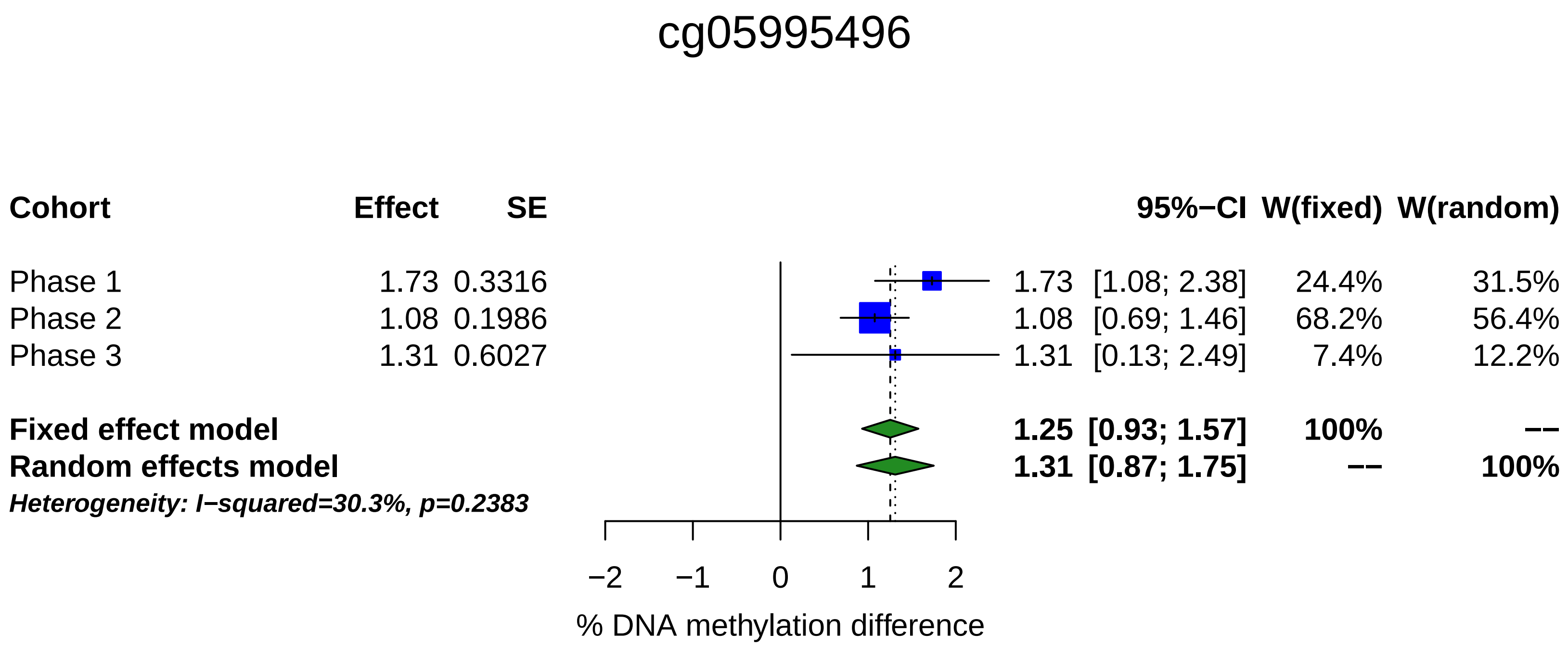
cg05995496
Cohort
Effect
SE
95%−CI
W(fixed)
W(random)
Phase 1 1.73 0.3316 1.73 [1.08; 2.38] 24.4% 31.5%
Phase 2 1.08 0.1986 1.08 [0.69; 1.46] 68.2% 56.4%
Phase 3 1.31 0.6027 1.31 [0.13; 2.49] 7.4% 12.2%
Fixed effect model 1.25 [0.93; 1.57] 100% −−
Random effects model 1.31 [0.87; 1.75] −− 100%
Heterogeneity: I−squared=30.3%, p=0.2383
−2
−1
0
1
2
% DNA methylation difference

cg23480341

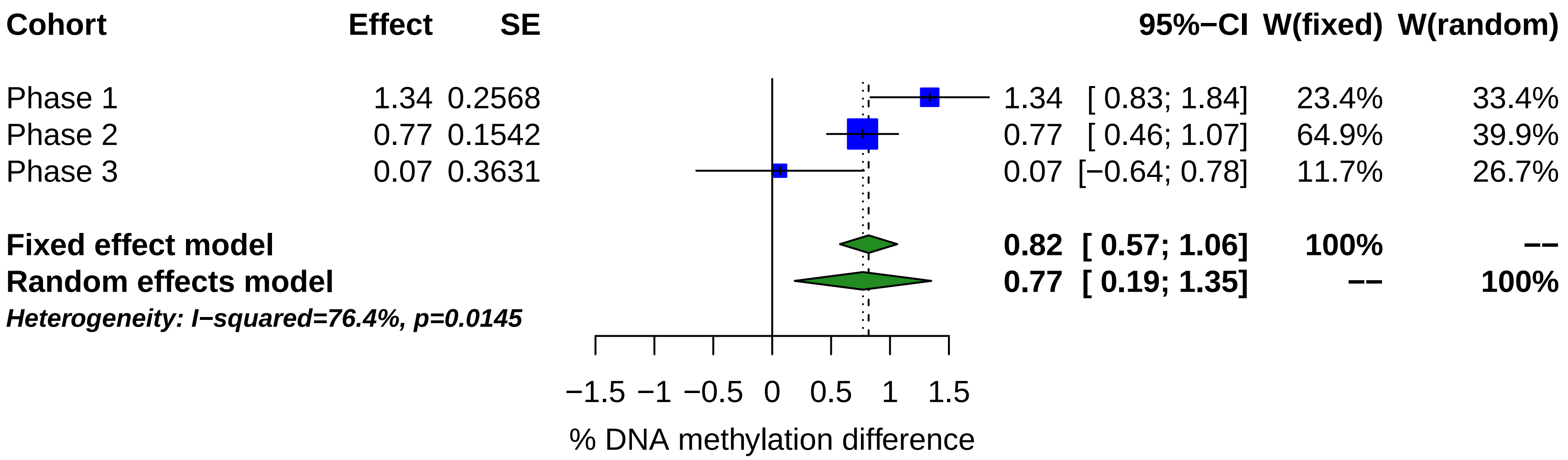
Cohort
Effect
SE
95%−CI
W(fixed)
W(random)
Phase 1 1.34 0.2568 1.34 [0.83; 1.84] 23.4% 33.4%
Phase 2 0.77 0.1542 0.77 [0.46; 1.07] 64.9% 39.9%
Phase 3 0.07 0.3631 0.07 [−0.64; 0.78] 11.7% 26.7%
Fixed effect model 0.82 [0.57; 1.06] 100% −−
Random effects model 0.77 [0.19; 1.35] −− 100%
Heterogeneity: I−squared=76.4%, p=0.0145
−1.5
−1
−0.5
0
0.5
1
1.5
% DNA methylation difference

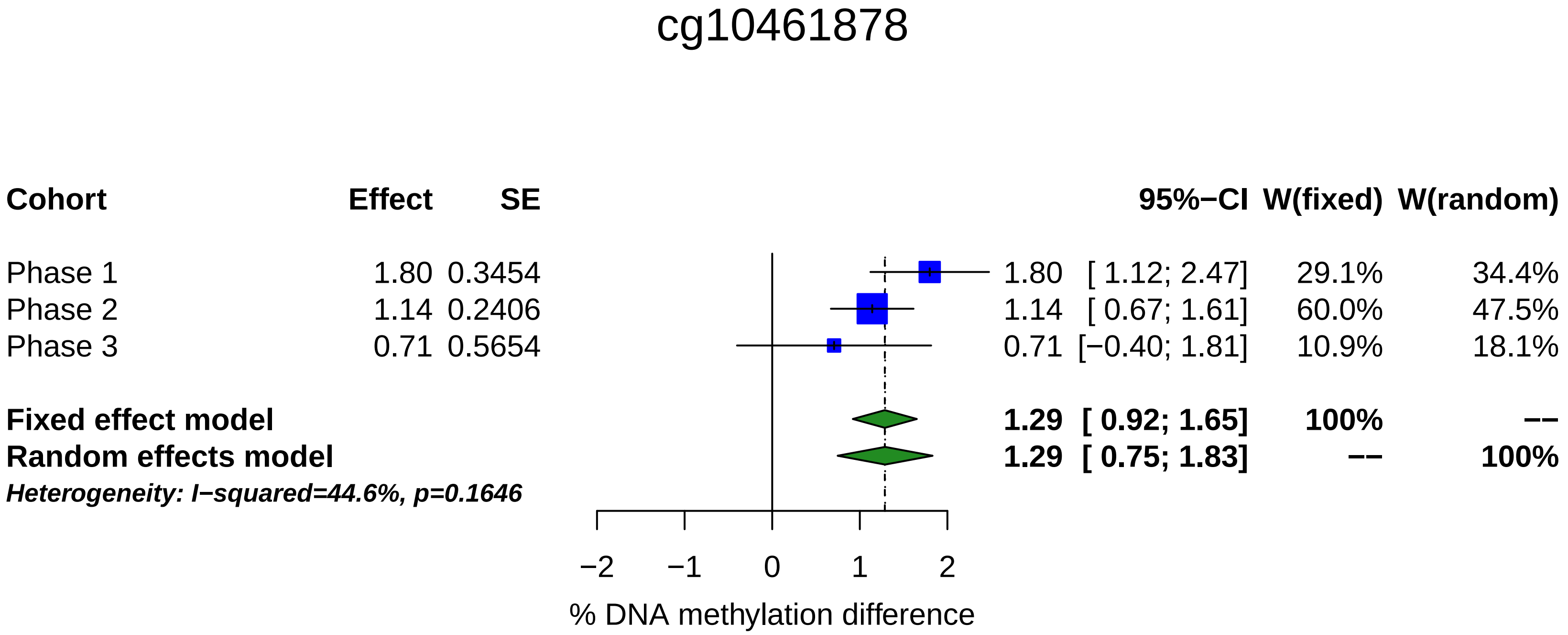
cg10461878
Cohort
Effect
SE
95%−CI
W(fixed)
W(random)
Phase 1
1.80
0.3454
1.80 [1.12; 2.47]
29.1%
34.4%
Phase 2
1.14
0.2406
1.14 [0.67; 1.61]
60.0%
47.5%
Phase 3
0.71
0.5654
0.71 [−0.40; 1.81]
10.9%
18.1%
Fixed effect model
1.29 [0.92; 1.65]
100%
−−
Random effects model
1.29 [0.75; 1.83]
−−
100%
Heterogeneity: I−squared=44.6%, p=0.1646
−2
−1
0
1
2
% DNA methylation difference

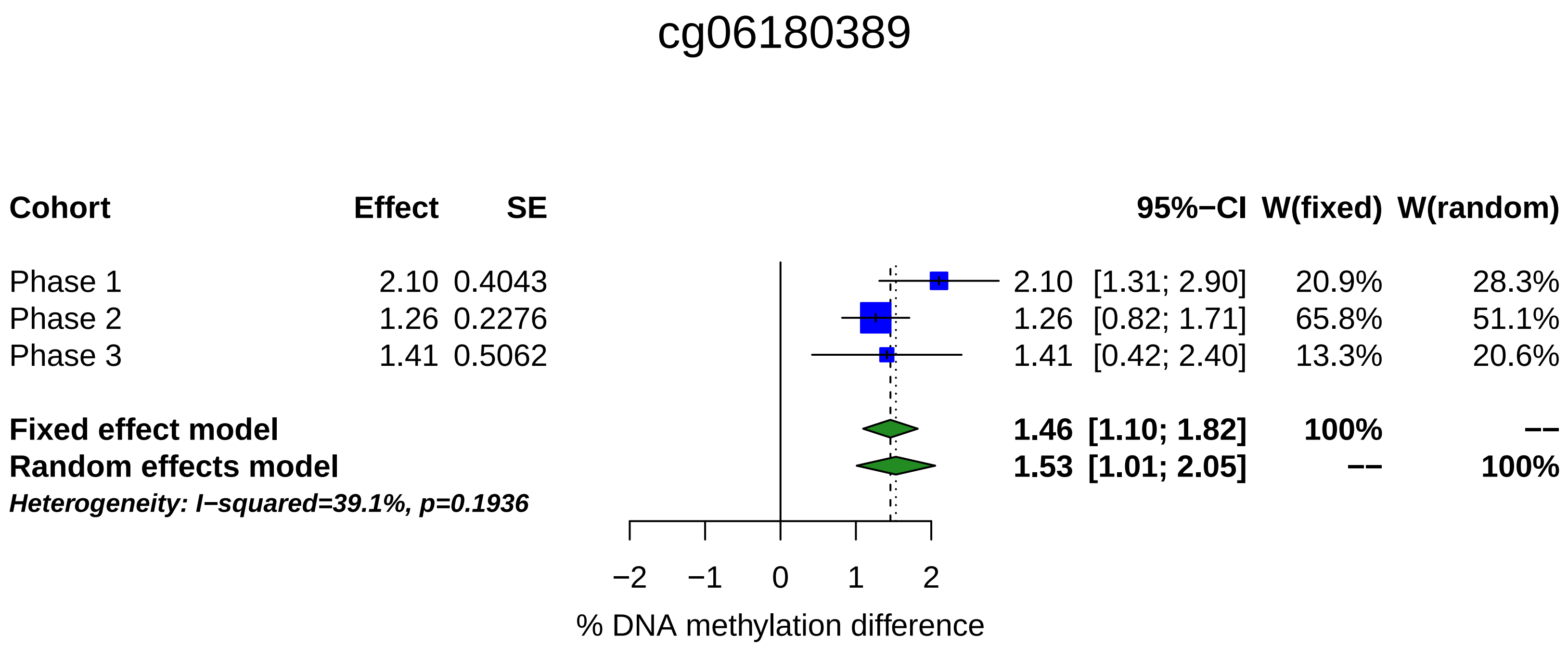
cg06180389
Cohort
Effect
SE
95%−CI
W(fixed)
W(random)
Phase 1 2.10 0.4043 2.10 [1.31; 2.90] 20.9% 28.3%
Phase 2 1.26 0.2276 1.26 [0.82; 1.71] 65.8% 51.1%
Phase 3 1.41 0.5062 1.41 [0.42; 2.40] 13.3% 20.6%
Fixed effect model 1.46 [1.10; 1.82] 100% −−
Random effects model 1.53 [1.01; 2.05] −− 100%
Heterogeneity: I−squared=39.1%, p=0.1936
−2
−1
0
1
2
% DNA methylation difference

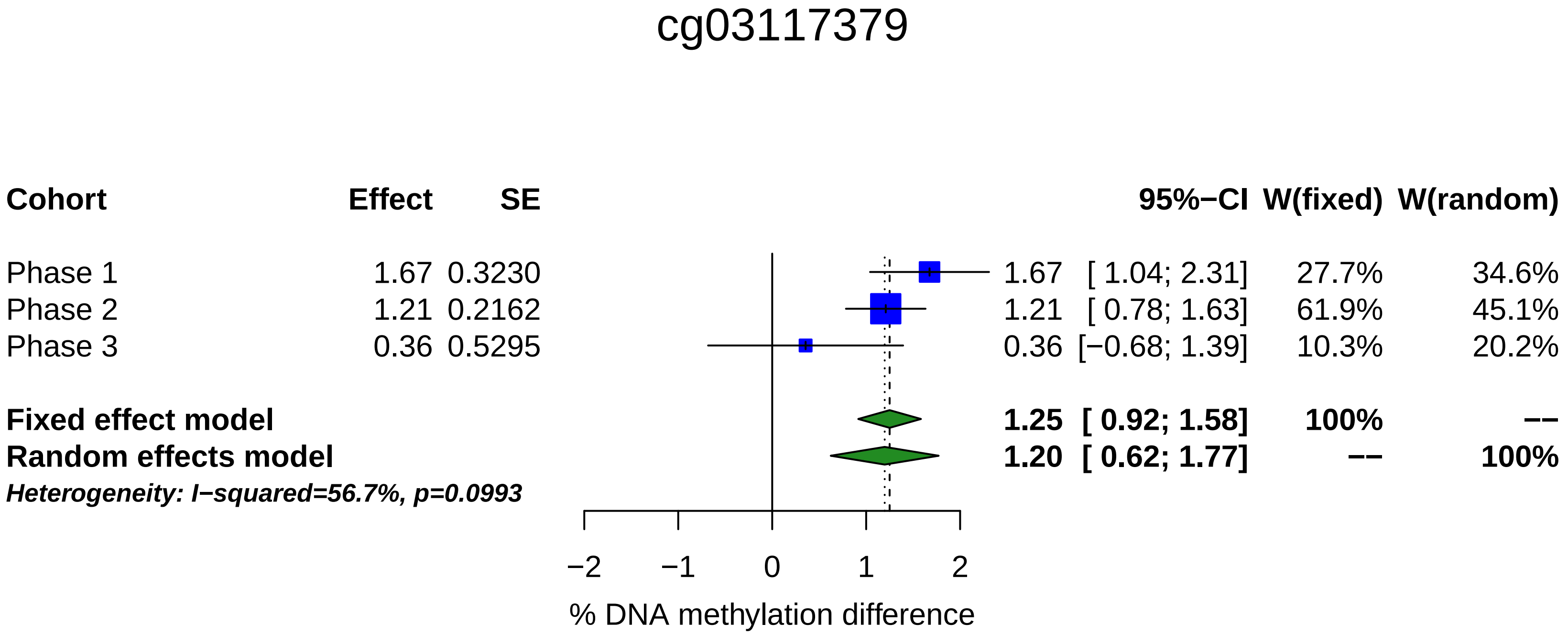
cg03117379
Cohort
Effect
SE
95%−CI
W(fixed)
W(random)
Phase 1 1.67 0.3230 1.67 [1.04; 2.31] 27.7% 34.6%
Phase 2 1.21 0.2162 1.21 [0.78; 1.63] 61.9% 45.1%
Phase 3 0.36 0.5295 0.36 [−0.68; 1.39] 10.3% 20.2%
Fixed effect model 1.25 [0.92; 1.58] 100% −−
Random effects model 1.20 [0.62; 1.77] −− 100%
Heterogeneity: I−squared=56.7%, p=0.0993
−2
−1
0
1
2
% DNA methylation difference

cg03655692

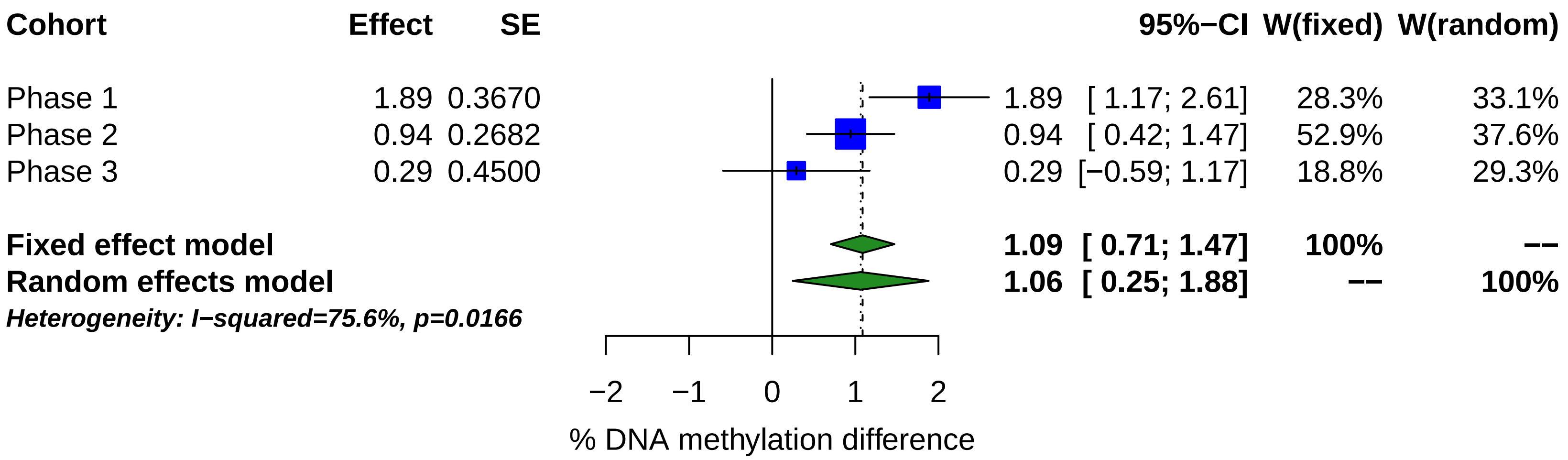
Cohort
Effect
SE
95%−CI
W(fixed)
W(random)
Phase 1 1.89 0.3670 1.89 [1.17; 2.61] 28.3% 33.1%
Phase 2 0.94 0.2682 0.94 [0.42; 1.47] 52.9% 37.6%
Phase 3 0.29 0.4500 0.29 [−0.59; 1.17] 18.8% 29.3%
Fixed effect model 1.09 [0.71; 1.47] 100% −−
Random effects model 1.06 [0.25; 1.88] −− 100%
Heterogeneity: I−squared=75.6%, p=0.0166
−2
−1
0
1
2
% DNA methylation difference

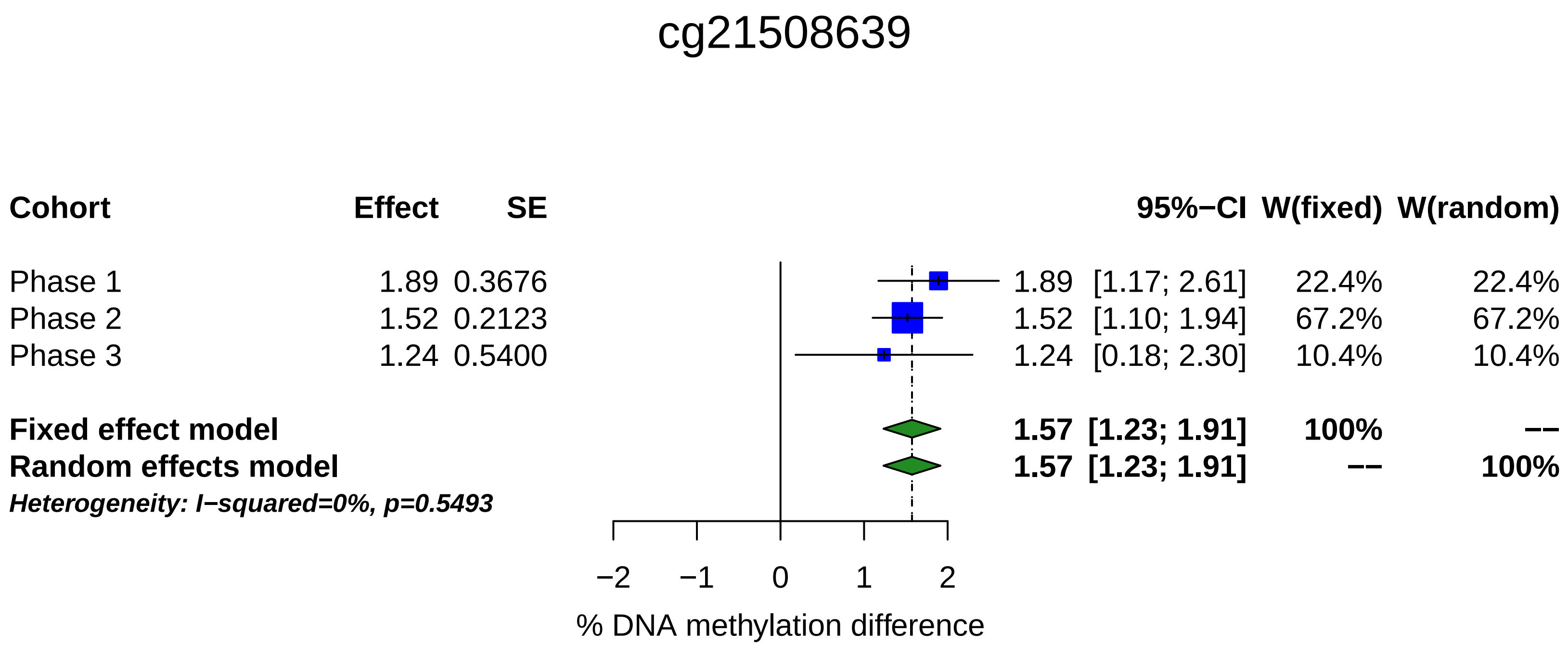
cg21508639
Cohort
Effect
SE
95%−CI
W(fixed)
W(random)
Phase 1 1.89 0.3676 1.89 [1.17; 2.61] 22.4% 22.4%
Phase 2 1.52 0.2123 1.52 [1.10; 1.94] 67.2% 67.2%
Phase 3 1.24 0.5400 1.24 [0.18; 2.30] 10.4% 10.4%
Fixed effect model 1.57 [1.23; 1.91] 100% −−
Random effects model 1.57 [1.23; 1.91] −− 100%
Heterogeneity: I−squared=0%, p=0.5493
−2
−1
0
1
2
% DNA methylation difference

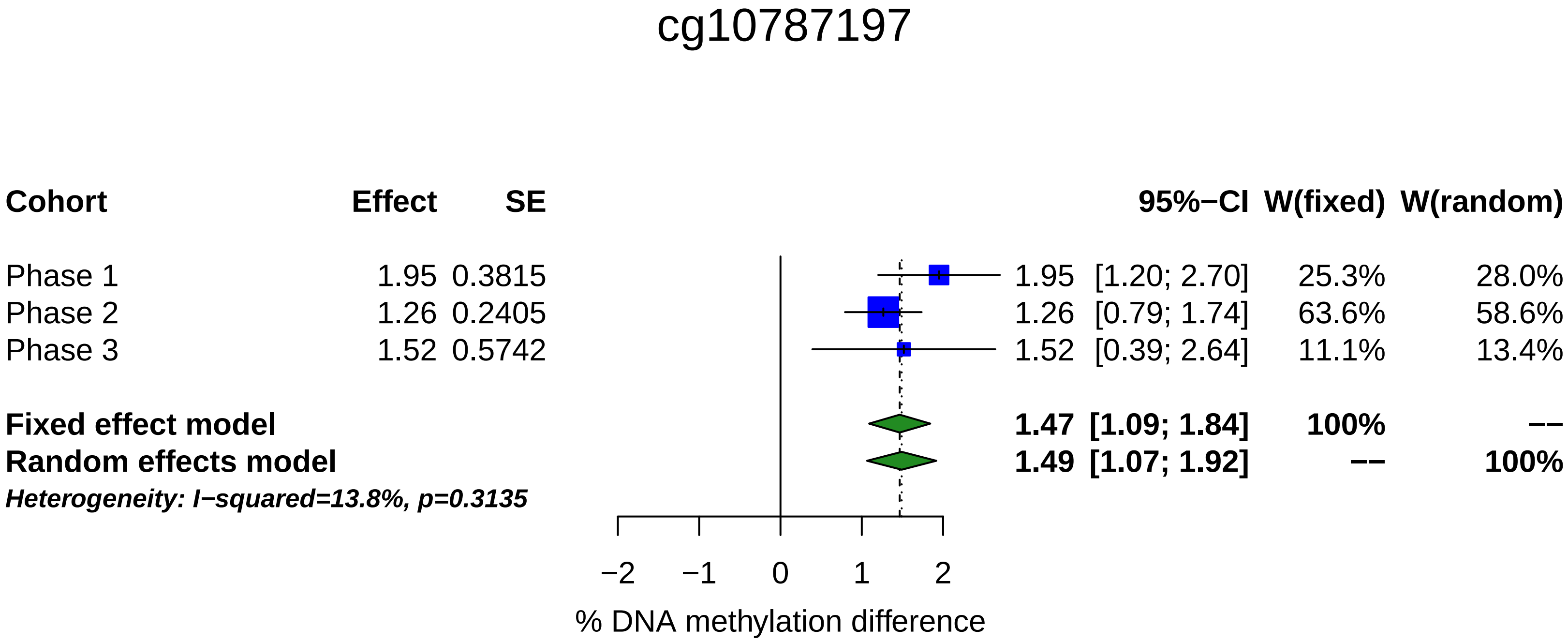
cg10787197
Cohort
Effect
SE
95%−CI
W(fixed)
W(random)
Phase 1 1.95 0.3815 1.95 [1.20; 2.70] 25.3% 28.0%
Phase 2 1.26 0.2405 1.26 [0.79; 1.74] 63.6% 58.6%
Phase 3 1.52 0.5742 1.52 [0.39; 2.64] 11.1% 13.4%
Fixed effect model 1.47 [1.09; 1.84] 100% −−
Random effects model 1.49 [1.07; 1.92] −− 100%
Heterogeneity: I−squared=13.8%, p=0.3135
−2
−1
0
1
2
% DNA methylation difference

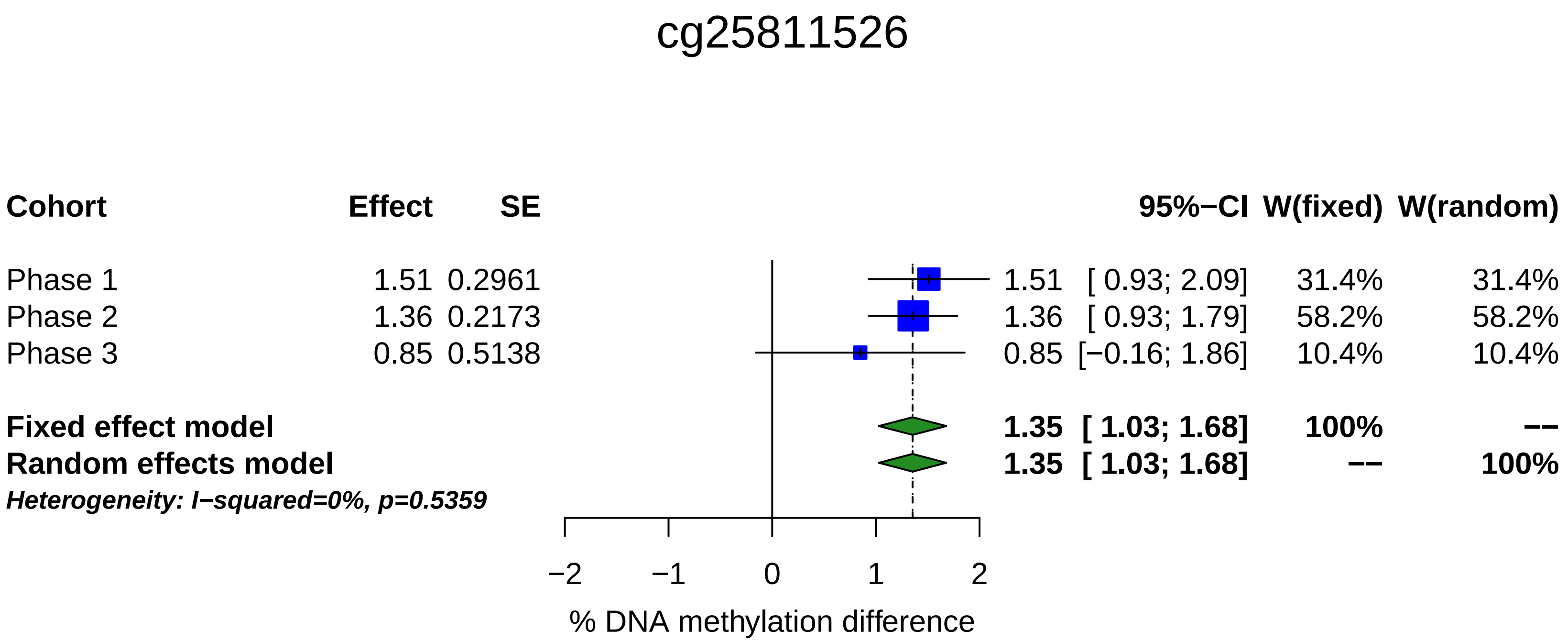
cg25811526
Cohort
Effect
SE
95%−CI
W(fixed)
W(random)
Phase 1
1.51
0.2961
1.51 [0.93; 2.09]
31.4%
31.4%
Phase 2
1.36
0.2173
1.36 [0.93; 1.79]
58.2%
58.2%
Phase 3
0.85
0.5138
0.85 [−0.16; 1.86]
10.4%
10.4%
Fixed effect model
1.35 [1.03; 1.68]
100%
−−
Random effects model
1.35 [1.03; 1.68]
−−
100%
Heterogeneity: I−squared=0%, p=0.5359
−2
−1
0
1
2
% DNA methylation difference

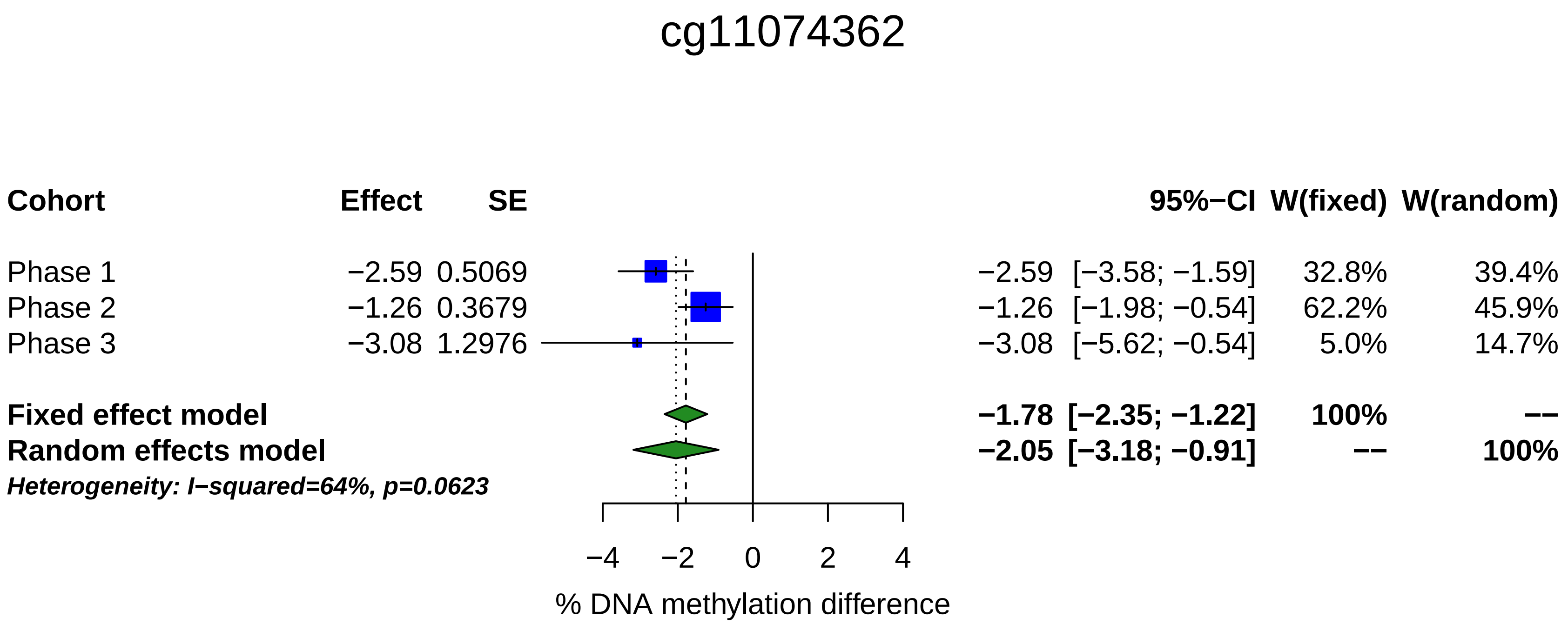
cg11074362
Cohort
Effect
SE
95%−CI
W(fixed)
W(random)
Phase 1 −2.59 0.5069 −2.59 [−3.58; −1.59] 32.8% 39.4%
Phase 2 −1.26 0.3679 −1.26 [−1.98; −0.54] 62.2% 45.9%
Phase 3 −3.08 1.2976 −3.08 [−5.62; −0.54] 5.0% 14.7%
Fixed effect model −1.78 [−2.35; −1.22] 100% −−
Random effects model −2.05 [−3.18; −0.91] −− 100%
Heterogeneity: I−squared=64%, p=0.0623
−4
−2
0
2
4
% DNA methylation difference

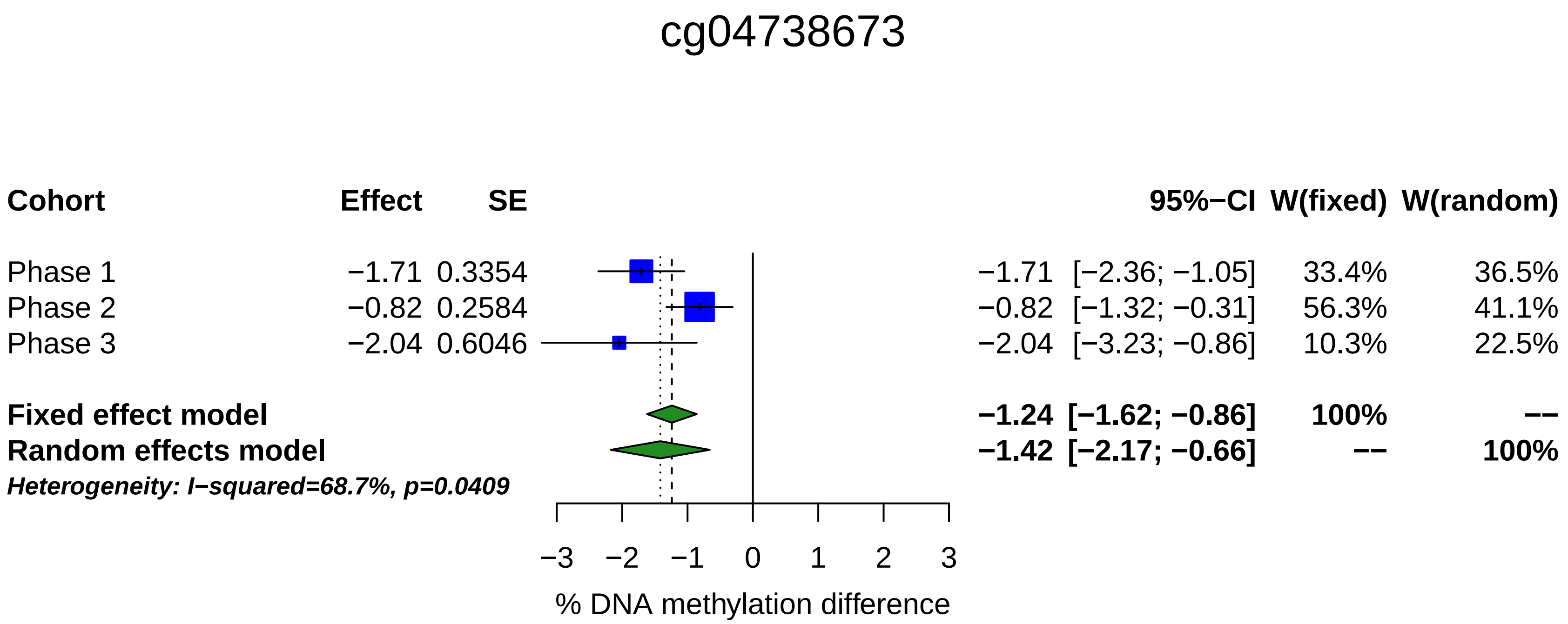
cg04738673
Cohort
Effect
SE
95%−CI
W(fixed)
W(random)
Phase 1 −1.71 0.3354 −1.71 [−2.36; −1.05] 33.4% 36.5%
Phase 2 −0.82 0.2584 −0.82 [−1.32; −0.31] 56.3% 41.1%
Phase 3 −2.04 0.6046 −2.04 [−3.23; −0.86] 10.3% 22.5%
Fixed effect model −1.24 [−1.62; −0.86] 100% −−
Random effects model −1.42 [−2.17; −0.66] −− 100%
Heterogeneity: I−squared=68.7%, p=0.0409
−3
−2
−1
0
1
2
3
% DNA methylation difference

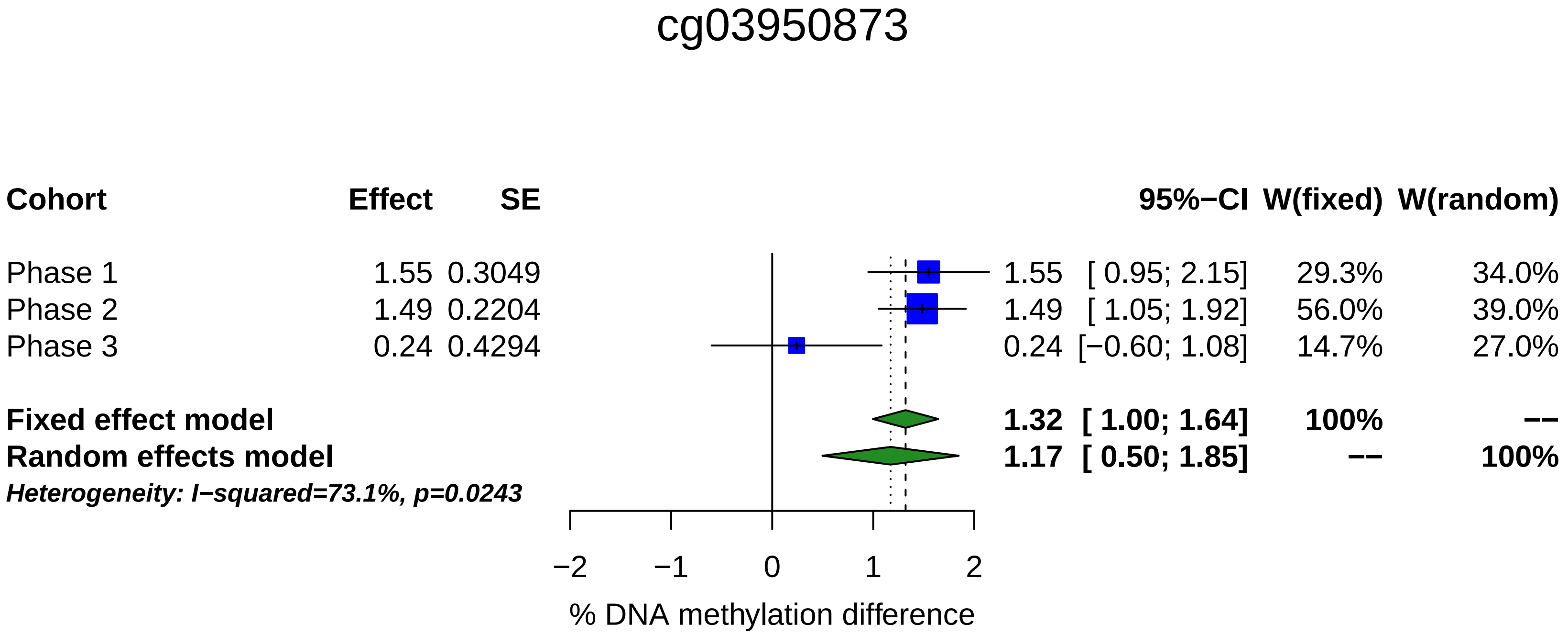
cg03950873
Cohort
Effect
SE
95%−CI
W(fixed)
W(random)
Phase 1 1.55 0.3049 1.55 [0.95; 2.15] 29.3% 34.0%
Phase 2 1.49 0.2204 1.49 [1.05; 1.92] 56.0% 39.0%
Phase 3 0.24 0.4294 0.24 [−0.60; 1.08] 14.7% 27.0%
Fixed effect model 1.32 [1.00; 1.64] 100% −−
Random effects model 1.17 [0.50; 1.85] −− 100%
Heterogeneity: I−squared=73.1%, p=0.0243
−2
−1
0
1
2
% DNA methylation difference

cg14130119

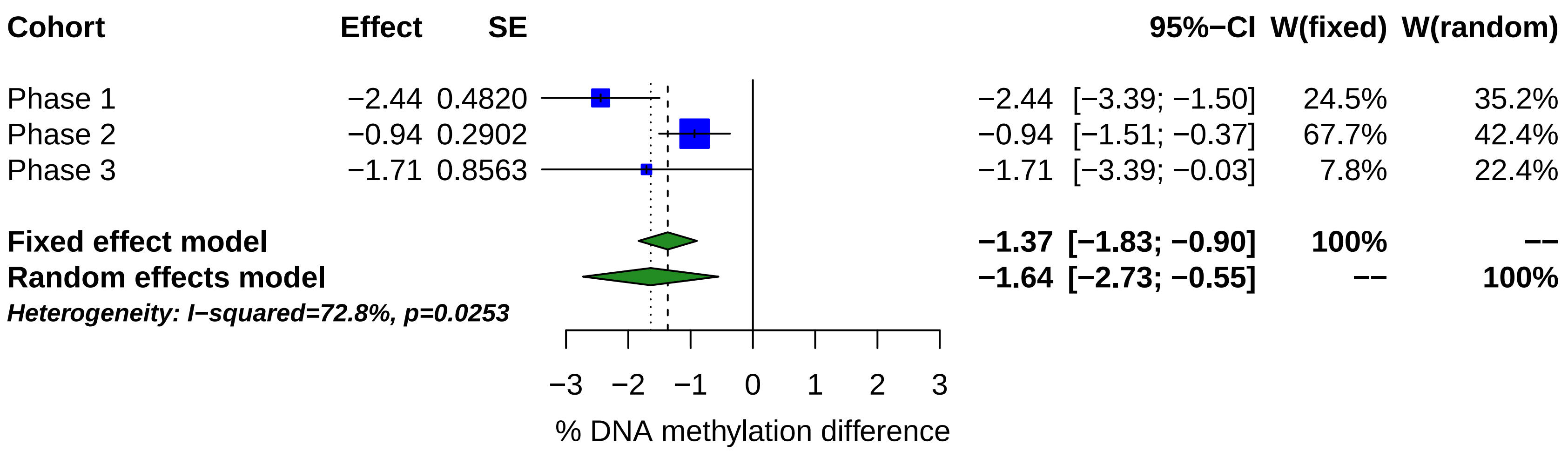

Cohort
Effect
SE
95%−CI
W(fixed)
W(random)
Phase 1 −2.44 0.4820 −2.44 [−3.39; −1.50] 24.5% 35.2%
Phase 2 −0.94 0.2902 −0.94 [−1.51; −0.37] 67.7% 42.4%
Phase 3 −1.71 0.8563 −1.71 [−3.39; −0.03] 7.8% 22.4%
Fixed effect model −1.37 [−1.83; −0.90] 100% −−
Random effects model −1.64 [−2.73; −0.55] −− 100%
Heterogeneity: I−squared=72.8%, p=0.0253
−3 −2 −1 0 1 2 3
% DNA methylation difference

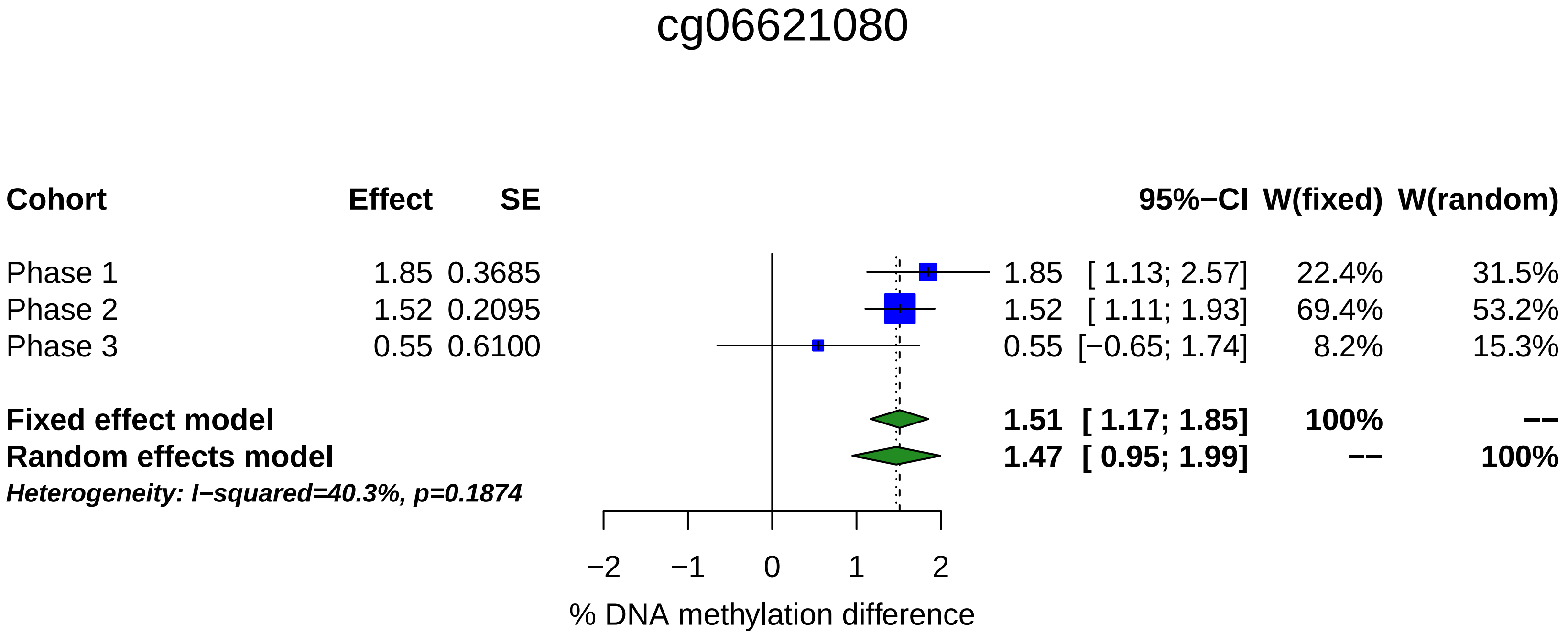
cg06621080
Cohort
Effect
SE
95%−CI
W(fixed)
W(random)
Phase 1 1.85 0.3685 1.85 [1.13; 2.57] 22.4% 31.5%
Phase 2 1.52 0.2095 1.52 [1.11; 1.93] 69.4% 53.2%
Phase 3 0.55 0.6100 0.55 [−0.65; 1.74] 8.2% 15.3%
Fixed effect model 1.51 [1.17; 1.85] 100% −−
Random effects model 1.47 [0.95; 1.99] −− 100%
Heterogeneity: I−squared=40.3%, p=0.1874
−2
−1
0
1
2
% DNA methylation difference

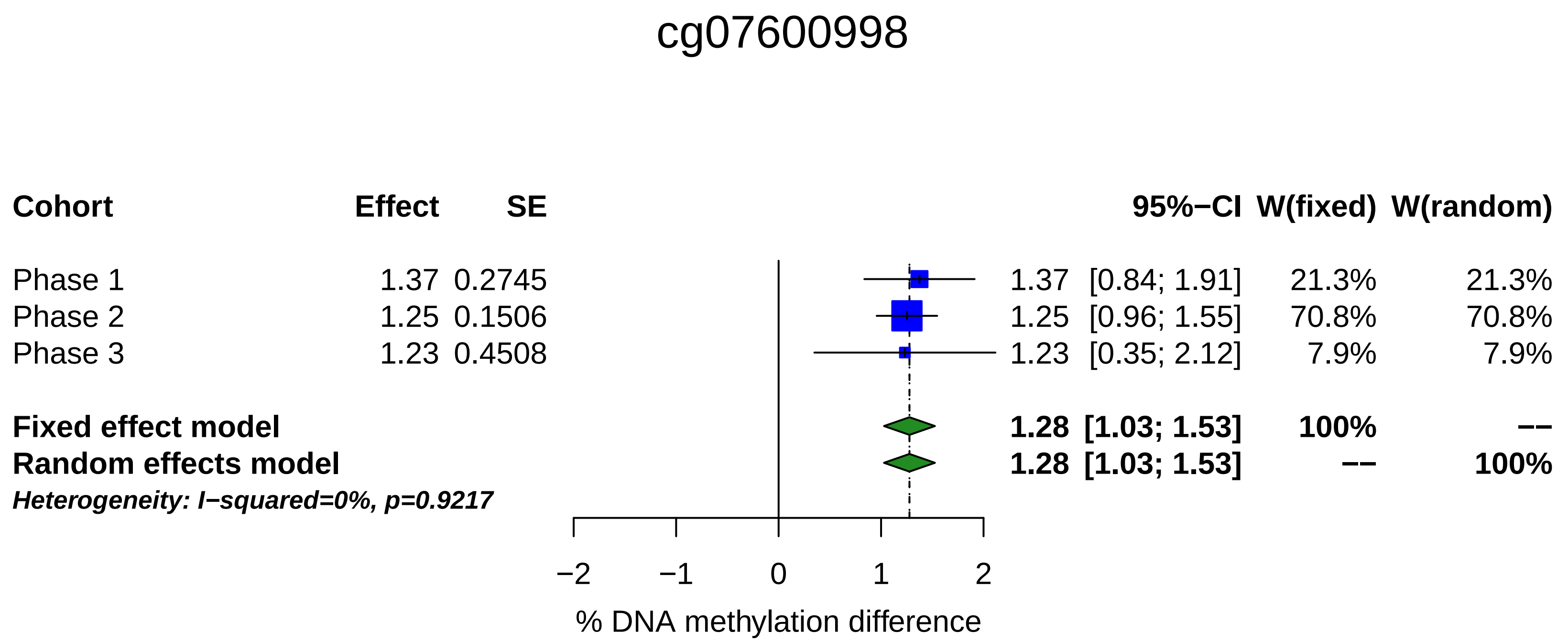
cg07600998
Cohort
Effect
SE
95%−CI
W(fixed)
W(random)
Phase 1 1.37 0.2745 1.37 [0.84; 1.91] 21.3% 21.3%
Phase 2 1.25 0.1506 1.25 [0.96; 1.55] 70.8% 70.8%
Phase 3 1.23 0.4508 1.23 [0.35; 2.12] 7.9% 7.9%
Fixed effect model 1.28 [1.03; 1.53] 100% −−
Random effects model 1.28 [1.03; 1.53] −− 100%
Heterogeneity: I−squared=0%, p=0.9217
−2
−1
0
1
2
% DNA methylation difference

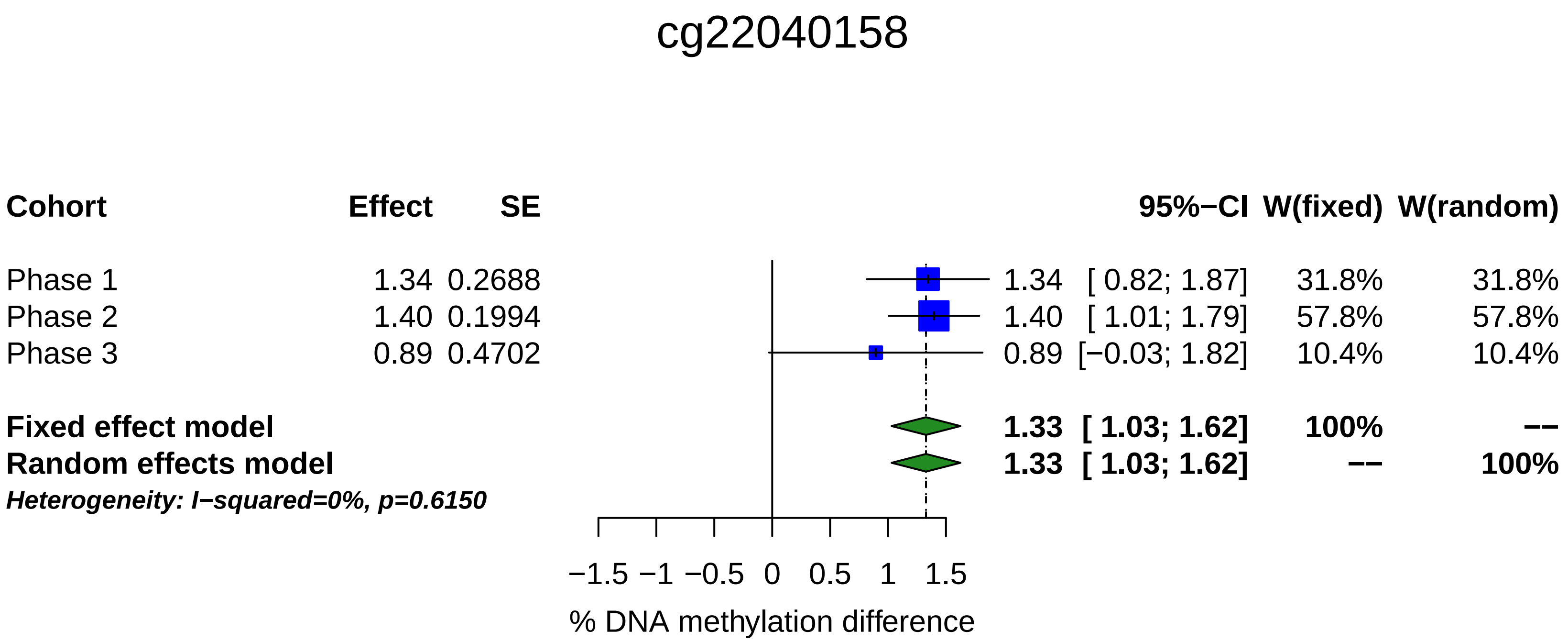
cg22040158
Cohort
Effect
SE
95%−CI
W(fixed)
W(random)
Phase 1 1.34 0.2688 1.34 [0.82; 1.87] 31.8% 31.8%
Phase 2 1.40 0.1994 1.40 [1.01; 1.79] 57.8% 57.8%
Phase 3 0.89 0.4702 0.89 [−0.03; 1.82] 10.4% 10.4%
Fixed effect model 1.33 [1.03; 1.62] 100% −−
Random effects model 1.33 [1.03; 1.62] −− 100%
Heterogeneity: I−squared=0%, p=0.6150
−1.5
−1
−0.5
0
0.5
1
1.5
% DNA methylation difference

cg12044923

Cohort	Effect	SE	95%−CI	W(fixed)	W(random)
Phase 1	1.33	0.2674	1.33 [0.80; 1.85]	30.2%	34.3%
Phase 2	1.53	0.1975	1.53 [1.15; 1.92]	55.4%	41.7%
Phase 3	0.56	0.3888	0.56 [−0.20; 1.32]	14.3%	24.0%
**Fixed effect model**			**1.33 [1.04; 1.62]**	**100%**	**−−**
**Random effects model**			**1.23 [0.74; 1.72]**	**−−**	**100%**

Heterogeneity: I−squared=60.1%, p=0.0817

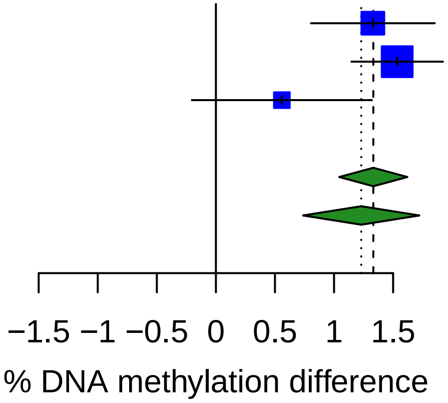

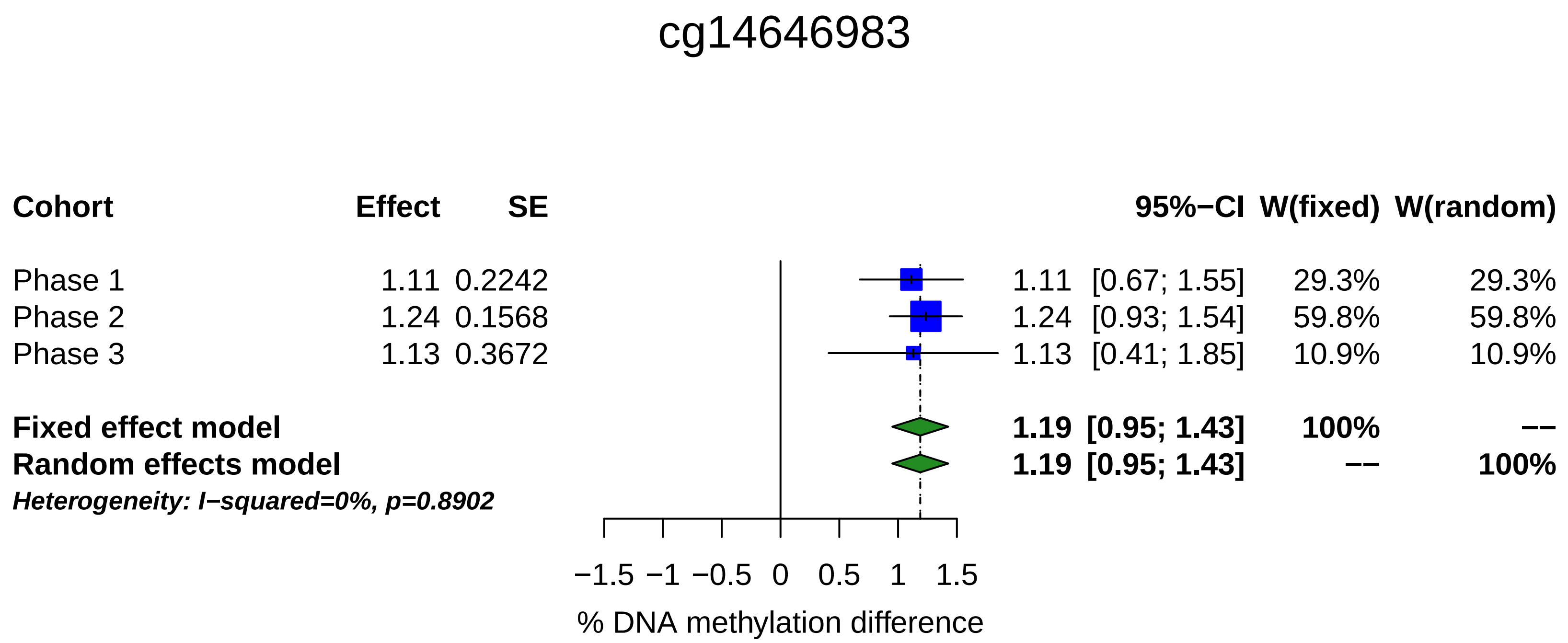
cg14646983
Cohort
Effect
SE
95%−CI
W(fixed)
W(random)
Phase 1 1.11 0.2242 1.11 [0.67; 1.55] 29.3% 29.3%
Phase 2 1.24 0.1568 1.24 [0.93; 1.54] 59.8% 59.8%
Phase 3 1.13 0.3672 1.13 [0.41; 1.85] 10.9% 10.9%
Fixed effect model 1.19 [0.95; 1.43] 100% −−
Random effects model 1.19 [0.95; 1.43] −− 100%
Heterogeneity: I−squared=0%, p=0.8902
−1.5 −1 −0.5 0 0.5 1 1.5
% DNA methylation difference

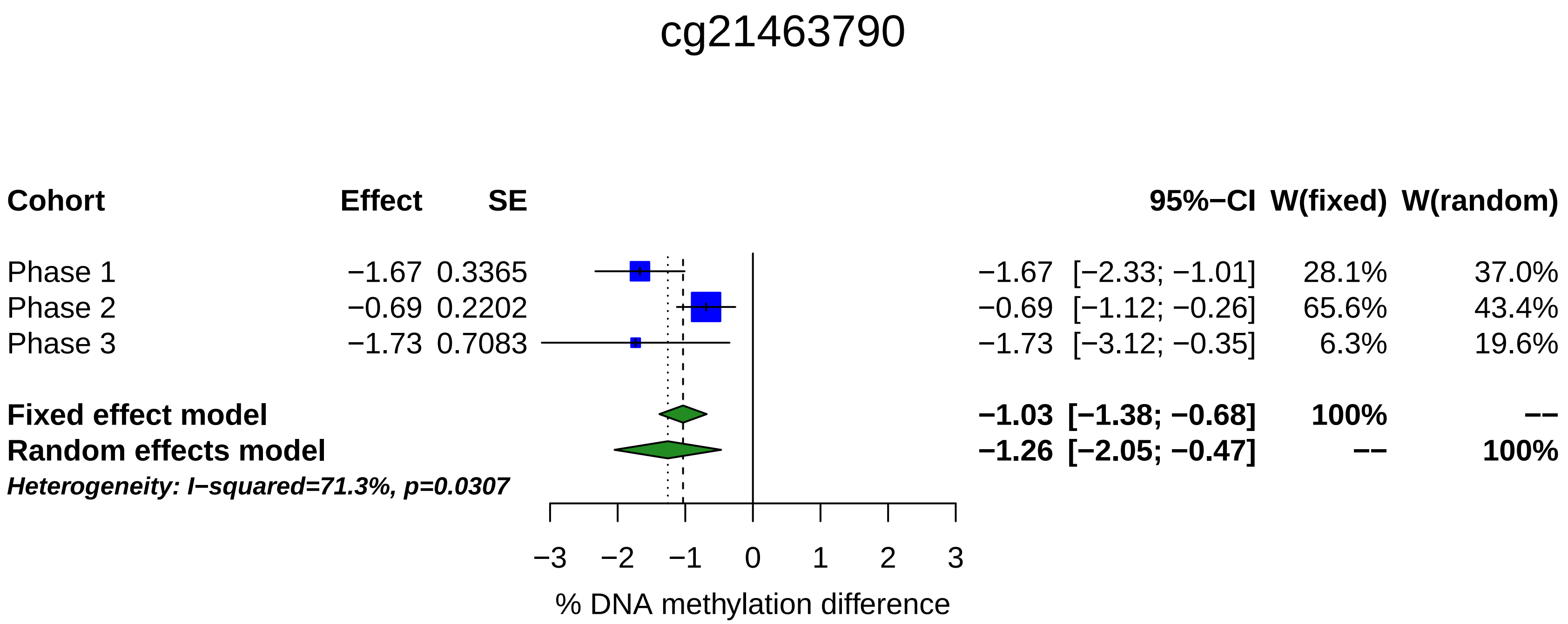
cg21463790
Cohort
Effect
SE
95%−CI
W(fixed)
W(random)
Phase 1 −1.67 0.3365 −1.67 [−2.33; −1.01] 28.1% 37.0%
Phase 2 −0.69 0.2202 −0.69 [−1.12; −0.26] 65.6% 43.4%
Phase 3 −1.73 0.7083 −1.73 [−3.12; −0.35] 6.3% 19.6%
Fixed effect model −1.03 [−1.38; −0.68] 100% −−
Random effects model −1.26 [−2.05; −0.47] −− 100%
Heterogeneity: I−squared=71.3%, p=0.0307
−3
−2
−1
0
1
2
3
% DNA methylation difference

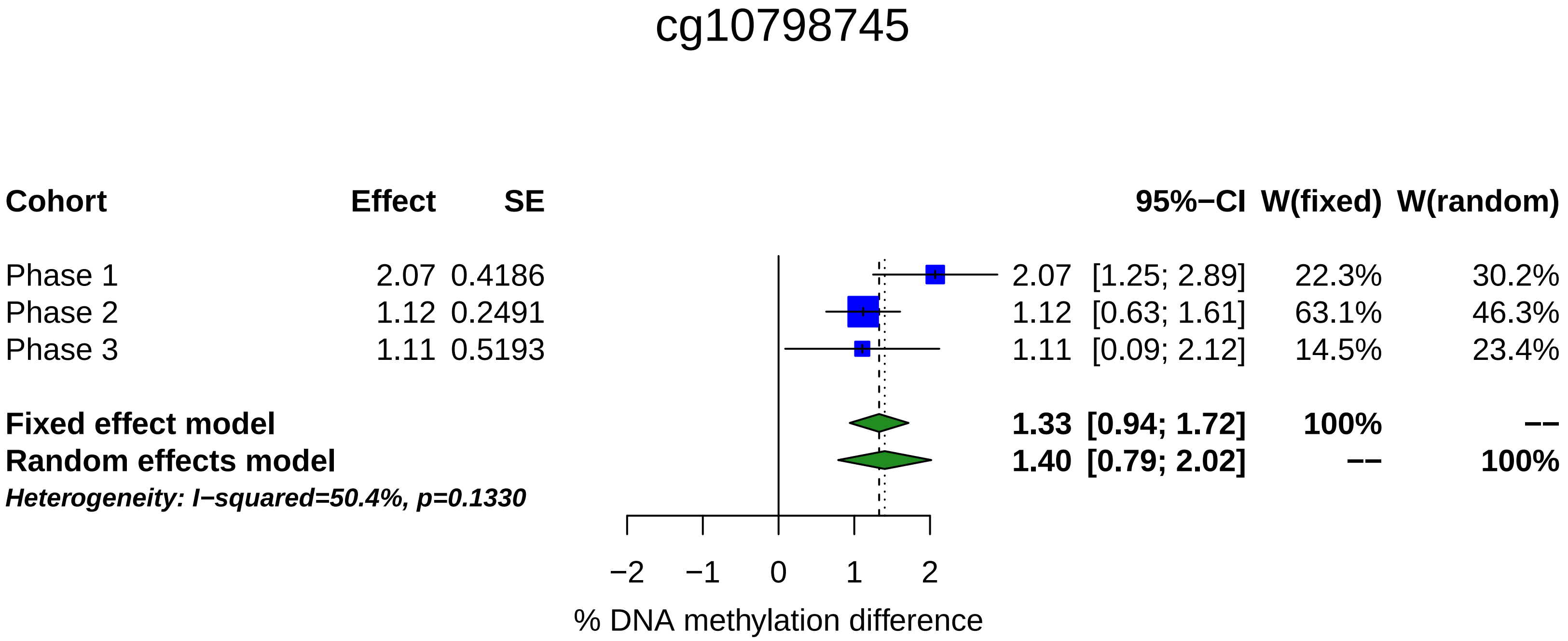
cg10798745
Cohort
Effect
SE
95%−CI
W(fixed)
W(random)
Phase 1 2.07 0.4186 2.07 [1.25; 2.89] 22.3% 30.2%
Phase 2 1.12 0.2491 1.12 [0.63; 1.61] 63.1% 46.3%
Phase 3 1.11 0.5193 1.11 [0.09; 2.12] 14.5% 23.4%
Fixed effect model 1.33 [0.94; 1.72] 100% −−
Random effects model 1.40 [0.79; 2.02] −− 100%
Heterogeneity: I−squared=50.4%, p=0.1330
−2
−1
0
1
2
% DNA methylation difference

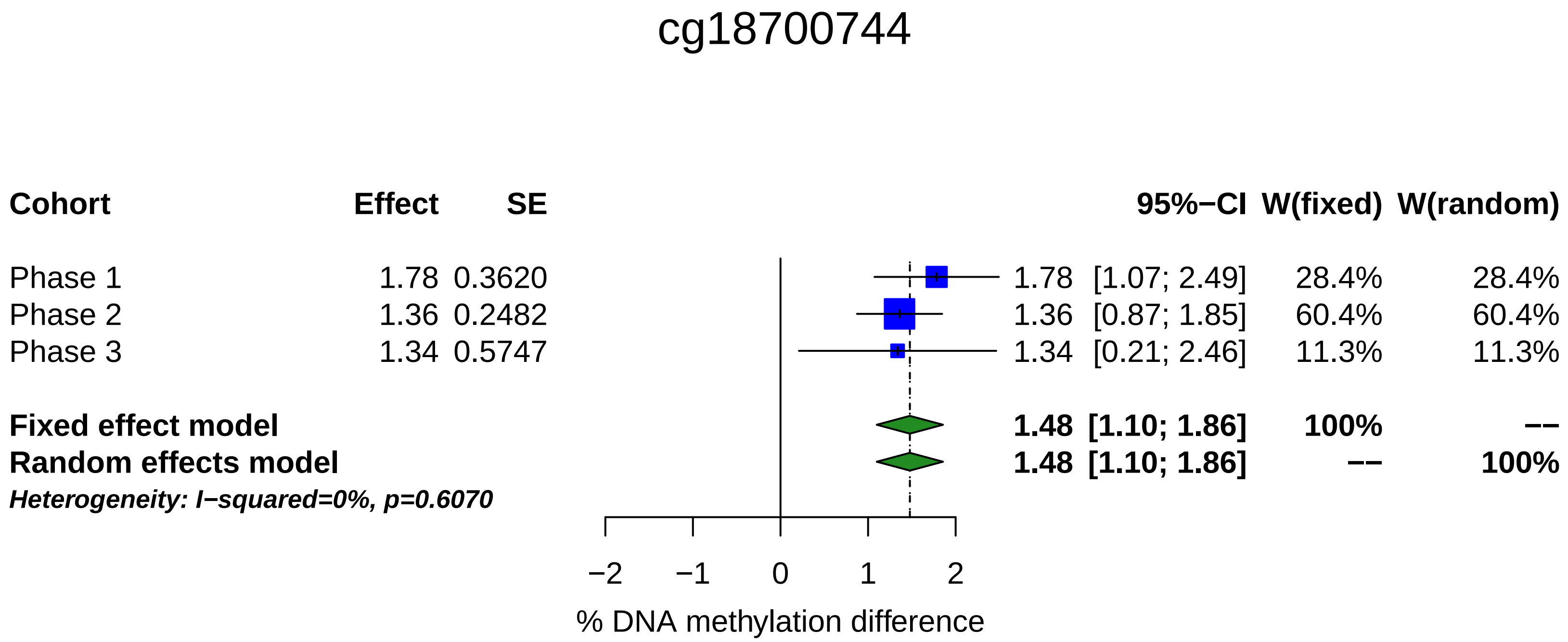
cg18700744
Cohort
Effect
SE
95%−CI
W(fixed)
W(random)
Phase 1 1.78 0.3620 1.78 [1.07; 2.49] 28.4% 28.4%
Phase 2 1.36 0.2482 1.36 [0.87; 1.85] 60.4% 60.4%
Phase 3 1.34 0.5747 1.34 [0.21; 2.46] 11.3% 11.3%
Fixed effect model 1.48 [1.10; 1.86] 100% −−
Random effects model 1.48 [1.10; 1.86] −− 100%
Heterogeneity: I−squared=0%, p=0.6070
−2
−1
0
1
2
% DNA methylation difference

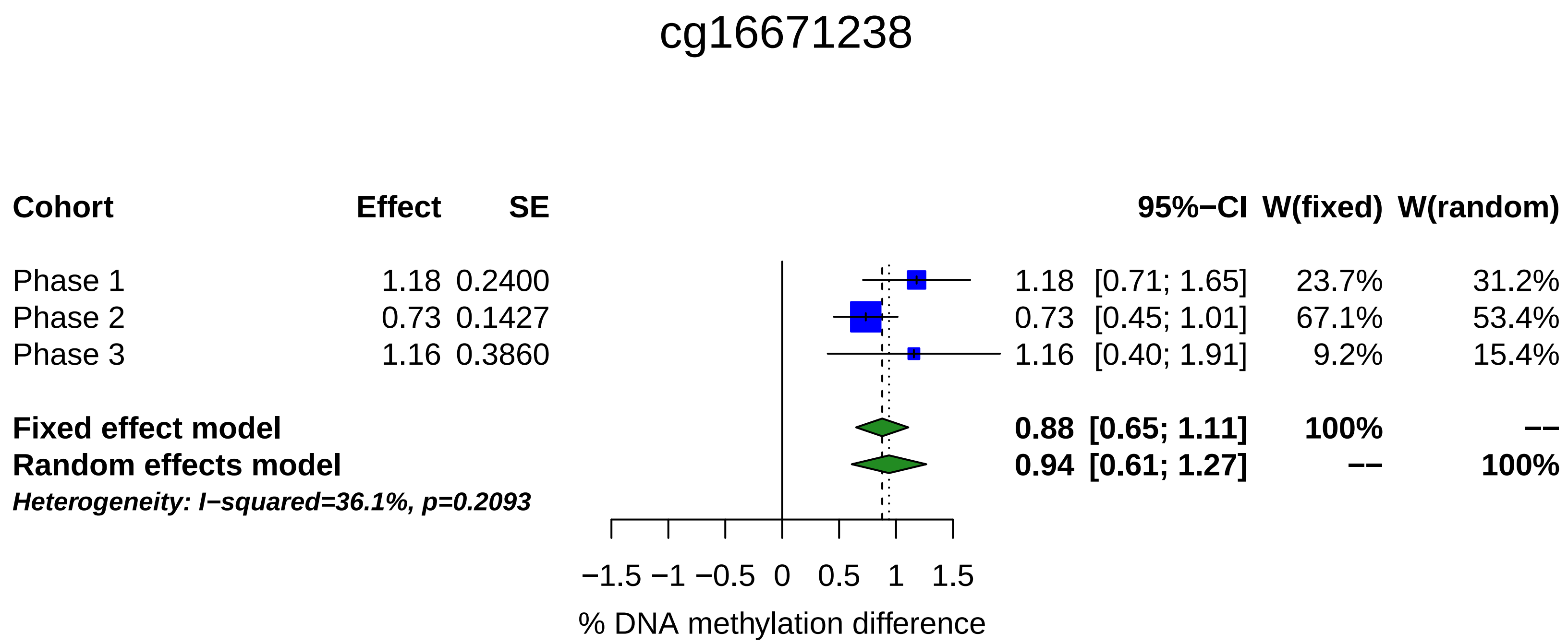
cg16671238
Cohort
Effect
SE
95%−CI
W(fixed)
W(random)
Phase 1
1.18
0.2400
1.18 [0.71; 1.65]
23.7%
31.2%
Phase 2
0.73
0.1427
0.73 [0.45; 1.01]
67.1%
53.4%
Phase 3
1.16
0.3860
1.16 [0.40; 1.91]
9.2%
15.4%
Fixed effect model
0.88 [0.65; 1.11]
100%
−−
Random effects model
0.94 [0.61; 1.27]
−−
100%
Heterogeneity: I−squared=36.1%, p=0.2093
−1.5
−1
−0.5
0
0.5
1
1.5
% DNA methylation difference

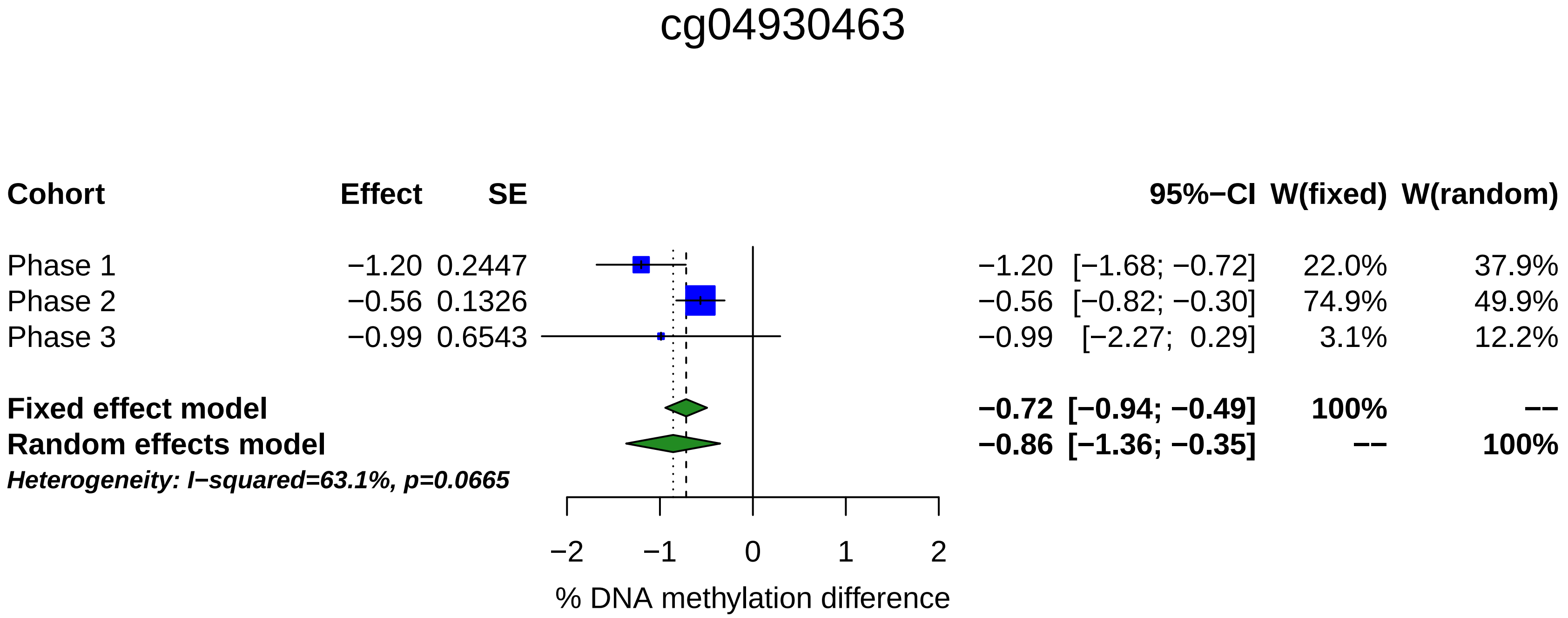
cg04930463
Cohort
Effect
SE
95%−CI
W(fixed)
W(random)
Phase 1 −1.20 0.2447 −1.20 [−1.68; −0.72] 22.0% 37.9%
Phase 2 −0.56 0.1326 −0.56 [−0.82; −0.30] 74.9% 49.9%
Phase 3 −0.99 0.6543 −0.99 [−2.27; 0.29] 3.1% 12.2%
Fixed effect model −0.72 [−0.94; −0.49] 100% −−
Random effects model −0.86 [−1.36; −0.35] −− 100%
Heterogeneity: I−squared=63.1%, p=0.0665
−2
−1
0
1
2
% DNA methylation difference

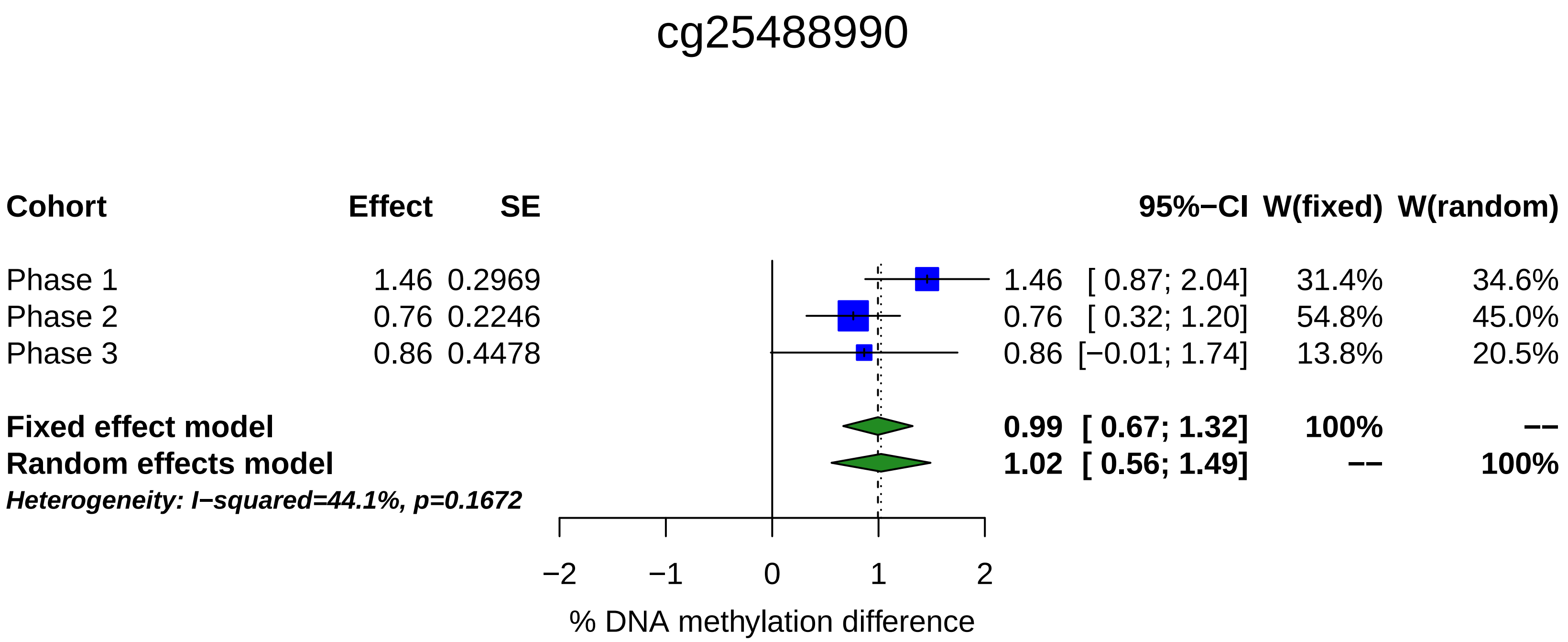
cg25488990
Cohort
Effect
SE
95%−CI
W(fixed)
W(random)
Phase 1 1.46 0.2969 1.46 [0.87; 2.04] 31.4% 34.6%
Phase 2 0.76 0.2246 0.76 [0.32; 1.20] 54.8% 45.0%
Phase 3 0.86 0.4478 0.86 [−0.01; 1.74] 13.8% 20.5%
Fixed effect model 0.99 [0.67; 1.32] 100% −−
Random effects model 1.02 [0.56; 1.49] −− 100%
Heterogeneity: I−squared=44.1%, p=0.1672
−2
−1
0
1
2
% DNA methylation difference

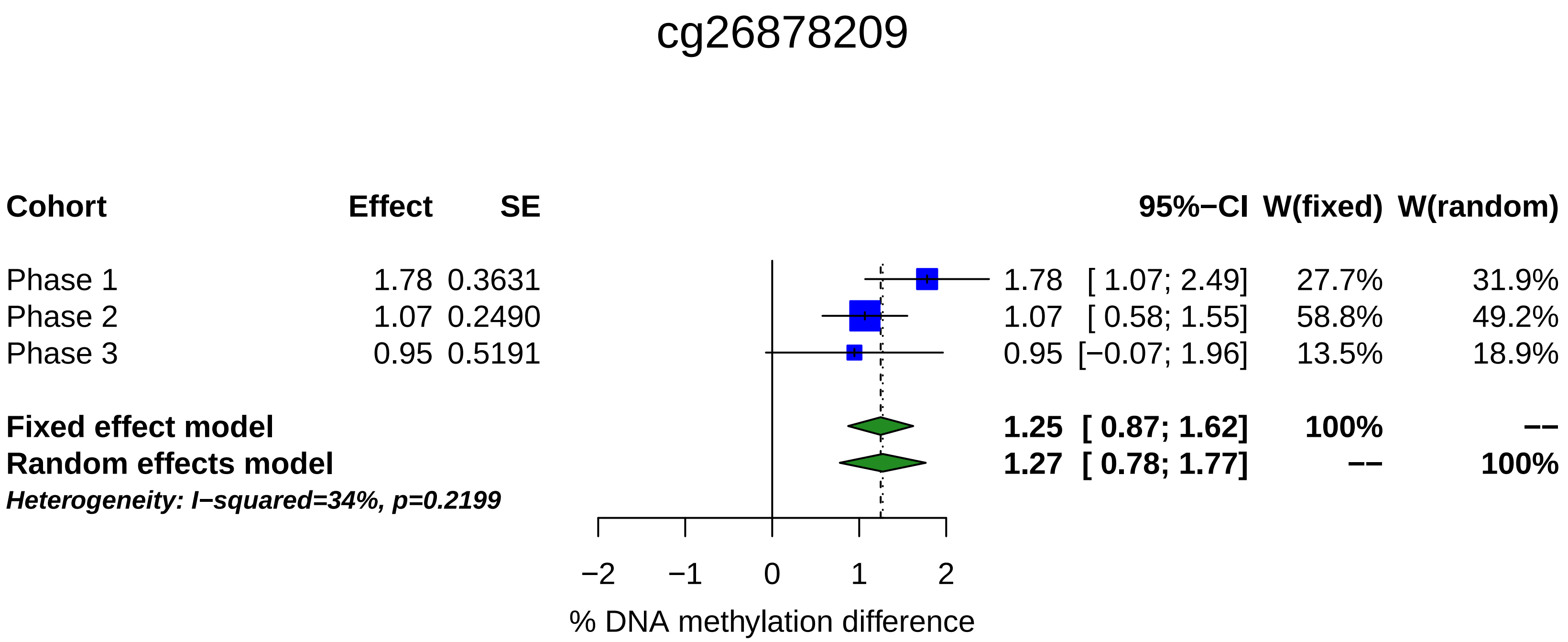
cg26878209
Cohort
Effect
SE
95%−CI
W(fixed)
W(random)
Phase 1 1.78 0.3631 1.78 [1.07; 2.49] 27.7% 31.9%
Phase 2 1.07 0.2490 1.07 [0.58; 1.55] 58.8% 49.2%
Phase 3 0.95 0.5191 0.95 [−0.07; 1.96] 13.5% 18.9%
Fixed effect model 1.25 [0.87; 1.62] 100% −−
Random effects model 1.27 [0.78; 1.77] −− 100%
Heterogeneity: I−squared=34%, p=0.2199
−2
−1
0
1
2
% DNA methylation difference

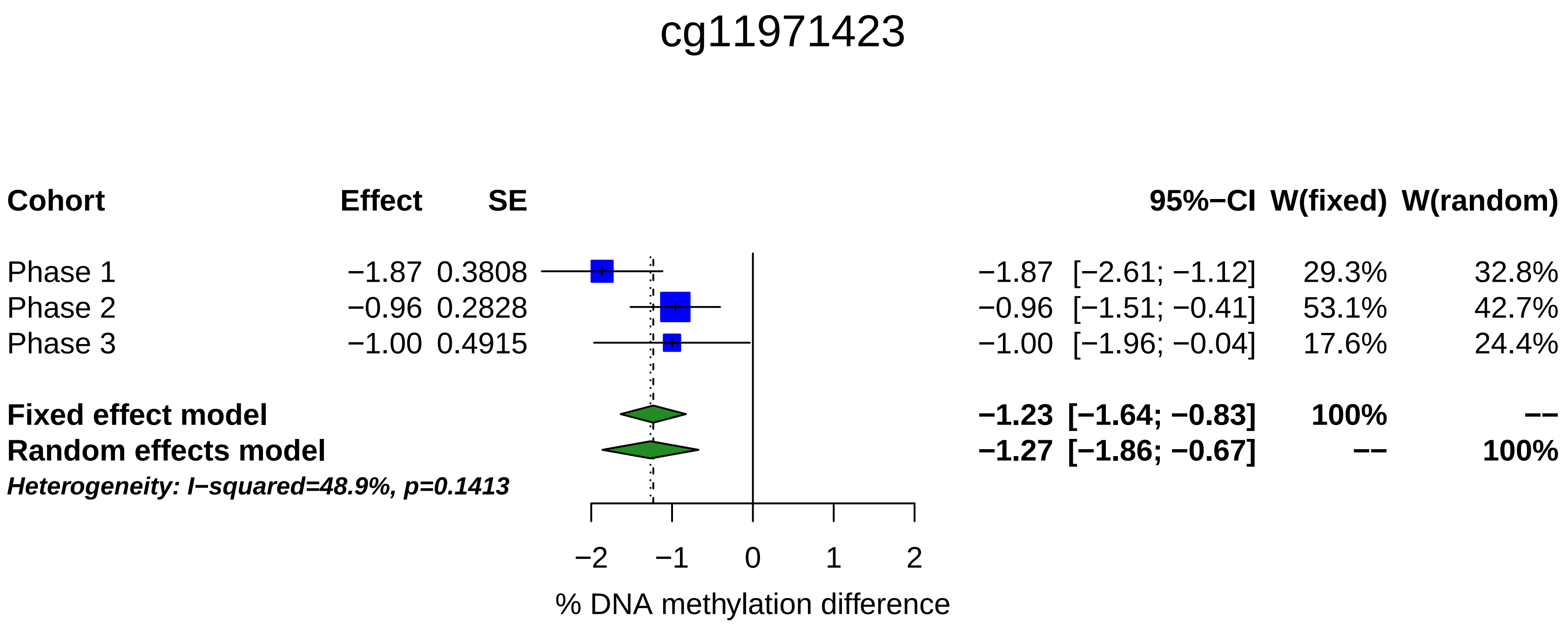
cg11971423
Cohort
Effect
SE
95%−CI
W(fixed)
W(random)
Phase 1 −1.87 0.3808 −1.87 [−2.61; −1.12] 29.3% 32.8%
Phase 2 −0.96 0.2828 −0.96 [−1.51; −0.41] 53.1% 42.7%
Phase 3 −1.00 0.4915 −1.00 [−1.96; −0.04] 17.6% 24.4%
Fixed effect model −1.23 [−1.64; −0.83] 100% −−
Random effects model −1.27 [−1.86; −0.67] −− 100%
Heterogeneity: I−squared=48.9%, p=0.1413
−2
−1
0
1
2
% DNA methylation difference

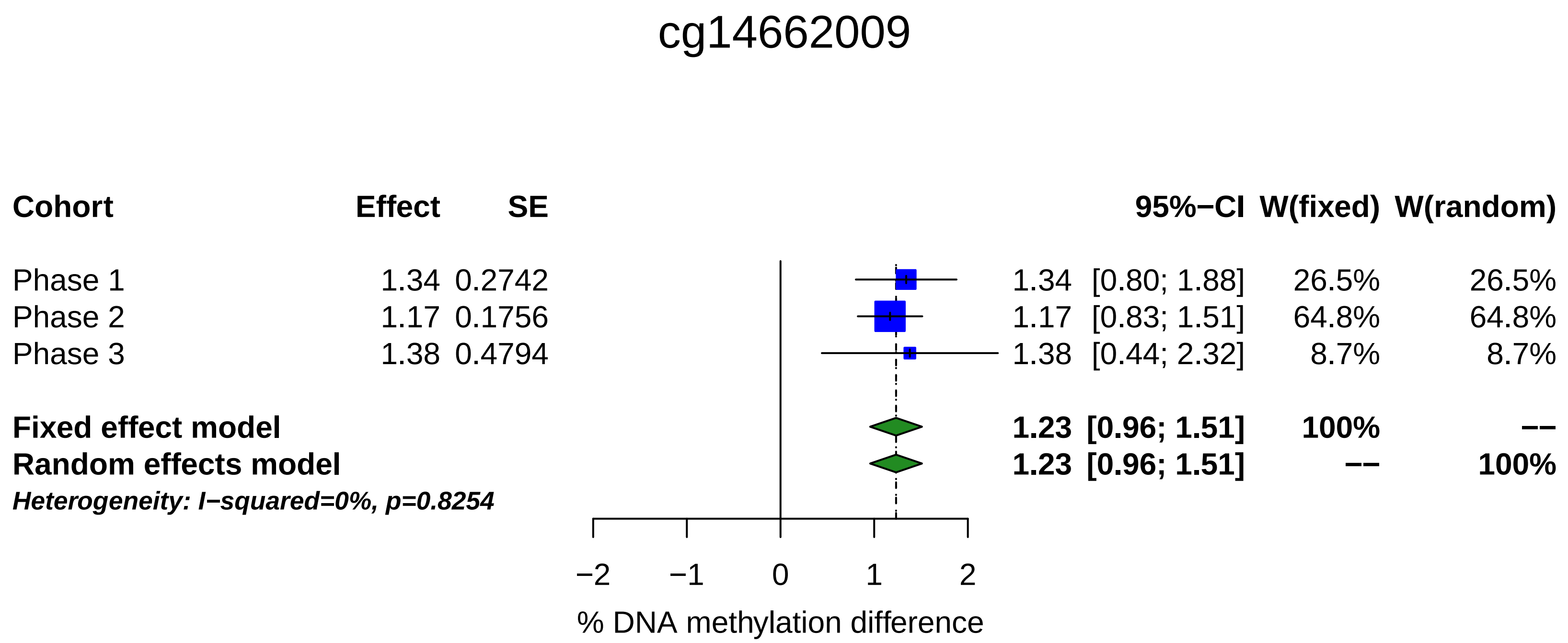
cg14662009
Cohort
Effect
SE
95%−CI
W(fixed)
W(random)
Phase 1 1.34 0.2742 1.34 [0.80; 1.88] 26.5% 26.5%
Phase 2 1.17 0.1756 1.17 [0.83; 1.51] 64.8% 64.8%
Phase 3 1.38 0.4794 1.38 [0.44; 2.32] 8.7% 8.7%
Fixed effect model 1.23 [0.96; 1.51] 100% −−
Random effects model 1.23 [0.96; 1.51] −− 100%
Heterogeneity: I−squared=0%, p=0.8254
−2
−1
0
1
2
% DNA methylation difference

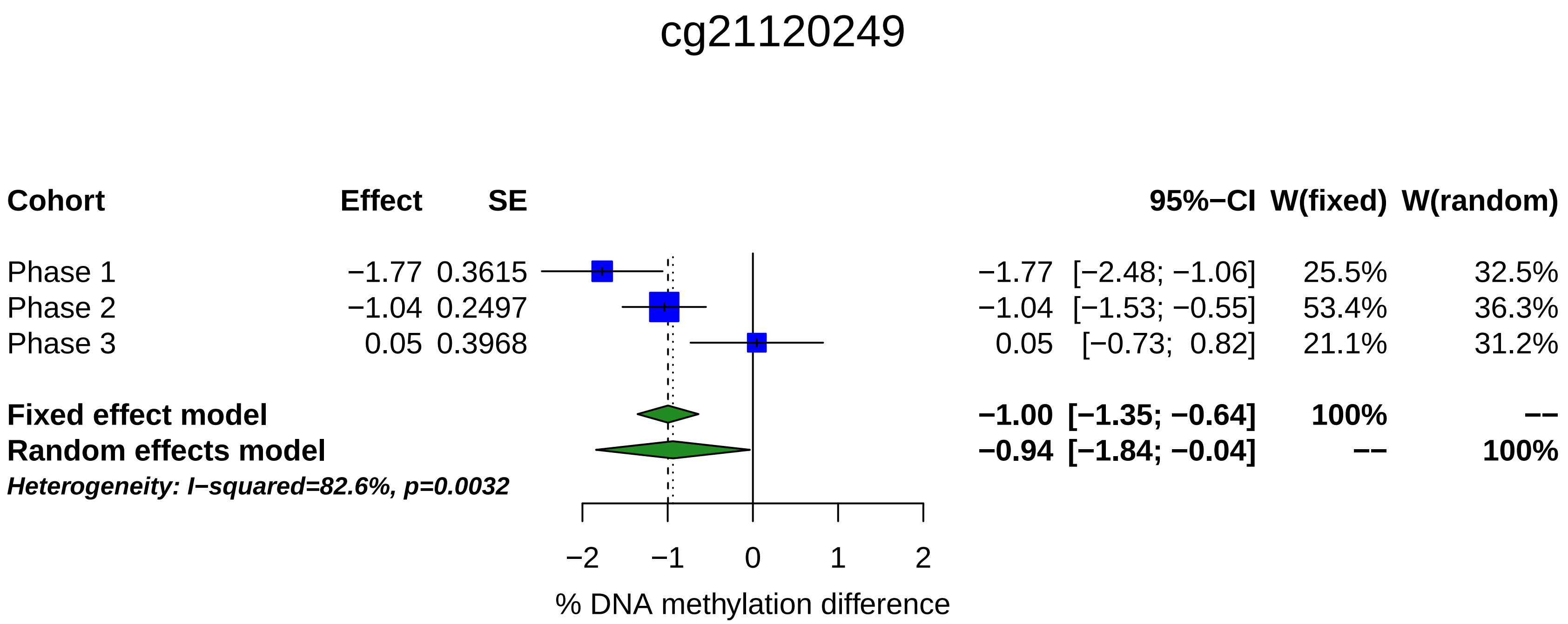
cg21120249
Cohort
Effect
SE
95%−CI
W(fixed)
W(random)
Phase 1 −1.77 0.3615 −1.77 [−2.48; −1.06] 25.5% 32.5%
Phase 2 −1.04 0.2497 −1.04 [−1.53; −0.55] 53.4% 36.3%
Phase 3 0.05 0.3968 0.05 [−0.73; 0.82] 21.1% 31.2%
Fixed effect model −1.00 [−1.35; −0.64] 100% −−
Random effects model −0.94 [−1.84; −0.04] −− 100%
Heterogeneity: I−squared=82.6%, p=0.0032
−2
−1
0
1
2
% DNA methylation difference

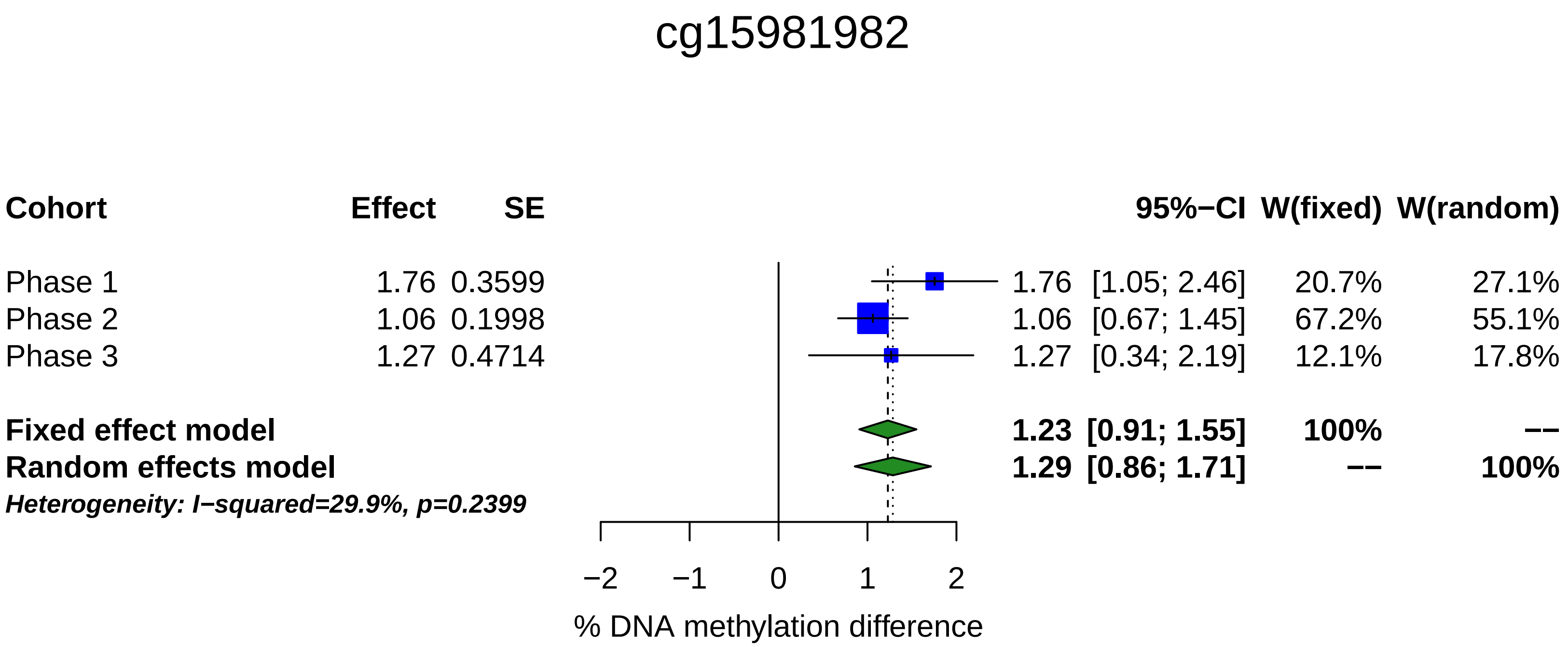
cg15981982
Cohort
Effect
SE
95%−CI
W(fixed)
W(random)
Phase 1
1.76
0.3599
1.76 [1.05; 2.46]
20.7%
27.1%
Phase 2
1.06
0.1998
1.06 [0.67; 1.45]
67.2%
55.1%
Phase 3
1.27
0.4714
1.27 [0.34; 2.19]
12.1%
17.8%
Fixed effect model
1.23 [0.91; 1.55]
100%
−−
Random effects model
1.29 [0.86; 1.71]
−−
100%
Heterogeneity: I−squared=29.9%, p=0.2399
−2
−1
0
1
2
% DNA methylation difference

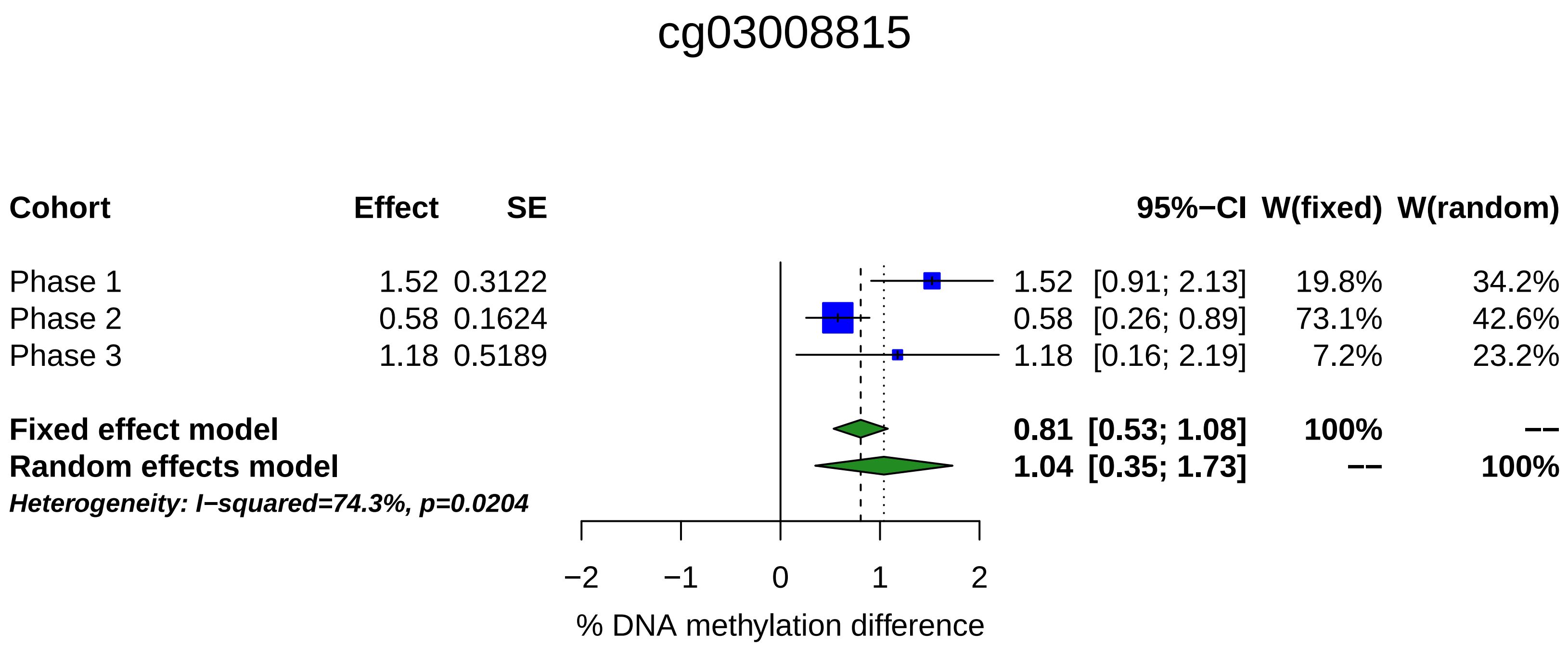
cg03008815
Cohort | Effect | SE | 95%−CI | W(fixed) | W(random)
Phase 1 | 1.52 | 0.3122 | 1.52 [0.91; 2.13] | 19.8% | 34.2%
Phase 2 | 0.58 | 0.1624 | 0.58 [0.26; 0.89] | 73.1% | 42.6%
Phase 3 | 1.18 | 0.5189 | 1.18 [0.16; 2.19] | 7.2% | 23.2%
Fixed effect model | 0.81 [0.53; 1.08] | 100% | −−
Random effects model | 1.04 [0.35; 1.73] | −− | 100%
Heterogeneity: I−squared=74.3%, p=0.0204
−2
−1
0
1
2
% DNA methylation difference

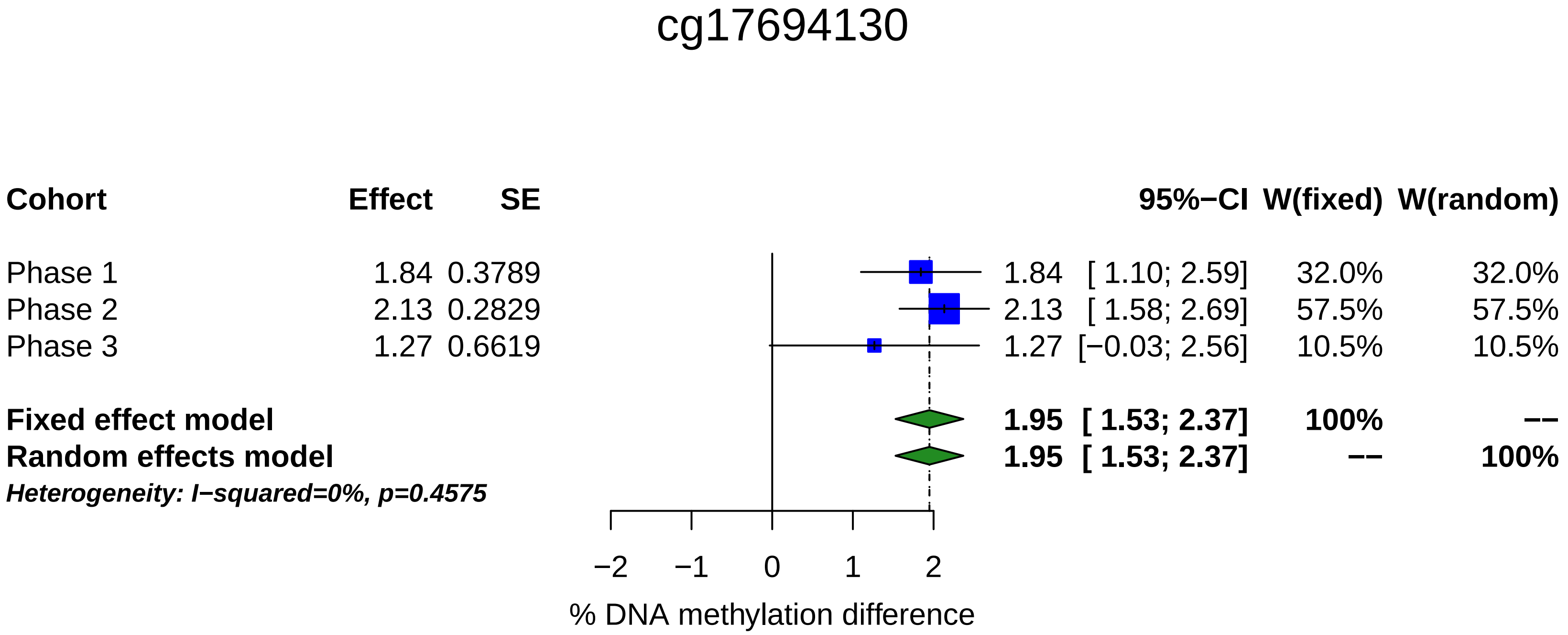
cg17694130
Cohort Effect SE 95%−CI W(fixed) W(random)
Phase 1 1.84 0.3789 1.84 [1.10; 2.59] 32.0% 32.0%
Phase 2 2.13 0.2829 2.13 [1.58; 2.69] 57.5% 57.5%
Phase 3 1.27 0.6619 1.27 [−0.03; 2.56] 10.5% 10.5%
Fixed effect model 1.95 [1.53; 2.37] 100% −−
Random effects model 1.95 [1.53; 2.37] −− 100%
Heterogeneity: I−squared=0%, p=0.4575
−2 −1 0 1 2
% DNA methylation difference

cg24004007

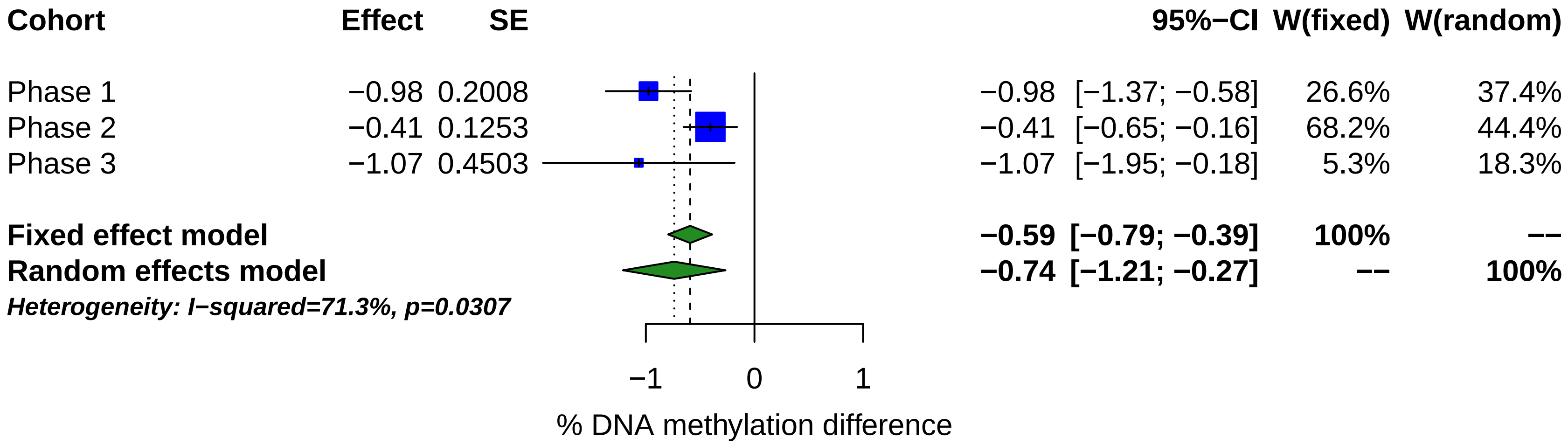

Cohort
Effect
SE
95%−CI
W(fixed)
W(random)
Phase 1 −0.98 0.2008 −0.98 [−1.37; −0.58] 26.6% 37.4%
Phase 2 −0.41 0.1253 −0.41 [−0.65; −0.16] 68.2% 44.4%
Phase 3 −1.07 0.4503 −1.07 [−1.95; −0.18] 5.3% 18.3%
Fixed effect model −0.59 [−0.79; −0.39] 100% −−
Random effects model −0.74 [−1.21; −0.27] −− 100%
Heterogeneity: I−squared=71.3%, p=0.0307
−1
0
1
% DNA methylation difference

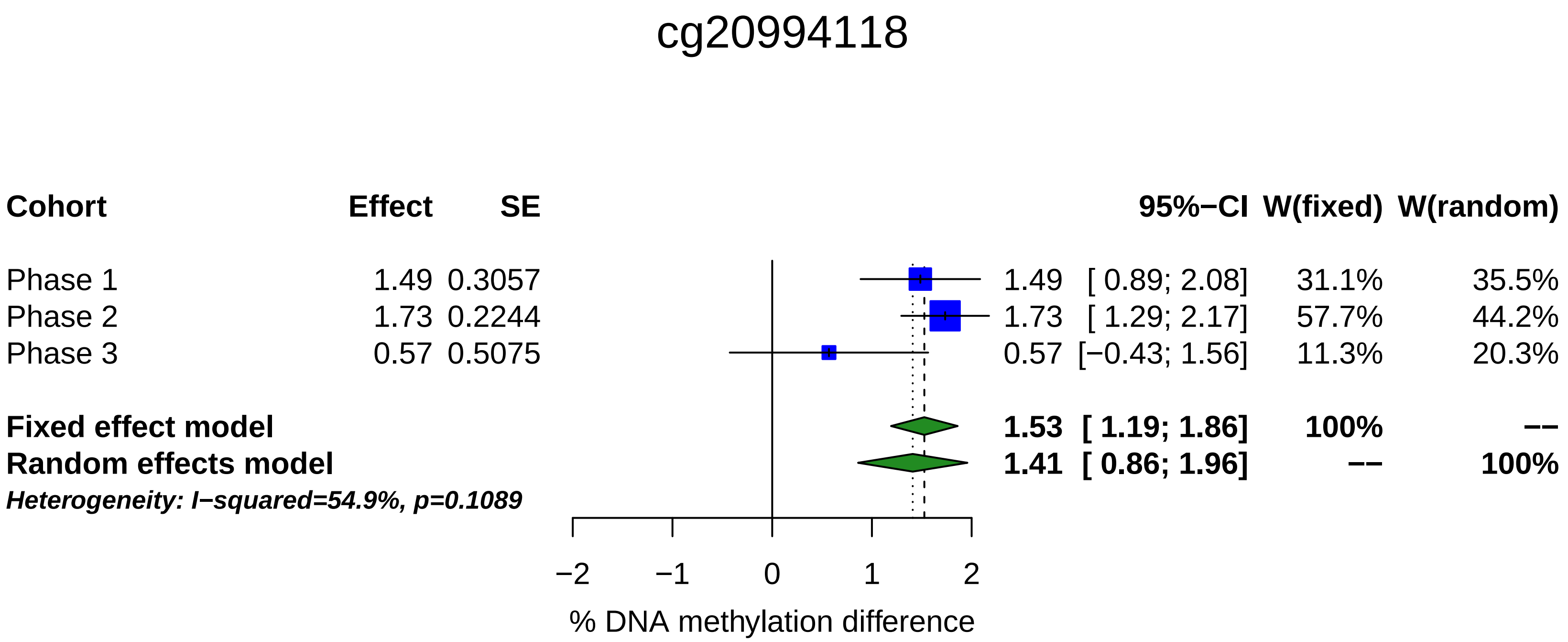
cg20994118
Cohort
Effect
SE
95%−CI
W(fixed)
W(random)
Phase 1 1.49 0.3057 1.49 [0.89; 2.08] 31.1% 35.5%
Phase 2 1.73 0.2244 1.73 [1.29; 2.17] 57.7% 44.2%
Phase 3 0.57 0.5075 0.57 [−0.43; 1.56] 11.3% 20.3%
Fixed effect model 1.53 [1.19; 1.86] 100% −−
Random effects model 1.41 [0.86; 1.96] −− 100%
Heterogeneity: I−squared=54.9%, p=0.1089
−2
−1
0
1
2
% DNA methylation difference

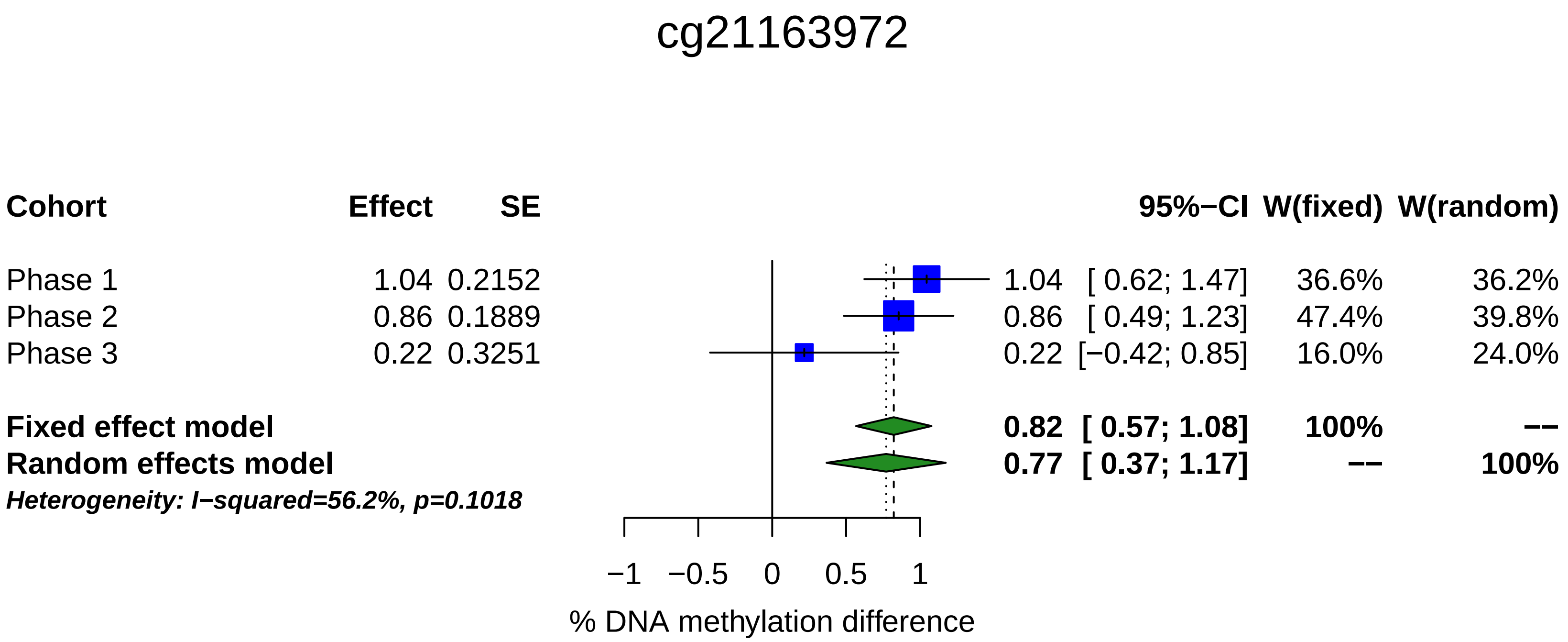
cg21163972
Cohort
Effect
SE
95%−CI
W(fixed)
W(random)
Phase 1 1.04 0.2152 1.04 [0.62; 1.47] 36.6% 36.2%
Phase 2 0.86 0.1889 0.86 [0.49; 1.23] 47.4% 39.8%
Phase 3 0.22 0.3251 0.22 [−0.42; 0.85] 16.0% 24.0%
Fixed effect model 0.82 [0.57; 1.08] 100% −−
Random effects model 0.77 [0.37; 1.17] −− 100%
Heterogeneity: I−squared=56.2%, p=0.1018
−1
−0.5
0
0.5
1
% DNA methylation difference

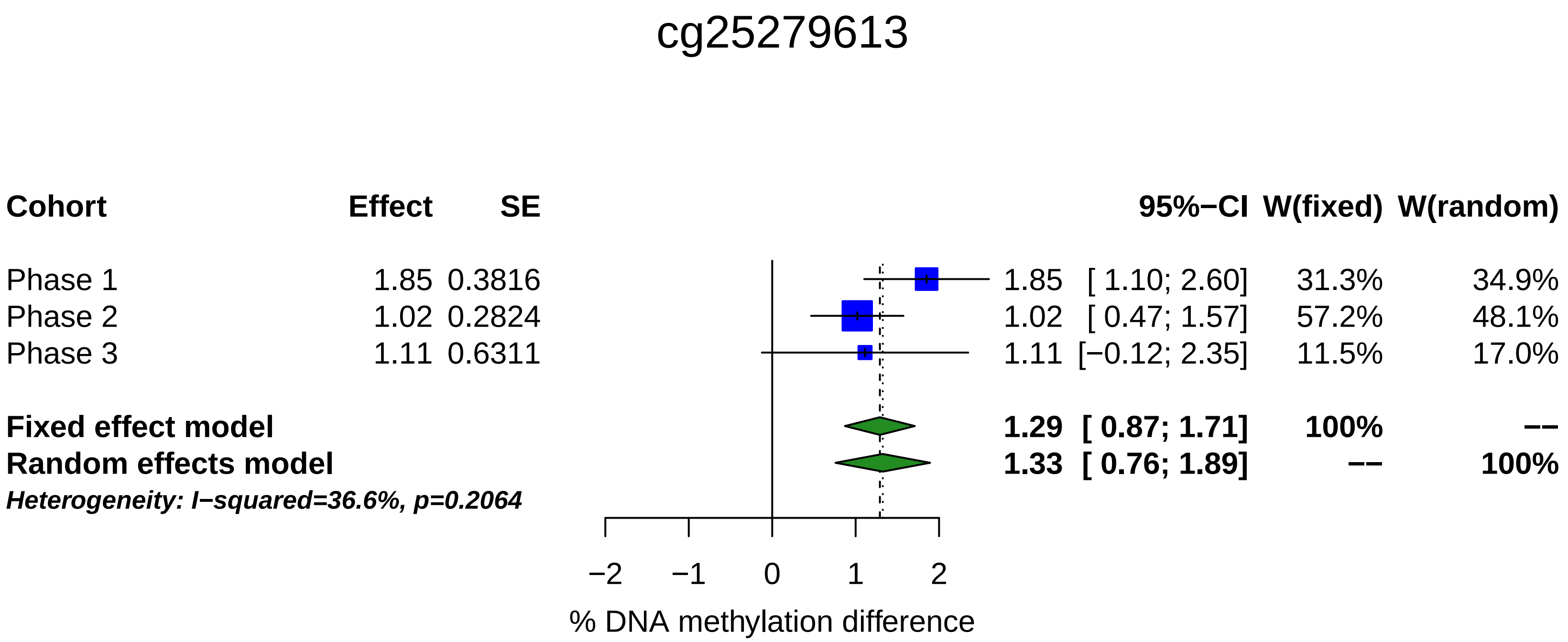
cg25279613
Cohort
Effect
SE
95%−CI
W(fixed)
W(random)
Phase 1 1.85 0.3816 1.85 [1.10; 2.60] 31.3% 34.9%
Phase 2 1.02 0.2824 1.02 [0.47; 1.57] 57.2% 48.1%
Phase 3 1.11 0.6311 1.11 [−0.12; 2.35] 11.5% 17.0%
Fixed effect model 1.29 [0.87; 1.71] 100% −−
Random effects model 1.33 [0.76; 1.89] −− 100%
Heterogeneity: I−squared=36.6%, p=0.2064
−2
−1
0
1
2
% DNA methylation difference

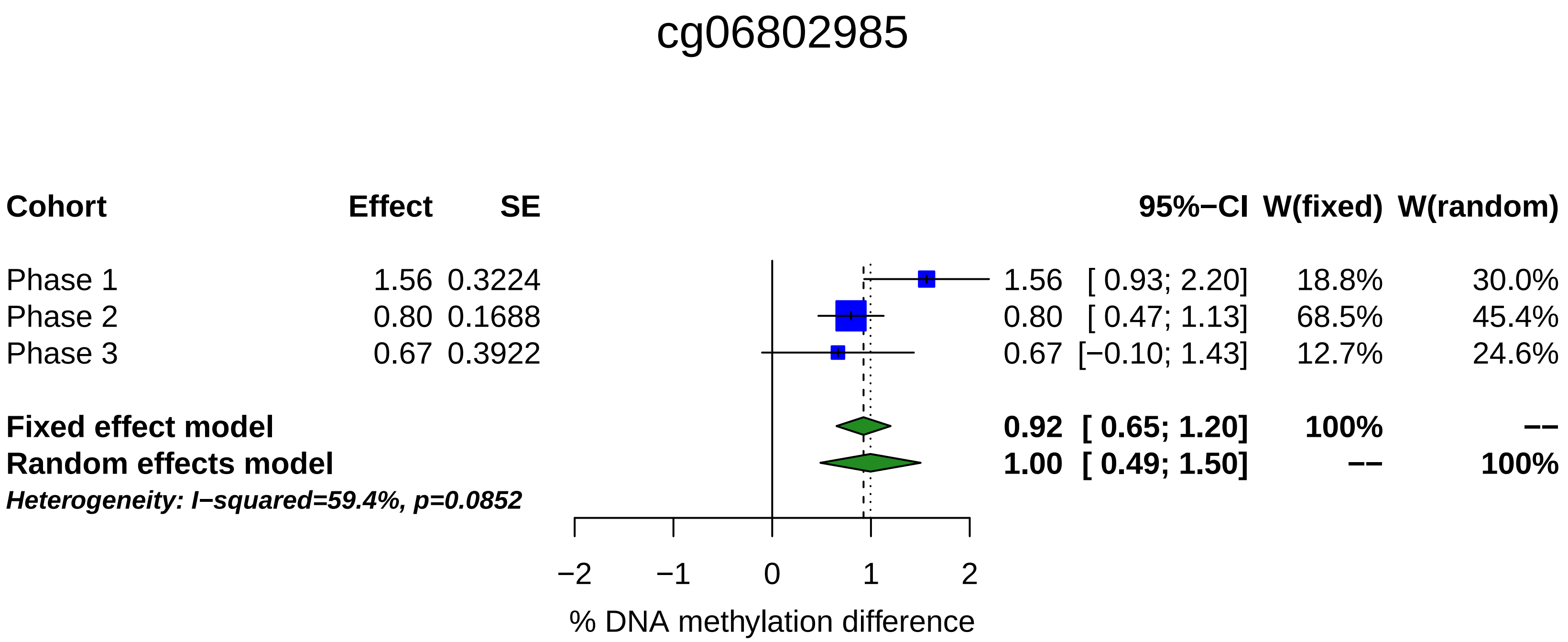
cg06802985
Cohort
Effect
SE
95%−CI
W(fixed)
W(random)
Phase 1 1.56 0.3224 1.56 [0.93; 2.20] 18.8% 30.0%
Phase 2 0.80 0.1688 0.80 [0.47; 1.13] 68.5% 45.4%
Phase 3 0.67 0.3922 0.67 [−0.10; 1.43] 12.7% 24.6%
Fixed effect model 0.92 [0.65; 1.20] 100% −−
Random effects model 1.00 [0.49; 1.50] −− 100%
Heterogeneity: I−squared=59.4%, p=0.0852
−2
−1
0
1
2
% DNA methylation difference

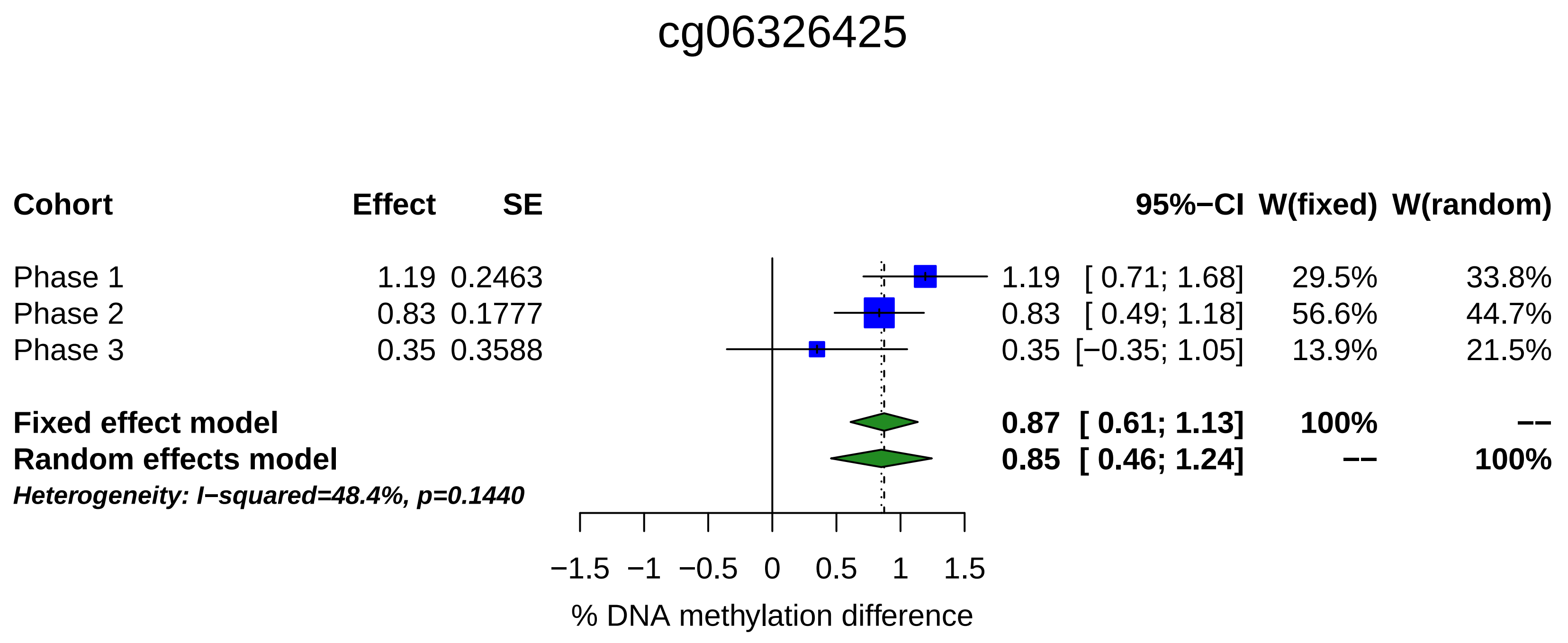
cg06326425
Cohort
Effect
SE
95%−CI
W(fixed)
W(random)
Phase 1 1.19 0.2463 1.19 [0.71; 1.68] 29.5% 33.8%
Phase 2 0.83 0.1777 0.83 [0.49; 1.18] 56.6% 44.7%
Phase 3 0.35 0.3588 0.35 [−0.35; 1.05] 13.9% 21.5%
Fixed effect model 0.87 [0.61; 1.13] 100% −−
Random effects model 0.85 [0.46; 1.24] −− 100%
Heterogeneity: I−squared=48.4%, p=0.1440
−1.5 −1 −0.5 0 0.5 1 1.5
% DNA methylation difference

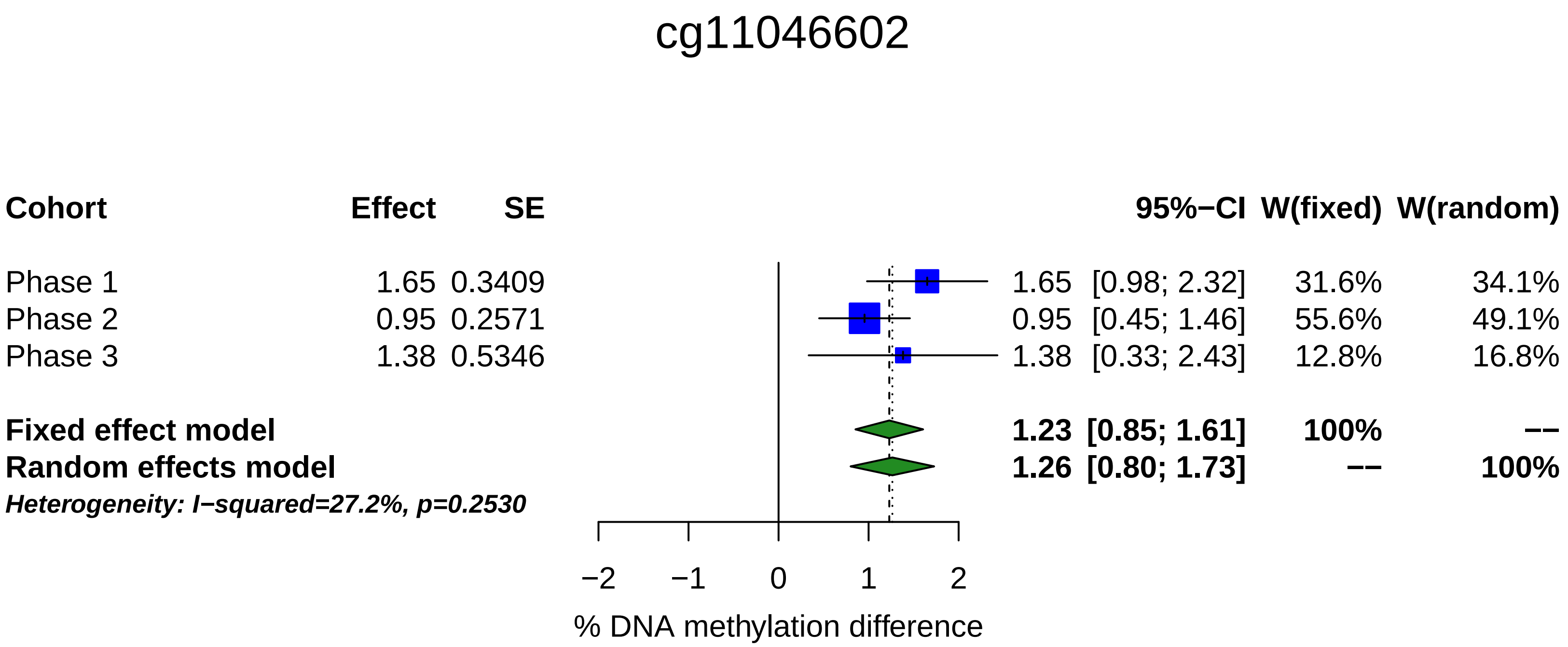
cg11046602
Cohort
Effect
SE
95%−CI
W(fixed)
W(random)
Phase 1 1.65 0.3409 1.65 [0.98; 2.32] 31.6% 34.1%
Phase 2 0.95 0.2571 0.95 [0.45; 1.46] 55.6% 49.1%
Phase 3 1.38 0.5346 1.38 [0.33; 2.43] 12.8% 16.8%
Fixed effect model 1.23 [0.85; 1.61] 100% −−
Random effects model 1.26 [0.80; 1.73] −− 100%
Heterogeneity: I−squared=27.2%, p=0.2530
−2
−1
0
1
2
% DNA methylation difference

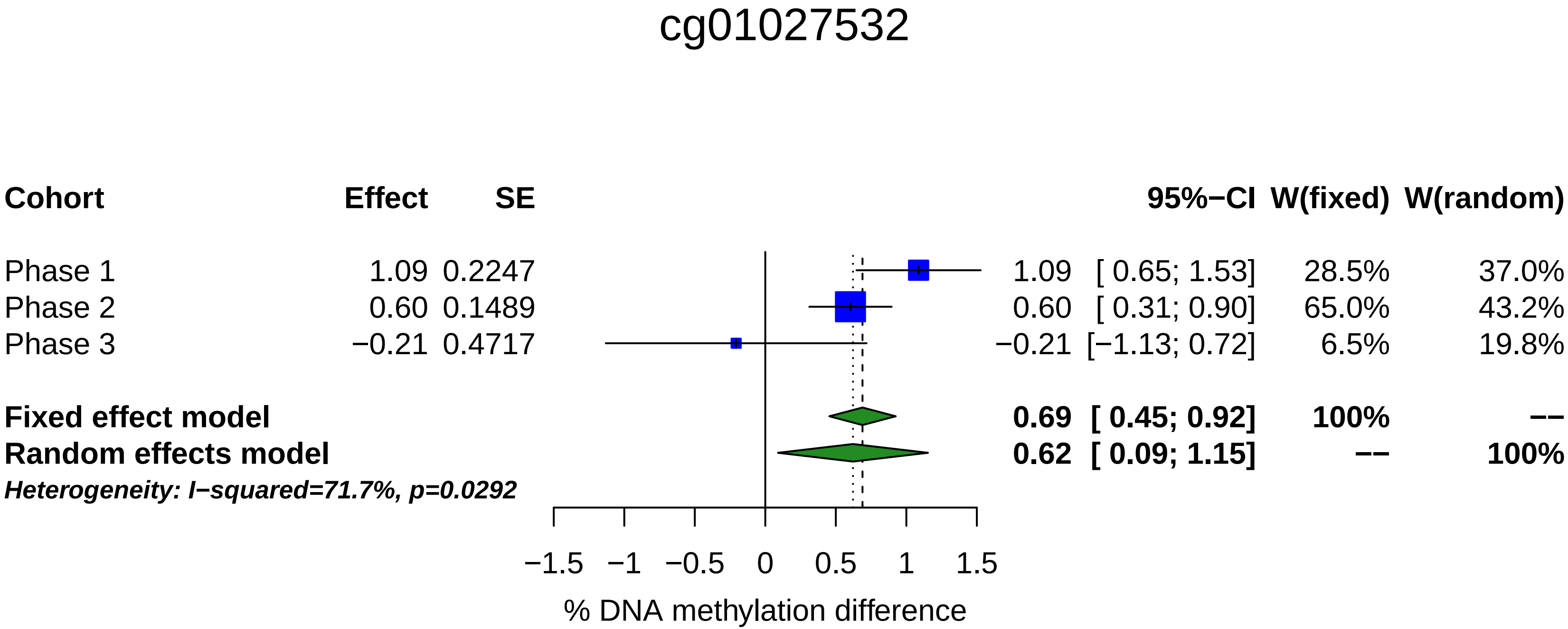
cg01027532
Cohort
Effect
SE
95%−CI
W(fixed)
W(random)
Phase 1 1.09 0.2247 1.09 [0.65; 1.53] 28.5% 37.0%
Phase 2 0.60 0.1489 0.60 [0.31; 0.90] 65.0% 43.2%
Phase 3 −0.21 0.4717 −0.21 [−1.13; 0.72] 6.5% 19.8%
Fixed effect model 0.69 [0.45; 0.92] 100% −−
Random effects model 0.62 [0.09; 1.15] −− 100%
Heterogeneity: I−squared=71.7%, p=0.0292
−1.5 −1 −0.5 0 0.5 1 1.5
% DNA methylation difference

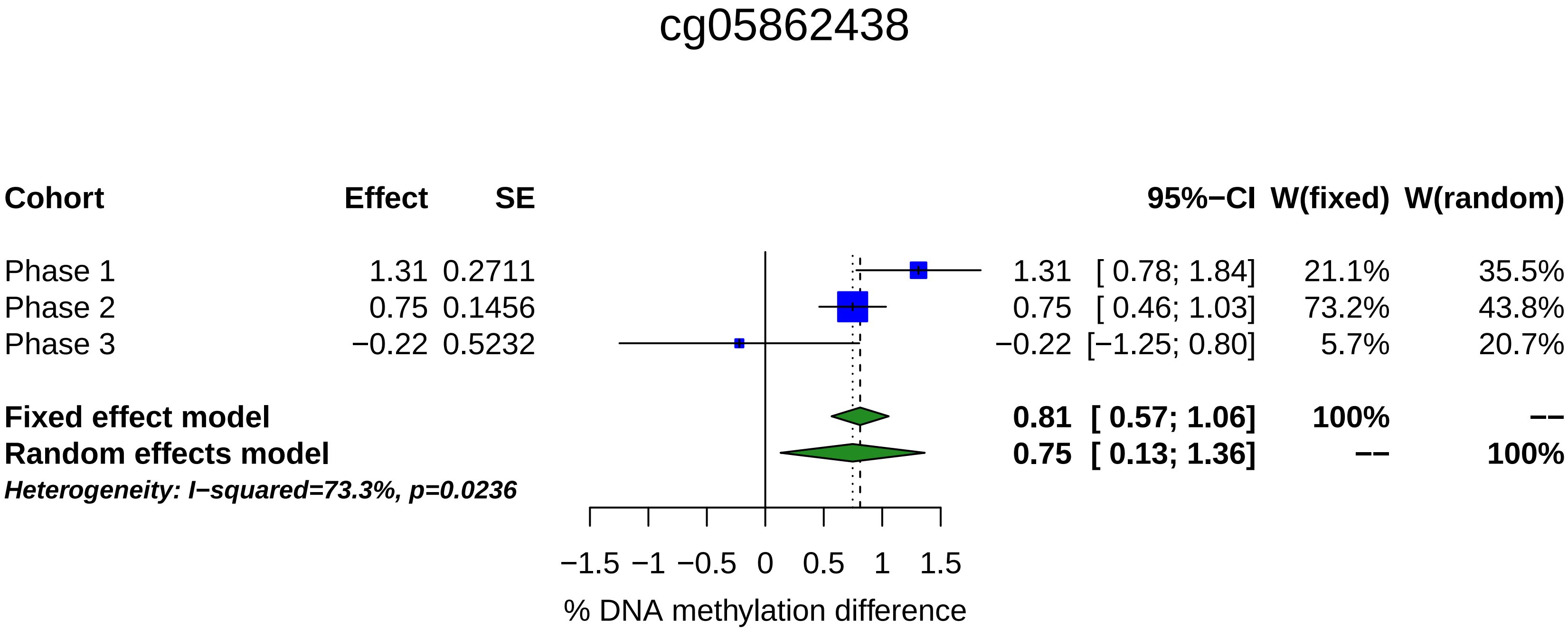
cg05862438
Cohort
Effect
SE
95%−CI
W(fixed)
W(random)
Phase 1 1.31 0.2711 1.31 [0.78; 1.84] 21.1% 35.5%
Phase 2 0.75 0.1456 0.75 [0.46; 1.03] 73.2% 43.8%
Phase 3 −0.22 0.5232 −0.22 [−1.25; 0.80] 5.7% 20.7%
Fixed effect model 0.81 [0.57; 1.06] 100% −−
Random effects model 0.75 [0.13; 1.36] −− 100%
Heterogeneity: I−squared=73.3%, p=0.0236
−1.5 −1 −0.5 0 0.5 1 1.5
% DNA methylation difference

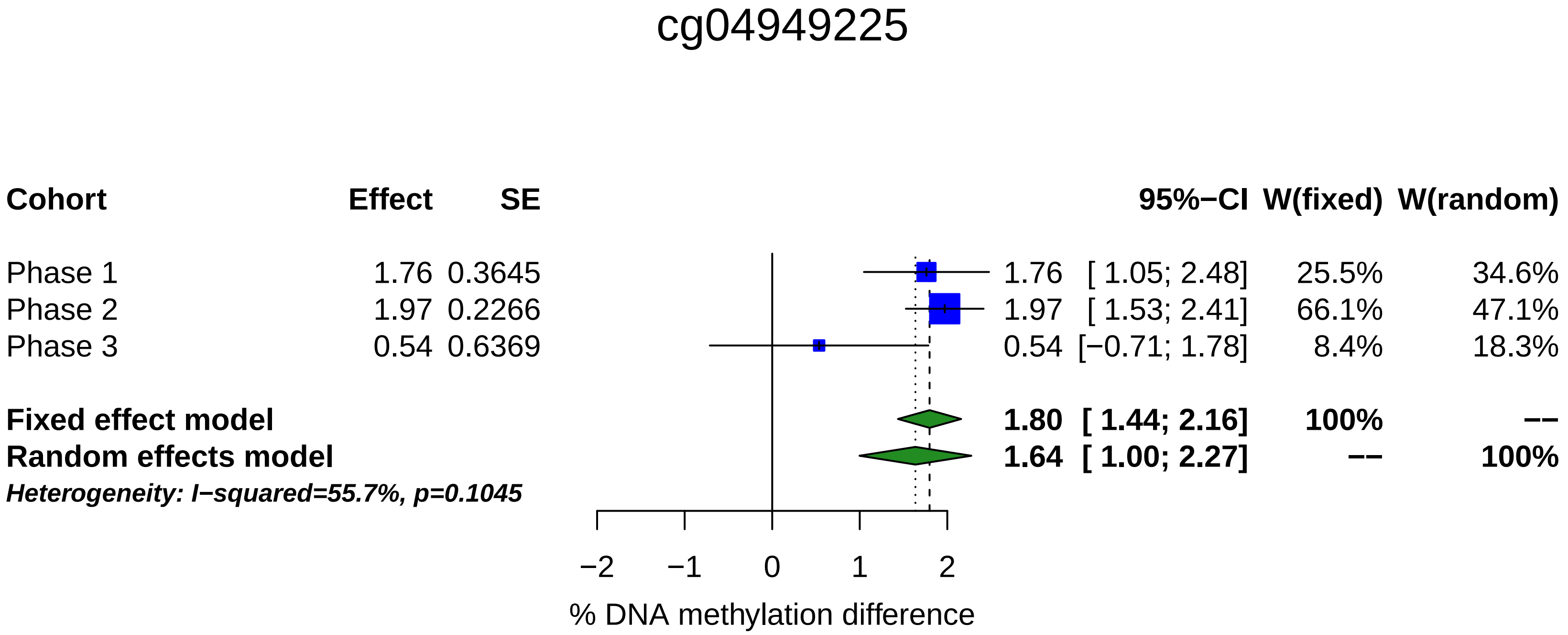

cg04949225
Cohort
Effect
SE
95%−CI
W(fixed)
W(random)
Phase 1 1.76 0.3645 1.76 [1.05; 2.48] 25.5% 34.6%
Phase 2 1.97 0.2266 1.97 [1.53; 2.41] 66.1% 47.1%
Phase 3 0.54 0.6369 0.54 [−0.71; 1.78] 8.4% 18.3%
Fixed effect model 1.80 [1.44; 2.16] 100% −−
Random effects model 1.64 [1.00; 2.27] −− 100%
Heterogeneity: I−squared=55.7%, p=0.1045
−2
−1
0
1
2
% DNA methylation difference

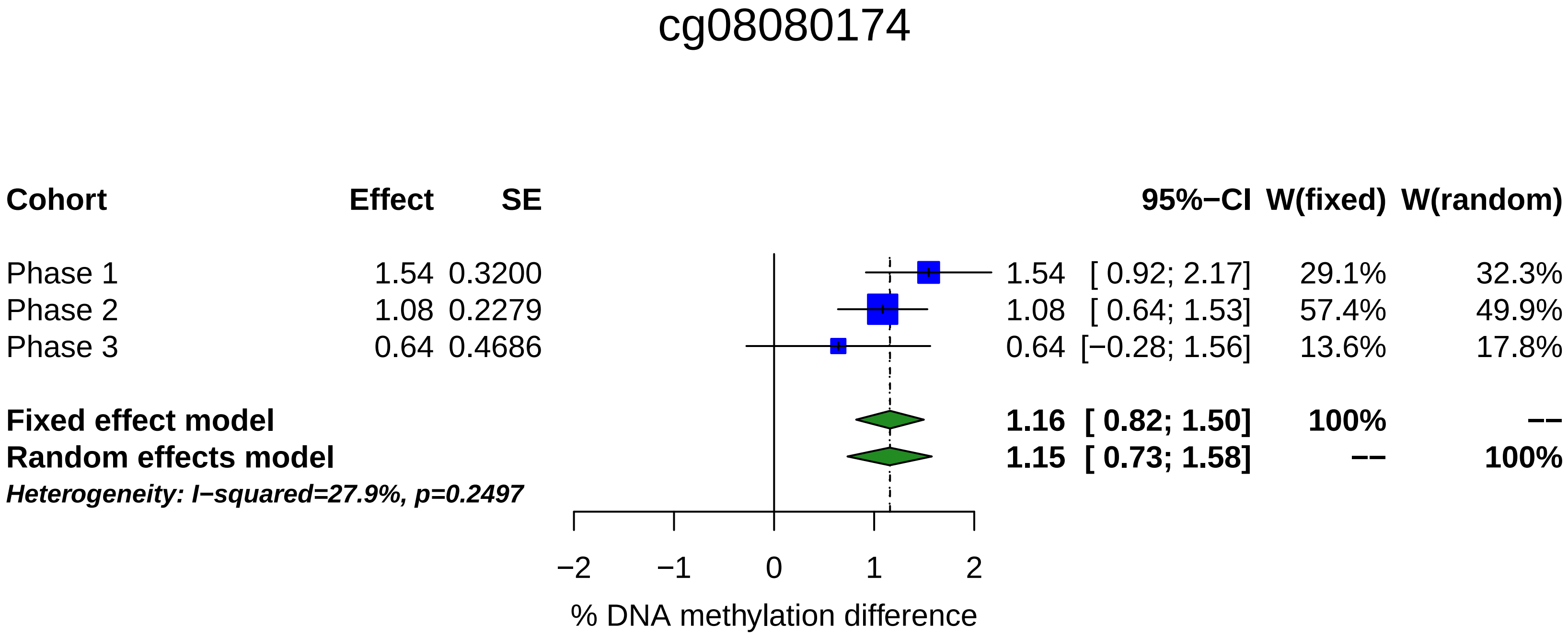
cg08080174
Cohort
Effect
SE
95%−CI
W(fixed)
W(random)
Phase 1 1.54 0.3200 1.54 [0.92; 2.17] 29.1% 32.3%
Phase 2 1.08 0.2279 1.08 [0.64; 1.53] 57.4% 49.9%
Phase 3 0.64 0.4686 0.64 [−0.28; 1.56] 13.6% 17.8%
Fixed effect model 1.16 [0.82; 1.50] 100% −−
Random effects model 1.15 [0.73; 1.58] −− 100%
Heterogeneity: I−squared=27.9%, p=0.2497
−2
−1
0
1
2
% DNA methylation difference

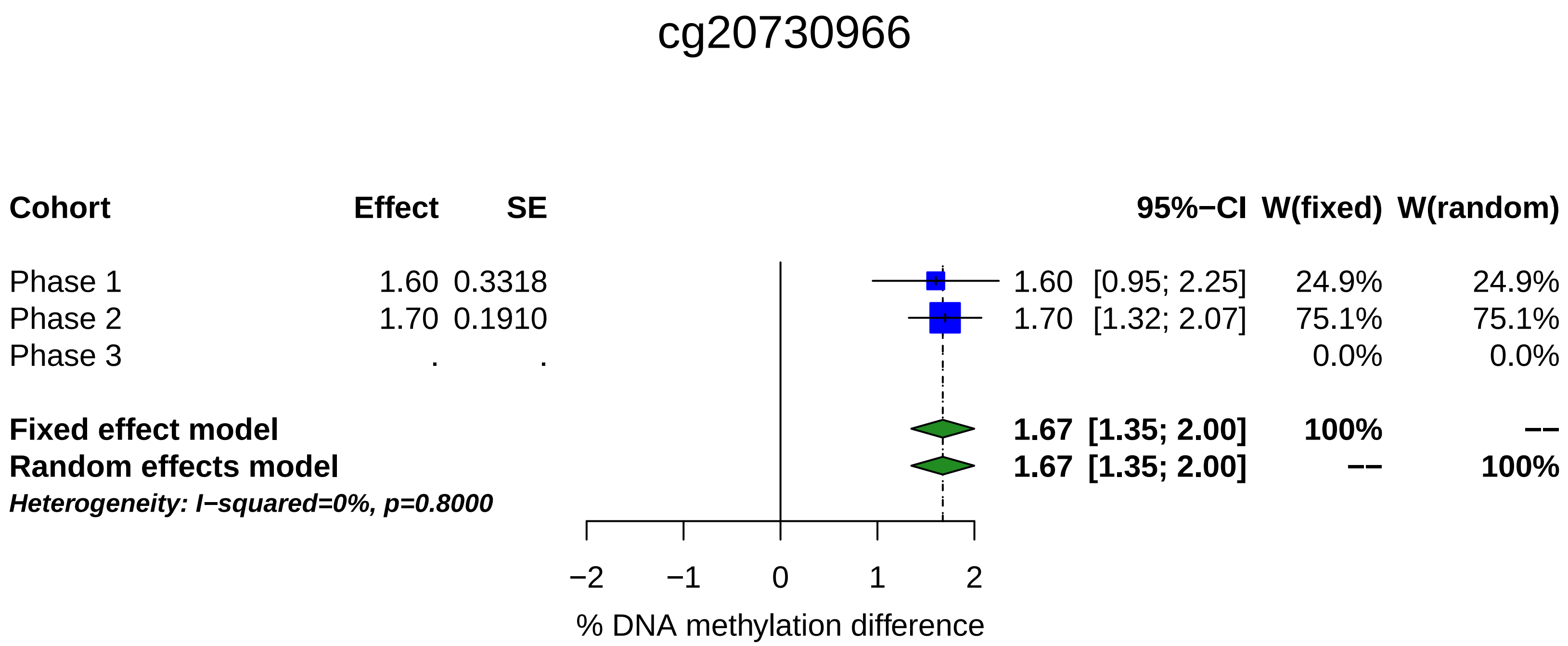
cg20730966
Cohort
Effect
SE
95%−CI
W(fixed)
W(random)
Phase 1
1.60
0.3318
1.60
[0.95; 2.25]
24.9%
24.9%
Phase 2
1.70
0.1910
1.70
[1.32; 2.07]
75.1%
75.1%
Phase 3
.
.
0.0%
0.0%
Fixed effect model
1.67
[1.35; 2.00]
100%
−−
Random effects model
1.67
[1.35; 2.00]
−−
100%
Heterogeneity: I−squared=0%, p=0.8000
−2
−1
0
1
2
% DNA methylation difference

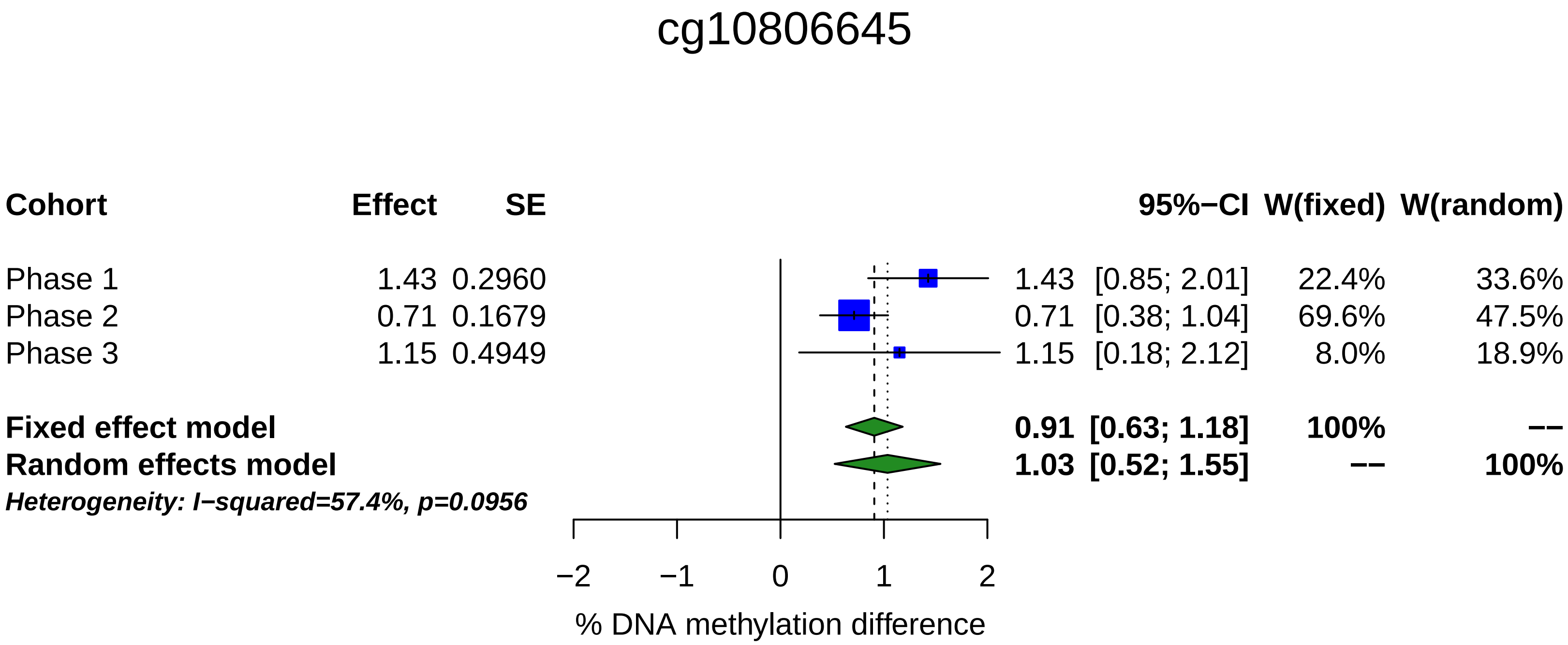
cg10806645
Cohort
Effect
SE
95%−CI
W(fixed)
W(random)
Phase 1 1.43 0.2960 1.43 [0.85; 2.01] 22.4% 33.6%
Phase 2 0.71 0.1679 0.71 [0.38; 1.04] 69.6% 47.5%
Phase 3 1.15 0.4949 1.15 [0.18; 2.12] 8.0% 18.9%
Fixed effect model 0.91 [0.63; 1.18] 100% −−
Random effects model 1.03 [0.52; 1.55] −− 100%
Heterogeneity: I−squared=57.4%, p=0.0956
−2
−1
0
1
2
% DNA methylation difference

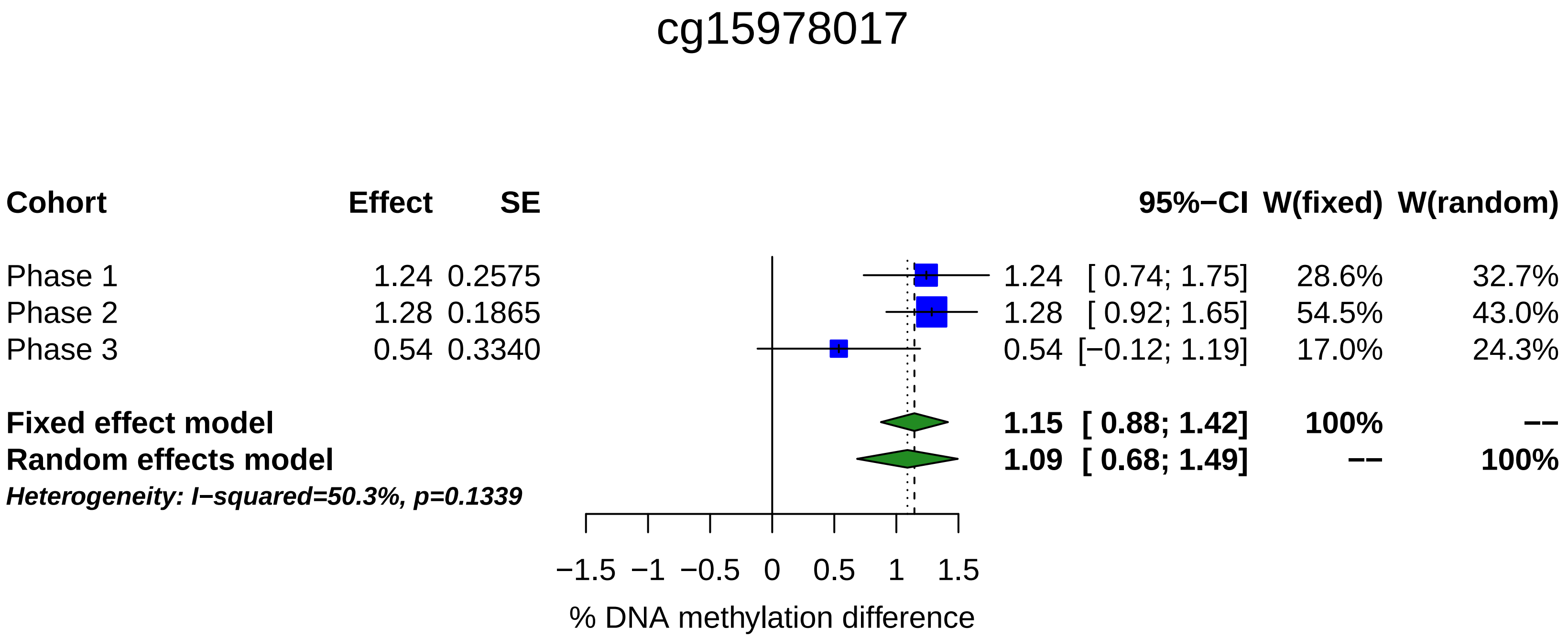
cg15978017
Cohort
Effect
SE
95%−CI
W(fixed)
W(random)
Phase 1 1.24 0.2575 1.24 [0.74; 1.75] 28.6% 32.7%
Phase 2 1.28 0.1865 1.28 [0.92; 1.65] 54.5% 43.0%
Phase 3 0.54 0.3340 0.54 [−0.12; 1.19] 17.0% 24.3%
Fixed effect model 1.15 [0.88; 1.42] 100% −−
Random effects model 1.09 [0.68; 1.49] −− 100%
Heterogeneity: I−squared=50.3%, p=0.1339
−1.5 −1 −0.5 0 0.5 1 1.5
% DNA methylation difference

cg12763430

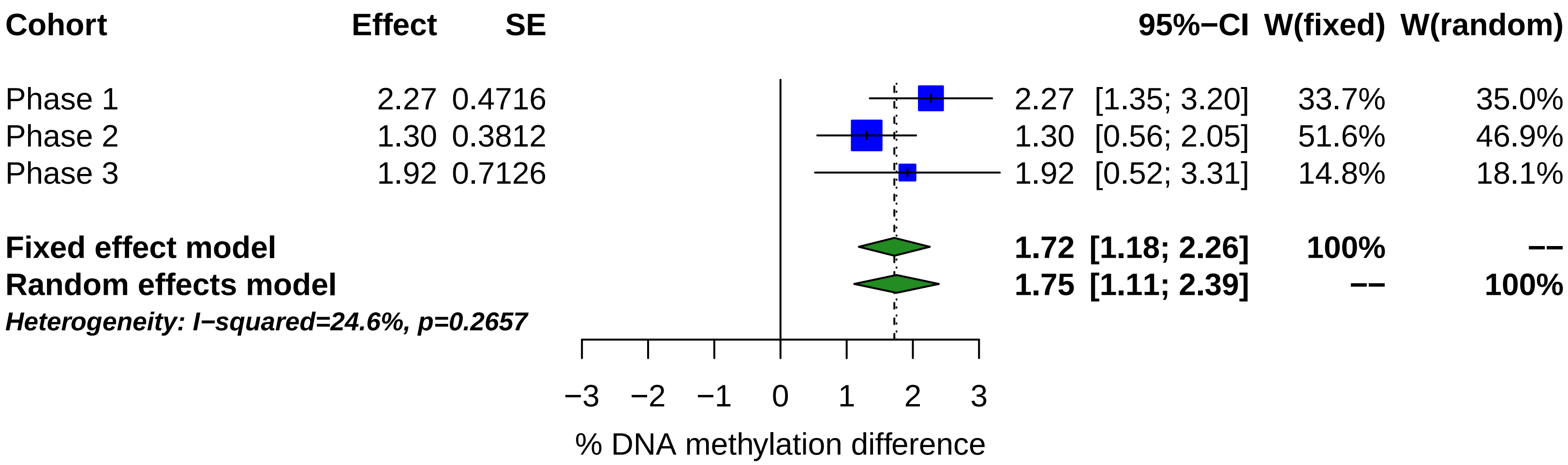
Cohort Effect SE 95%−CI W(fixed) W(random)
Phase 1 2.27 0.4716 2.27 [1.35; 3.20] 33.7% 35.0%
Phase 2 1.30 0.3812 1.30 [0.56; 2.05] 51.6% 46.9%
Phase 3 1.92 0.7126 1.92 [0.52; 3.31] 14.8% 18.1%
Fixed effect model 1.72 [1.18; 2.26] 100% −−
Random effects model 1.75 [1.11; 2.39] −− 100%
Heterogeneity: I−squared=24.6%, p=0.2657
−3 −2 −1 0 1 2 3
% DNA methylation difference

cg06922393

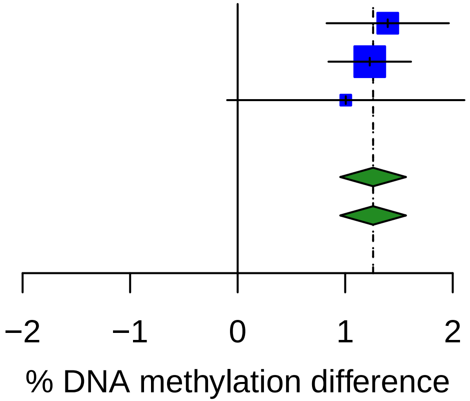

Cohort	Effect	SE	95%−CI	W(fixed)	W(random)
Phase 1	1.40	0.2901	1.40 [0.83; 1.96]	28.9%	28.9%
Phase 2	1.23	0.1959	1.23 [0.84; 1.61]	63.4%	63.4%
Phase 3	1.01	0.5629	1.01 [−0.10; 2.11]	7.7%	7.7%
**Fixed effect model**			**1.26 [0.95; 1.57]**	**100%**	**−−**
**Random effects model**			**1.26 [0.95; 1.57]**	**−−**	**100%**

Heterogeneity: I−squared=0%, p=0.7989

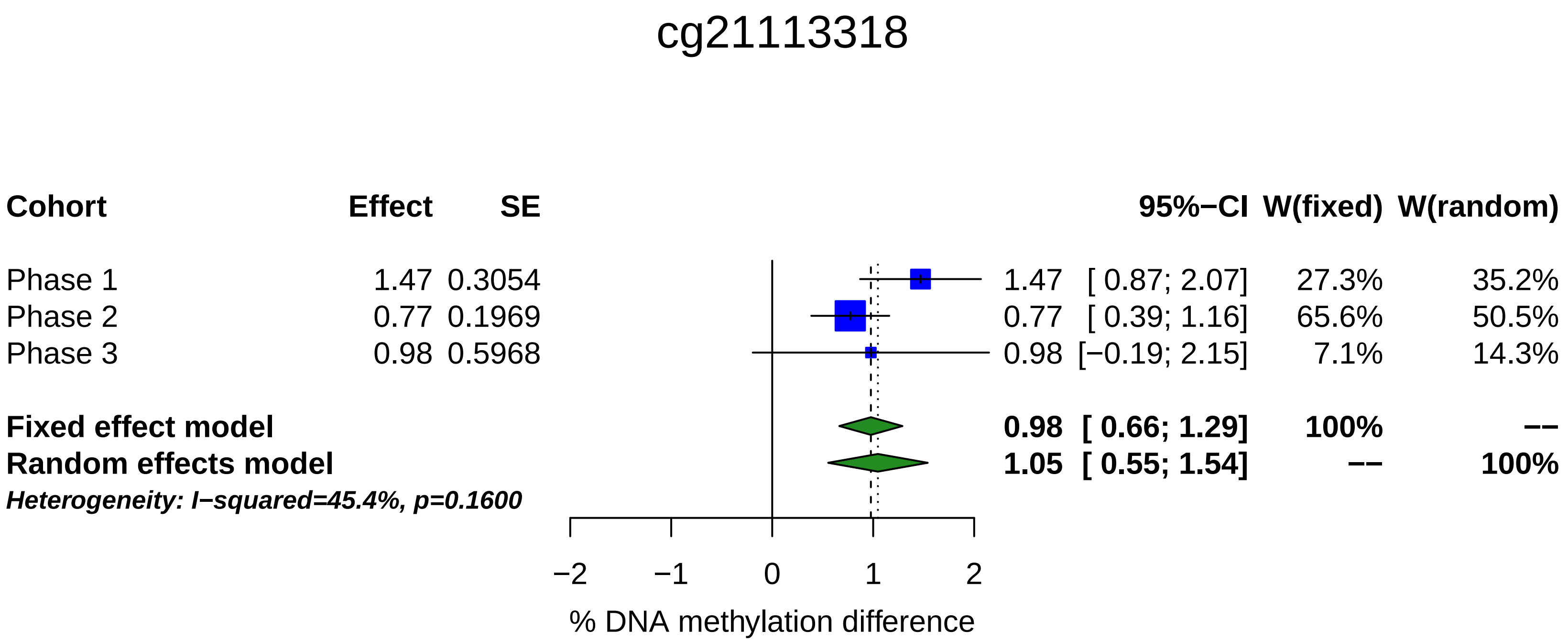
cg21113318
Cohort
Effect
SE
95%−CI
W(fixed)
W(random)
Phase 1 1.47 0.3054 1.47 [0.87; 2.07] 27.3% 35.2%
Phase 2 0.77 0.1969 0.77 [0.39; 1.16] 65.6% 50.5%
Phase 3 0.98 0.5968 0.98 [−0.19; 2.15] 7.1% 14.3%
Fixed effect model 0.98 [0.66; 1.29] 100% −−
Random effects model 1.05 [0.55; 1.54] −− 100%
Heterogeneity: I−squared=45.4%, p=0.1600
−2
−1
0
1
2
% DNA methylation difference

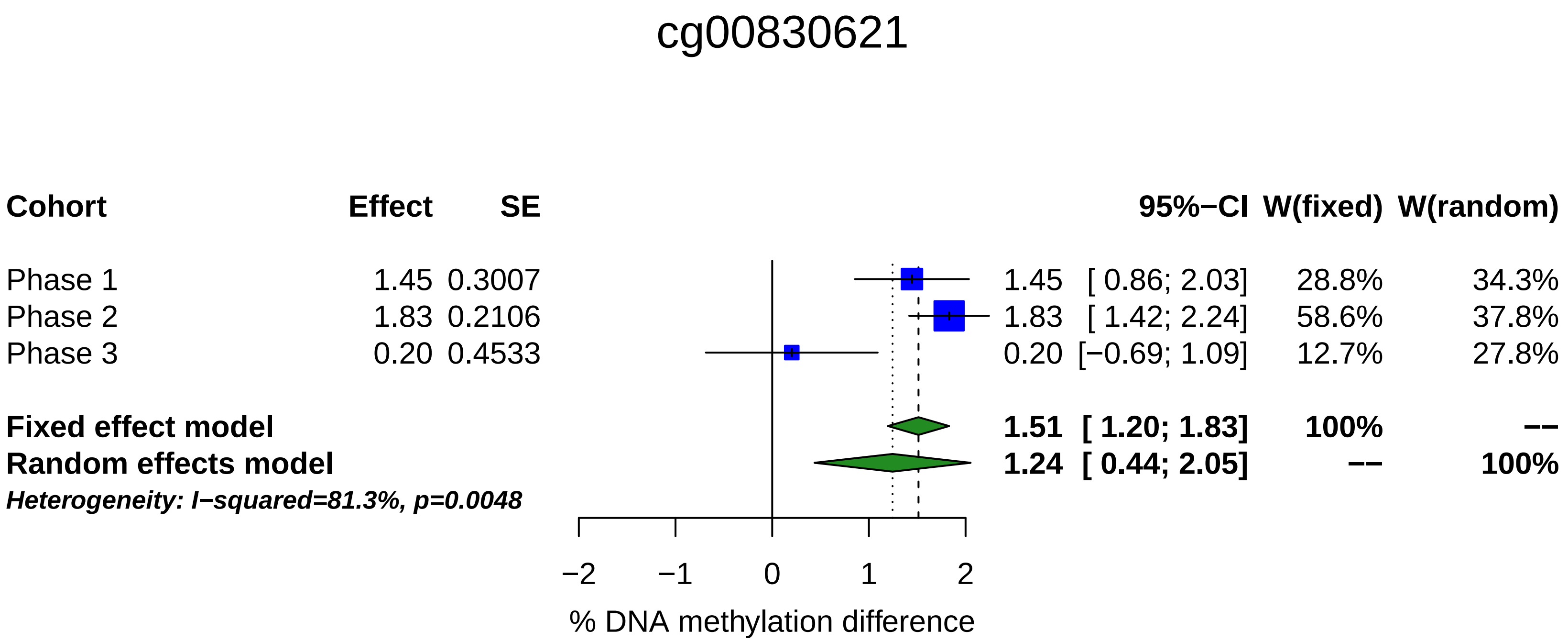
cg00830621
Cohort
Effect
SE
95%−CI
W(fixed)
W(random)
Phase 1 1.45 0.3007 1.45 [0.86; 2.03] 28.8% 34.3%
Phase 2 1.83 0.2106 1.83 [1.42; 2.24] 58.6% 37.8%
Phase 3 0.20 0.4533 0.20 [−0.69; 1.09] 12.7% 27.8%
Fixed effect model 1.51 [1.20; 1.83] 100% −−
Random effects model 1.24 [0.44; 2.05] −− 100%
Heterogeneity: I−squared=81.3%, p=0.0048
−2
−1
0
1
2
% DNA methylation difference

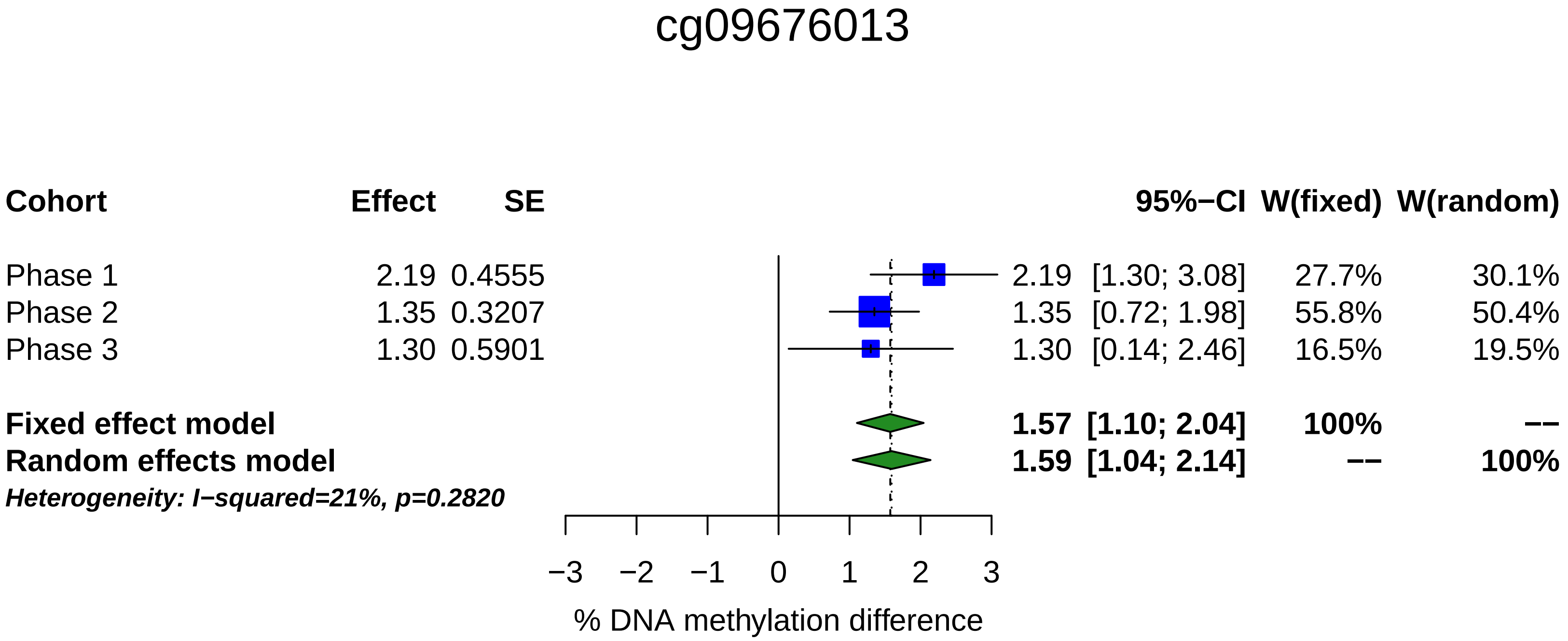
cg09676013
Cohort
Effect
SE
95%−CI
W(fixed)
W(random)
Phase 1 2.19 0.4555 2.19 [1.30; 3.08] 27.7% 30.1%
Phase 2 1.35 0.3207 1.35 [0.72; 1.98] 55.8% 50.4%
Phase 3 1.30 0.5901 1.30 [0.14; 2.46] 16.5% 19.5%
Fixed effect model 1.57 [1.10; 2.04] 100% −−
Random effects model 1.59 [1.04; 2.14] −− 100%
Heterogeneity: I−squared=21%, p=0.2820
−3 −2 −1 0 1 2 3
% DNA methylation difference

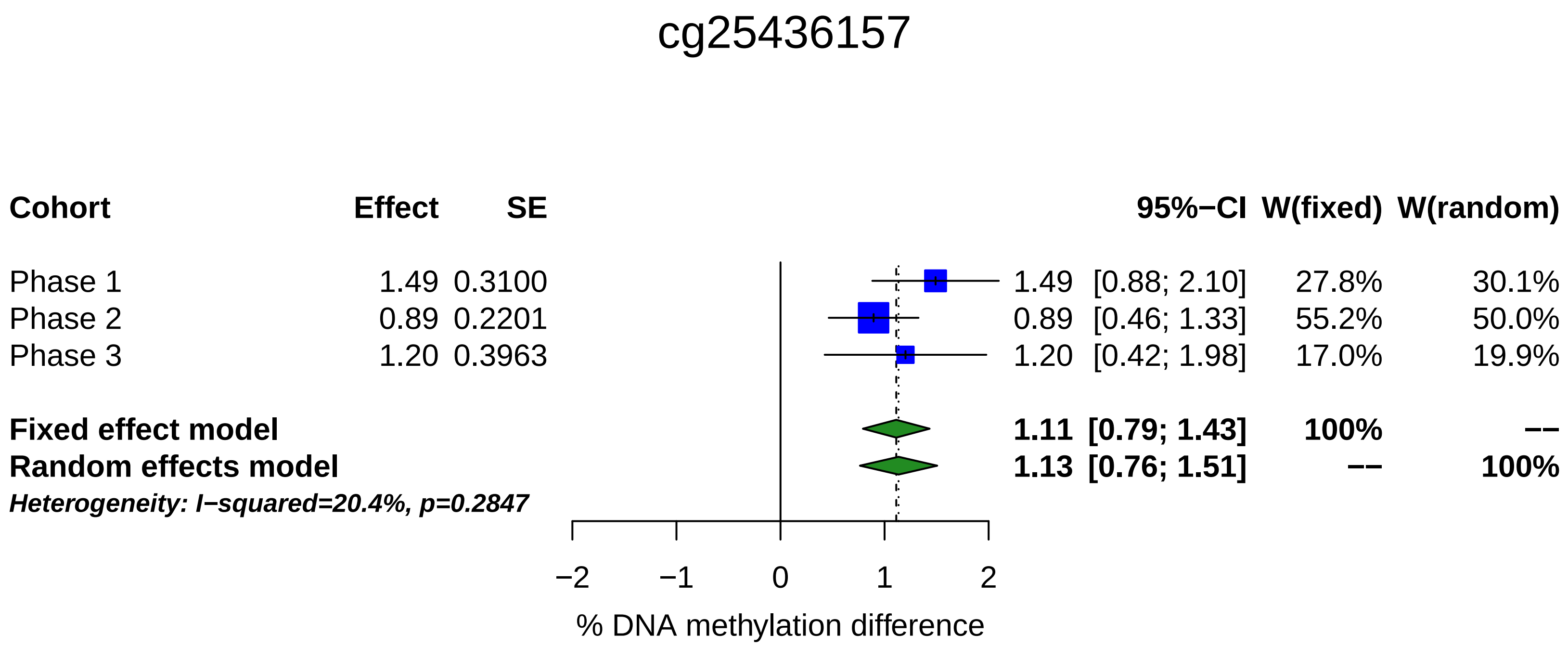
cg25436157
Cohort
Effect
SE
95%−CI
W(fixed)
W(random)
Phase 1 1.49 0.3100 1.49 [0.88; 2.10] 27.8% 30.1%
Phase 2 0.89 0.2201 0.89 [0.46; 1.33] 55.2% 50.0%
Phase 3 1.20 0.3963 1.20 [0.42; 1.98] 17.0% 19.9%
Fixed effect model 1.11 [0.79; 1.43] 100% −−
Random effects model 1.13 [0.76; 1.51] −− 100%
Heterogeneity: I−squared=20.4%, p=0.2847
−2
−1
0
1
2
% DNA methylation difference

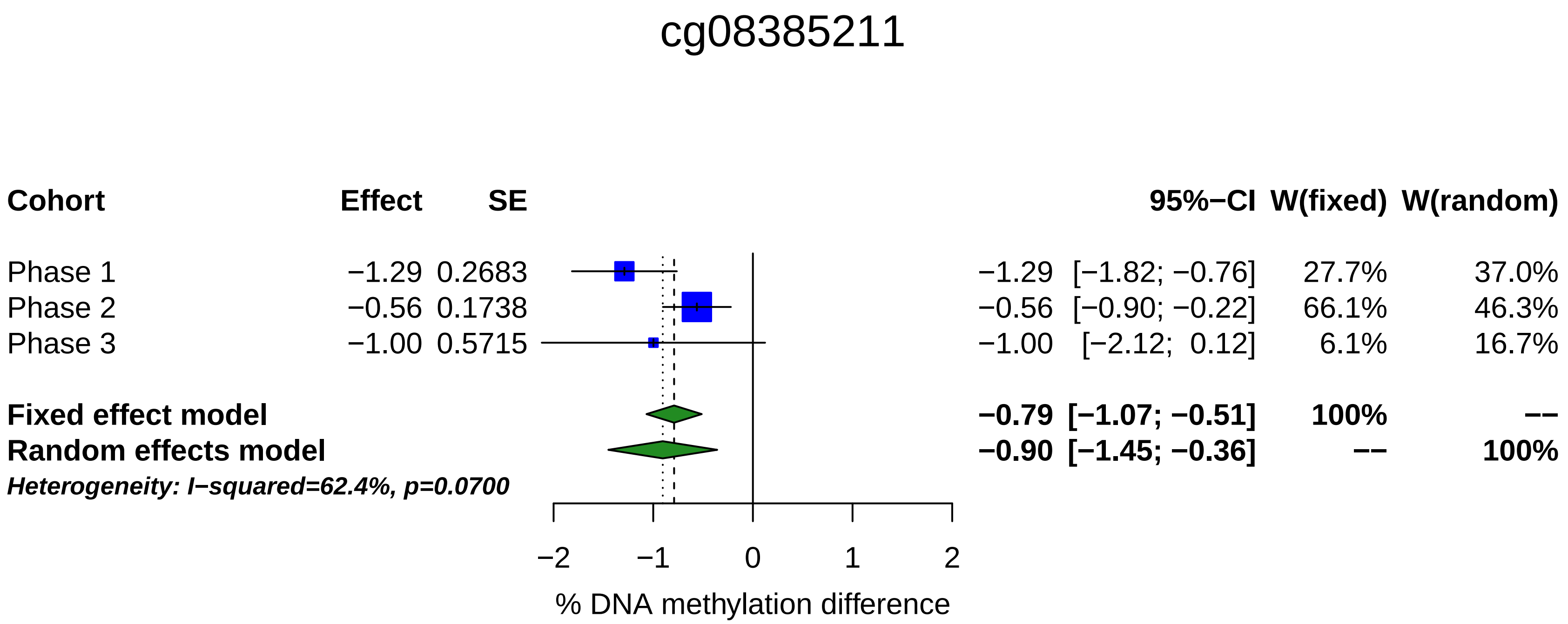
cg08385211
Cohort
Effect
SE
95%−CI
W(fixed)
W(random)
Phase 1 −1.29 0.2683 −1.29 [−1.82; −0.76] 27.7% 37.0%
Phase 2 −0.56 0.1738 −0.56 [−0.90; −0.22] 66.1% 46.3%
Phase 3 −1.00 0.5715 −1.00 [−2.12; 0.12] 6.1% 16.7%
Fixed effect model −0.79 [−1.07; −0.51] 100% --
Random effects model −0.90 [−1.45; −0.36] -- 100%
Heterogeneity: I−squared=62.4%, p=0.0700
−2
−1
0
1
2
% DNA methylation difference

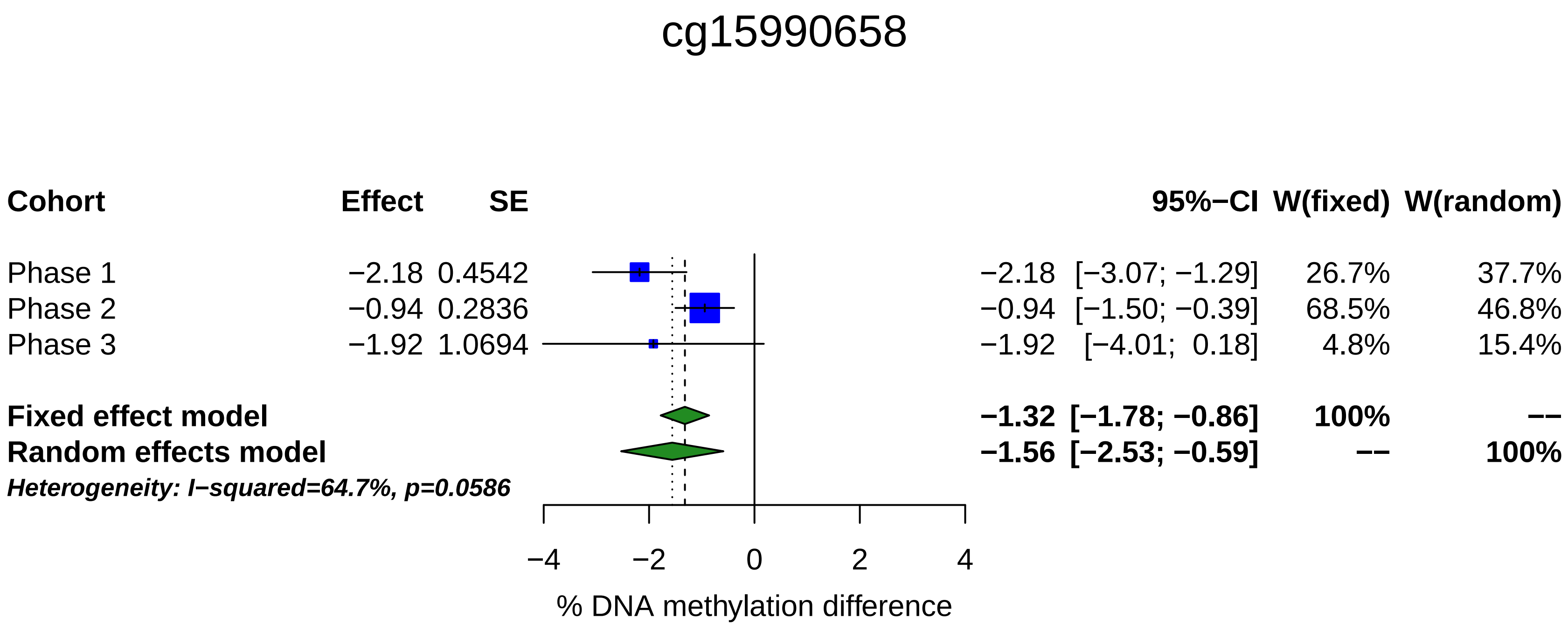
cg15990658
Cohort
Effect
SE
95%−CI
W(fixed)
W(random)
Phase 1 −2.18 0.4542 −2.18 [−3.07; −1.29] 26.7% 37.7%
Phase 2 −0.94 0.2836 −0.94 [−1.50; −0.39] 68.5% 46.8%
Phase 3 −1.92 1.0694 −1.92 [−4.01; 0.18] 4.8% 15.4%
Fixed effect model −1.32 [−1.78; −0.86] 100% −−
Random effects model −1.56 [−2.53; −0.59] −− 100%
Heterogeneity: I−squared=64.7%, p=0.0586
−4
−2
0
2
4
% DNA methylation difference

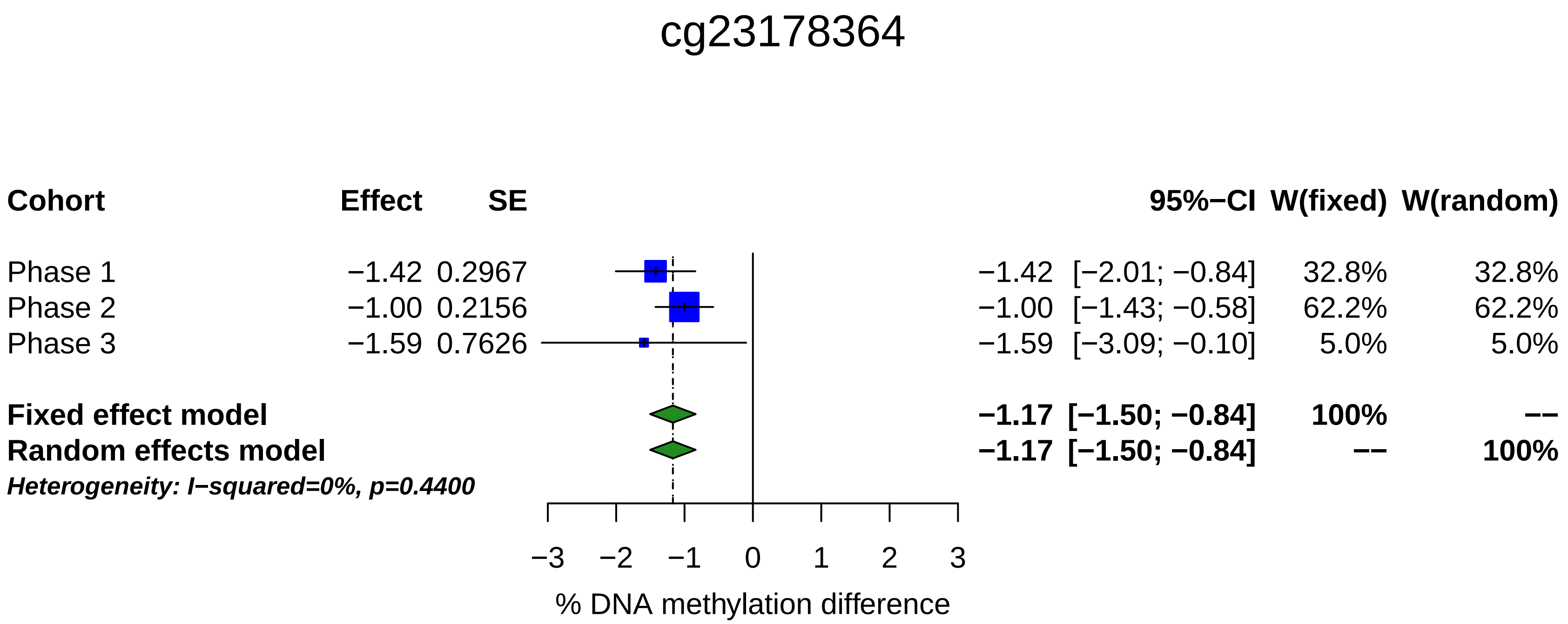
cg23178364
Cohort
Effect
SE
95%−CI
W(fixed)
W(random)
Phase 1 −1.42 0.2967 −1.42 [−2.01; −0.84] 32.8% 32.8%
Phase 2 −1.00 0.2156 −1.00 [−1.43; −0.58] 62.2% 62.2%
Phase 3 −1.59 0.7626 −1.59 [−3.09; −0.10] 5.0% 5.0%
Fixed effect model −1.17 [−1.50; −0.84] 100% −−
Random effects model −1.17 [−1.50; −0.84] −− 100%
Heterogeneity: I−squared=0%, p=0.4400
−3
−2
−1
0
1
2
3
% DNA methylation difference

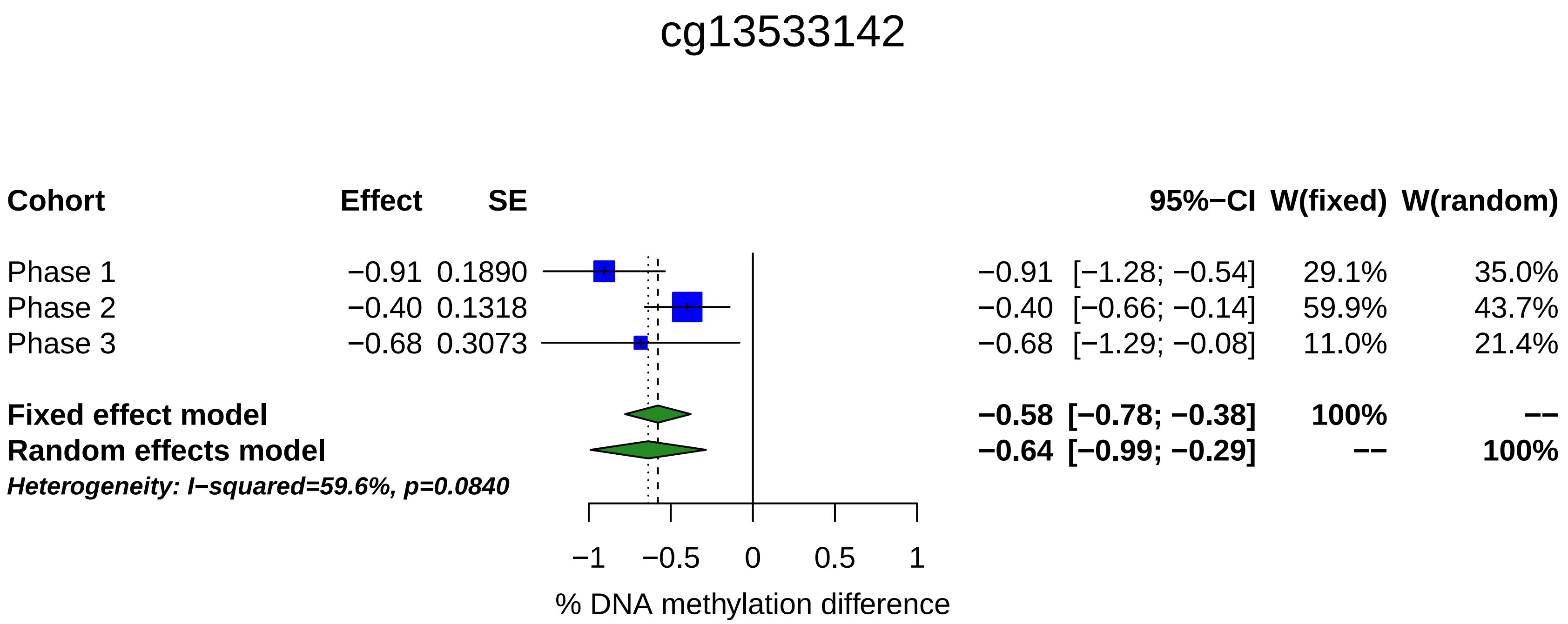
cg13533142
Cohort
Effect
SE
95%−CI
W(fixed)
W(random)
Phase 1 −0.91 0.1890 −0.91 [−1.28; −0.54] 29.1% 35.0%
Phase 2 −0.40 0.1318 −0.40 [−0.66; −0.14] 59.9% 43.7%
Phase 3 −0.68 0.3073 −0.68 [−1.29; −0.08] 11.0% 21.4%
Fixed effect model −0.58 [−0.78; −0.38] 100% −−
Random effects model −0.64 [−0.99; −0.29] −− 100%
Heterogeneity: I−squared=59.6%, p=0.0840
−1
−0.5
0
0.5
1
% DNA methylation difference

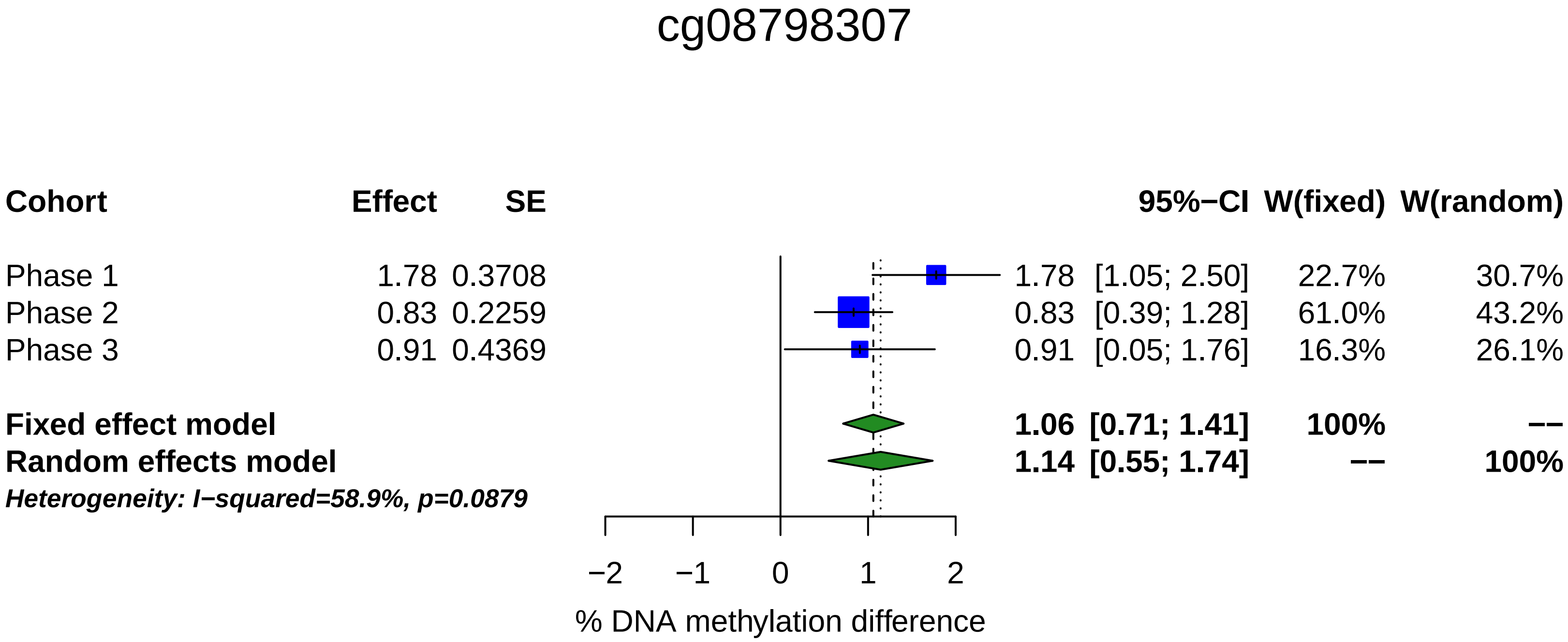

cg08798307
Cohort
Effect
SE
95%−CI
W(fixed)
W(random)
Phase 1
1.78
0.3708
1.78 [1.05; 2.50]
22.7%
30.7%
Phase 2
0.83
0.2259
0.83 [0.39; 1.28]
61.0%
43.2%
Phase 3
0.91
0.4369
0.91 [0.05; 1.76]
16.3%
26.1%
Fixed effect model
1.06 [0.71; 1.41]
100%
−−
Random effects model
1.14 [0.55; 1.74]
−−
100%
Heterogeneity: I−squared=58.9%, p=0.0879
−2
−1
0
1
2
% DNA methylation difference

cg09504612

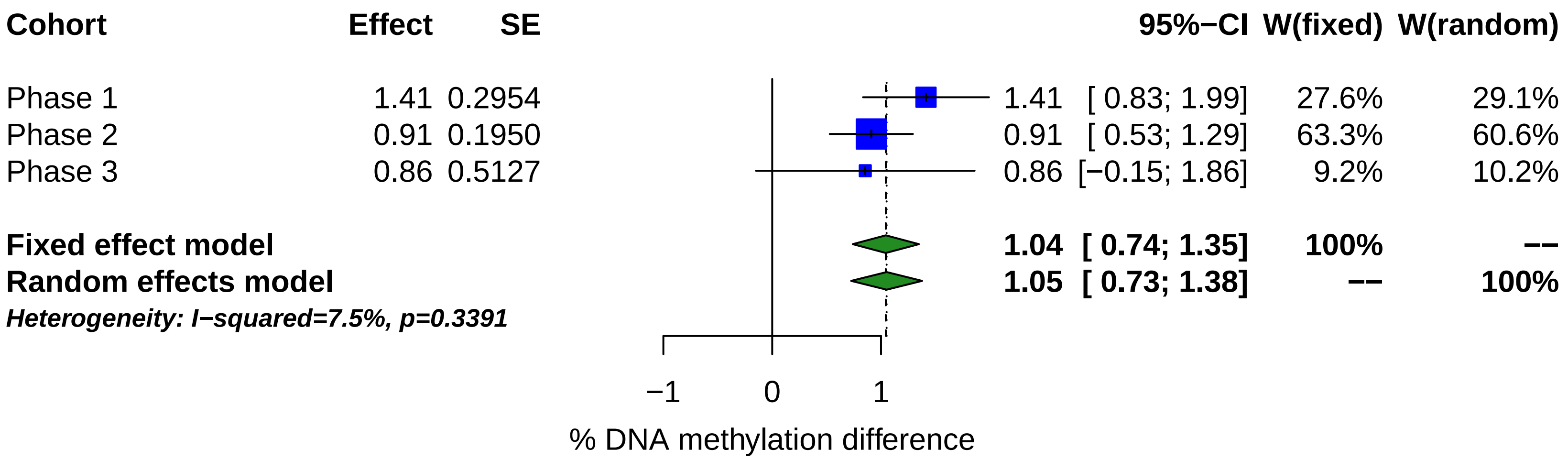

Cohort
Effect
SE
95%−CI
W(fixed)
W(random)
Phase 1 1.41 0.2954 1.41 [0.83; 1.99] 27.6% 29.1%
Phase 2 0.91 0.1950 0.91 [0.53; 1.29] 63.3% 60.6%
Phase 3 0.86 0.5127 0.86 [−0.15; 1.86] 9.2% 10.2%
Fixed effect model 1.04 [0.74; 1.35] 100% −−
Random effects model 1.05 [0.73; 1.38] −− 100%
Heterogeneity: I−squared=7.5%, p=0.3391
−1
0
1
% DNA methylation difference

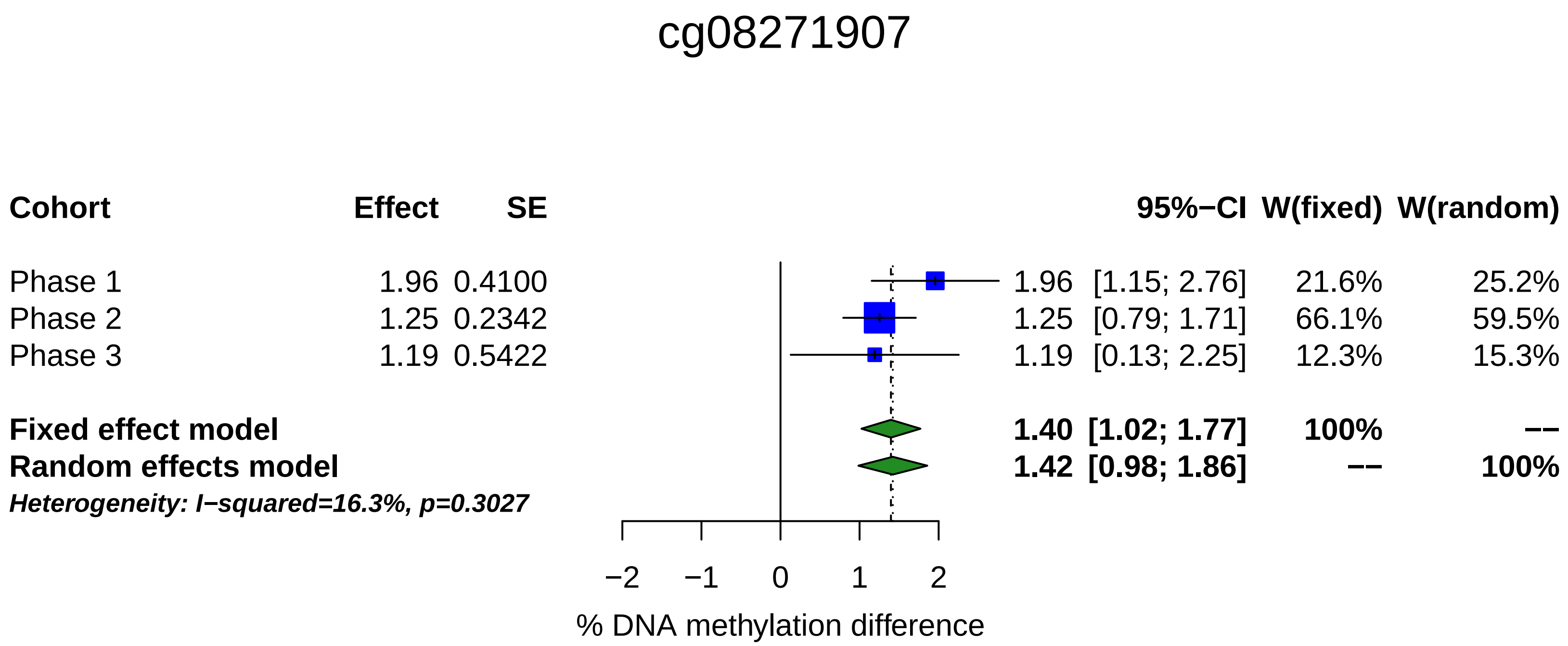
cg08271907
Cohort
Effect
SE
95%−CI
W(fixed)
W(random)
Phase 1 1.96 0.4100 1.96 [1.15; 2.76] 21.6% 25.2%
Phase 2 1.25 0.2342 1.25 [0.79; 1.71] 66.1% 59.5%
Phase 3 1.19 0.5422 1.19 [0.13; 2.25] 12.3% 15.3%
Fixed effect model 1.40 [1.02; 1.77] 100% −−
Random effects model 1.42 [0.98; 1.86] −− 100%
Heterogeneity: I−squared=16.3%, p=0.3027
−2
−1
0
1
2
% DNA methylation difference

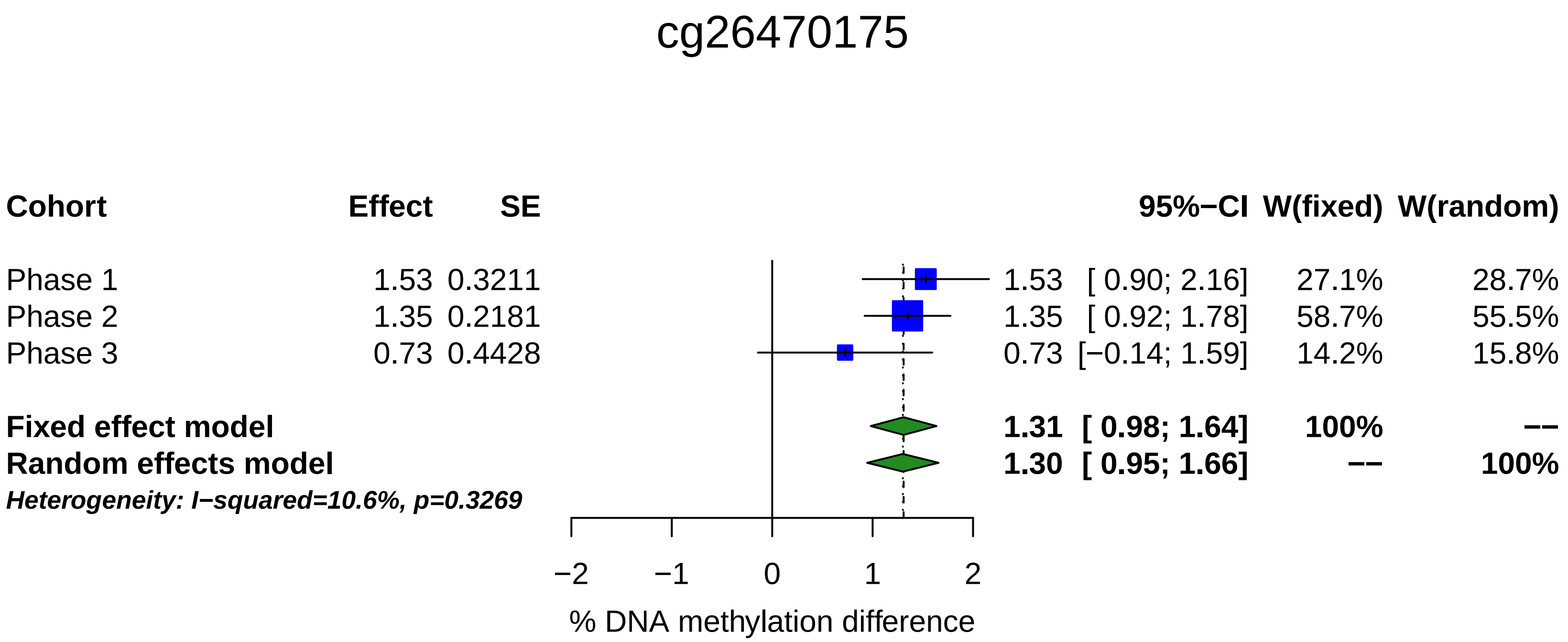
cg26470175
Cohort
Effect
SE
95%−CI
W(fixed)
W(random)
Phase 1 1.53 0.3211 1.53 [0.90; 2.16] 27.1% 28.7%
Phase 2 1.35 0.2181 1.35 [0.92; 1.78] 58.7% 55.5%
Phase 3 0.73 0.4428 0.73 [−0.14; 1.59] 14.2% 15.8%
Fixed effect model 1.31 [0.98; 1.64] 100% −−
Random effects model 1.30 [0.95; 1.66] −− 100%
Heterogeneity: I−squared=10.6%, p=0.3269
−2
−1
0
1
2
% DNA methylation difference

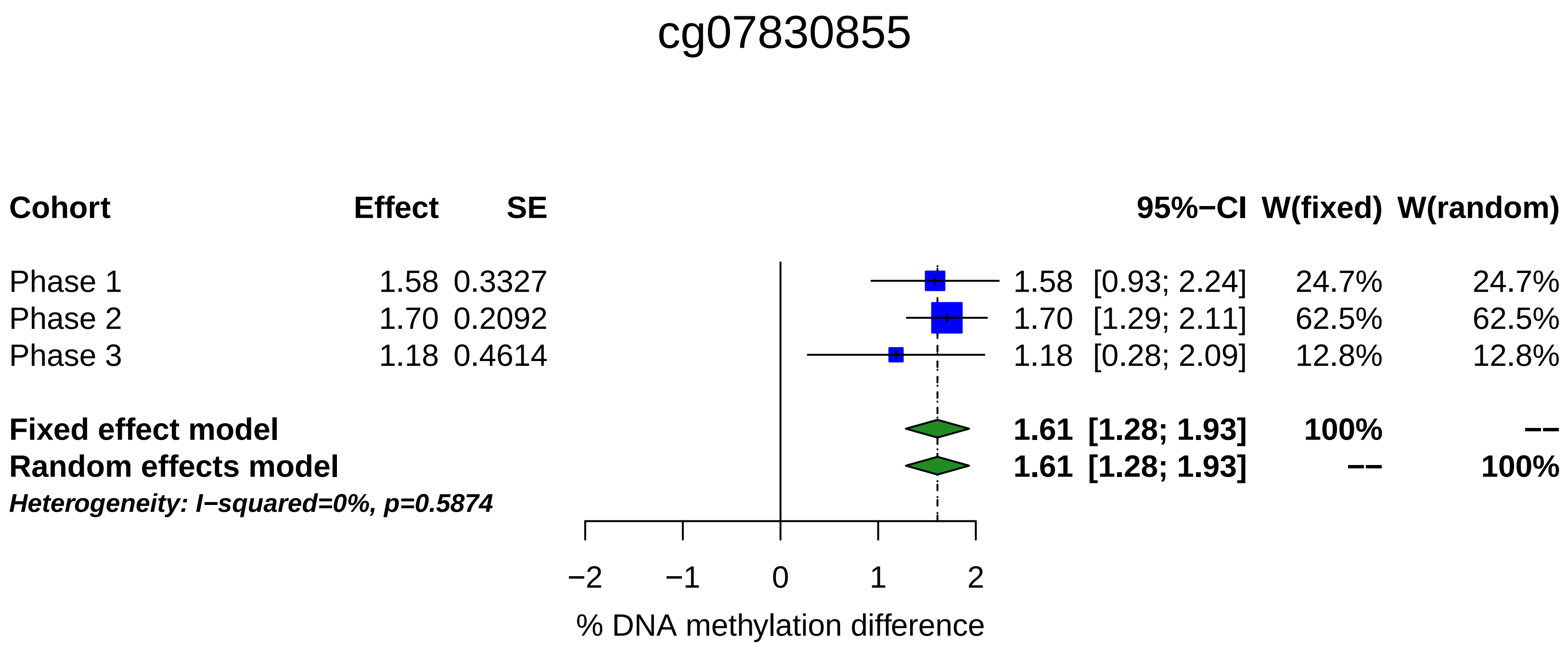
cg07830855
Cohort
Effect
SE
95%−CI
W(fixed)
W(random)
Phase 1 1.58 0.3327 1.58 [0.93; 2.24] 24.7% 24.7%
Phase 2 1.70 0.2092 1.70 [1.29; 2.11] 62.5% 62.5%
Phase 3 1.18 0.4614 1.18 [0.28; 2.09] 12.8% 12.8%
Fixed effect model 1.61 [1.28; 1.93] 100% −−
Random effects model 1.61 [1.28; 1.93] −− 100%
Heterogeneity: I−squared=0%, p=0.5874
−2
−1
0
1
2
% DNA methylation difference

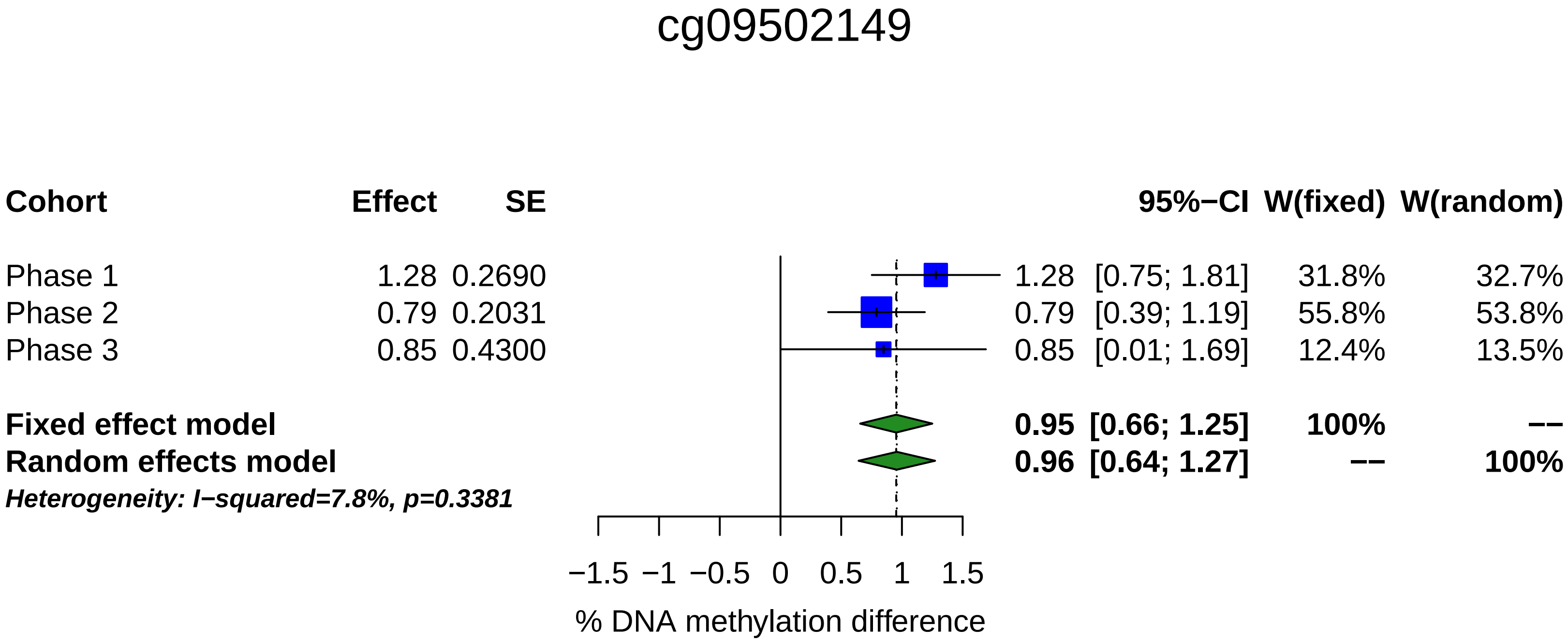
cg09502149
Cohort
Effect
SE
95%−CI
W(fixed)
W(random)
Phase 1 1.28 0.2690 1.28 [0.75; 1.81] 31.8% 32.7%
Phase 2 0.79 0.2031 0.79 [0.39; 1.19] 55.8% 53.8%
Phase 3 0.85 0.4300 0.85 [0.01; 1.69] 12.4% 13.5%
Fixed effect model 0.95 [0.66; 1.25] 100% −−
Random effects model 0.96 [0.64; 1.27] −− 100%
Heterogeneity: I−squared=7.8%, p=0.3381
−1.5 −1 −0.5 0 0.5 1 1.5
% DNA methylation difference

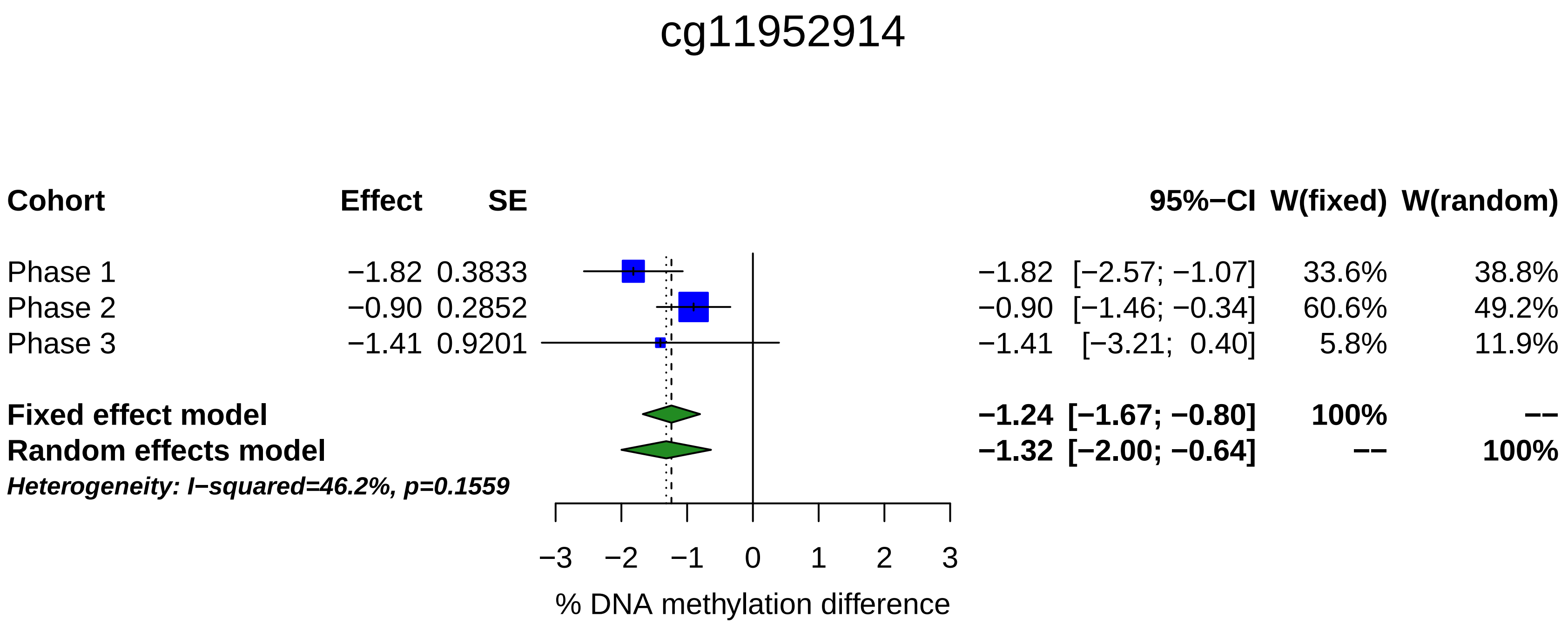
cg11952914
Cohort
Effect
SE
95%−CI
W(fixed)
W(random)
Phase 1 −1.82 0.3833 −1.82 [−2.57; −1.07] 33.6% 38.8%
Phase 2 −0.90 0.2852 −0.90 [−1.46; −0.34] 60.6% 49.2%
Phase 3 −1.41 0.9201 −1.41 [−3.21; 0.40] 5.8% 11.9%
Fixed effect model −1.24 [−1.67; −0.80] 100% −−
Random effects model −1.32 [−2.00; −0.64] −− 100%
Heterogeneity: I−squared=46.2%, p=0.1559
−3
−2
−1
0
1
2
3
% DNA methylation difference

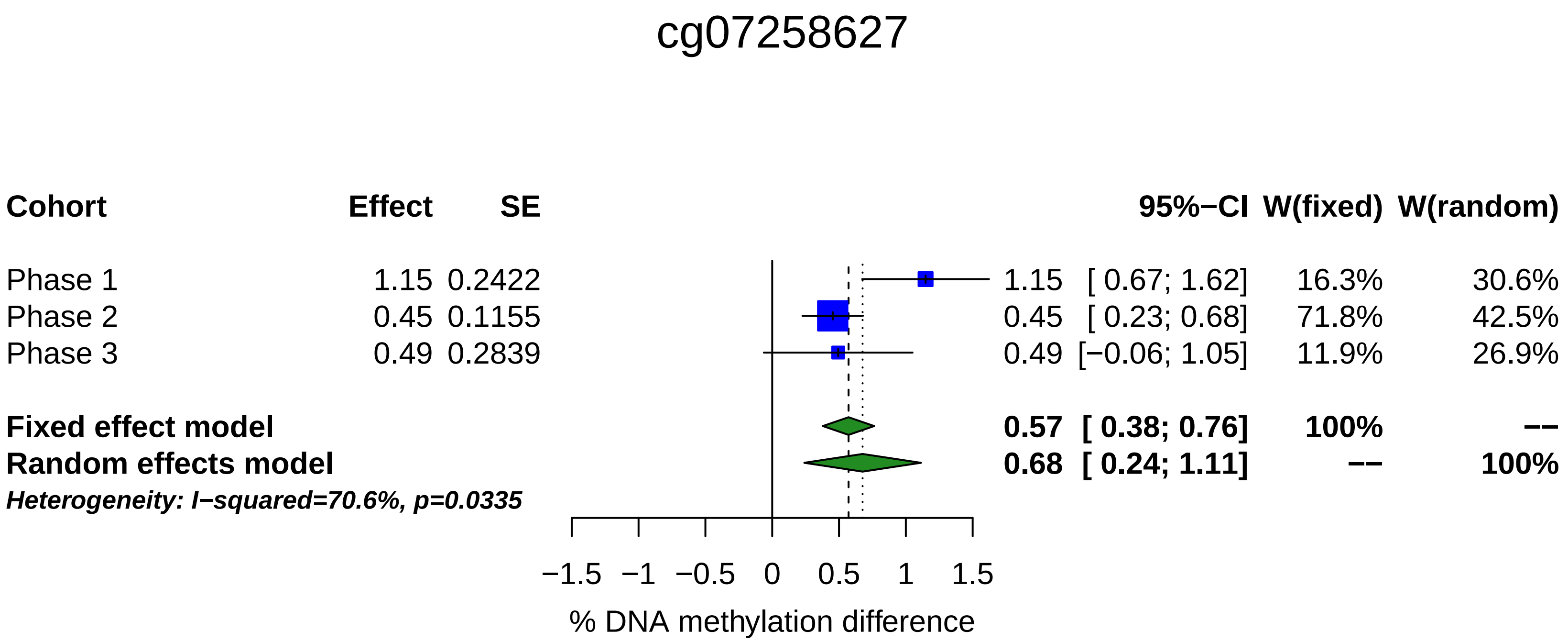
cg07258627
Cohort
Effect
SE
95%−CI
W(fixed)
W(random)
Phase 1
1.15
0.2422
1.15 [0.67; 1.62]
16.3%
30.6%
Phase 2
0.45
0.1155
0.45 [0.23; 0.68]
71.8%
42.5%
Phase 3
0.49
0.2839
0.49 [−0.06; 1.05]
11.9%
26.9%
Fixed effect model
0.57 [0.38; 0.76]
100%
−−
Random effects model
0.68 [0.24; 1.11]
−−
100%
Heterogeneity: I−squared=70.6%, p=0.0335
−1.5
−1
−0.5
0
0.5
1
1.5
% DNA methylation difference

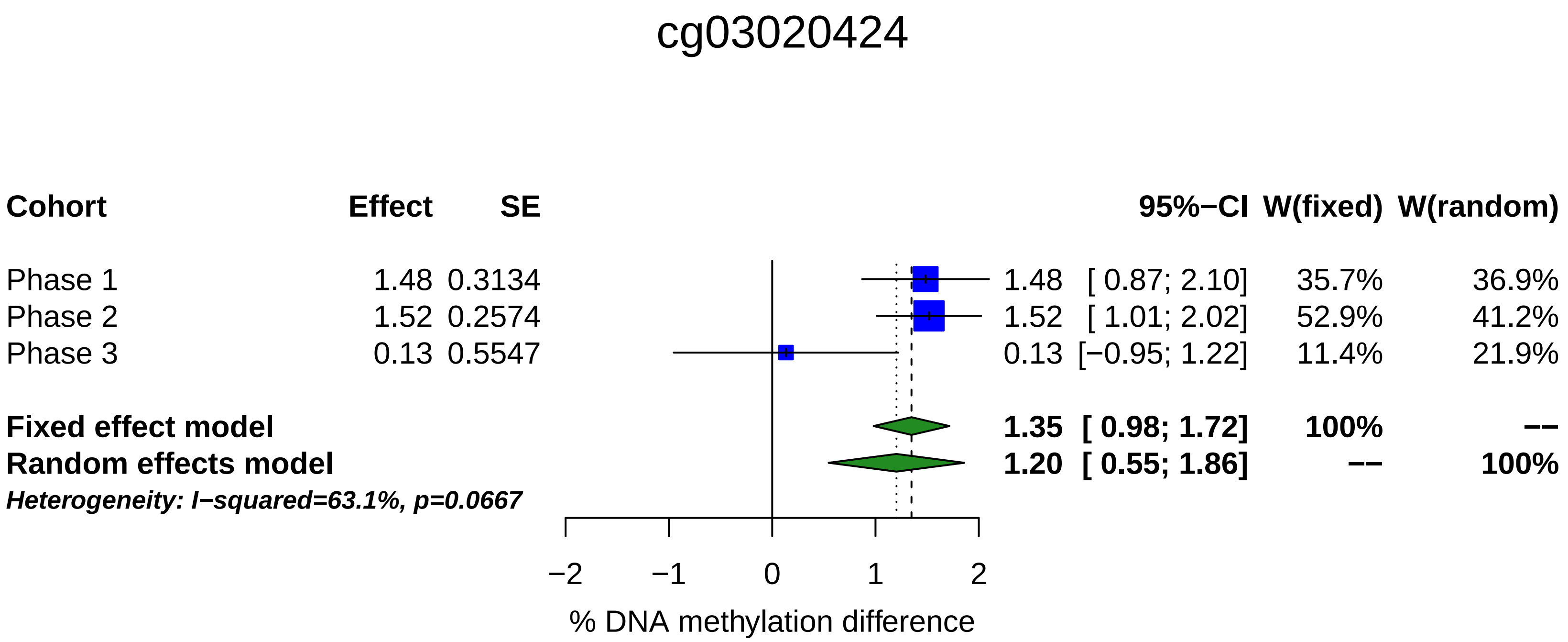
cg03020424
Cohort
Effect
SE
95%−CI
W(fixed)
W(random)
Phase 1 1.48 0.3134 1.48 [0.87; 2.10] 35.7% 36.9%
Phase 2 1.52 0.2574 1.52 [1.01; 2.02] 52.9% 41.2%
Phase 3 0.13 0.5547 0.13 [−0.95; 1.22] 11.4% 21.9%
Fixed effect model 1.35 [0.98; 1.72] 100% −−
Random effects model 1.20 [0.55; 1.86] −− 100%
Heterogeneity: I−squared=63.1%, p=0.0667
−2
−1
0
1
2
% DNA methylation difference

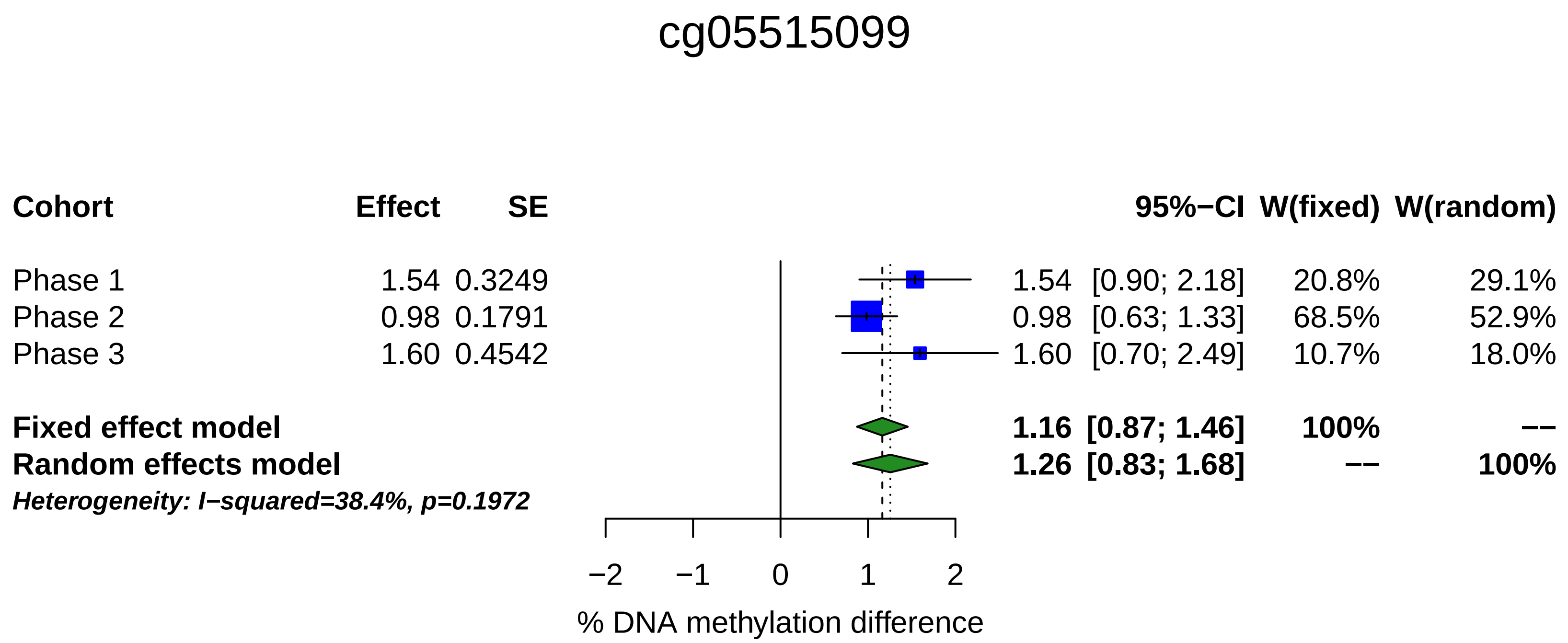
cg05515099
Cohort
Effect
SE
95%−CI
W(fixed)
W(random)
Phase 1
1.54 0.3249
1.54 [0.90; 2.18] 20.8% 29.1%
Phase 2
0.98 0.1791
0.98 [0.63; 1.33] 68.5% 52.9%
Phase 3
1.60 0.4542
1.60 [0.70; 2.49] 10.7% 18.0%
Fixed effect model
1.16 [0.87; 1.46] 100% −−
Random effects model
1.26 [0.83; 1.68] −− 100%
Heterogeneity: I−squared=38.4%, p=0.1972
−2
−1
0
1
2
% DNA methylation difference

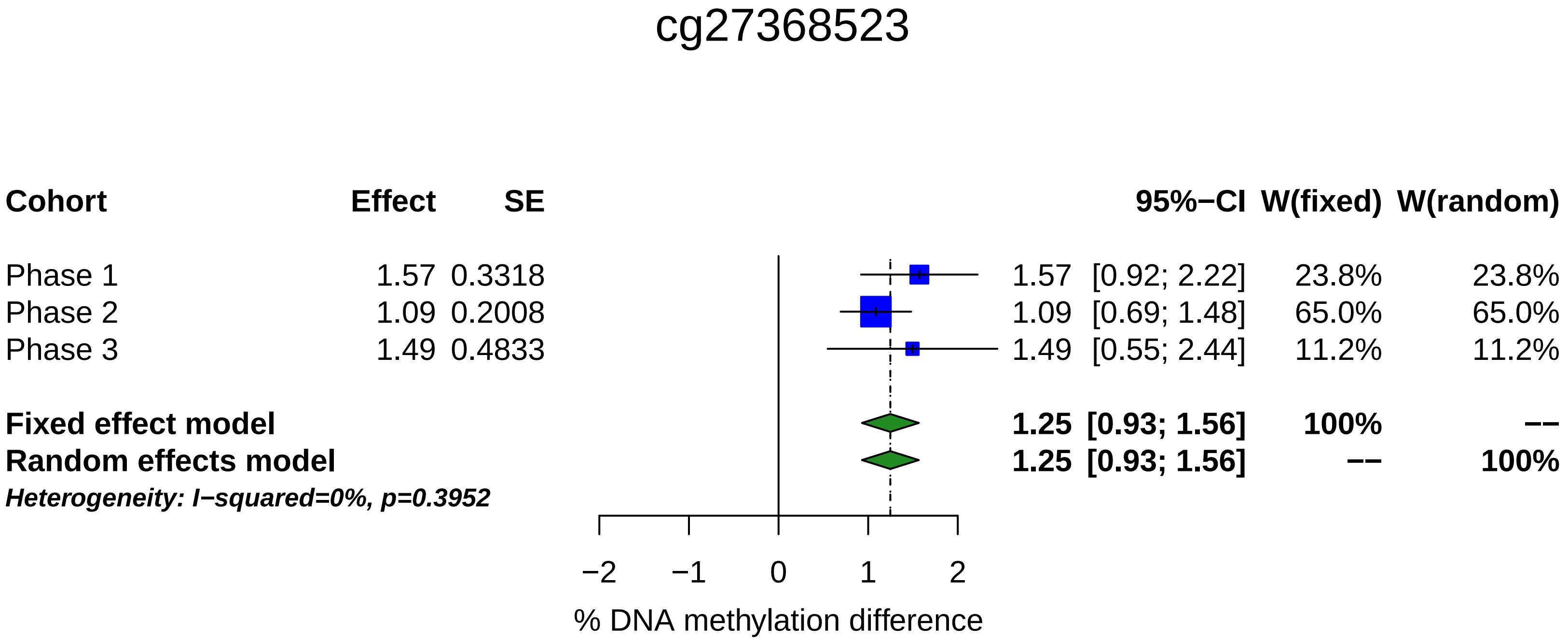
cg27368523
Cohort
Effect
SE
95%−CI
W(fixed)
W(random)
Phase 1
1.57
0.3318
1.57 [0.92; 2.22]
23.8%
23.8%
Phase 2
1.09
0.2008
1.09 [0.69; 1.48]
65.0%
65.0%
Phase 3
1.49
0.4833
1.49 [0.55; 2.44]
11.2%
11.2%
Fixed effect model
1.25 [0.93; 1.56]
100%
−−
Random effects model
1.25 [0.93; 1.56]
−−
100%
Heterogeneity: I−squared=0%, p=0.3952
−2
−1
0
1
2
% DNA methylation difference

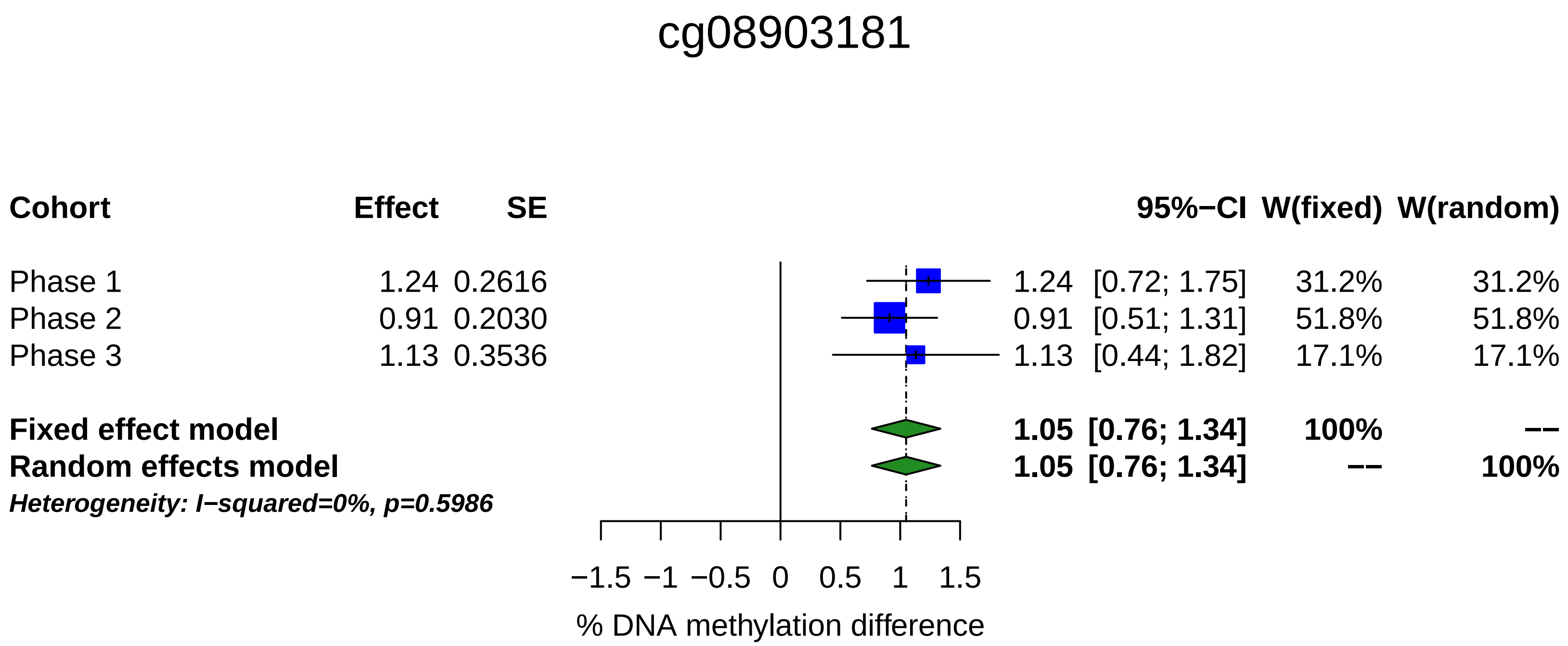
cg08903181
Cohort
Effect
SE
95%−CI
W(fixed)
W(random)
Phase 1 1.24 0.2616 1.24 [0.72; 1.75] 31.2% 31.2%
Phase 2 0.91 0.2030 0.91 [0.51; 1.31] 51.8% 51.8%
Phase 3 1.13 0.3536 1.13 [0.44; 1.82] 17.1% 17.1%
Fixed effect model 1.05 [0.76; 1.34] 100% −−
Random effects model 1.05 [0.76; 1.34] −− 100%
Heterogeneity: I−squared=0%, p=0.5986
−1.5 −1 −0.5 0 0.5 1 1.5
% DNA methylation difference

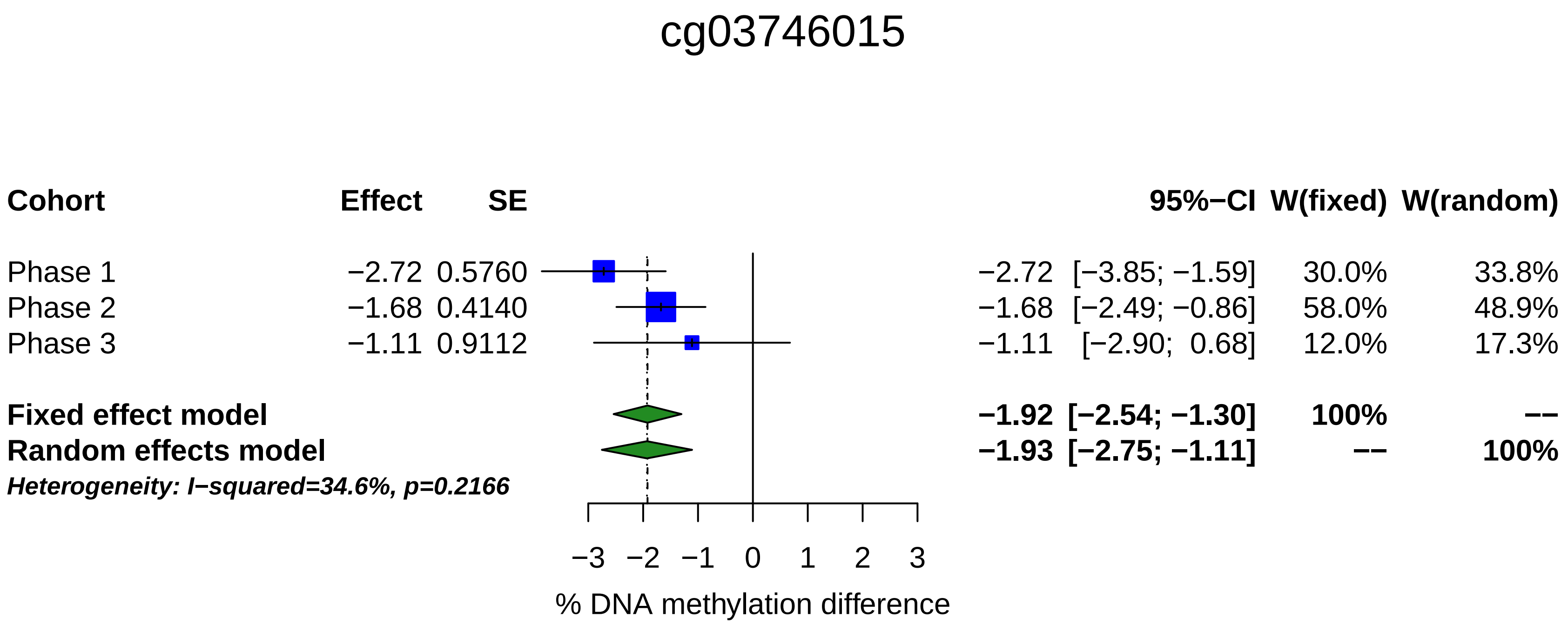
cg03746015
Cohort
Effect
SE
95%−CI
W(fixed)
W(random)
Phase 1 −2.72 0.5760 −2.72 [−3.85; −1.59] 30.0% 33.8%
Phase 2 −1.68 0.4140 −1.68 [−2.49; −0.86] 58.0% 48.9%
Phase 3 −1.11 0.9112 −1.11 [−2.90; 0.68] 12.0% 17.3%
Fixed effect model −1.92 [−2.54; −1.30] 100% −−
Random effects model −1.93 [−2.75; −1.11] −− 100%
Heterogeneity: I−squared=34.6%, p=0.2166
−3 −2 −1 0 1 2 3
% DNA methylation difference

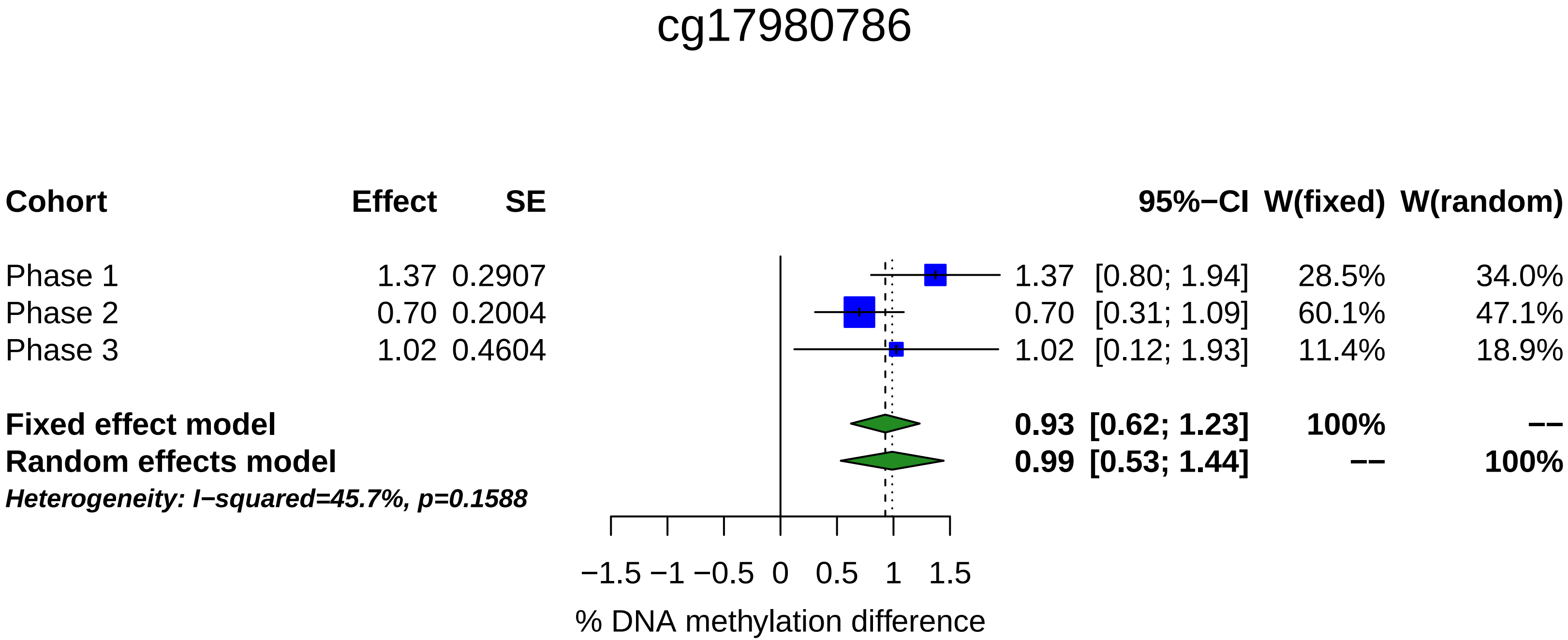
cg17980786
Cohort
Effect
SE
95%−CI
W(fixed)
W(random)
Phase 1 1.37 0.2907 1.37 [0.80; 1.94] 28.5% 34.0%
Phase 2 0.70 0.2004 0.70 [0.31; 1.09] 60.1% 47.1%
Phase 3 1.02 0.4604 1.02 [0.12; 1.93] 11.4% 18.9%
Fixed effect model 0.93 [0.62; 1.23] 100% −−
Random effects model 0.99 [0.53; 1.44] −− 100%
Heterogeneity: I−squared=45.7%, p=0.1588
−1.5 −1 −0.5 0 0.5 1 1.5
% DNA methylation difference

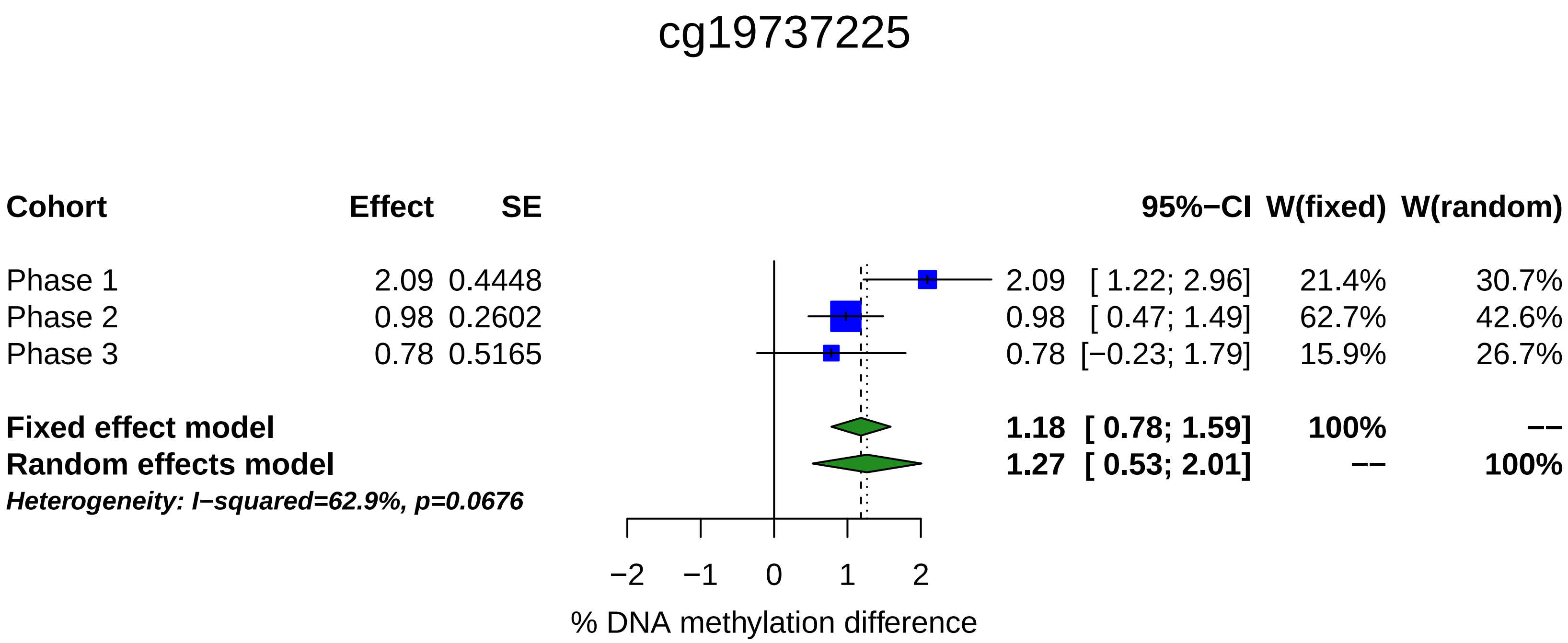
cg19737225
Cohort
Effect
SE
95%−CI
W(fixed)
W(random)
Phase 1 2.09 0.4448 2.09 [1.22; 2.96] 21.4% 30.7%
Phase 2 0.98 0.2602 0.98 [0.47; 1.49] 62.7% 42.6%
Phase 3 0.78 0.5165 0.78 [−0.23; 1.79] 15.9% 26.7%
Fixed effect model 1.18 [0.78; 1.59] 100% −−
Random effects model 1.27 [0.53; 2.01] −− 100%
Heterogeneity: I−squared=62.9%, p=0.0676
−2
−1
0
1
2
% DNA methylation difference

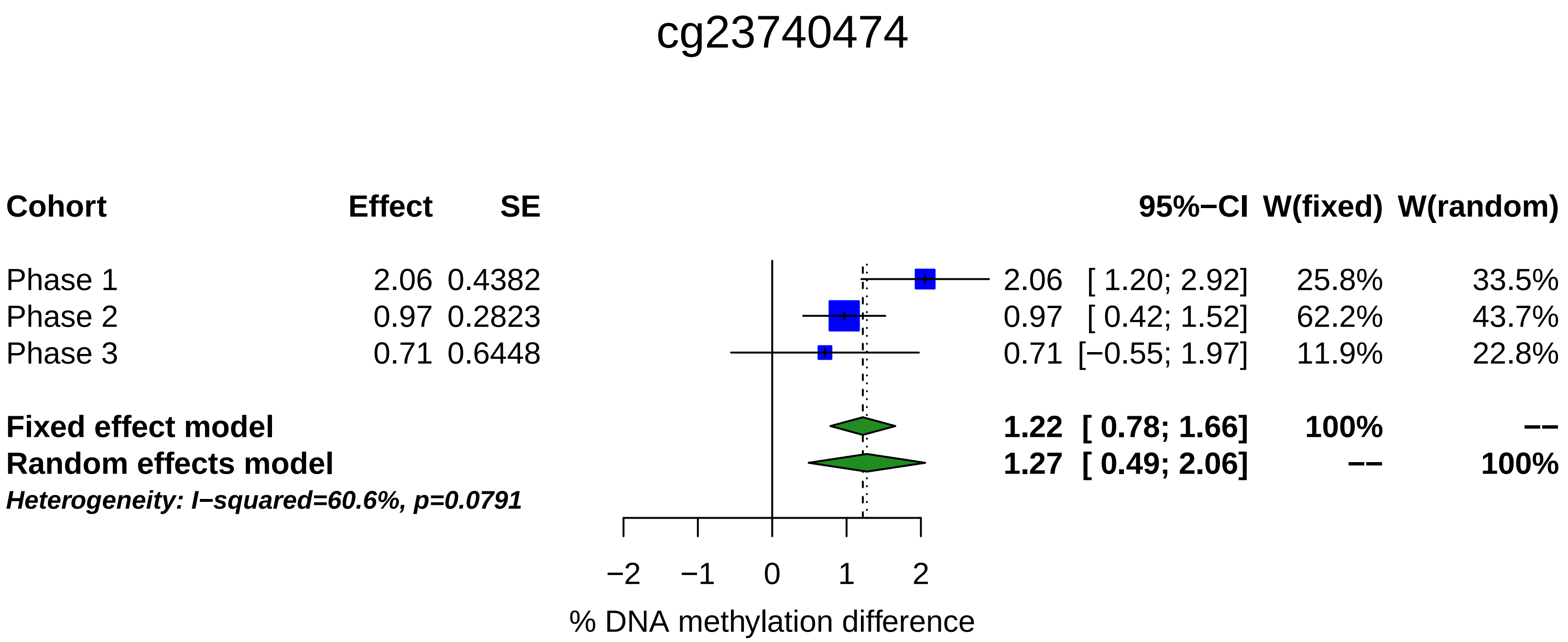
cg23740474
Cohort
Effect
SE
95%−CI
W(fixed)
W(random)
Phase 1 2.06 0.4382 2.06 [1.20; 2.92] 25.8% 33.5%
Phase 2 0.97 0.2823 0.97 [0.42; 1.52] 62.2% 43.7%
Phase 3 0.71 0.6448 0.71 [−0.55; 1.97] 11.9% 22.8%
Fixed effect model 1.22 [0.78; 1.66] 100% −−
Random effects model 1.27 [0.49; 2.06] −− 100%
Heterogeneity: I−squared=60.6%, p=0.0791
−2
−1
0
1
2
% DNA methylation difference

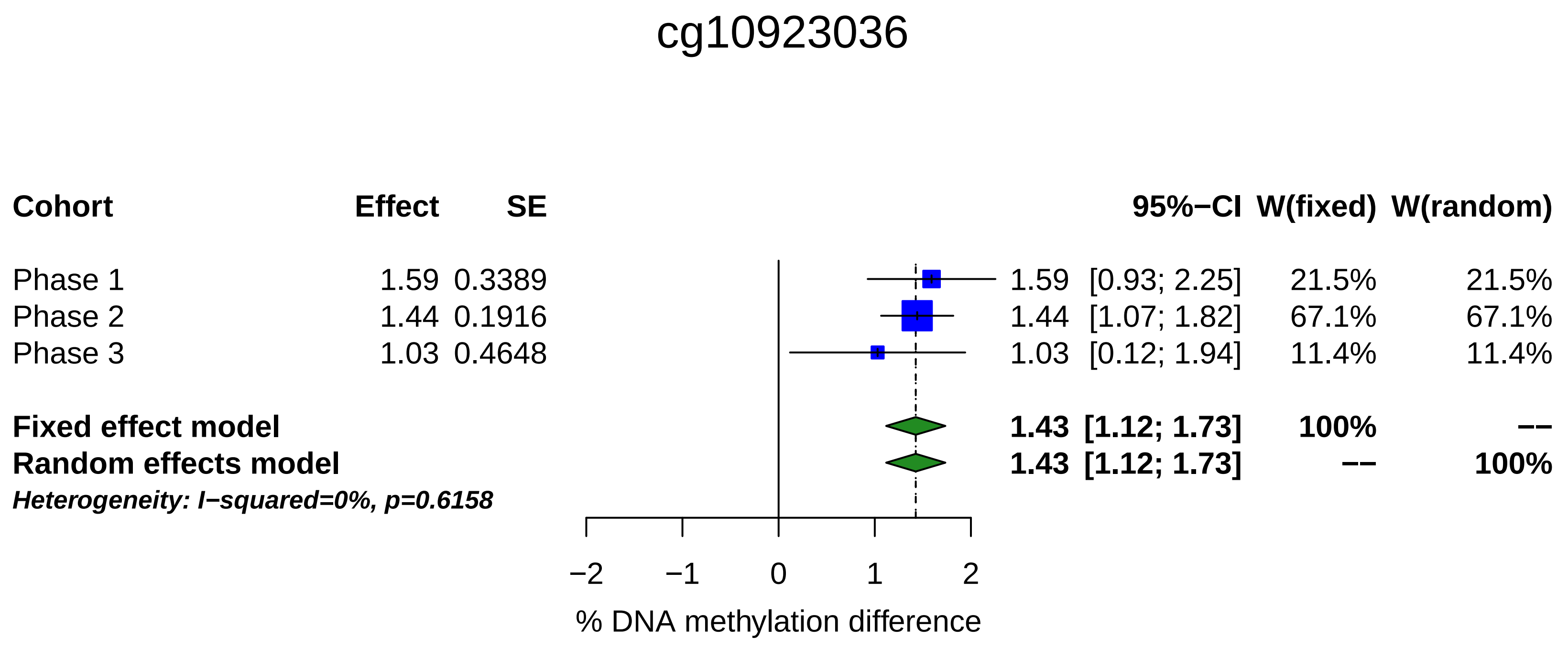

cg10923036
Cohort
Effect
SE
95%−CI
W(fixed)
W(random)
Phase 1 1.59 0.3389 1.59 [0.93; 2.25] 21.5% 21.5%
Phase 2 1.44 0.1916 1.44 [1.07; 1.82] 67.1% 67.1%
Phase 3 1.03 0.4648 1.03 [0.12; 1.94] 11.4% 11.4%
Fixed effect model 1.43 [1.12; 1.73] 100% −−
Random effects model 1.43 [1.12; 1.73] −− 100%
Heterogeneity: I−squared=0%, p=0.6158
−2
−1
0
1
2
% DNA methylation difference

cg04819337

Cohort	Effect	SE	95%−CI	W(fixed)	W(random)
Phase 1	1.35	0.2874	1.35 [0.78; 1.91]	30.9%	34.9%
Phase 2	0.88	0.2126	0.88 [0.46; 1.30]	56.5%	38.9%
Phase 3	−0.18	0.4495	−0.18 [−1.06; 0.71]	12.6%	26.2%
**Fixed effect model**			**0.89 [0.58; 1.20]**	**100%**	**−−**
**Random effects model**			**0.77 [0.07; 1.46]**	**−−**	**100%**

Heterogeneity: I−squared=75.5%, p=0.0169

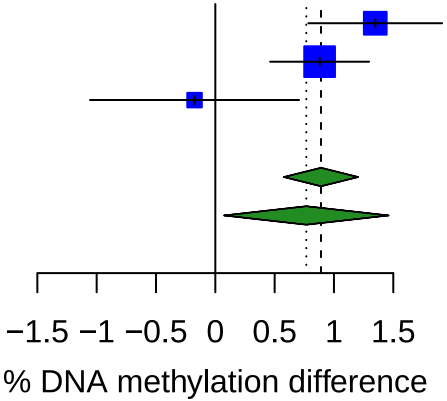

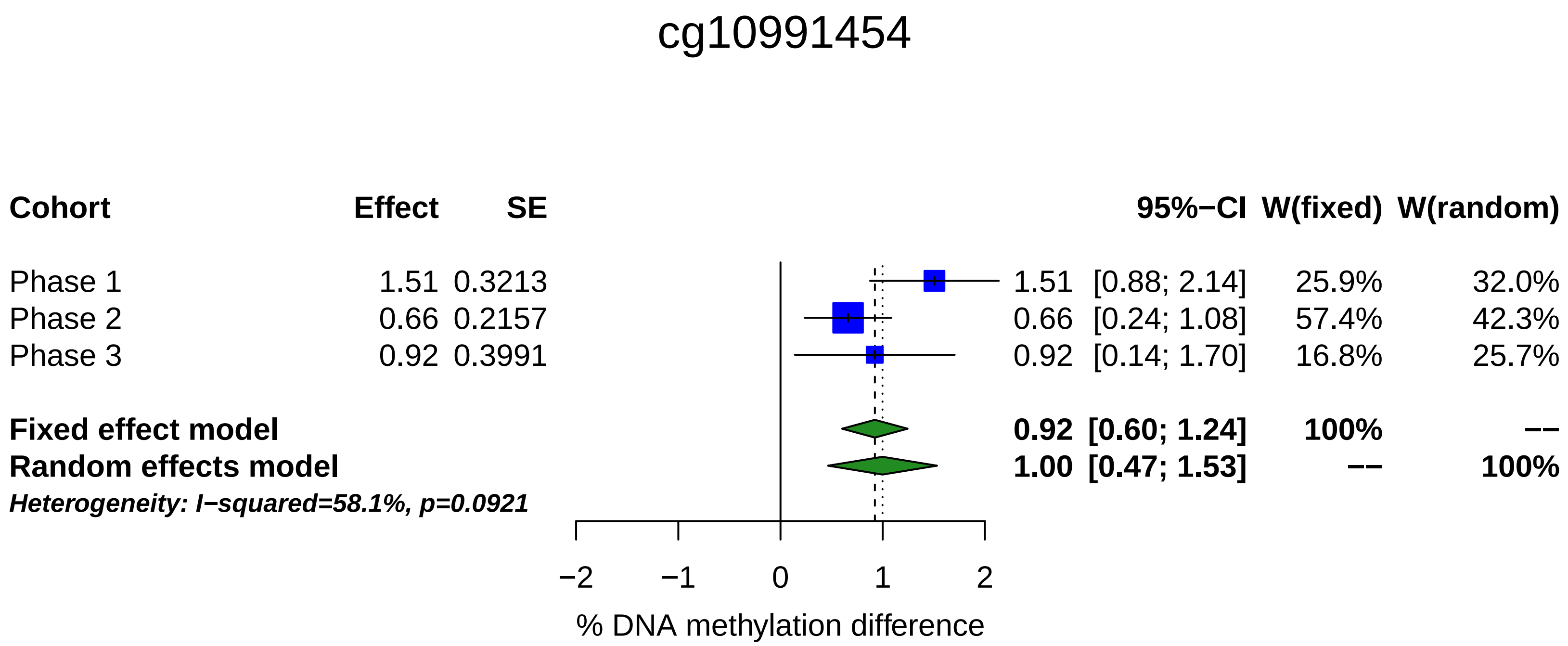
cg10991454
Cohort
Effect
SE
95%−CI
W(fixed)
W(random)
Phase 1 1.51 0.3213 1.51 [0.88; 2.14] 25.9% 32.0%
Phase 2 0.66 0.2157 0.66 [0.24; 1.08] 57.4% 42.3%
Phase 3 0.92 0.3991 0.92 [0.14; 1.70] 16.8% 25.7%
Fixed effect model 0.92 [0.60; 1.24] 100% −−
Random effects model 1.00 [0.47; 1.53] −− 100%
Heterogeneity: I−squared=58.1%, p=0.0921
−2
−1
0
1
2
% DNA methylation difference

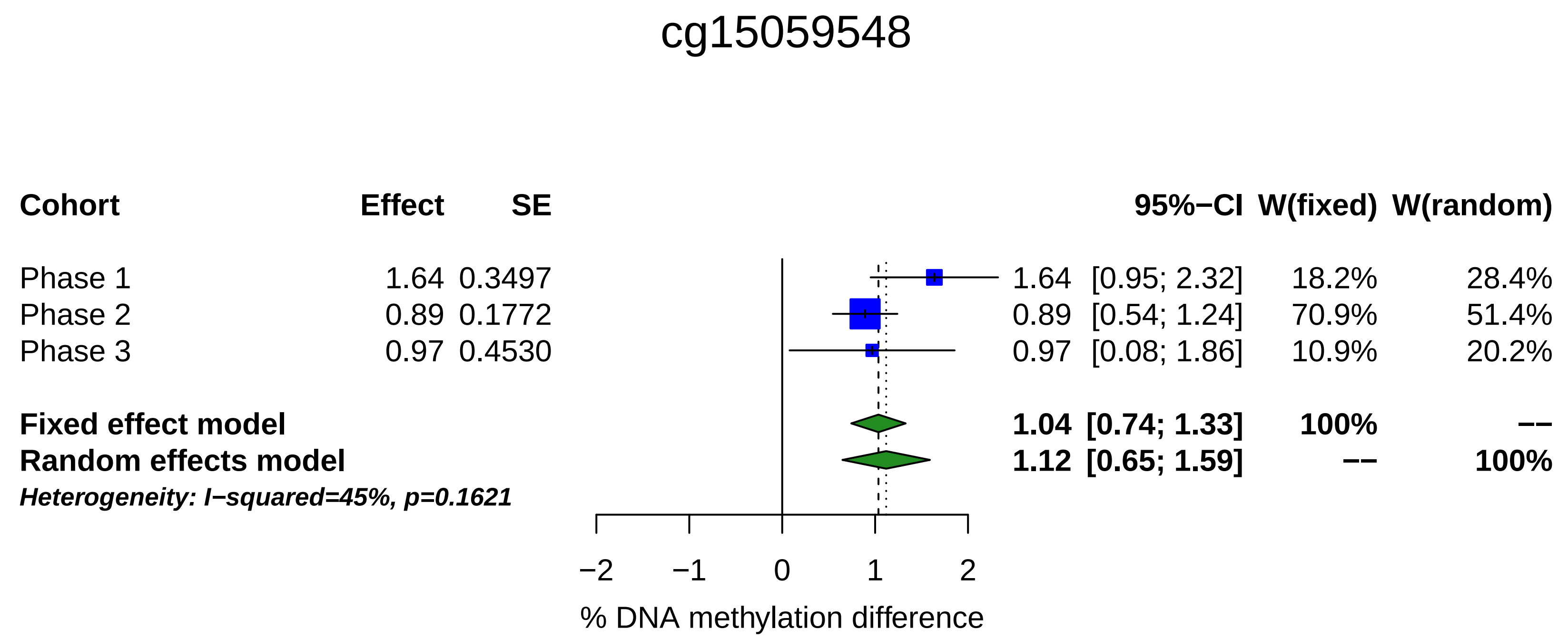
cg15059548
Cohort
Effect
SE
95%−CI
W(fixed)
W(random)
Phase 1 1.64 0.3497 1.64 [0.95; 2.32] 18.2% 28.4%
Phase 2 0.89 0.1772 0.89 [0.54; 1.24] 70.9% 51.4%
Phase 3 0.97 0.4530 0.97 [0.08; 1.86] 10.9% 20.2%
Fixed effect model 1.04 [0.74; 1.33] 100% −−
Random effects model 1.12 [0.65; 1.59] −− 100%
Heterogeneity: I−squared=45%, p=0.1621
−2
−1
0
1
2
% DNA methylation difference

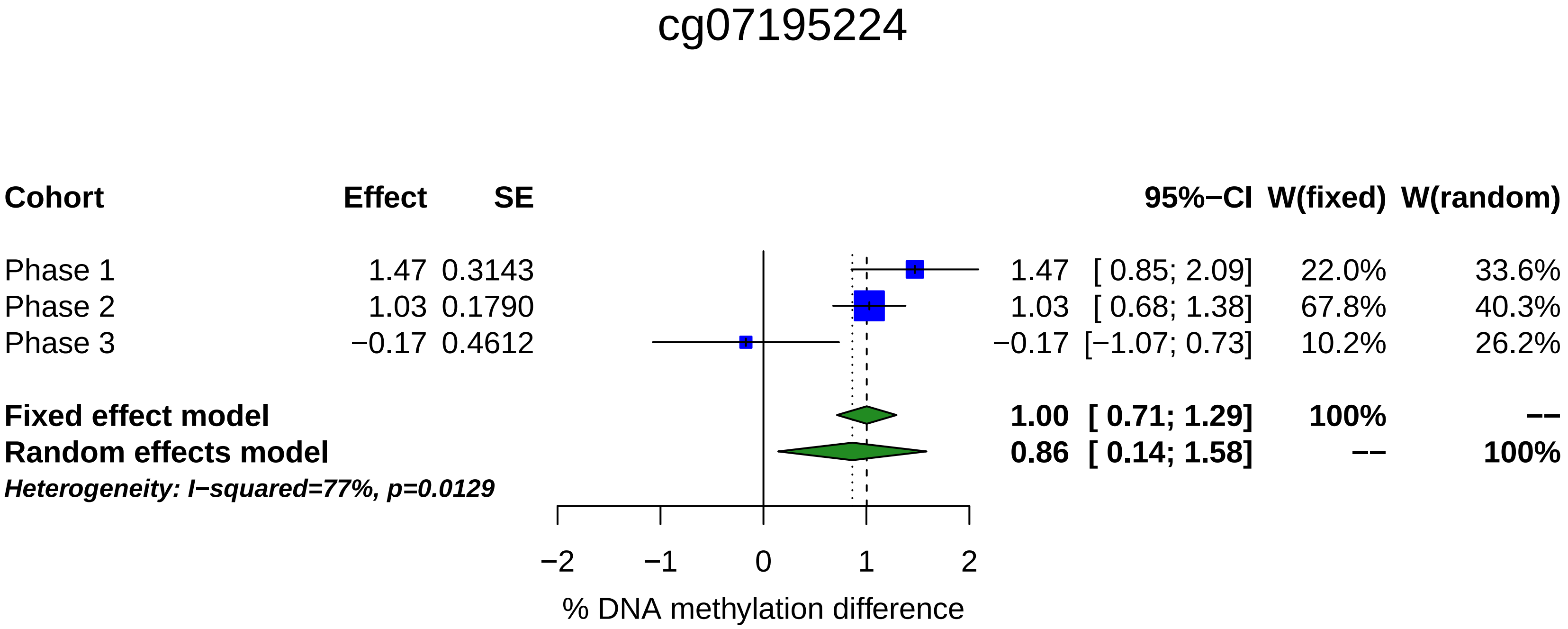
cg07195224
Cohort
Effect
SE
95%−CI
W(fixed)
W(random)
Phase 1
1.47
0.3143
1.47
[0.85; 2.09]
22.0%
33.6%
Phase 2
1.03
0.1790
1.03
[0.68; 1.38]
67.8%
40.3%
Phase 3
−0.17
0.4612
−0.17
[−1.07; 0.73]
10.2%
26.2%
Fixed effect model
1.00
[0.71; 1.29]
100%
−−
Random effects model
0.86
[0.14; 1.58]
−−
100%
Heterogeneity: I−squared=77%, p=0.0129
−2
−1
0
1
2
% DNA methylation difference

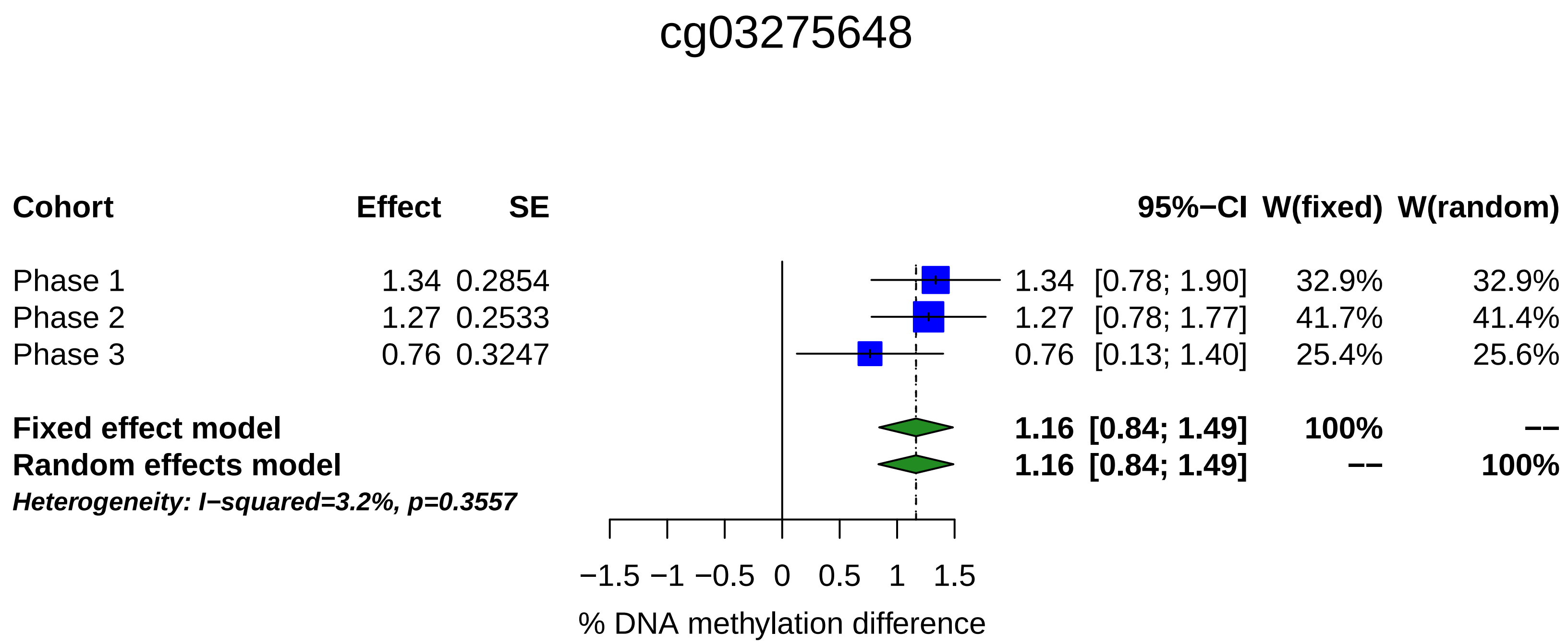
cg03275648
Cohort
Effect
SE
95%−CI
W(fixed)
W(random)
Phase 1 1.34 0.2854 1.34 [0.78; 1.90] 32.9% 32.9%
Phase 2 1.27 0.2533 1.27 [0.78; 1.77] 41.7% 41.4%
Phase 3 0.76 0.3247 0.76 [0.13; 1.40] 25.4% 25.6%
Fixed effect model 1.16 [0.84; 1.49] 100% −−
Random effects model 1.16 [0.84; 1.49] −− 100%
Heterogeneity: I−squared=3.2%, p=0.3557
−1.5 −1 −0.5 0 0.5 1 1.5
% DNA methylation difference

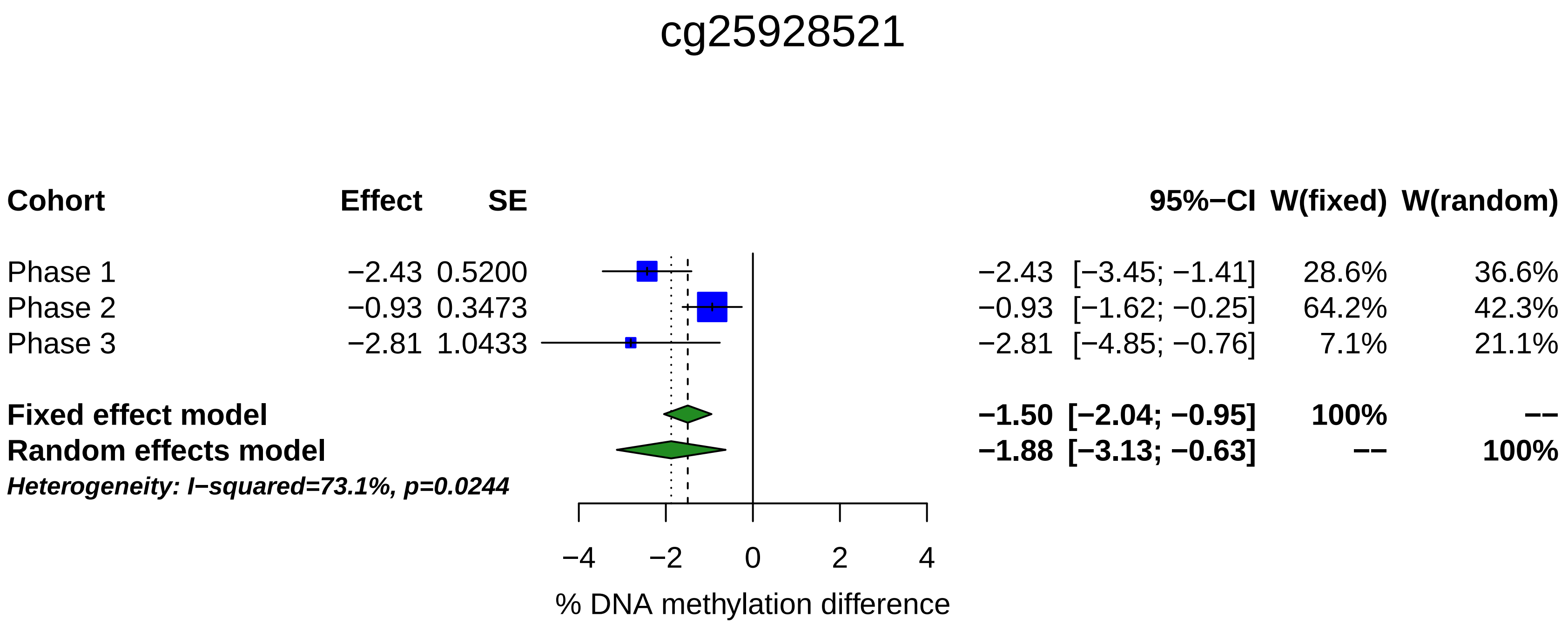
cg25928521
Cohort
Effect
SE
95%−CI
W(fixed)
W(random)
Phase 1 −2.43 0.5200 −2.43 [−3.45; −1.41] 28.6% 36.6%
Phase 2 −0.93 0.3473 −0.93 [−1.62; −0.25] 64.2% 42.3%
Phase 3 −2.81 1.0433 −2.81 [−4.85; −0.76] 7.1% 21.1%
Fixed effect model −1.50 [−2.04; −0.95] 100% −−
Random effects model −1.88 [−3.13; −0.63] −− 100%
Heterogeneity: I−squared=73.1%, p=0.0244
−4
−2
0
2
4
% DNA methylation difference

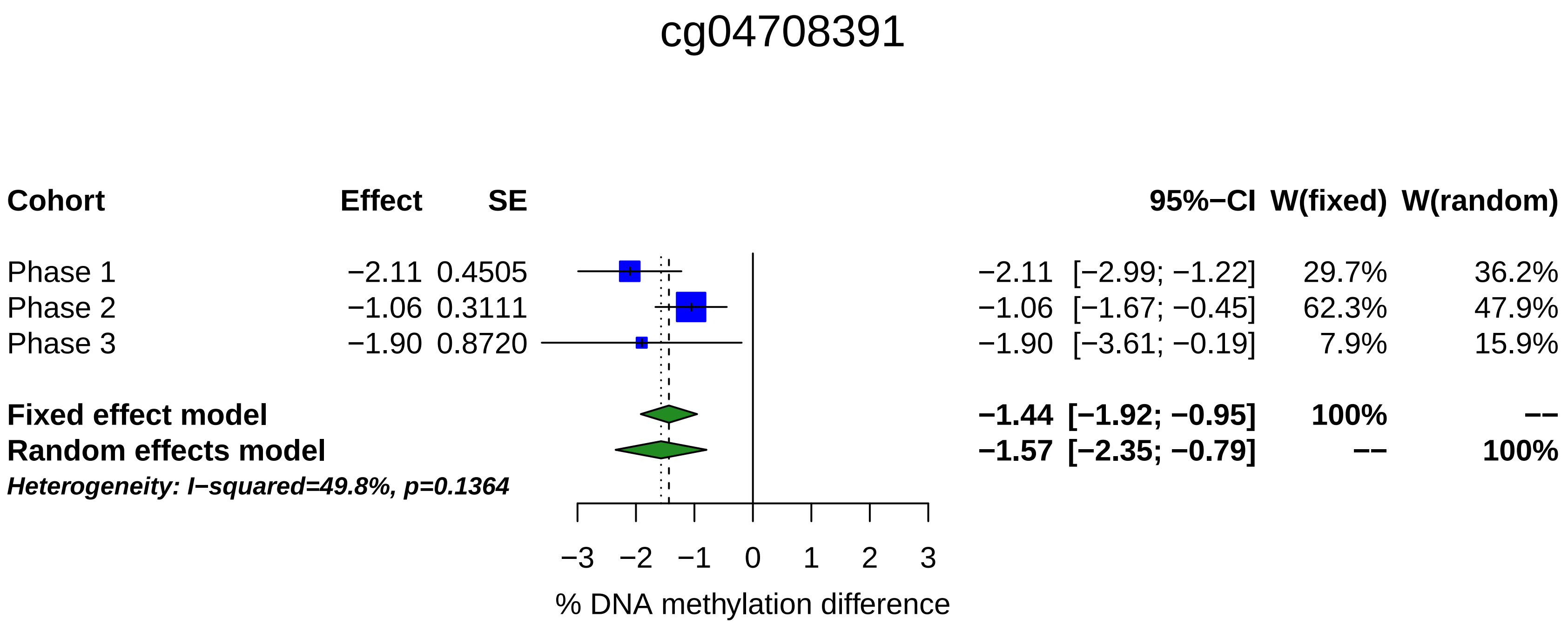
cg04708391
Cohort
Effect
SE
95%−CI
W(fixed)
W(random)
Phase 1 −2.11 0.4505 −2.11 [−2.99; −1.22] 29.7% 36.2%
Phase 2 −1.06 0.3111 −1.06 [−1.67; −0.45] 62.3% 47.9%
Phase 3 −1.90 0.8720 −1.90 [−3.61; −0.19] 7.9% 15.9%
Fixed effect model −1.44 [−1.92; −0.95] 100% −−
Random effects model −1.57 [−2.35; −0.79] −− 100%
Heterogeneity: I−squared=49.8%, p=0.1364
−3
−2
−1
0
1
2
3
% DNA methylation difference

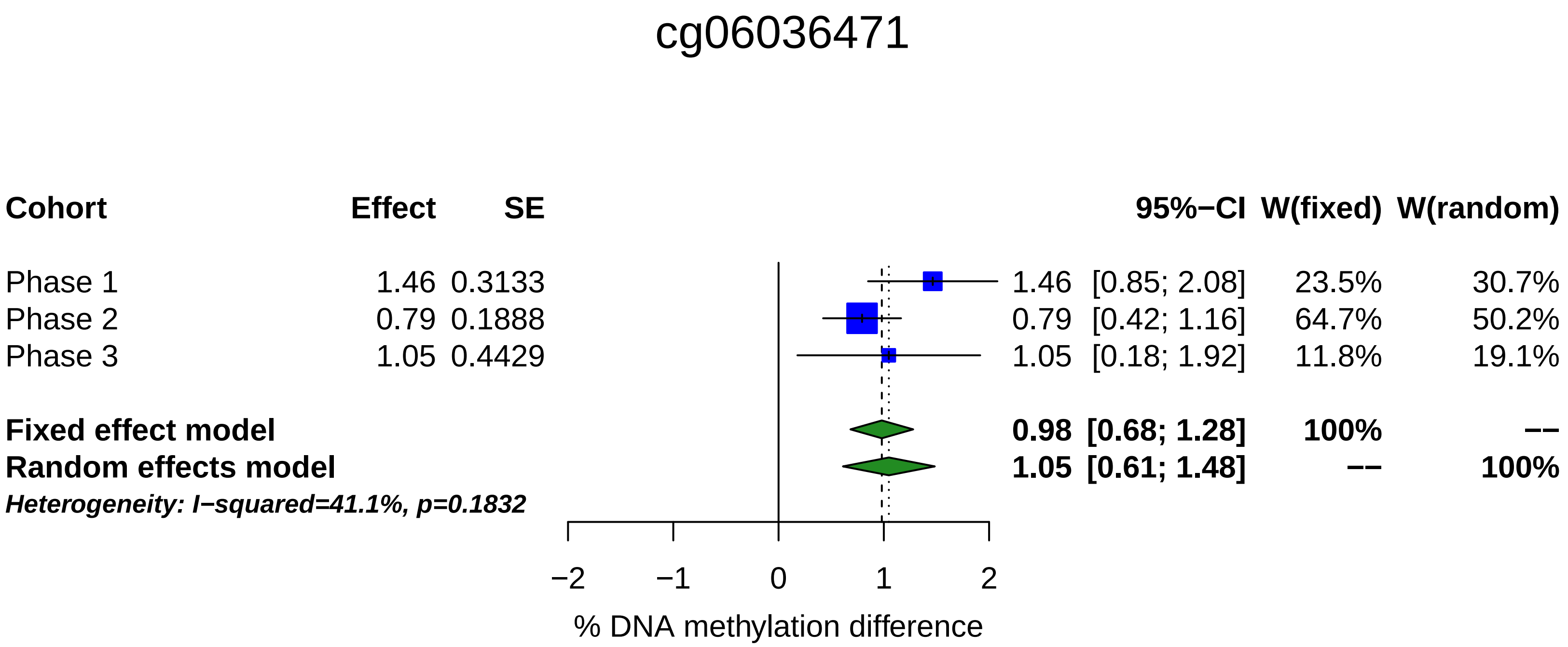
cg06036471
Cohort
Effect
SE
95%−CI
W(fixed)
W(random)
Phase 1 1.46 0.3133 1.46 [0.85; 2.08] 23.5% 30.7%
Phase 2 0.79 0.1888 0.79 [0.42; 1.16] 64.7% 50.2%
Phase 3 1.05 0.4429 1.05 [0.18; 1.92] 11.8% 19.1%
Fixed effect model 0.98 [0.68; 1.28] 100% −−
Random effects model 1.05 [0.61; 1.48] −− 100%
Heterogeneity: I−squared=41.1%, p=0.1832
−2
−1
0
1
2
% DNA methylation difference

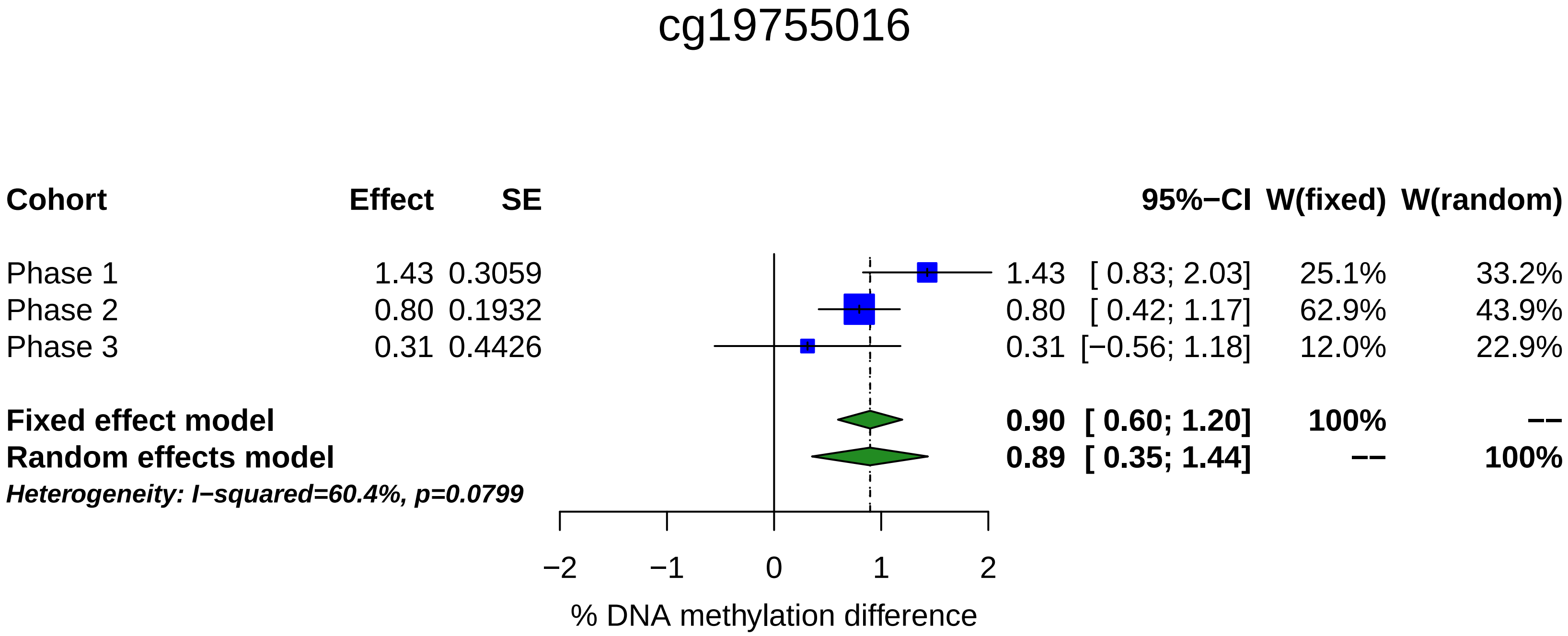
cg19755016
Cohort
Effect
SE
95%−CI
W(fixed)
W(random)
Phase 1 1.43 0.3059 1.43 [0.83; 2.03] 25.1% 33.2%
Phase 2 0.80 0.1932 0.80 [0.42; 1.17] 62.9% 43.9%
Phase 3 0.31 0.4426 0.31 [−0.56; 1.18] 12.0% 22.9%
Fixed effect model 0.90 [0.60; 1.20] 100% −−
Random effects model 0.89 [0.35; 1.44] −− 100%
Heterogeneity: I−squared=60.4%, p=0.0799
−2
−1
0
1
2
% DNA methylation difference

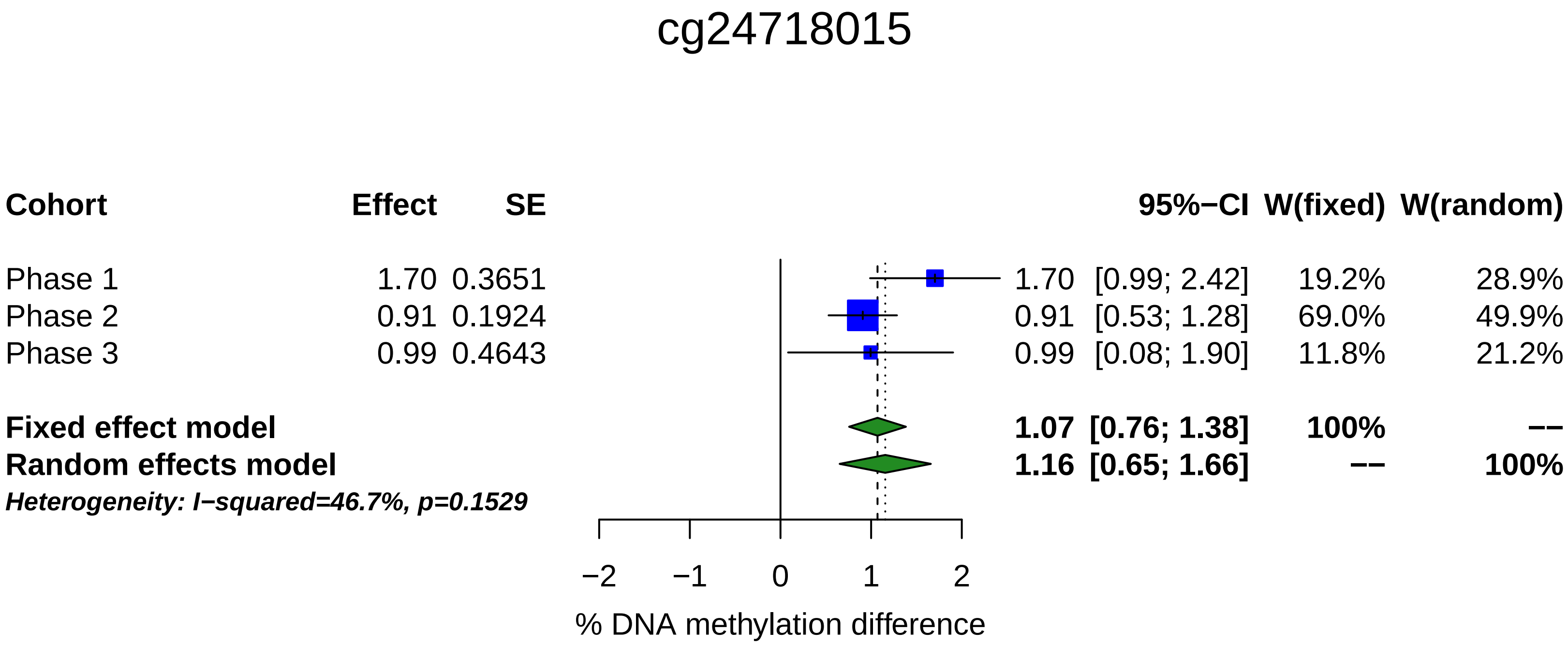
cg24718015
Cohort
Effect
SE
95%−CI
W(fixed)
W(random)
Phase 1 1.70 0.3651 1.70 [0.99; 2.42] 19.2% 28.9%
Phase 2 0.91 0.1924 0.91 [0.53; 1.28] 69.0% 49.9%
Phase 3 0.99 0.4643 0.99 [0.08; 1.90] 11.8% 21.2%
Fixed effect model 1.07 [0.76; 1.38] 100% −−
Random effects model 1.16 [0.65; 1.66] −− 100%
Heterogeneity: I−squared=46.7%, p=0.1529
−2
−1
0
1
2
% DNA methylation difference

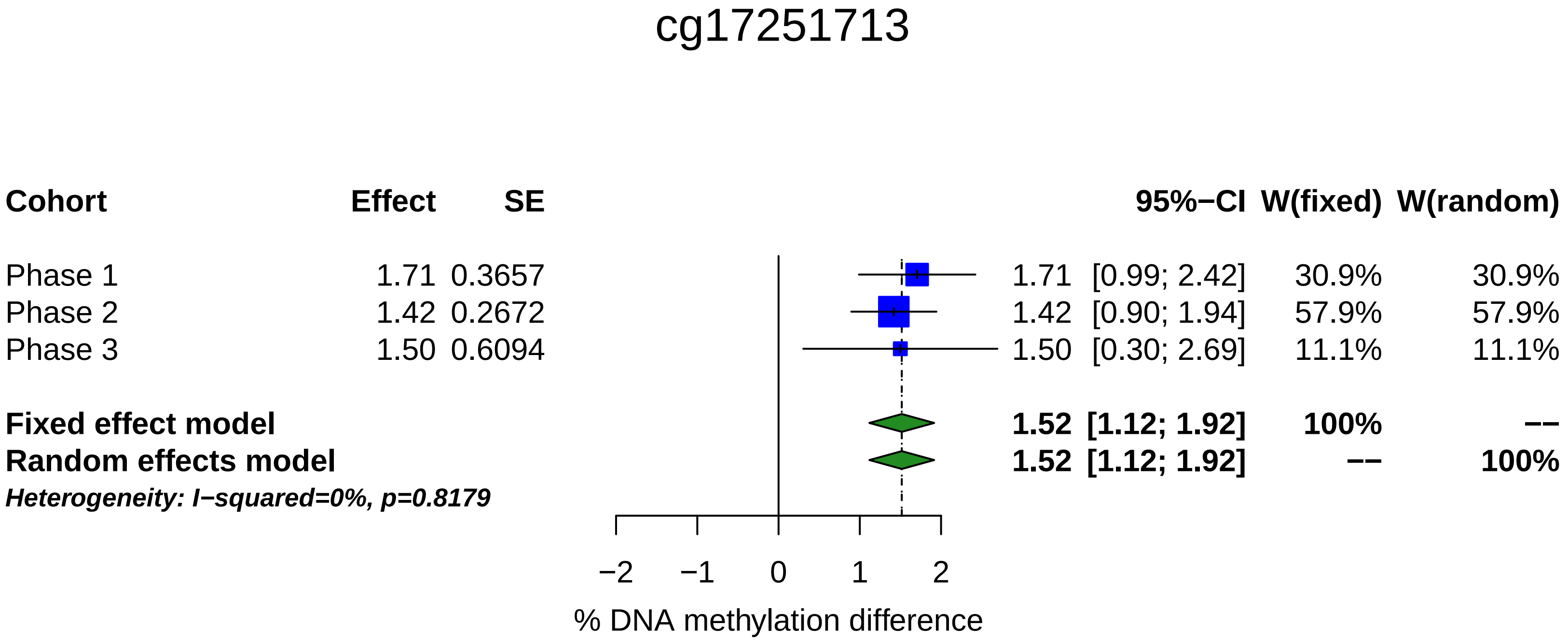
cg17251713
Cohort
Effect
SE
95%−CI
W(fixed)
W(random)
Phase 1 1.71 0.3657 1.71 [0.99; 2.42] 30.9% 30.9%
Phase 2 1.42 0.2672 1.42 [0.90; 1.94] 57.9% 57.9%
Phase 3 1.50 0.6094 1.50 [0.30; 2.69] 11.1% 11.1%
Fixed effect model 1.52 [1.12; 1.92] 100% −−
Random effects model 1.52 [1.12; 1.92] −− 100%
Heterogeneity: I−squared=0%, p=0.8179
−2
−1
0
1
2
% DNA methylation difference

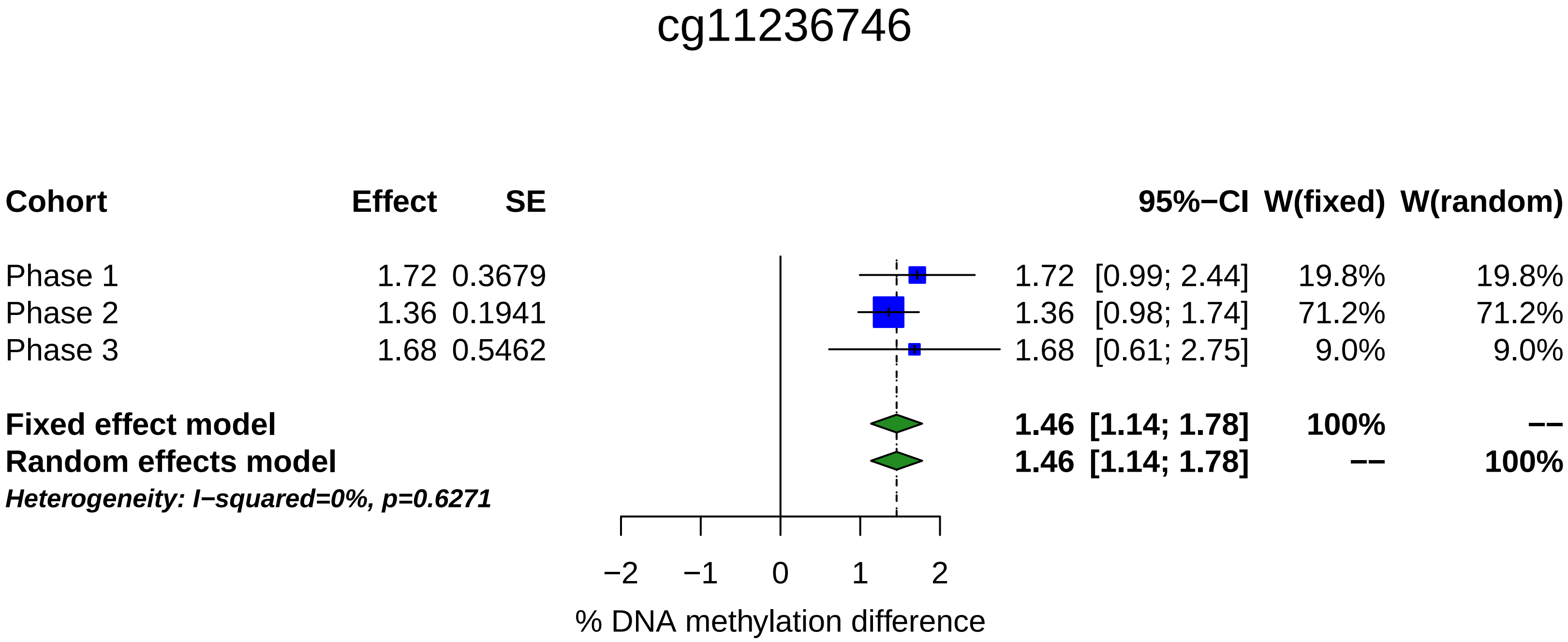
cg11236746
Cohort
Effect
SE
95%−CI
W(fixed)
W(random)
Phase 1 1.72 0.3679 1.72 [0.99; 2.44] 19.8% 19.8%
Phase 2 1.36 0.1941 1.36 [0.98; 1.74] 71.2% 71.2%
Phase 3 1.68 0.5462 1.68 [0.61; 2.75] 9.0% 9.0%
Fixed effect model 1.46 [1.14; 1.78] 100% −−
Random effects model 1.46 [1.14; 1.78] −− 100%
Heterogeneity: I−squared=0%, p=0.6271
−2
−1
0
1
2
% DNA methylation difference

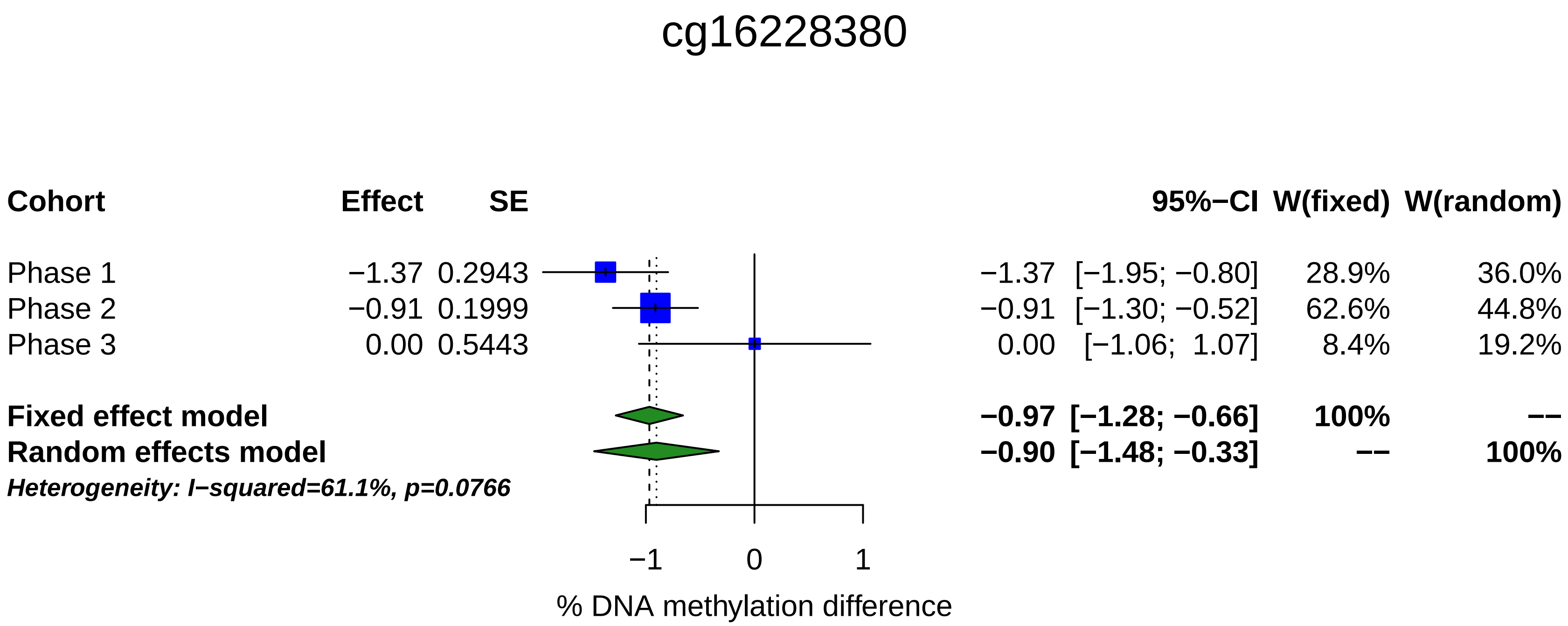
cg16228380
Cohort
Effect
SE
95%−CI
W(fixed)
W(random)
Phase 1 −1.37 0.2943 −1.37 [−1.95; −0.80] 28.9% 36.0%
Phase 2 −0.91 0.1999 −0.91 [−1.30; −0.52] 62.6% 44.8%
Phase 3 0.00 0.5443 0.00 [−1.06; 1.07] 8.4% 19.2%
Fixed effect model −0.97 [−1.28; −0.66] 100% −−
Random effects model −0.90 [−1.48; −0.33] −− 100%
Heterogeneity: I−squared=61.1%, p=0.0766
−1
0
1
% DNA methylation difference

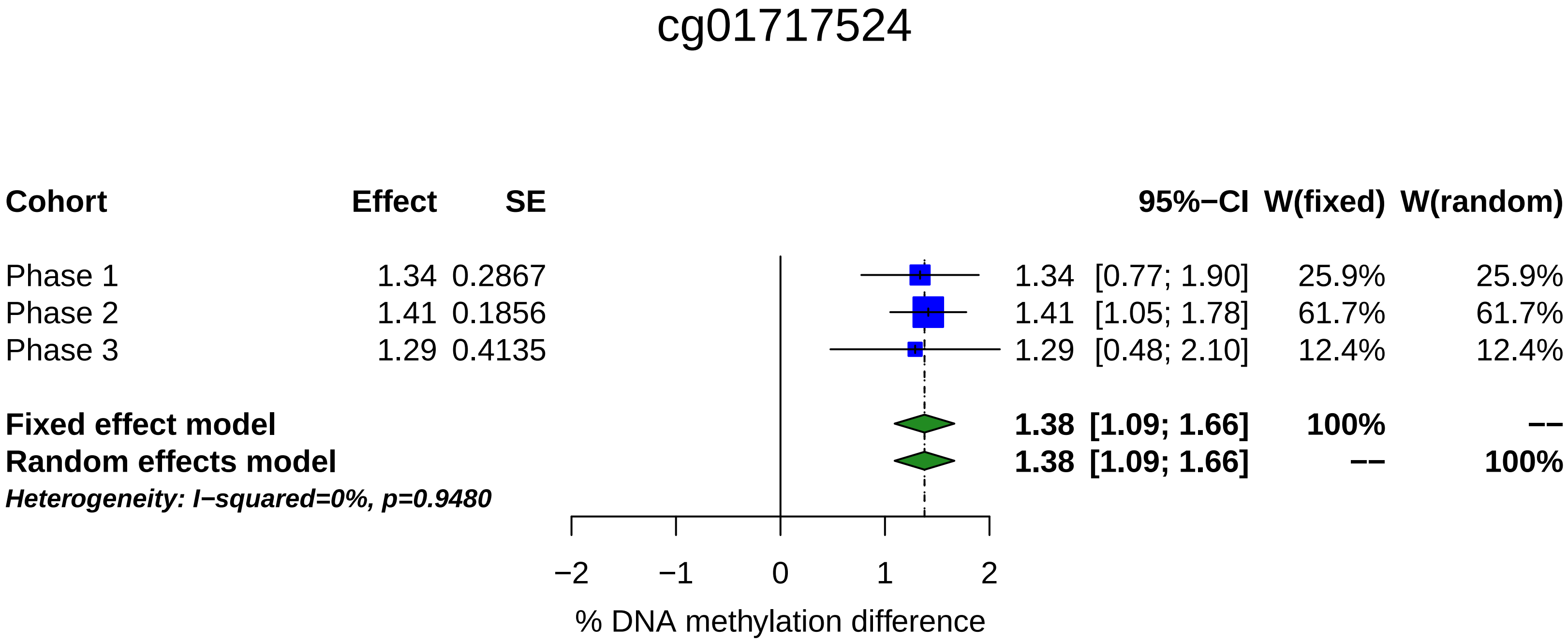
cg01717524
Cohort
Effect
SE
95%−CI
W(fixed)
W(random)
Phase 1 1.34 0.2867 1.34 [0.77; 1.90] 25.9% 25.9%
Phase 2 1.41 0.1856 1.41 [1.05; 1.78] 61.7% 61.7%
Phase 3 1.29 0.4135 1.29 [0.48; 2.10] 12.4% 12.4%
Fixed effect model 1.38 [1.09; 1.66] 100% −−
Random effects model 1.38 [1.09; 1.66] −− 100%
Heterogeneity: I−squared=0%, p=0.9480
−2
−1
0
1
2
% DNA methylation difference

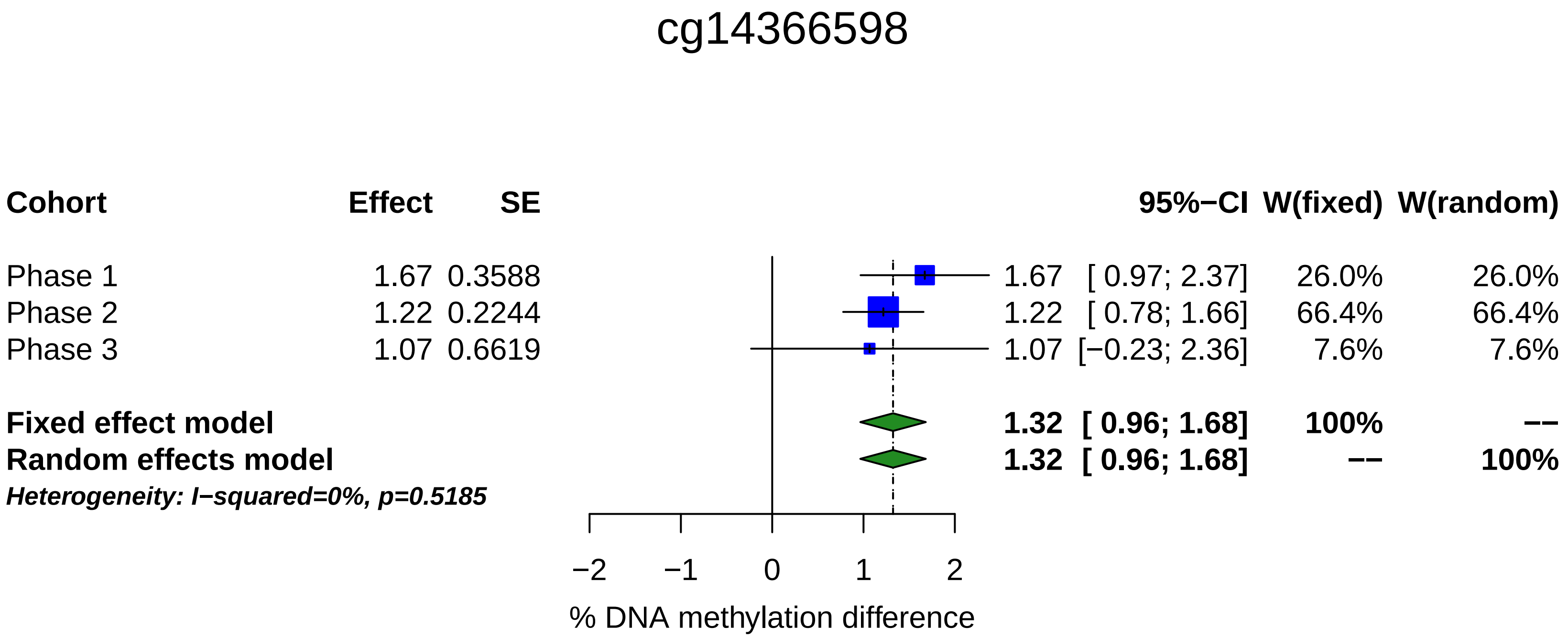
cg14366598
Cohort
Effect
SE
95%−CI
W(fixed)
W(random)
Phase 1 1.67 0.3588 1.67 [0.97; 2.37] 26.0% 26.0%
Phase 2 1.22 0.2244 1.22 [0.78; 1.66] 66.4% 66.4%
Phase 3 1.07 0.6619 1.07 [−0.23; 2.36] 7.6% 7.6%
Fixed effect model 1.32 [0.96; 1.68] 100% −−
Random effects model 1.32 [0.96; 1.68] −− 100%
Heterogeneity: I−squared=0%, p=0.5185
−2
−1
0
1
2
% DNA methylation difference

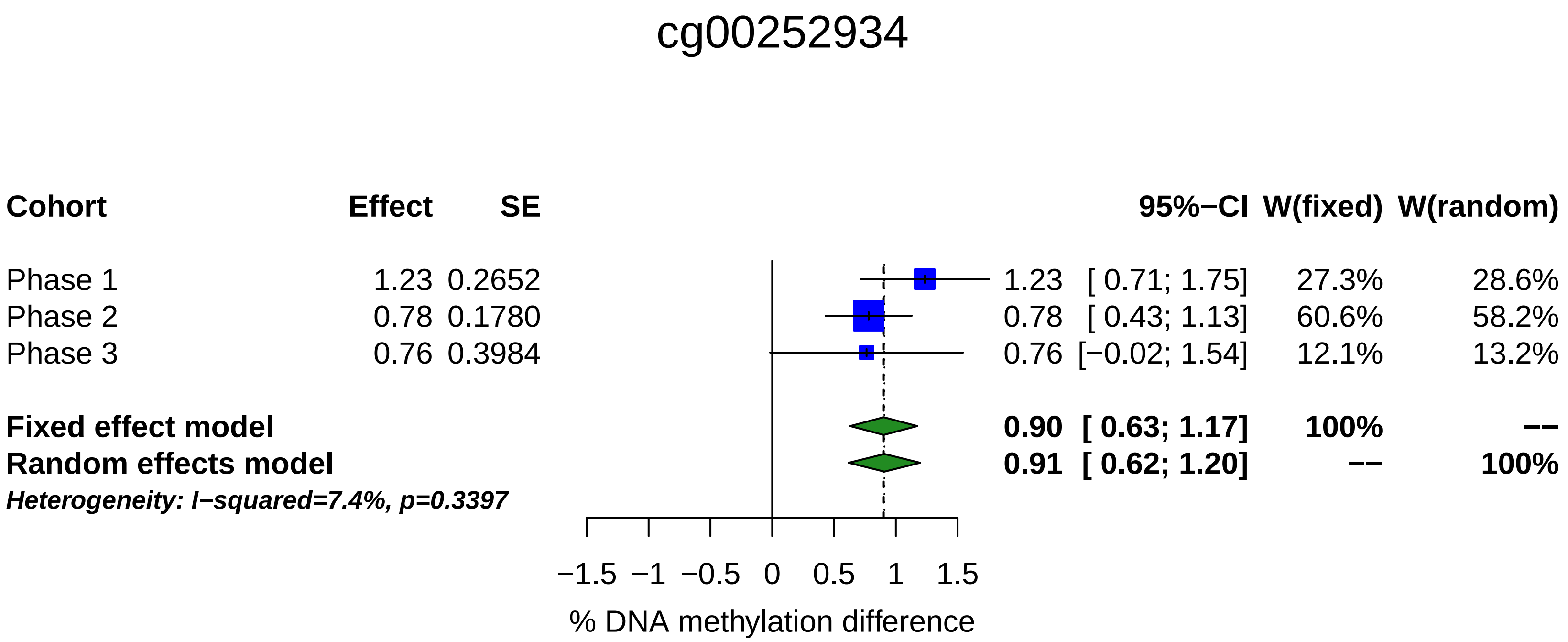
cg00252934
Cohort
Effect
SE
95%−CI
W(fixed)
W(random)
Phase 1 1.23 0.2652 1.23 [0.71; 1.75] 27.3% 28.6%
Phase 2 0.78 0.1780 0.78 [0.43; 1.13] 60.6% 58.2%
Phase 3 0.76 0.3984 0.76 [−0.02; 1.54] 12.1% 13.2%
Fixed effect model 0.90 [0.63; 1.17] 100% −−
Random effects model 0.91 [0.62; 1.20] −− 100%
Heterogeneity: I−squared=7.4%, p=0.3397
−1.5 −1 −0.5 0 0.5 1 1.5
% DNA methylation difference

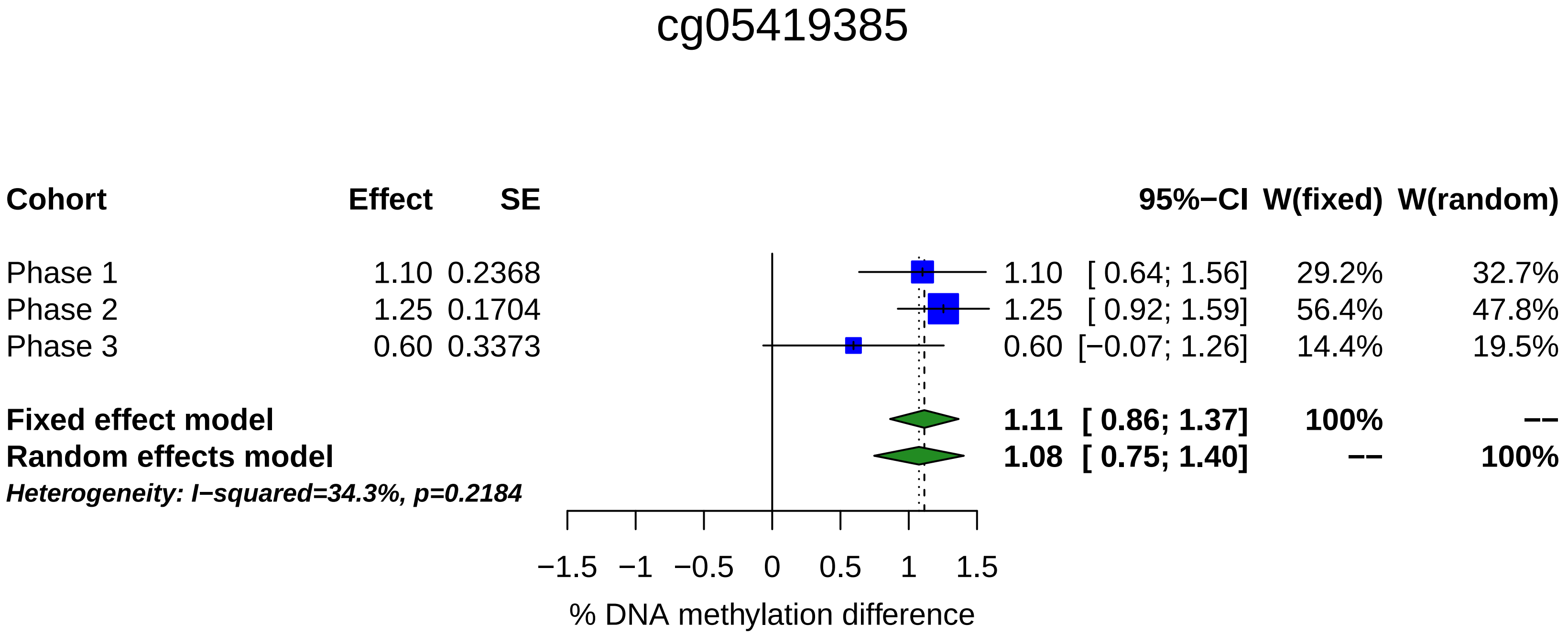
cg05419385
Cohort
Effect
SE
95%−CI
W(fixed)
W(random)
Phase 1 1.10 0.2368 1.10 [0.64; 1.56] 29.2% 32.7%
Phase 2 1.25 0.1704 1.25 [0.92; 1.59] 56.4% 47.8%
Phase 3 0.60 0.3373 0.60 [−0.07; 1.26] 14.4% 19.5%
Fixed effect model 1.11 [0.86; 1.37] 100% −−
Random effects model 1.08 [0.75; 1.40] −− 100%
Heterogeneity: I−squared=34.3%, p=0.2184
−1.5
−1
−0.5
0
0.5
1
1.5
% DNA methylation difference

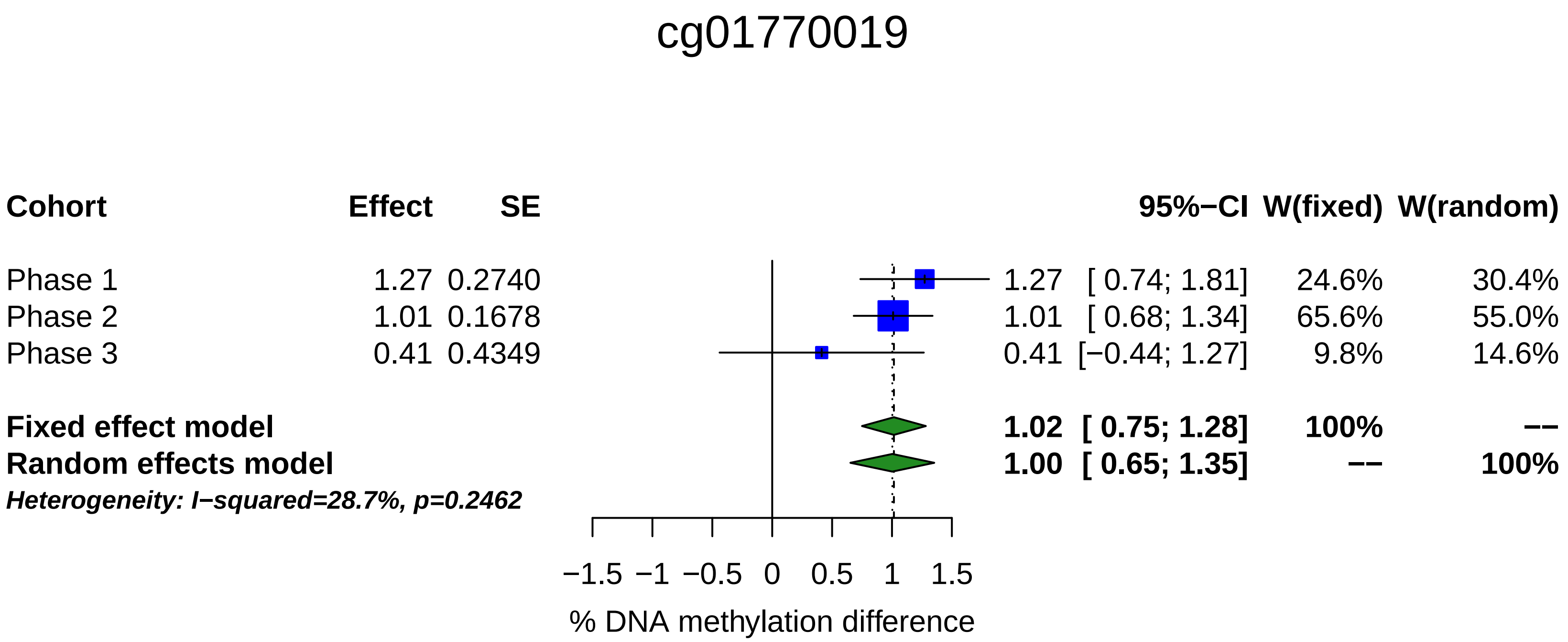
cg01770019
Cohort
Effect
SE
95%−CI
W(fixed)
W(random)
Phase 1 1.27 0.2740 1.27 [0.74; 1.81] 24.6% 30.4%
Phase 2 1.01 0.1678 1.01 [0.68; 1.34] 65.6% 55.0%
Phase 3 0.41 0.4349 0.41 [−0.44; 1.27] 9.8% 14.6%
Fixed effect model 1.02 [0.75; 1.28] 100% −−
Random effects model 1.00 [0.65; 1.35] −− 100%
Heterogeneity: I−squared=28.7%, p=0.2462
−1.5 −1 −0.5 0 0.5 1 1.5
% DNA methylation difference

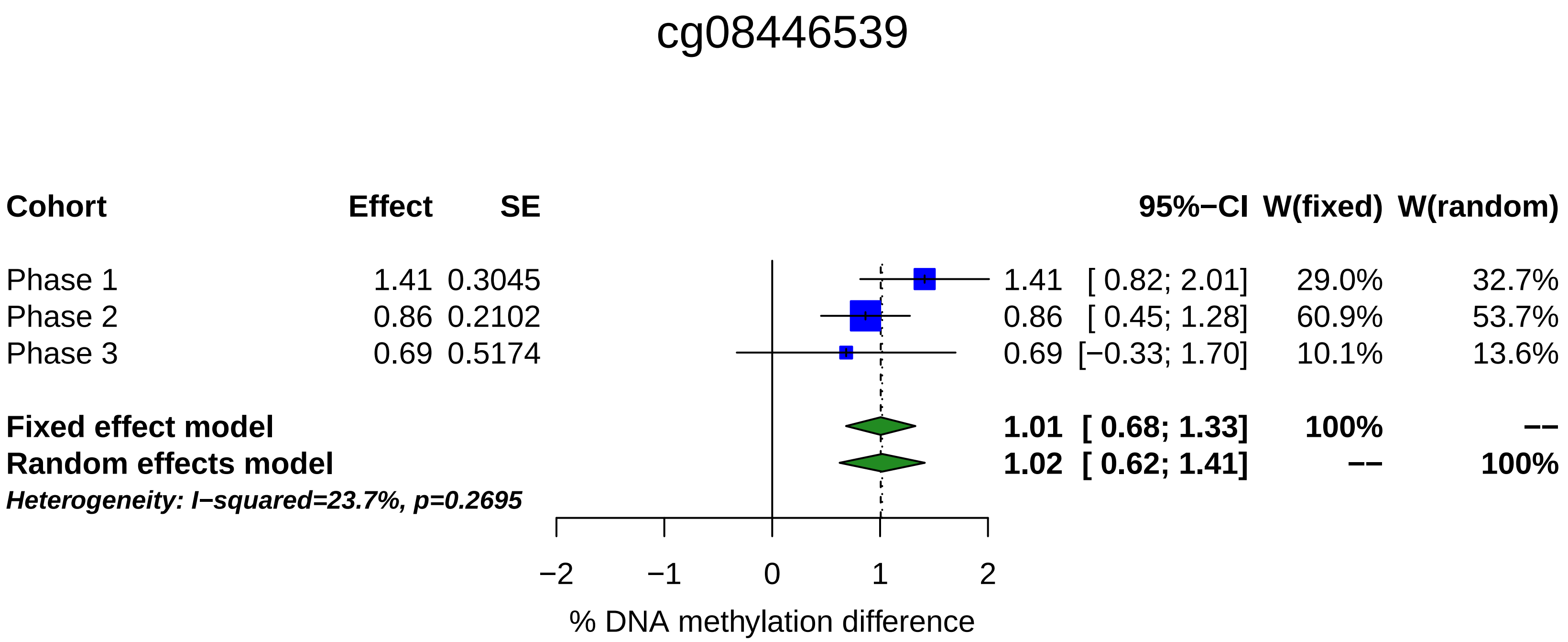
cg08446539
Cohort
Effect
SE
95%−CI
W(fixed)
W(random)
Phase 1 1.41 0.3045 1.41 [0.82; 2.01] 29.0% 32.7%
Phase 2 0.86 0.2102 0.86 [0.45; 1.28] 60.9% 53.7%
Phase 3 0.69 0.5174 0.69 [−0.33; 1.70] 10.1% 13.6%
Fixed effect model 1.01 [0.68; 1.33] 100% −−
Random effects model 1.02 [0.62; 1.41] −− 100%
Heterogeneity: I−squared=23.7%, p=0.2695
−2
−1
0
1
2
% DNA methylation difference

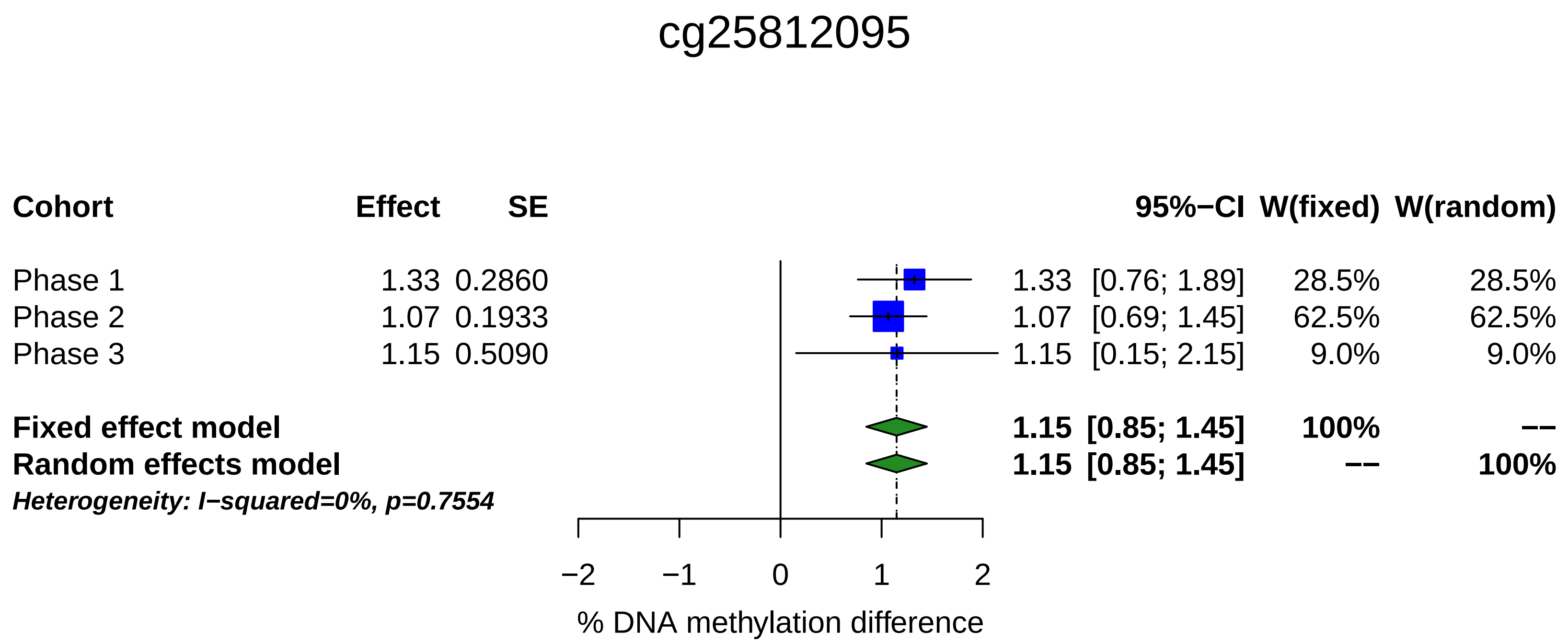
cg25812095
Cohort
Effect
SE
95%−CI
W(fixed)
W(random)
Phase 1 1.33 0.2860 1.33 [0.76; 1.89] 28.5% 28.5%
Phase 2 1.07 0.1933 1.07 [0.69; 1.45] 62.5% 62.5%
Phase 3 1.15 0.5090 1.15 [0.15; 2.15] 9.0% 9.0%
Fixed effect model 1.15 [0.85; 1.45] 100% −−
Random effects model 1.15 [0.85; 1.45] −− 100%
Heterogeneity: I−squared=0%, p=0.7554
−2
−1
0
1
2
% DNA methylation difference

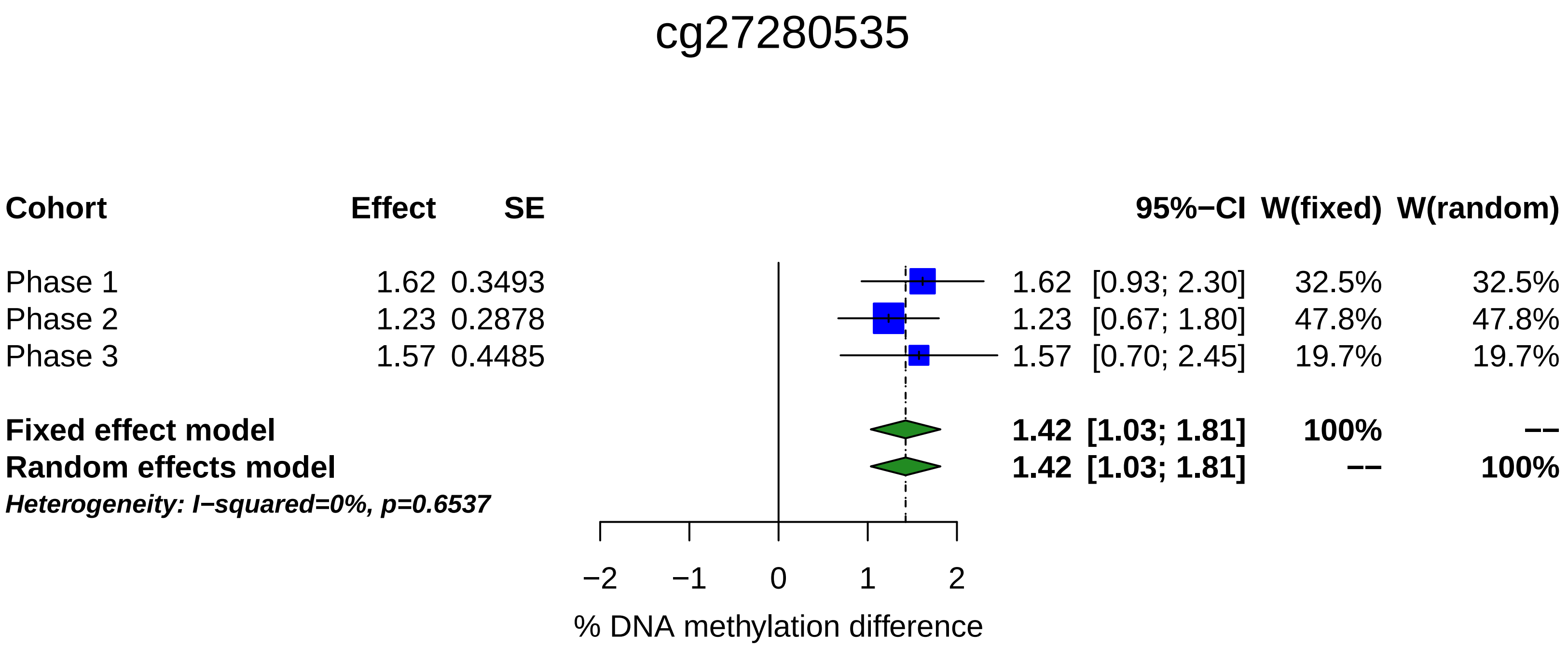
cg27280535
Cohort
Effect
SE
95%−CI
W(fixed)
W(random)
Phase 1
1.62
0.3493
1.62 [0.93; 2.30]
32.5%
32.5%
Phase 2
1.23
0.2878
1.23 [0.67; 1.80]
47.8%
47.8%
Phase 3
1.57
0.4485
1.57 [0.70; 2.45]
19.7%
19.7%
Fixed effect model
1.42 [1.03; 1.81]
100%
−−
Random effects model
1.42 [1.03; 1.81]
−−
100%
Heterogeneity: I−squared=0%, p=0.6537
−2
−1
0
1
2
% DNA methylation difference

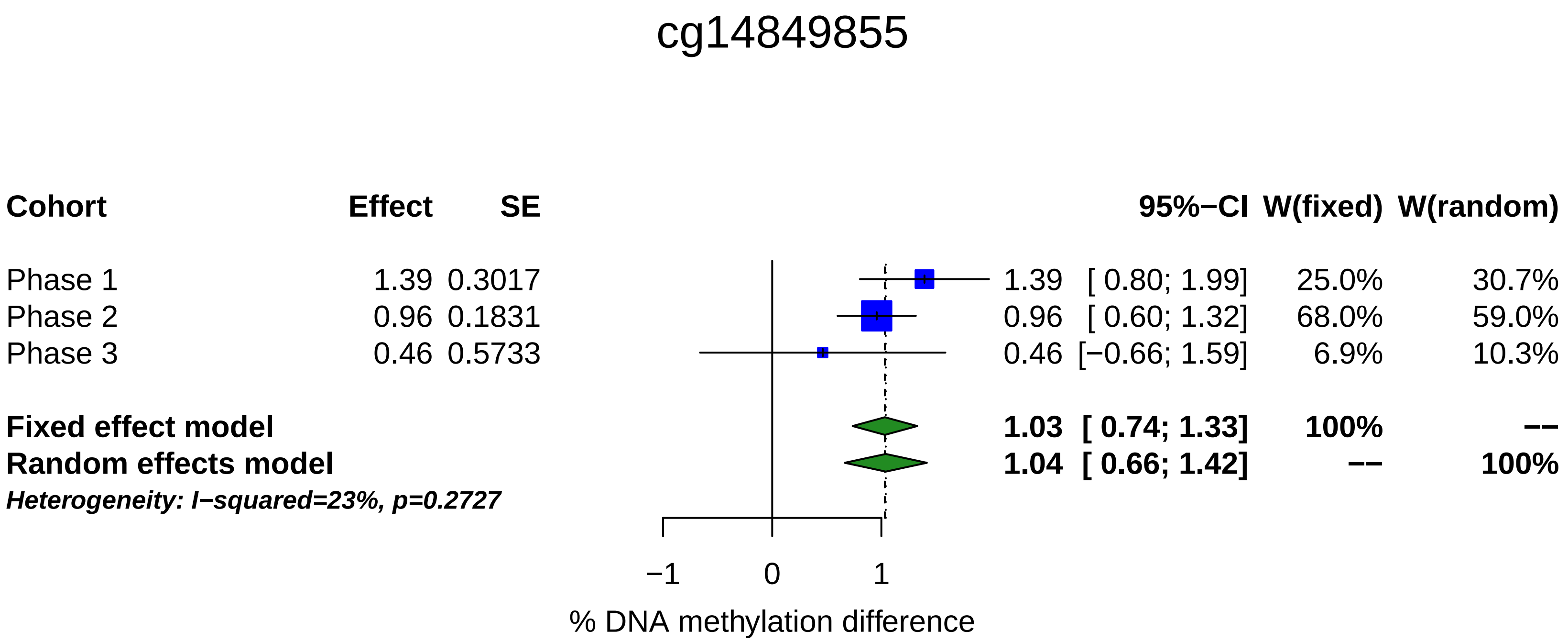
cg14849855
Cohort
Effect
SE
95%−CI
W(fixed)
W(random)
Phase 1 1.39 0.3017 1.39 [0.80; 1.99] 25.0% 30.7%
Phase 2 0.96 0.1831 0.96 [0.60; 1.32] 68.0% 59.0%
Phase 3 0.46 0.5733 0.46 [−0.66; 1.59] 6.9% 10.3%
Fixed effect model 1.03 [0.74; 1.33] 100% −−
Random effects model 1.04 [0.66; 1.42] −− 100%
Heterogeneity: I−squared=23%, p=0.2727
−1
0
1
% DNA methylation difference

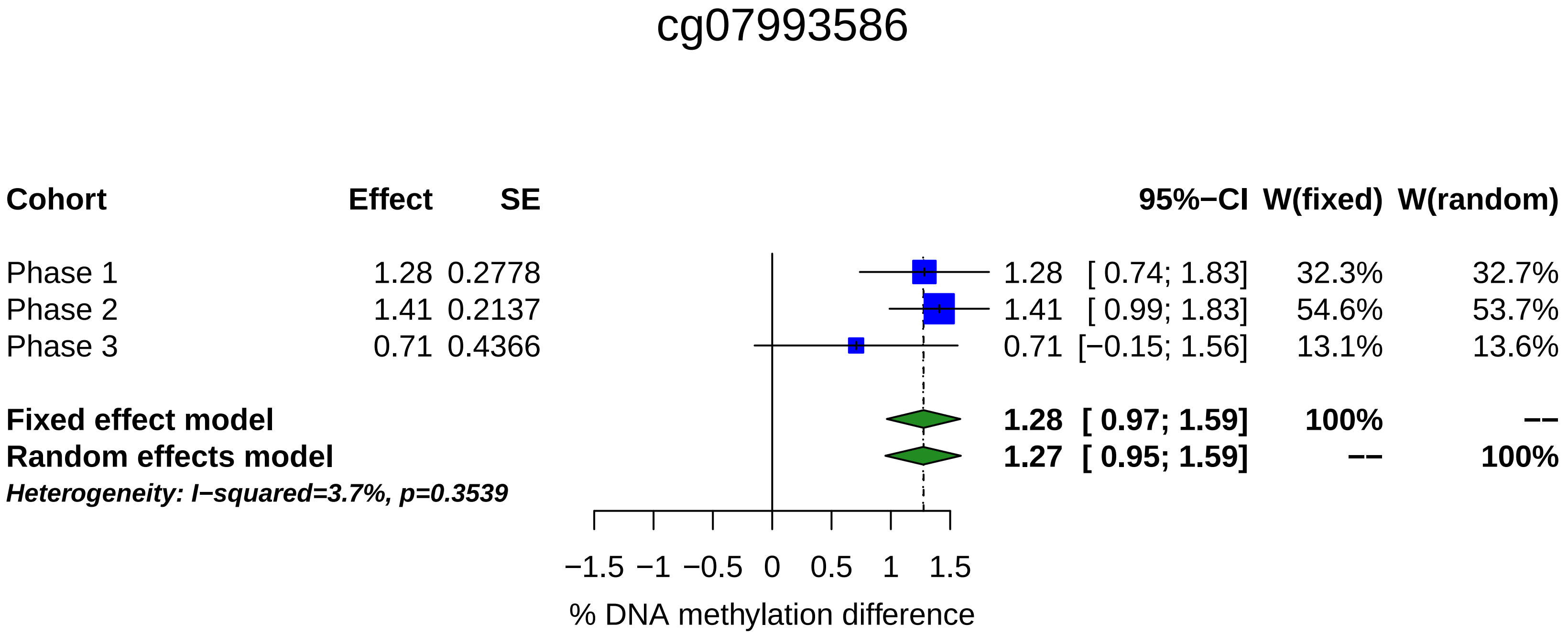
cg07993586
Cohort
Effect
SE
95%−CI
W(fixed)
W(random)
Phase 1 1.28 0.2778 1.28 [0.74; 1.83] 32.3% 32.7%
Phase 2 1.41 0.2137 1.41 [0.99; 1.83] 54.6% 53.7%
Phase 3 0.71 0.4366 0.71 [−0.15; 1.56] 13.1% 13.6%
Fixed effect model 1.28 [0.97; 1.59] 100% −−
Random effects model 1.27 [0.95; 1.59] −− 100%
Heterogeneity: I−squared=3.7%, p=0.3539
−1.5 −1 −0.5 0 0.5 1 1.5
% DNA methylation difference

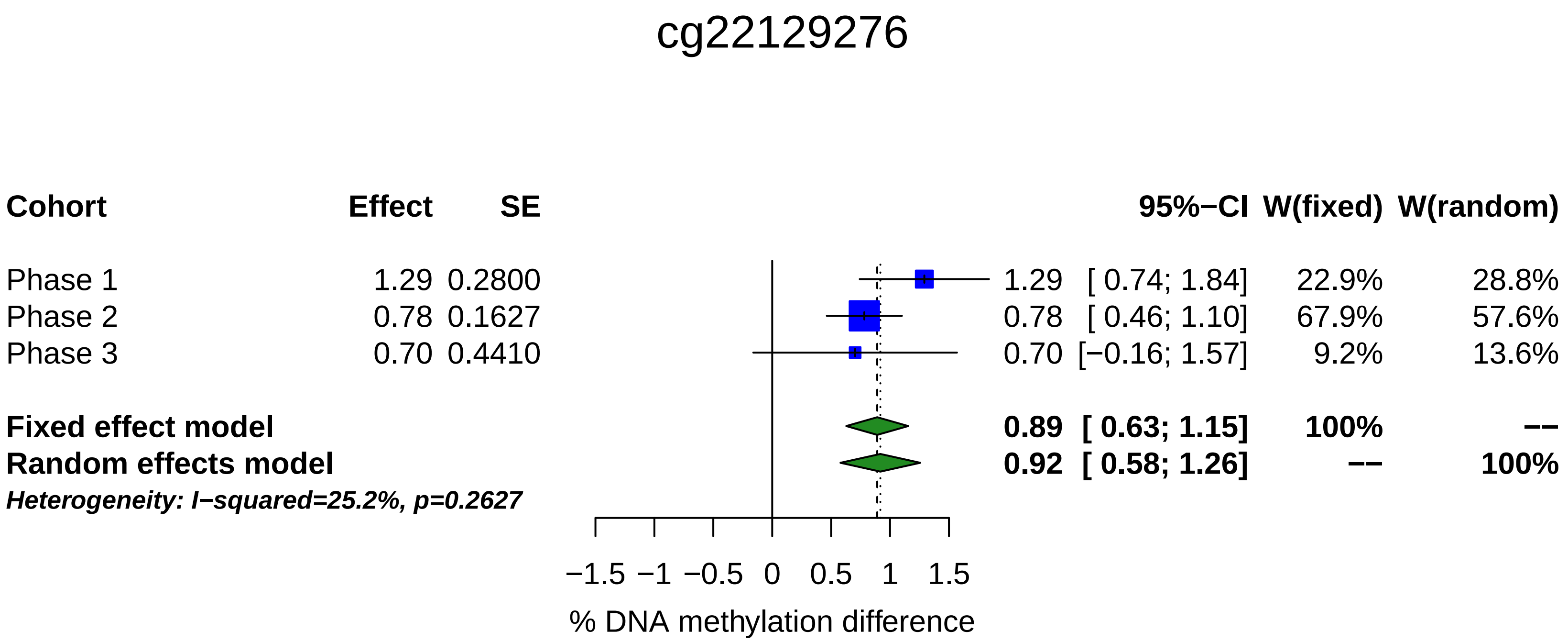
cg22129276
Cohort
Effect
SE
95%−CI
W(fixed)
W(random)
Phase 1 1.29 0.2800 1.29 [0.74; 1.84] 22.9% 28.8%
Phase 2 0.78 0.1627 0.78 [0.46; 1.10] 67.9% 57.6%
Phase 3 0.70 0.4410 0.70 [−0.16; 1.57] 9.2% 13.6%
Fixed effect model 0.89 [0.63; 1.15] 100% −−
Random effects model 0.92 [0.58; 1.26] −− 100%
Heterogeneity: I−squared=25.2%, p=0.2627
−1.5 −1 −0.5 0 0.5 1 1.5
% DNA methylation difference

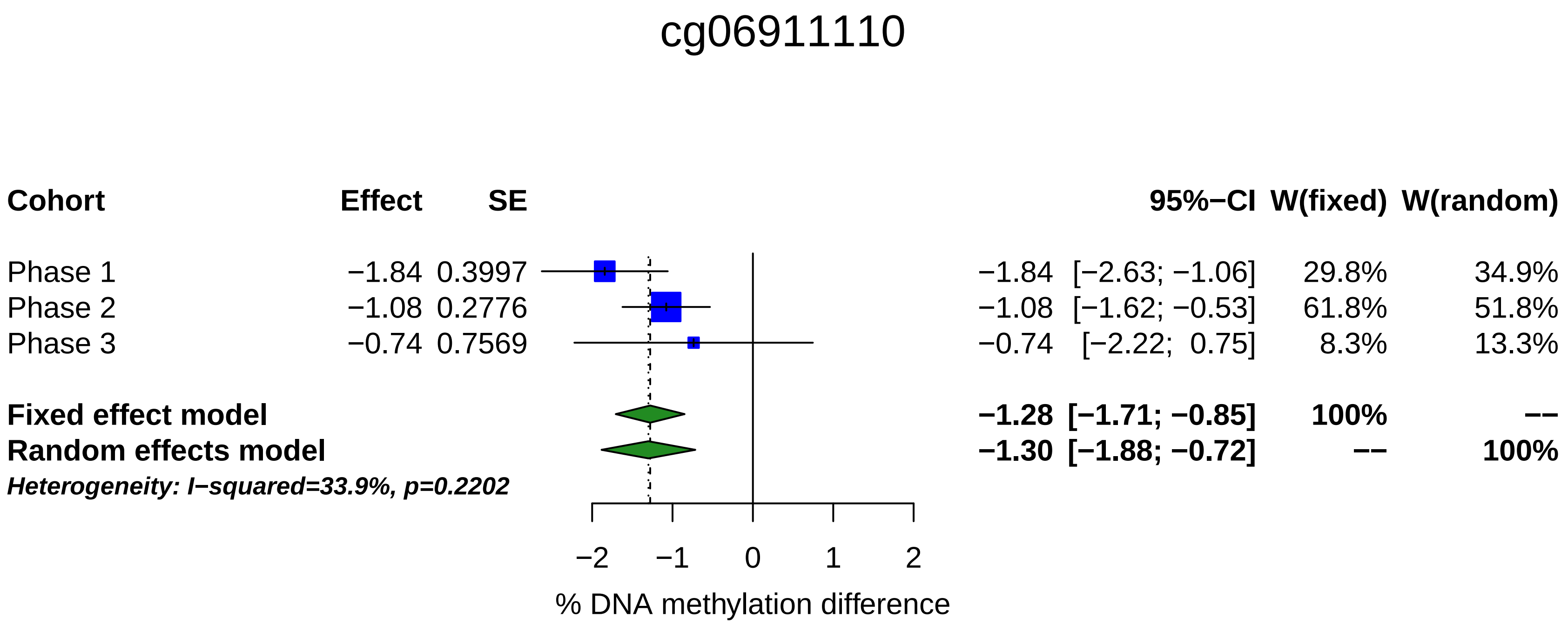
cg06911110
Cohort
Effect
SE
95%−CI
W(fixed)
W(random)
Phase 1 −1.84 0.3997 −1.84 [−2.63; −1.06] 29.8% 34.9%
Phase 2 −1.08 0.2776 −1.08 [−1.62; −0.53] 61.8% 51.8%
Phase 3 −0.74 0.7569 −0.74 [−2.22; 0.75] 8.3% 13.3%
Fixed effect model −1.28 [−1.71; −0.85] 100% −−
Random effects model −1.30 [−1.88; −0.72] −− 100%
Heterogeneity: I−squared=33.9%, p=0.2202
−2
−1
0
1
2
% DNA methylation difference

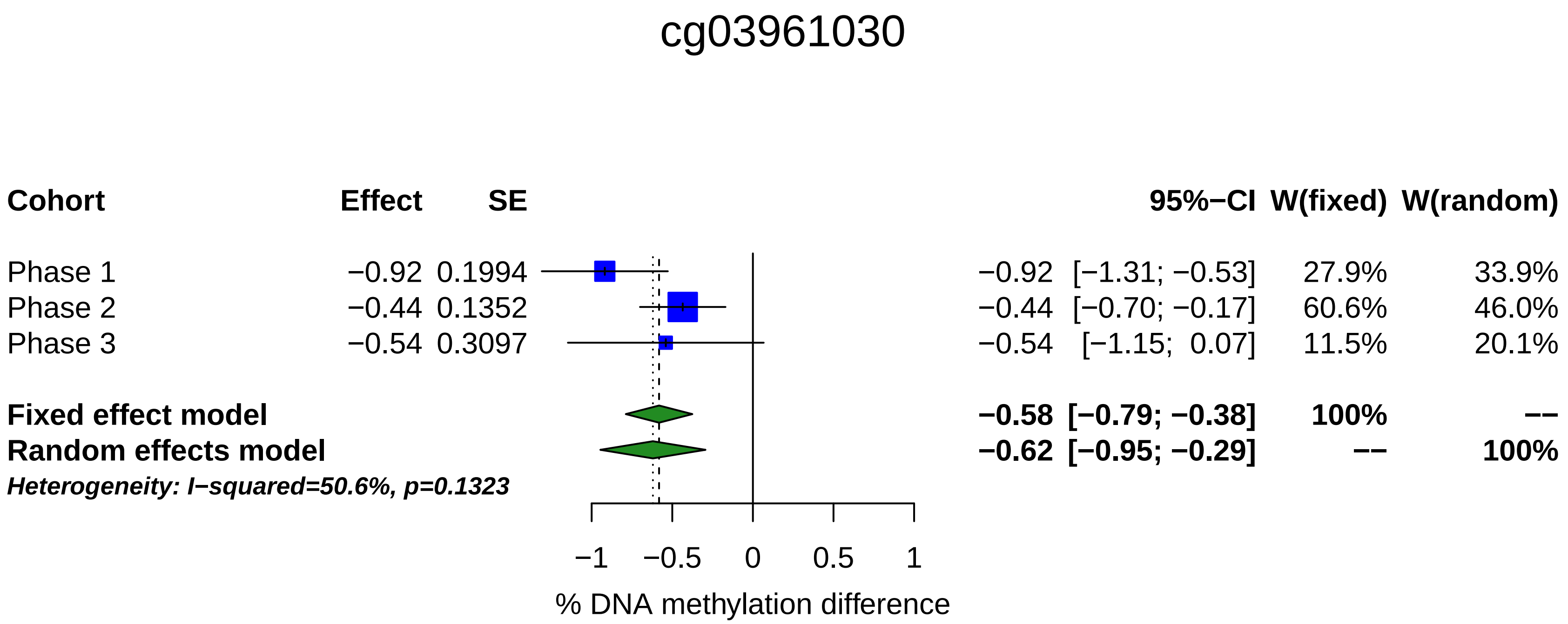
cg03961030
Cohort
Effect
SE
95%−CI
W(fixed)
W(random)
Phase 1 −0.92 0.1994 −0.92 [−1.31; −0.53] 27.9% 33.9%
Phase 2 −0.44 0.1352 −0.44 [−0.70; −0.17] 60.6% 46.0%
Phase 3 −0.54 0.3097 −0.54 [−1.15; 0.07] 11.5% 20.1%
Fixed effect model −0.58 [−0.79; −0.38] 100% −−
Random effects model −0.62 [−0.95; −0.29] −− 100%
Heterogeneity: I−squared=50.6%, p=0.1323
−1
−0.5
0
0.5
1
% DNA methylation difference

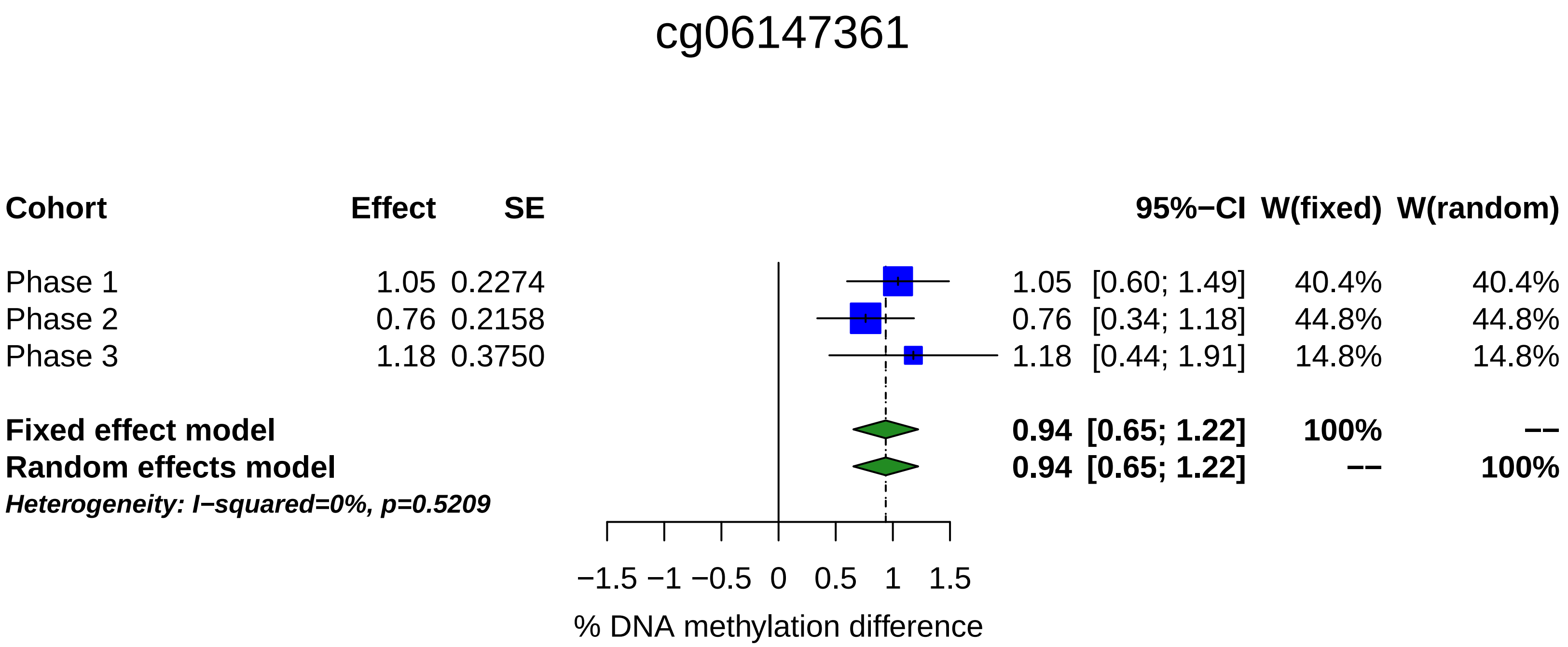
cg06147361
Cohort
Effect
SE
95%−CI
W(fixed)
W(random)
Phase 1 1.05 0.2274 1.05 [0.60; 1.49] 40.4% 40.4%
Phase 2 0.76 0.2158 0.76 [0.34; 1.18] 44.8% 44.8%
Phase 3 1.18 0.3750 1.18 [0.44; 1.91] 14.8% 14.8%
Fixed effect model 0.94 [0.65; 1.22] 100% −−
Random effects model 0.94 [0.65; 1.22] −− 100%
Heterogeneity: I−squared=0%, p=0.5209
−1.5 −1 −0.5 0 0.5 1 1.5
% DNA methylation difference

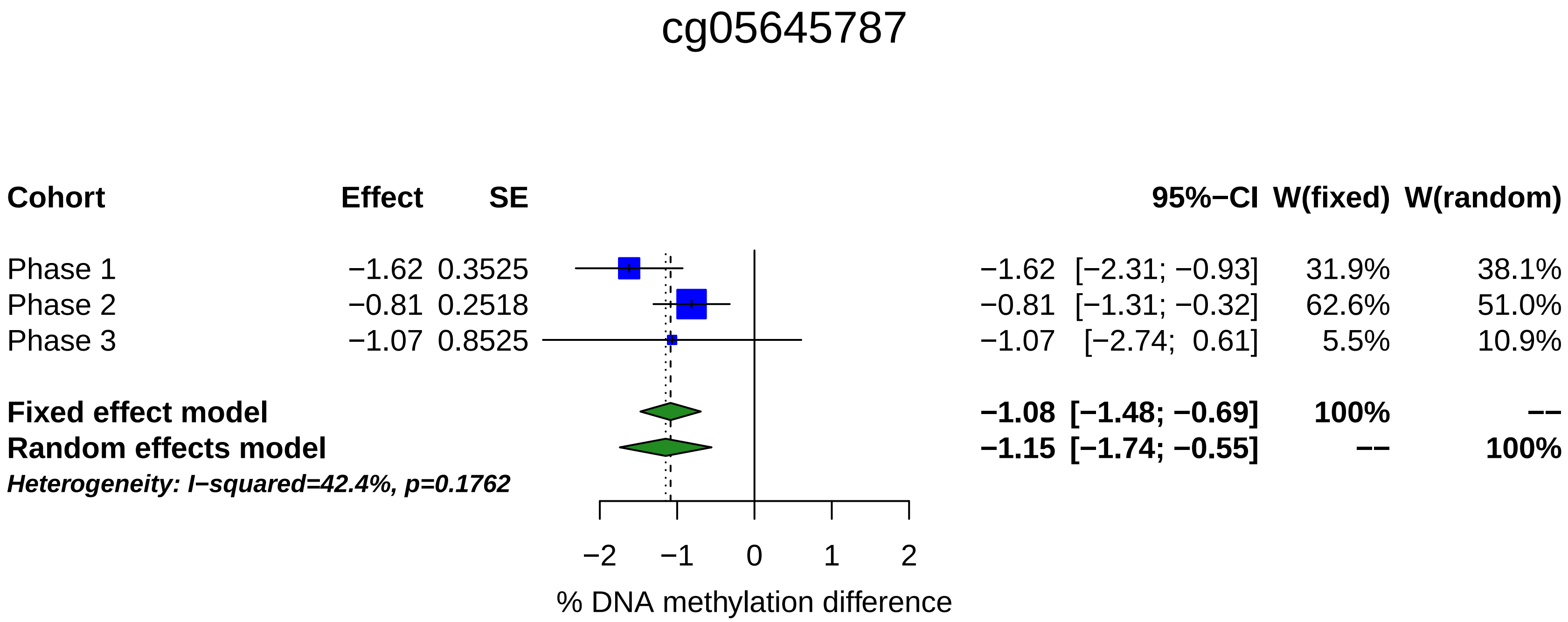
cg05645787
Cohort
Effect
SE
95%−CI
W(fixed)
W(random)
Phase 1 −1.62 0.3525 −1.62 [−2.31; −0.93] 31.9% 38.1%
Phase 2 −0.81 0.2518 −0.81 [−1.31; −0.32] 62.6% 51.0%
Phase 3 −1.07 0.8525 −1.07 [−2.74; 0.61] 5.5% 10.9%
Fixed effect model −1.08 [−1.48; −0.69] 100% −−
Random effects model −1.15 [−1.74; −0.55] −− 100%
Heterogeneity: I−squared=42.4%, p=0.1762
−2
−1
0
1
2
% DNA methylation difference

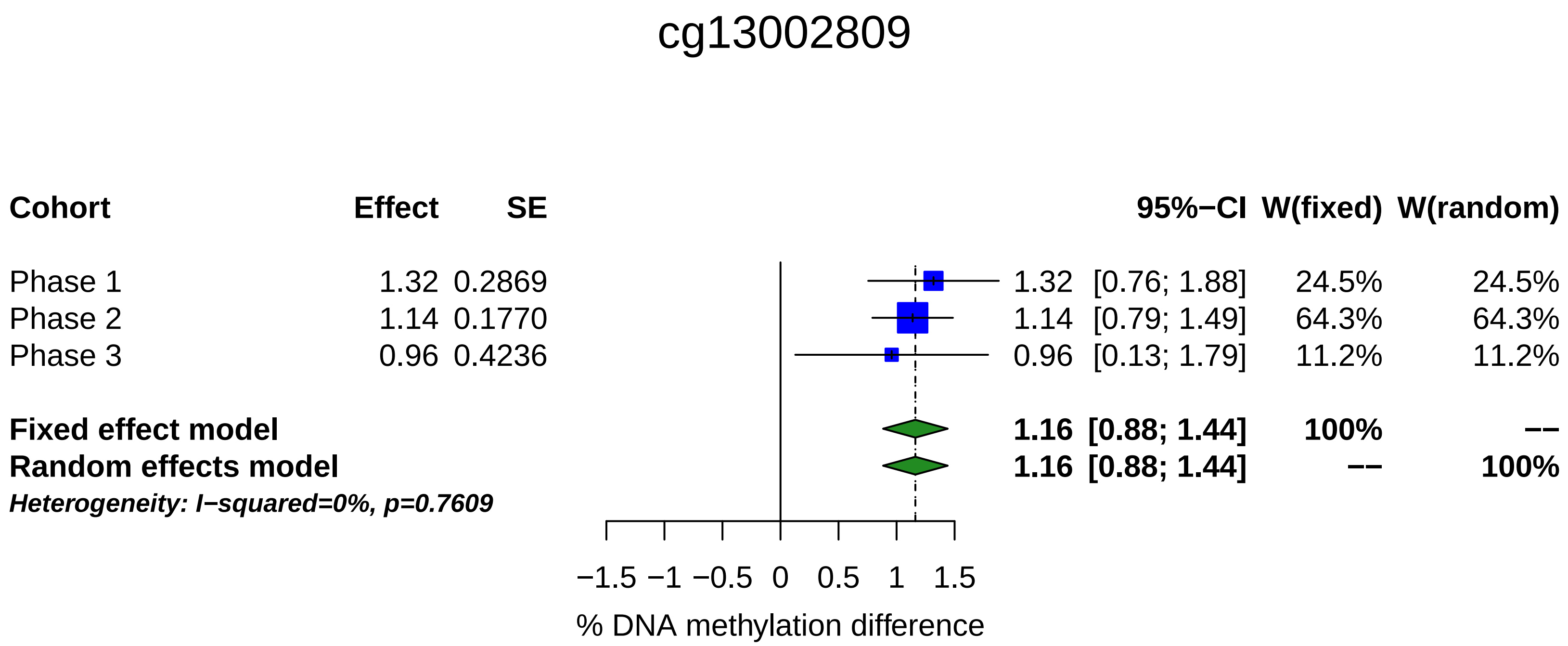
cg13002809
Cohort
Effect
SE
95%−CI
W(fixed)
W(random)
Phase 1
1.32
0.2869
1.32 [0.76; 1.88]
24.5%
24.5%
Phase 2
1.14
0.1770
1.14 [0.79; 1.49]
64.3%
64.3%
Phase 3
0.96
0.4236
0.96 [0.13; 1.79]
11.2%
11.2%
Fixed effect model
1.16 [0.88; 1.44]
100%
−−
Random effects model
1.16 [0.88; 1.44]
−−
100%
Heterogeneity: I−squared=0%, p=0.7609
−1.5 −1 −0.5 0 0.5 1 1.5
% DNA methylation difference

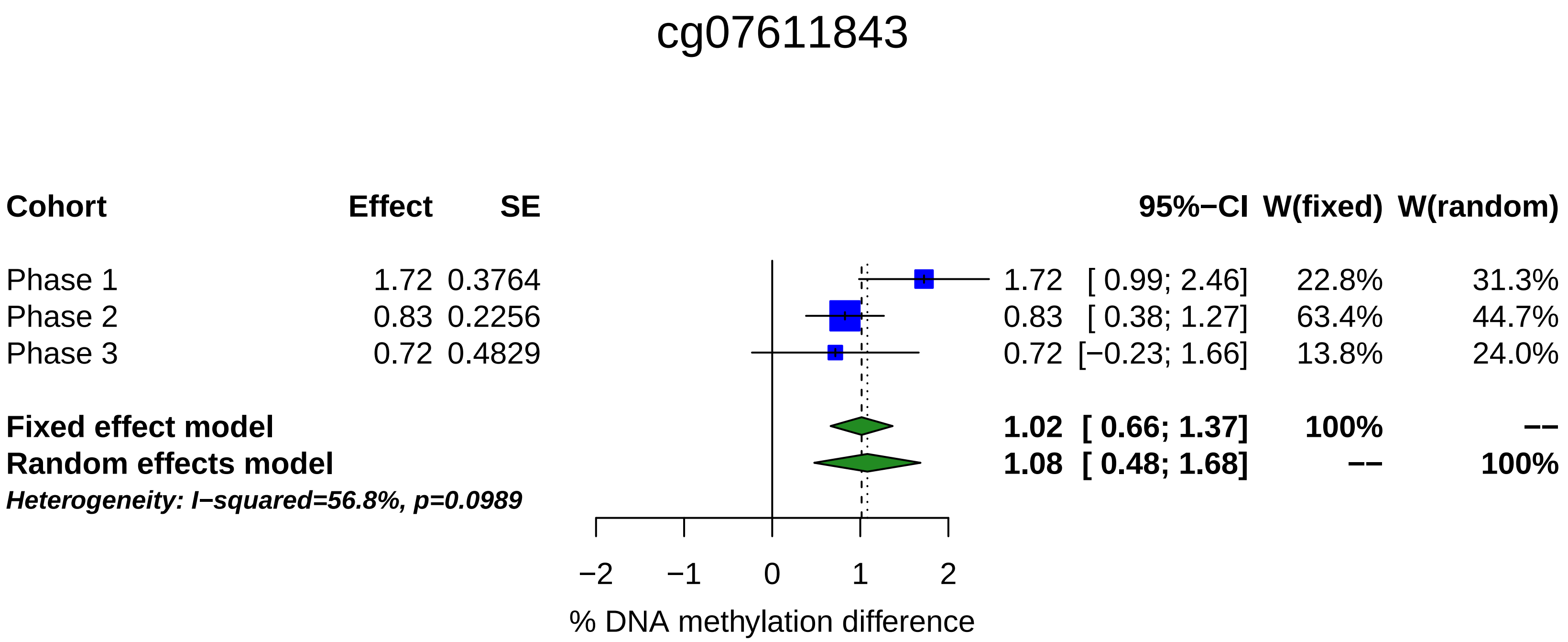
cg07611843
Cohort
Effect
SE
95%−CI
W(fixed)
W(random)
Phase 1 1.72 0.3764 1.72 [0.99; 2.46] 22.8% 31.3%
Phase 2 0.83 0.2256 0.83 [0.38; 1.27] 63.4% 44.7%
Phase 3 0.72 0.4829 0.72 [−0.23; 1.66] 13.8% 24.0%
Fixed effect model 1.02 [0.66; 1.37] 100% −−
Random effects model 1.08 [0.48; 1.68] −− 100%
Heterogeneity: I−squared=56.8%, p=0.0989
−2
−1
0
1
2
% DNA methylation difference

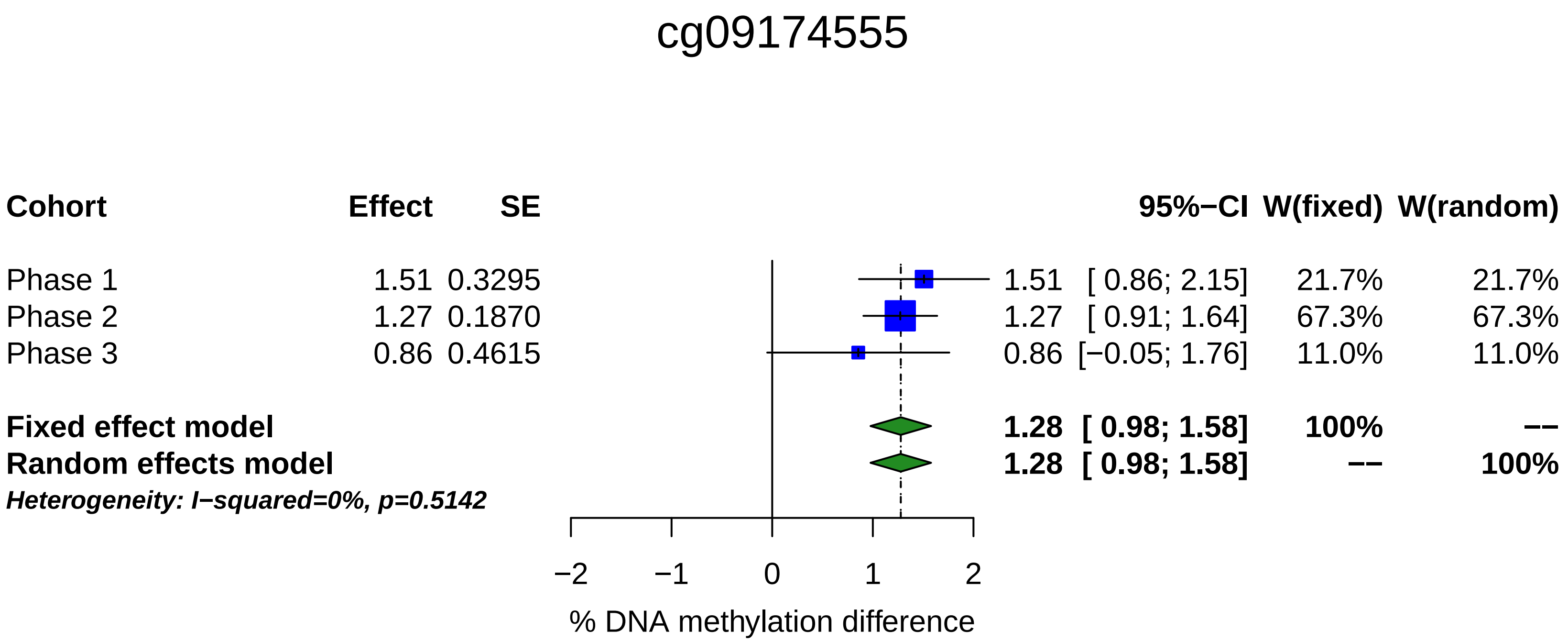
cg09174555
Cohort
Effect
SE
95%−CI
W(fixed)
W(random)
Phase 1 1.51 0.3295 1.51 [0.86; 2.15] 21.7% 21.7%
Phase 2 1.27 0.1870 1.27 [0.91; 1.64] 67.3% 67.3%
Phase 3 0.86 0.4615 0.86 [−0.05; 1.76] 11.0% 11.0%
Fixed effect model 1.28 [0.98; 1.58] 100% −−
Random effects model 1.28 [0.98; 1.58] −− 100%
Heterogeneity: I−squared=0%, p=0.5142
−2
−1
0
1
2
% DNA methylation difference

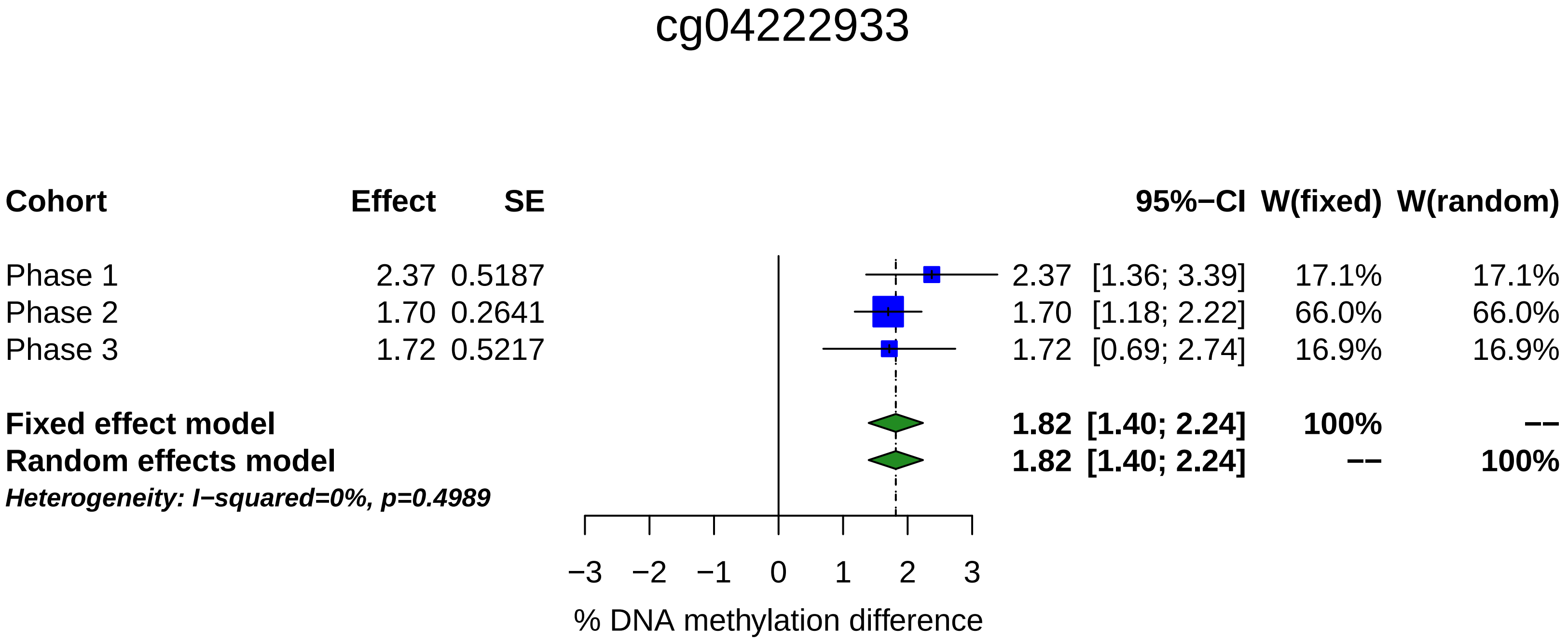
cg04222933
Cohort
Effect
SE
95%−CI
W(fixed)
W(random)
Phase 1 2.37 0.5187 2.37 [1.36; 3.39] 17.1% 17.1%
Phase 2 1.70 0.2641 1.70 [1.18; 2.22] 66.0% 66.0%
Phase 3 1.72 0.5217 1.72 [0.69; 2.74] 16.9% 16.9%
Fixed effect model 1.82 [1.40; 2.24] 100% −−
Random effects model 1.82 [1.40; 2.24] −− 100%
Heterogeneity: I−squared=0%, p=0.4989
−3
−2
−1
0
1
2
3
% DNA methylation difference

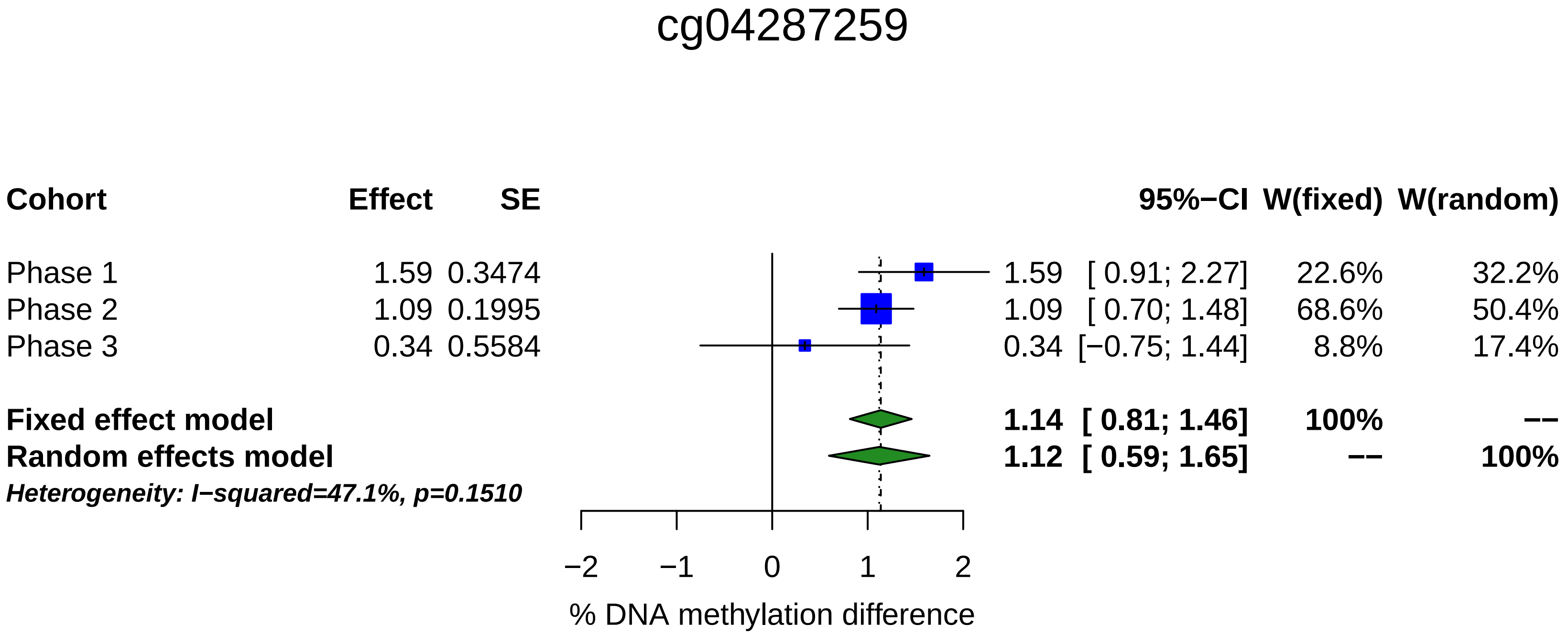
cg04287259
Cohort
Effect
SE
95%−CI
W(fixed)
W(random)
Phase 1 1.59 0.3474 1.59 [0.91; 2.27] 22.6% 32.2%
Phase 2 1.09 0.1995 1.09 [0.70; 1.48] 68.6% 50.4%
Phase 3 0.34 0.5584 0.34 [−0.75; 1.44] 8.8% 17.4%
Fixed effect model 1.14 [0.81; 1.46] 100% −−
Random effects model 1.12 [0.59; 1.65] −− 100%
Heterogeneity: I−squared=47.1%, p=0.1510
−2
−1
0
1
2
% DNA methylation difference

cg12524168

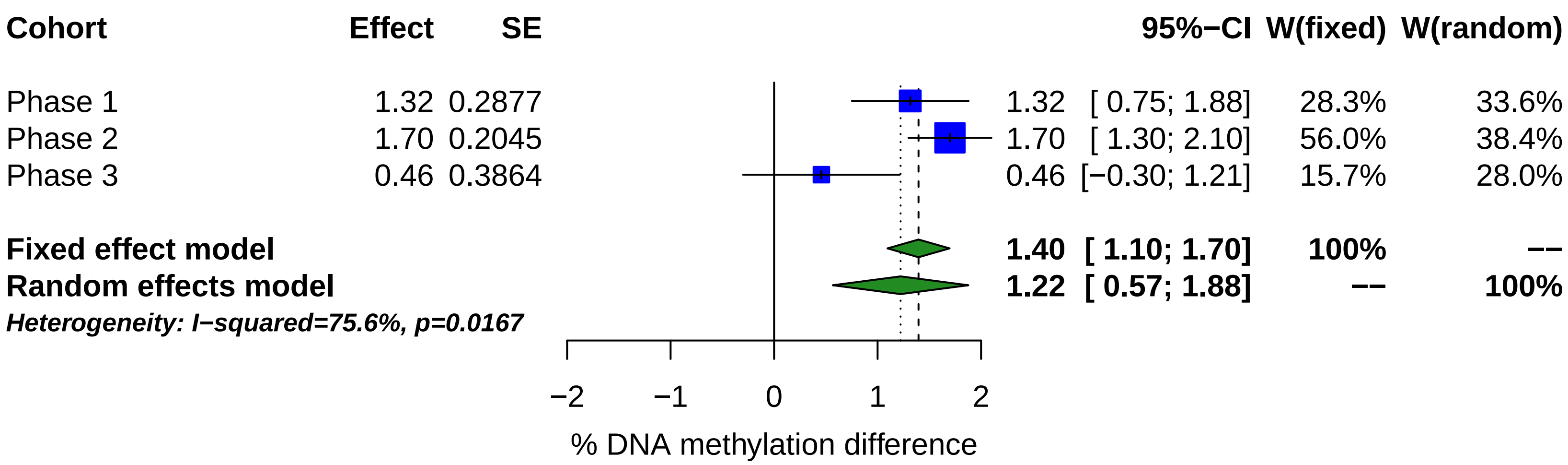

Cohort
Effect
SE
95%−CI
W(fixed)
W(random)
Phase 1
1.32
0.2877
1.32 [0.75; 1.88]
28.3%
33.6%
Phase 2
1.70
0.2045
1.70 [1.30; 2.10]
56.0%
38.4%
Phase 3
0.46
0.3864
0.46 [−0.30; 1.21]
15.7%
28.0%
Fixed effect model
1.40 [1.10; 1.70]
100%
−−
Random effects model
1.22 [0.57; 1.88]
−−
100%
Heterogeneity: I−squared=75.6%, p=0.0167
−2
−1
0
1
2
% DNA methylation difference

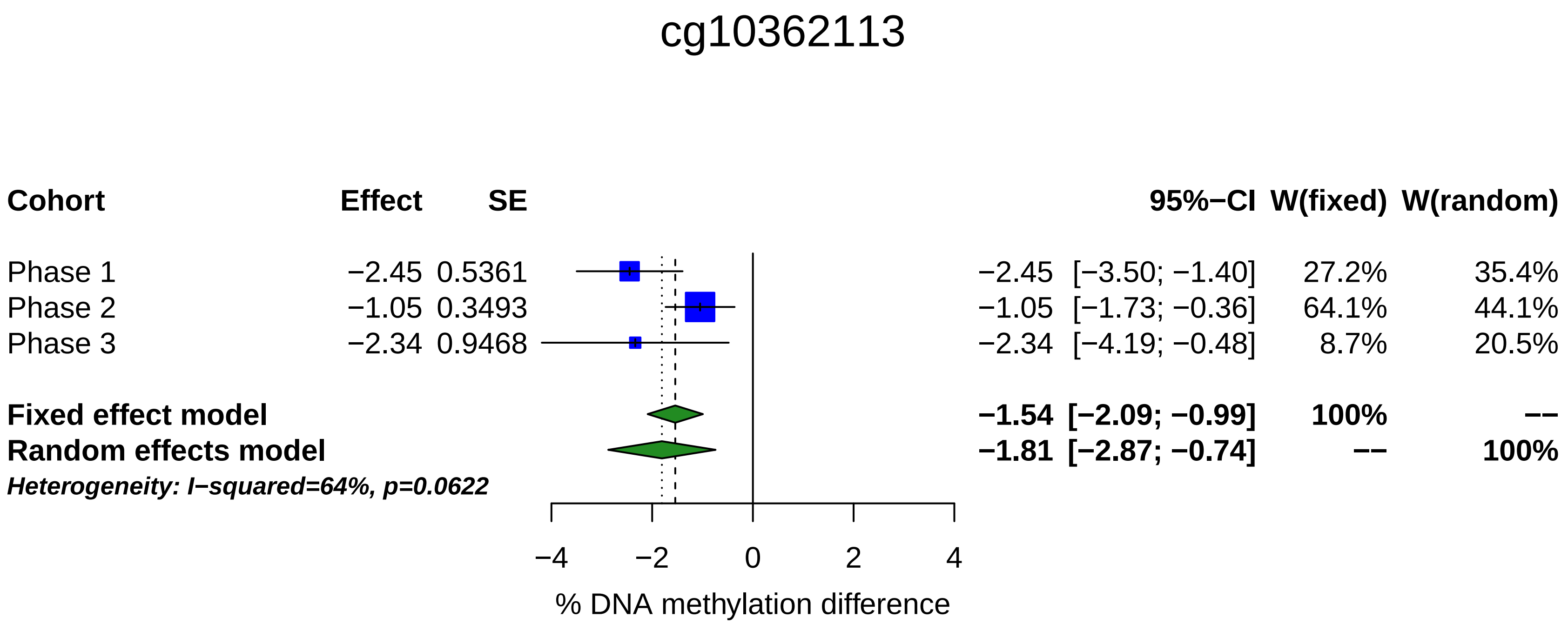

cg10362113
Cohort
Effect
SE
95%−CI
W(fixed)
W(random)
Phase 1
−2.45
0.5361
−2.45 [−3.50; −1.40]
27.2%
35.4%
Phase 2
−1.05
0.3493
−1.05 [−1.73; −0.36]
64.1%
44.1%
Phase 3
−2.34
0.9468
−2.34 [−4.19; −0.48]
8.7%
20.5%
Fixed effect model
−1.54 [−2.09; −0.99]
100%
−−
Random effects model
−1.81 [−2.87; −0.74]
−−
100%
Heterogeneity: I−squared=64%, p=0.0622
−4
−2
0
2
4
% DNA methylation difference

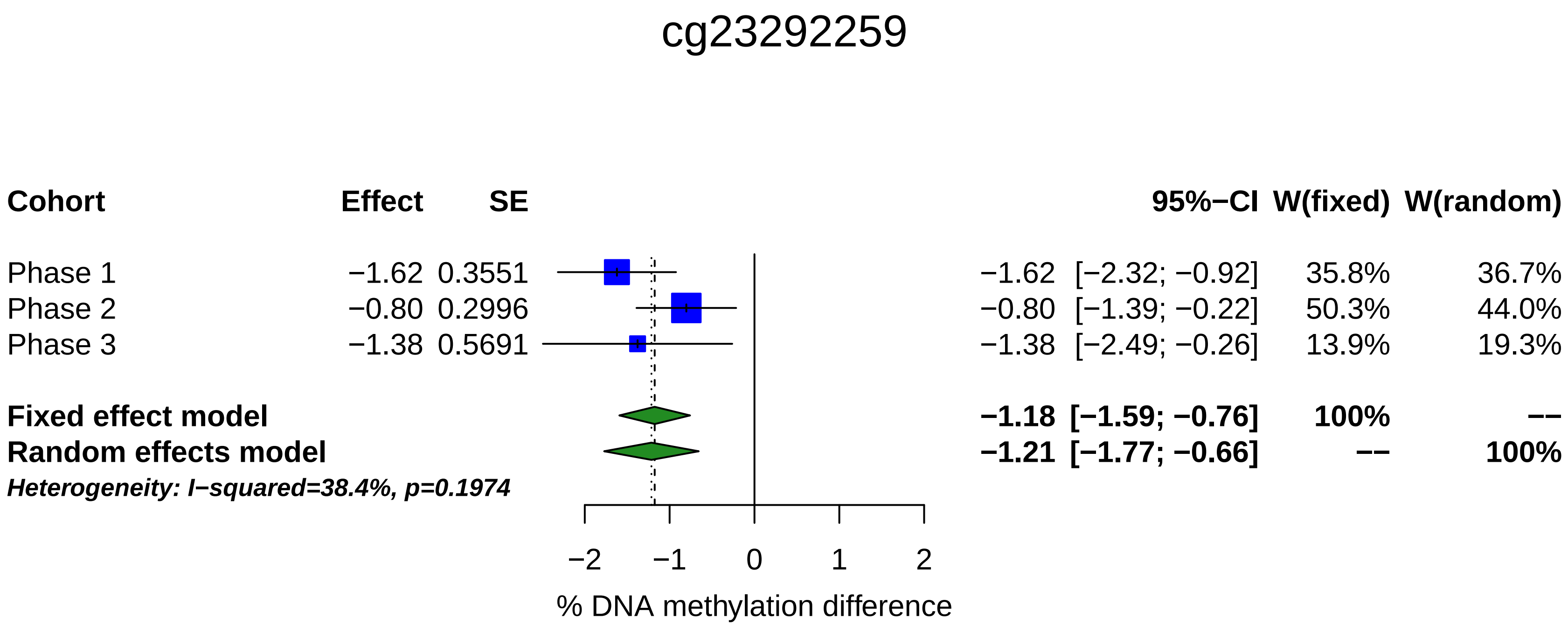
cg23292259
Cohort
Effect
SE
95%−CI
W(fixed)
W(random)
Phase 1 −1.62 0.3551 −1.62 [−2.32; −0.92] 35.8% 36.7%
Phase 2 −0.80 0.2996 −0.80 [−1.39; −0.22] 50.3% 44.0%
Phase 3 −1.38 0.5691 −1.38 [−2.49; −0.26] 13.9% 19.3%
Fixed effect model −1.18 [−1.59; −0.76] 100% −−
Random effects model −1.21 [−1.77; −0.66] −− 100%
Heterogeneity: I−squared=38.4%, p=0.1974
−2
−1
0
1
2
% DNA methylation difference

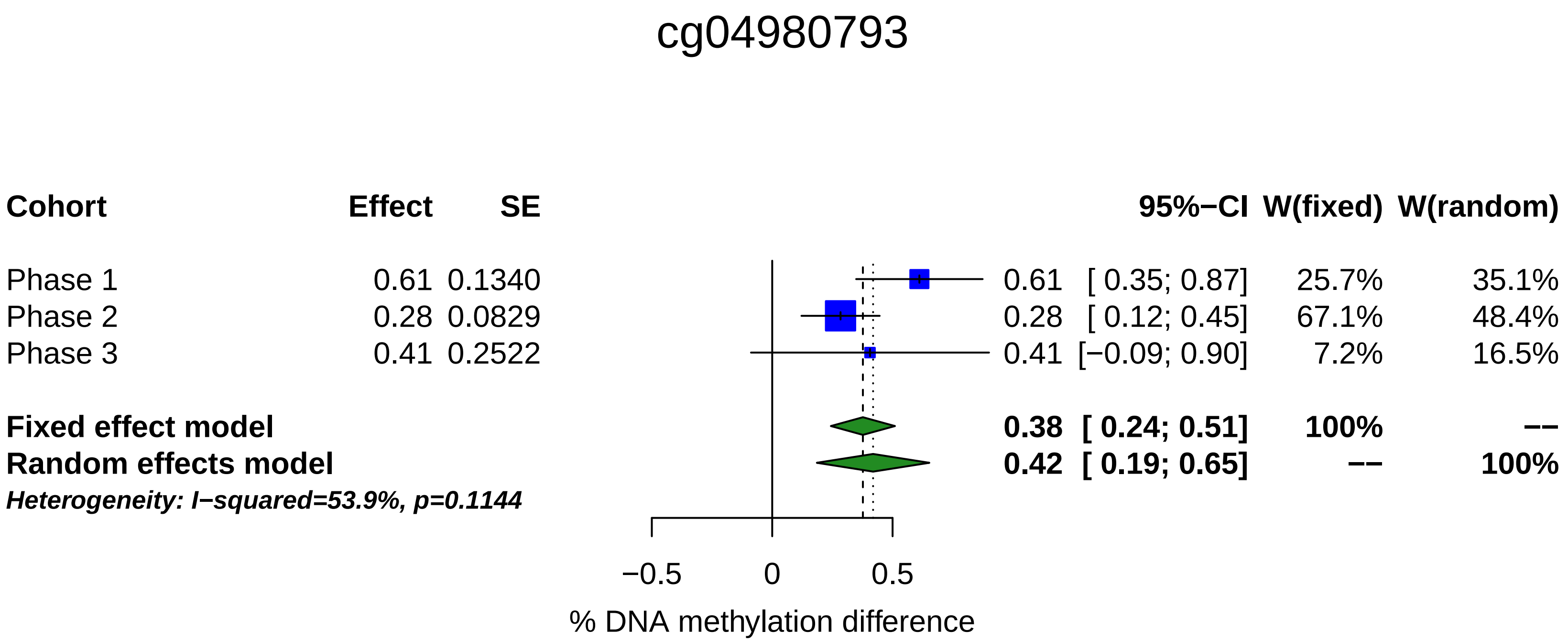
cg04980793
Cohort
Effect
SE
95%−CI
W(fixed)
W(random)
Phase 1 0.61 0.1340 0.61 [0.35; 0.87] 25.7% 35.1%
Phase 2 0.28 0.0829 0.28 [0.12; 0.45] 67.1% 48.4%
Phase 3 0.41 0.2522 0.41 [−0.09; 0.90] 7.2% 16.5%
Fixed effect model 0.38 [0.24; 0.51] 100% −−
Random effects model 0.42 [0.19; 0.65] −− 100%
Heterogeneity: I−squared=53.9%, p=0.1144
−0.5
0
0.5
% DNA methylation difference

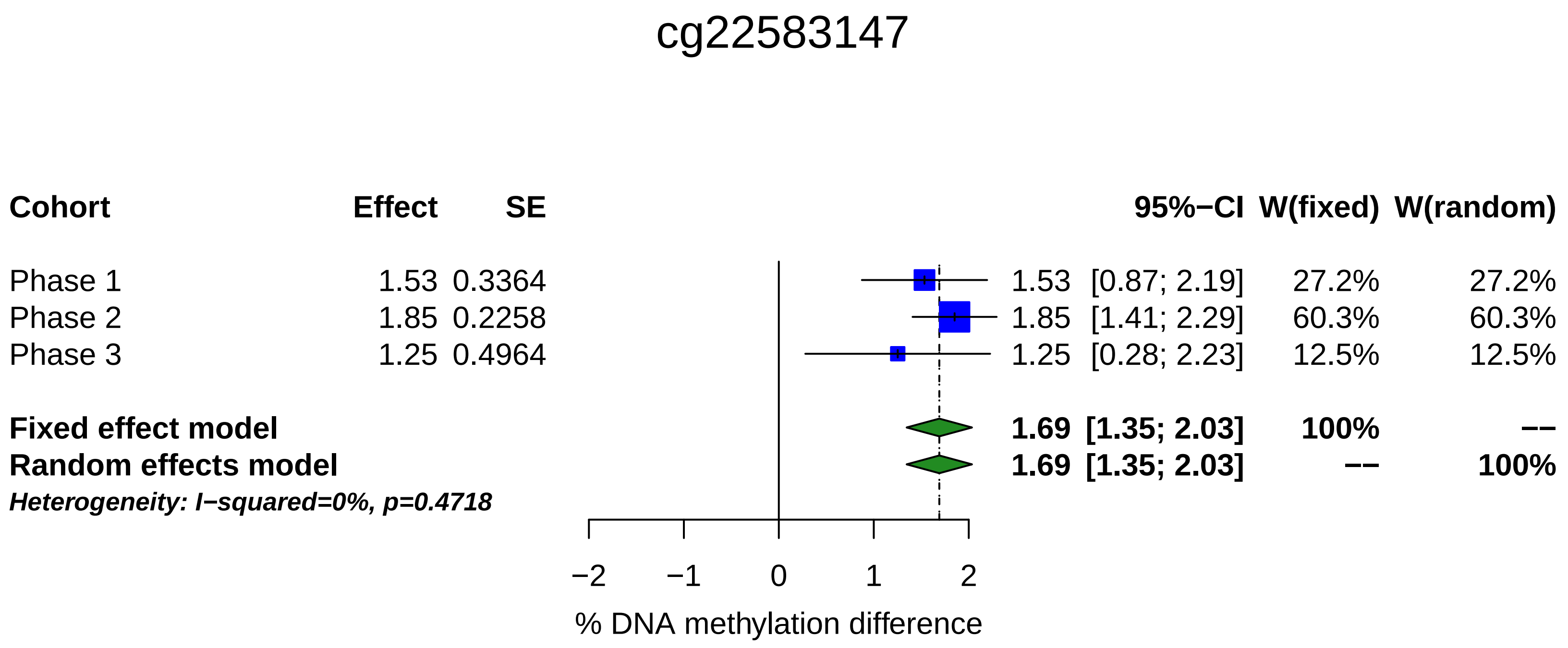
cg22583147
Cohort
Effect
SE
95%−CI
W(fixed)
W(random)
Phase 1 1.53 0.3364 1.53 [0.87; 2.19] 27.2% 27.2%
Phase 2 1.85 0.2258 1.85 [1.41; 2.29] 60.3% 60.3%
Phase 3 1.25 0.4964 1.25 [0.28; 2.23] 12.5% 12.5%
Fixed effect model 1.69 [1.35; 2.03] 100% −−
Random effects model 1.69 [1.35; 2.03] −− 100%
Heterogeneity: I−squared=0%, p=0.4718
−2
−1
0
1
2
% DNA methylation difference

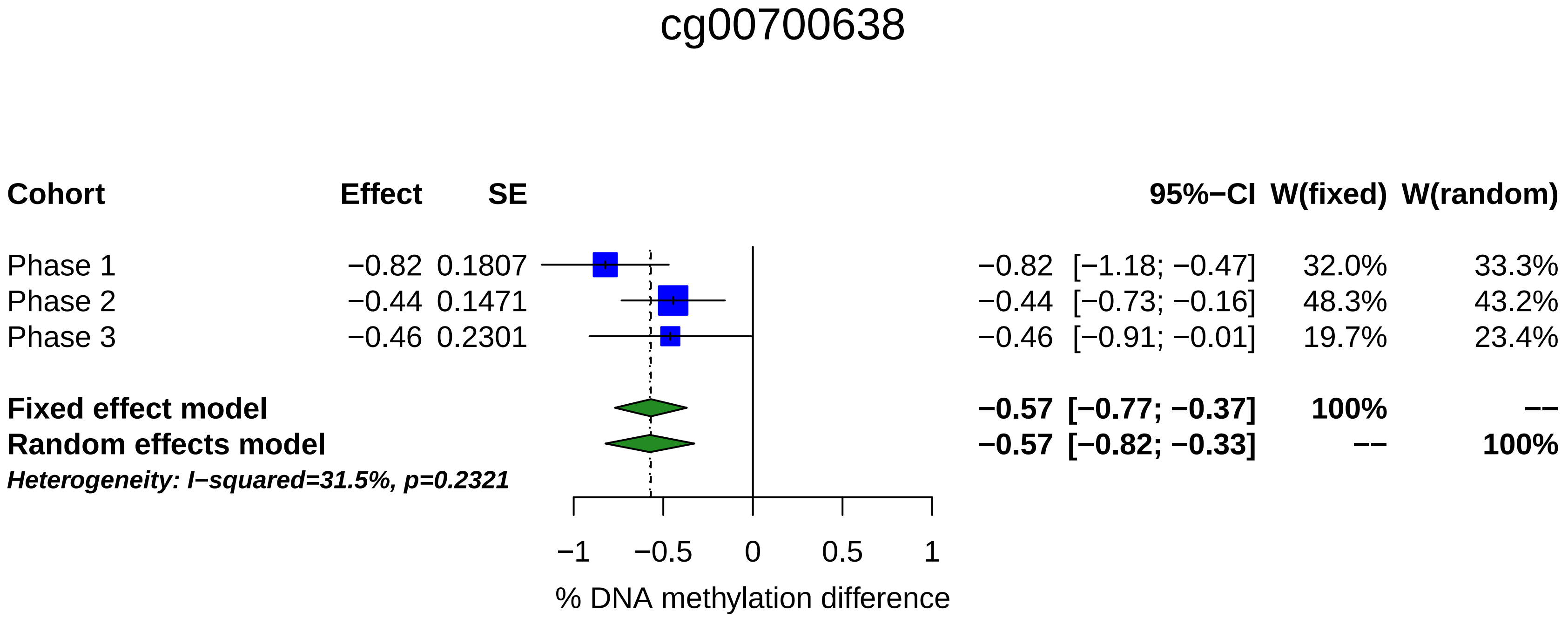
cg00700638
Cohort
Effect
SE
95%−CI
W(fixed)
W(random)
Phase 1 −0.82 0.1807 −0.82 [−1.18; −0.47] 32.0% 33.3%
Phase 2 −0.44 0.1471 −0.44 [−0.73; −0.16] 48.3% 43.2%
Phase 3 −0.46 0.2301 −0.46 [−0.91; −0.01] 19.7% 23.4%
Fixed effect model −0.57 [−0.77; −0.37] 100% −−
Random effects model −0.57 [−0.82; −0.33] −− 100%
Heterogeneity: I−squared=31.5%, p=0.2321
−1
−0.5
0
0.5
1
% DNA methylation difference

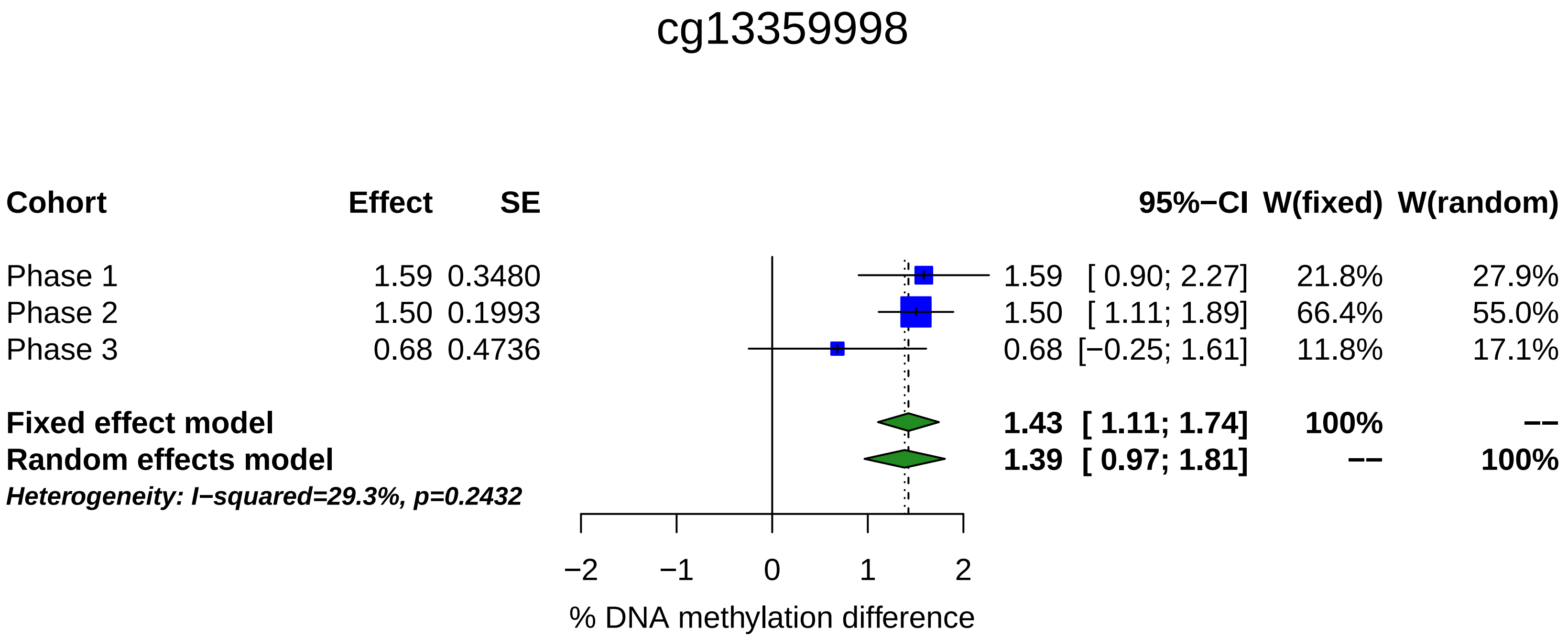
cg13359998
Cohort
Effect
SE
95%−CI
W(fixed)
W(random)
Phase 1 1.59 0.3480 1.59 [0.90; 2.27] 21.8% 27.9%
Phase 2 1.50 0.1993 1.50 [1.11; 1.89] 66.4% 55.0%
Phase 3 0.68 0.4736 0.68 [−0.25; 1.61] 11.8% 17.1%
Fixed effect model 1.43 [1.11; 1.74] 100% −−
Random effects model 1.39 [0.97; 1.81] −− 100%
Heterogeneity: I−squared=29.3%, p=0.2432
−2
−1
0
1
2
% DNA methylation difference

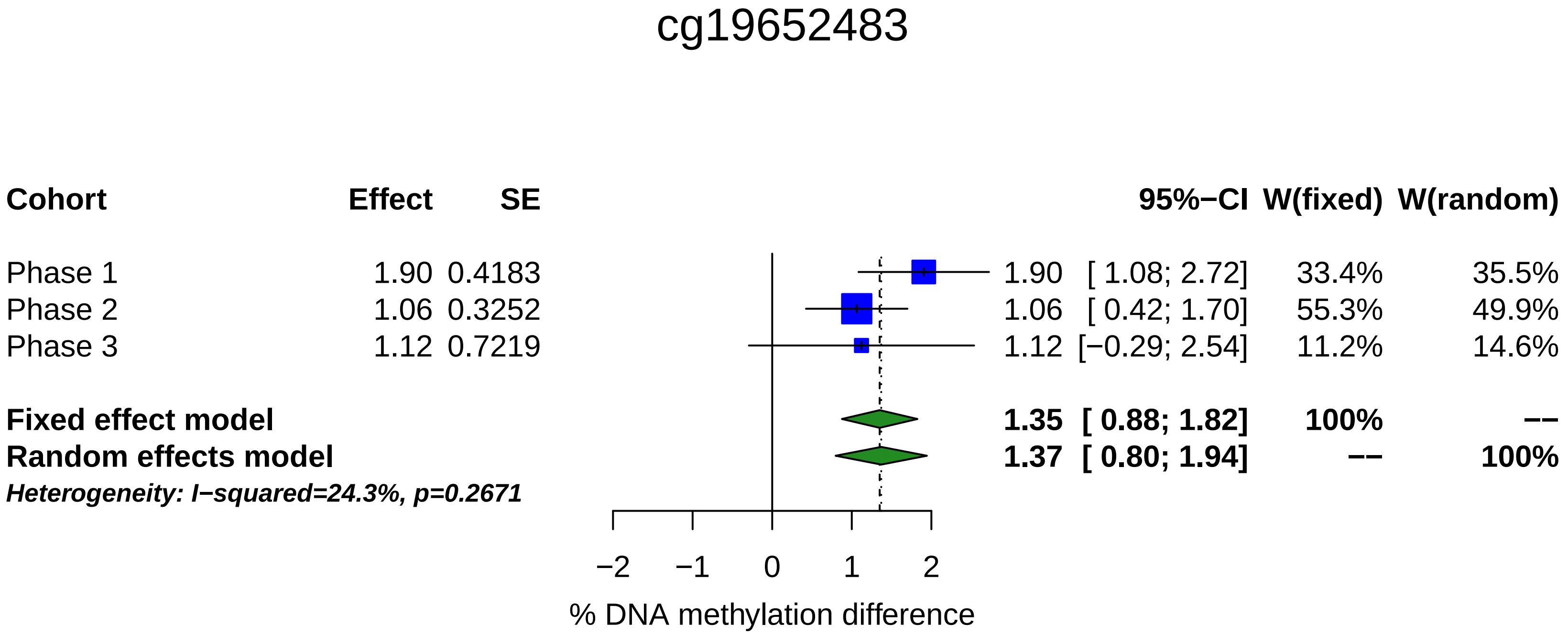
cg19652483
Cohort
Effect
SE
95%−CI
W(fixed)
W(random)
Phase 1 1.90 0.4183 1.90 [1.08; 2.72] 33.4% 35.5%
Phase 2 1.06 0.3252 1.06 [0.42; 1.70] 55.3% 49.9%
Phase 3 1.12 0.7219 1.12 [−0.29; 2.54] 11.2% 14.6%
Fixed effect model 1.35 [0.88; 1.82] 100% −−
Random effects model 1.37 [0.80; 1.94] −− 100%
Heterogeneity: I−squared=24.3%, p=0.2671
−2
−1
0
1
2
% DNA methylation difference

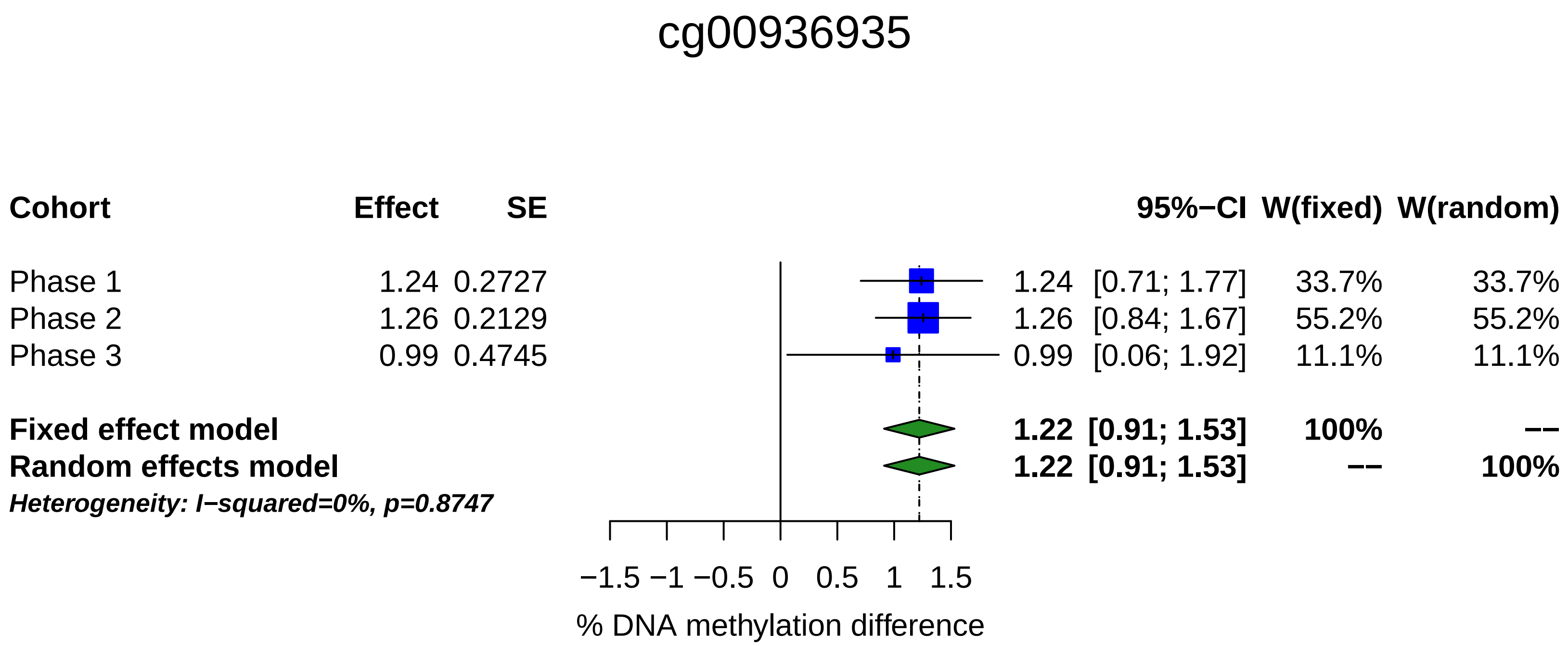
cg00936935
Cohort
Effect
SE
95%−CI
W(fixed)
W(random)
Phase 1 1.24 0.2727 1.24 [0.71; 1.77] 33.7% 33.7%
Phase 2 1.26 0.2129 1.26 [0.84; 1.67] 55.2% 55.2%
Phase 3 0.99 0.4745 0.99 [0.06; 1.92] 11.1% 11.1%
Fixed effect model 1.22 [0.91; 1.53] 100% −−
Random effects model 1.22 [0.91; 1.53] −− 100%
Heterogeneity: I−squared=0%, p=0.8747
−1.5 −1 −0.5 0 0.5 1 1.5
% DNA methylation difference

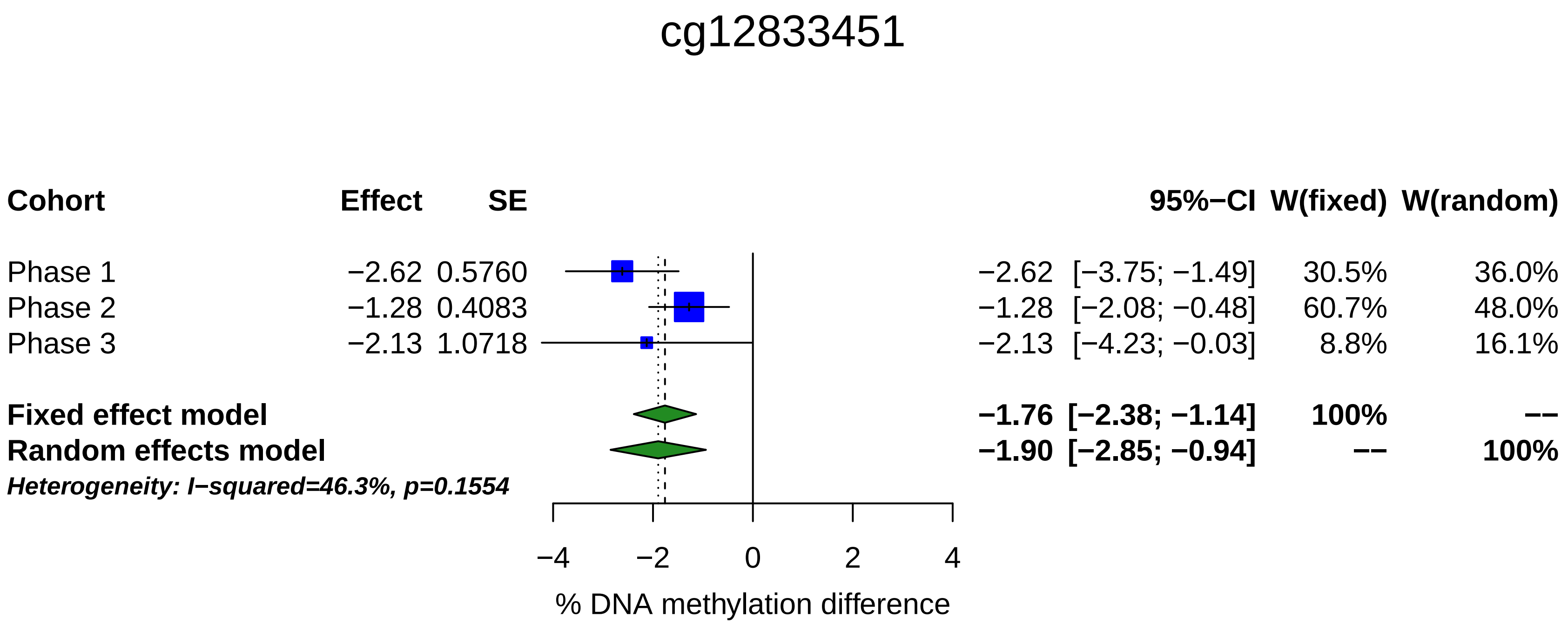
cg12833451
Cohort
Effect
SE
95%−CI
W(fixed)
W(random)
Phase 1 −2.62 0.5760 −2.62 [−3.75; −1.49] 30.5% 36.0%
Phase 2 −1.28 0.4083 −1.28 [−2.08; −0.48] 60.7% 48.0%
Phase 3 −2.13 1.0718 −2.13 [−4.23; −0.03] 8.8% 16.1%
Fixed effect model −1.76 [−2.38; −1.14] 100% −−
Random effects model −1.90 [−2.85; −0.94] −− 100%
Heterogeneity: I−squared=46.3%, p=0.1554
−4
−2
0
2
4
% DNA methylation difference

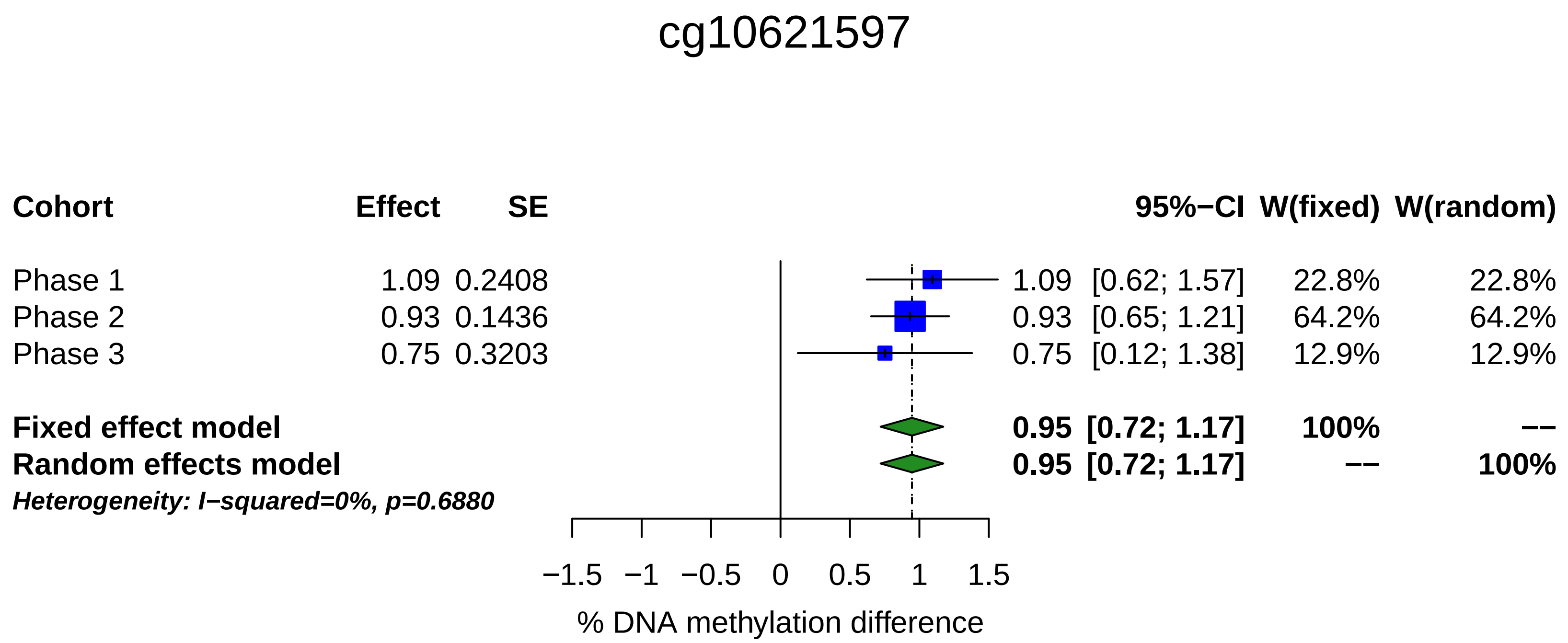
cg10621597
Cohort
Effect
SE
95%−CI
W(fixed)
W(random)
Phase 1 1.09 0.2408 1.09 [0.62; 1.57] 22.8% 22.8%
Phase 2 0.93 0.1436 0.93 [0.65; 1.21] 64.2% 64.2%
Phase 3 0.75 0.3203 0.75 [0.12; 1.38] 12.9% 12.9%
Fixed effect model 0.95 [0.72; 1.17] 100% −−
Random effects model 0.95 [0.72; 1.17] −− 100%
Heterogeneity: I−squared=0%, p=0.6880
−1.5
−1
−0.5
0
0.5
1
1.5
% DNA methylation difference

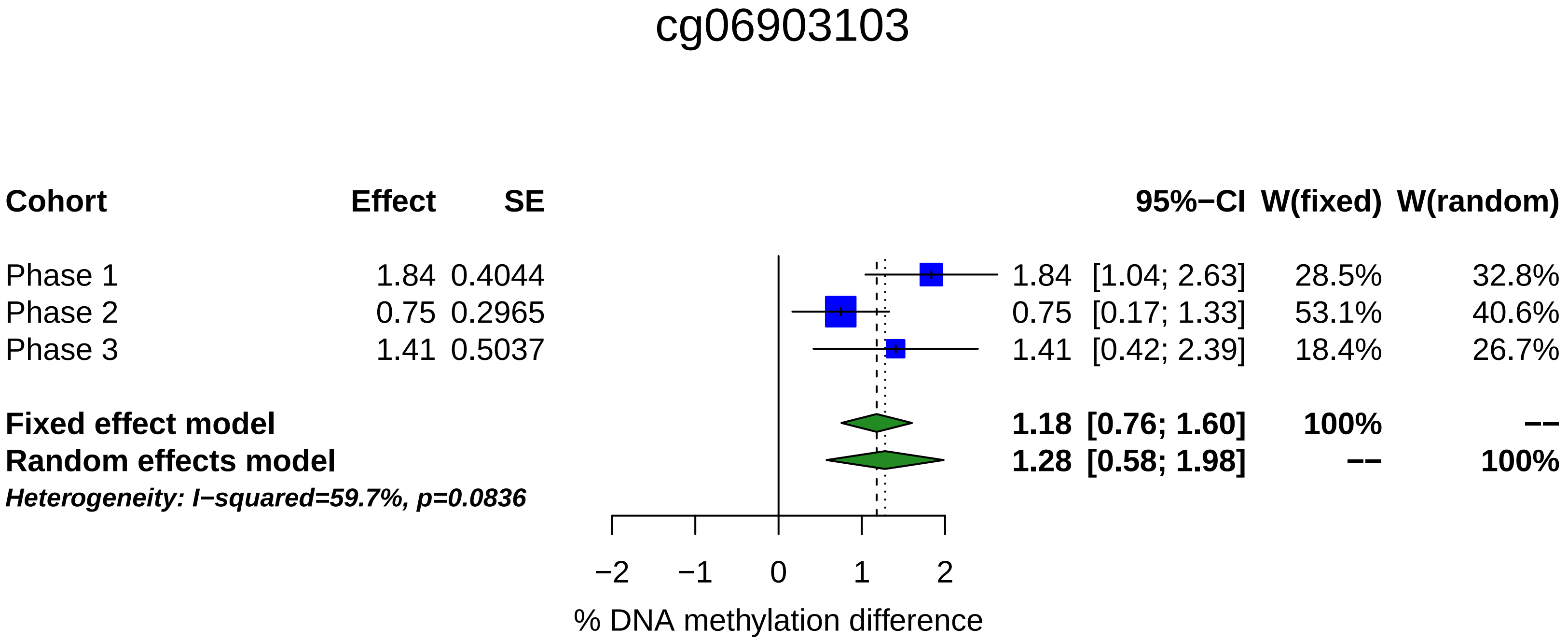
cg06903103
Cohort
Effect
SE
95%−CI
W(fixed)
W(random)
Phase 1 1.84 0.4044 1.84 [1.04; 2.63] 28.5% 32.8%
Phase 2 0.75 0.2965 0.75 [0.17; 1.33] 53.1% 40.6%
Phase 3 1.41 0.5037 1.41 [0.42; 2.39] 18.4% 26.7%
Fixed effect model 1.18 [0.76; 1.60] 100% −−
Random effects model 1.28 [0.58; 1.98] −− 100%
Heterogeneity: I−squared=59.7%, p=0.0836
−2
−1
0
1
2
% DNA methylation difference

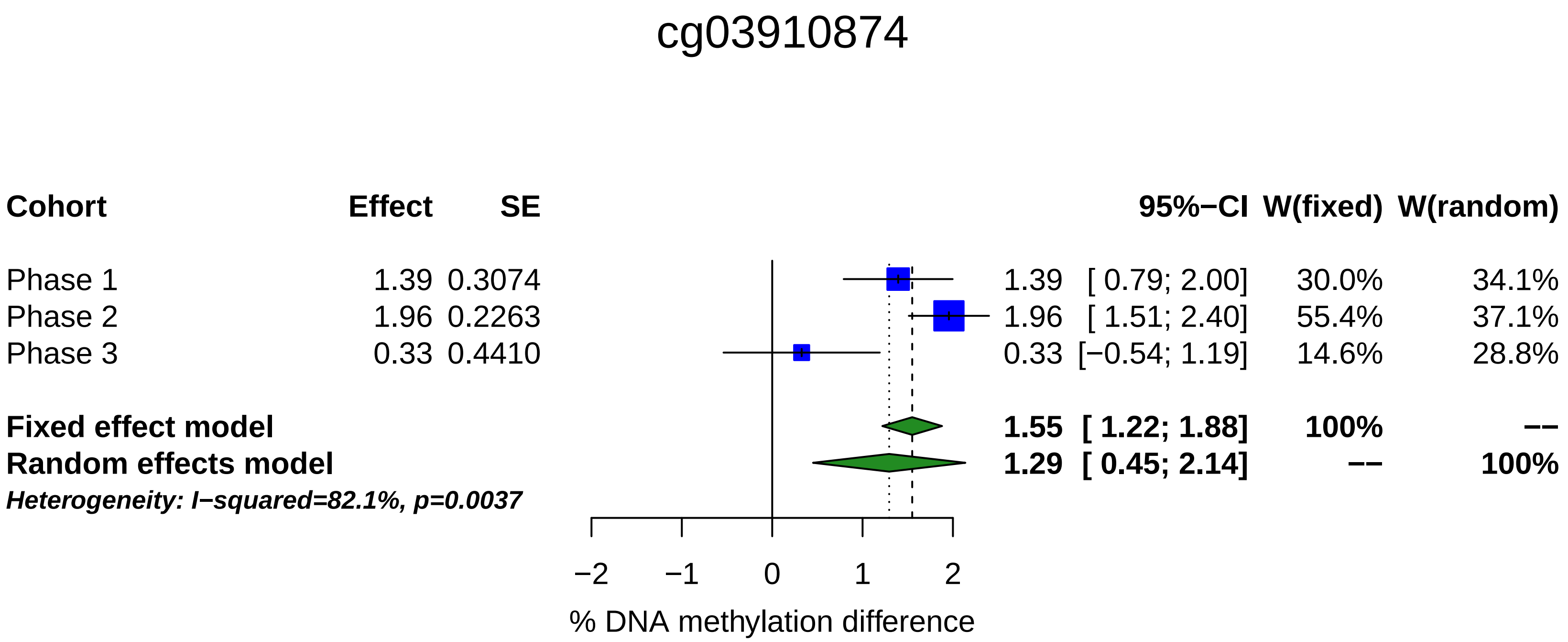
cg03910874
Cohort
Effect
SE
95%−CI
W(fixed)
W(random)
Phase 1
1.39
0.3074
1.39 [0.79; 2.00]
30.0%
34.1%
Phase 2
1.96
0.2263
1.96 [1.51; 2.40]
55.4%
37.1%
Phase 3
0.33
0.4410
0.33 [−0.54; 1.19]
14.6%
28.8%
Fixed effect model
1.55 [1.22; 1.88]
100%
−−
Random effects model
1.29 [0.45; 2.14]
−−
100%
Heterogeneity: I−squared=82.1%, p=0.0037
−2
−1
0
1
2
% DNA methylation difference

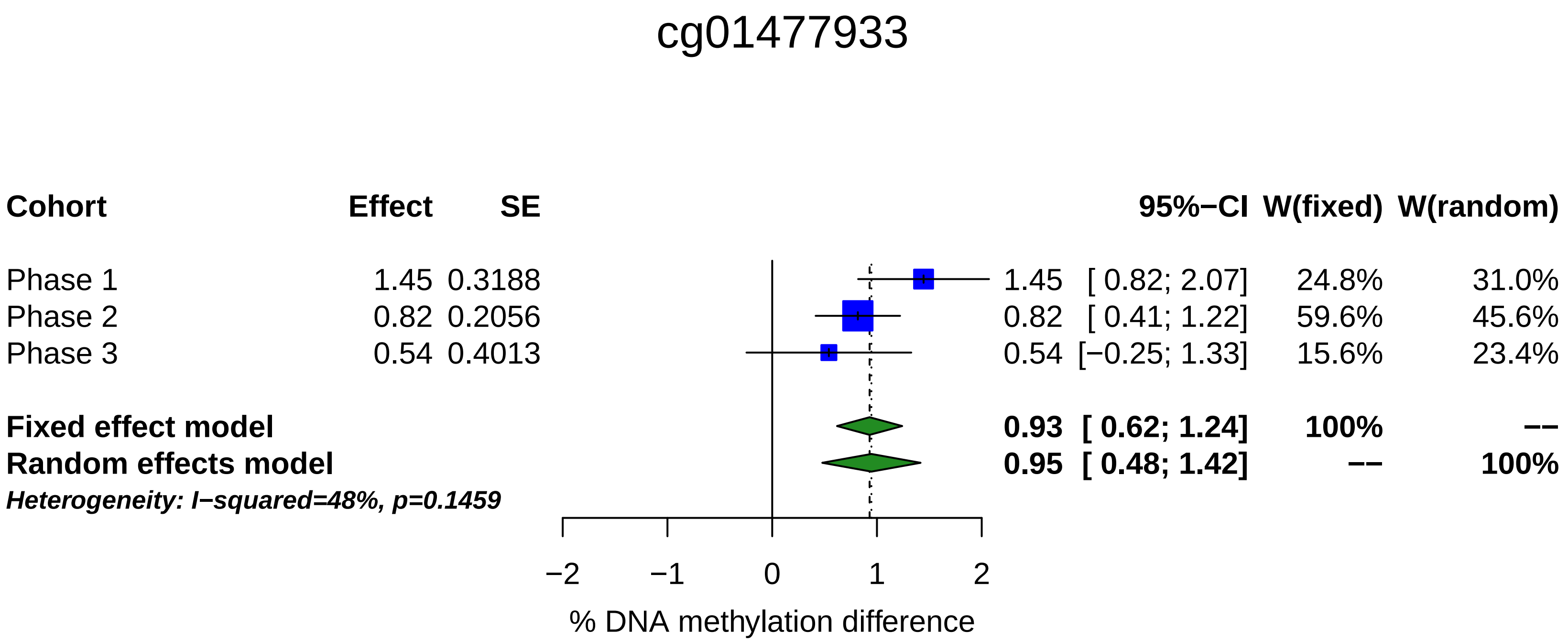
cg01477933
Cohort
Effect
SE
95%−CI
W(fixed)
W(random)
Phase 1 1.45 0.3188 1.45 [0.82; 2.07] 24.8% 31.0%
Phase 2 0.82 0.2056 0.82 [0.41; 1.22] 59.6% 45.6%
Phase 3 0.54 0.4013 0.54 [−0.25; 1.33] 15.6% 23.4%
Fixed effect model 0.93 [0.62; 1.24] 100% −−
Random effects model 0.95 [0.48; 1.42] −− 100%
Heterogeneity: I−squared=48%, p=0.1459
−2
−1
0
1
2
% DNA methylation difference

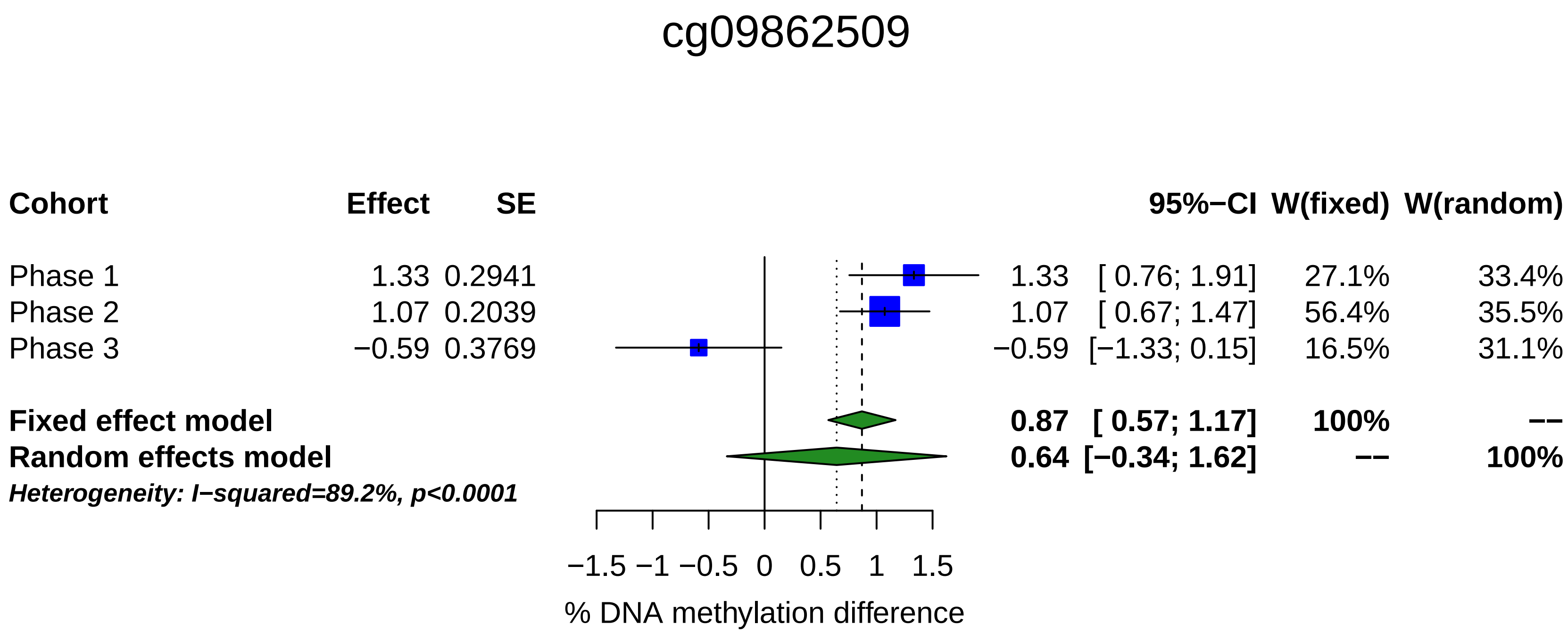
cg09862509
Cohort
Effect
SE
95%−CI
W(fixed)
W(random)
Phase 1 1.33 0.2941 1.33 [0.76; 1.91] 27.1% 33.4%
Phase 2 1.07 0.2039 1.07 [0.67; 1.47] 56.4% 35.5%
Phase 3 −0.59 0.3769 −0.59 [−1.33; 0.15] 16.5% 31.1%
Fixed effect model 0.87 [0.57; 1.17] 100% −−
Random effects model 0.64 [−0.34; 1.62] −− 100%
Heterogeneity: I−squared=89.2%, p<0.0001
−1.5 −1 −0.5 0 0.5 1 1.5
% DNA methylation difference

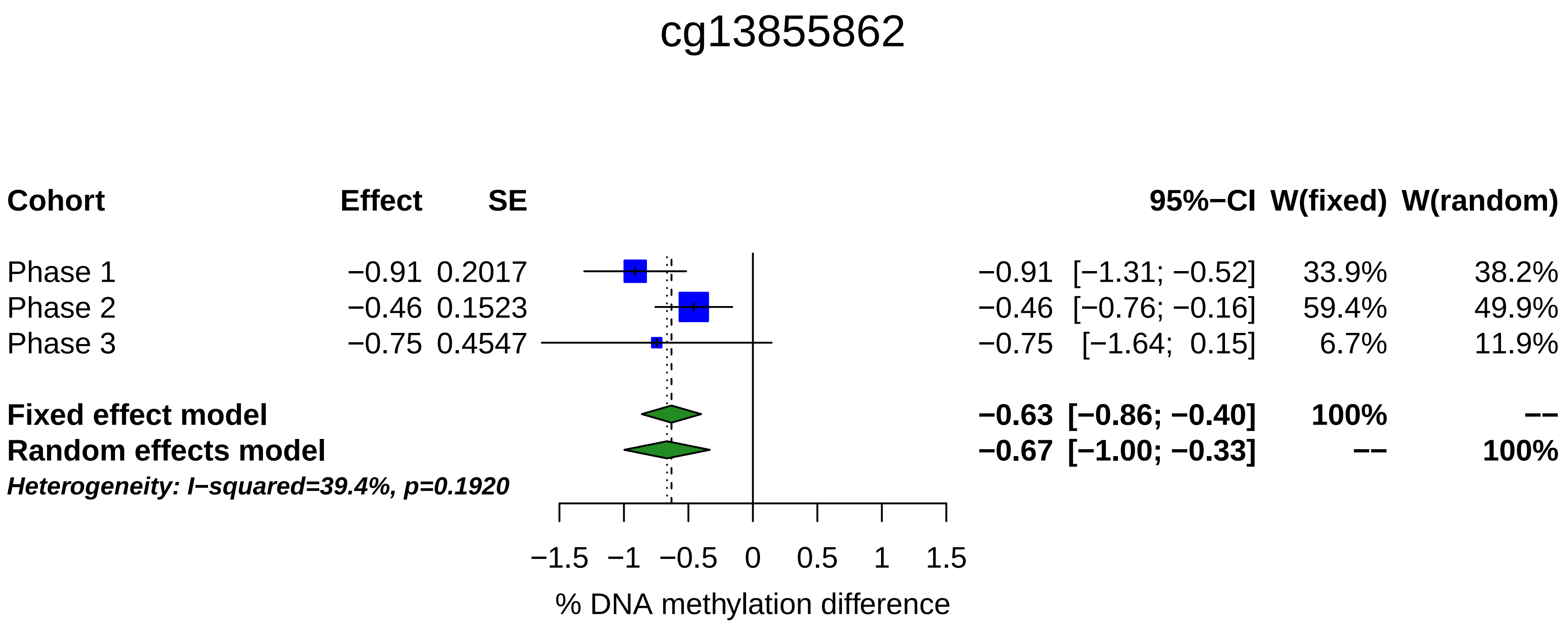
cg13855862
Cohort
Effect
SE
95%−CI
W(fixed)
W(random)
Phase 1 −0.91 0.2017 −0.91 [−1.31; −0.52] 33.9% 38.2%
Phase 2 −0.46 0.1523 −0.46 [−0.76; −0.16] 59.4% 49.9%
Phase 3 −0.75 0.4547 −0.75 [−1.64; 0.15] 6.7% 11.9%
Fixed effect model −0.63 [−0.86; −0.40] 100% −−
Random effects model −0.67 [−1.00; −0.33] −− 100%
Heterogeneity: I−squared=39.4%, p=0.1920
−1.5 −1 −0.5 0 0.5 1 1.5
% DNA methylation difference

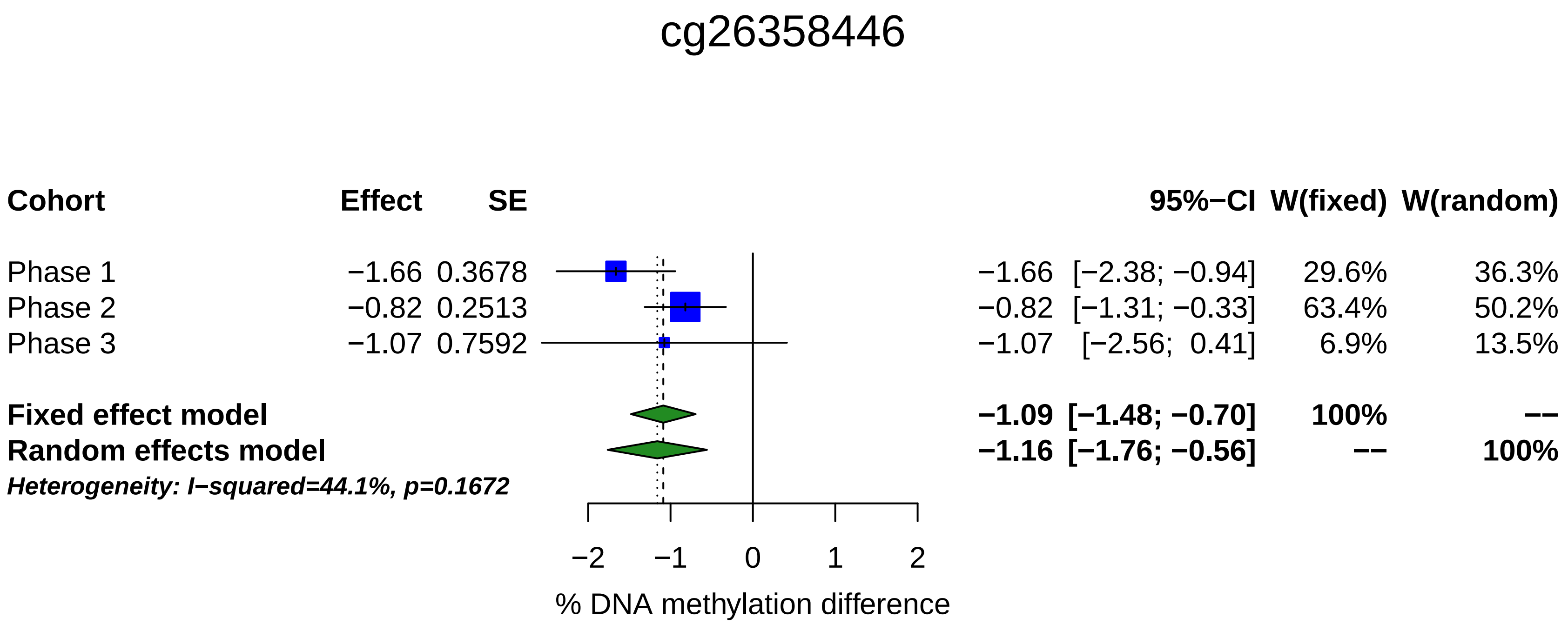
cg26358446
Cohort
Effect
SE
95%−CI
W(fixed)
W(random)
Phase 1 −1.66 0.3678 −1.66 [−2.38; −0.94] 29.6% 36.3%
Phase 2 −0.82 0.2513 −0.82 [−1.31; −0.33] 63.4% 50.2%
Phase 3 −1.07 0.7592 −1.07 [−2.56; 0.41] 6.9% 13.5%
Fixed effect model −1.09 [−1.48; −0.70] 100% --
Random effects model −1.16 [−1.76; −0.56] -- 100%
Heterogeneity: I−squared=44.1%, p=0.1672
−2
−1
0
1
2
% DNA methylation difference

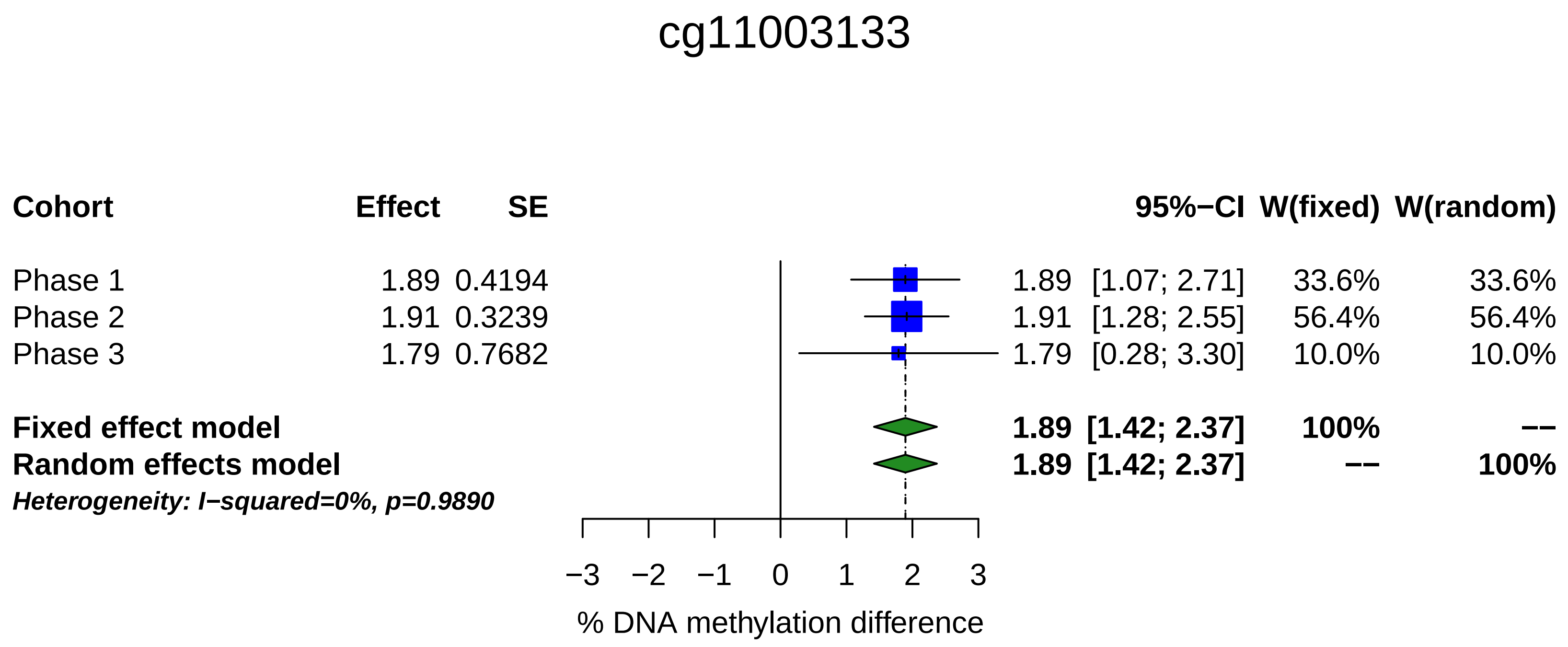
cg11003133
Cohort
Effect
SE
95%−CI
W(fixed)
W(random)
Phase 1 1.89 0.4194 1.89 [1.07; 2.71] 33.6% 33.6%
Phase 2 1.91 0.3239 1.91 [1.28; 2.55] 56.4% 56.4%
Phase 3 1.79 0.7682 1.79 [0.28; 3.30] 10.0% 10.0%
Fixed effect model 1.89 [1.42; 2.37] 100% −−
Random effects model 1.89 [1.42; 2.37] −− 100%
Heterogeneity: I−squared=0%, p=0.9890
−3 −2 −1 0 1 2 3
% DNA methylation difference

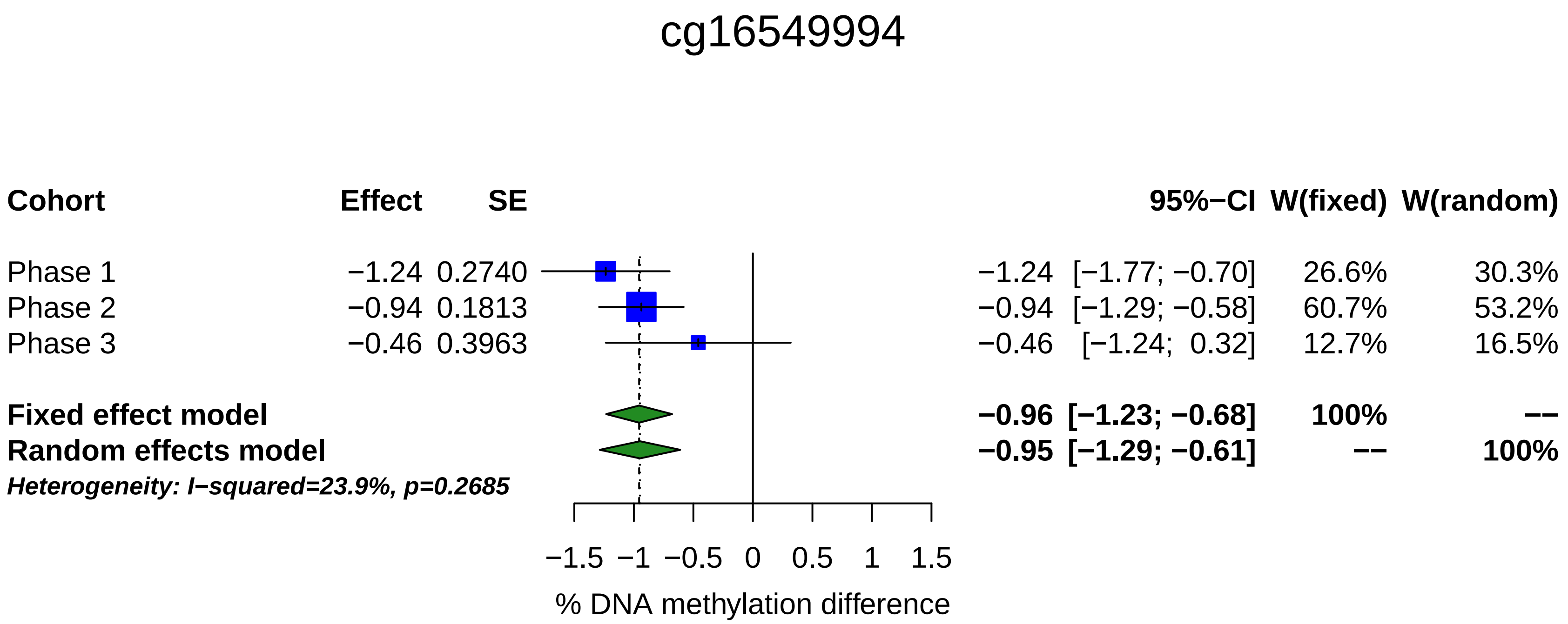
cg16549994
Cohort
Effect
SE
95%−CI
W(fixed)
W(random)
Phase 1 −1.24 0.2740 −1.24 [−1.77; −0.70] 26.6% 30.3%
Phase 2 −0.94 0.1813 −0.94 [−1.29; −0.58] 60.7% 53.2%
Phase 3 −0.46 0.3963 −0.46 [−1.24; 0.32] 12.7% 16.5%
Fixed effect model −0.96 [−1.23; −0.68] 100% −−
Random effects model −0.95 [−1.29; −0.61] −− 100%
Heterogeneity: I−squared=23.9%, p=0.2685
−1.5
−1
−0.5
0
0.5
1
1.5
% DNA methylation difference

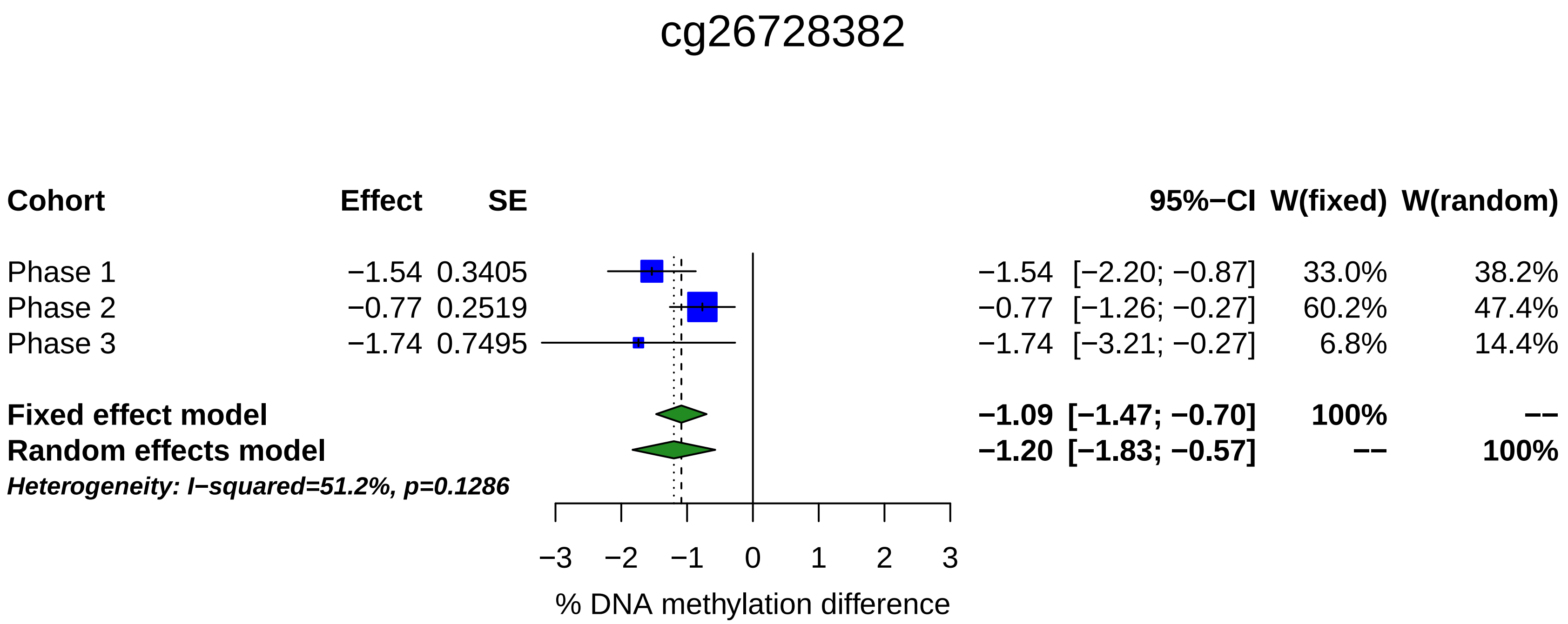
cg26728382
Cohort
Effect
SE
95%−CI
W(fixed)
W(random)
Phase 1 −1.54 0.3405 −1.54 [−2.20; −0.87] 33.0% 38.2%
Phase 2 −0.77 0.2519 −0.77 [−1.26; −0.27] 60.2% 47.4%
Phase 3 −1.74 0.7495 −1.74 [−3.21; −0.27] 6.8% 14.4%
Fixed effect model −1.09 [−1.47; −0.70] 100% −−
Random effects model −1.20 [−1.83; −0.57] −− 100%
Heterogeneity: I−squared=51.2%, p=0.1286
−3
−2
−1
0
1
2
3
% DNA methylation difference

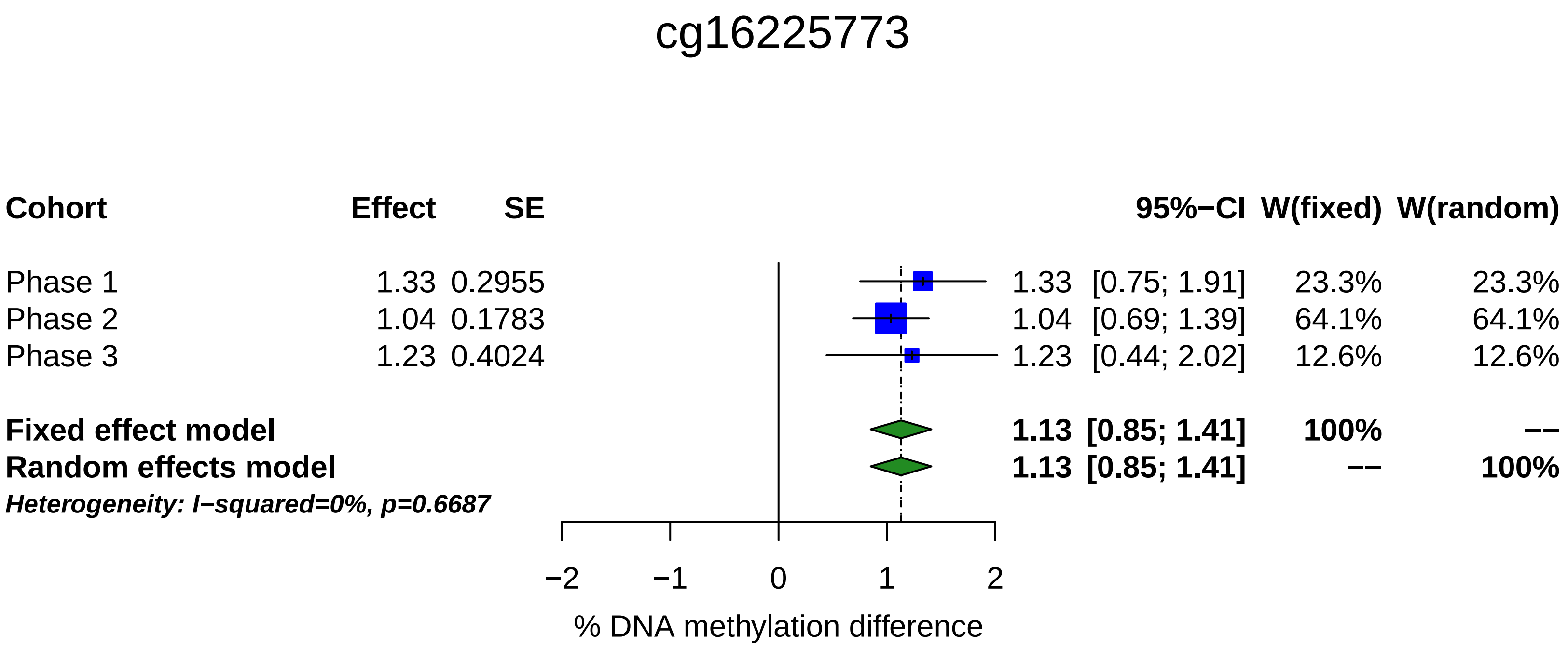
cg16225773
Cohort
Effect
SE
95%−CI
W(fixed)
W(random)
Phase 1 1.33 0.2955 1.33 [0.75; 1.91] 23.3% 23.3%
Phase 2 1.04 0.1783 1.04 [0.69; 1.39] 64.1% 64.1%
Phase 3 1.23 0.4024 1.23 [0.44; 2.02] 12.6% 12.6%
Fixed effect model 1.13 [0.85; 1.41] 100% −−
Random effects model 1.13 [0.85; 1.41] −− 100%
Heterogeneity: I−squared=0%, p=0.6687
−2
−1
0
1
2
% DNA methylation difference

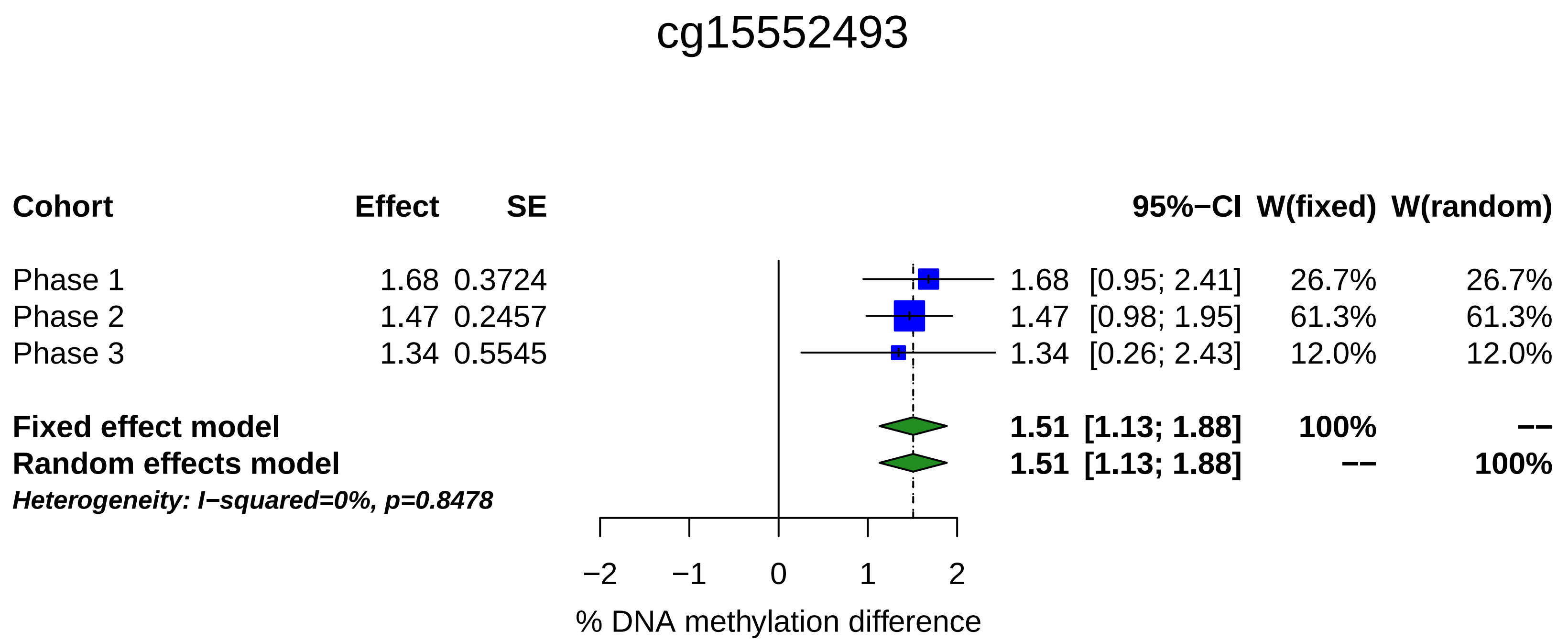
cg15552493
Cohort
Effect
SE
95%−CI
W(fixed)
W(random)
Phase 1 1.68 0.3724 1.68 [0.95; 2.41] 26.7% 26.7%
Phase 2 1.47 0.2457 1.47 [0.98; 1.95] 61.3% 61.3%
Phase 3 1.34 0.5545 1.34 [0.26; 2.43] 12.0% 12.0%
Fixed effect model 1.51 [1.13; 1.88] 100% −−
Random effects model 1.51 [1.13; 1.88] −− 100%
Heterogeneity: I−squared=0%, p=0.8478
−2
−1
0
1
2
% DNA methylation difference

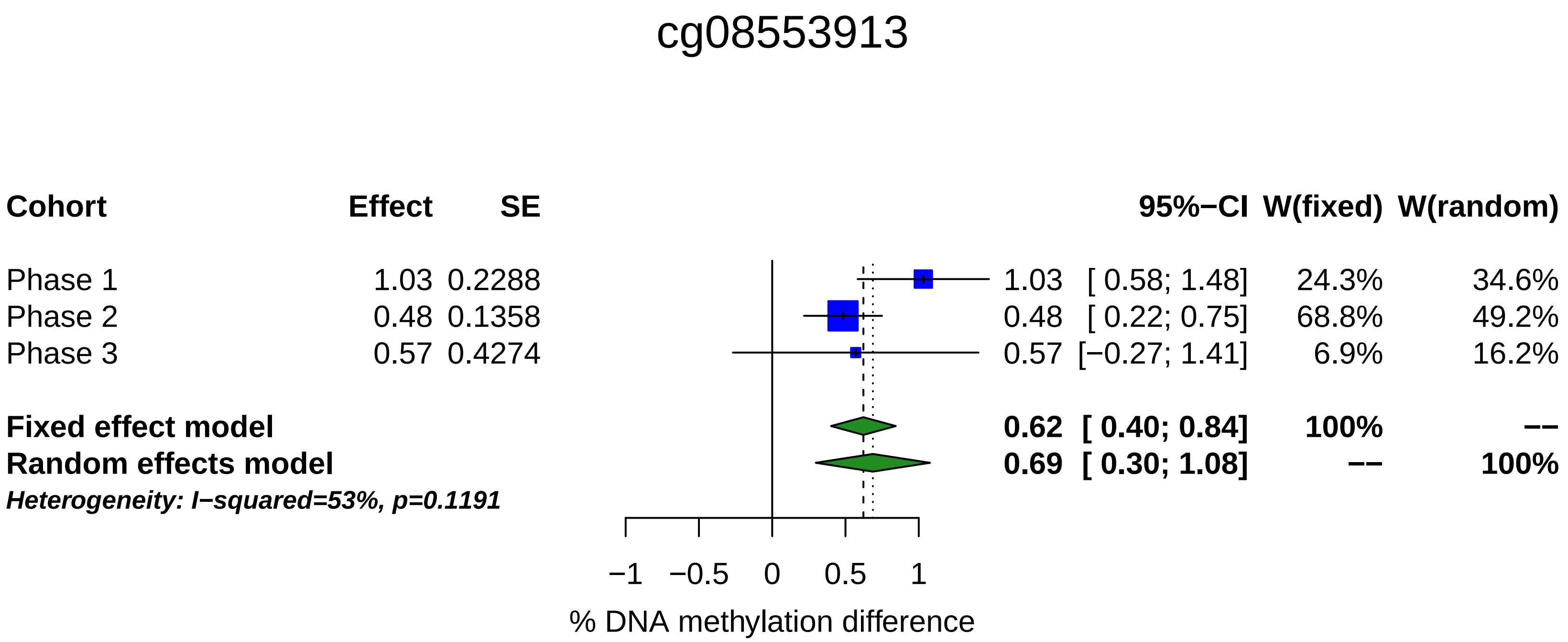
cg08553913
Cohort
Effect
SE
95%−CI
W(fixed)
W(random)
Phase 1 1.03 0.2288 1.03 [0.58; 1.48] 24.3% 34.6%
Phase 2 0.48 0.1358 0.48 [0.22; 0.75] 68.8% 49.2%
Phase 3 0.57 0.4274 0.57 [−0.27; 1.41] 6.9% 16.2%
Fixed effect model 0.62 [0.40; 0.84] 100% −−
Random effects model 0.69 [0.30; 1.08] −− 100%
Heterogeneity: I−squared=53%, p=0.1191
−1
−0.5
0
0.5
1
% DNA methylation difference

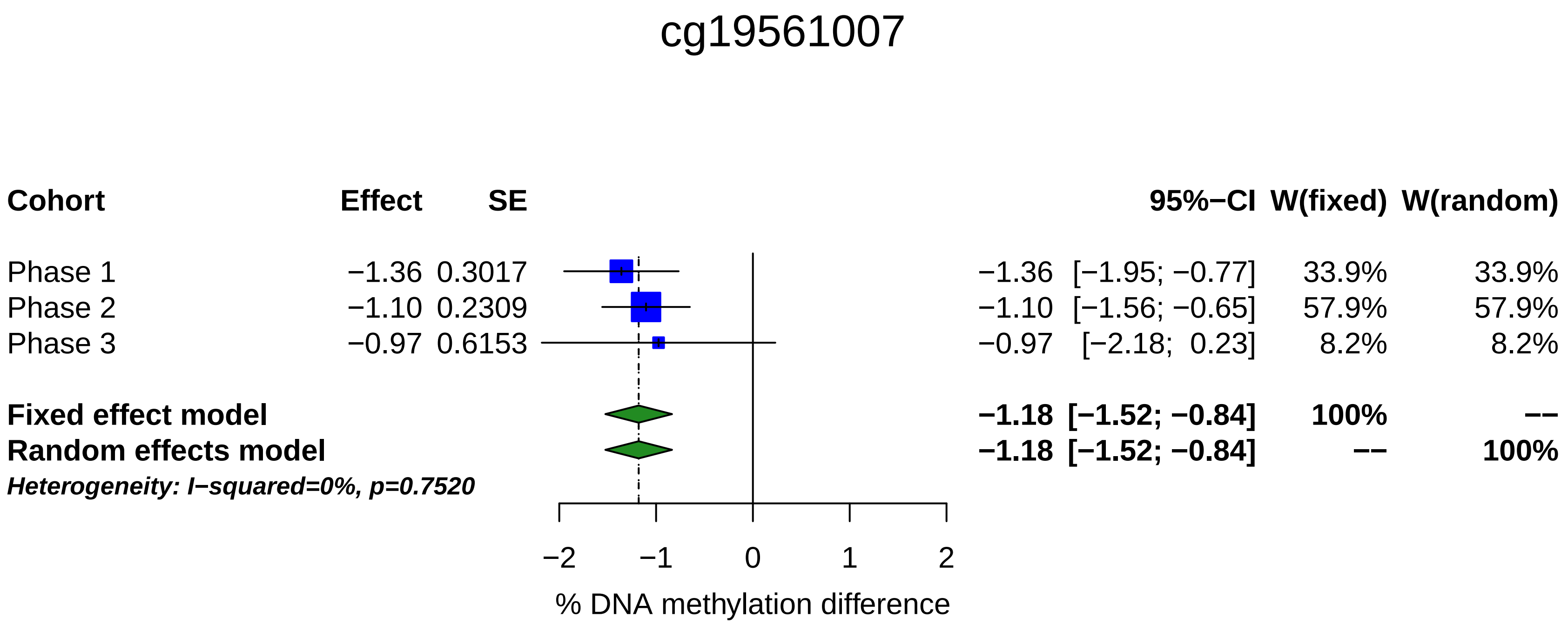
cg19561007
Cohort
Effect
SE
95%−CI
W(fixed)
W(random)
Phase 1 −1.36 0.3017 −1.36 [−1.95; −0.77] 33.9% 33.9%
Phase 2 −1.10 0.2309 −1.10 [−1.56; −0.65] 57.9% 57.9%
Phase 3 −0.97 0.6153 −0.97 [−2.18; 0.23] 8.2% 8.2%
Fixed effect model −1.18 [−1.52; −0.84] 100% −−
Random effects model −1.18 [−1.52; −0.84] −− 100%
Heterogeneity: I−squared=0%, p=0.7520
−2
−1
0
1
2
% DNA methylation difference

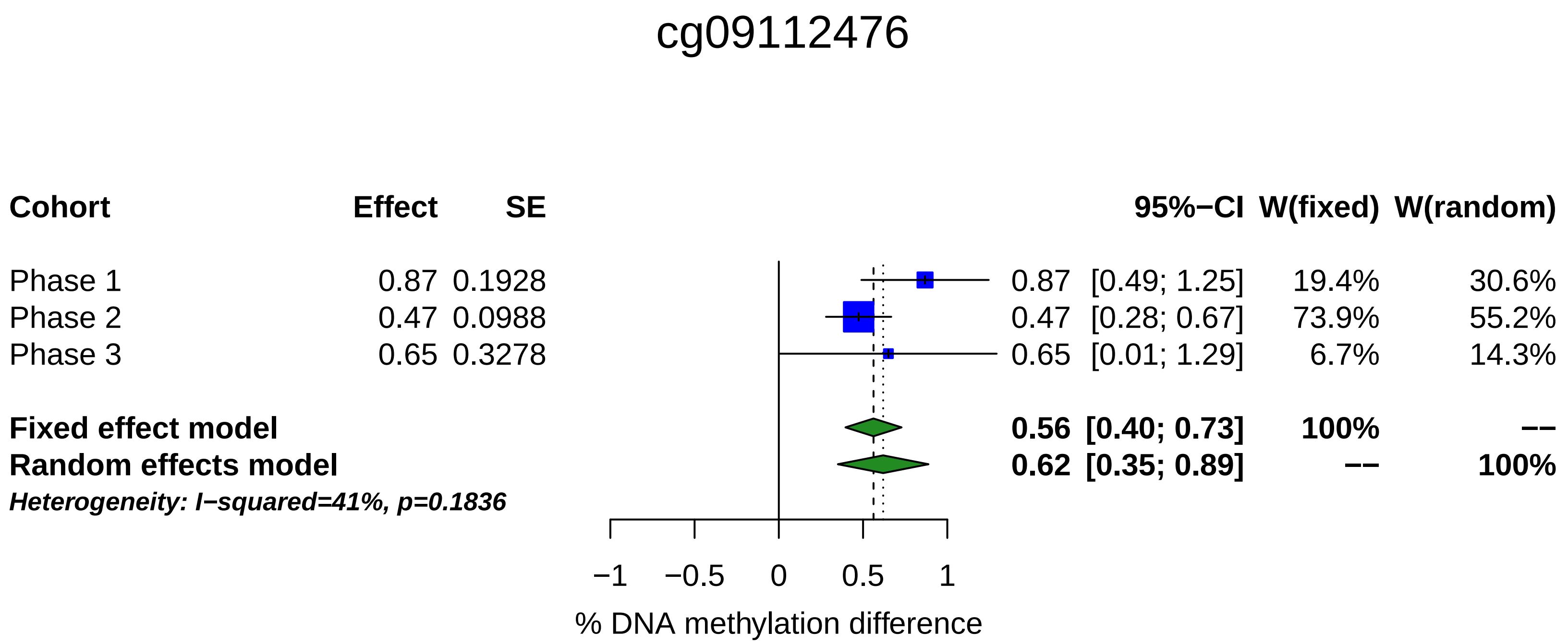

cg09112476
Cohort
Effect
SE
95%−CI
W(fixed)
W(random)
Phase 1 0.87 0.1928 0.87 [0.49; 1.25] 19.4% 30.6%
Phase 2 0.47 0.0988 0.47 [0.28; 0.67] 73.9% 55.2%
Phase 3 0.65 0.3278 0.65 [0.01; 1.29] 6.7% 14.3%
Fixed effect model 0.56 [0.40; 0.73] 100% −−
Random effects model 0.62 [0.35; 0.89] −− 100%
Heterogeneity: I−squared=41%, p=0.1836
−1
−0.5
0
0.5
1
% DNA methylation difference

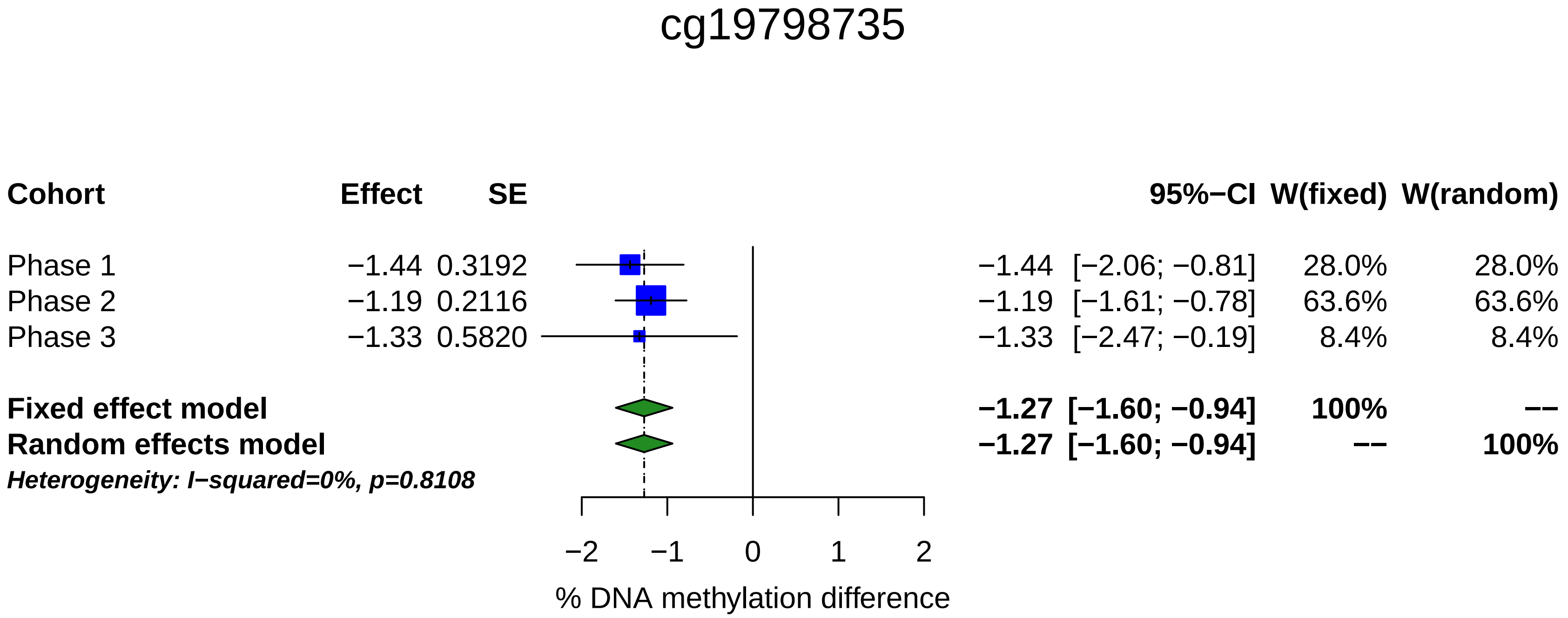
cg19798735
Cohort
Effect
SE
95%−CI
W(fixed)
W(random)
Phase 1 −1.44 0.3192 −1.44 [−2.06; −0.81] 28.0% 28.0%
Phase 2 −1.19 0.2116 −1.19 [−1.61; −0.78] 63.6% 63.6%
Phase 3 −1.33 0.5820 −1.33 [−2.47; −0.19] 8.4% 8.4%
Fixed effect model −1.27 [−1.60; −0.94] 100% −−
Random effects model −1.27 [−1.60; −0.94] −− 100%
Heterogeneity: I−squared=0%, p=0.8108
−2
−1
0
1
2
% DNA methylation difference

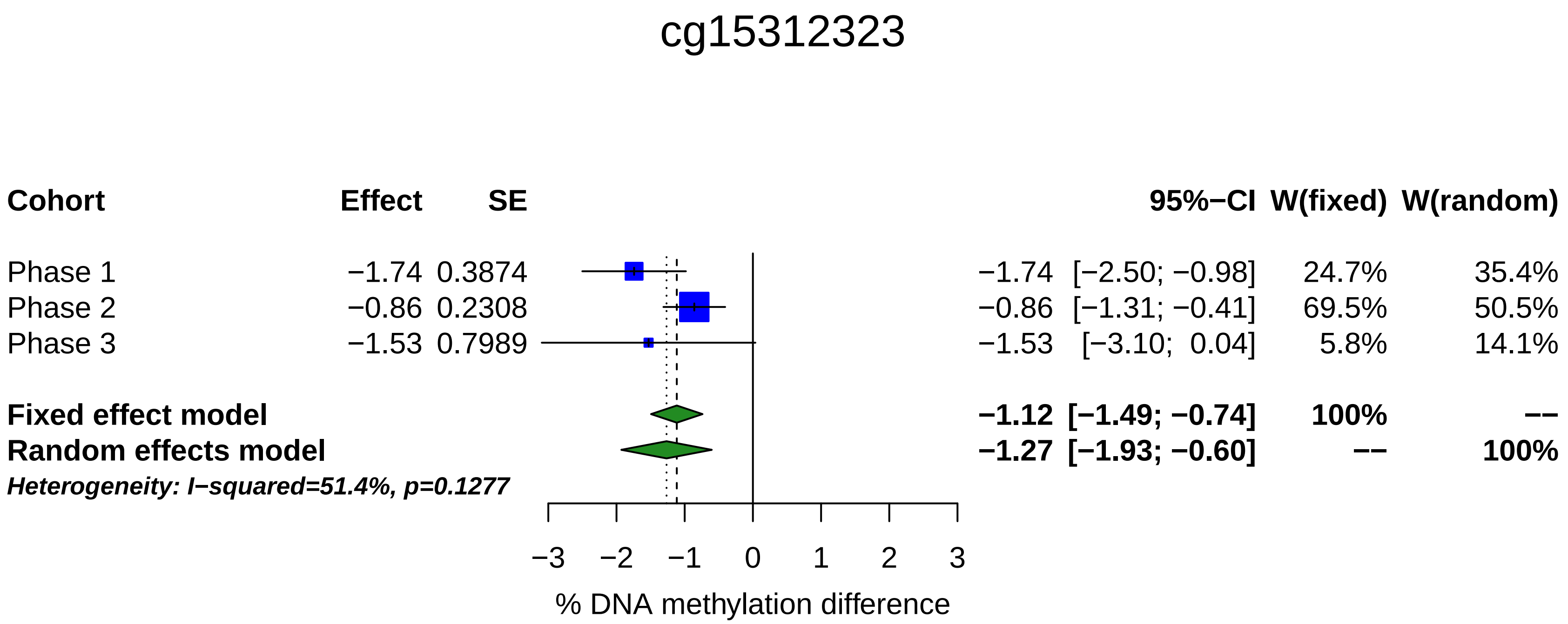
cg15312323
Cohort
Effect
SE
95%−CI
W(fixed)
W(random)
Phase 1 −1.74 0.3874 −1.74 [−2.50; −0.98] 24.7% 35.4%
Phase 2 −0.86 0.2308 −0.86 [−1.31; −0.41] 69.5% 50.5%
Phase 3 −1.53 0.7989 −1.53 [−3.10; 0.04] 5.8% 14.1%
Fixed effect model −1.12 [−1.49; −0.74] 100% −−
Random effects model −1.27 [−1.93; −0.60] −− 100%
Heterogeneity: I−squared=51.4%, p=0.1277
−3
−2
−1
0
1
2
3
% DNA methylation difference

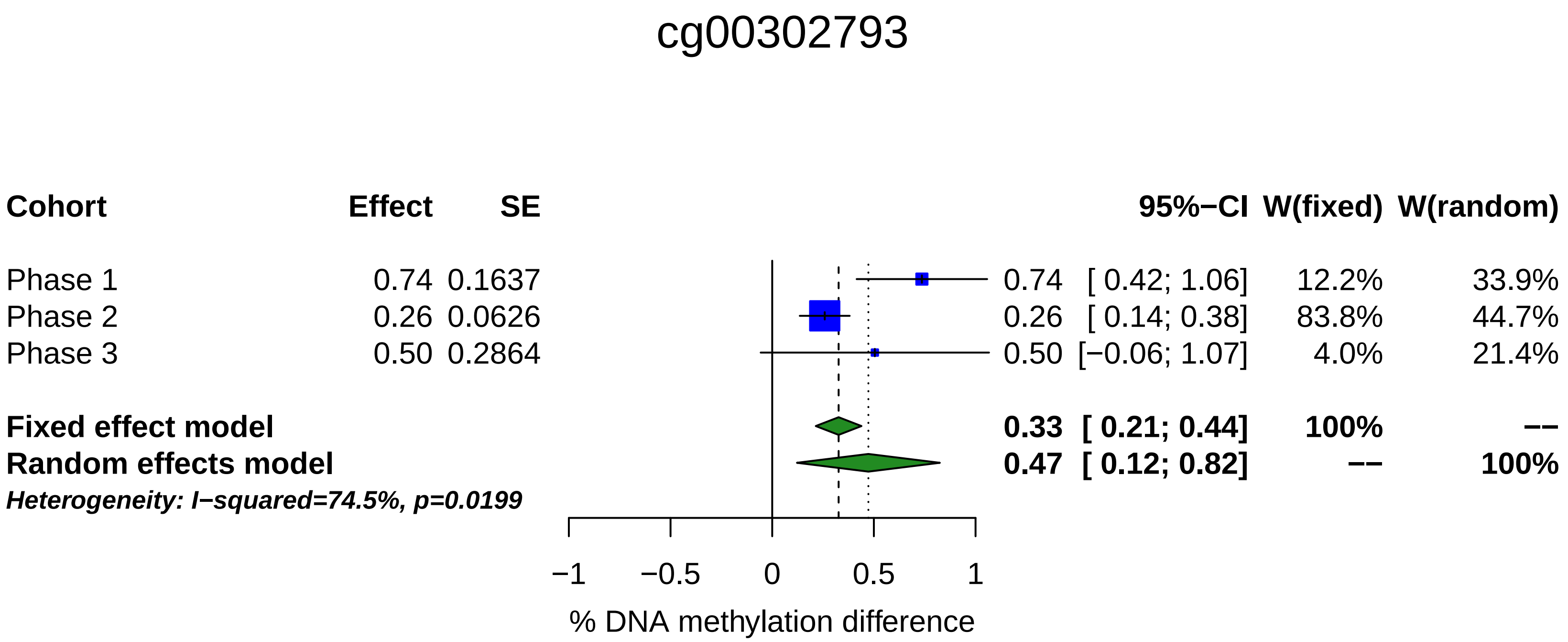
cg00302793
Cohort
Effect
SE
95%−CI
W(fixed)
W(random)
Phase 1 0.74 0.1637 0.74 [0.42; 1.06] 12.2% 33.9%
Phase 2 0.26 0.0626 0.26 [0.14; 0.38] 83.8% 44.7%
Phase 3 0.50 0.2864 0.50 [−0.06; 1.07] 4.0% 21.4%
Fixed effect model 0.33 [0.21; 0.44] 100% −−
Random effects model 0.47 [0.12; 0.82] −− 100%
Heterogeneity: I−squared=74.5%, p=0.0199
−1
−0.5
0
0.5
1
% DNA methylation difference

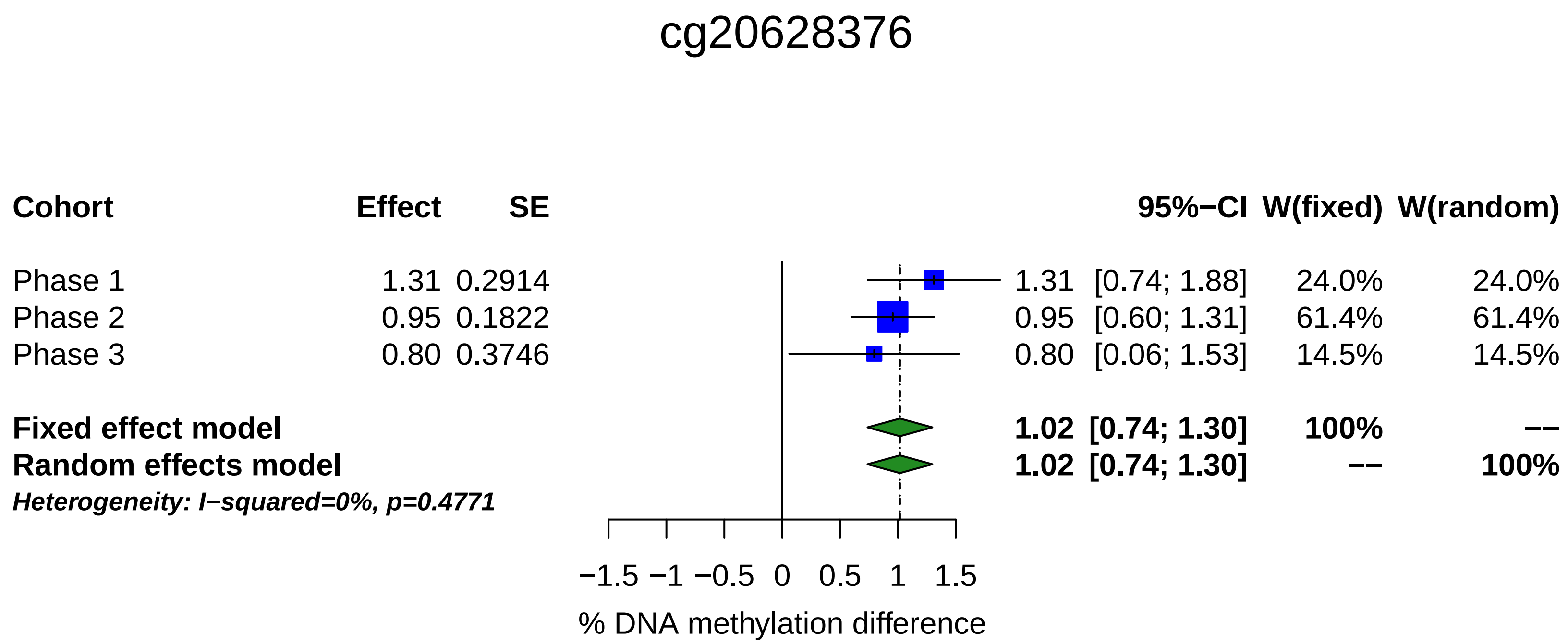
cg20628376
Cohort
Effect
SE
95%−CI
W(fixed)
W(random)
Phase 1 1.31 0.2914 1.31 [0.74; 1.88] 24.0% 24.0%
Phase 2 0.95 0.1822 0.95 [0.60; 1.31] 61.4% 61.4%
Phase 3 0.80 0.3746 0.80 [0.06; 1.53] 14.5% 14.5%
Fixed effect model 1.02 [0.74; 1.30] 100% −−
Random effects model 1.02 [0.74; 1.30] −− 100%
Heterogeneity: I−squared=0%, p=0.4771
−1.5 −1 −0.5 0 0.5 1 1.5
% DNA methylation difference

cg26712188

Cohort	Effect	SE	95%−CI	W(fixed)	W(random)
Phase 1	−1.82	0.4063	−1.82 [−2.62; −1.03]	28.1%	35.7%
Phase 2	−0.80	0.2703	−0.80 [−1.33; −0.27]	63.4%	41.1%
Phase 3	−2.54	0.7365	−2.54 [−3.99; −1.10]	8.5%	23.1%
**Fixed effect model**			**−1.23 [−1.66; −0.81]**	**100%**	**−−**
**Random effects model**			**−1.57 [−2.54; −0.59]**	**−−**	**100%**

Heterogeneity: I−squared=74.6%, p=0.0196

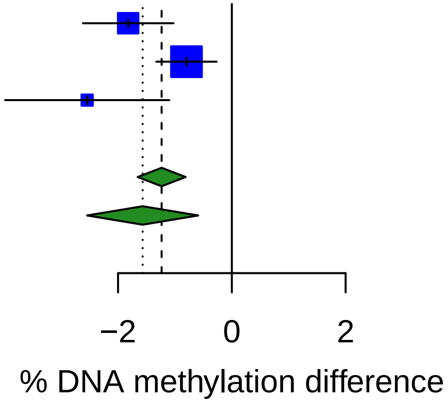

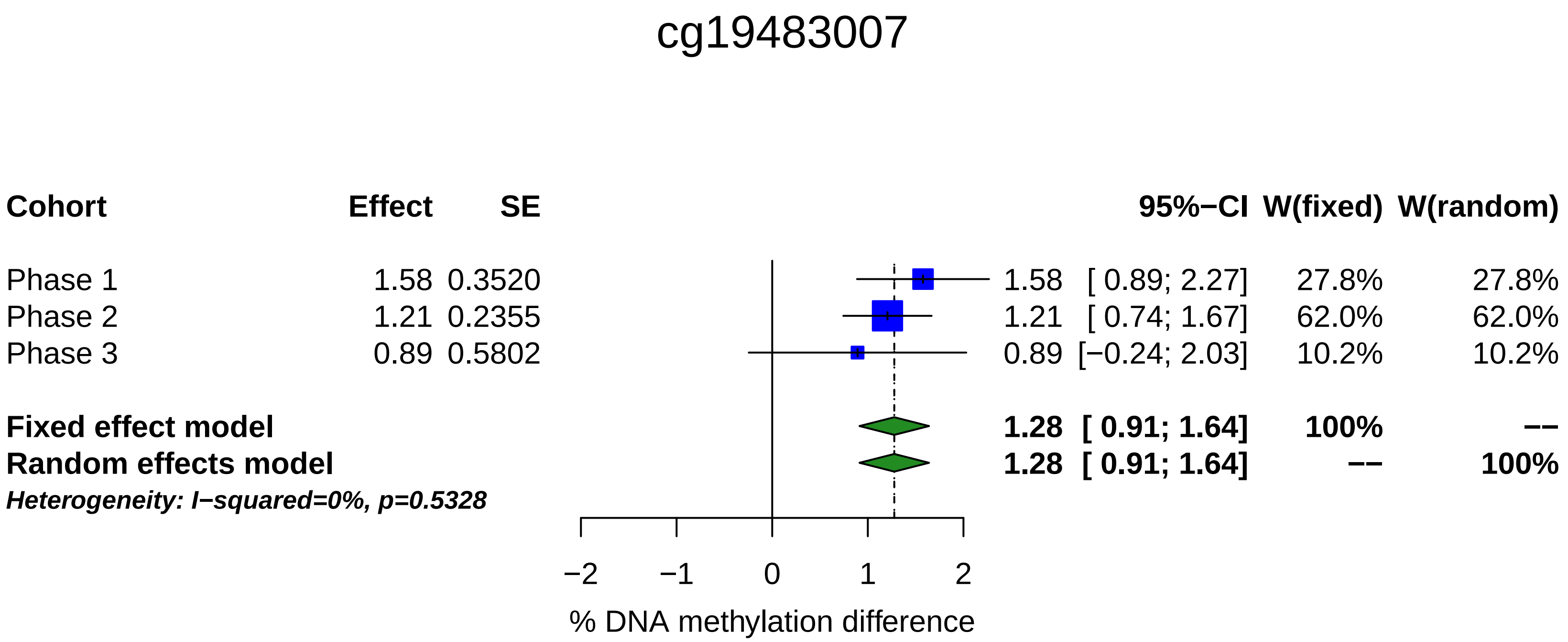
cg19483007
Cohort
Effect
SE
95%−CI
W(fixed)
W(random)
Phase 1 1.58 0.3520 1.58 [0.89; 2.27] 27.8% 27.8%
Phase 2 1.21 0.2355 1.21 [0.74; 1.67] 62.0% 62.0%
Phase 3 0.89 0.5802 0.89 [−0.24; 2.03] 10.2% 10.2%
Fixed effect model 1.28 [0.91; 1.64] 100% −−
Random effects model 1.28 [0.91; 1.64] −− 100%
Heterogeneity: I−squared=0%, p=0.5328
−2
−1
0
1
2
% DNA methylation difference

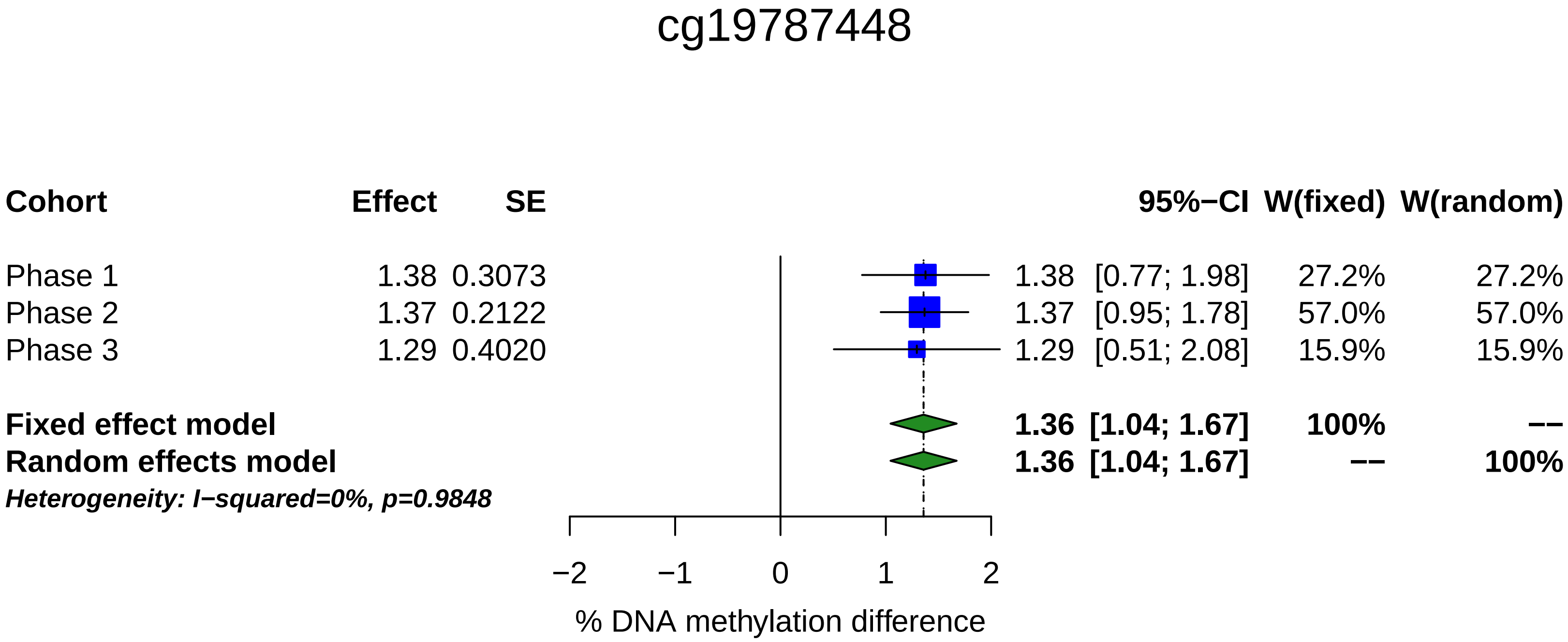
cg19787448
Cohort
Effect
SE
95%−CI
W(fixed)
W(random)
Phase 1 1.38 0.3073 1.38 [0.77; 1.98] 27.2% 27.2%
Phase 2 1.37 0.2122 1.37 [0.95; 1.78] 57.0% 57.0%
Phase 3 1.29 0.4020 1.29 [0.51; 2.08] 15.9% 15.9%
Fixed effect model 1.36 [1.04; 1.67] 100% −−
Random effects model 1.36 [1.04; 1.67] −− 100%
Heterogeneity: I−squared=0%, p=0.9848
−2
−1
0
1
2
% DNA methylation difference

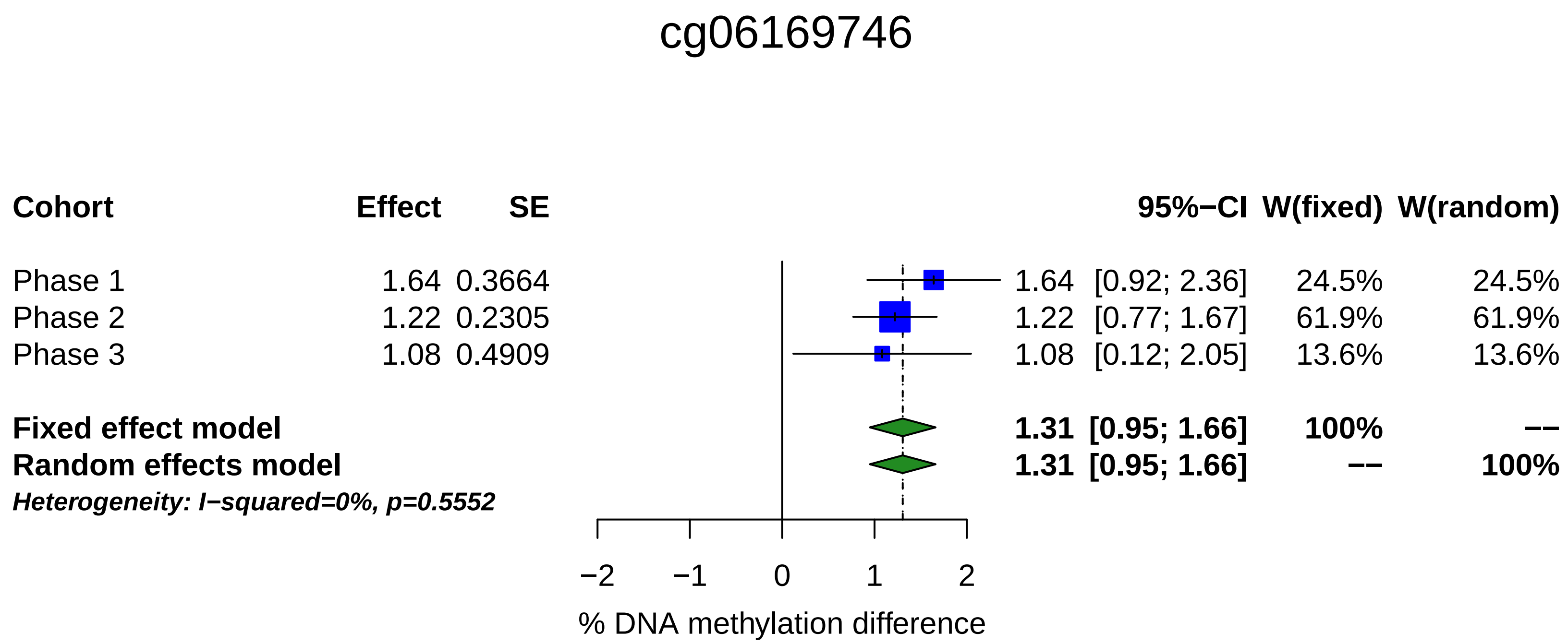
cg06169746
Cohort
Effect
SE
95%−CI
W(fixed)
W(random)
Phase 1 1.64 0.3664 1.64 [0.92; 2.36] 24.5% 24.5%
Phase 2 1.22 0.2305 1.22 [0.77; 1.67] 61.9% 61.9%
Phase 3 1.08 0.4909 1.08 [0.12; 2.05] 13.6% 13.6%
Fixed effect model 1.31 [0.95; 1.66] 100% −−
Random effects model 1.31 [0.95; 1.66] −− 100%
Heterogeneity: I−squared=0%, p=0.5552
−2
−1
0
1
2
% DNA methylation difference

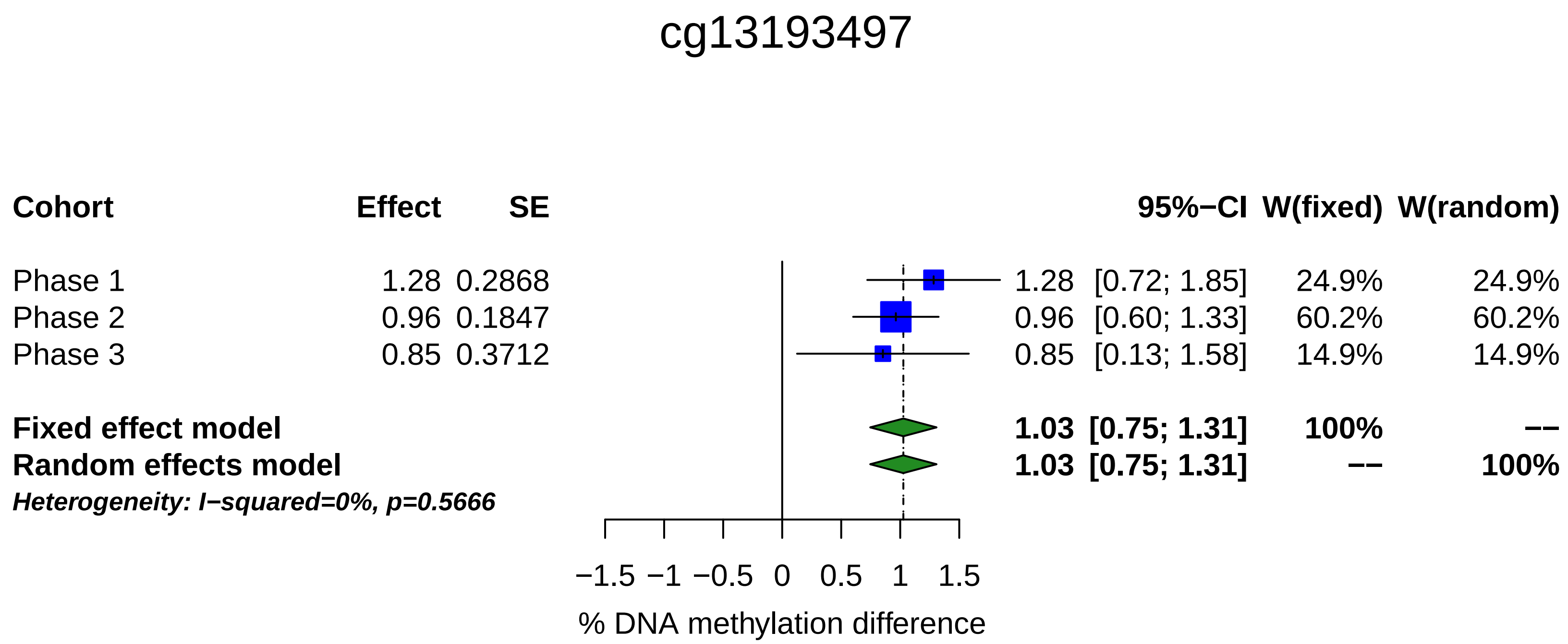
cg13193497
Cohort
Effect
SE
95%−CI
W(fixed)
W(random)
Phase 1 1.28 0.2868 1.28 [0.72; 1.85] 24.9% 24.9%
Phase 2 0.96 0.1847 0.96 [0.60; 1.33] 60.2% 60.2%
Phase 3 0.85 0.3712 0.85 [0.13; 1.58] 14.9% 14.9%
Fixed effect model 1.03 [0.75; 1.31] 100% −−
Random effects model 1.03 [0.75; 1.31] −− 100%
Heterogeneity: I−squared=0%, p=0.5666
−1.5 −1 −0.5 0 0.5 1 1.5
% DNA methylation difference

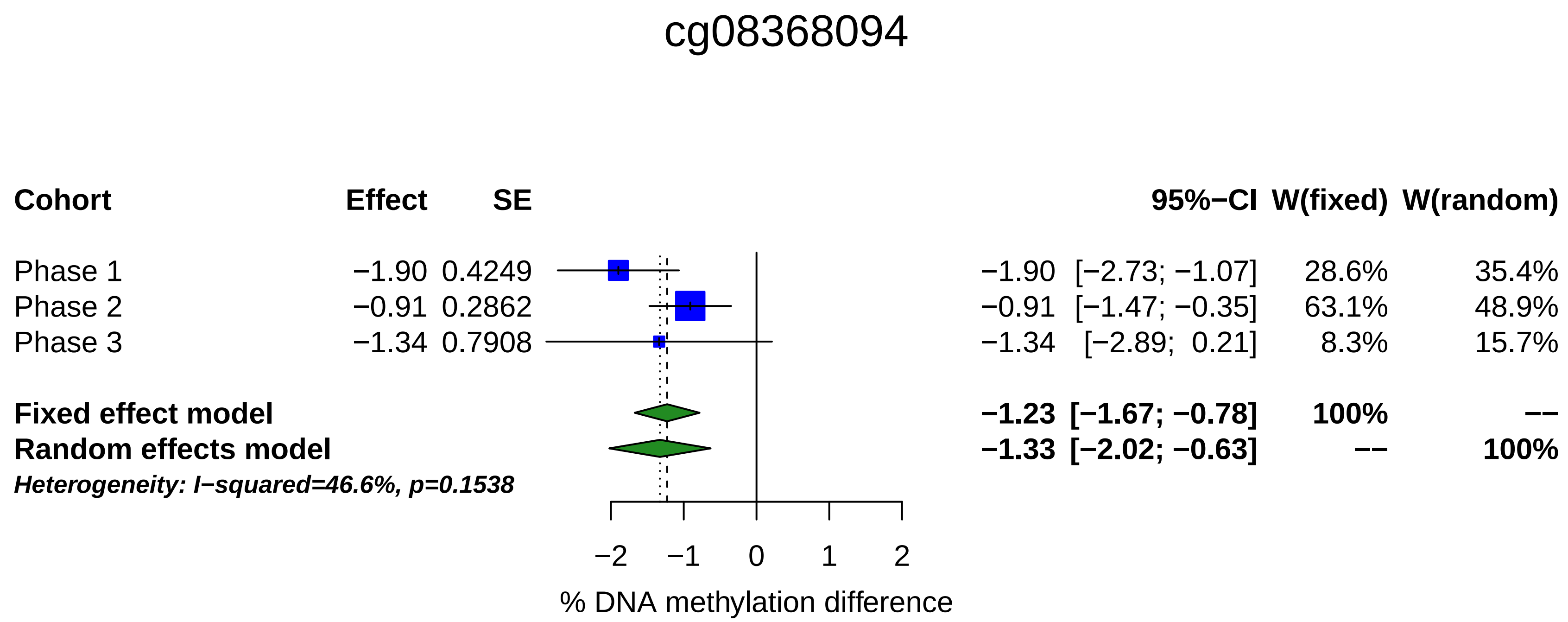
cg08368094
Cohort
Effect
SE
95%−CI
W(fixed)
W(random)
Phase 1 −1.90 0.4249 −1.90 [−2.73; −1.07] 28.6% 35.4%
Phase 2 −0.91 0.2862 −0.91 [−1.47; −0.35] 63.1% 48.9%
Phase 3 −1.34 0.7908 −1.34 [−2.89; 0.21] 8.3% 15.7%
Fixed effect model −1.23 [−1.67; −0.78] 100% −−
Random effects model −1.33 [−2.02; −0.63] −− 100%
Heterogeneity: I−squared=46.6%, p=0.1538
−2
−1
0
1
2
% DNA methylation difference

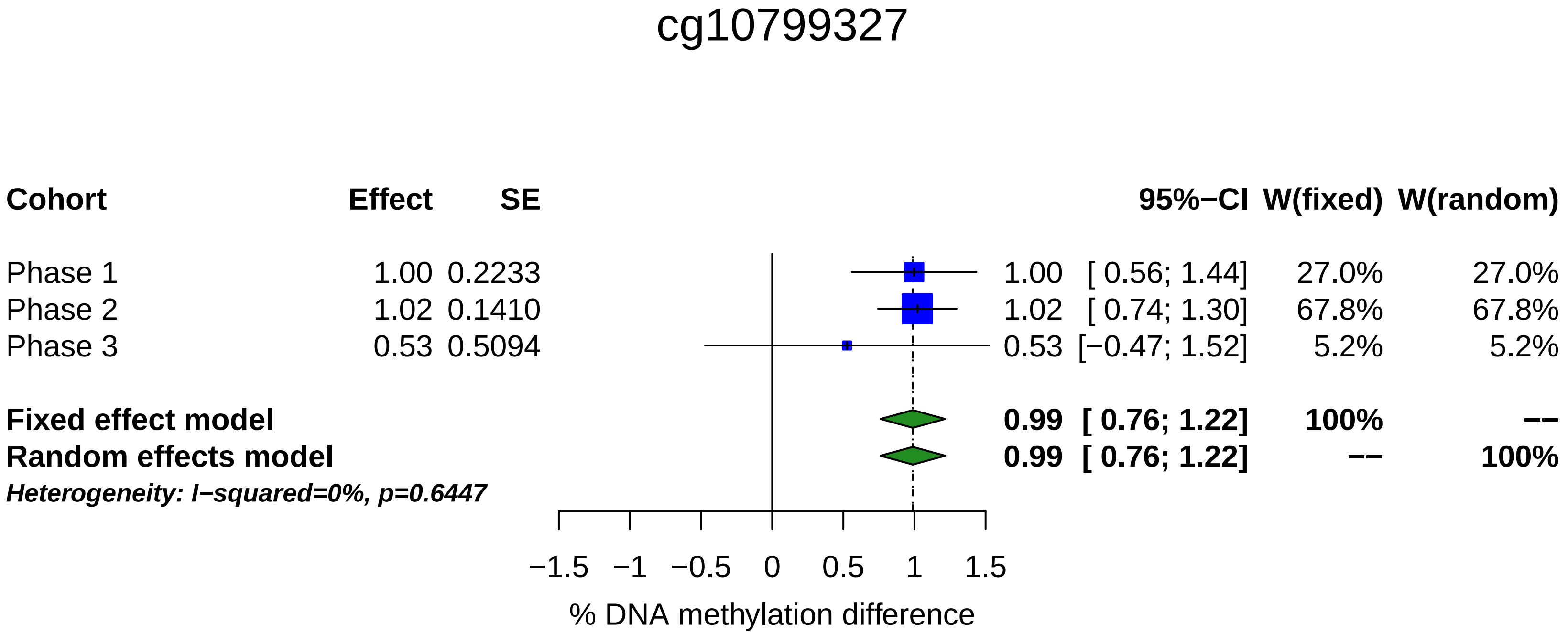
cg10799327
Cohort
Effect
SE
95%−CI
W(fixed)
W(random)
Phase 1 1.00 0.2233 1.00 [0.56; 1.44] 27.0% 27.0%
Phase 2 1.02 0.1410 1.02 [0.74; 1.30] 67.8% 67.8%
Phase 3 0.53 0.5094 0.53 [−0.47; 1.52] 5.2% 5.2%
Fixed effect model 0.99 [0.76; 1.22] 100% −−
Random effects model 0.99 [0.76; 1.22] −− 100%
Heterogeneity: I−squared=0%, p=0.6447
−1.5
−1
−0.5
0
0.5
1
1.5
% DNA methylation difference

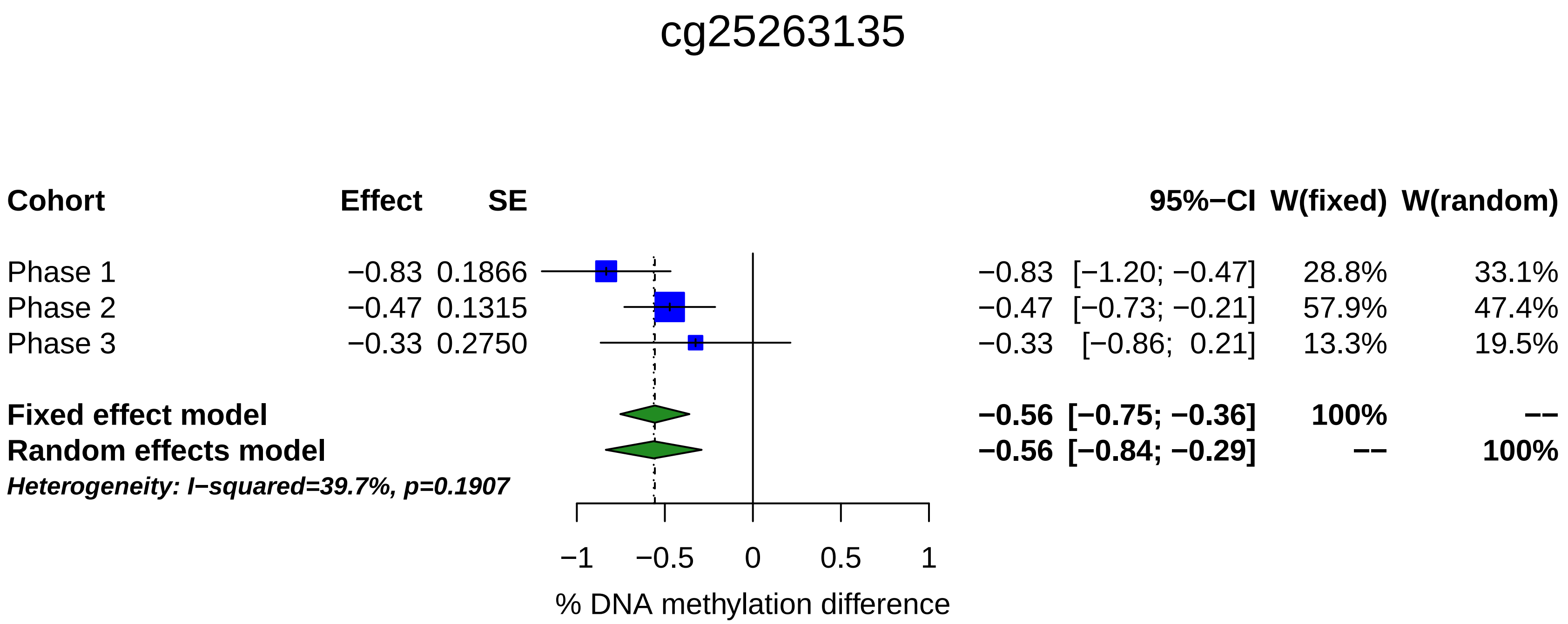
cg25263135
Cohort
Effect
SE
95%−CI
W(fixed)
W(random)
Phase 1 −0.83 0.1866 −0.83 [−1.20; −0.47] 28.8% 33.1%
Phase 2 −0.47 0.1315 −0.47 [−0.73; −0.21] 57.9% 47.4%
Phase 3 −0.33 0.2750 −0.33 [−0.86; 0.21] 13.3% 19.5%
Fixed effect model −0.56 [−0.75; −0.36] 100% −−
Random effects model −0.56 [−0.84; −0.29] −− 100%
Heterogeneity: I−squared=39.7%, p=0.1907
−1
−0.5
0
0.5
1
% DNA methylation difference

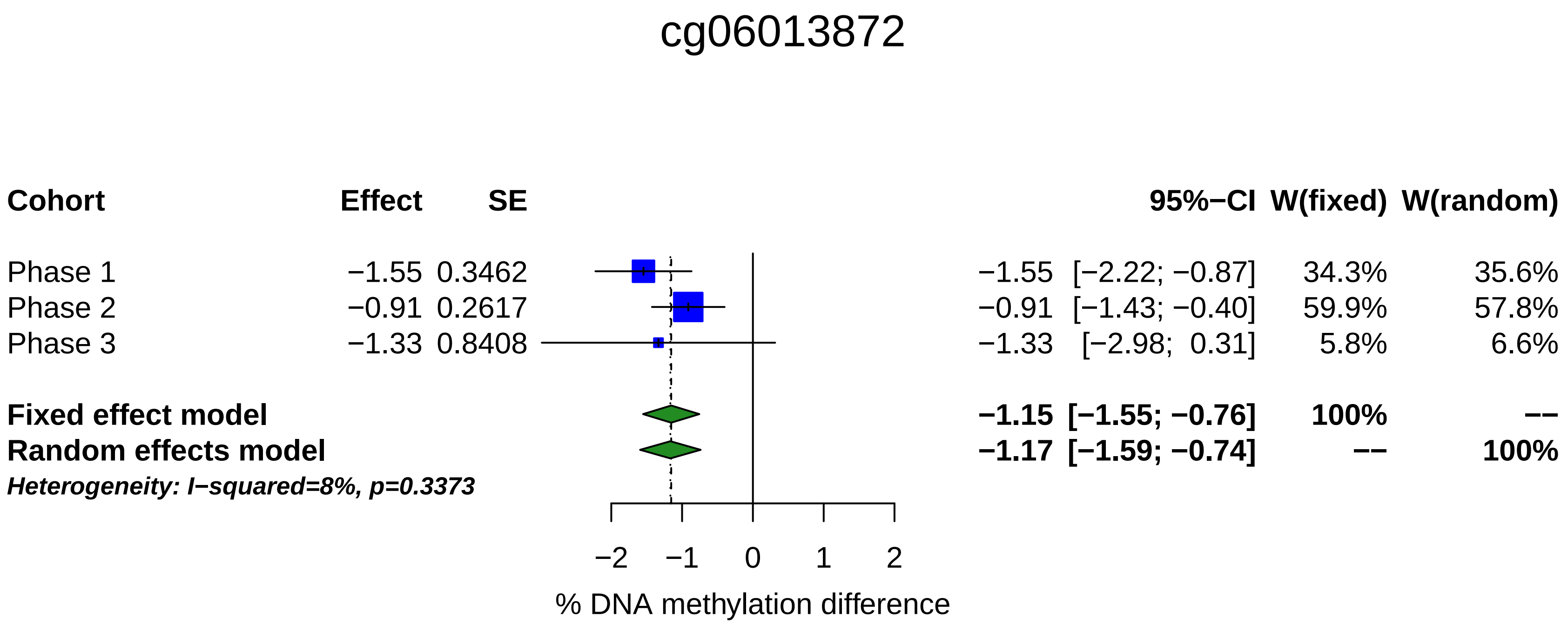
cg06013872
Cohort
Effect
SE
95%−CI
W(fixed)
W(random)
Phase 1 −1.55 0.3462 −1.55 [−2.22; −0.87] 34.3% 35.6%
Phase 2 −0.91 0.2617 −0.91 [−1.43; −0.40] 59.9% 57.8%
Phase 3 −1.33 0.8408 −1.33 [−2.98; 0.31] 5.8% 6.6%
Fixed effect model −1.15 [−1.55; −0.76] 100% −−
Random effects model −1.17 [−1.59; −0.74] −− 100%
Heterogeneity: I−squared=8%, p=0.3373
−2
−1
0
1
2
% DNA methylation difference

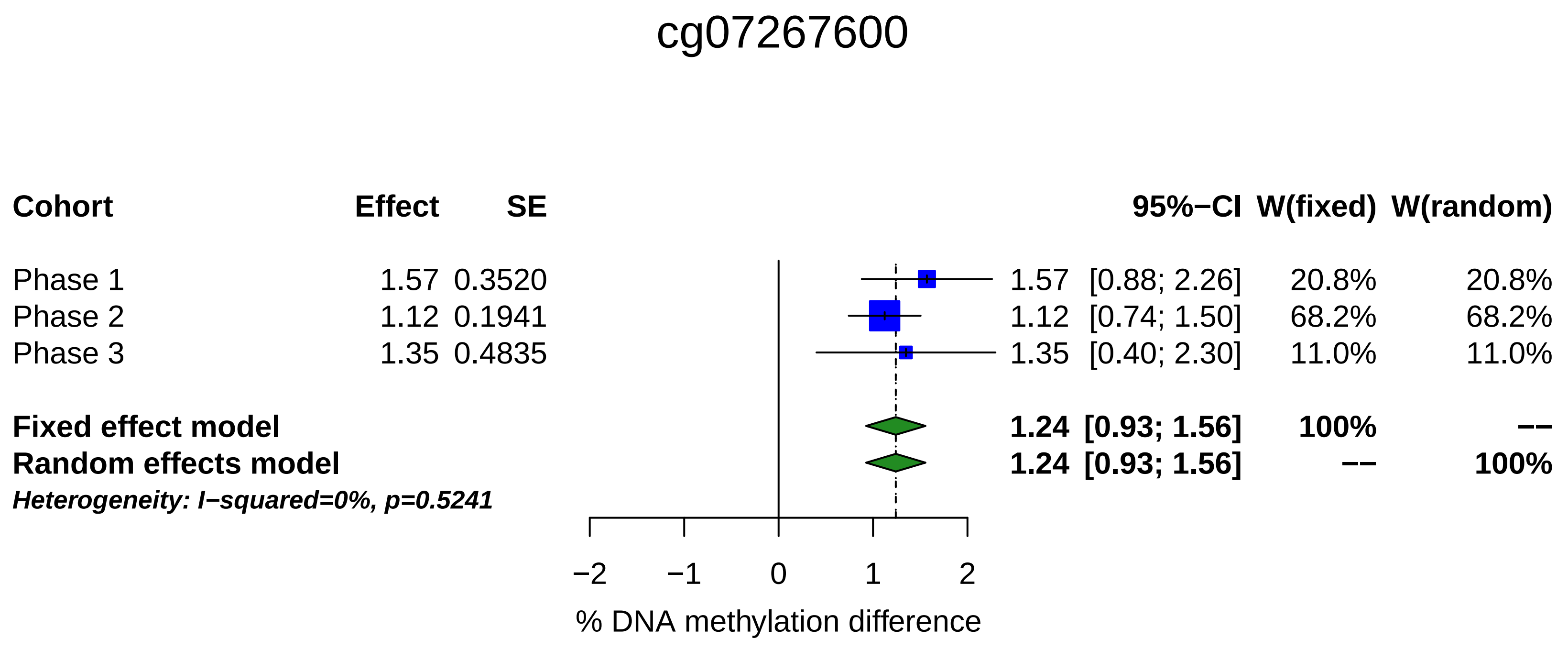
cg07267600
Cohort
Effect
SE
95%−CI
W(fixed)
W(random)
Phase 1 1.57 0.3520 1.57 [0.88; 2.26] 20.8% 20.8%
Phase 2 1.12 0.1941 1.12 [0.74; 1.50] 68.2% 68.2%
Phase 3 1.35 0.4835 1.35 [0.40; 2.30] 11.0% 11.0%
Fixed effect model 1.24 [0.93; 1.56] 100% −−
Random effects model 1.24 [0.93; 1.56] −− 100%
Heterogeneity: I−squared=0%, p=0.5241
−2
−1
0
1
2
% DNA methylation difference

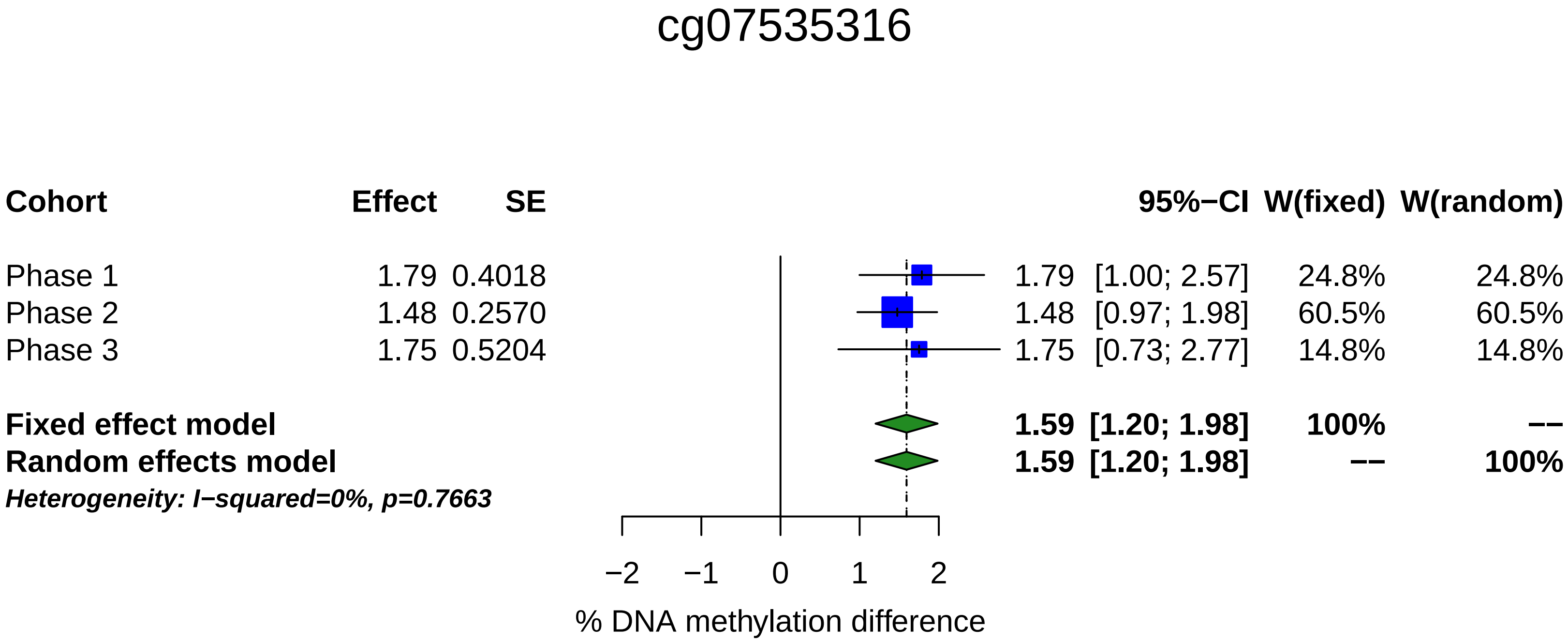
cg07535316
Cohort
Effect
SE
95%−CI
W(fixed)
W(random)
Phase 1 1.79 0.4018 1.79 [1.00; 2.57] 24.8% 24.8%
Phase 2 1.48 0.2570 1.48 [0.97; 1.98] 60.5% 60.5%
Phase 3 1.75 0.5204 1.75 [0.73; 2.77] 14.8% 14.8%
Fixed effect model 1.59 [1.20; 1.98] 100% −−
Random effects model 1.59 [1.20; 1.98] −− 100%
Heterogeneity: I−squared=0%, p=0.7663
−2
−1
0
1
2
% DNA methylation difference

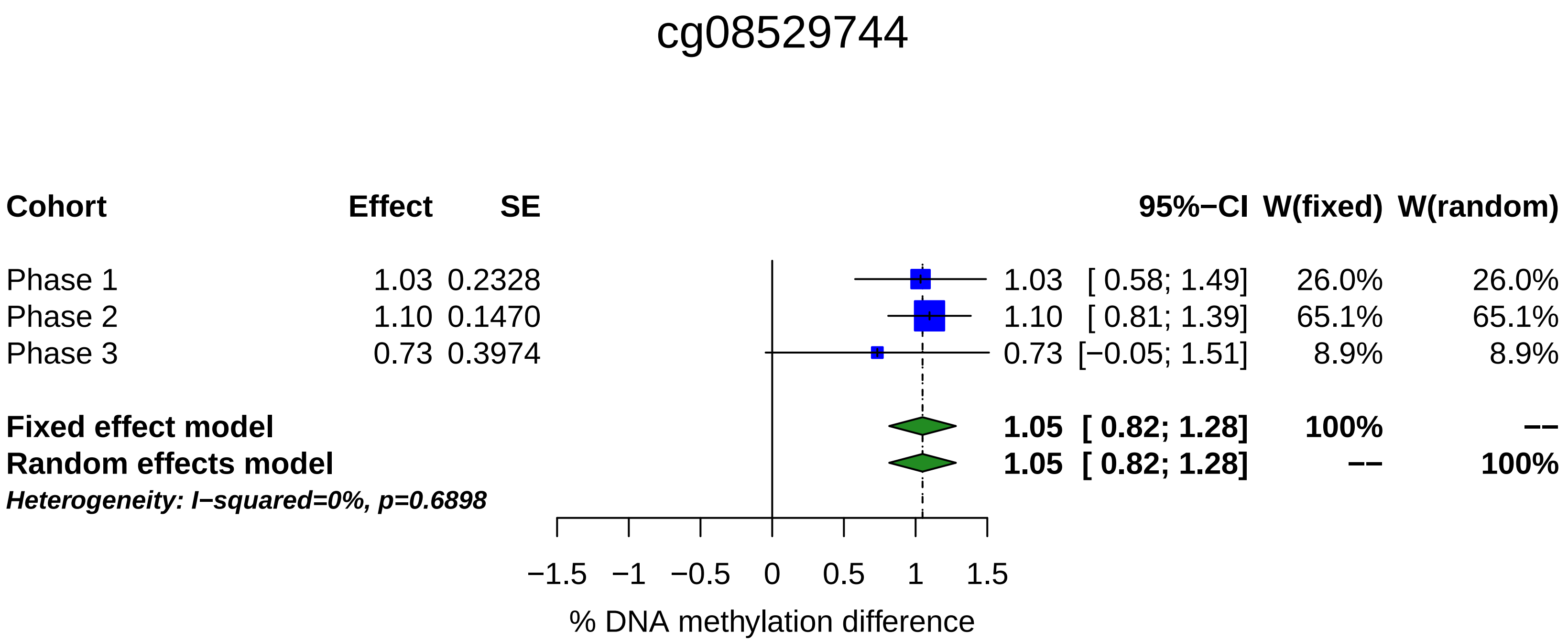
cg08529744
Cohort
Effect
SE
95%−CI
W(fixed)
W(random)
Phase 1 1.03 0.2328 1.03 [0.58; 1.49] 26.0% 26.0%
Phase 2 1.10 0.1470 1.10 [0.81; 1.39] 65.1% 65.1%
Phase 3 0.73 0.3974 0.73 [−0.05; 1.51] 8.9% 8.9%
Fixed effect model 1.05 [0.82; 1.28] 100% −−
Random effects model 1.05 [0.82; 1.28] −− 100%
Heterogeneity: I−squared=0%, p=0.6898
−1.5
−1
−0.5
0
0.5
1
1.5
% DNA methylation difference

cg23598089

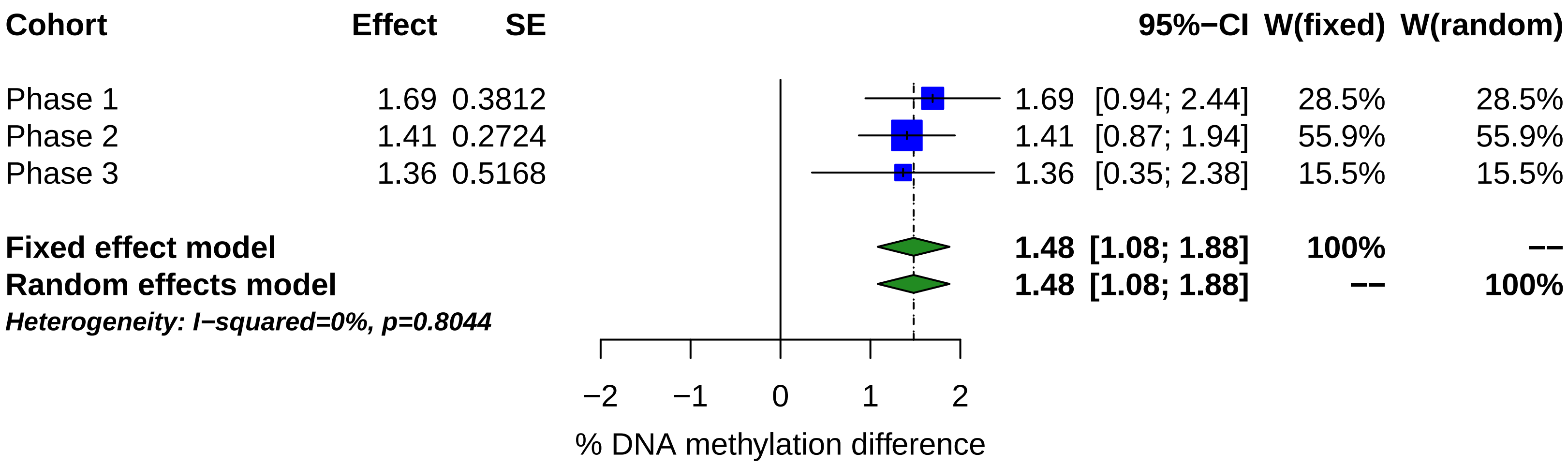
Cohort
Effect
SE
95%−CI
W(fixed)
W(random)
Phase 1
1.69
0.3812
1.69 [0.94; 2.44]
28.5%
28.5%
Phase 2
1.41
0.2724
1.41 [0.87; 1.94]
55.9%
55.9%
Phase 3
1.36
0.5168
1.36 [0.35; 2.38]
15.5%
15.5%
Fixed effect model
1.48 [1.08; 1.88]
100%
−−
Random effects model
1.48 [1.08; 1.88]
−−
100%
Heterogeneity: I−squared=0%, p=0.8044
−2
−1
0
1
2
% DNA methylation difference

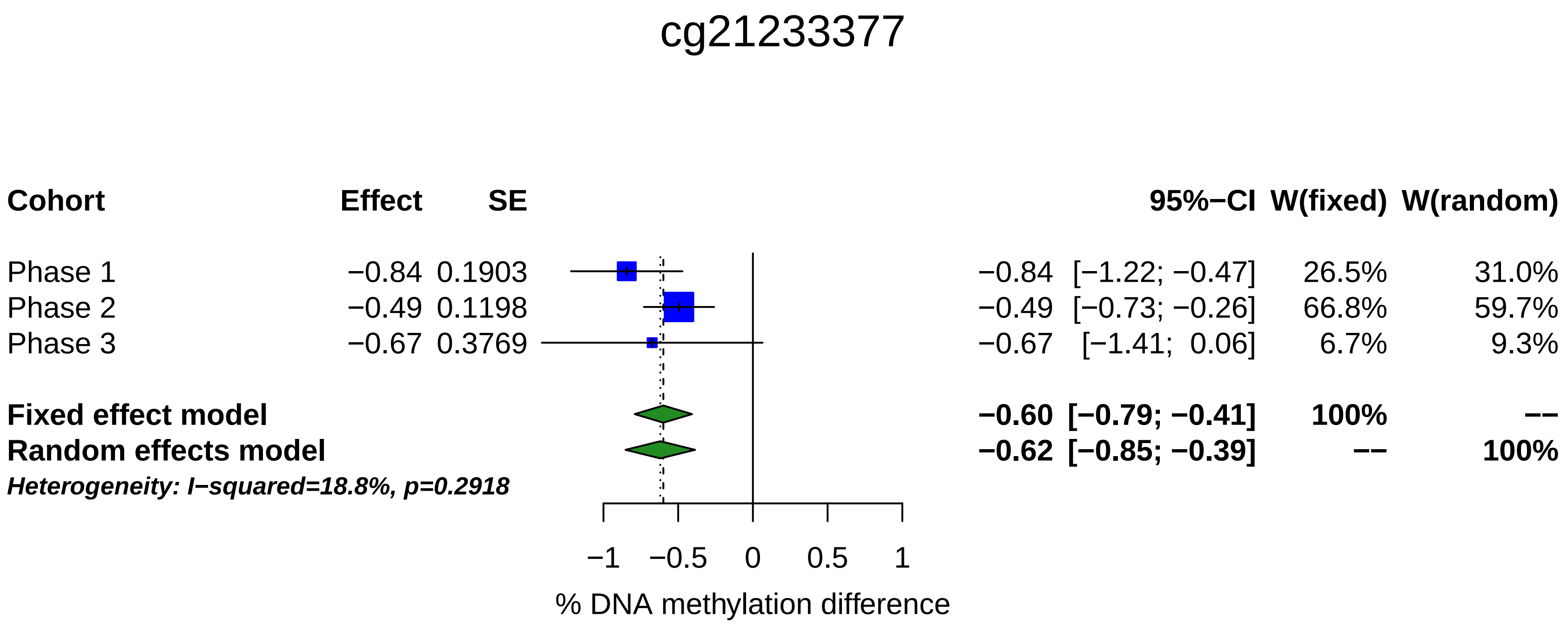
cg21233377
Cohort
Effect
SE
95%−CI
W(fixed)
W(random)
Phase 1 −0.84 0.1903 −0.84 [−1.22; −0.47] 26.5% 31.0%
Phase 2 −0.49 0.1198 −0.49 [−0.73; −0.26] 66.8% 59.7%
Phase 3 −0.67 0.3769 −0.67 [−1.41; 0.06] 6.7% 9.3%
Fixed effect model −0.60 [−0.79; −0.41] 100% −−
Random effects model −0.62 [−0.85; −0.39] −− 100%
Heterogeneity: I−squared=18.8%, p=0.2918
−1
−0.5
0
0.5
1
% DNA methylation difference

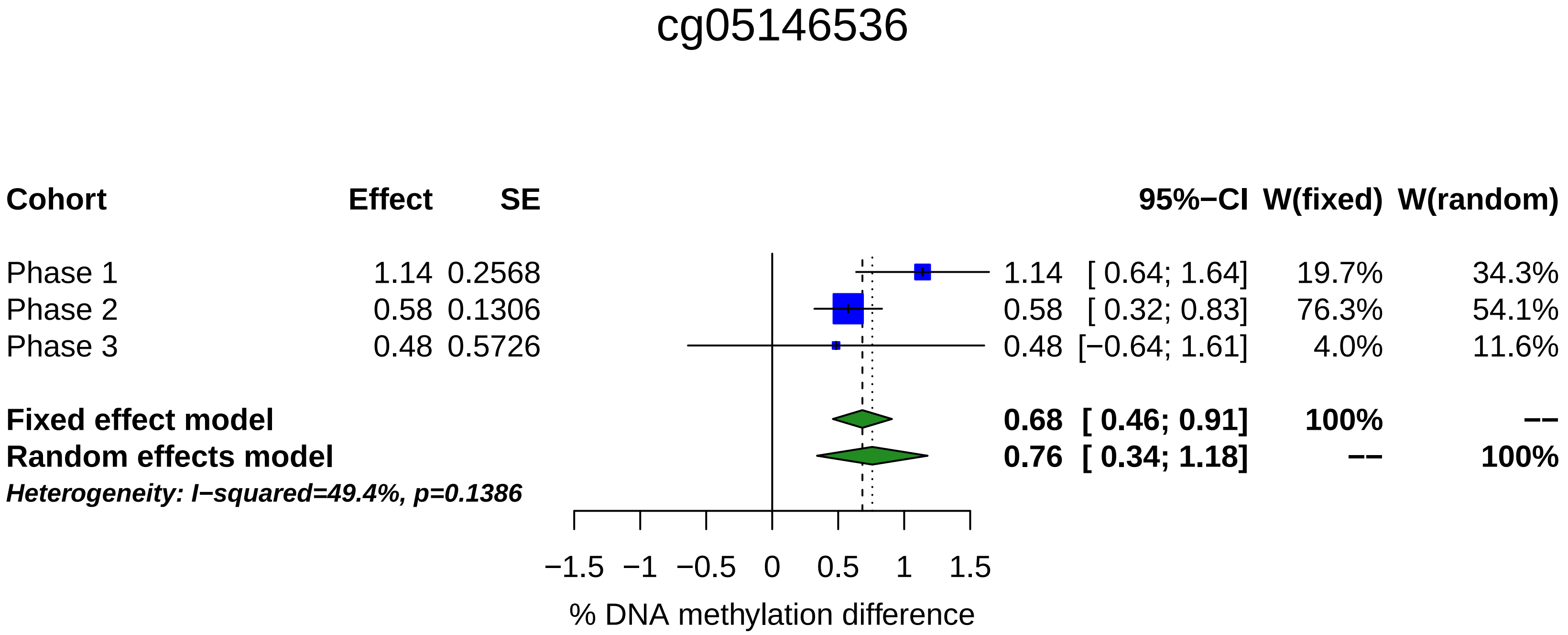
cg05146536
Cohort
Effect
SE
95%−CI
W(fixed)
W(random)
Phase 1 1.14 0.2568 1.14 [0.64; 1.64] 19.7% 34.3%
Phase 2 0.58 0.1306 0.58 [0.32; 0.83] 76.3% 54.1%
Phase 3 0.48 0.5726 0.48 [−0.64; 1.61] 4.0% 11.6%
Fixed effect model 0.68 [0.46; 0.91] 100% −−
Random effects model 0.76 [0.34; 1.18] −− 100%
Heterogeneity: I−squared=49.4%, p=0.1386
−1.5 −1 −0.5 0 0.5 1 1.5
% DNA methylation difference

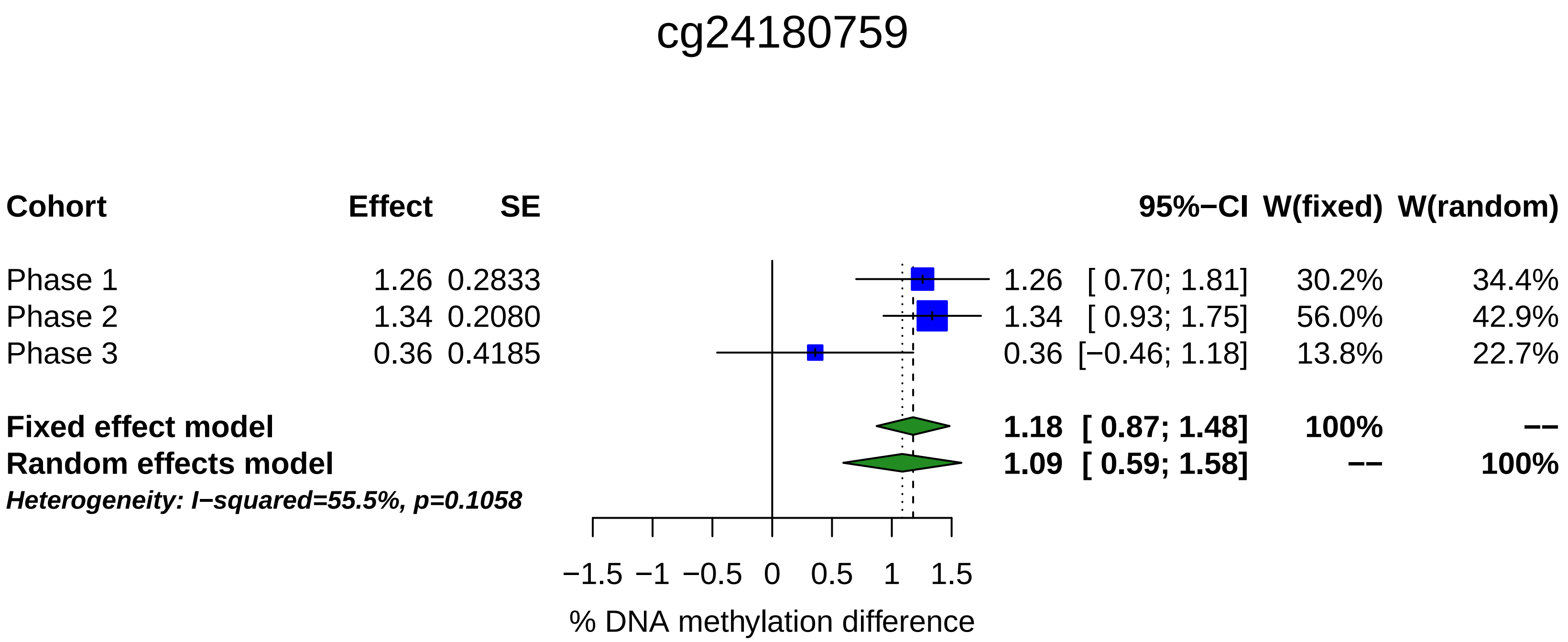
cg24180759
Cohort
Effect
SE
95%−CI
W(fixed)
W(random)
Phase 1
1.26
0.2833
1.26 [0.70; 1.81]
30.2%
34.4%
Phase 2
1.34
0.2080
1.34 [0.93; 1.75]
56.0%
42.9%
Phase 3
0.36
0.4185
0.36 [−0.46; 1.18]
13.8%
22.7%
Fixed effect model
1.18 [0.87; 1.48]
100%
−−
Random effects model
1.09 [0.59; 1.58]
−−
100%
Heterogeneity: I−squared=55.5%, p=0.1058
−1.5 −1 −0.5 0 0.5 1 1.5
% DNA methylation difference

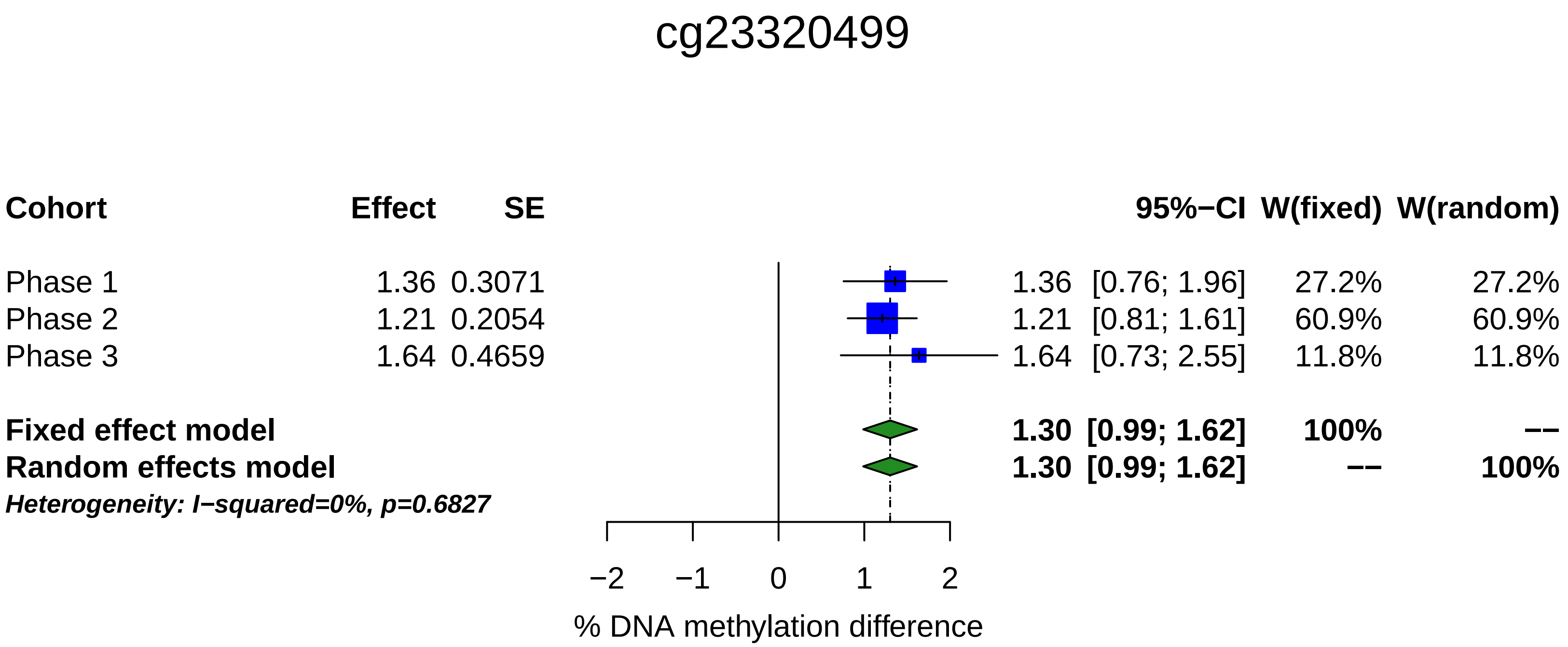
cg23320499
Cohort
Effect
SE
95%−CI
W(fixed)
W(random)
Phase 1 1.36 0.3071 1.36 [0.76; 1.96] 27.2% 27.2%
Phase 2 1.21 0.2054 1.21 [0.81; 1.61] 60.9% 60.9%
Phase 3 1.64 0.4659 1.64 [0.73; 2.55] 11.8% 11.8%
Fixed effect model 1.30 [0.99; 1.62] 100% −−
Random effects model 1.30 [0.99; 1.62] −− 100%
Heterogeneity: I−squared=0%, p=0.6827
−2
−1
0
1
2
% DNA methylation difference

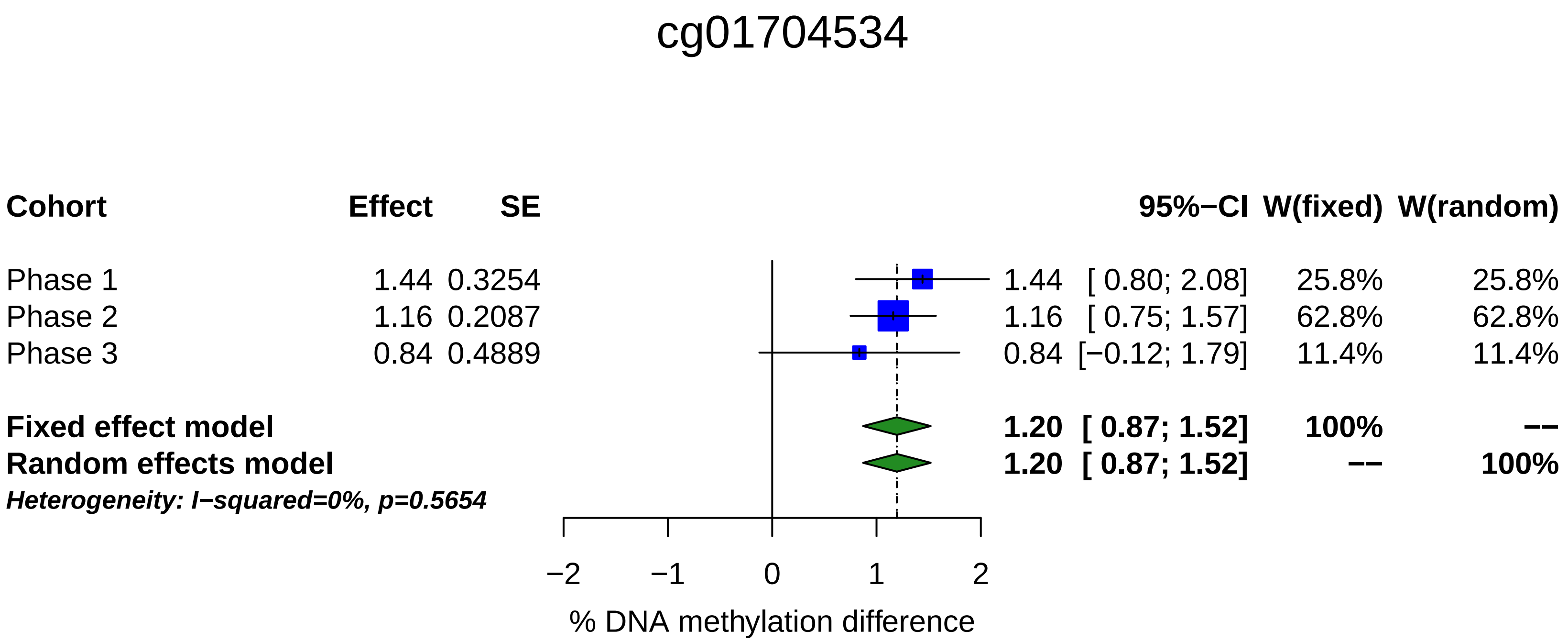
cg01704534
Cohort
Effect
SE
95%−CI
W(fixed)
W(random)
Phase 1
1.44
0.3254
1.44
[0.80; 2.08]
25.8%
25.8%
Phase 2
1.16
0.2087
1.16
[0.75; 1.57]
62.8%
62.8%
Phase 3
0.84
0.4889
0.84
[−0.12; 1.79]
11.4%
11.4%
Fixed effect model
1.20
[0.87; 1.52]
100%
−−
Random effects model
1.20
[0.87; 1.52]
−−
100%
Heterogeneity: I−squared=0%, p=0.5654
−2
−1
0
1
2
% DNA methylation difference

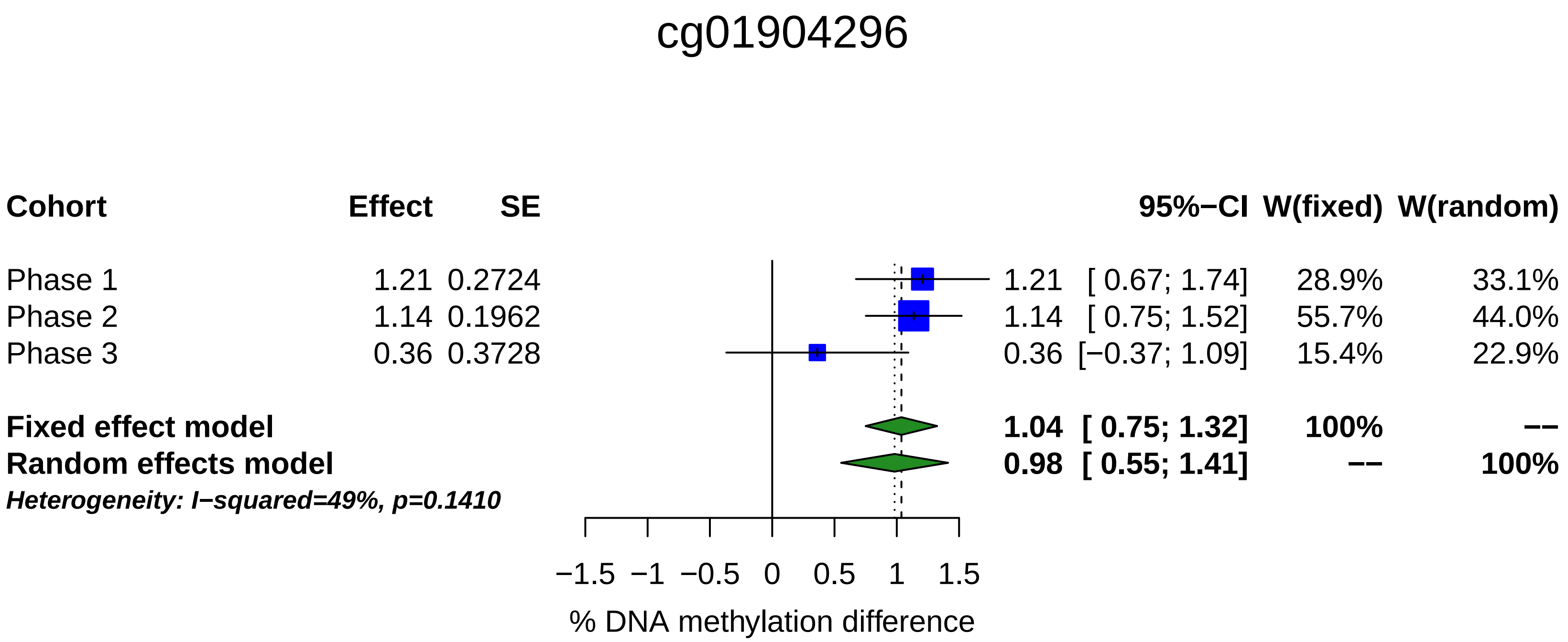
cg01904296
Cohort
Effect
SE
95%−CI
W(fixed)
W(random)
Phase 1 1.21 0.2724 1.21 [0.67; 1.74] 28.9% 33.1%
Phase 2 1.14 0.1962 1.14 [0.75; 1.52] 55.7% 44.0%
Phase 3 0.36 0.3728 0.36 [−0.37; 1.09] 15.4% 22.9%
Fixed effect model 1.04 [0.75; 1.32] 100% −−
Random effects model 0.98 [0.55; 1.41] −− 100%
Heterogeneity: I−squared=49%, p=0.1410
−1.5 −1 −0.5 0 0.5 1 1.5
% DNA methylation difference

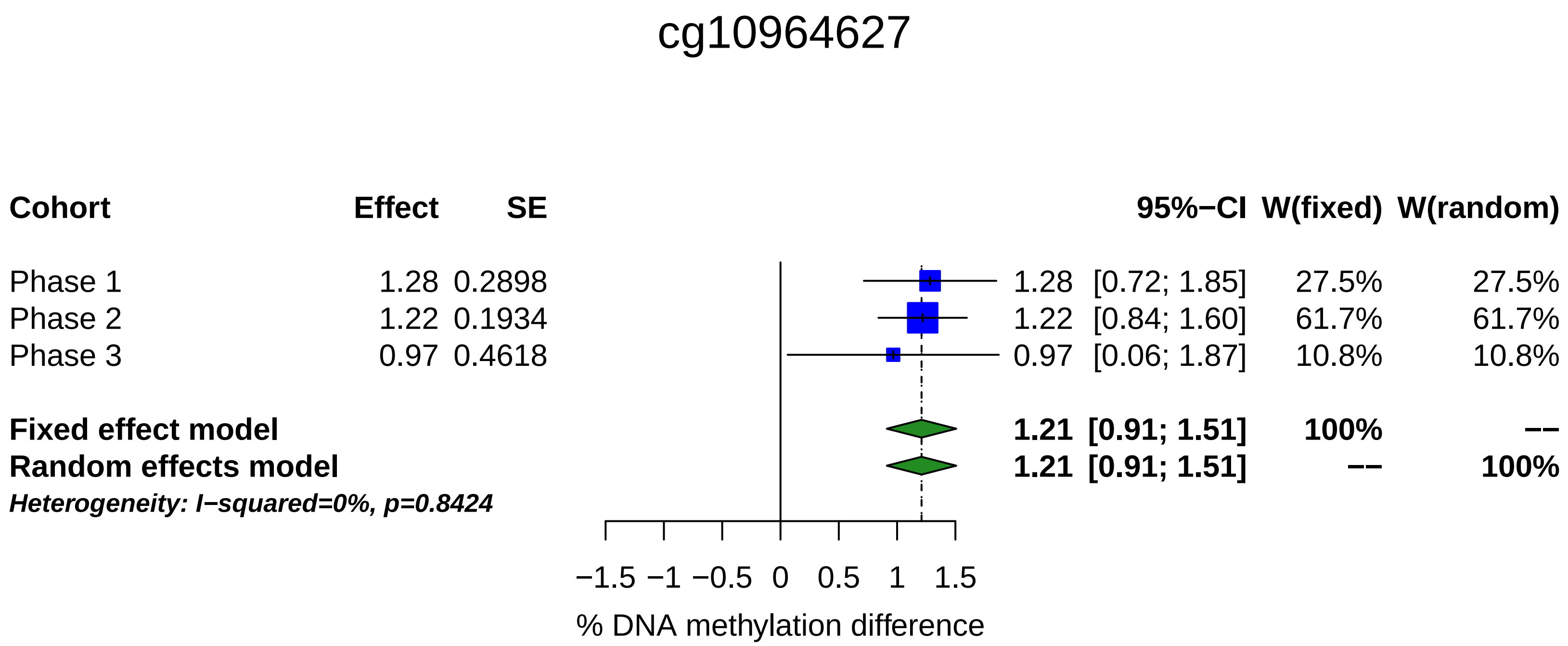
cg10964627
Cohort
Effect
SE
95%−CI
W(fixed)
W(random)
Phase 1 1.28 0.2898 1.28 [0.72; 1.85] 27.5% 27.5%
Phase 2 1.22 0.1934 1.22 [0.84; 1.60] 61.7% 61.7%
Phase 3 0.97 0.4618 0.97 [0.06; 1.87] 10.8% 10.8%
Fixed effect model 1.21 [0.91; 1.51] 100% −−
Random effects model 1.21 [0.91; 1.51] −− 100%
Heterogeneity: I−squared=0%, p=0.8424
−1.5 −1 −0.5 0 0.5 1 1.5
% DNA methylation difference

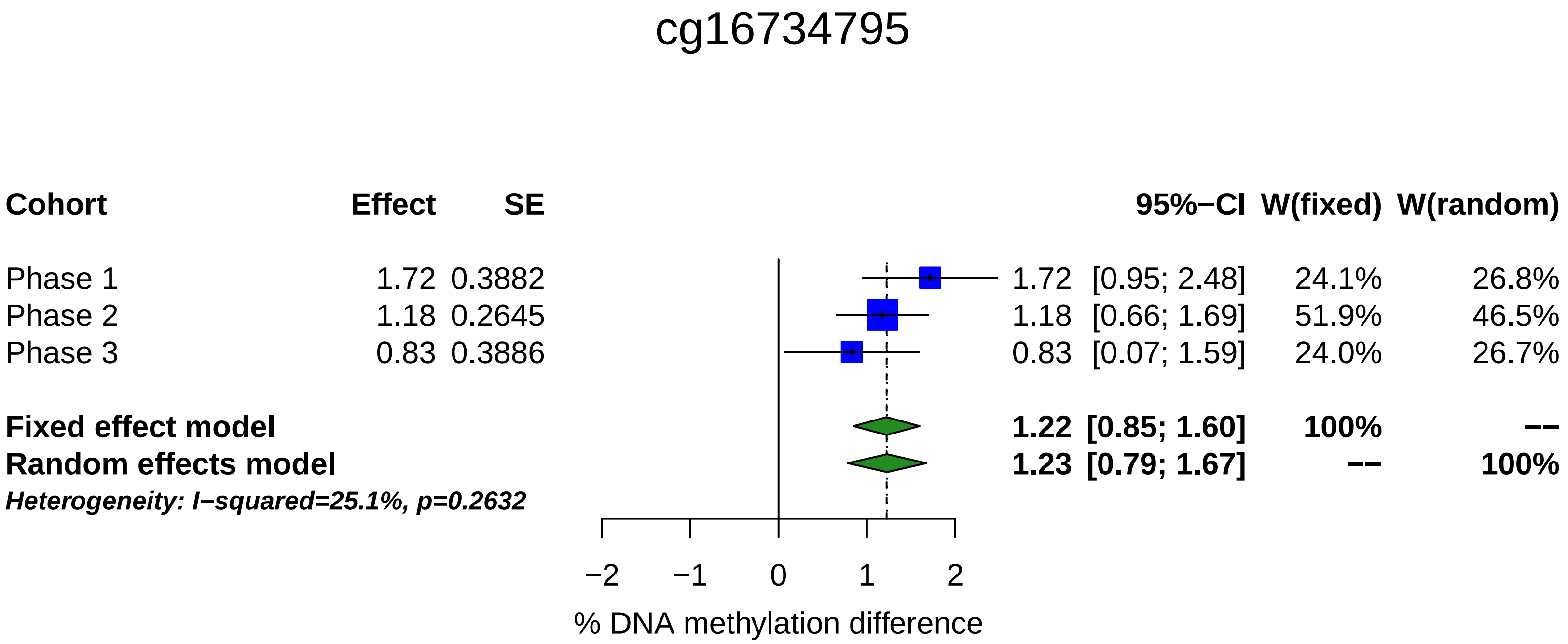
cg16734795
Cohort
Effect
SE
95%−CI
W(fixed)
W(random)
Phase 1
1.72
0.3882
1.72 [0.95; 2.48]
24.1%
26.8%
Phase 2
1.18
0.2645
1.18 [0.66; 1.69]
51.9%
46.5%
Phase 3
0.83
0.3886
0.83 [0.07; 1.59]
24.0%
26.7%
Fixed effect model
1.22 [0.85; 1.60]
100%
−−
Random effects model
1.23 [0.79; 1.67]
−−
100%
Heterogeneity: I−squared=25.1%, p=0.2632
−2
−1
0
1
2
% DNA methylation difference

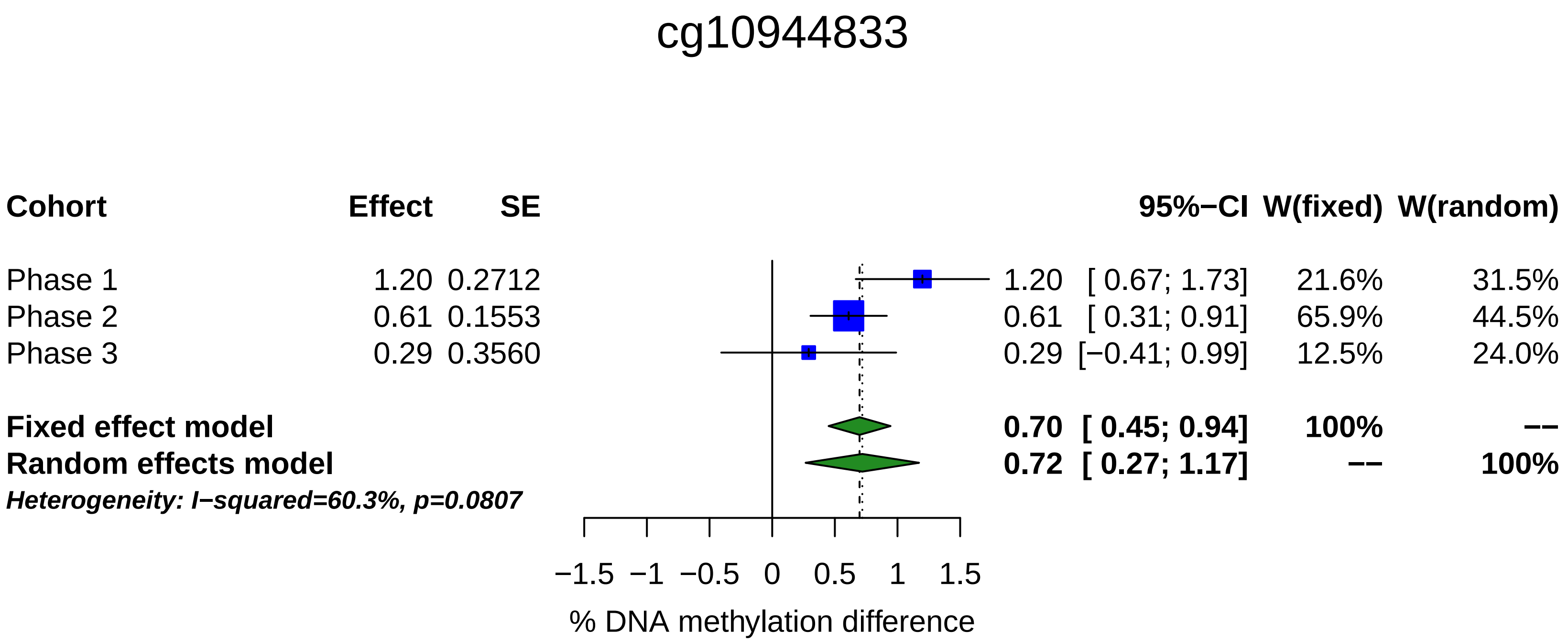
cg10944833
Cohort
Effect
SE
95%−CI
W(fixed)
W(random)
Phase 1 1.20 0.2712 1.20 [0.67; 1.73] 21.6% 31.5%
Phase 2 0.61 0.1553 0.61 [0.31; 0.91] 65.9% 44.5%
Phase 3 0.29 0.3560 0.29 [−0.41; 0.99] 12.5% 24.0%
Fixed effect model 0.70 [0.45; 0.94] 100% −−
Random effects model 0.72 [0.27; 1.17] −− 100%
Heterogeneity: I−squared=60.3%, p=0.0807
−1.5 −1 −0.5 0 0.5 1 1.5
% DNA methylation difference

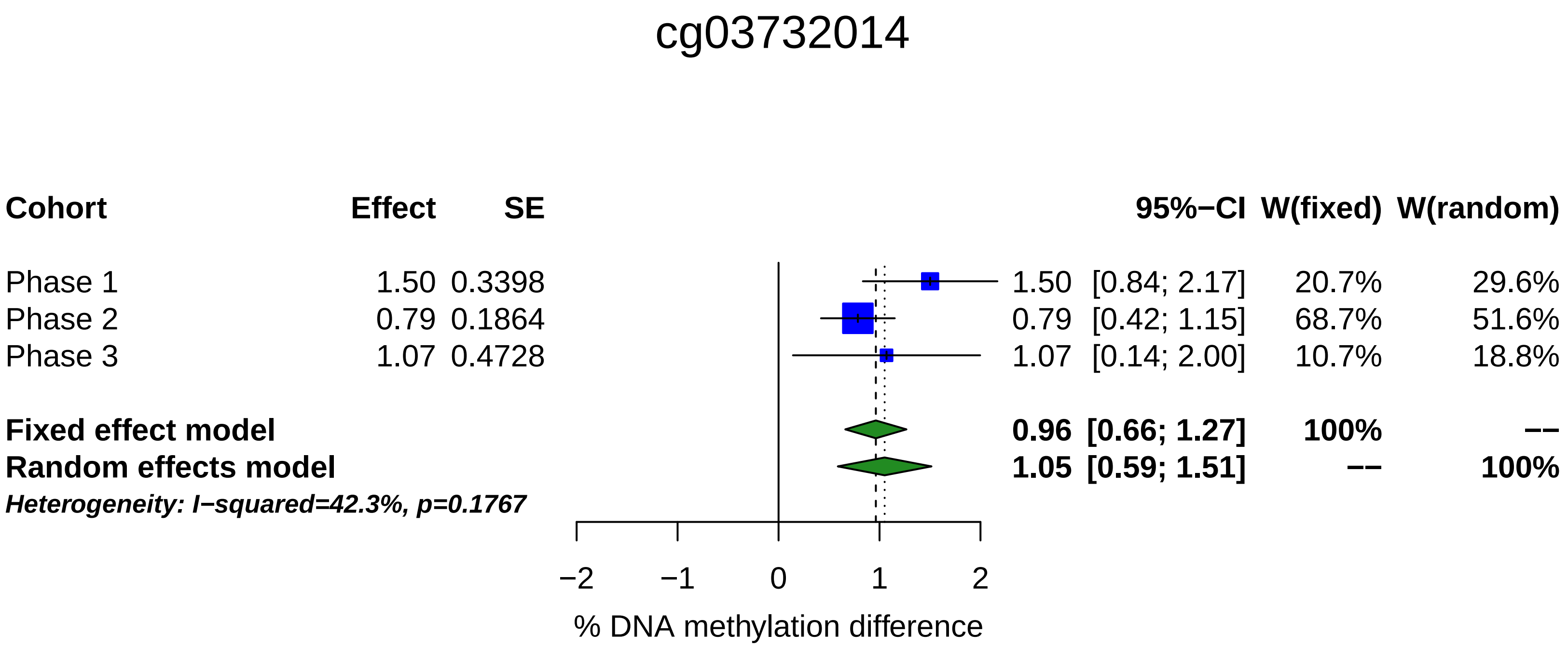

cg03732014
Cohort
Effect
SE
95%−CI
W(fixed)
W(random)
Phase 1 1.50 0.3398 1.50 [0.84; 2.17] 20.7% 29.6%
Phase 2 0.79 0.1864 0.79 [0.42; 1.15] 68.7% 51.6%
Phase 3 1.07 0.4728 1.07 [0.14; 2.00] 10.7% 18.8%
Fixed effect model 0.96 [0.66; 1.27] 100% −−
Random effects model 1.05 [0.59; 1.51] −− 100%
Heterogeneity: I−squared=42.3%, p=0.1767
−2
−1
0
1
2
% DNA methylation difference

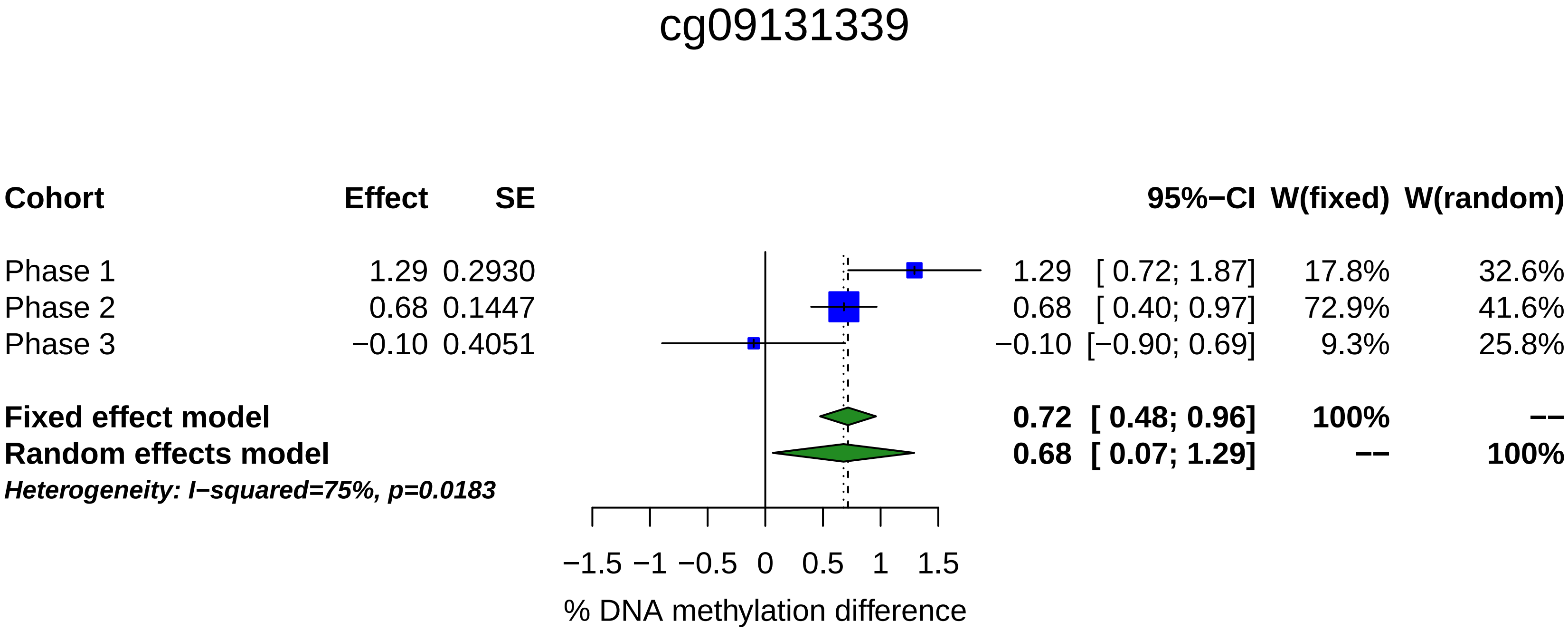
cg09131339
Cohort
Effect
SE
95%−CI
W(fixed)
W(random)
Phase 1 1.29 0.2930 1.29 [0.72; 1.87] 17.8% 32.6%
Phase 2 0.68 0.1447 0.68 [0.40; 0.97] 72.9% 41.6%
Phase 3 −0.10 0.4051 −0.10 [−0.90; 0.69] 9.3% 25.8%
Fixed effect model 0.72 [0.48; 0.96] 100% −−
Random effects model 0.68 [0.07; 1.29] −− 100%
Heterogeneity: I−squared=75%, p=0.0183
−1.5
−1
−0.5
0
0.5
1
1.5
% DNA methylation difference

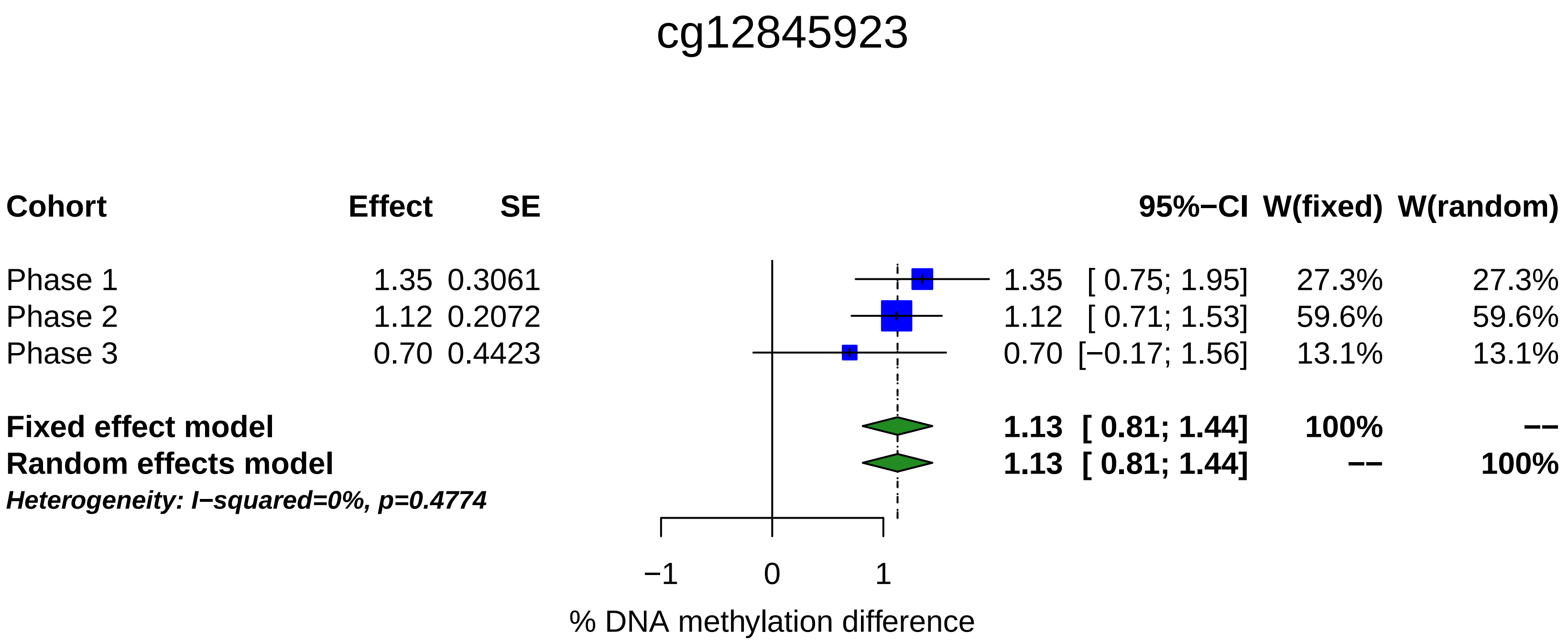
cg12845923
Cohort
Effect
SE
95%−CI
W(fixed)
W(random)
Phase 1
1.35
0.3061
1.35
[0.75; 1.95]
27.3%
27.3%
Phase 2
1.12
0.2072
1.12
[0.71; 1.53]
59.6%
59.6%
Phase 3
0.70
0.4423
0.70
[−0.17; 1.56]
13.1%
13.1%
Fixed effect model
1.13
[0.81; 1.44]
100%
−−
Random effects model
1.13
[0.81; 1.44]
−−
100%
Heterogeneity: I−squared=0%, p=0.4774
−1
0
1
% DNA methylation difference

cg04813697

Cohort	Effect	SE	95%−CI	W(fixed)	W(random)
Phase 1	2.20	0.4985	2.20 [1.22; 3.18]	26.3%	32.2%
Phase 2	1.80	0.3327	1.80 [1.15; 2.45]	59.0%	44.8%
Phase 3	0.55	0.6641	0.55 [−0.75; 1.85]	14.8%	23.1%
**Fixed effect model**			**1.72 [1.22; 2.22]**	**100%**	**−−**
**Random effects model**			**1.64 [0.86; 2.42]**	**−−**	**100%**

Heterogeneity: I−squared=50.9%, p=0.1303

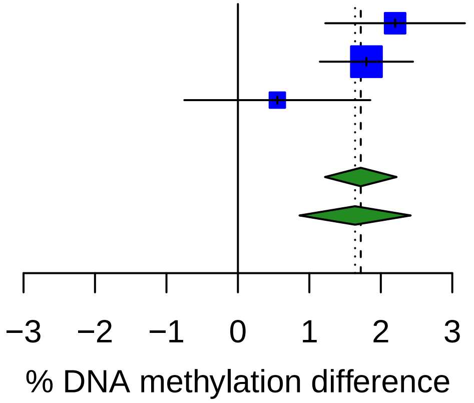

cg07715909

Cohort	Effect	SE	95%−CI	W(fixed)	W(random)
Phase 1	0.99	0.2249	0.99 [0.55; 1.43]	22.4%	30.4%
Phase 2	0.50	0.1334	0.50 [0.24; 0.77]	63.6%	46.5%
Phase 3	0.86	0.2838	0.86 [0.31; 1.42]	14.0%	23.1%
**Fixed effect model**			**0.66 [0.46; 0.87]**	**100%**	**−−**
**Random effects model**			**0.74 [0.40; 1.07]**	**−−**	**100%**

Heterogeneity: I−squared=50.5%, p=0.1327

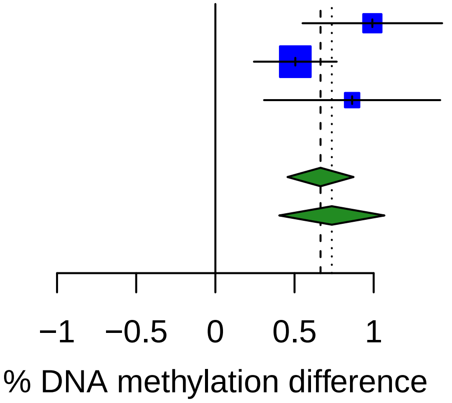

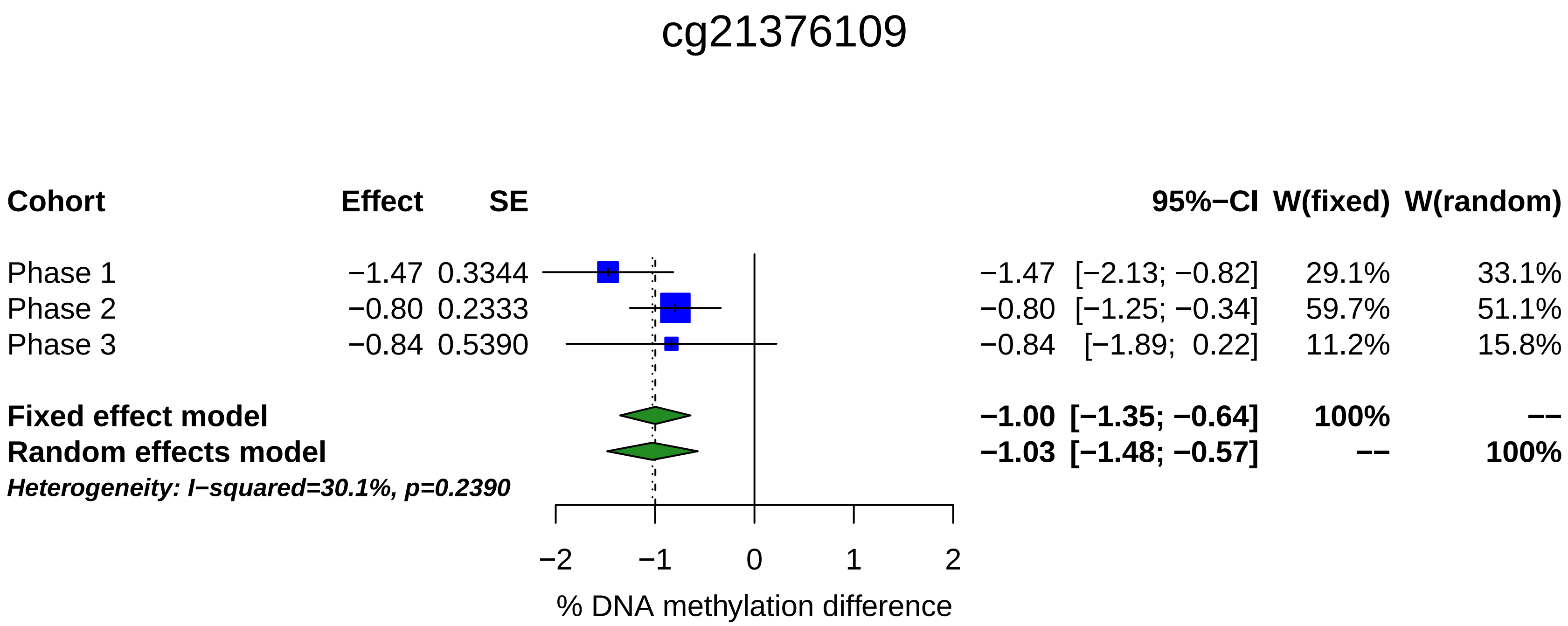
cg21376109
Cohort
Effect
SE
95%−CI
W(fixed)
W(random)
Phase 1 −1.47 0.3344 −1.47 [−2.13; −0.82] 29.1% 33.1%
Phase 2 −0.80 0.2333 −0.80 [−1.25; −0.34] 59.7% 51.1%
Phase 3 −0.84 0.5390 −0.84 [−1.89; 0.22] 11.2% 15.8%
Fixed effect model −1.00 [−1.35; −0.64] 100% −−
Random effects model −1.03 [−1.48; −0.57] −− 100%
Heterogeneity: I−squared=30.1%, p=0.2390
−2
−1
0
1
2
% DNA methylation difference

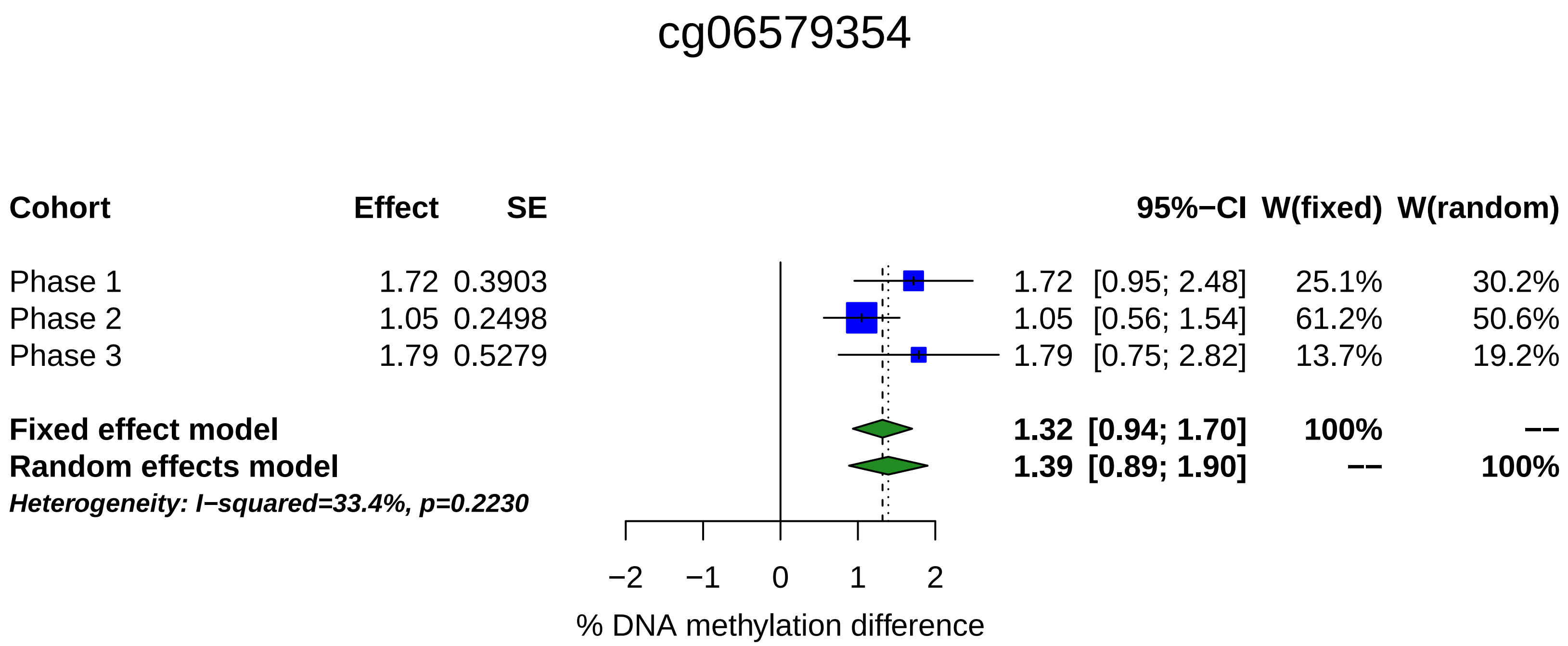
cg06579354
Cohort
Effect
SE
95%−CI
W(fixed)
W(random)
Phase 1 1.72 0.3903 1.72 [0.95; 2.48] 25.1% 30.2%
Phase 2 1.05 0.2498 1.05 [0.56; 1.54] 61.2% 50.6%
Phase 3 1.79 0.5279 1.79 [0.75; 2.82] 13.7% 19.2%
Fixed effect model 1.32 [0.94; 1.70] 100% −−
Random effects model 1.39 [0.89; 1.90] −− 100%
Heterogeneity: I−squared=33.4%, p=0.2230
−2
−1
0
1
2
% DNA methylation difference

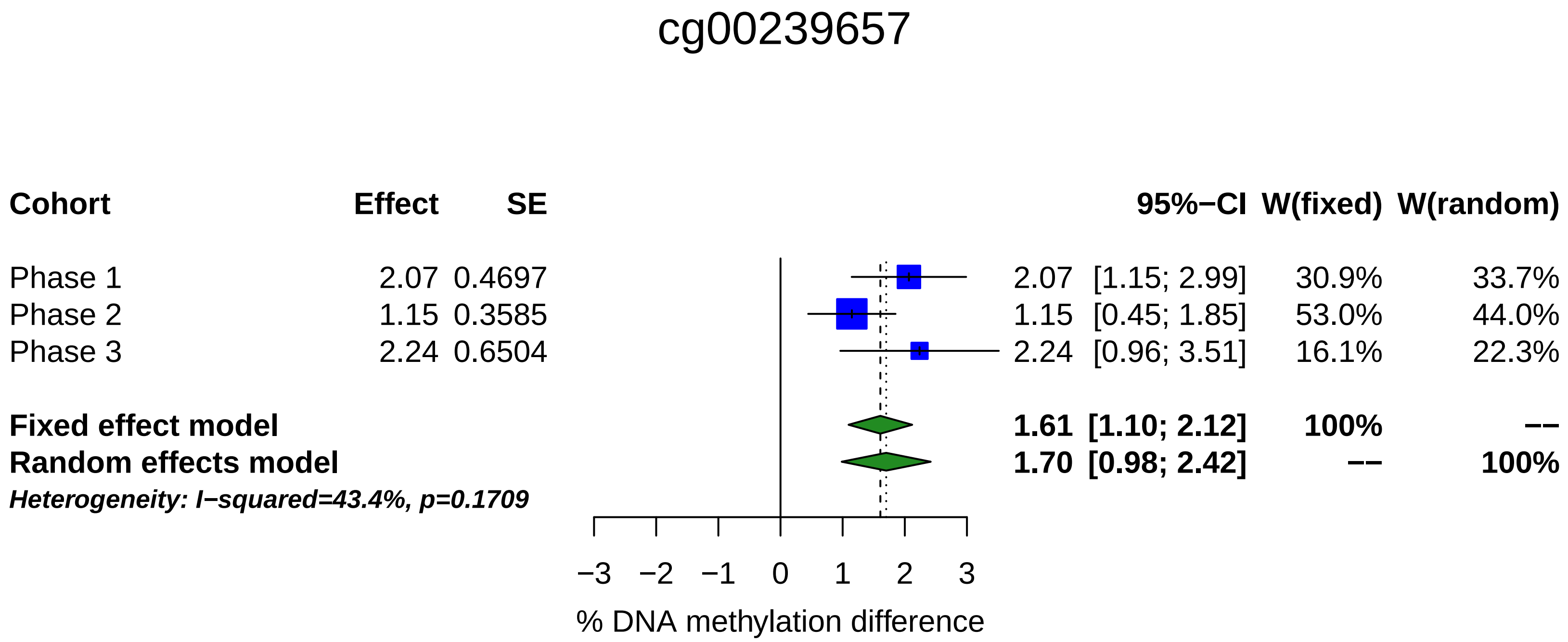
cg00239657
Cohort
Effect
SE
95%−CI
W(fixed)
W(random)
Phase 1 2.07 0.4697 2.07 [1.15; 2.99] 30.9% 33.7%
Phase 2 1.15 0.3585 1.15 [0.45; 1.85] 53.0% 44.0%
Phase 3 2.24 0.6504 2.24 [0.96; 3.51] 16.1% 22.3%
Fixed effect model 1.61 [1.10; 2.12] 100% −−
Random effects model 1.70 [0.98; 2.42] −− 100%
Heterogeneity: I−squared=43.4%, p=0.1709
−3 −2 −1 0 1 2 3
% DNA methylation difference

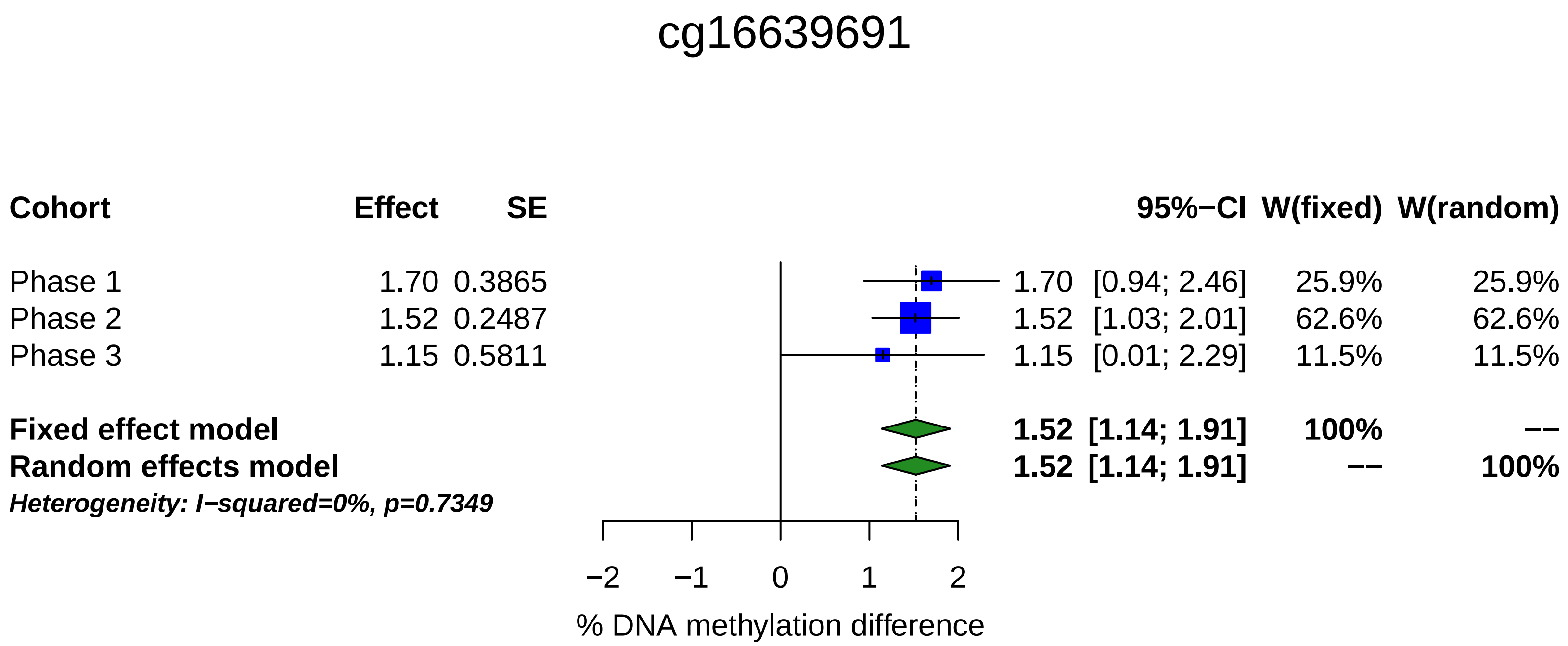
cg16639691
Cohort
Effect
SE
95%−CI
W(fixed)
W(random)
Phase 1 1.70 0.3865 1.70 [0.94; 2.46] 25.9% 25.9%
Phase 2 1.52 0.2487 1.52 [1.03; 2.01] 62.6% 62.6%
Phase 3 1.15 0.5811 1.15 [0.01; 2.29] 11.5% 11.5%
Fixed effect model 1.52 [1.14; 1.91] 100% −−
Random effects model 1.52 [1.14; 1.91] −− 100%
Heterogeneity: I−squared=0%, p=0.7349
−2
−1
0
1
2
% DNA methylation difference

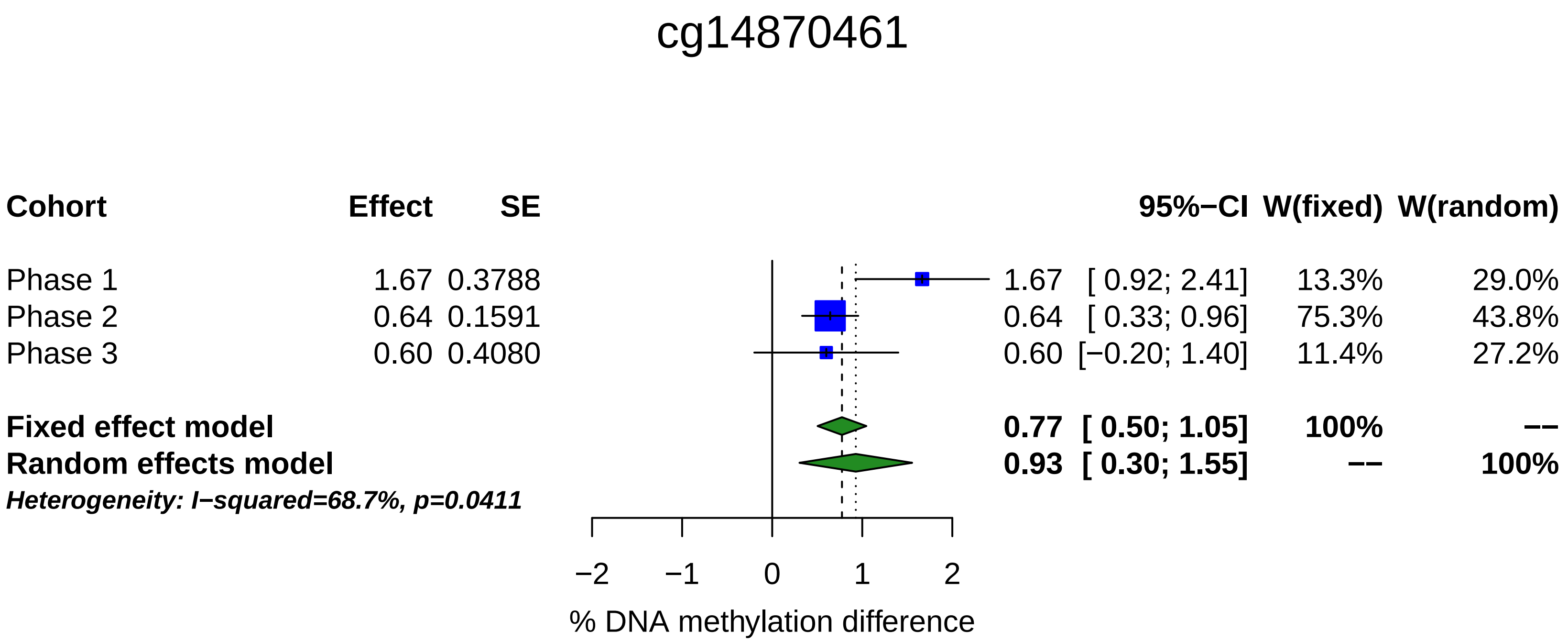
cg14870461
Cohort
Effect
SE
95%−CI
W(fixed)
W(random)
Phase 1 1.67 0.3788 1.67 [0.92; 2.41] 13.3% 29.0%
Phase 2 0.64 0.1591 0.64 [0.33; 0.96] 75.3% 43.8%
Phase 3 0.60 0.4080 0.60 [−0.20; 1.40] 11.4% 27.2%
Fixed effect model 0.77 [0.50; 1.05] 100% −−
Random effects model 0.93 [0.30; 1.55] −− 100%
Heterogeneity: I−squared=68.7%, p=0.0411
−2
−1
0
1
2
% DNA methylation difference

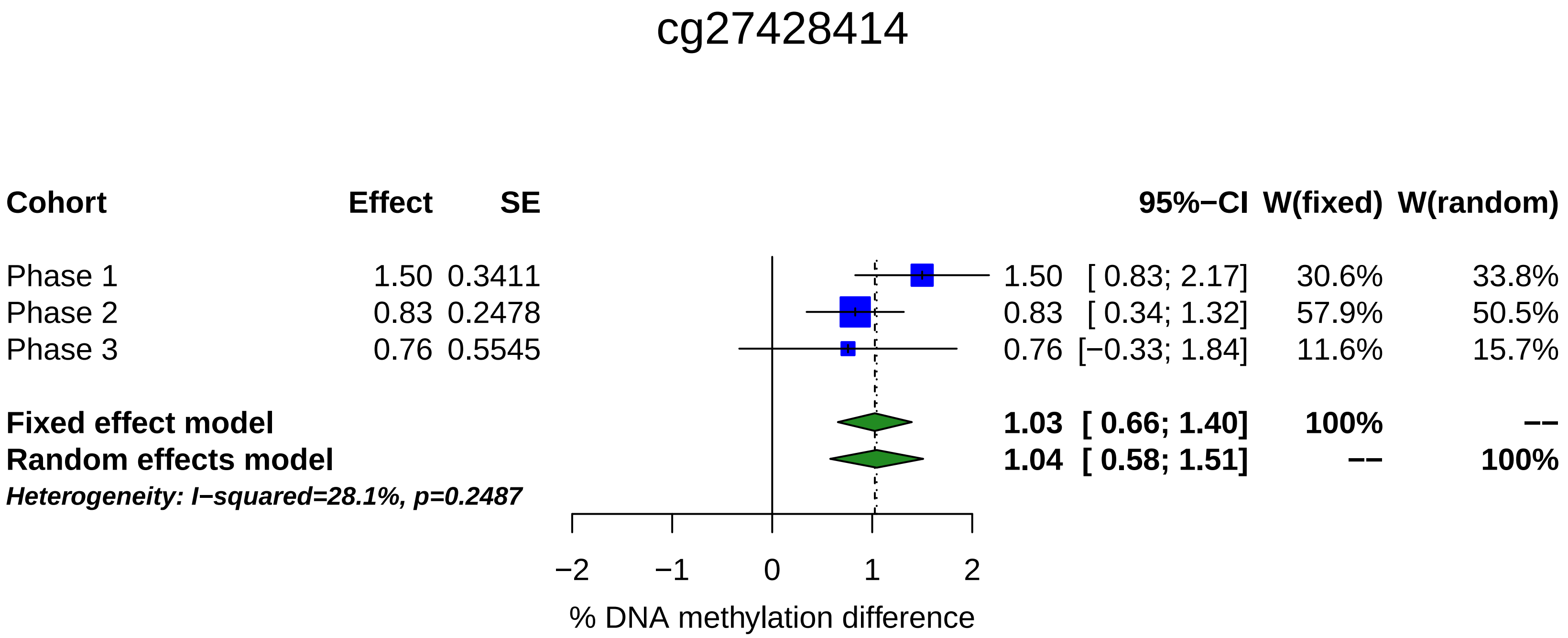
cg27428414
Cohort
Effect
SE
95%−CI
W(fixed)
W(random)
Phase 1 1.50 0.3411 1.50 [0.83; 2.17] 30.6% 33.8%
Phase 2 0.83 0.2478 0.83 [0.34; 1.32] 57.9% 50.5%
Phase 3 0.76 0.5545 0.76 [−0.33; 1.84] 11.6% 15.7%
Fixed effect model 1.03 [0.66; 1.40] 100% −−
Random effects model 1.04 [0.58; 1.51] −− 100%
Heterogeneity: I−squared=28.1%, p=0.2487
−2
−1
0
1
2
% DNA methylation difference

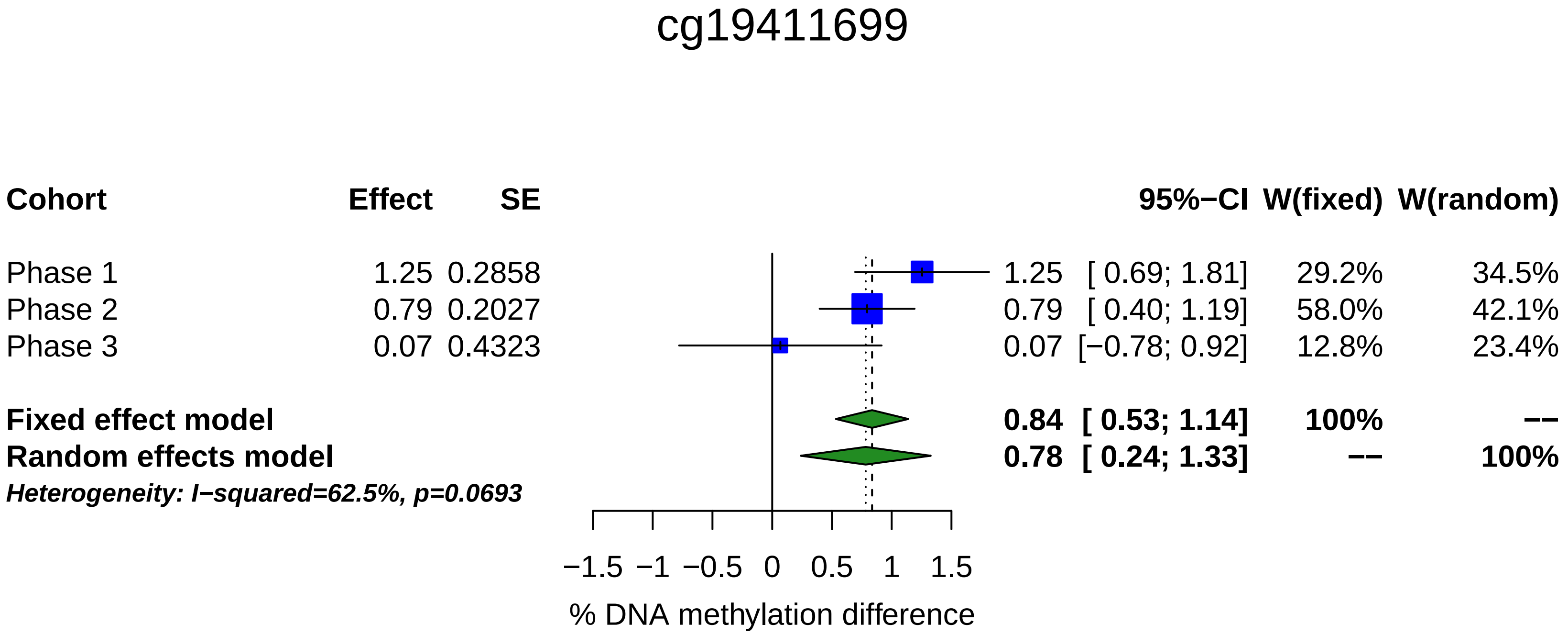
cg19411699
Cohort
Effect
SE
95%−CI
W(fixed)
W(random)
Phase 1
1.25
0.2858
1.25 [0.69; 1.81]
29.2%
34.5%
Phase 2
0.79
0.2027
0.79 [0.40; 1.19]
58.0%
42.1%
Phase 3
0.07
0.4323
0.07 [−0.78; 0.92]
12.8%
23.4%
Fixed effect model
0.84 [0.53; 1.14]
100%
−−
Random effects model
0.78 [0.24; 1.33]
−−
100%
Heterogeneity: I−squared=62.5%, p=0.0693
−1.5
−1
−0.5
0
0.5
1
1.5
% DNA methylation difference

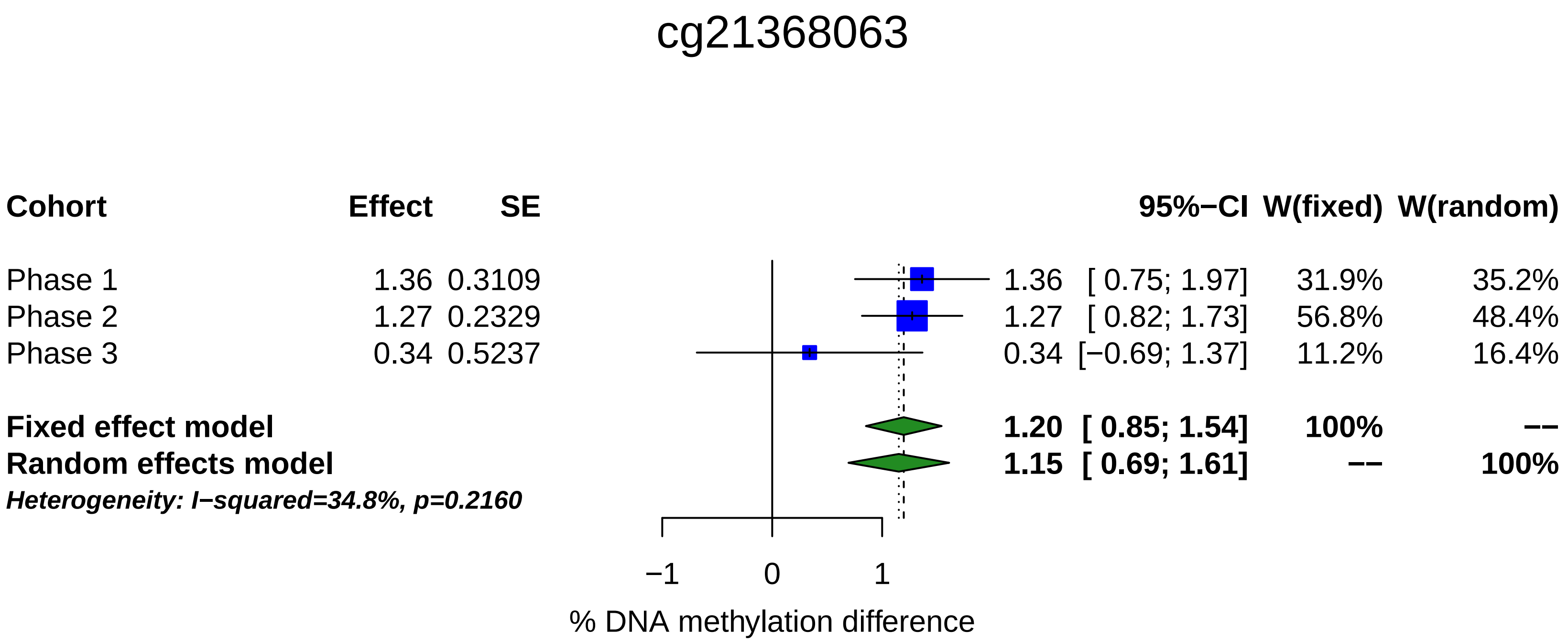
cg21368063
Cohort
Effect
SE
95%−CI
W(fixed)
W(random)
Phase 1 1.36 0.3109 1.36 [0.75; 1.97] 31.9% 35.2%
Phase 2 1.27 0.2329 1.27 [0.82; 1.73] 56.8% 48.4%
Phase 3 0.34 0.5237 0.34 [−0.69; 1.37] 11.2% 16.4%
Fixed effect model 1.20 [0.85; 1.54] 100% −−
Random effects model 1.15 [0.69; 1.61] −− 100%
Heterogeneity: I−squared=34.8%, p=0.2160
−1
0
1
% DNA methylation difference

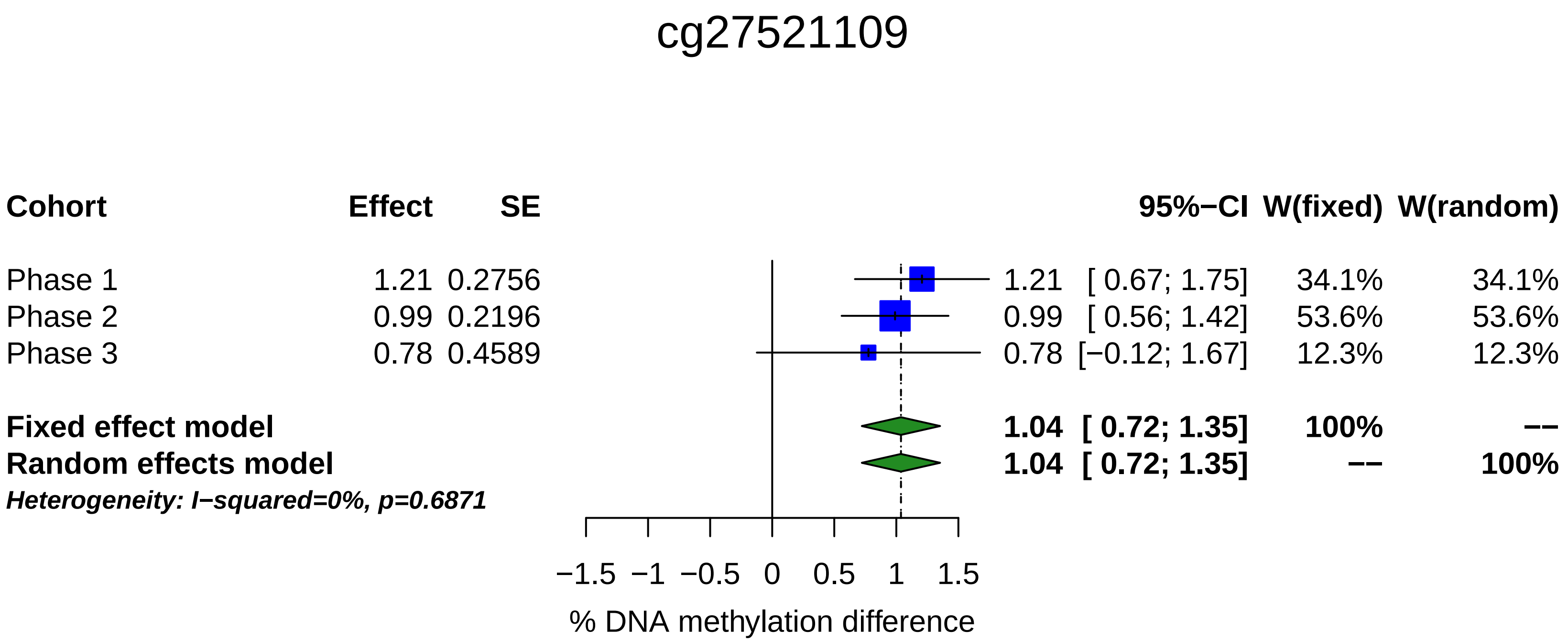
cg27521109
Cohort
Effect
SE
95%−CI
W(fixed)
W(random)
Phase 1 1.21 0.2756 1.21 [0.67; 1.75] 34.1% 34.1%
Phase 2 0.99 0.2196 0.99 [0.56; 1.42] 53.6% 53.6%
Phase 3 0.78 0.4589 0.78 [−0.12; 1.67] 12.3% 12.3%
Fixed effect model 1.04 [0.72; 1.35] 100% −−
Random effects model 1.04 [0.72; 1.35] −− 100%
Heterogeneity: I−squared=0%, p=0.6871
−1.5 −1 −0.5 0 0.5 1 1.5
% DNA methylation difference

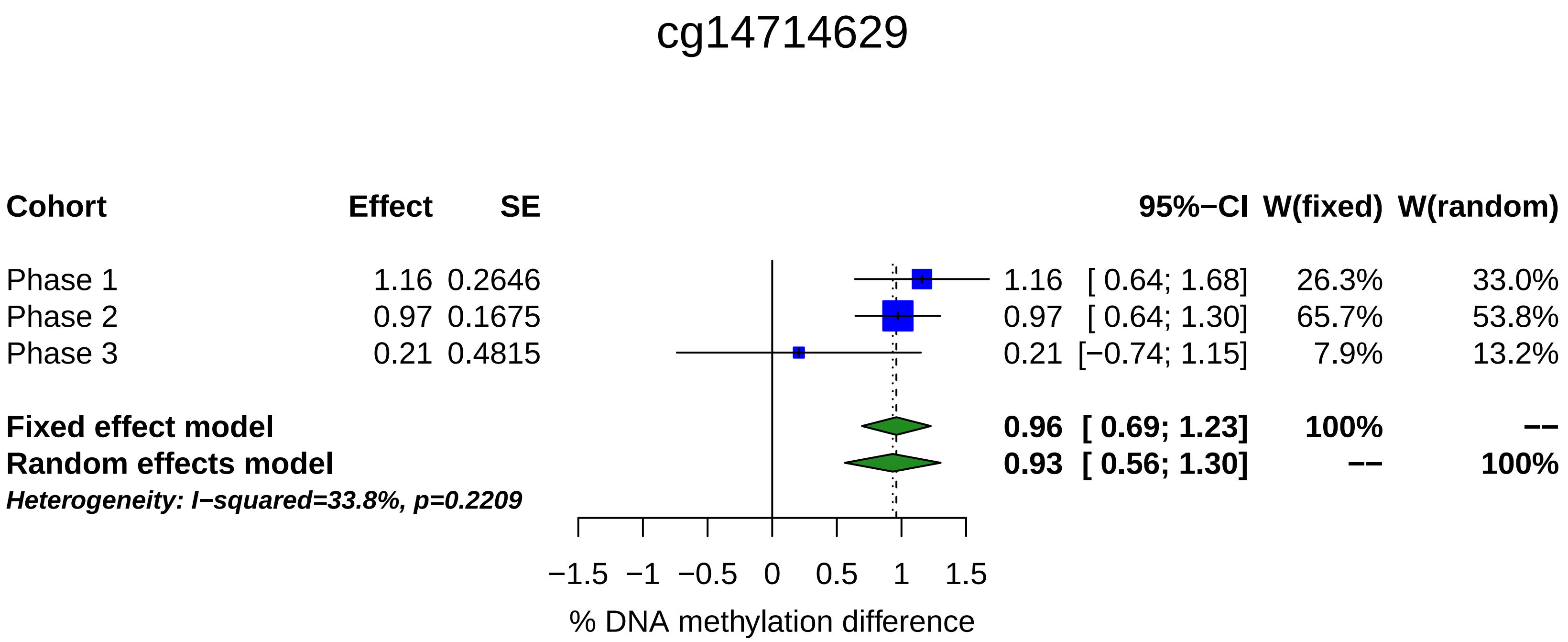
cg14714629
Cohort
Effect
SE
95%−CI
W(fixed)
W(random)
Phase 1 1.16 0.2646 1.16 [0.64; 1.68] 26.3% 33.0%
Phase 2 0.97 0.1675 0.97 [0.64; 1.30] 65.7% 53.8%
Phase 3 0.21 0.4815 0.21 [−0.74; 1.15] 7.9% 13.2%
Fixed effect model 0.96 [0.69; 1.23] 100% −−
Random effects model 0.93 [0.56; 1.30] −− 100%
Heterogeneity: I−squared=33.8%, p=0.2209
−1.5
−1
−0.5
0
0.5
1
1.5
% DNA methylation difference

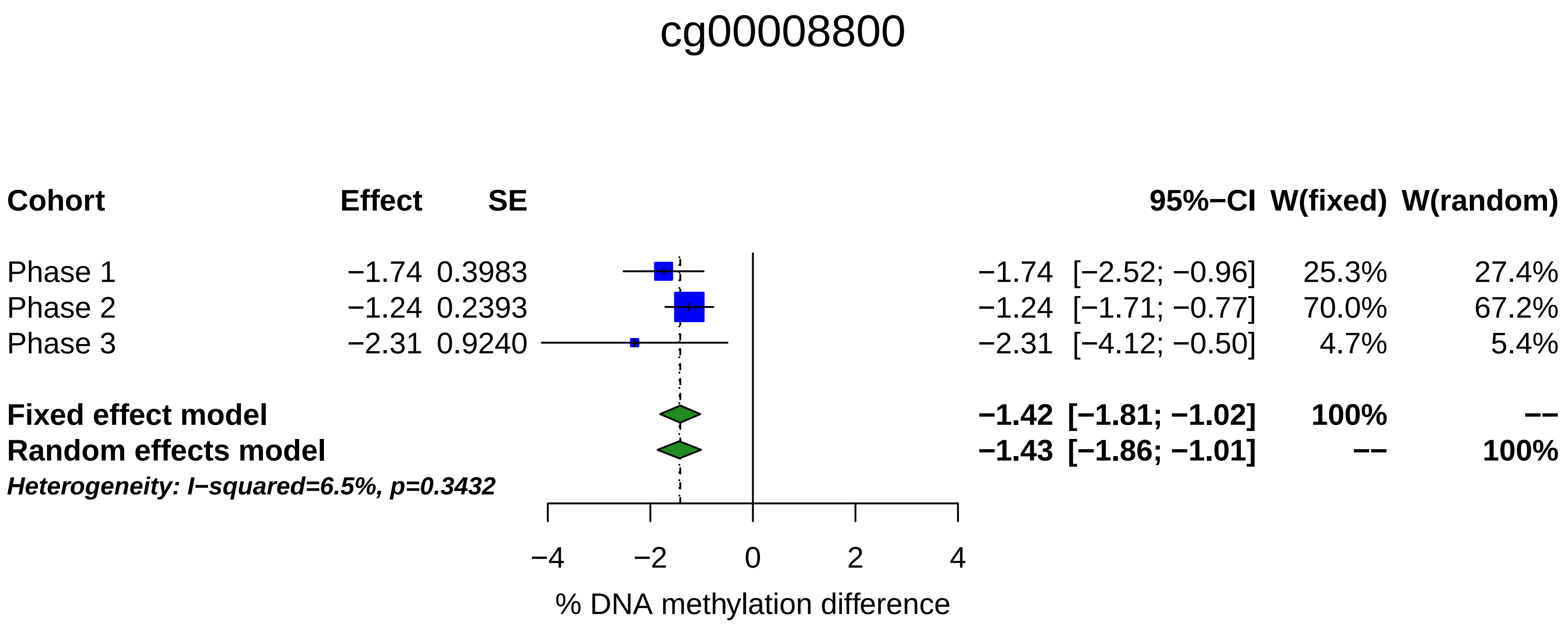
cg00008800
Cohort
Effect
SE
95%−CI
W(fixed)
W(random)
Phase 1 −1.74 0.3983 −1.74 [−2.52; −0.96] 25.3% 27.4%
Phase 2 −1.24 0.2393 −1.24 [−1.71; −0.77] 70.0% 67.2%
Phase 3 −2.31 0.9240 −2.31 [−4.12; −0.50] 4.7% 5.4%
Fixed effect model −1.42 [−1.81; −1.02] 100% −−
Random effects model −1.43 [−1.86; −1.01] −− 100%
Heterogeneity: I−squared=6.5%, p=0.3432
−4
−2
0
2
4
% DNA methylation difference

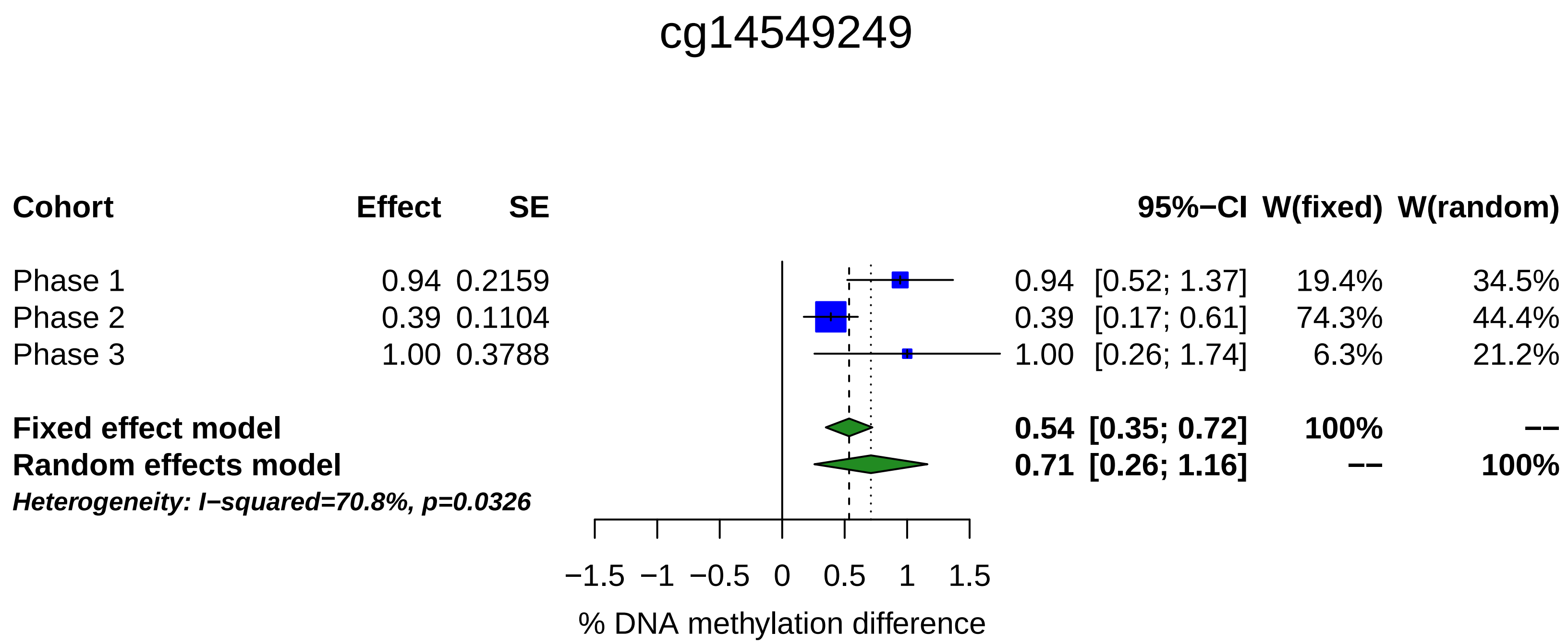
cg14549249
Cohort
Effect
SE
95%−CI
W(fixed)
W(random)
Phase 1 0.94 0.2159 0.94 [0.52; 1.37] 19.4% 34.5%
Phase 2 0.39 0.1104 0.39 [0.17; 0.61] 74.3% 44.4%
Phase 3 1.00 0.3788 1.00 [0.26; 1.74] 6.3% 21.2%
Fixed effect model 0.54 [0.35; 0.72] 100% −−
Random effects model 0.71 [0.26; 1.16] −− 100%
Heterogeneity: I−squared=70.8%, p=0.0326
−1.5 −1 −0.5 0 0.5 1 1.5
% DNA methylation difference

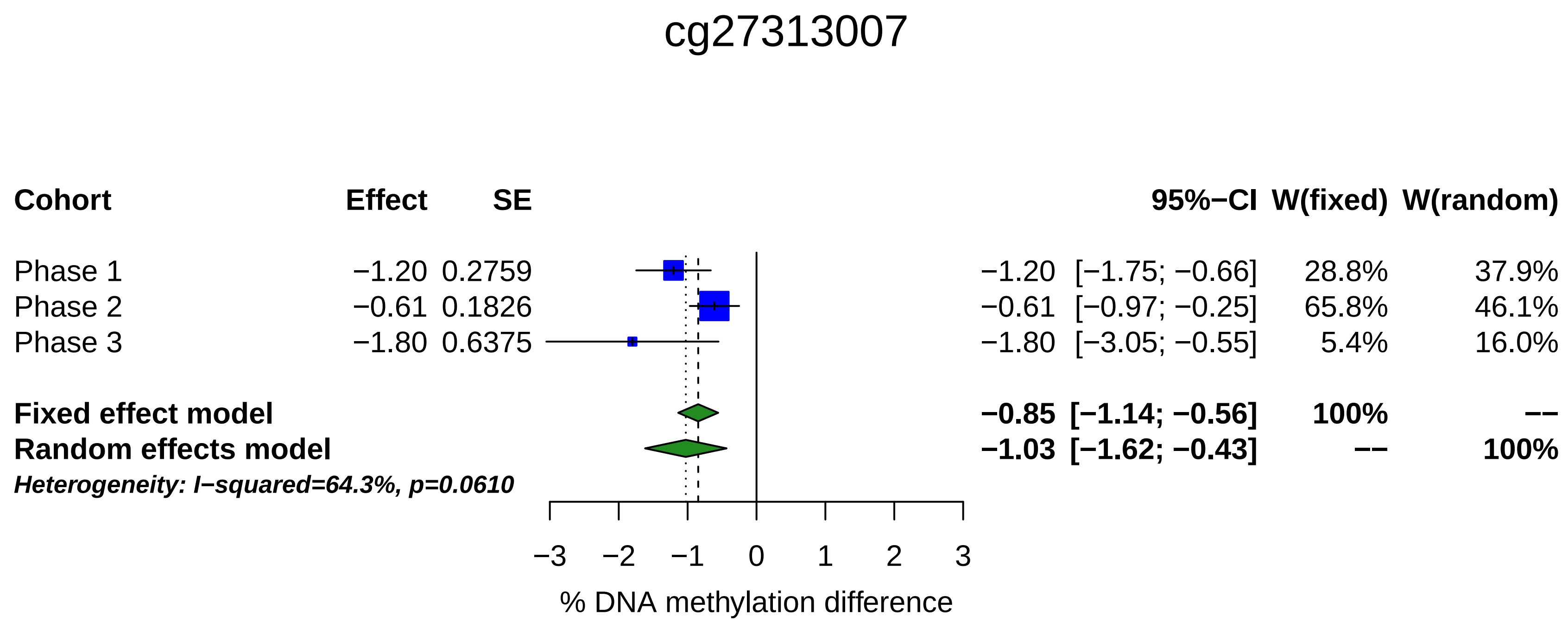
cg27313007
Cohort
Effect
SE
95%−CI
W(fixed)
W(random)
Phase 1 −1.20 0.2759 −1.20 [−1.75; −0.66] 28.8% 37.9%
Phase 2 −0.61 0.1826 −0.61 [−0.97; −0.25] 65.8% 46.1%
Phase 3 −1.80 0.6375 −1.80 [−3.05; −0.55] 5.4% 16.0%
Fixed effect model −0.85 [−1.14; −0.56] 100% −−
Random effects model −1.03 [−1.62; −0.43] −− 100%
Heterogeneity: I−squared=64.3%, p=0.0610
−3
−2
−1
0
1
2
3
% DNA methylation difference

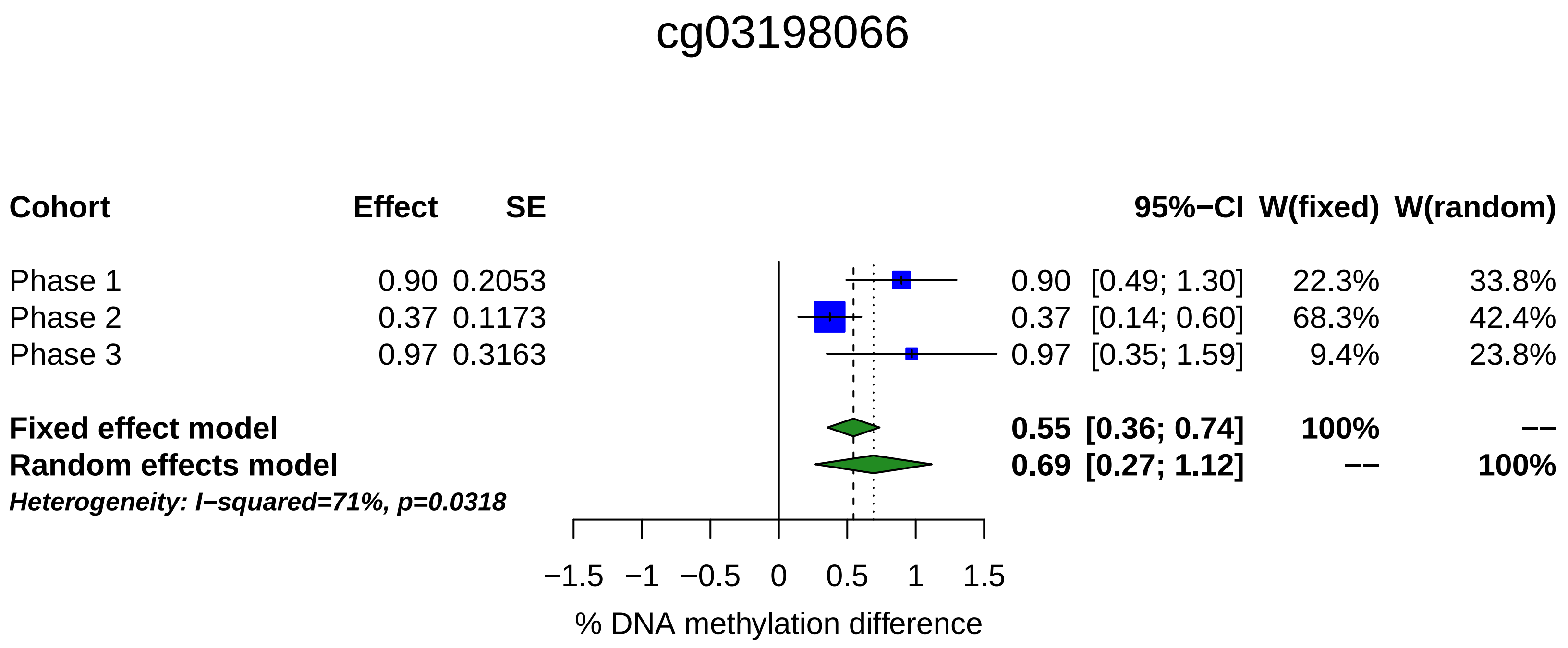
cg03198066
Cohort
Effect
SE
95%−CI
W(fixed)
W(random)
Phase 1 0.90 0.2053 0.90 [0.49; 1.30] 22.3% 33.8%
Phase 2 0.37 0.1173 0.37 [0.14; 0.60] 68.3% 42.4%
Phase 3 0.97 0.3163 0.97 [0.35; 1.59] 9.4% 23.8%
Fixed effect model 0.55 [0.36; 0.74] 100% −−
Random effects model 0.69 [0.27; 1.12] −− 100%
Heterogeneity: I−squared=71%, p=0.0318
−1.5 −1 −0.5 0 0.5 1 1.5
% DNA methylation difference

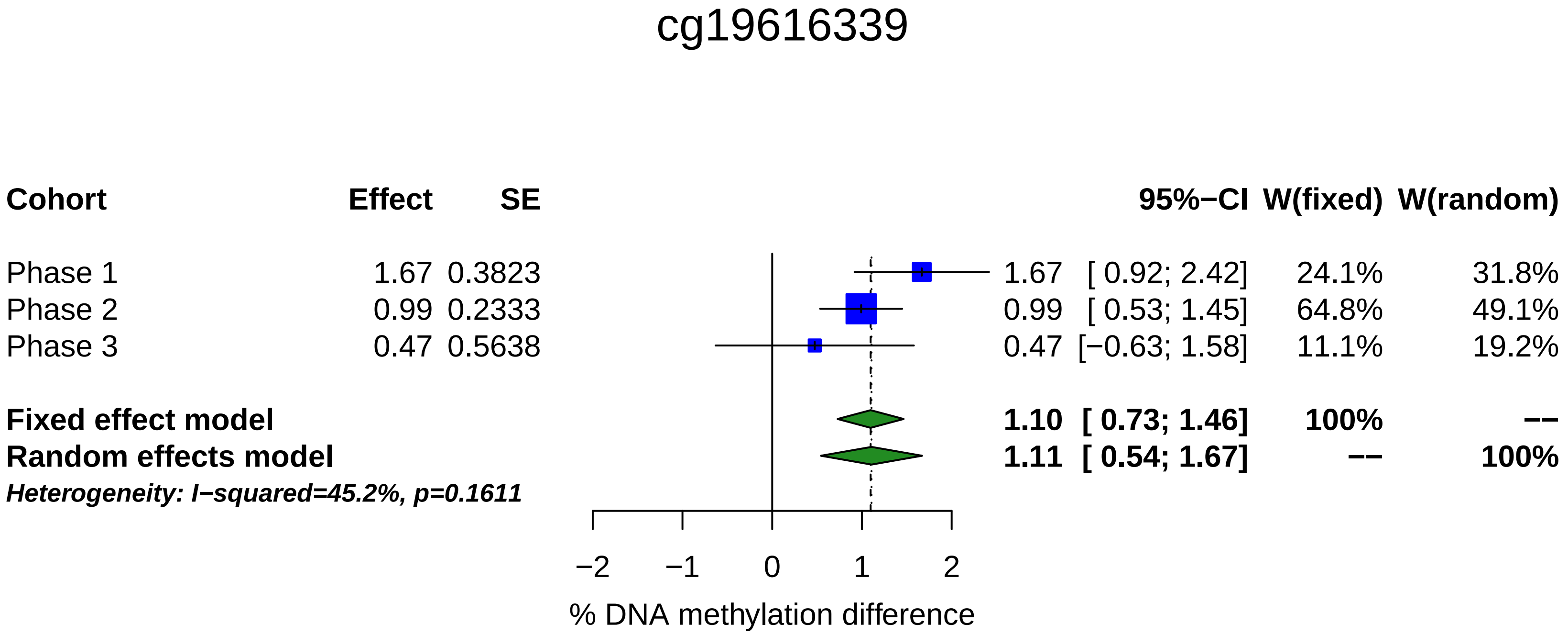
cg19616339
Cohort
Effect
SE
95%−CI
W(fixed)
W(random)
Phase 1 1.67 0.3823 1.67 [0.92; 2.42] 24.1% 31.8%
Phase 2 0.99 0.2333 0.99 [0.53; 1.45] 64.8% 49.1%
Phase 3 0.47 0.5638 0.47 [−0.63; 1.58] 11.1% 19.2%
Fixed effect model 1.10 [0.73; 1.46] 100% −−
Random effects model 1.11 [0.54; 1.67] −− 100%
Heterogeneity: I−squared=45.2%, p=0.1611
−2
−1
0
1
2
% DNA methylation difference

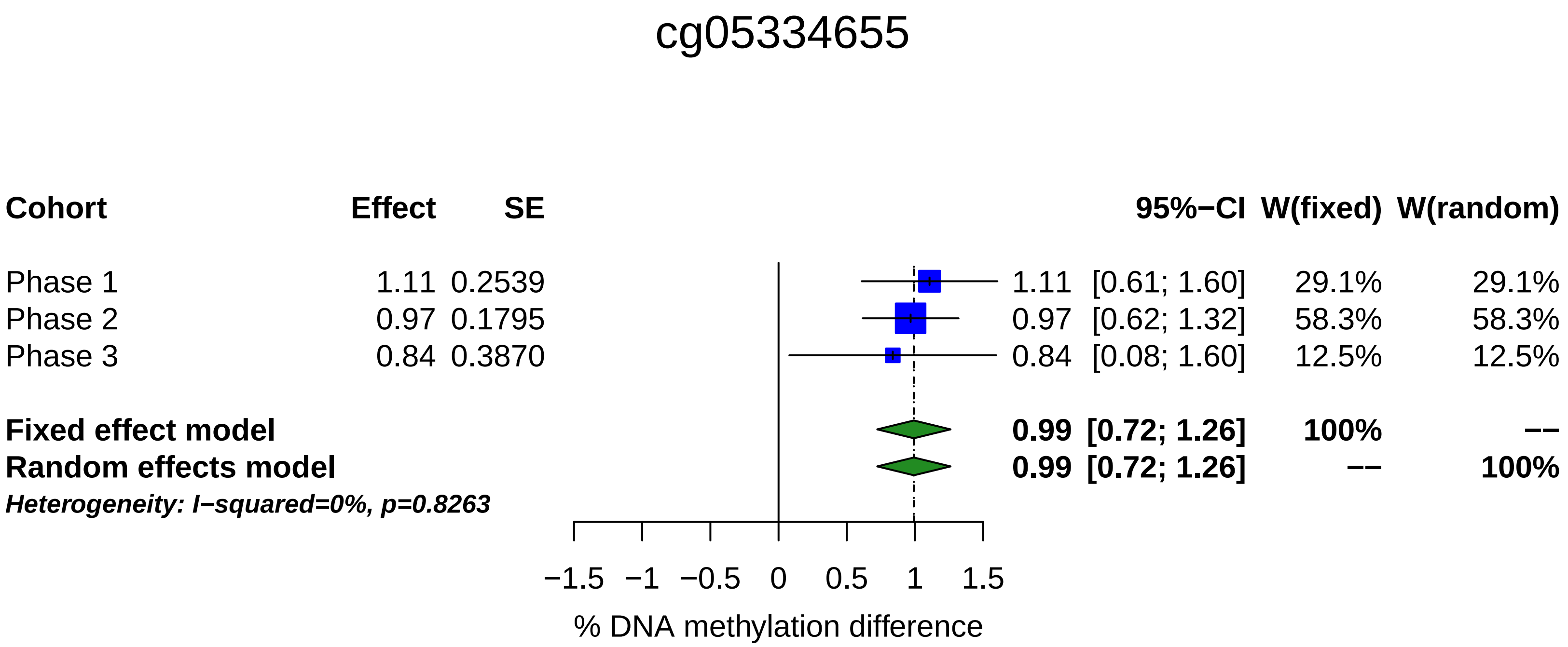
cg05334655
Cohort
Effect
SE
95%−CI
W(fixed)
W(random)
Phase 1 1.11 0.2539 1.11 [0.61; 1.60] 29.1% 29.1%
Phase 2 0.97 0.1795 0.97 [0.62; 1.32] 58.3% 58.3%
Phase 3 0.84 0.3870 0.84 [0.08; 1.60] 12.5% 12.5%
Fixed effect model 0.99 [0.72; 1.26] 100% −−
Random effects model 0.99 [0.72; 1.26] −− 100%
Heterogeneity: I−squared=0%, p=0.8263
−1.5 −1 −0.5 0 0.5 1 1.5
% DNA methylation difference

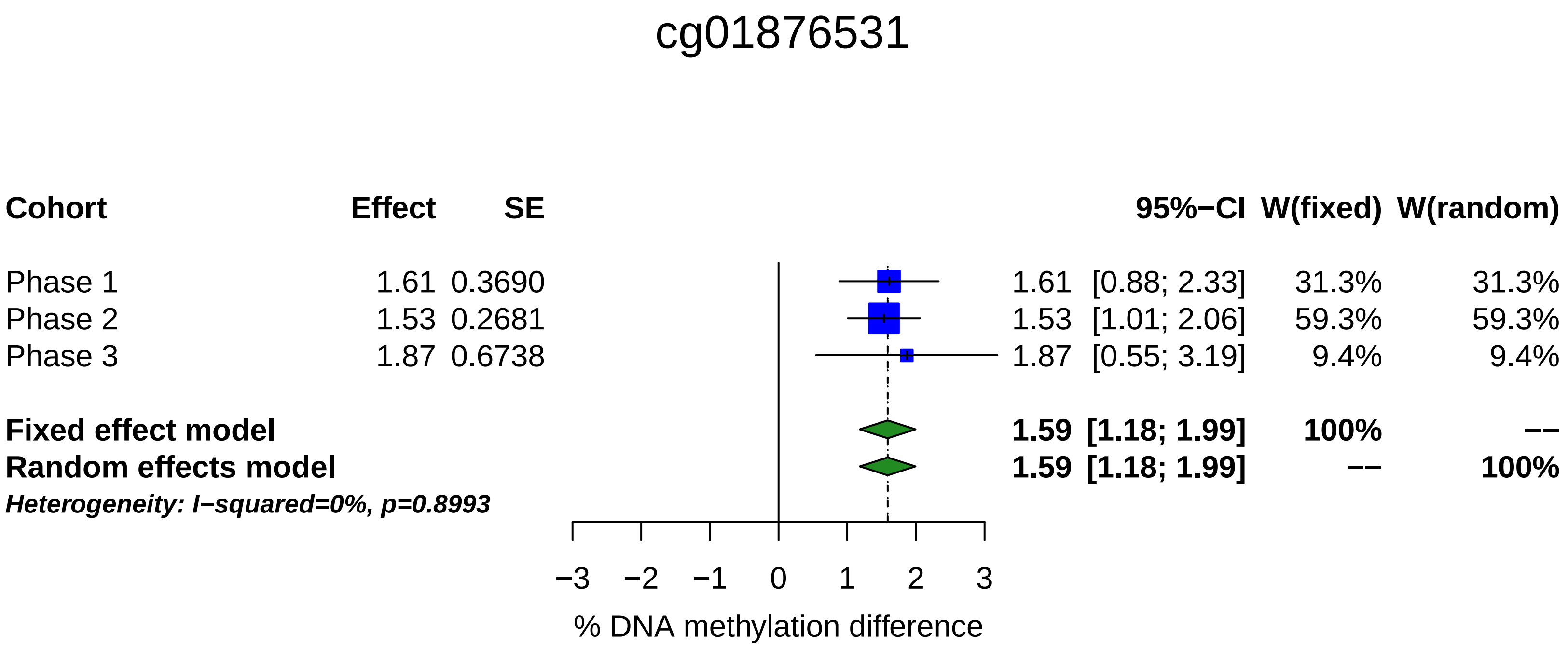
cg01876531
Cohort
Effect
SE
95%−CI
W(fixed)
W(random)
Phase 1 1.61 0.3690 1.61 [0.88; 2.33] 31.3% 31.3%
Phase 2 1.53 0.2681 1.53 [1.01; 2.06] 59.3% 59.3%
Phase 3 1.87 0.6738 1.87 [0.55; 3.19] 9.4% 9.4%
Fixed effect model 1.59 [1.18; 1.99] 100% −−
Random effects model 1.59 [1.18; 1.99] −− 100%
Heterogeneity: I−squared=0%, p=0.8993
−3 −2 −1 0 1 2 3
% DNA methylation difference

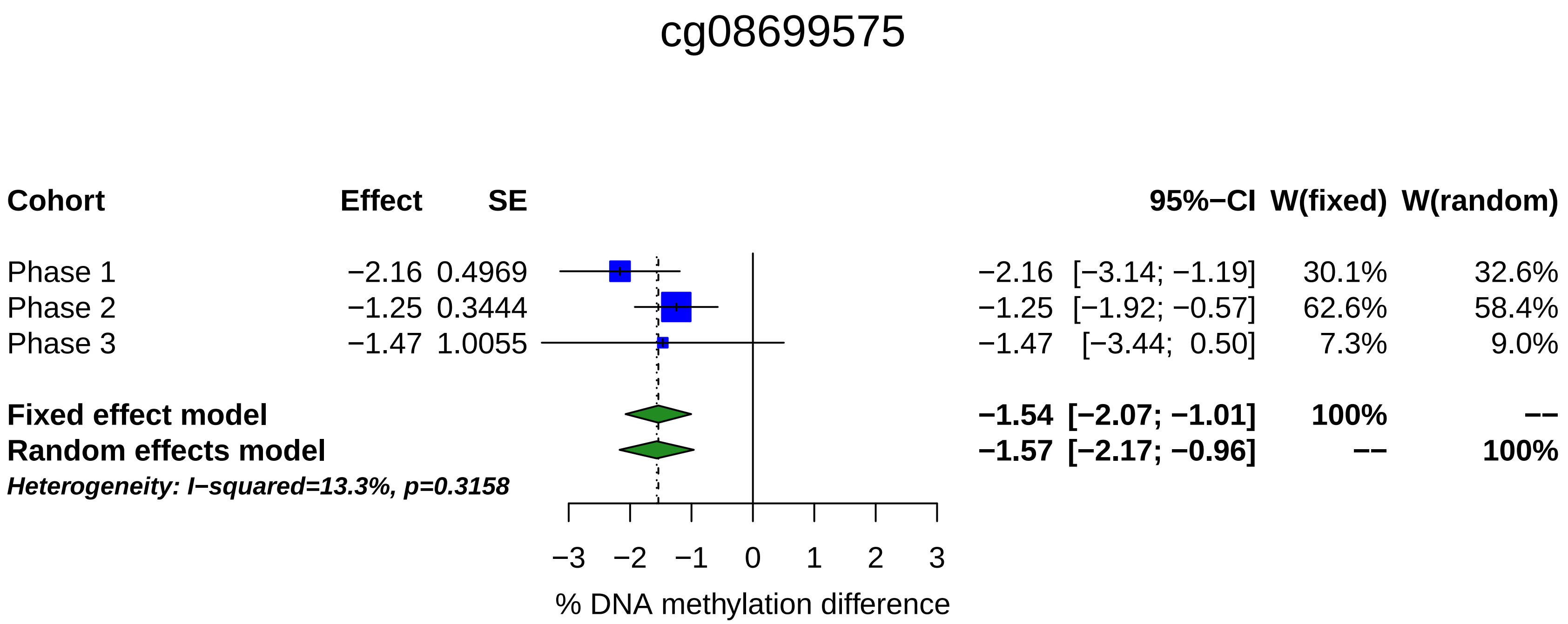
cg08699575
Cohort
Effect
SE
95%−CI
W(fixed)
W(random)
Phase 1 −2.16 0.4969 −2.16 [−3.14; −1.19] 30.1% 32.6%
Phase 2 −1.25 0.3444 −1.25 [−1.92; −0.57] 62.6% 58.4%
Phase 3 −1.47 1.0055 −1.47 [−3.44; 0.50] 7.3% 9.0%
Fixed effect model −1.54 [−2.07; −1.01] 100% −−
Random effects model −1.57 [−2.17; −0.96] −− 100%
Heterogeneity: I−squared=13.3%, p=0.3158
−3
−2
−1
0
1
2
3
% DNA methylation difference

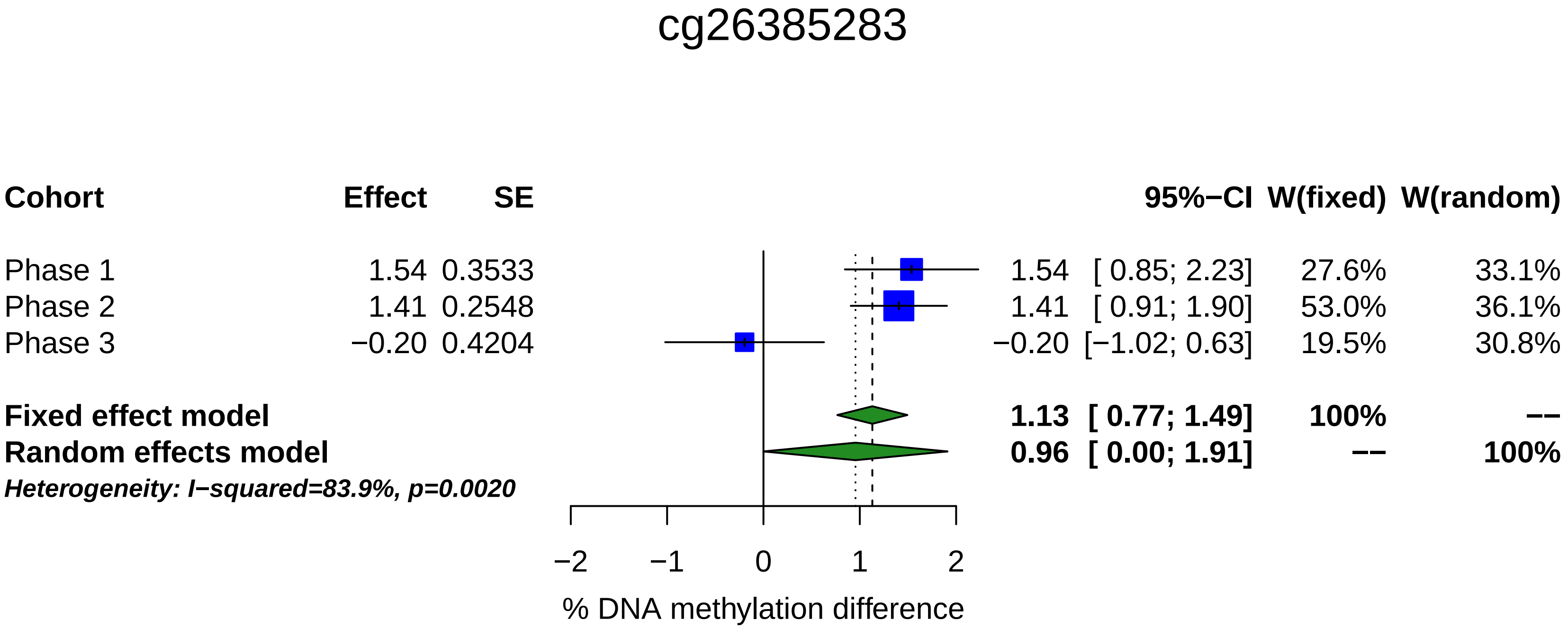
cg26385283
Cohort
Effect
SE
95%−CI
W(fixed)
W(random)
Phase 1
1.54
0.3533
1.54 [0.85; 2.23]
27.6%
33.1%
Phase 2
1.41
0.2548
1.41 [0.91; 1.90]
53.0%
36.1%
Phase 3
−0.20
0.4204
−0.20 [−1.02; 0.63]
19.5%
30.8%
Fixed effect model
1.13 [0.77; 1.49]
100%
−−
Random effects model
0.96 [0.00; 1.91]
−−
100%
Heterogeneity: I−squared=83.9%, p=0.0020
−2
−1
0
1
2
% DNA methylation difference

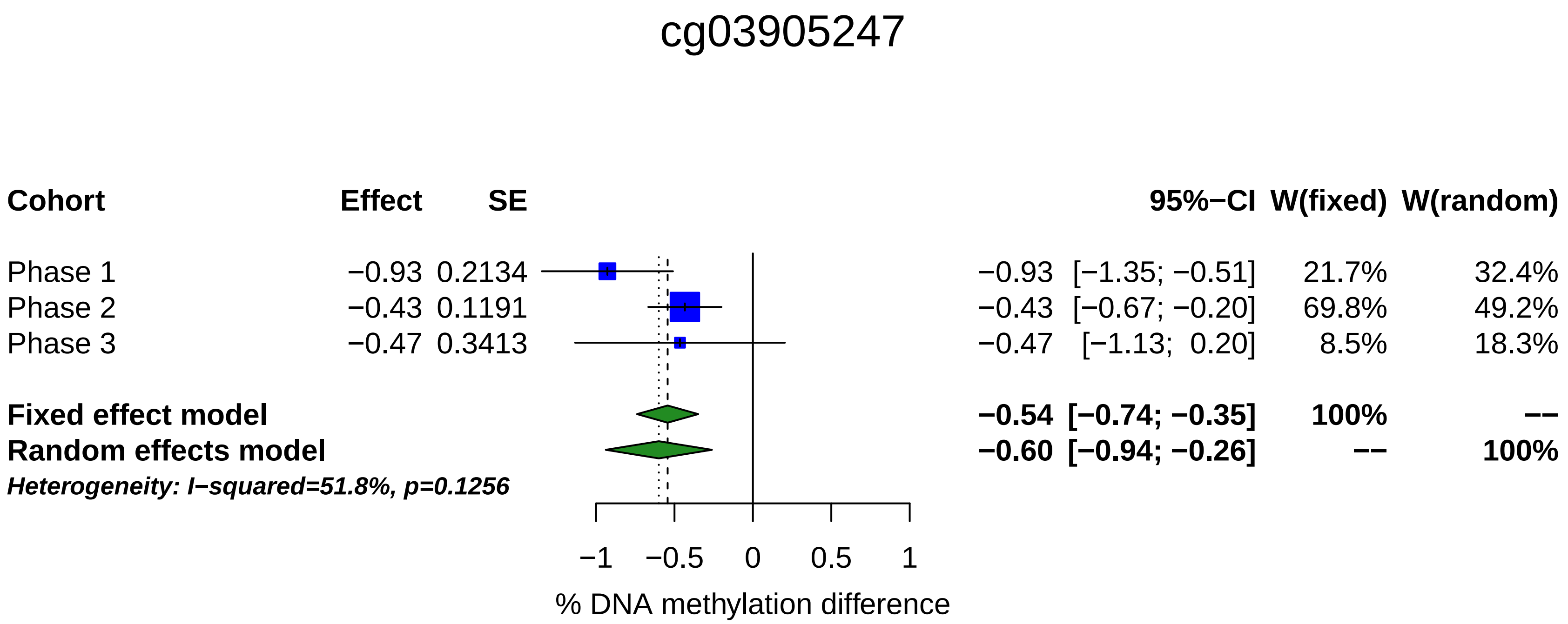
cg03905247
Cohort
Effect
SE
95%−CI
W(fixed)
W(random)
Phase 1 −0.93 0.2134 −0.93 [−1.35; −0.51] 21.7% 32.4%
Phase 2 −0.43 0.1191 −0.43 [−0.67; −0.20] 69.8% 49.2%
Phase 3 −0.47 0.3413 −0.47 [−1.13; 0.20] 8.5% 18.3%
Fixed effect model −0.54 [−0.74; −0.35] 100% −−
Random effects model −0.60 [−0.94; −0.26] −− 100%
Heterogeneity: I−squared=51.8%, p=0.1256
−1
−0.5
0
0.5
1
% DNA methylation difference

cg00218893

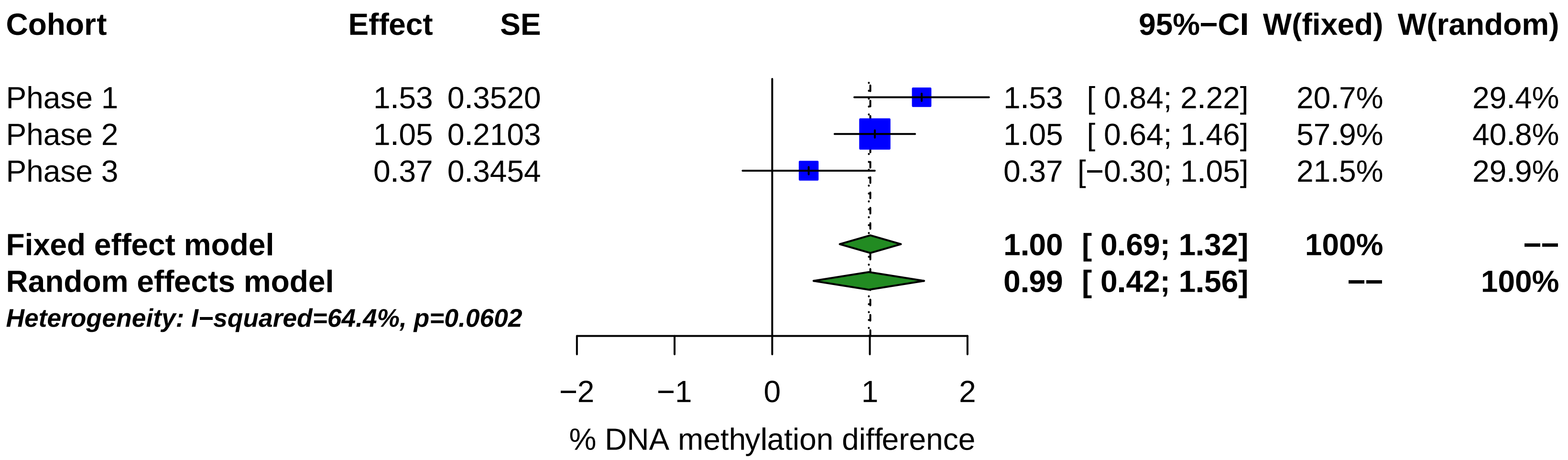
Cohort
Effect
SE
95%−CI
W(fixed)
W(random)
Phase 1 1.53 0.3520 1.53 [0.84; 2.22] 20.7% 29.4%
Phase 2 1.05 0.2103 1.05 [0.64; 1.46] 57.9% 40.8%
Phase 3 0.37 0.3454 0.37 [−0.30; 1.05] 21.5% 29.9%
Fixed effect model 1.00 [0.69; 1.32] 100% −−
Random effects model 0.99 [0.42; 1.56] −− 100%
Heterogeneity: I−squared=64.4%, p=0.0602
−2
−1
0
1
2
% DNA methylation difference

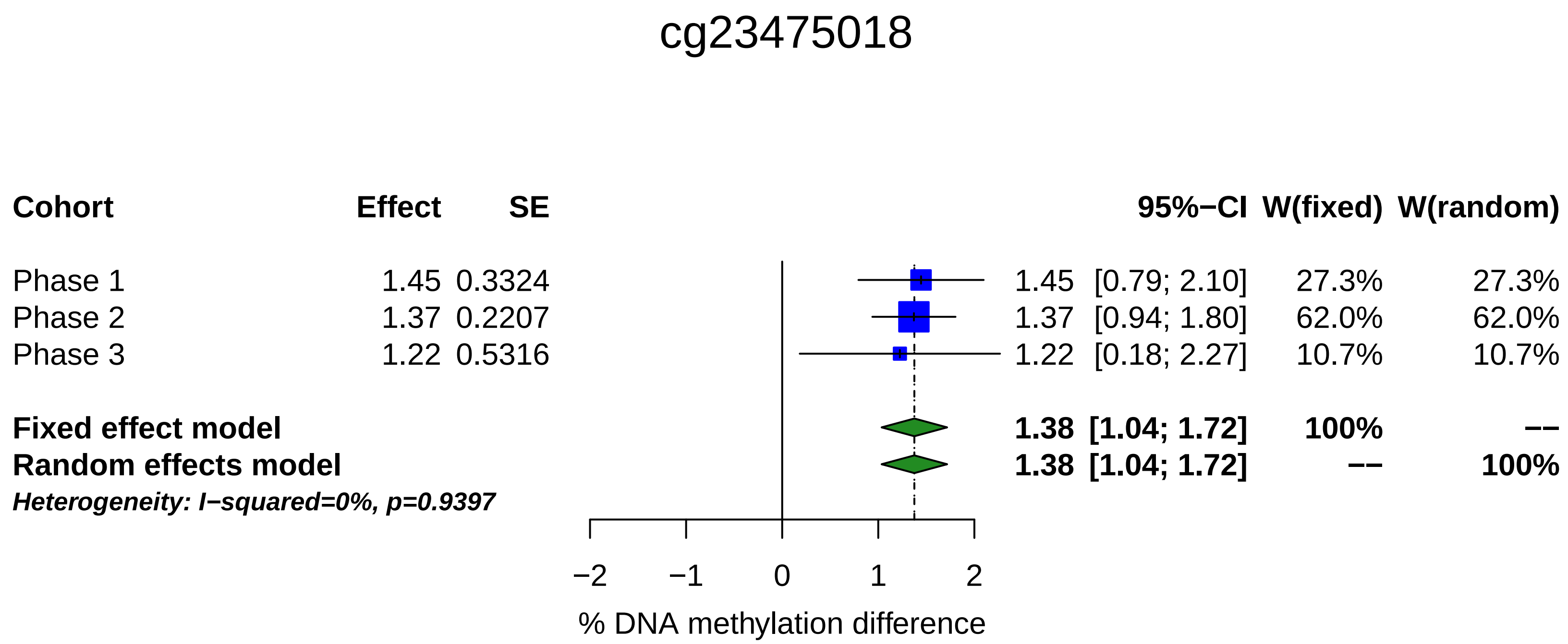
cg23475018
Cohort
Effect
SE
95%−CI
W(fixed)
W(random)
Phase 1
1.45
0.3324
1.45 [0.79; 2.10]
27.3%
27.3%
Phase 2
1.37
0.2207
1.37 [0.94; 1.80]
62.0%
62.0%
Phase 3
1.22
0.5316
1.22 [0.18; 2.27]
10.7%
10.7%
Fixed effect model
1.38 [1.04; 1.72]
100%
−−
Random effects model
1.38 [1.04; 1.72]
−−
100%
Heterogeneity: I−squared=0%, p=0.9397
−2
−1
0
1
2
% DNA methylation difference

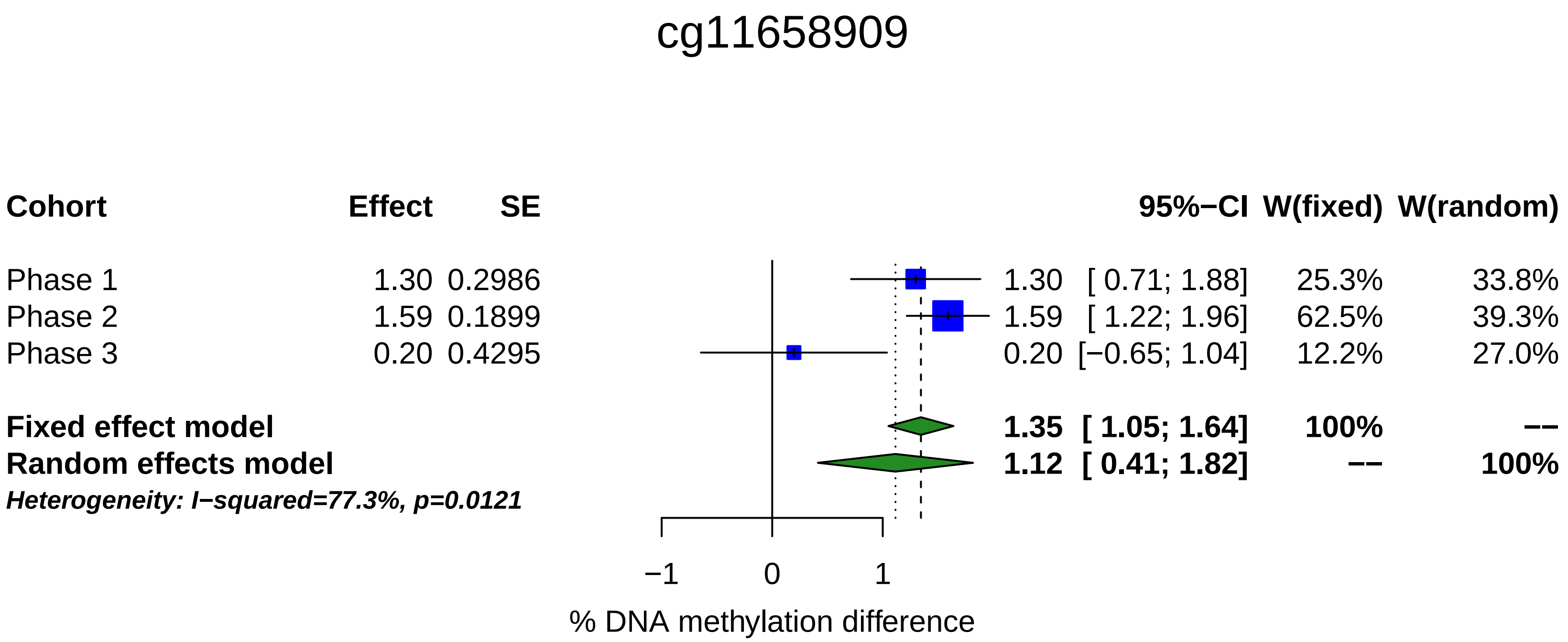
cg11658909
Cohort
Effect
SE
95%−CI
W(fixed)
W(random)
Phase 1 1.30 0.2986 1.30 [0.71; 1.88] 25.3% 33.8%
Phase 2 1.59 0.1899 1.59 [1.22; 1.96] 62.5% 39.3%
Phase 3 0.20 0.4295 0.20 [−0.65; 1.04] 12.2% 27.0%
Fixed effect model 1.35 [1.05; 1.64] 100% −−
Random effects model 1.12 [0.41; 1.82] −− 100%
Heterogeneity: I−squared=77.3%, p=0.0121
−1
0
1
% DNA methylation difference

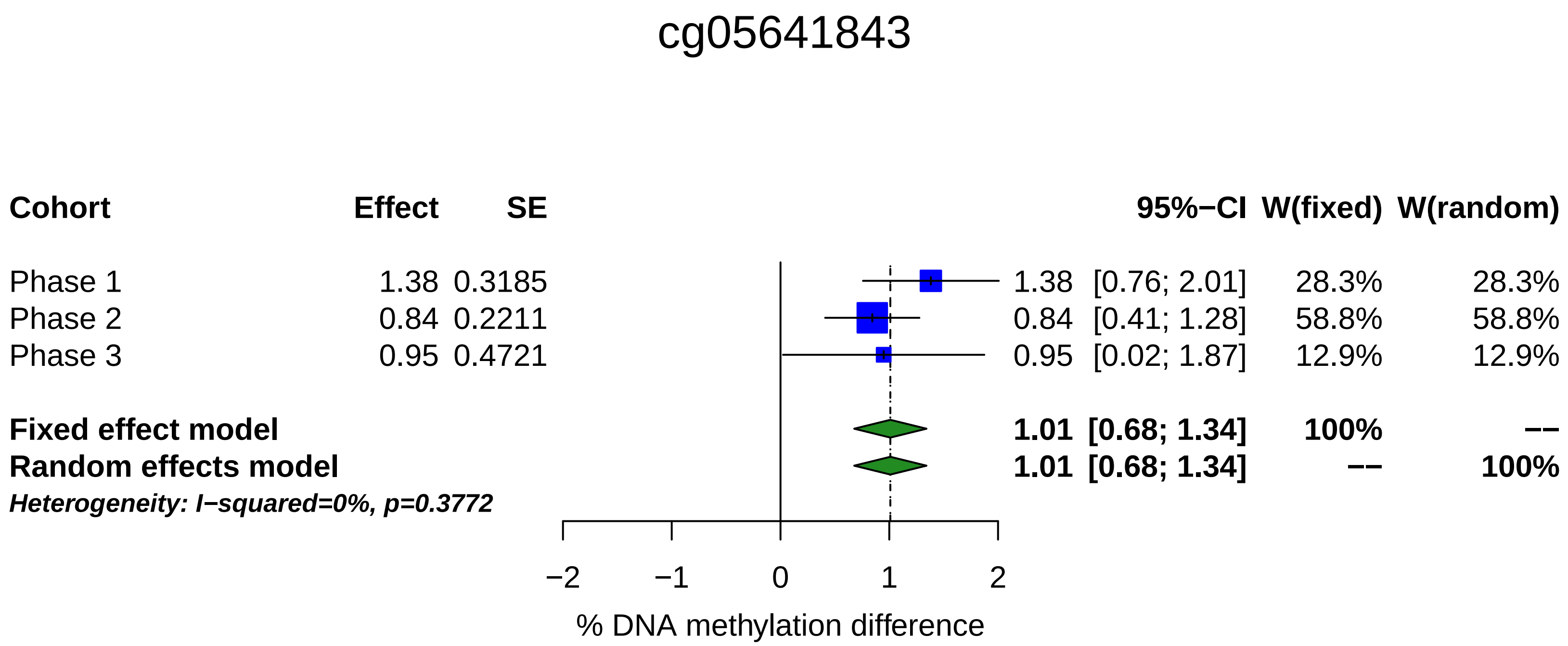
cg05641843
Cohort
Effect
SE
95%−CI
W(fixed)
W(random)
Phase 1 1.38 0.3185 1.38 [0.76; 2.01] 28.3% 28.3%
Phase 2 0.84 0.2211 0.84 [0.41; 1.28] 58.8% 58.8%
Phase 3 0.95 0.4721 0.95 [0.02; 1.87] 12.9% 12.9%
Fixed effect model 1.01 [0.68; 1.34] 100% −−
Random effects model 1.01 [0.68; 1.34] −− 100%
Heterogeneity: I−squared=0%, p=0.3772
−2
−1
0
1
2
% DNA methylation difference

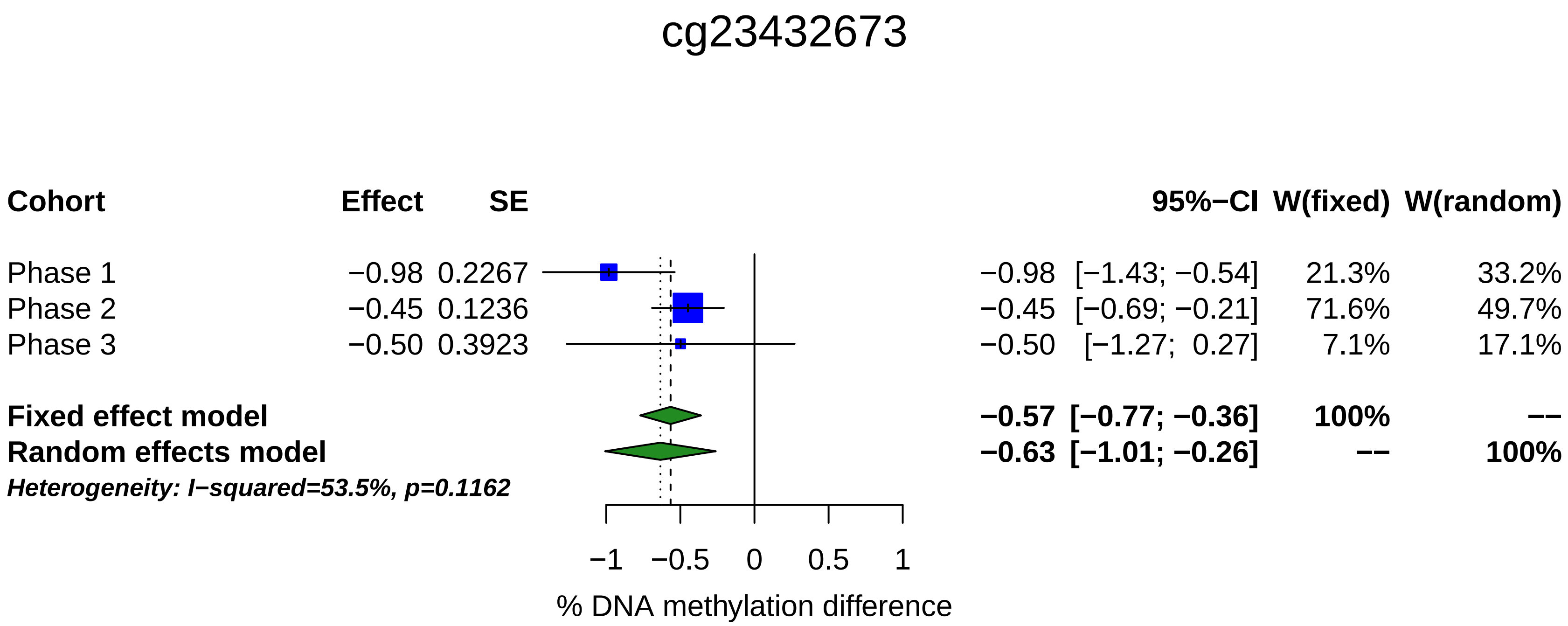
cg23432673
Cohort
Effect
SE
95%−CI
W(fixed)
W(random)
Phase 1 −0.98 0.2267 −0.98 [−1.43; −0.54] 21.3% 33.2%
Phase 2 −0.45 0.1236 −0.45 [−0.69; −0.21] 71.6% 49.7%
Phase 3 −0.50 0.3923 −0.50 [−1.27; 0.27] 7.1% 17.1%
Fixed effect model −0.57 [−0.77; −0.36] 100% −−
Random effects model −0.63 [−1.01; −0.26] −− 100%
Heterogeneity: I−squared=53.5%, p=0.1162
−1
−0.5
0
0.5
1
% DNA methylation difference

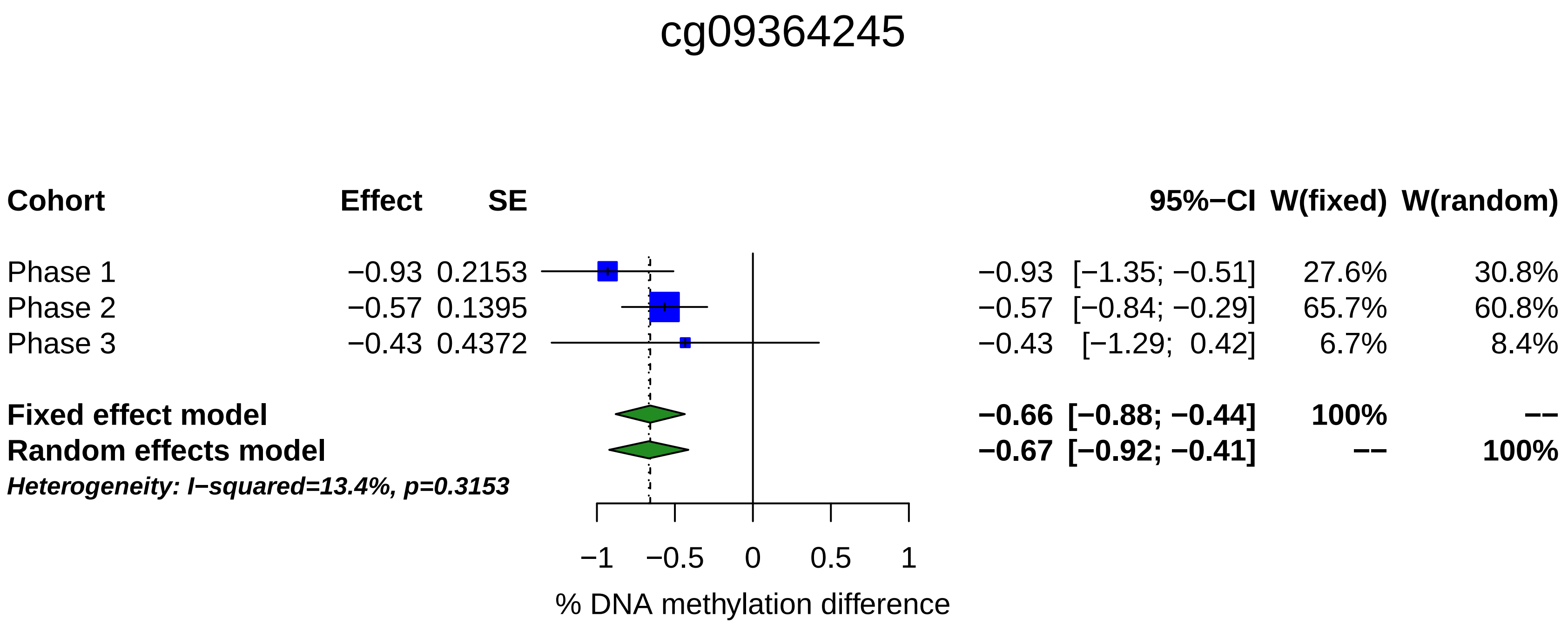
cg09364245
Cohort
Effect
SE
95%−CI
W(fixed)
W(random)
Phase 1 −0.93 0.2153 −0.93 [−1.35; −0.51] 27.6% 30.8%
Phase 2 −0.57 0.1395 −0.57 [−0.84; −0.29] 65.7% 60.8%
Phase 3 −0.43 0.4372 −0.43 [−1.29; 0.42] 6.7% 8.4%
Fixed effect model −0.66 [−0.88; −0.44] 100% −−
Random effects model −0.67 [−0.92; −0.41] −− 100%
Heterogeneity: I−squared=13.4%, p=0.3153
−1
−0.5
0
0.5
1
% DNA methylation difference

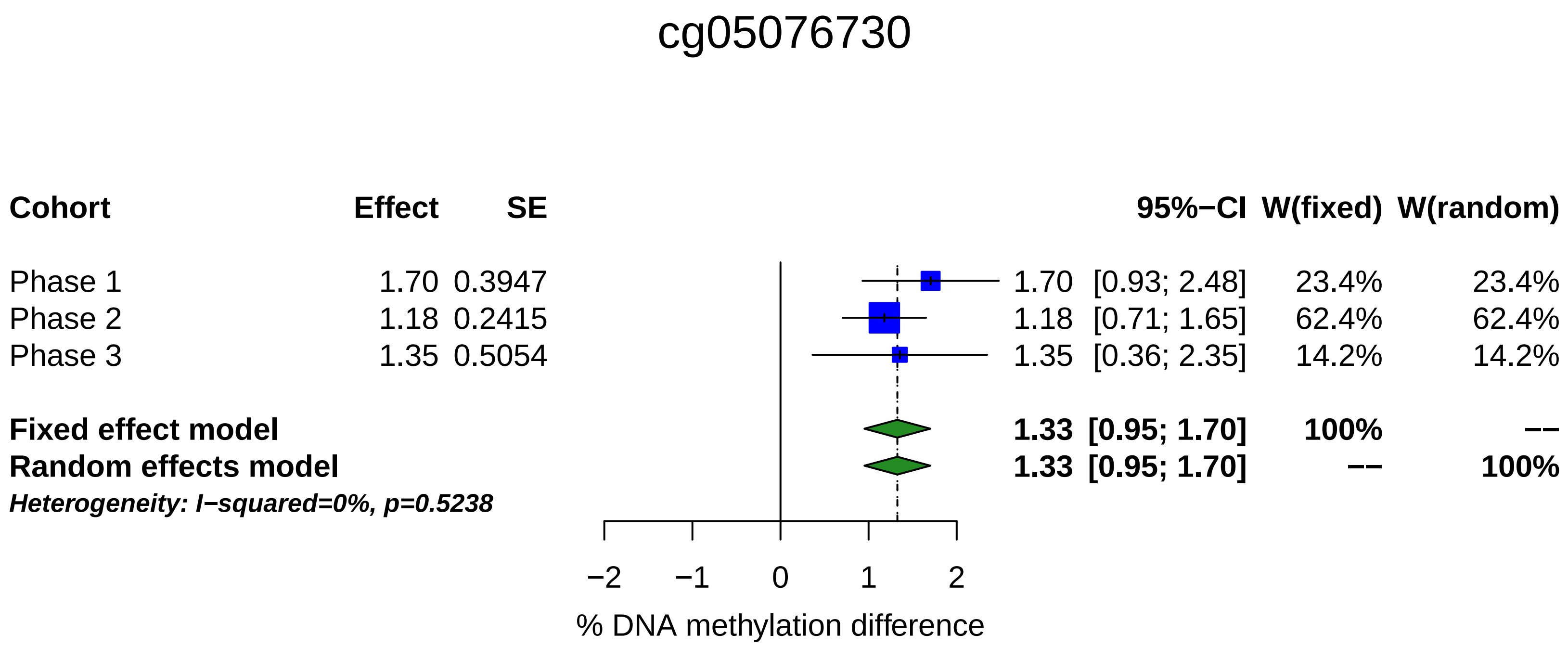
cg05076730
Cohort
Effect
SE
95%−CI
W(fixed)
W(random)
Phase 1 1.70 0.3947 1.70 [0.93; 2.48] 23.4% 23.4%
Phase 2 1.18 0.2415 1.18 [0.71; 1.65] 62.4% 62.4%
Phase 3 1.35 0.5054 1.35 [0.36; 2.35] 14.2% 14.2%
Fixed effect model 1.33 [0.95; 1.70] 100% −−
Random effects model 1.33 [0.95; 1.70] −− 100%
Heterogeneity: I−squared=0%, p=0.5238
−2
−1
0
1
2
% DNA methylation difference

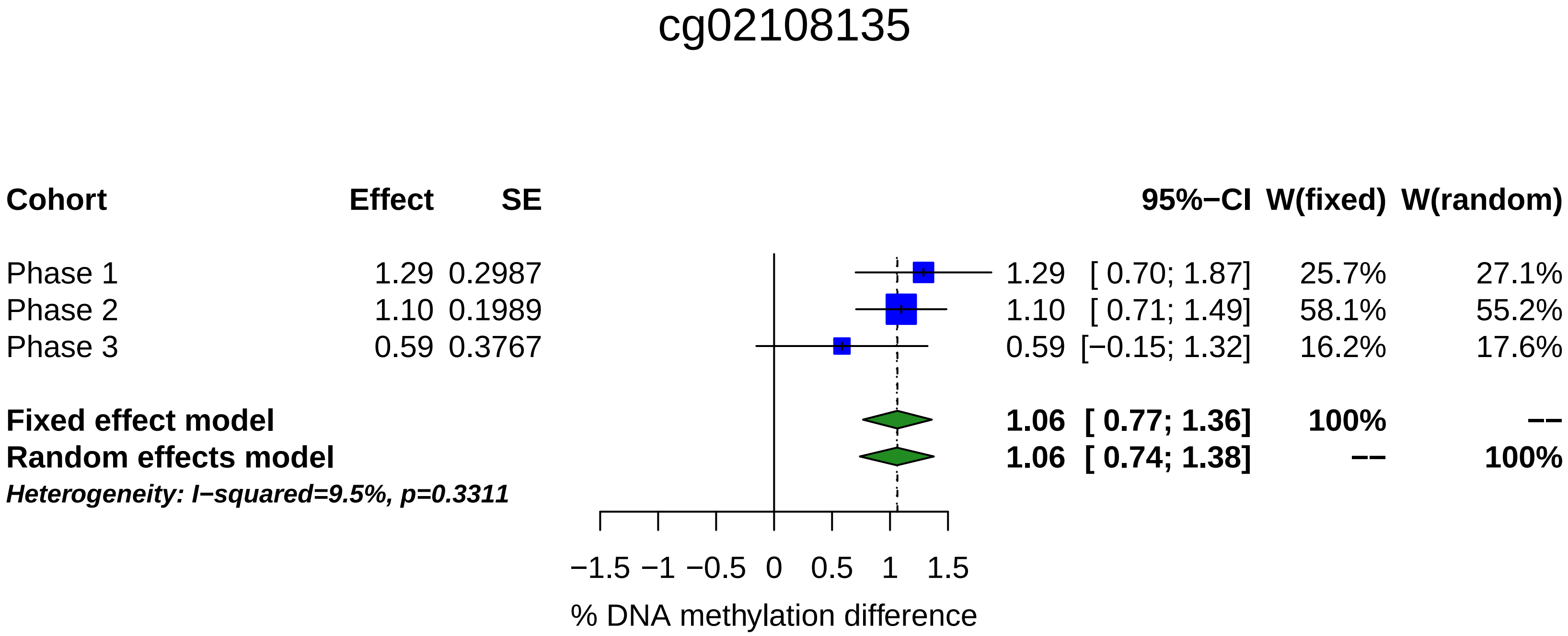
cg02108135
Cohort
Effect
SE
95%−CI
W(fixed)
W(random)
Phase 1 1.29 0.2987 1.29 [0.70; 1.87] 25.7% 27.1%
Phase 2 1.10 0.1989 1.10 [0.71; 1.49] 58.1% 55.2%
Phase 3 0.59 0.3767 0.59 [−0.15; 1.32] 16.2% 17.6%
Fixed effect model 1.06 [0.77; 1.36] 100% −−
Random effects model 1.06 [0.74; 1.38] −− 100%
Heterogeneity: I−squared=9.5%, p=0.3311
−1.5 −1 −0.5 0 0.5 1 1.5
% DNA methylation difference

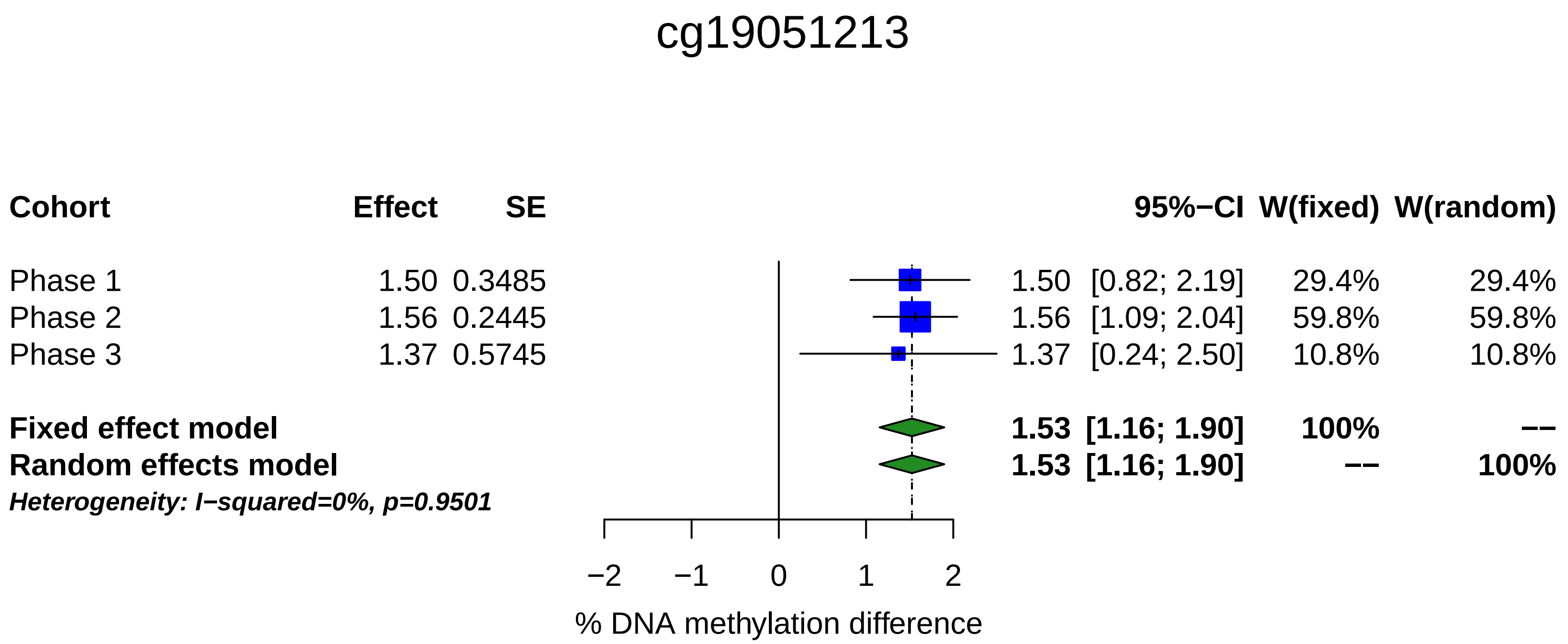
cg19051213
Cohort
Effect
SE
95%−CI
W(fixed)
W(random)
Phase 1 1.50 0.3485 1.50 [0.82; 2.19] 29.4% 29.4%
Phase 2 1.56 0.2445 1.56 [1.09; 2.04] 59.8% 59.8%
Phase 3 1.37 0.5745 1.37 [0.24; 2.50] 10.8% 10.8%
Fixed effect model 1.53 [1.16; 1.90] 100% −−
Random effects model 1.53 [1.16; 1.90] −− 100%
Heterogeneity: I−squared=0%, p=0.9501
−2
−1
0
1
2
% DNA methylation difference

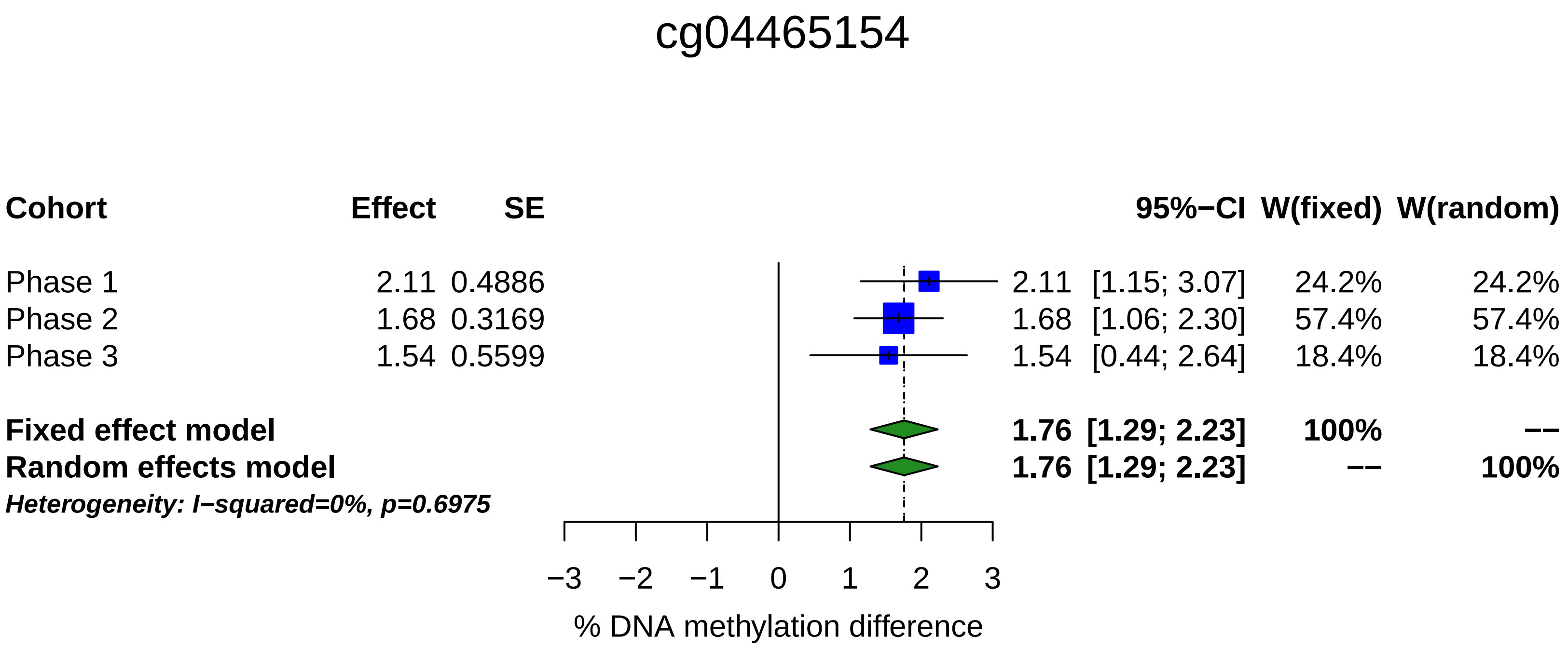
cg04465154
Cohort
Effect
SE
95%−CI
W(fixed)
W(random)
Phase 1 2.11 0.4886 2.11 [1.15; 3.07] 24.2% 24.2%
Phase 2 1.68 0.3169 1.68 [1.06; 2.30] 57.4% 57.4%
Phase 3 1.54 0.5599 1.54 [0.44; 2.64] 18.4% 18.4%
Fixed effect model 1.76 [1.29; 2.23] 100% −−
Random effects model 1.76 [1.29; 2.23] −− 100%
Heterogeneity: I−squared=0%, p=0.6975
−3 −2 −1 0 1 2 3
% DNA methylation difference

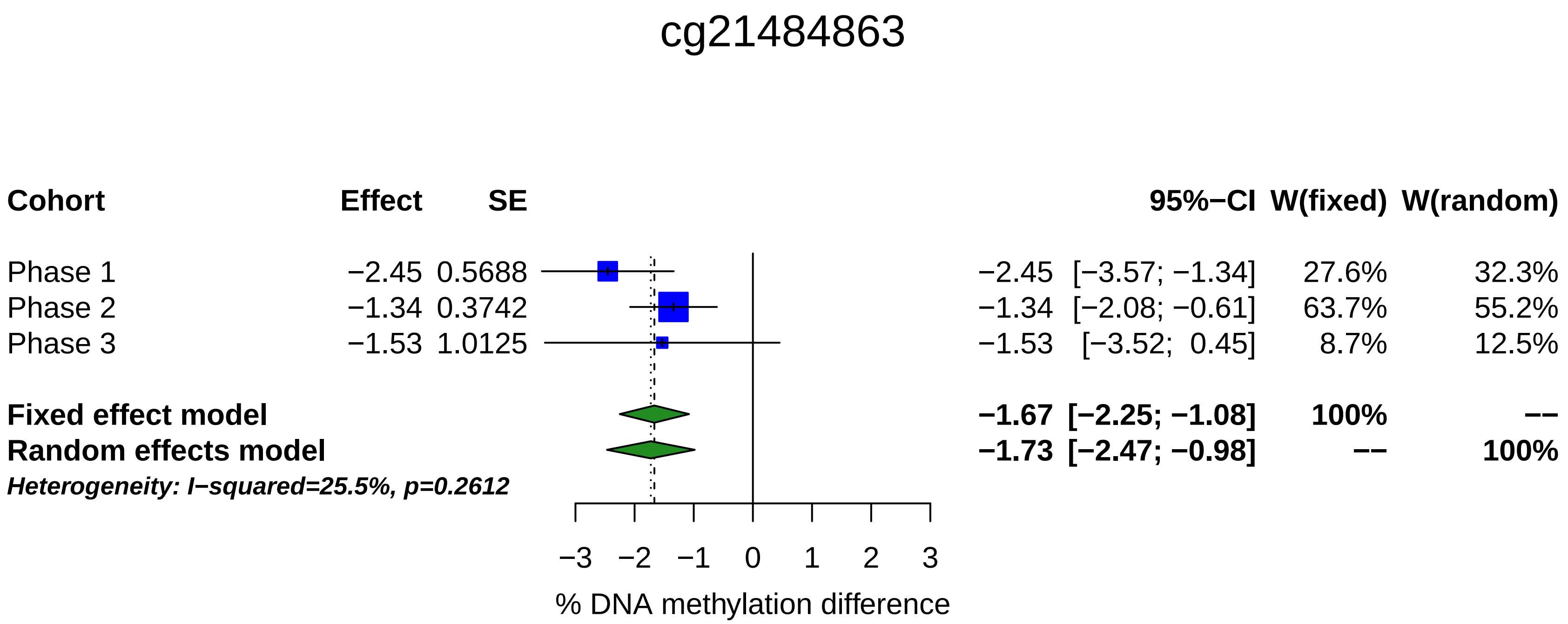
cg21484863
Cohort
Effect
SE
95%−CI
W(fixed)
W(random)
Phase 1 −2.45 0.5688 −2.45 [−3.57; −1.34] 27.6% 32.3%
Phase 2 −1.34 0.3742 −1.34 [−2.08; −0.61] 63.7% 55.2%
Phase 3 −1.53 1.0125 −1.53 [−3.52; 0.45] 8.7% 12.5%
Fixed effect model −1.67 [−2.25; −1.08] 100% −−
Random effects model −1.73 [−2.47; −0.98] −− 100%
Heterogeneity: I−squared=25.5%, p=0.2612
−3
−2
−1
0
1
2
3
% DNA methylation difference

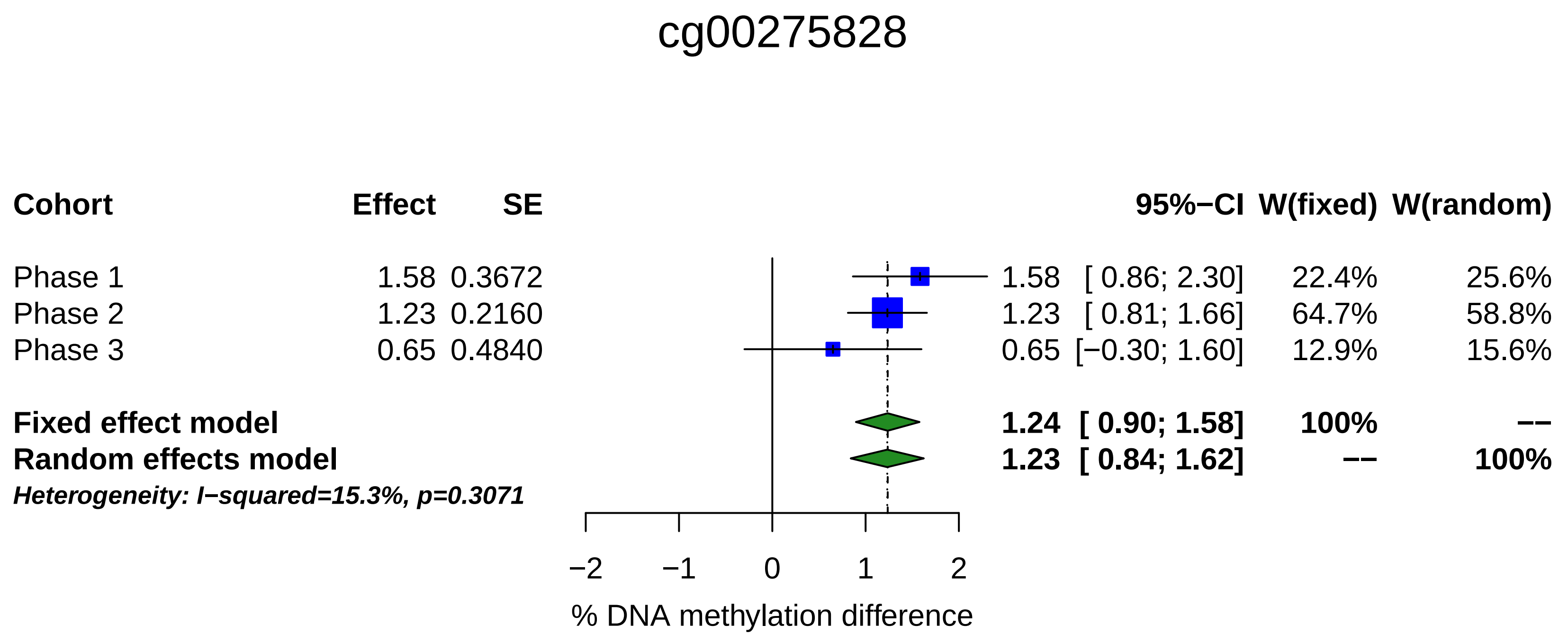
cg00275828
Cohort
Effect
SE
95%−CI
W(fixed)
W(random)
Phase 1 1.58 0.3672 1.58 [0.86; 2.30] 22.4% 25.6%
Phase 2 1.23 0.2160 1.23 [0.81; 1.66] 64.7% 58.8%
Phase 3 0.65 0.4840 0.65 [−0.30; 1.60] 12.9% 15.6%
Fixed effect model 1.24 [0.90; 1.58] 100% −−
Random effects model 1.23 [0.84; 1.62] −− 100%
Heterogeneity: I−squared=15.3%, p=0.3071
−2
−1
0
1
2
% DNA methylation difference

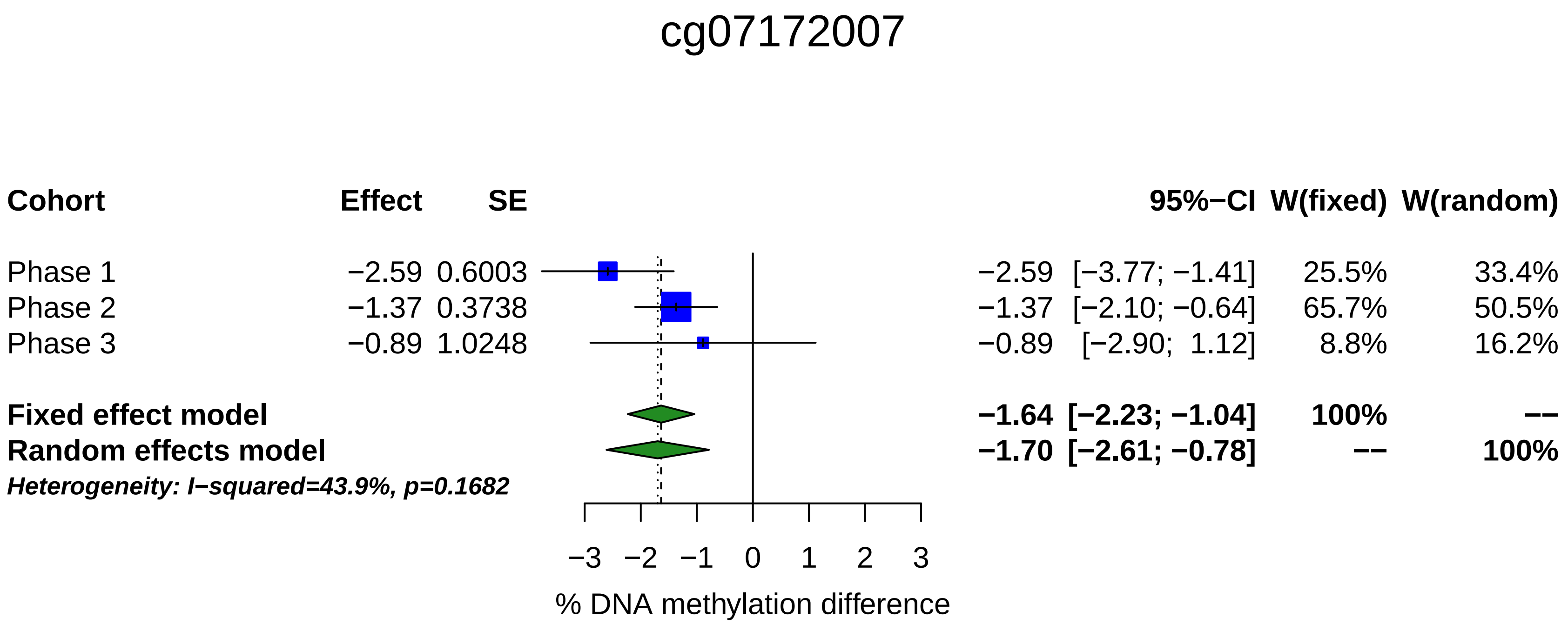
cg07172007
Cohort
Effect
SE
95%−CI
W(fixed)
W(random)
Phase 1 −2.59 0.6003 −2.59 [−3.77; −1.41] 25.5% 33.4%
Phase 2 −1.37 0.3738 −1.37 [−2.10; −0.64] 65.7% 50.5%
Phase 3 −0.89 1.0248 −0.89 [−2.90; 1.12] 8.8% 16.2%
Fixed effect model −1.64 [−2.23; −1.04] 100% −−
Random effects model −1.70 [−2.61; −0.78] −− 100%
Heterogeneity: I−squared=43.9%, p=0.1682
−3
−2
−1
0
1
2
3
% DNA methylation difference

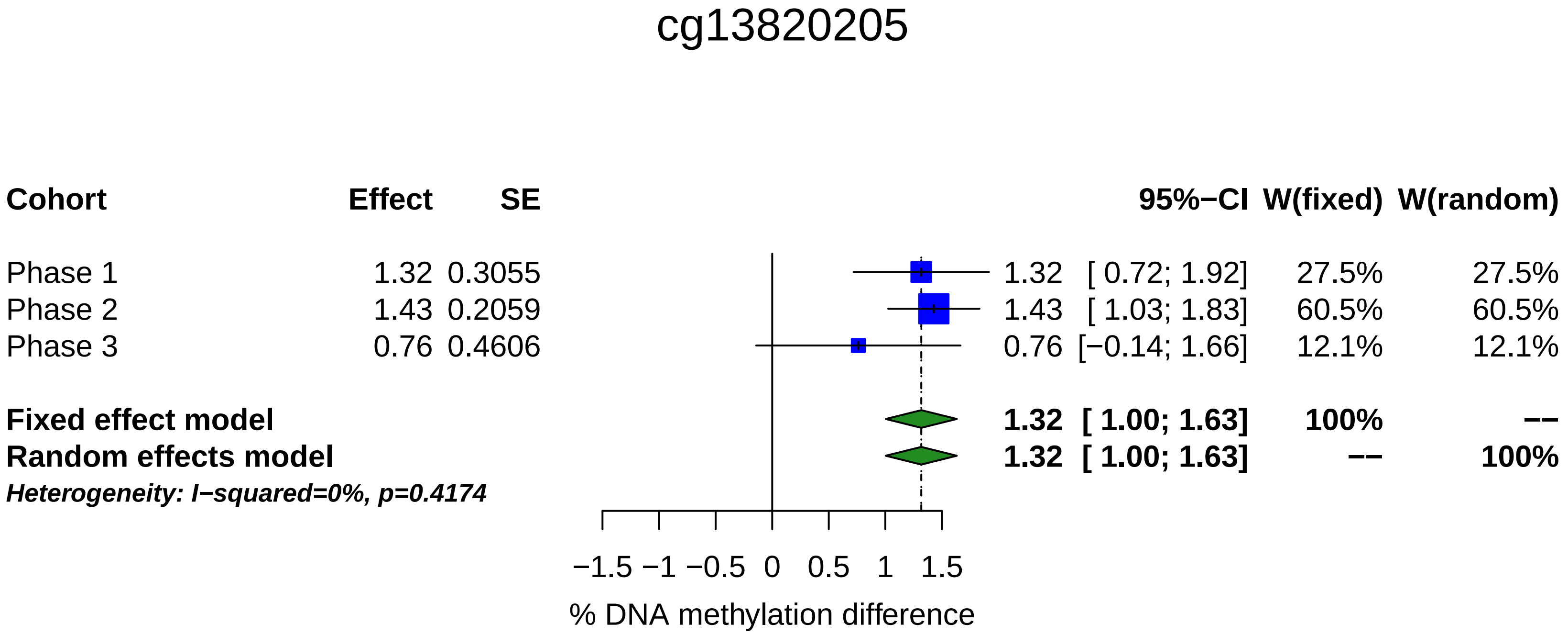
cg13820205
Cohort
Effect
SE
95%−CI
W(fixed)
W(random)
Phase 1 1.32 0.3055 1.32 [0.72; 1.92] 27.5% 27.5%
Phase 2 1.43 0.2059 1.43 [1.03; 1.83] 60.5% 60.5%
Phase 3 0.76 0.4606 0.76 [−0.14; 1.66] 12.1% 12.1%
Fixed effect model 1.32 [1.00; 1.63] 100% −−
Random effects model 1.32 [1.00; 1.63] −− 100%
Heterogeneity: I−squared=0%, p=0.4174
−1.5
−1
−0.5
0
0.5
1
1.5
% DNA methylation difference

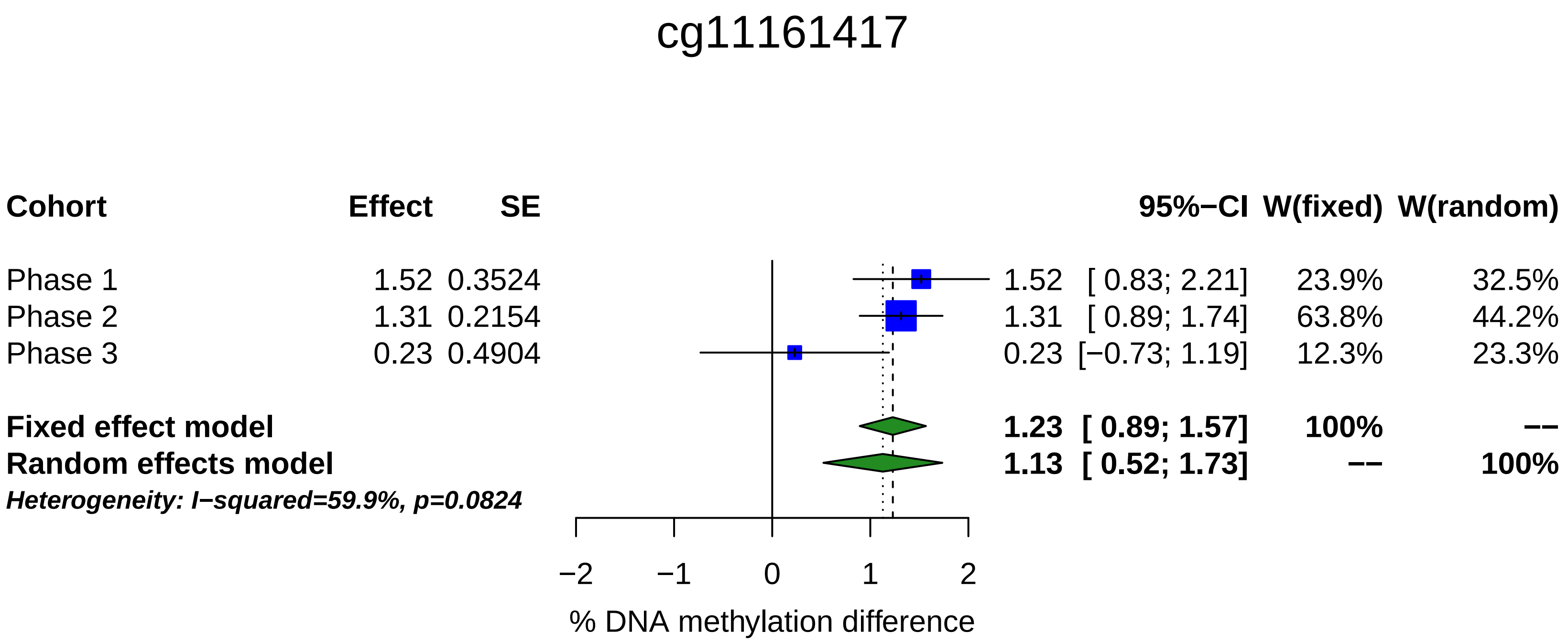
cg11161417
Cohort
Effect
SE
95%−CI
W(fixed)
W(random)
Phase 1 1.52 0.3524
Phase 2 1.31 0.2154
Phase 3 0.23 0.4904
1.52 [0.83; 2.21] 23.9% 32.5%
1.31 [0.89; 1.74] 63.8% 44.2%
0.23 [−0.73; 1.19] 12.3% 23.3%
Fixed effect model
Random effects model
1.23 [0.89; 1.57] 100% −−
1.13 [0.52; 1.73] −− 100%
Heterogeneity: I−squared=59.9%, p=0.0824
−2
−1
0
1
2
% DNA methylation difference

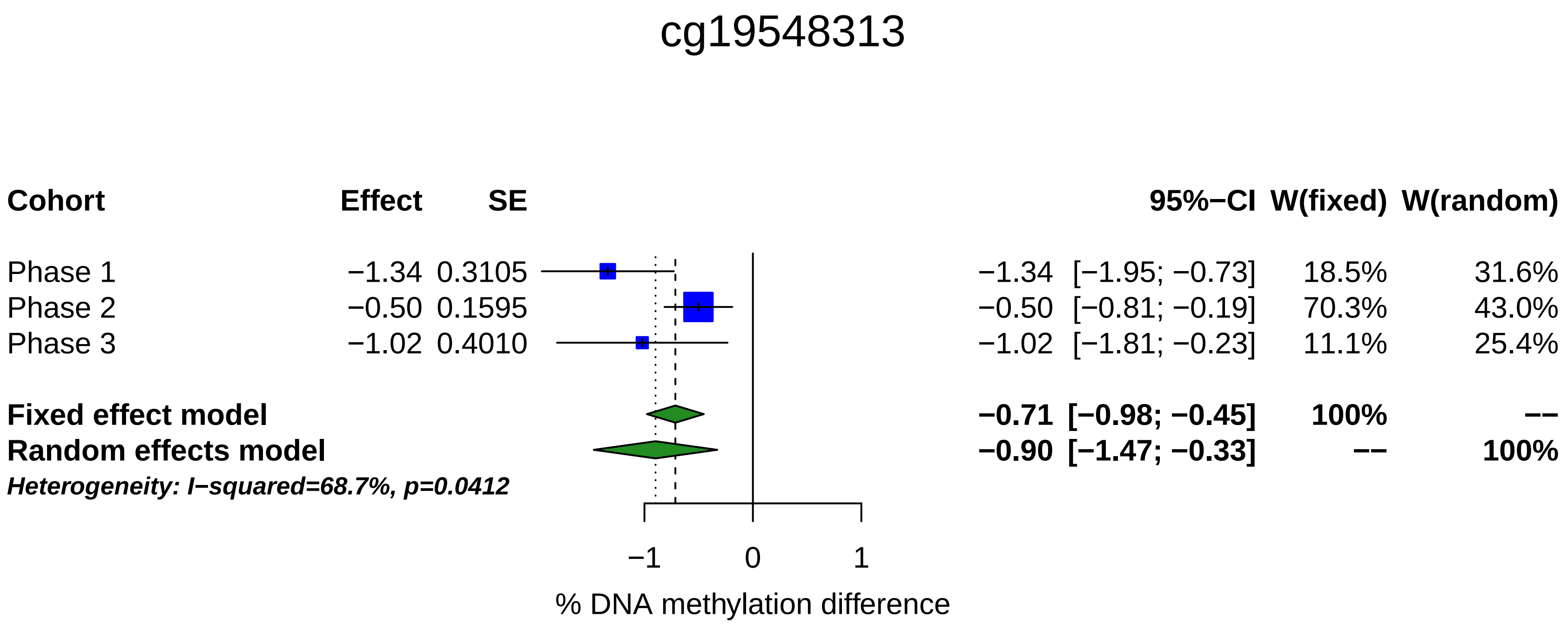
cg19548313
Cohort
Effect
SE
95%−CI
W(fixed)
W(random)
Phase 1 −1.34 0.3105 −1.34 [−1.95; −0.73] 18.5% 31.6%
Phase 2 −0.50 0.1595 −0.50 [−0.81; −0.19] 70.3% 43.0%
Phase 3 −1.02 0.4010 −1.02 [−1.81; −0.23] 11.1% 25.4%
Fixed effect model −0.71 [−0.98; −0.45] 100% −−
Random effects model −0.90 [−1.47; −0.33] −− 100%
Heterogeneity: I−squared=68.7%, p=0.0412
−1
0
1
% DNA methylation difference

cg07835482

Cohort	Effect	SE	95%−CI	W(fixed)	W(random)
Phase 1	1.94	0.4500	1.94 [1.06; 2.82]	26.3%	26.3%
Phase 2	1.22	0.3017	1.22 [0.63; 1.81]	58.6%	58.6%
Phase 3	1.43	0.5955	1.43 [0.26; 2.59]	15.0%	15.0%
**Fixed effect model**			**1.44 [0.99; 1.89]**	**100%**	**−−**
**Random effects model**			**1.44 [0.99; 1.89]**	**−−**	**100%**

Heterogeneity: I−squared=0%, p=0.4173

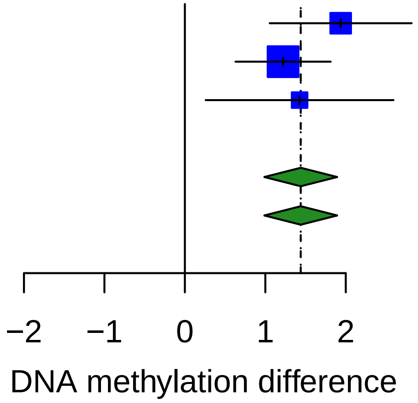

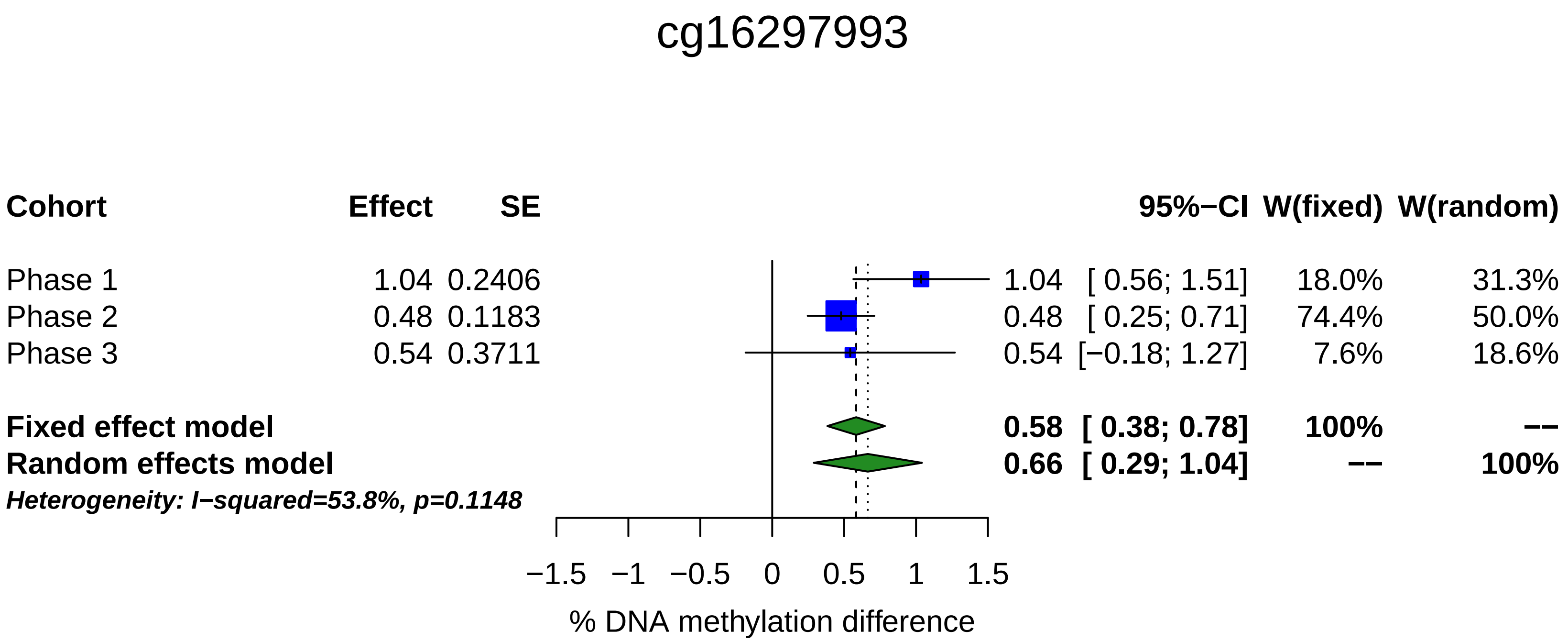
cg16297993
Cohort
Effect
SE
95%−CI
W(fixed)
W(random)
Phase 1
1.04
0.2406
1.04 [0.56; 1.51]
18.0%
31.3%
Phase 2
0.48
0.1183
0.48 [0.25; 0.71]
74.4%
50.0%
Phase 3
0.54
0.3711
0.54 [−0.18; 1.27]
7.6%
18.6%
Fixed effect model
0.58 [0.38; 0.78]
100%
−−
Random effects model
0.66 [0.29; 1.04]
−−
100%
Heterogeneity: I−squared=53.8%, p=0.1148
−1.5
−1
−0.5
0
0.5
1
1.5
% DNA methylation difference

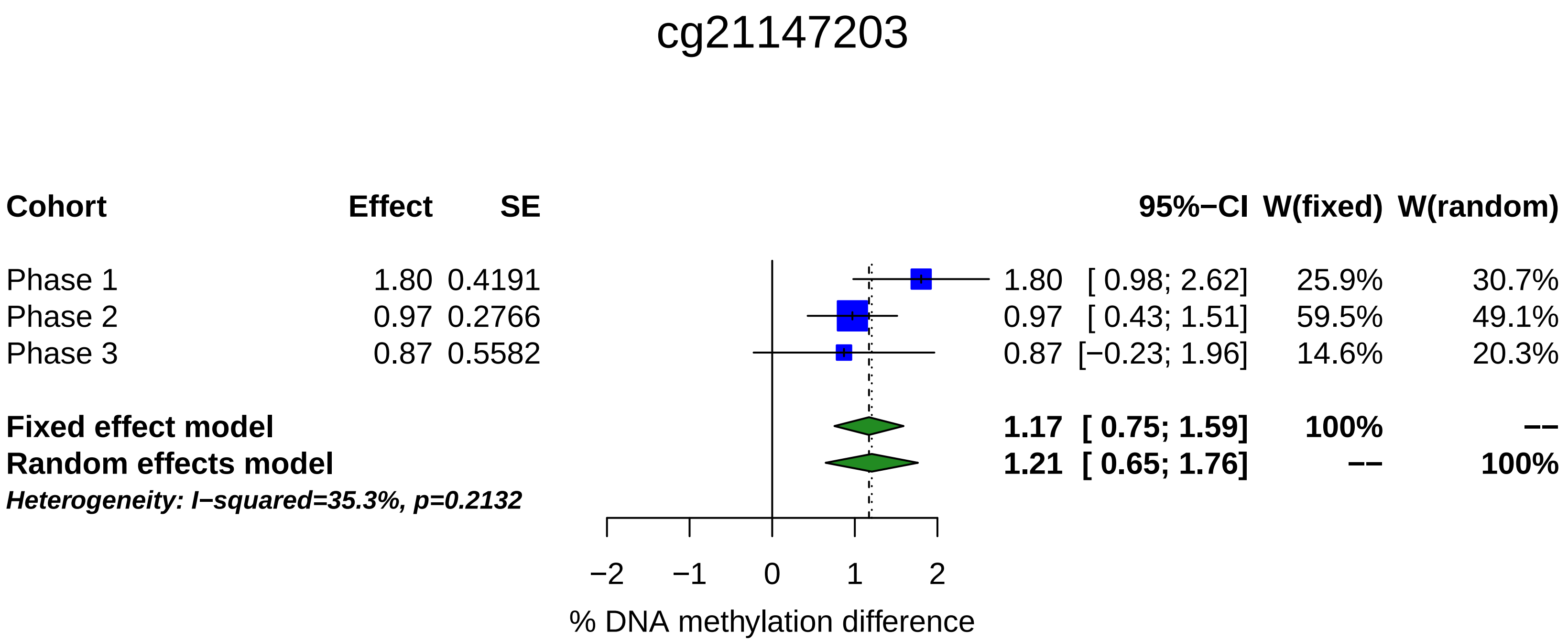
cg21147203
Cohort
Effect
SE
95%−CI
W(fixed)
W(random)
Phase 1 1.80 0.4191 1.80 [0.98; 2.62] 25.9% 30.7%
Phase 2 0.97 0.2766 0.97 [0.43; 1.51] 59.5% 49.1%
Phase 3 0.87 0.5582 0.87 [−0.23; 1.96] 14.6% 20.3%
Fixed effect model 1.17 [0.75; 1.59] 100% −−
Random effects model 1.21 [0.65; 1.76] −− 100%
Heterogeneity: I−squared=35.3%, p=0.2132
−2
−1
0
1
2
% DNA methylation difference

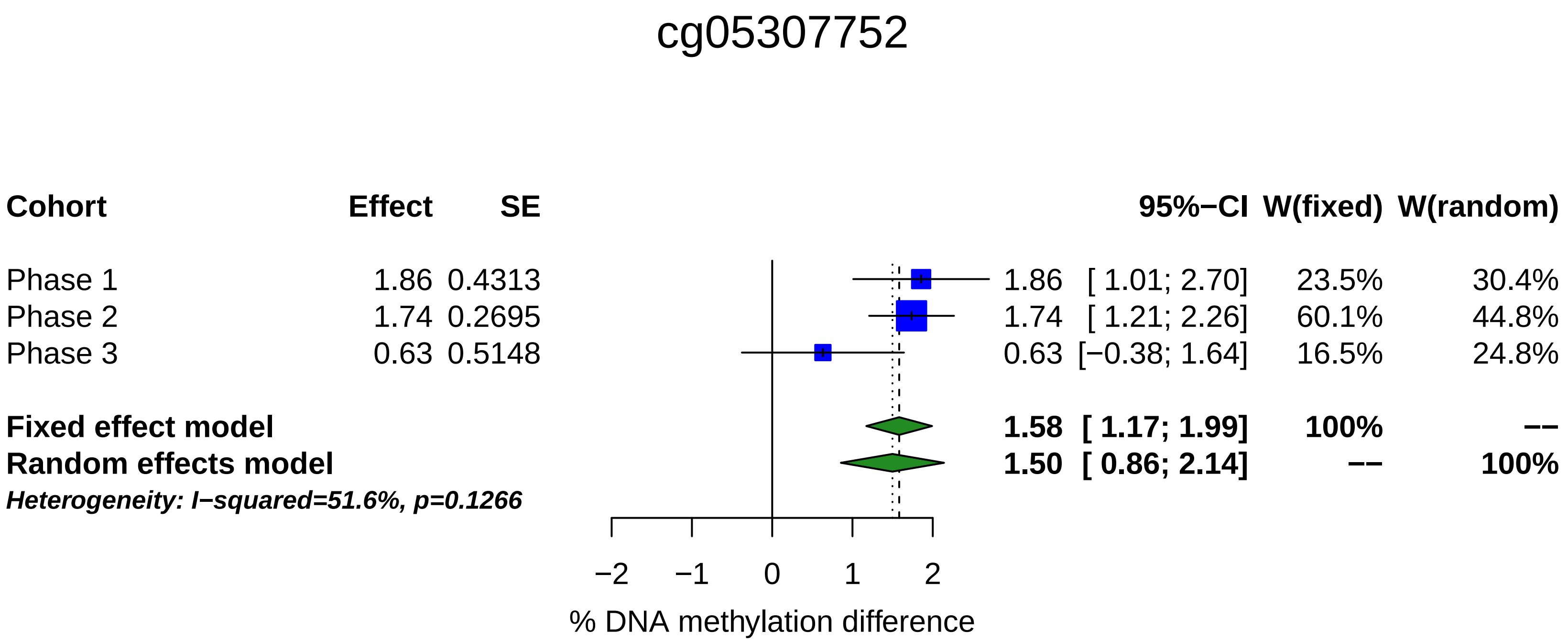
cg05307752
Cohort
Effect
SE
95%−CI
W(fixed)
W(random)
Phase 1 1.86 0.4313 1.86 [1.01; 2.70] 23.5% 30.4%
Phase 2 1.74 0.2695 1.74 [1.21; 2.26] 60.1% 44.8%
Phase 3 0.63 0.5148 0.63 [−0.38; 1.64] 16.5% 24.8%
Fixed effect model 1.58 [1.17; 1.99] 100% −−
Random effects model 1.50 [0.86; 2.14] −− 100%
Heterogeneity: I−squared=51.6%, p=0.1266
−2
−1
0
1
2
% DNA methylation difference

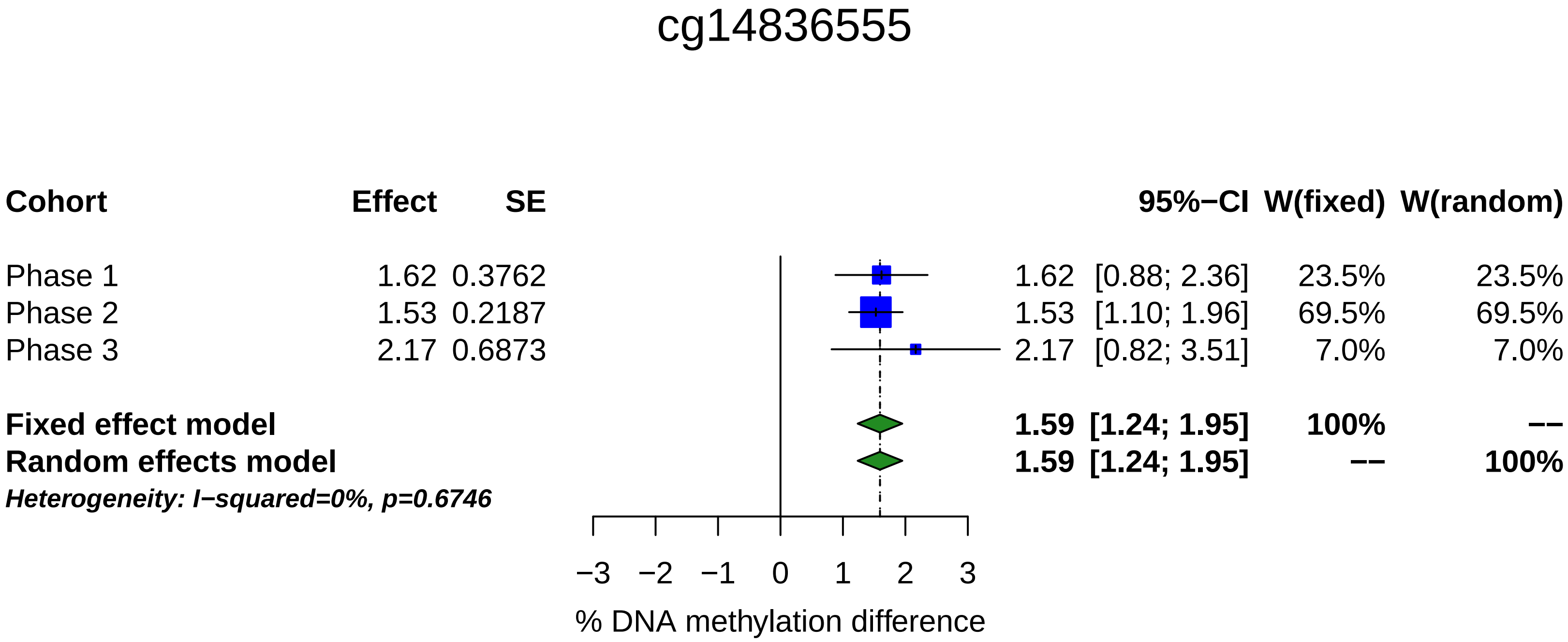

cg14836555
Cohort
Effect
SE
95%−CI
W(fixed)
W(random)
Phase 1 1.62 0.3762 1.62 [0.88; 2.36] 23.5% 23.5%
Phase 2 1.53 0.2187 1.53 [1.10; 1.96] 69.5% 69.5%
Phase 3 2.17 0.6873 2.17 [0.82; 3.51] 7.0% 7.0%
Fixed effect model 1.59 [1.24; 1.95] 100% −−
Random effects model 1.59 [1.24; 1.95] −− 100%
Heterogeneity: I−squared=0%, p=0.6746
−3
−2
−1
0
1
2
3
% DNA methylation difference

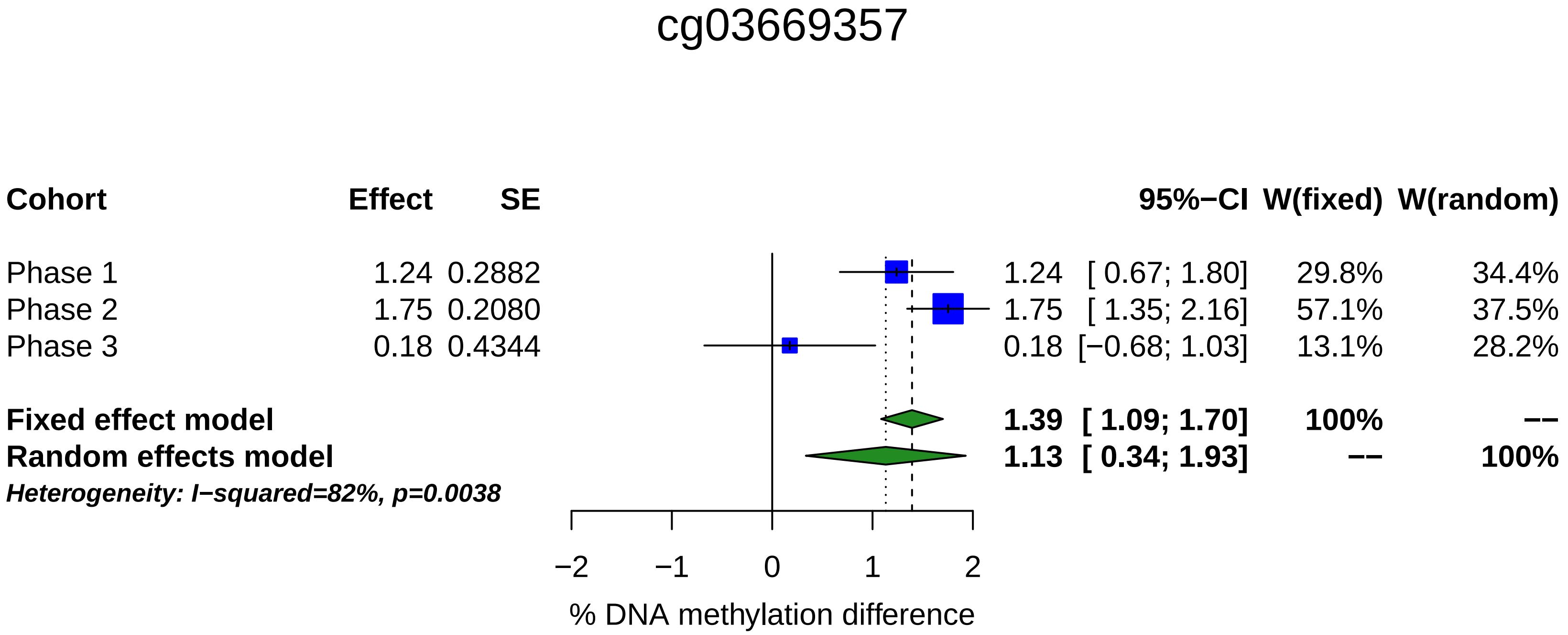
cg03669357
Cohort
Effect
SE
95%−CI
W(fixed)
W(random)
Phase 1
1.24
0.2882
1.24 [0.67; 1.80]
29.8%
34.4%
Phase 2
1.75
0.2080
1.75 [1.35; 2.16]
57.1%
37.5%
Phase 3
0.18
0.4344
0.18 [−0.68; 1.03]
13.1%
28.2%
Fixed effect model
1.39 [1.09; 1.70]
100%
−−
Random effects model
1.13 [0.34; 1.93]
−−
100%
Heterogeneity: I−squared=82%, p=0.0038
−2
−1
0
1
2
% DNA methylation difference

cg19430423

Cohort	Effect	SE		95%−CI	W(fixed)	W(random)
Phase 1	1.03	0.2400	1.03	[0.56; 1.50]	29.4%	32.0%
Phase 2	0.62	0.1643	0.62	[0.30; 0.94]	62.7%	58.4%
Phase 3	1.03	0.4638	1.03	[0.12; 1.94]	7.9%	9.6%
**Fixed effect model**			**0.78**	**[0.52; 1.03]**	**100%**	**−−**
**Random effects model**			**0.79**	**[0.50; 1.08]**	**−−**	**100%**

Heterogeneity: I−squared=13.2%, p=0.3158

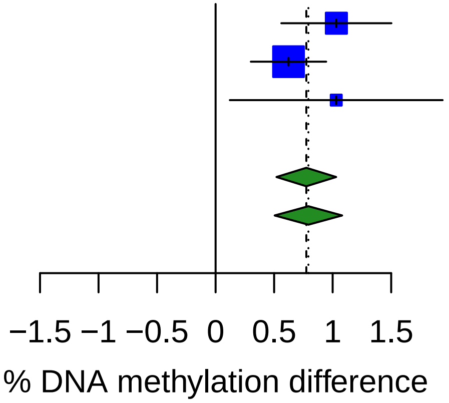

cg24152264

Cohort	Effect	SE	95%−CI	W(fixed)	W(random)
Phase 1	1.12	0.2606	1.12 [0.61; 1.63]	28.7%	33.8%
Phase 2	1.38	0.1852	1.38 [1.02; 1.75]	56.8%	36.2%
Phase 3	−0.22	0.3662	−0.22 [−0.93; 0.50]	14.5%	30.0%
**Fixed effect model**			**1.08 [0.80; 1.35]**	**100%**	**−−**
**Random effects model**			**0.81 [−0.01; 1.63]**	**−−**	**100%**

Heterogeneity: I−squared=86.9%, p=0.0005

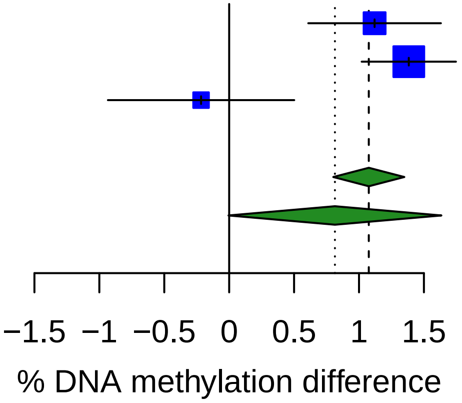

cg14583550

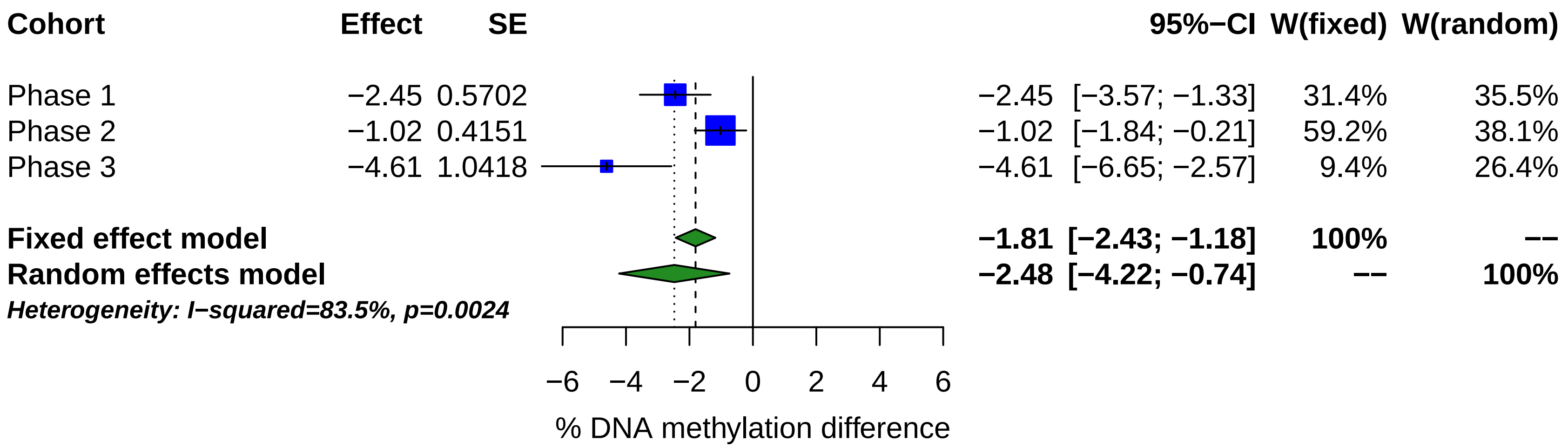

Cohort
Effect
SE
95%−CI
W(fixed)
W(random)
Phase 1 −2.45 0.5702 −2.45 [−3.57; −1.33] 31.4% 35.5%
Phase 2 −1.02 0.4151 −1.02 [−1.84; −0.21] 59.2% 38.1%
Phase 3 −4.61 1.0418 −4.61 [−6.65; −2.57] 9.4% 26.4%
Fixed effect model −1.81 [−2.43; −1.18] 100% −−
Random effects model −2.48 [−4.22; −0.74] −− 100%
Heterogeneity: I−squared=83.5%, p=0.0024
−6 −4 −2 0 2 4 6
% DNA methylation difference

cg23155293

Cohort	Effect	SE	95%−CI	W(fixed)	W(random)
Phase 1	−2.06	0.4812	−2.06 [−3.01; −1.12]	29.8%	29.8%
Phase 2	−1.28	0.3328	−1.28 [−1.93; −0.63]	62.2%	62.2%
Phase 3	−1.69	0.9269	−1.69 [−3.51; 0.13]	8.0%	8.0%
**Fixed effect model**			**−1.54 [−2.06; −1.03]**	**100%**	**−−**
**Random effects model**			**−1.54 [−2.06; −1.03]**	**−−**	**100%**

Heterogeneity: I−squared=0%, p=0.3992

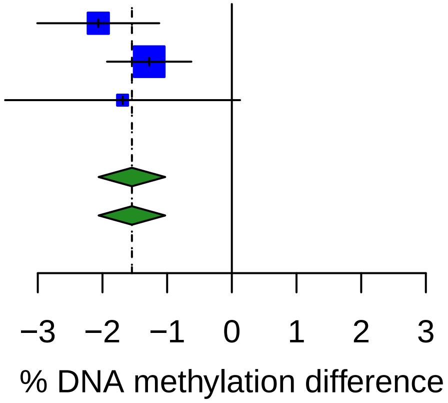

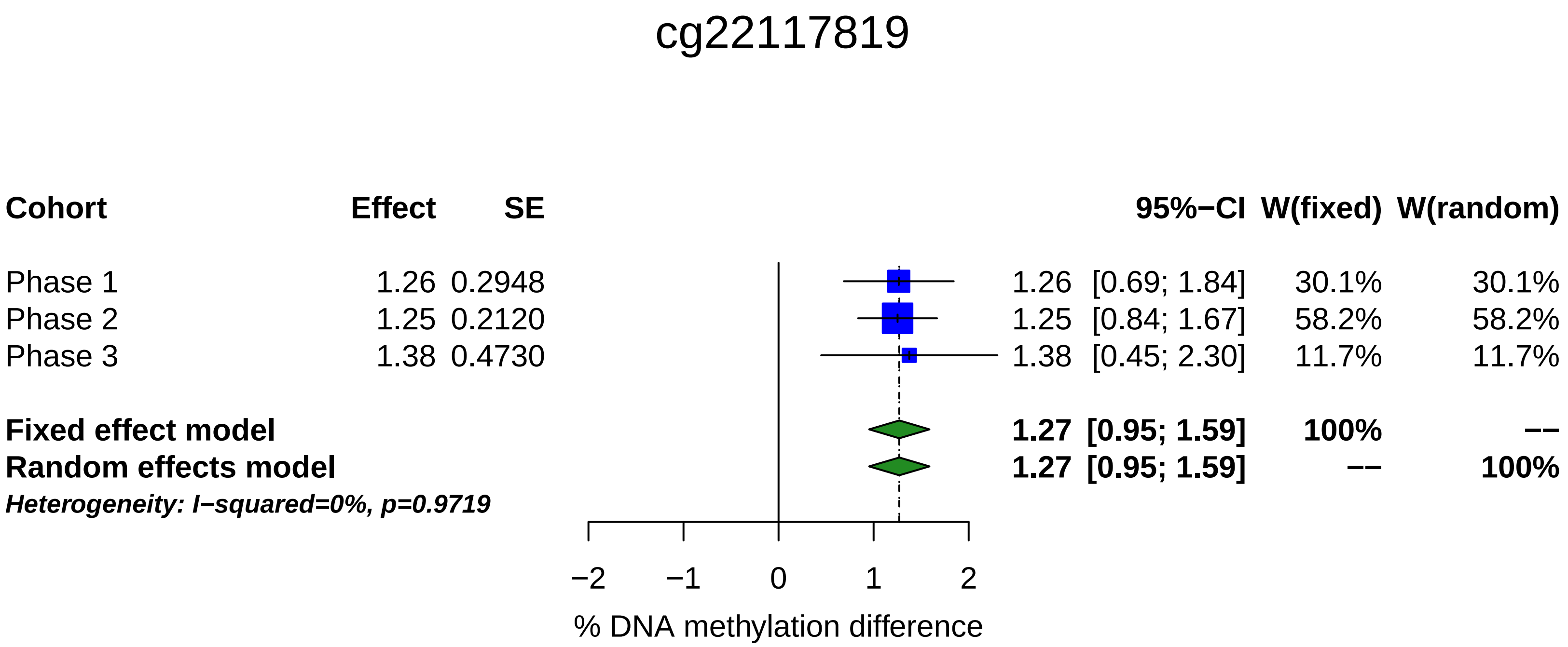
cg22117819
Cohort
Effect
SE
95%−CI
W(fixed)
W(random)
Phase 1
1.26
0.2948
1.26 [0.69; 1.84]
30.1%
30.1%
Phase 2
1.25
0.2120
1.25 [0.84; 1.67]
58.2%
58.2%
Phase 3
1.38
0.4730
1.38 [0.45; 2.30]
11.7%
11.7%
Fixed effect model
1.27 [0.95; 1.59]
100%
−−
Random effects model
1.27 [0.95; 1.59]
−−
100%
Heterogeneity: I−squared=0%, p=0.9719
−2
−1
0
1
2
% DNA methylation difference

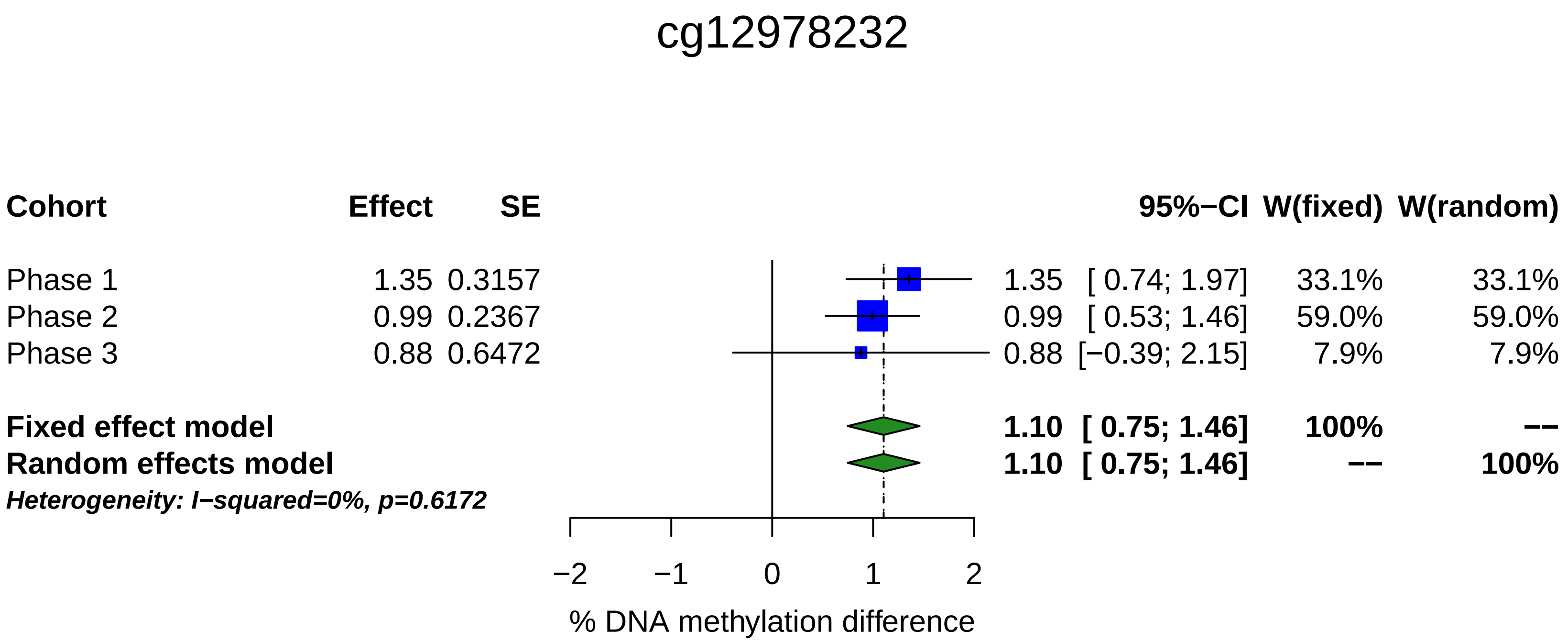
cg12978232
Cohort
Effect
SE
95%−CI
W(fixed)
W(random)
Phase 1 1.35 0.3157 1.35 [0.74; 1.97] 33.1% 33.1%
Phase 2 0.99 0.2367 0.99 [0.53; 1.46] 59.0% 59.0%
Phase 3 0.88 0.6472 0.88 [−0.39; 2.15] 7.9% 7.9%
Fixed effect model 1.10 [0.75; 1.46] 100% −−
Random effects model 1.10 [0.75; 1.46] −− 100%
Heterogeneity: I−squared=0%, p=0.6172
−2
−1
0
1
2
% DNA methylation difference

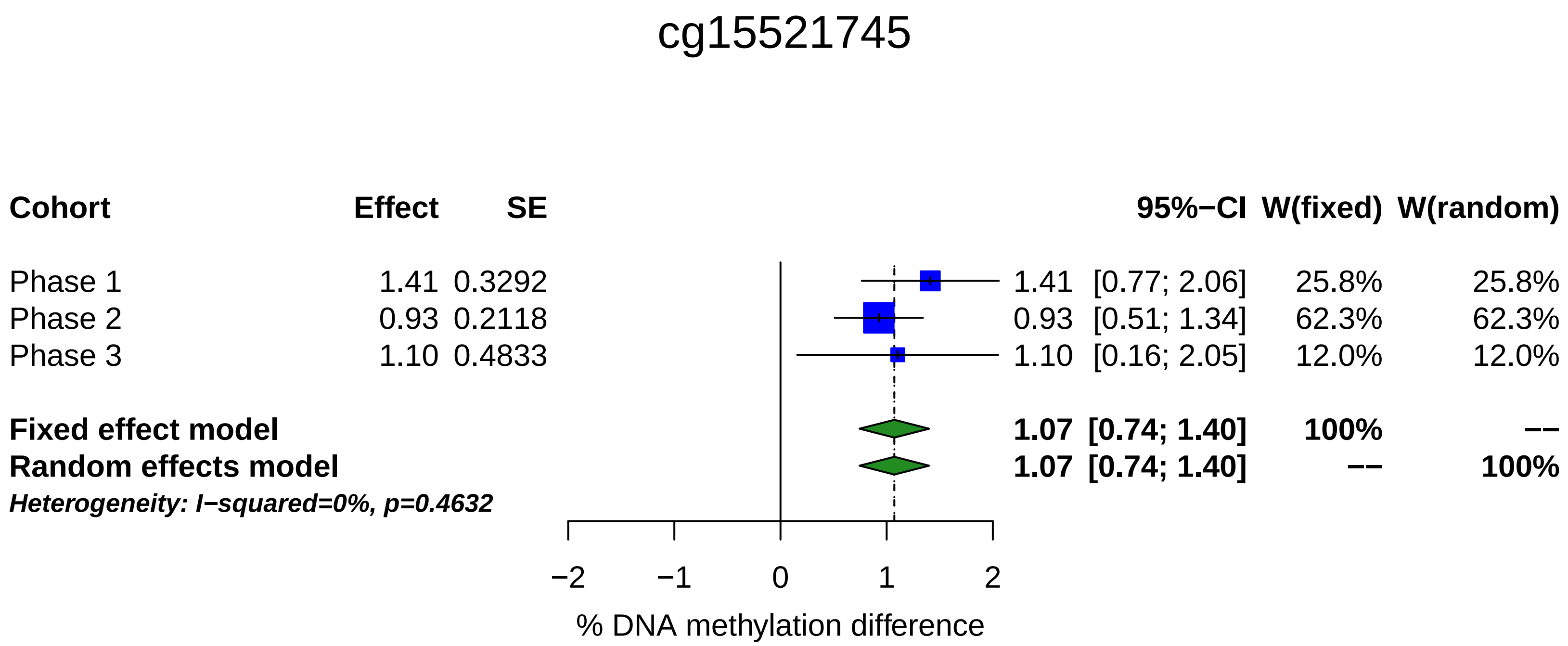
cg15521745
Cohort
Effect
SE
95%−CI
W(fixed)
W(random)
Phase 1 1.41 0.3292 1.41 [0.77; 2.06] 25.8% 25.8%
Phase 2 0.93 0.2118 0.93 [0.51; 1.34] 62.3% 62.3%
Phase 3 1.10 0.4833 1.10 [0.16; 2.05] 12.0% 12.0%
Fixed effect model 1.07 [0.74; 1.40] 100% −−
Random effects model 1.07 [0.74; 1.40] −− 100%
Heterogeneity: I−squared=0%, p=0.4632
−2
−1
0
1
2
% DNA methylation difference

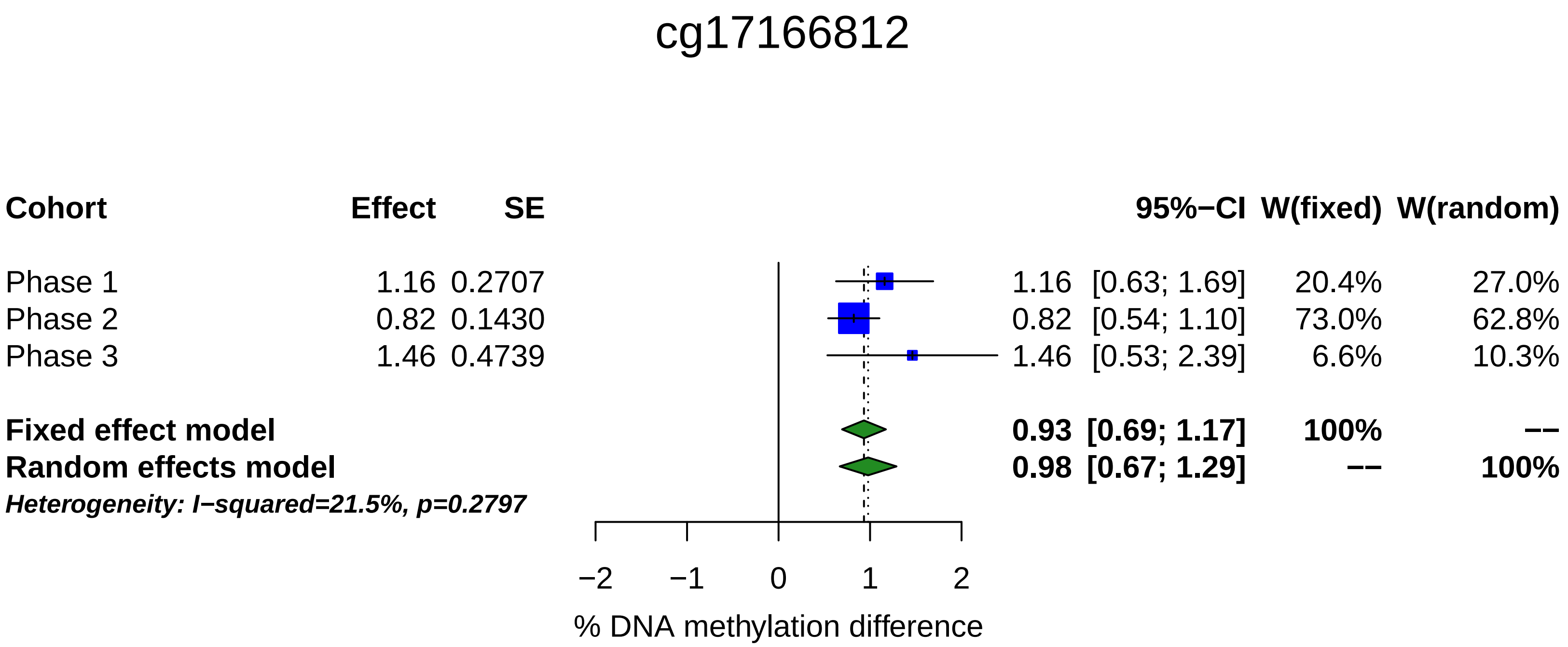
cg17166812
Cohort
Effect
SE
95%−CI
W(fixed)
W(random)
Phase 1
1.16
0.2707
1.16 [0.63; 1.69]
20.4%
27.0%
Phase 2
0.82
0.1430
0.82 [0.54; 1.10]
73.0%
62.8%
Phase 3
1.46
0.4739
1.46 [0.53; 2.39]
6.6%
10.3%
Fixed effect model
0.93 [0.69; 1.17]
100%
−−
Random effects model
0.98 [0.67; 1.29]
−−
100%
Heterogeneity: I−squared=21.5%, p=0.2797
−2
−1
0
1
2
% DNA methylation difference

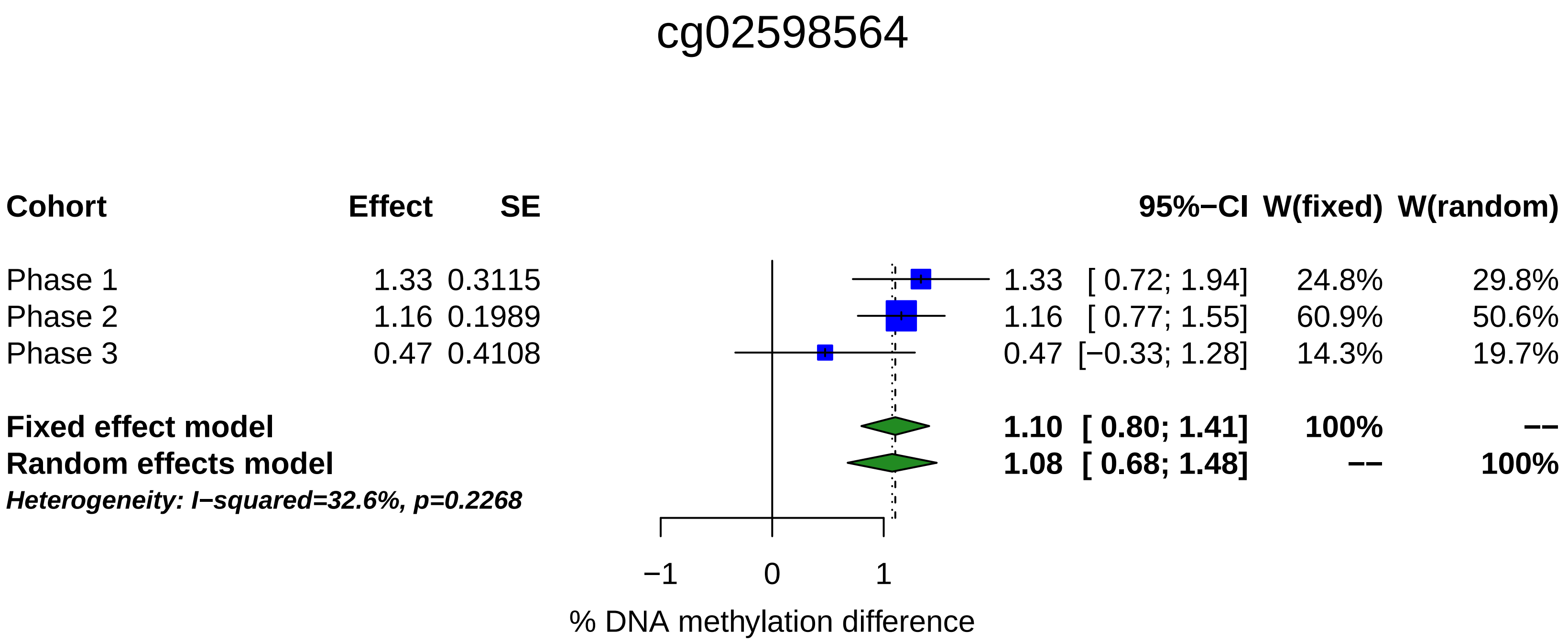
cg02598564
Cohort
Effect
SE
95%−CI
W(fixed)
W(random)
Phase 1 1.33 0.3115 1.33 [0.72; 1.94] 24.8% 29.8%
Phase 2 1.16 0.1989 1.16 [0.77; 1.55] 60.9% 50.6%
Phase 3 0.47 0.4108 0.47 [−0.33; 1.28] 14.3% 19.7%
Fixed effect model 1.10 [0.80; 1.41] 100% −−
Random effects model 1.08 [0.68; 1.48] −− 100%
Heterogeneity: I−squared=32.6%, p=0.2268
−1
0
1
% DNA methylation difference

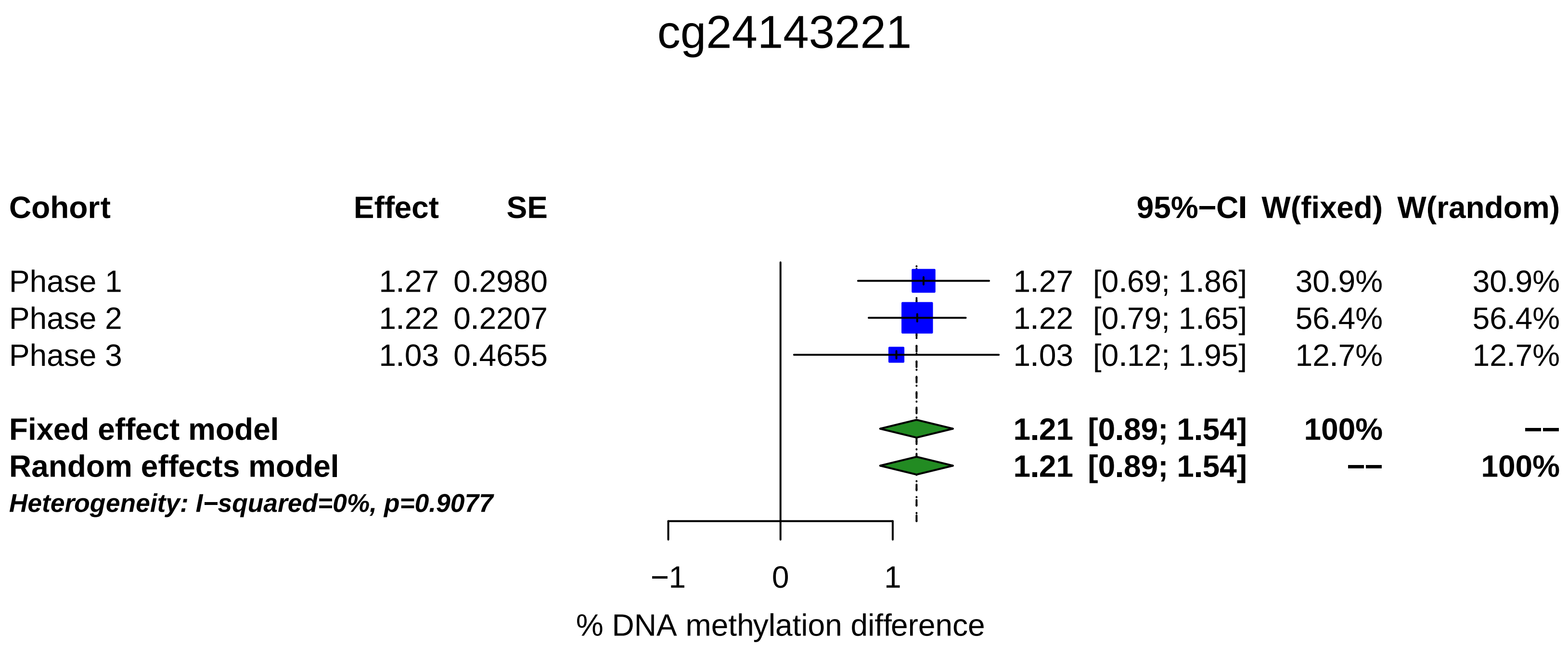
cg24143221
Cohort
Effect
SE
95%−CI
W(fixed)
W(random)
Phase 1 1.27 0.2980 1.27 [0.69; 1.86] 30.9% 30.9%
Phase 2 1.22 0.2207 1.22 [0.79; 1.65] 56.4% 56.4%
Phase 3 1.03 0.4655 1.03 [0.12; 1.95] 12.7% 12.7%
Fixed effect model 1.21 [0.89; 1.54] 100% −−
Random effects model 1.21 [0.89; 1.54] −− 100%
Heterogeneity: I−squared=0%, p=0.9077
−1
0
1
% DNA methylation difference

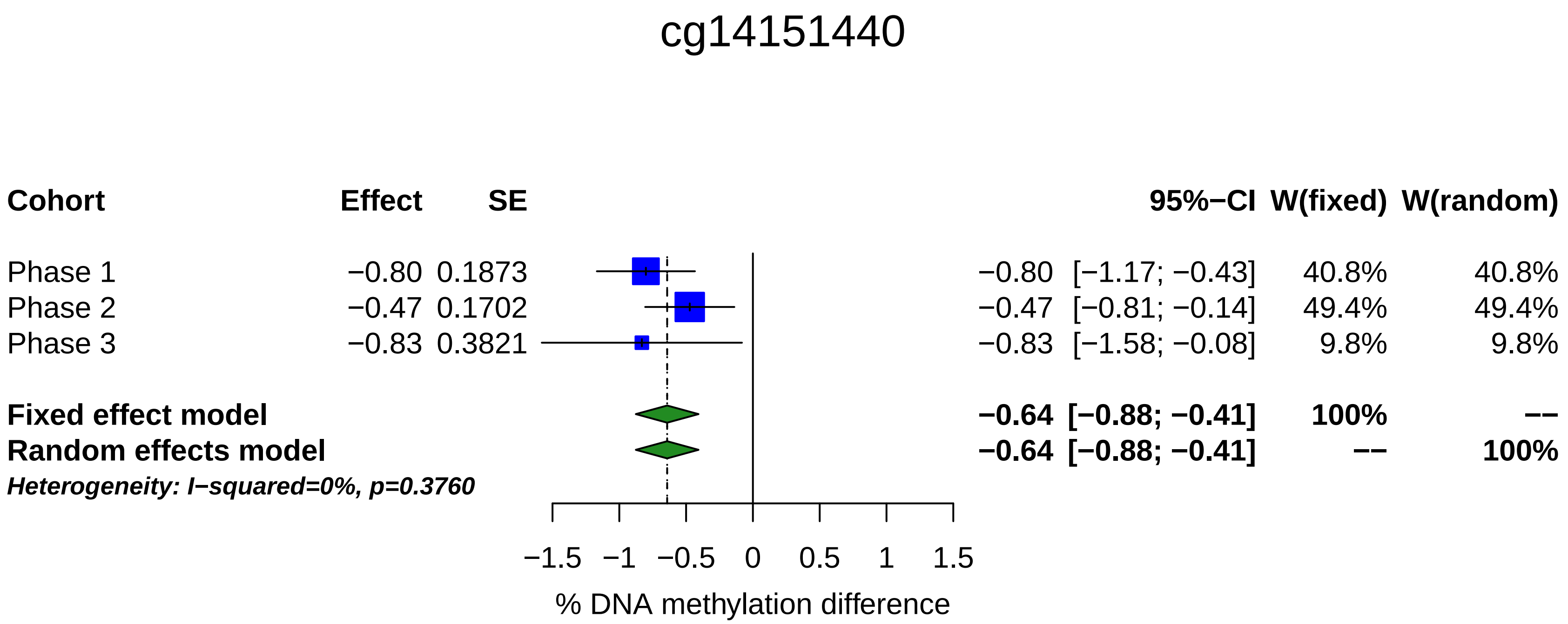
cg14151440
Cohort
Effect
SE
95%−CI
W(fixed)
W(random)
Phase 1 −0.80 0.1873 −0.80 [−1.17; −0.43] 40.8% 40.8%
Phase 2 −0.47 0.1702 −0.47 [−0.81; −0.14] 49.4% 49.4%
Phase 3 −0.83 0.3821 −0.83 [−1.58; −0.08] 9.8% 9.8%
Fixed effect model −0.64 [−0.88; −0.41] 100% −−
Random effects model −0.64 [−0.88; −0.41] −− 100%
Heterogeneity: I−squared=0%, p=0.3760
−1.5
−1
−0.5
0
0.5
1
1.5
% DNA methylation difference

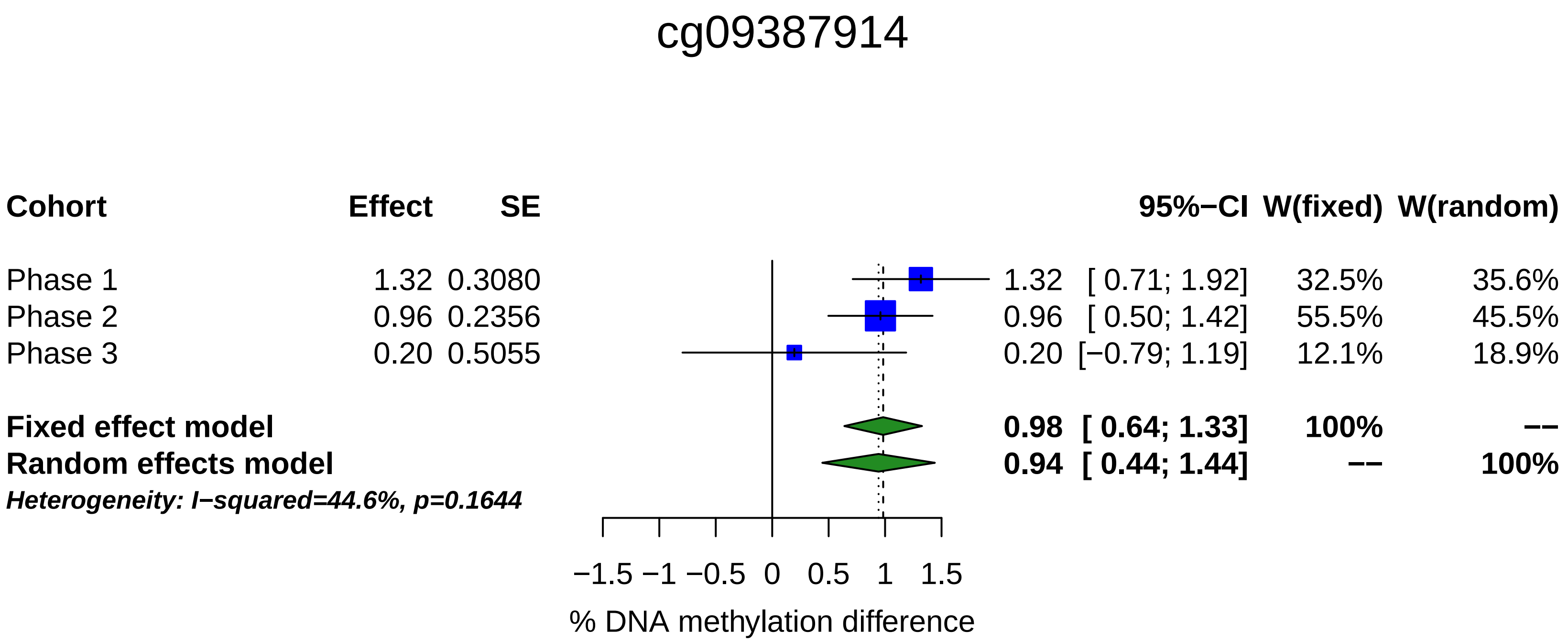
cg09387914
Cohort
Effect
SE
95%−CI
W(fixed)
W(random)
Phase 1
1.32
0.3080
1.32
[0.71; 1.92]
32.5%
35.6%
Phase 2
0.96
0.2356
0.96
[0.50; 1.42]
55.5%
45.5%
Phase 3
0.20
0.5055
0.20
[−0.79; 1.19]
12.1%
18.9%
Fixed effect model
0.98
[0.64; 1.33]
100%
−−
Random effects model
0.94
[0.44; 1.44]
−−
100%
Heterogeneity: I−squared=44.6%, p=0.1644
−1.5
−1
−0.5
0
0.5
1
1.5
% DNA methylation difference

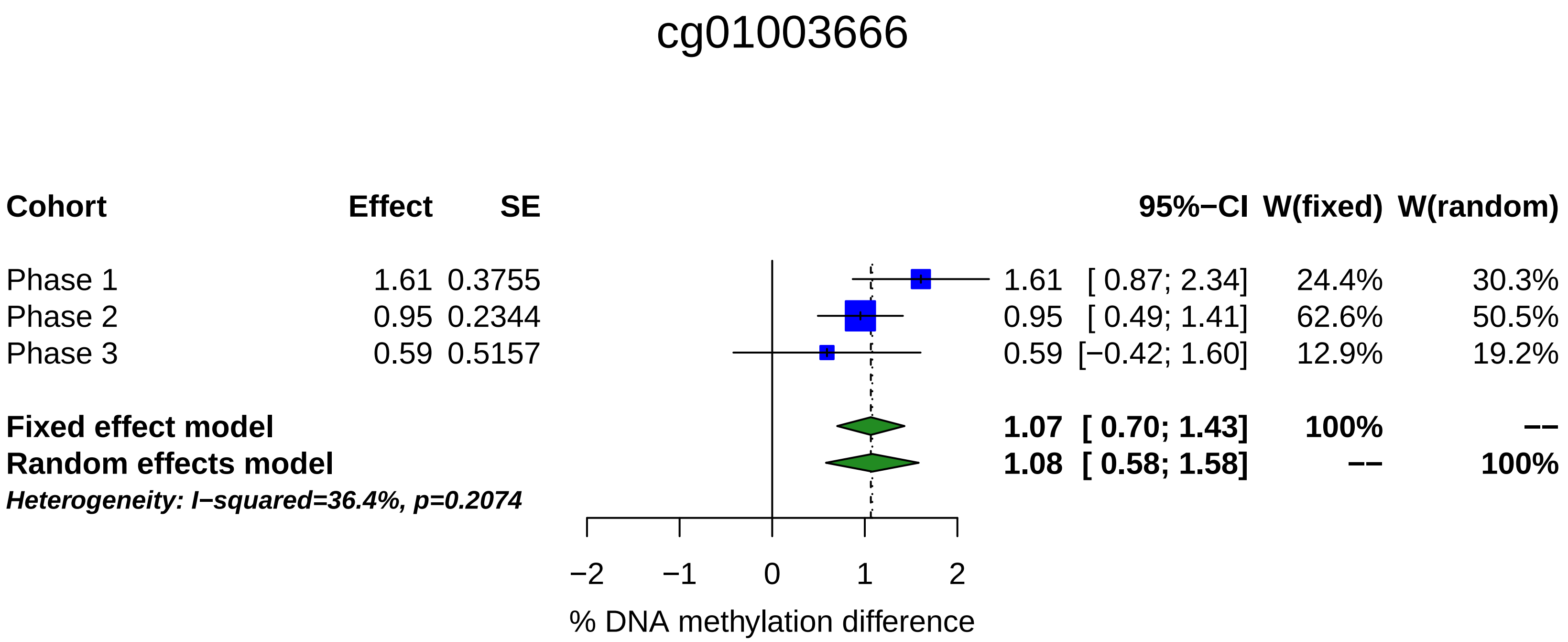
cg01003666
Cohort
Effect
SE
95%−CI
W(fixed)
W(random)
Phase 1 1.61 0.3755 1.61 [0.87; 2.34] 24.4% 30.3%
Phase 2 0.95 0.2344 0.95 [0.49; 1.41] 62.6% 50.5%
Phase 3 0.59 0.5157 0.59 [−0.42; 1.60] 12.9% 19.2%
Fixed effect model 1.07 [0.70; 1.43] 100% −−
Random effects model 1.08 [0.58; 1.58] −− 100%
Heterogeneity: I−squared=36.4%, p=0.2074
−2
−1
0
1
2
% DNA methylation difference

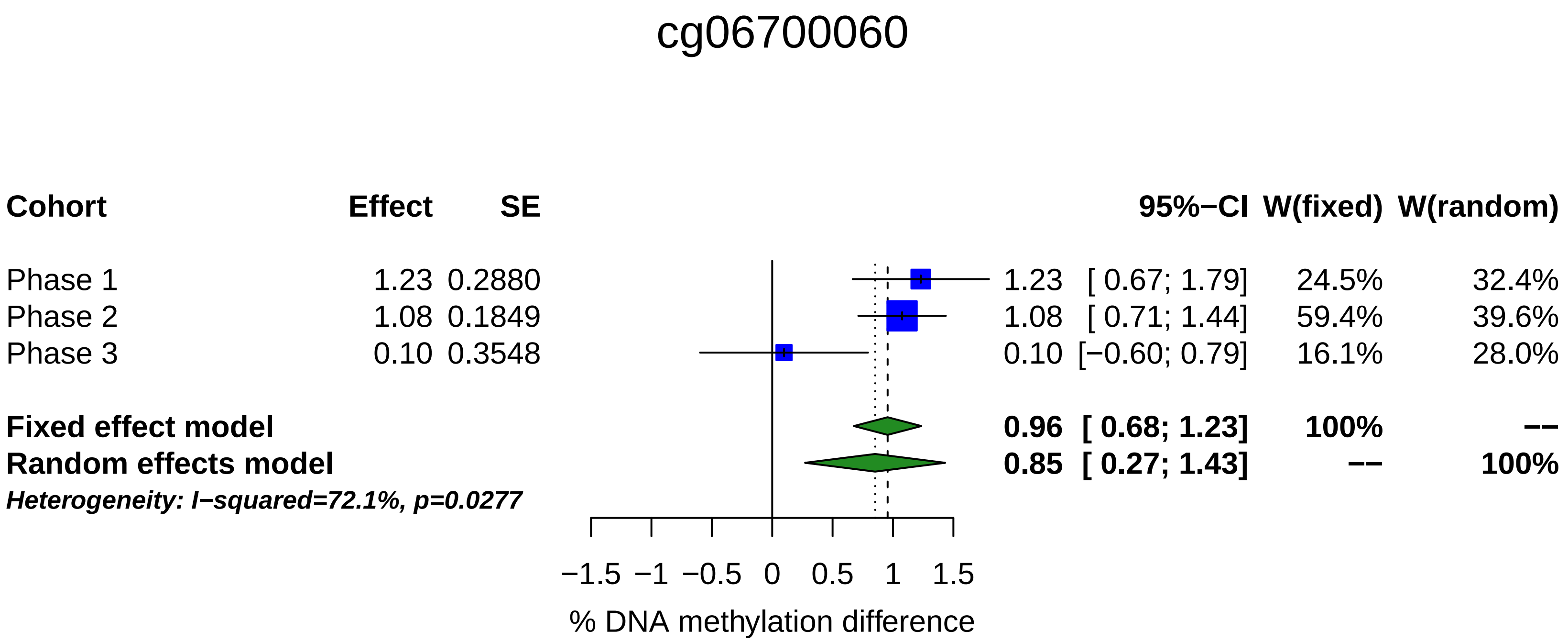
cg06700060
Cohort Effect SE 95%−CI W(fixed) W(random)
Phase 1 1.23 0.2880 1.23 [0.67; 1.79] 24.5% 32.4%
Phase 2 1.08 0.1849 1.08 [0.71; 1.44] 59.4% 39.6%
Phase 3 0.10 0.3548 0.10 [−0.60; 0.79] 16.1% 28.0%
Fixed effect model 0.96 [0.68; 1.23] 100% −−
Random effects model 0.85 [0.27; 1.43] −− 100%
Heterogeneity: I−squared=72.1%, p=0.0277
−1.5 −1 −0.5 0 0.5 1 1.5
% DNA methylation difference

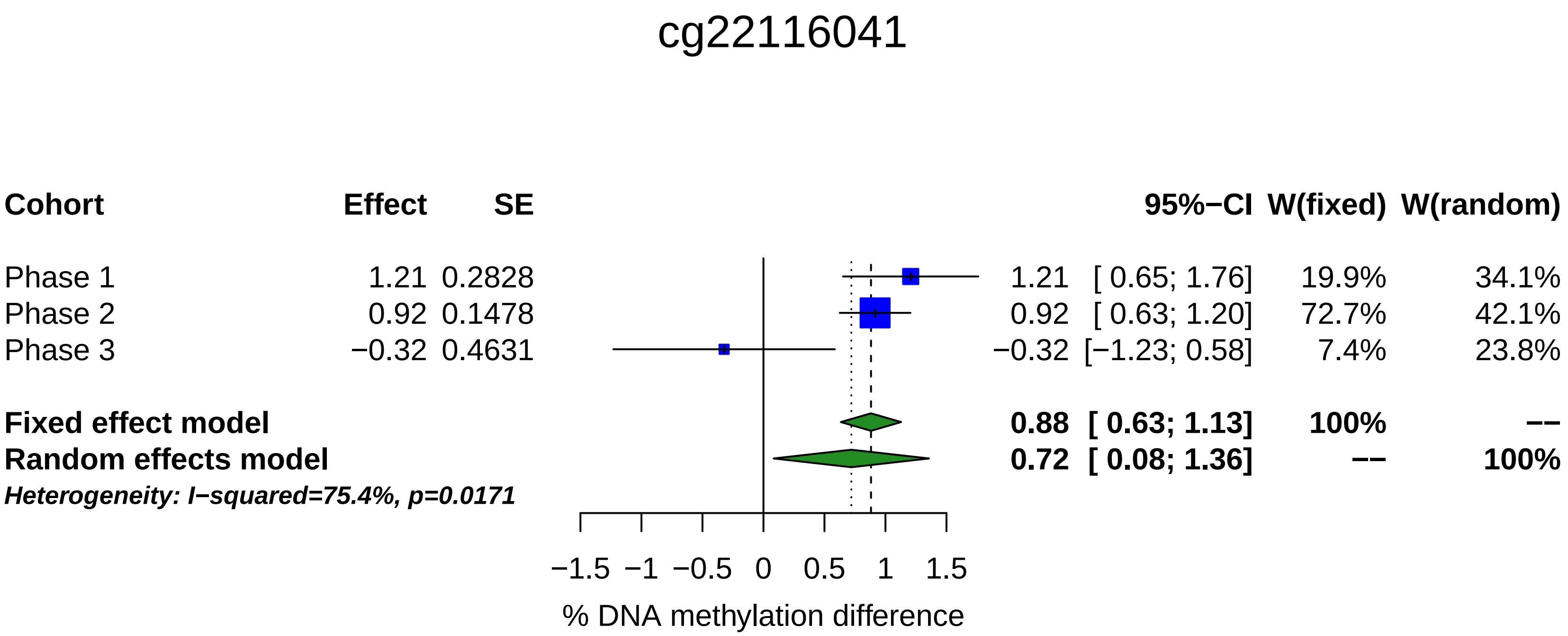
cg22116041
Cohort
Effect
SE
95%−CI
W(fixed)
W(random)
Phase 1 1.21 0.2828 1.21 [0.65; 1.76] 19.9% 34.1%
Phase 2 0.92 0.1478 0.92 [0.63; 1.20] 72.7% 42.1%
Phase 3 −0.32 0.4631 −0.32 [−1.23; 0.58] 7.4% 23.8%
Fixed effect model 0.88 [0.63; 1.13] 100% --
Random effects model 0.72 [0.08; 1.36] -- 100%
Heterogeneity: I−squared=75.4%, p=0.0171
−1.5 −1 −0.5 0 0.5 1 1.5
% DNA methylation difference

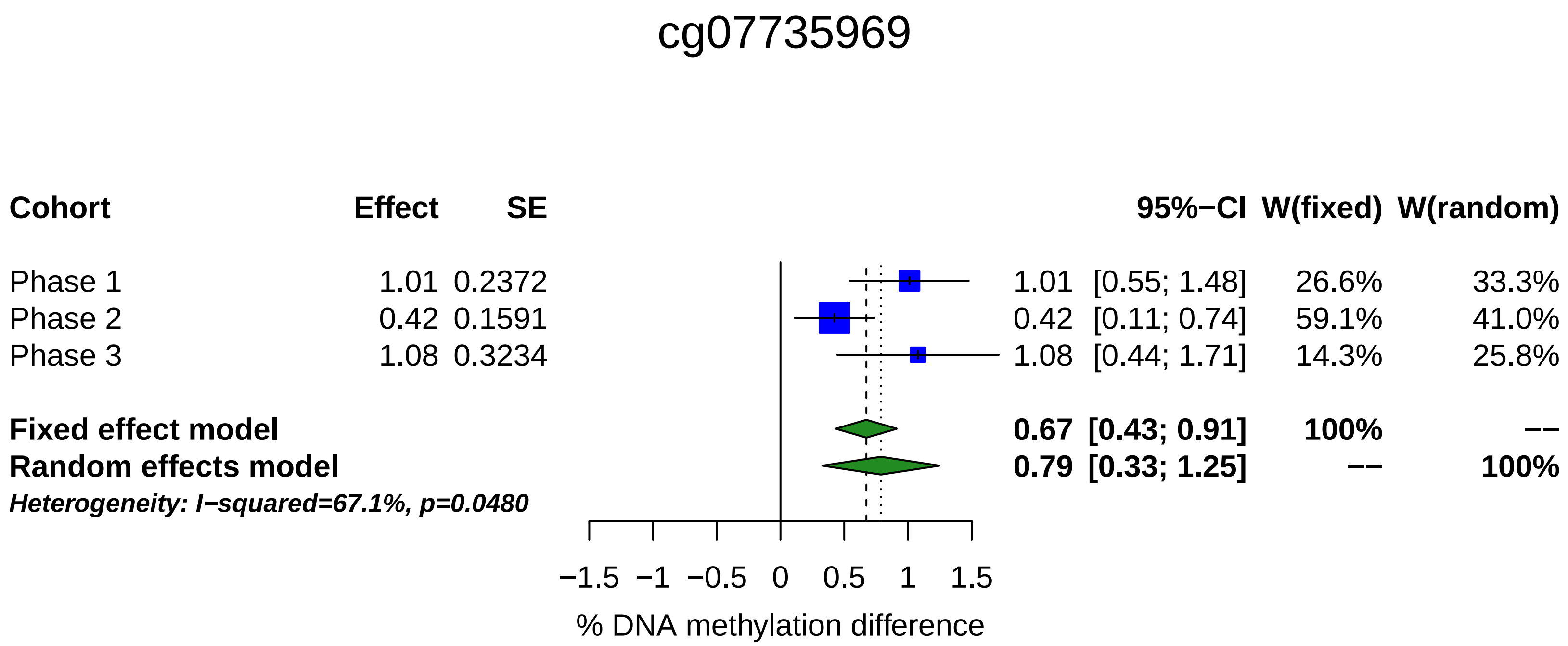
cg07735969
Cohort
Effect
SE
95%−CI
W(fixed)
W(random)
Phase 1 1.01 0.2372 1.01 [0.55; 1.48] 26.6% 33.3%
Phase 2 0.42 0.1591 0.42 [0.11; 0.74] 59.1% 41.0%
Phase 3 1.08 0.3234 1.08 [0.44; 1.71] 14.3% 25.8%
Fixed effect model 0.67 [0.43; 0.91] 100% −−
Random effects model 0.79 [0.33; 1.25] −− 100%
Heterogeneity: I−squared=67.1%, p=0.0480
−1.5 −1 −0.5 0 0.5 1 1.5
% DNA methylation difference

cg15969901

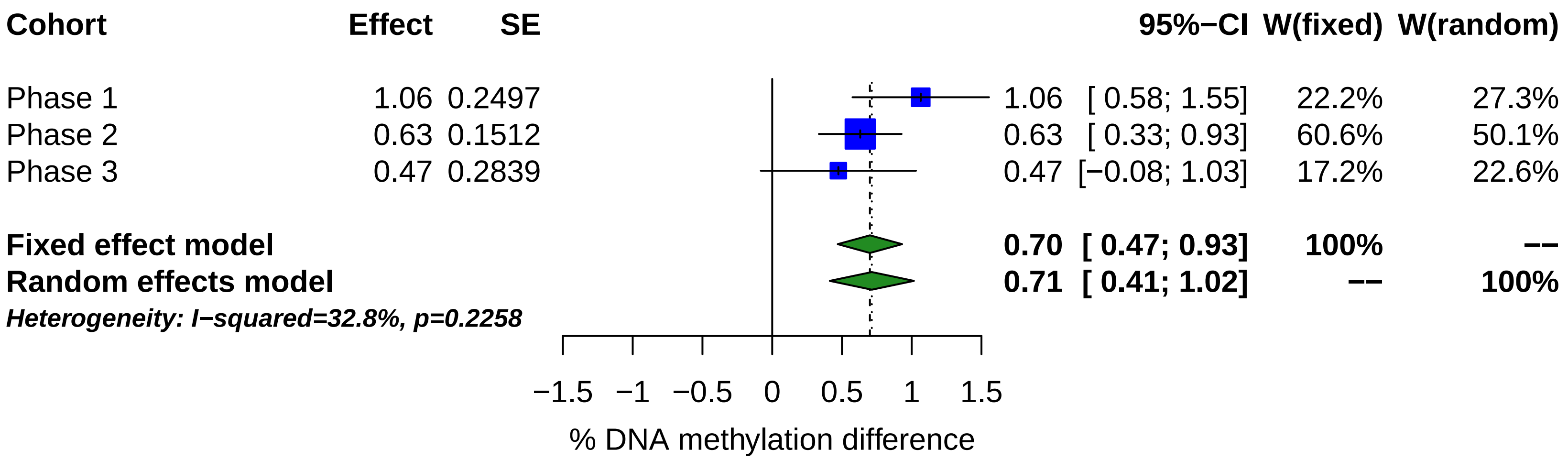
Cohort
Effect
SE
95%−CI
W(fixed)
W(random)
Phase 1 1.06 0.2497 1.06 [0.58; 1.55] 22.2% 27.3%
Phase 2 0.63 0.1512 0.63 [0.33; 0.93] 60.6% 50.1%
Phase 3 0.47 0.2839 0.47 [−0.08; 1.03] 17.2% 22.6%
Fixed effect model 0.70 [0.47; 0.93] 100% −−
Random effects model 0.71 [0.41; 1.02] −− 100%
Heterogeneity: I−squared=32.8%, p=0.2258
−1.5 −1 −0.5 0 0.5 1 1.5
% DNA methylation difference

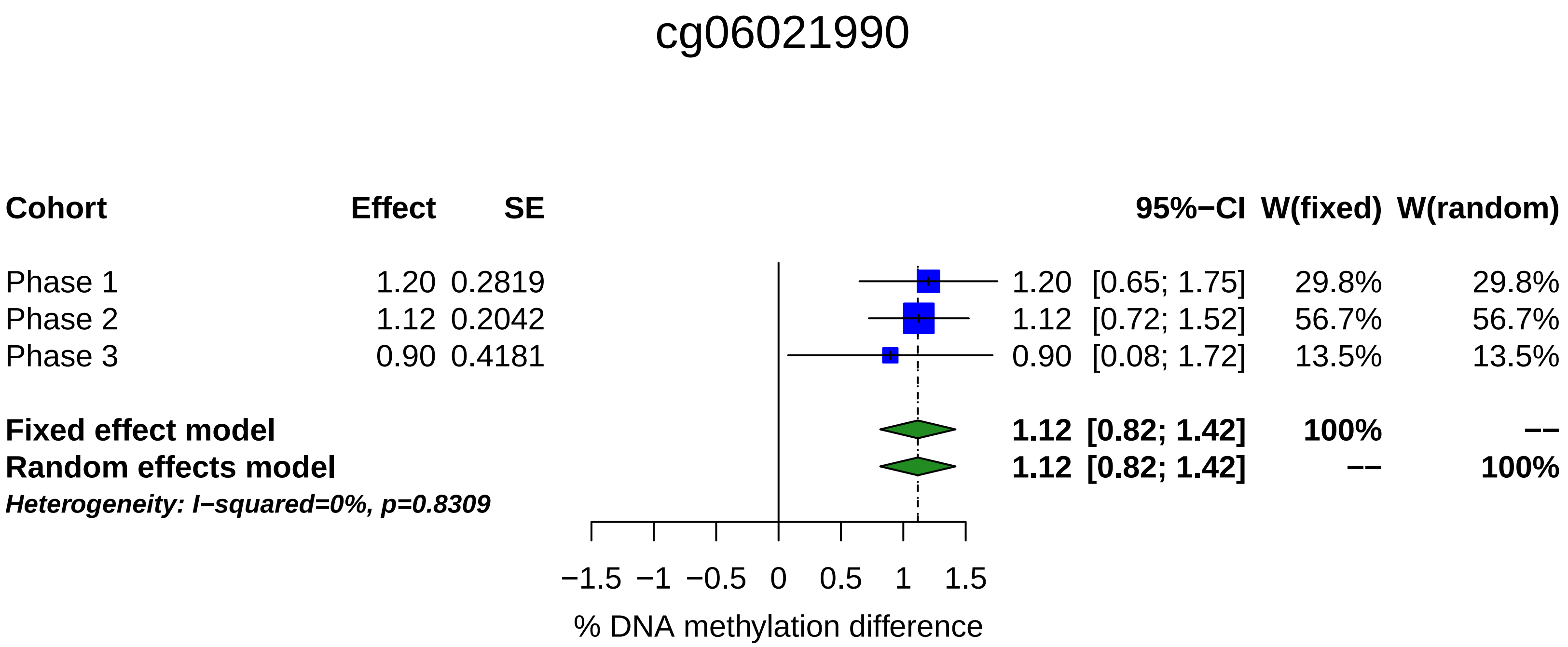
cg06021990
Cohort
Effect
SE
95%−CI
W(fixed)
W(random)
Phase 1 1.20 0.2819 1.20 [0.65; 1.75] 29.8% 29.8%
Phase 2 1.12 0.2042 1.12 [0.72; 1.52] 56.7% 56.7%
Phase 3 0.90 0.4181 0.90 [0.08; 1.72] 13.5% 13.5%
Fixed effect model 1.12 [0.82; 1.42] 100% −−
Random effects model 1.12 [0.82; 1.42] −− 100%
Heterogeneity: I−squared=0%, p=0.8309
−1.5 −1 −0.5 0 0.5 1 1.5
% DNA methylation difference

cg12332239

Cohort	Effect	SE		95%−CI	W(fixed)	W(random)
Phase 1	1.46	0.3435	1.46	[0.79; 2.14]	24.3%	29.4%
Phase 2	0.82	0.2162	0.82	[0.40; 1.24]	61.3%	50.5%
Phase 3	0.66	0.4449	0.66	[−0.21; 1.53]	14.5%	20.0%
**Fixed effect model**			**0.95**	**[0.62; 1.29]**	**100%**	**−−**
**Random effects model**			**0.98**	**[0.54; 1.42]**	**−−**	**100%**

Heterogeneity: I−squared=33.3%, p=0.2230

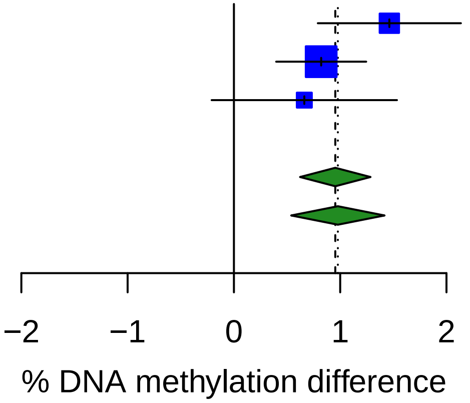

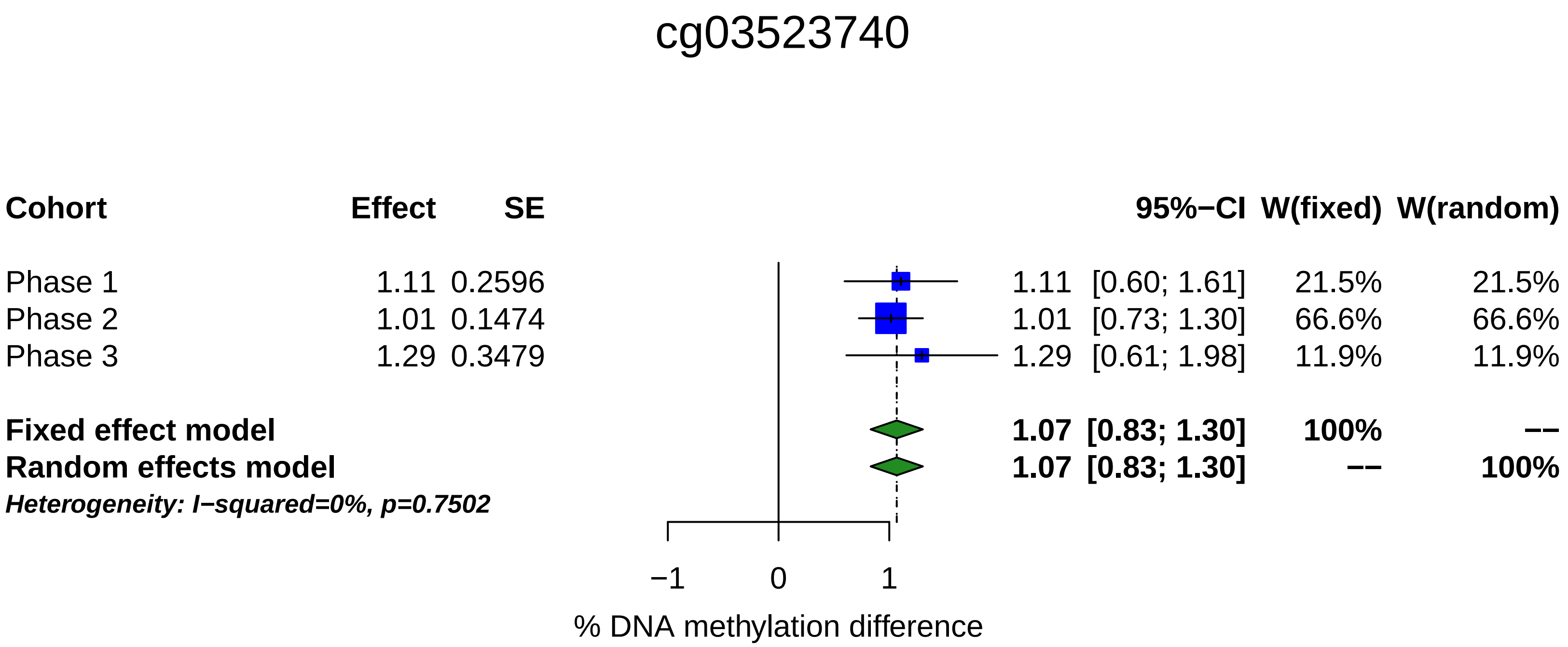
cg03523740
Cohort
Effect
SE
95%−CI
W(fixed)
W(random)
Phase 1 1.11 0.2596 1.11 [0.60; 1.61] 21.5% 21.5%
Phase 2 1.01 0.1474 1.01 [0.73; 1.30] 66.6% 66.6%
Phase 3 1.29 0.3479 1.29 [0.61; 1.98] 11.9% 11.9%
Fixed effect model 1.07 [0.83; 1.30] 100% −−
Random effects model 1.07 [0.83; 1.30] −− 100%
Heterogeneity: I−squared=0%, p=0.7502
−1
0
1
% DNA methylation difference

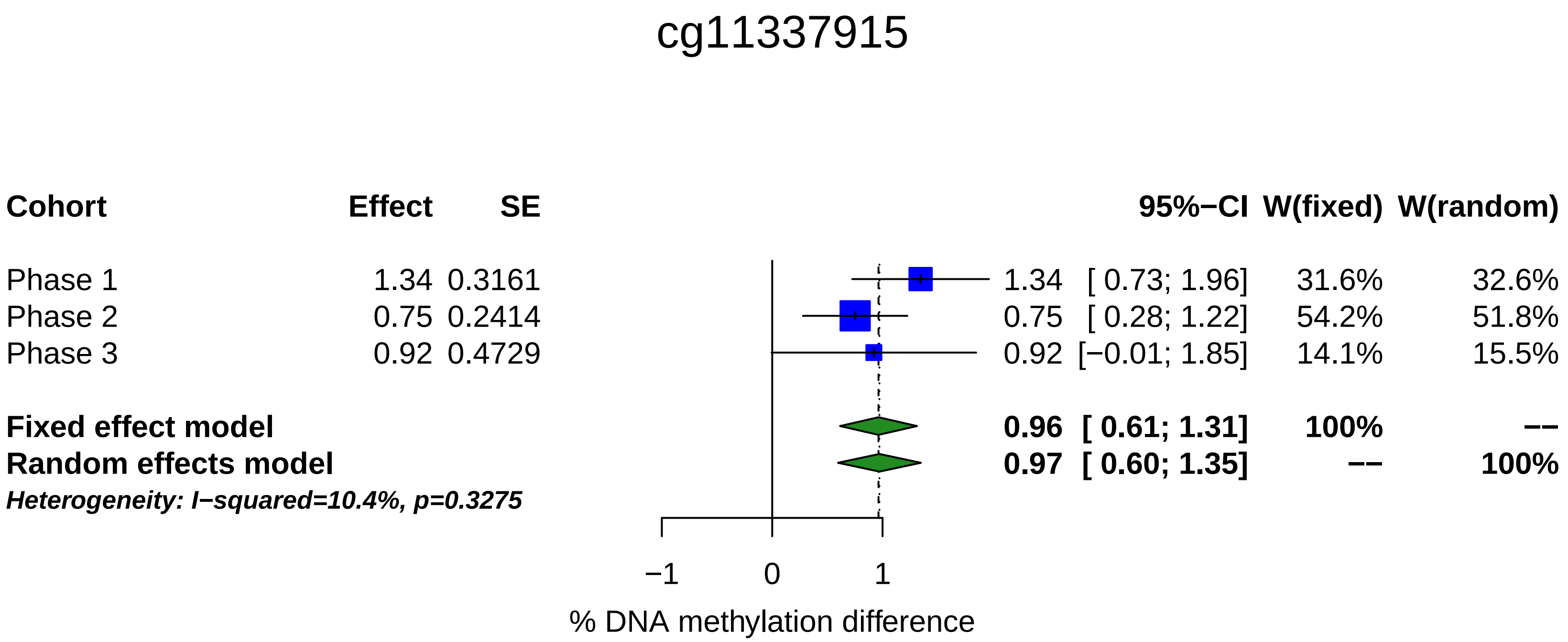
cg11337915
Cohort
Effect
SE
95%−CI
W(fixed)
W(random)
Phase 1 1.34 0.3161 1.34 [0.73; 1.96] 31.6% 32.6%
Phase 2 0.75 0.2414 0.75 [0.28; 1.22] 54.2% 51.8%
Phase 3 0.92 0.4729 0.92 [−0.01; 1.85] 14.1% 15.5%
Fixed effect model 0.96 [0.61; 1.31] 100% −−
Random effects model 0.97 [0.60; 1.35] −− 100%
Heterogeneity: I−squared=10.4%, p=0.3275
−1
0
1
% DNA methylation difference

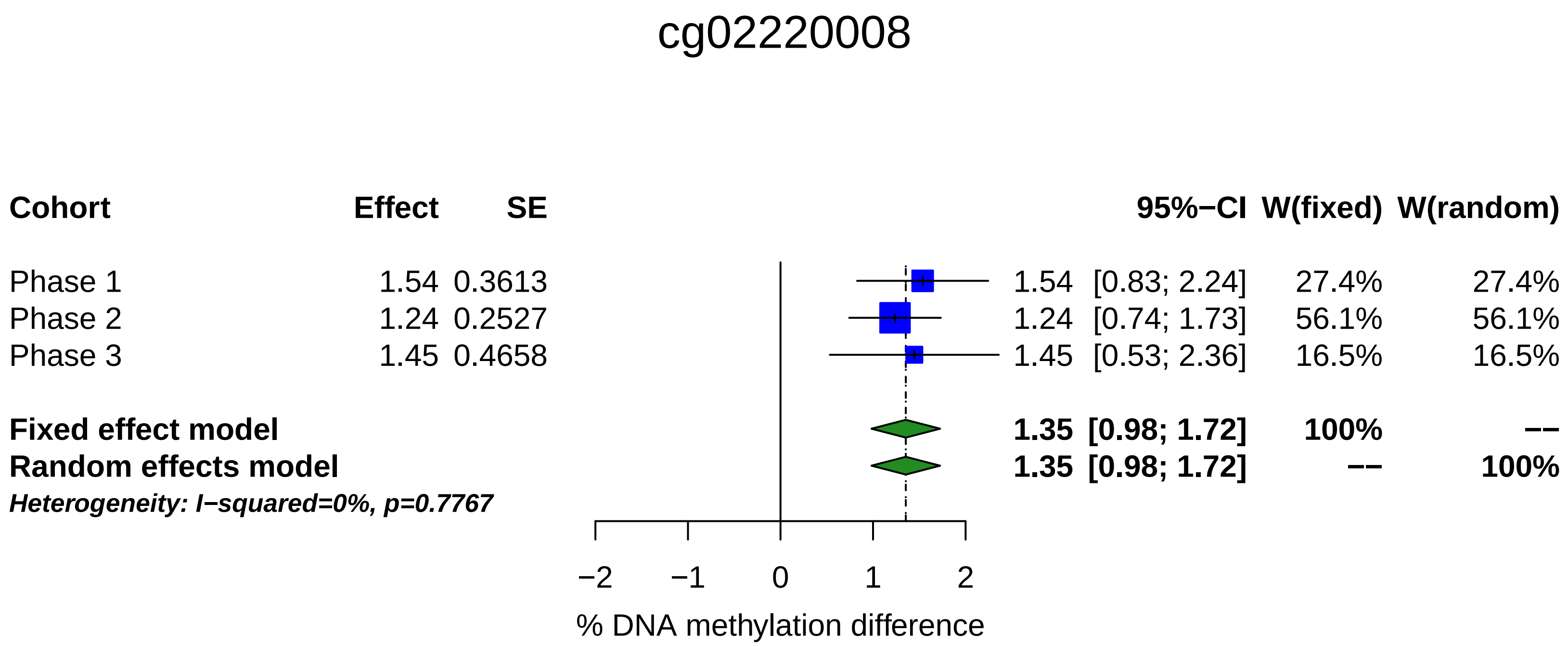
cg02220008
Cohort
Effect
SE
95%−CI
W(fixed)
W(random)
Phase 1 1.54 0.3613 1.54 [0.83; 2.24] 27.4% 27.4%
Phase 2 1.24 0.2527 1.24 [0.74; 1.73] 56.1% 56.1%
Phase 3 1.45 0.4658 1.45 [0.53; 2.36] 16.5% 16.5%
Fixed effect model 1.35 [0.98; 1.72] 100% −−
Random effects model 1.35 [0.98; 1.72] −− 100%
Heterogeneity: I−squared=0%, p=0.7767
−2
−1
0
1
2
% DNA methylation difference

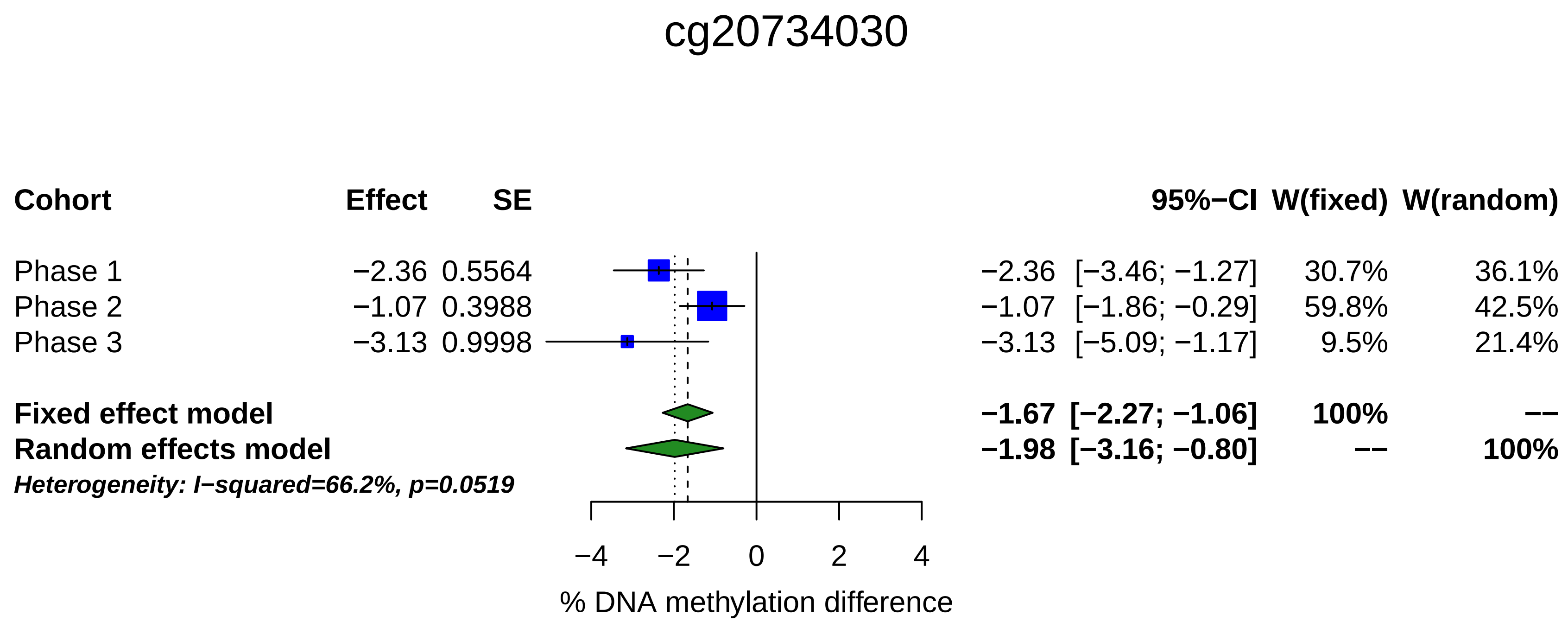
cg20734030
Cohort
Effect
SE
95%−CI
W(fixed)
W(random)
Phase 1 −2.36 0.5564 −2.36 [−3.46; −1.27] 30.7% 36.1%
Phase 2 −1.07 0.3988 −1.07 [−1.86; −0.29] 59.8% 42.5%
Phase 3 −3.13 0.9998 −3.13 [−5.09; −1.17] 9.5% 21.4%
Fixed effect model −1.67 [−2.27; −1.06] 100% −−
Random effects model −1.98 [−3.16; −0.80] −− 100%
Heterogeneity: I−squared=66.2%, p=0.0519
−4
−2
0
2
4
% DNA methylation difference

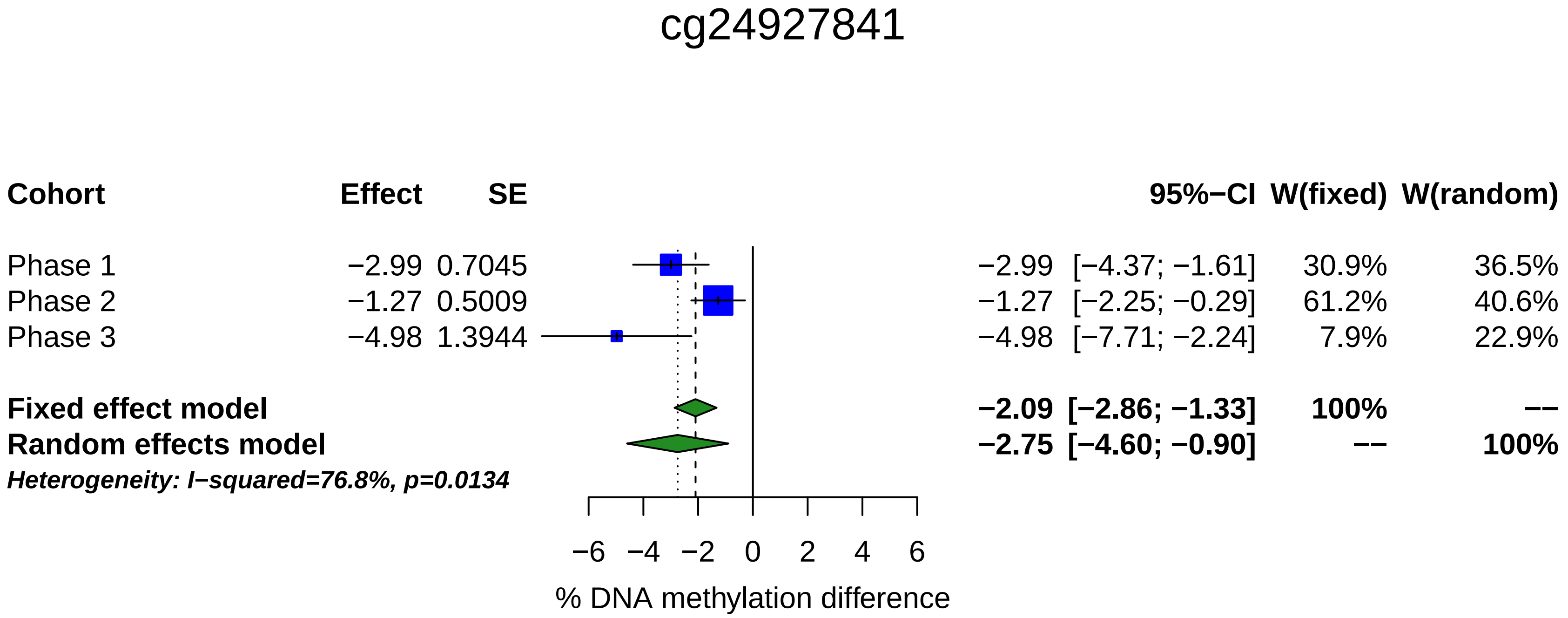
cg24927841
Cohort
Effect
SE
95%−CI
W(fixed)
W(random)
Phase 1 −2.99 0.7045 −2.99 [−4.37; −1.61] 30.9% 36.5%
Phase 2 −1.27 0.5009 −1.27 [−2.25; −0.29] 61.2% 40.6%
Phase 3 −4.98 1.3944 −4.98 [−7.71; −2.24] 7.9% 22.9%
Fixed effect model −2.09 [−2.86; −1.33] 100% −−
Random effects model −2.75 [−4.60; −0.90] −− 100%
Heterogeneity: I−squared=76.8%, p=0.0134
−6 −4 −2 0 2 4 6
% DNA methylation difference

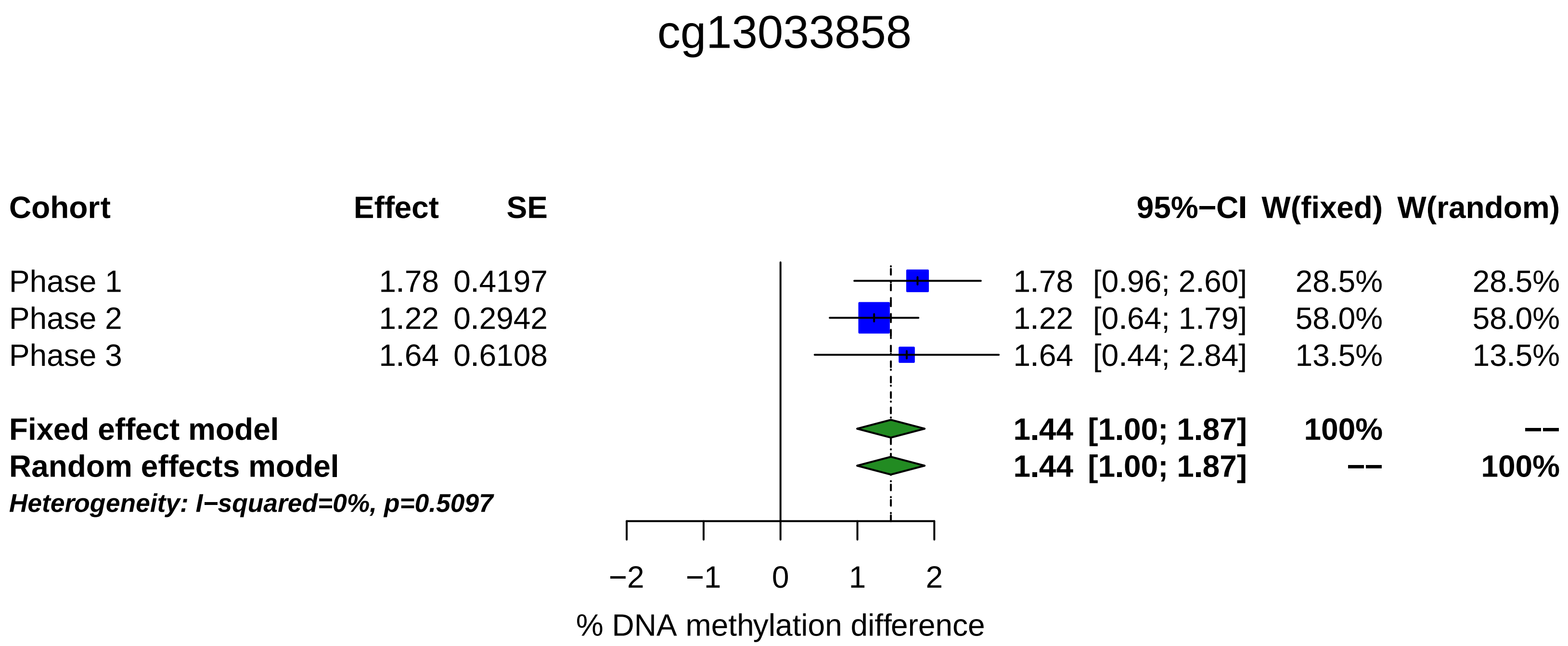
cg13033858
Cohort
Effect
SE
95%−CI
W(fixed)
W(random)
Phase 1 1.78 0.4197 1.78 [0.96; 2.60] 28.5% 28.5%
Phase 2 1.22 0.2942 1.22 [0.64; 1.79] 58.0% 58.0%
Phase 3 1.64 0.6108 1.64 [0.44; 2.84] 13.5% 13.5%
Fixed effect model 1.44 [1.00; 1.87] 100% −−
Random effects model 1.44 [1.00; 1.87] −− 100%
Heterogeneity: I−squared=0%, p=0.5097
−2
−1
0
1
2
% DNA methylation difference

cg03035167

Cohort	Effect	SE	95%−CI	W(fixed)	W(random)
Phase 1	1.23	0.2907	1.23 [0.66; 1.80]	27.2%	33.5%
Phase 2	1.29	0.1956	1.29 [0.90; 1.67]	60.1%	44.2%
Phase 3	0.30	0.4248	0.30 [−0.53; 1.14]	12.7%	22.3%
**Fixed effect model**			**1.15 [0.85; 1.44]**	**100%**	**−−**
**Random effects model**			**1.05 [0.55; 1.55]**	**−−**	**100%**

Heterogeneity: I−squared=55.9%, p=0.1034

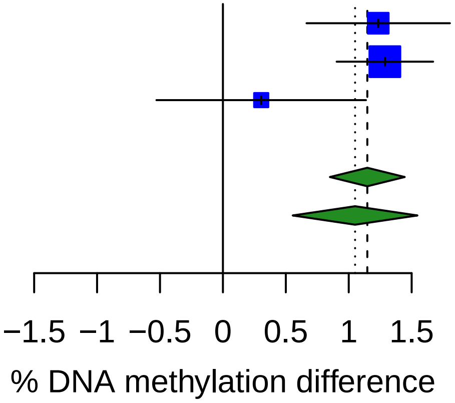

cg04275692

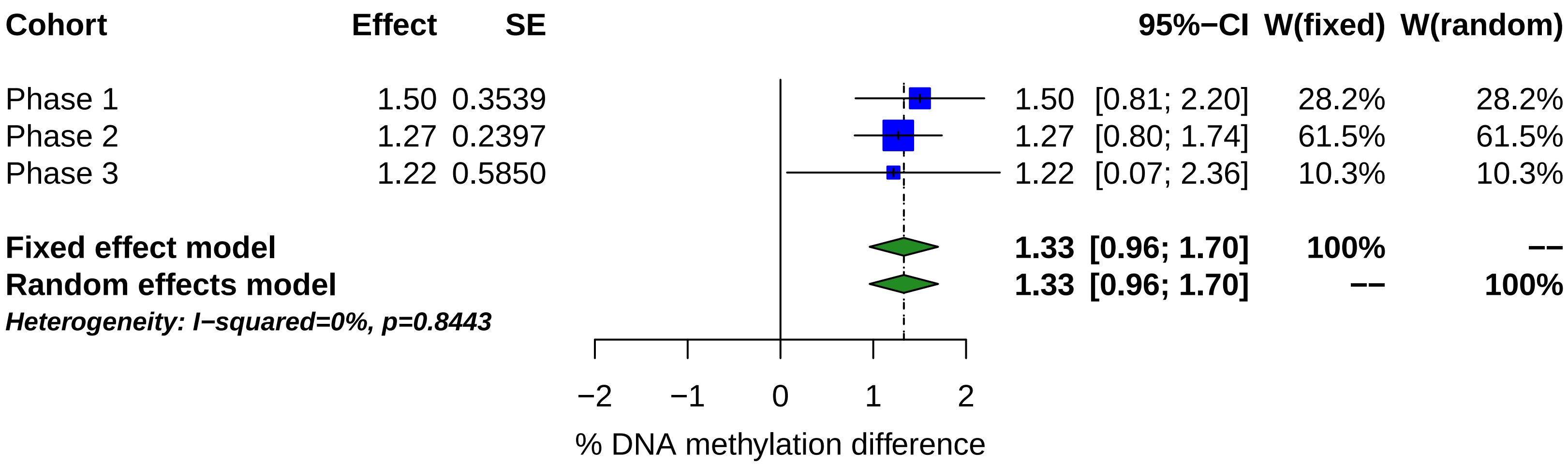
Cohort
Effect
SE
95%−CI
W(fixed)
W(random)
Phase 1 1.50 0.3539 1.50 [0.81; 2.20] 28.2% 28.2%
Phase 2 1.27 0.2397 1.27 [0.80; 1.74] 61.5% 61.5%
Phase 3 1.22 0.5850 1.22 [0.07; 2.36] 10.3% 10.3%
Fixed effect model 1.33 [0.96; 1.70] 100% −−
Random effects model 1.33 [0.96; 1.70] −− 100%
Heterogeneity: I−squared=0%, p=0.8443
−2
−1
0
1
2
% DNA methylation difference

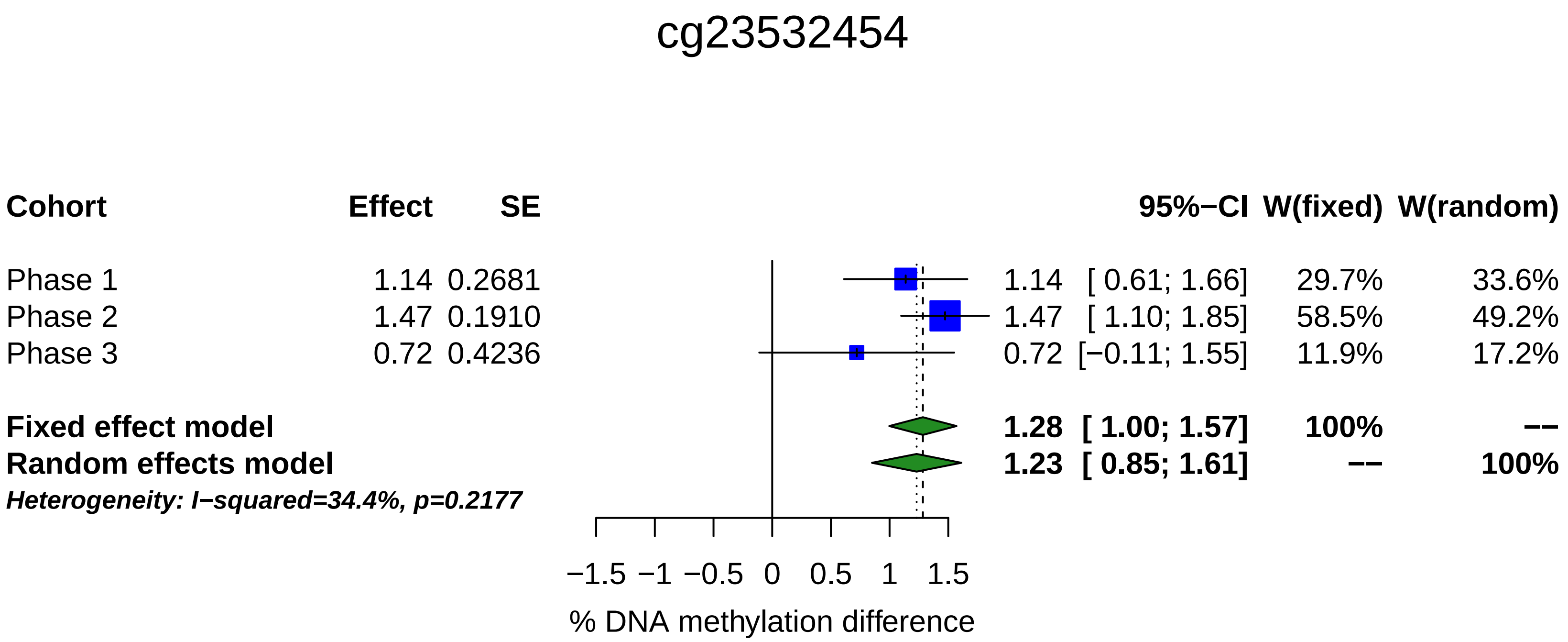
cg23532454
Cohort
Effect
SE
95%−CI
W(fixed)
W(random)
Phase 1 1.14 0.2681 1.14 [0.61; 1.66] 29.7% 33.6%
Phase 2 1.47 0.1910 1.47 [1.10; 1.85] 58.5% 49.2%
Phase 3 0.72 0.4236 0.72 [−0.11; 1.55] 11.9% 17.2%
Fixed effect model 1.28 [1.00; 1.57] 100% −−
Random effects model 1.23 [0.85; 1.61] −− 100%
Heterogeneity: I−squared=34.4%, p=0.2177
−1.5
−1
−0.5
0
0.5
1
1.5
% DNA methylation difference

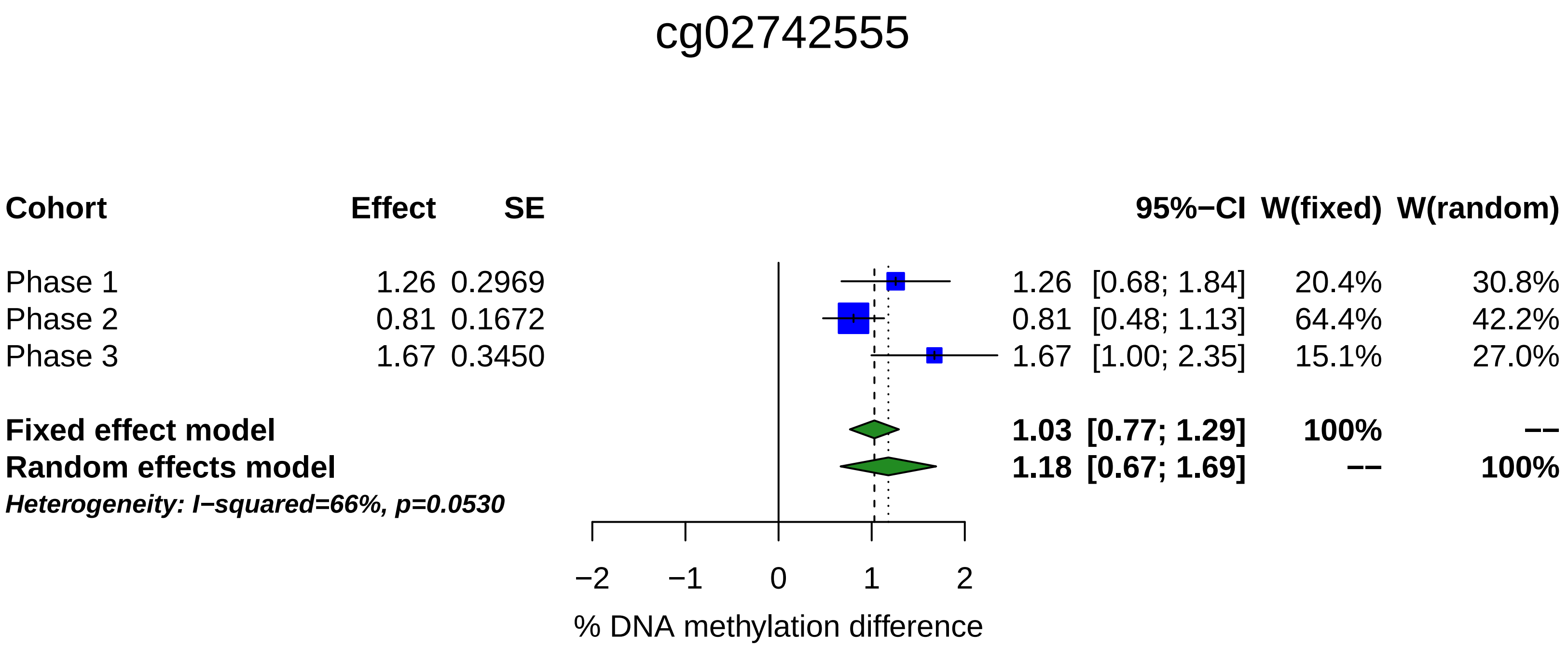
cg02742555
Cohort
Effect
SE
95%−CI
W(fixed)
W(random)
Phase 1 1.26 0.2969 1.26 [0.68; 1.84] 20.4% 30.8%
Phase 2 0.81 0.1672 0.81 [0.48; 1.13] 64.4% 42.2%
Phase 3 1.67 0.3450 1.67 [1.00; 2.35] 15.1% 27.0%
Fixed effect model 1.03 [0.77; 1.29] 100% −−
Random effects model 1.18 [0.67; 1.69] −− 100%
Heterogeneity: I−squared=66%, p=0.0530
−2
−1
0
1
2
% DNA methylation difference

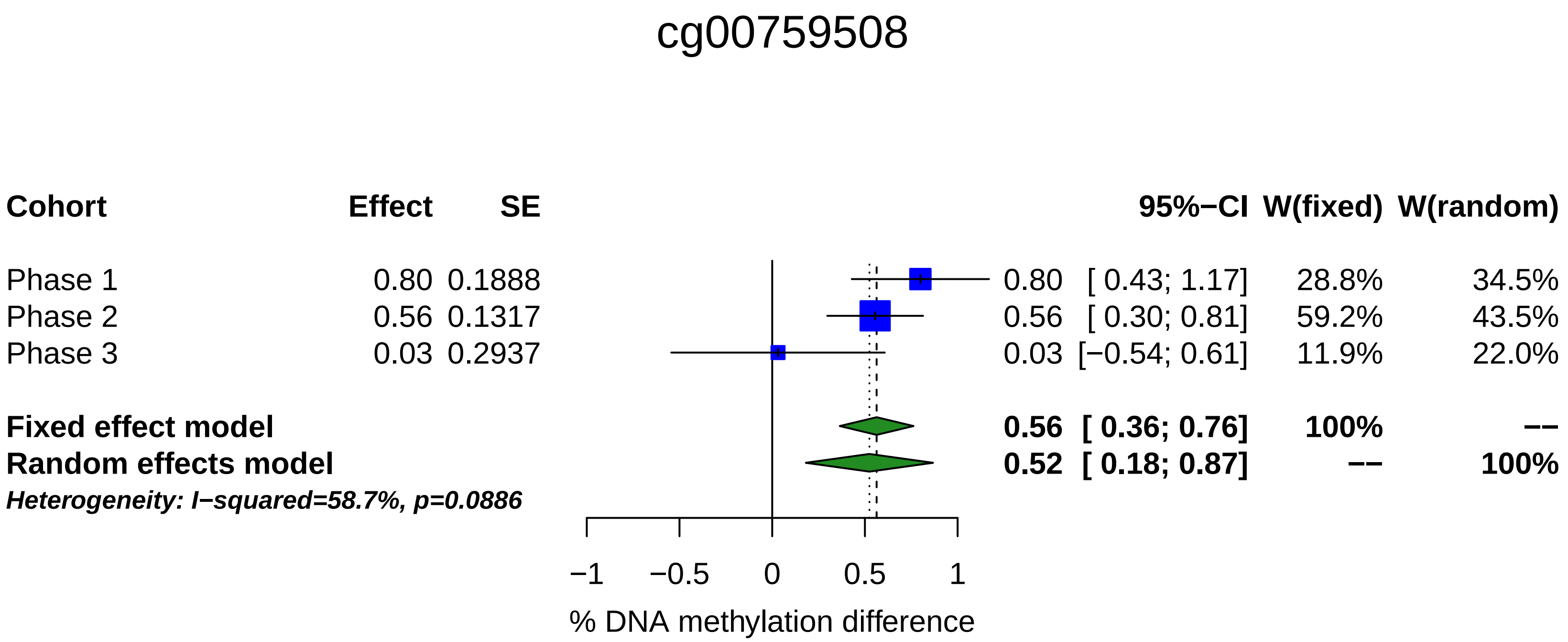
cg00759508
Cohort
Effect
SE
95%−CI
W(fixed)
W(random)
Phase 1 0.80 0.1888 0.80 [0.43; 1.17] 28.8% 34.5%
Phase 2 0.56 0.1317 0.56 [0.30; 0.81] 59.2% 43.5%
Phase 3 0.03 0.2937 0.03 [−0.54; 0.61] 11.9% 22.0%
Fixed effect model 0.56 [0.36; 0.76] 100% −−
Random effects model 0.52 [0.18; 0.87] −− 100%
Heterogeneity: I−squared=58.7%, p=0.0886
−1
−0.5
0
0.5
1
% DNA methylation difference

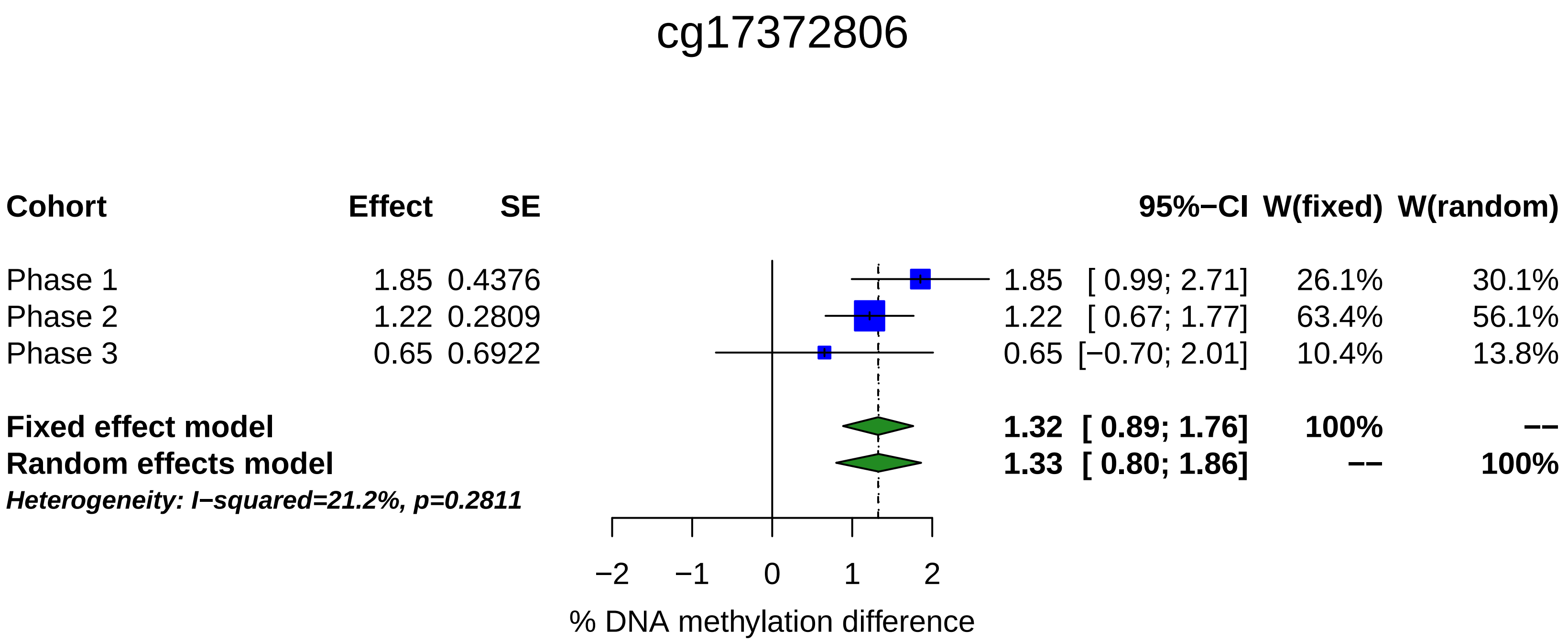
cg17372806
Cohort
Effect
SE
95%−CI
W(fixed)
W(random)
Phase 1 1.85 0.4376 1.85 [0.99; 2.71] 26.1% 30.1%
Phase 2 1.22 0.2809 1.22 [0.67; 1.77] 63.4% 56.1%
Phase 3 0.65 0.6922 0.65 [−0.70; 2.01] 10.4% 13.8%
Fixed effect model 1.32 [0.89; 1.76] 100% −−
Random effects model 1.33 [0.80; 1.86] −− 100%
Heterogeneity: I−squared=21.2%, p=0.2811
−2
−1
0
1
2
% DNA methylation difference

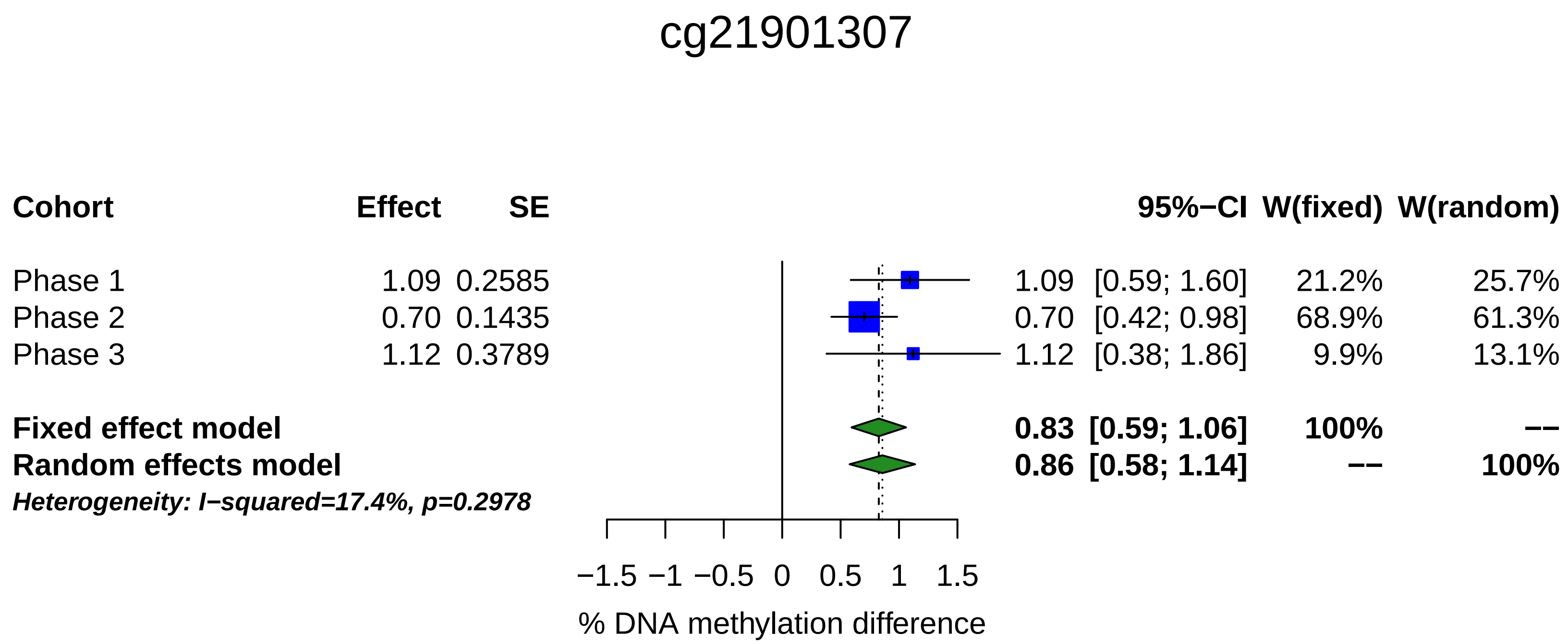
cg21901307
Cohort
Effect
SE
95%−CI
W(fixed)
W(random)
Phase 1 1.09 0.2585 1.09 [0.59; 1.60] 21.2% 25.7%
Phase 2 0.70 0.1435 0.70 [0.42; 0.98] 68.9% 61.3%
Phase 3 1.12 0.3789 1.12 [0.38; 1.86] 9.9% 13.1%
Fixed effect model 0.83 [0.59; 1.06] 100% −−
Random effects model 0.86 [0.58; 1.14] −− 100%
Heterogeneity: I−squared=17.4%, p=0.2978
−1.5 −1 −0.5 0 0.5 1 1.5
% DNA methylation difference

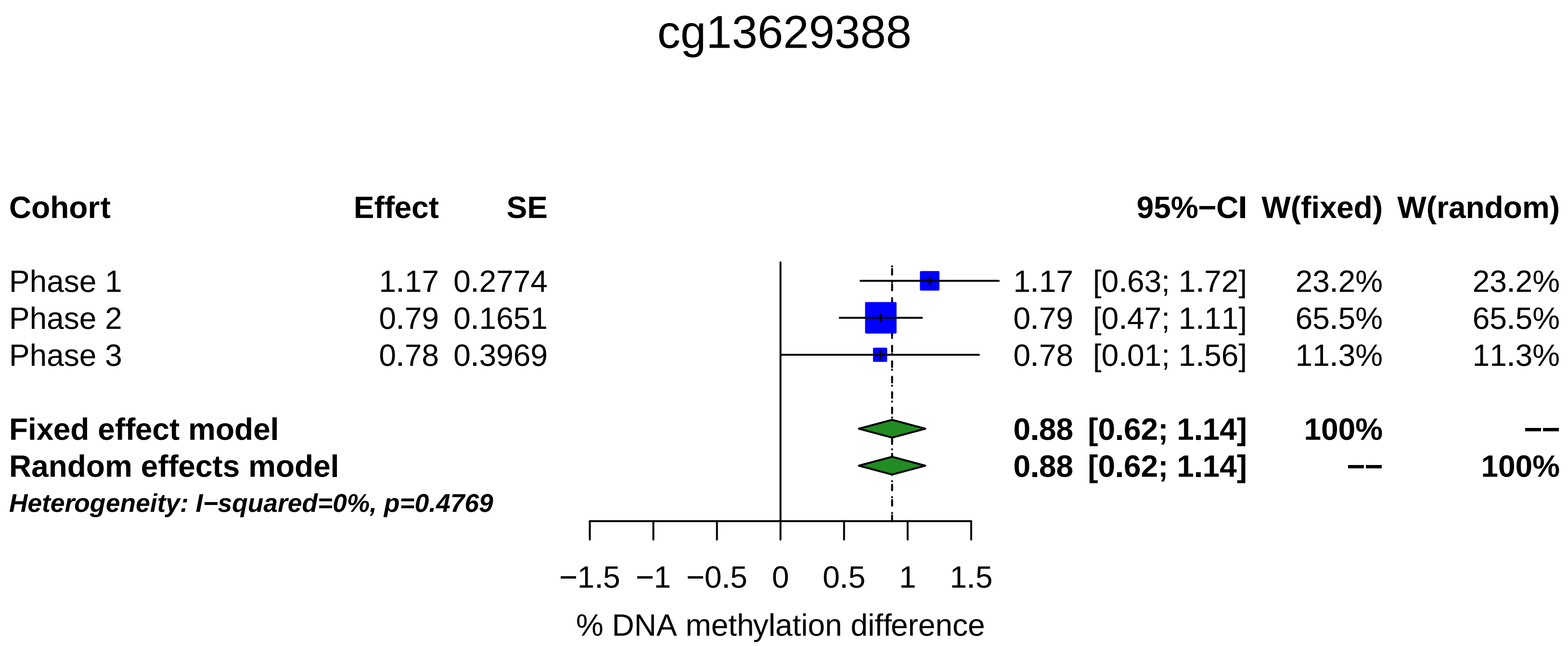
cg13629388
Cohort
Effect
SE
95%−CI
W(fixed)
W(random)
Phase 1 1.17 0.2774 1.17 [0.63; 1.72] 23.2% 23.2%
Phase 2 0.79 0.1651 0.79 [0.47; 1.11] 65.5% 65.5%
Phase 3 0.78 0.3969 0.78 [0.01; 1.56] 11.3% 11.3%
Fixed effect model 0.88 [0.62; 1.14] 100% −−
Random effects model 0.88 [0.62; 1.14] −− 100%
Heterogeneity: I−squared=0%, p=0.4769
−1.5 −1 −0.5 0 0.5 1 1.5
% DNA methylation difference

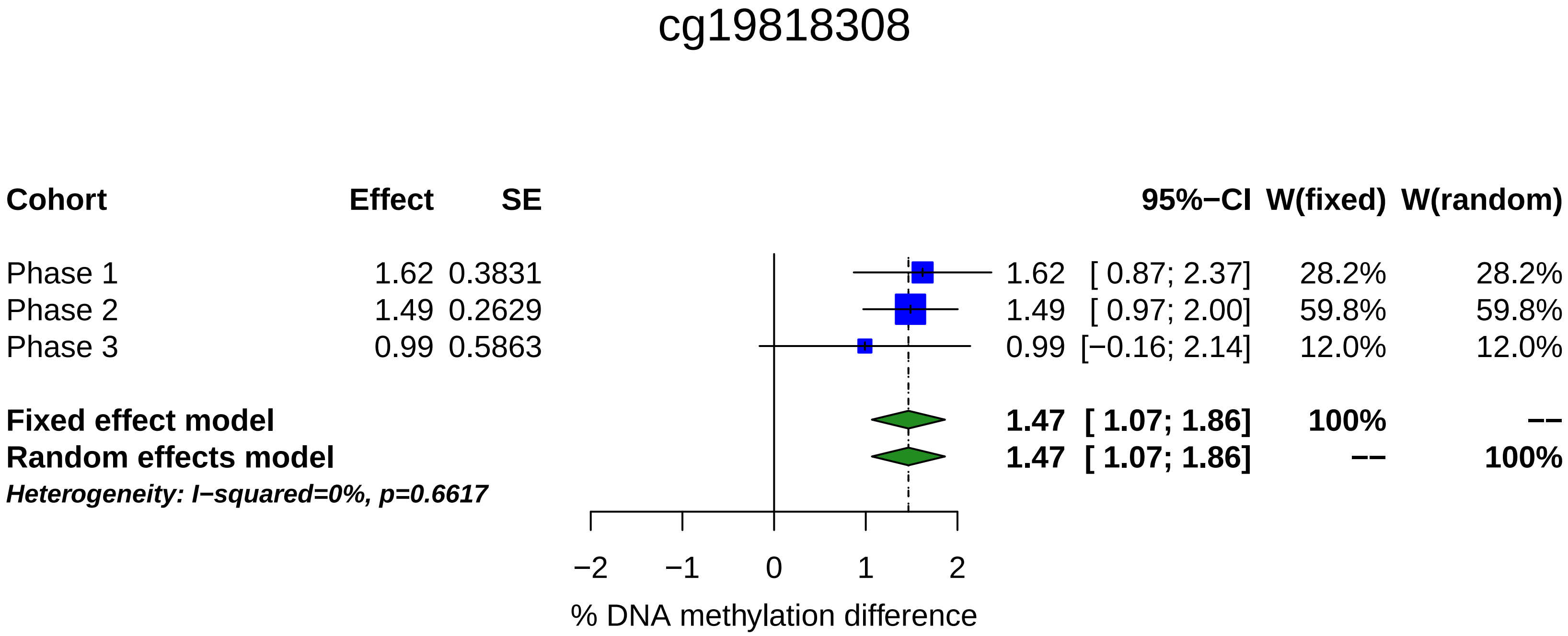
cg19818308
Cohort
Effect
SE
95%−CI
W(fixed)
W(random)
Phase 1
1.62
0.3831
1.62 [0.87; 2.37]
28.2%
28.2%
Phase 2
1.49
0.2629
1.49 [0.97; 2.00]
59.8%
59.8%
Phase 3
0.99
0.5863
0.99 [−0.16; 2.14]
12.0%
12.0%
Fixed effect model
1.47 [1.07; 1.86]
100%
−−
Random effects model
1.47 [1.07; 1.86]
−−
100%
Heterogeneity: I−squared=0%, p=0.6617
−2
−1
0
1
2
% DNA methylation difference

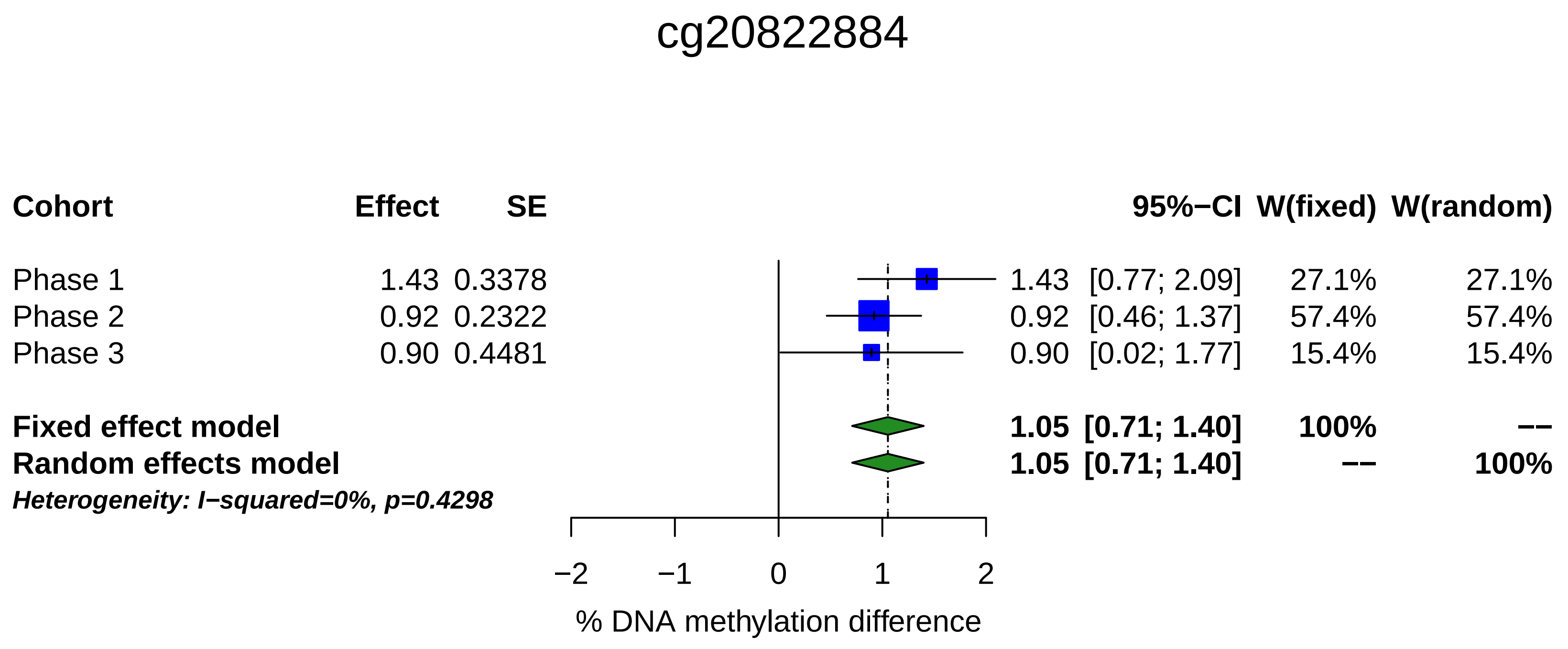
cg20822884
Cohort
Effect
SE
95%−CI
W(fixed)
W(random)
Phase 1 1.43 0.3378 1.43 [0.77; 2.09] 27.1% 27.1%
Phase 2 0.92 0.2322 0.92 [0.46; 1.37] 57.4% 57.4%
Phase 3 0.90 0.4481 0.90 [0.02; 1.77] 15.4% 15.4%
Fixed effect model 1.05 [0.71; 1.40] 100% −−
Random effects model 1.05 [0.71; 1.40] −− 100%
Heterogeneity: I−squared=0%, p=0.4298
−2
−1
0
1
2
% DNA methylation difference

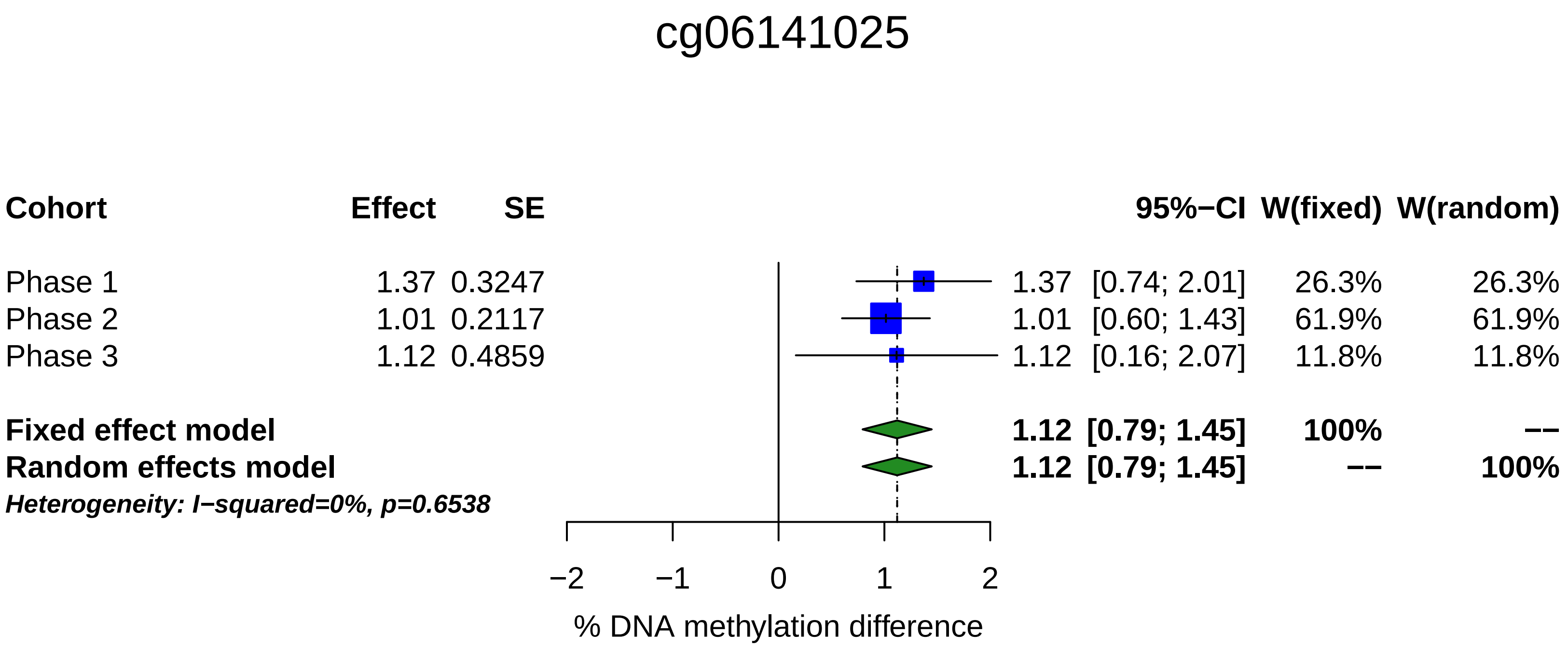
cg06141025
Cohort
Effect
SE
95%−CI
W(fixed)
W(random)
Phase 1 1.37 0.3247 1.37 [0.74; 2.01] 26.3% 26.3%
Phase 2 1.01 0.2117 1.01 [0.60; 1.43] 61.9% 61.9%
Phase 3 1.12 0.4859 1.12 [0.16; 2.07] 11.8% 11.8%
Fixed effect model 1.12 [0.79; 1.45] 100% −−
Random effects model 1.12 [0.79; 1.45] −− 100%
Heterogeneity: I−squared=0%, p=0.6538
−2
−1
0
1
2
% DNA methylation difference

cg26829395

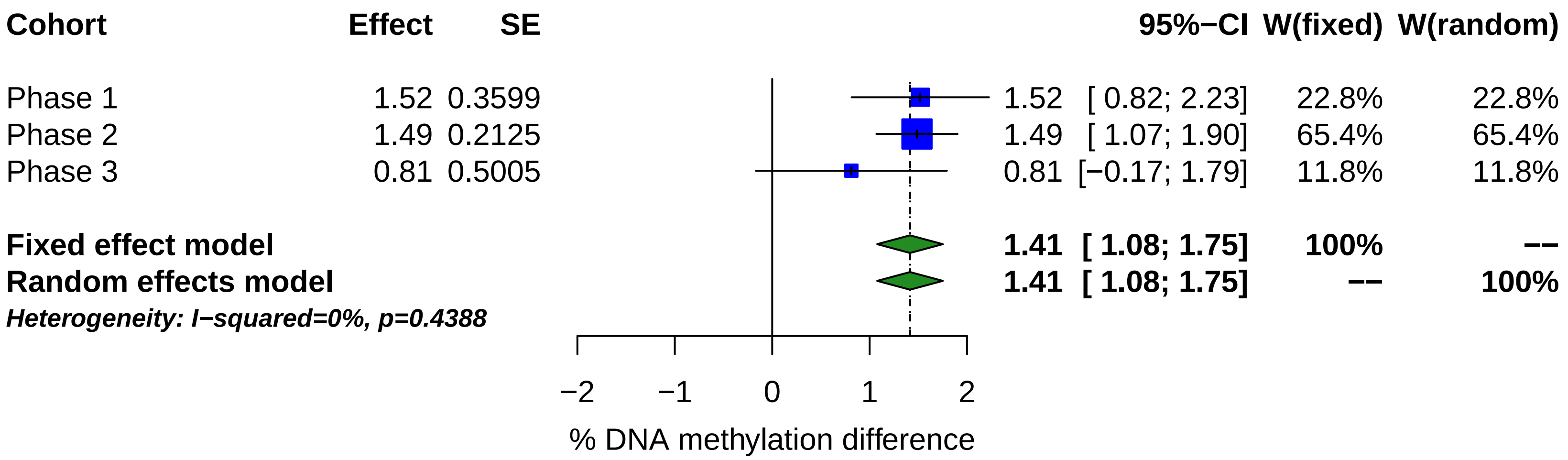
Cohort
Effect
SE
95%−CI
W(fixed)
W(random)
Phase 1 1.52 0.3599 1.52 [0.82; 2.23] 22.8% 22.8%
Phase 2 1.49 0.2125 1.49 [1.07; 1.90] 65.4% 65.4%
Phase 3 0.81 0.5005 0.81 [−0.17; 1.79] 11.8% 11.8%
Fixed effect model 1.41 [1.08; 1.75] 100% −−
Random effects model 1.41 [1.08; 1.75] −− 100%
Heterogeneity: I−squared=0%, p=0.4388
−2
−1
0
1
2
% DNA methylation difference

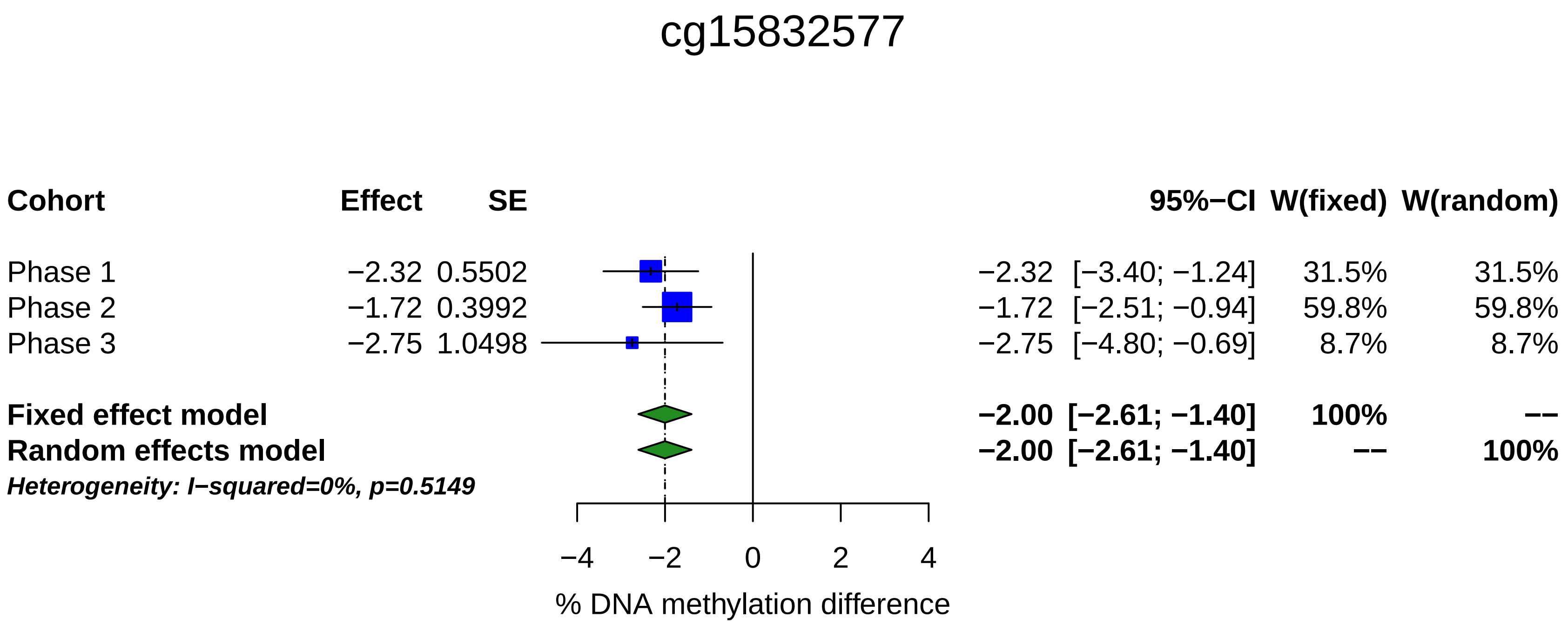
cg15832577
Cohort
Effect
SE
95%−CI
W(fixed)
W(random)
Phase 1 −2.32 0.5502 −2.32 [−3.40; −1.24] 31.5% 31.5%
Phase 2 −1.72 0.3992 −1.72 [−2.51; −0.94] 59.8% 59.8%
Phase 3 −2.75 1.0498 −2.75 [−4.80; −0.69] 8.7% 8.7%
Fixed effect model −2.00 [−2.61; −1.40] 100% −−
Random effects model −2.00 [−2.61; −1.40] −− 100%
Heterogeneity: I−squared=0%, p=0.5149
−4
−2
0
2
4
% DNA methylation difference

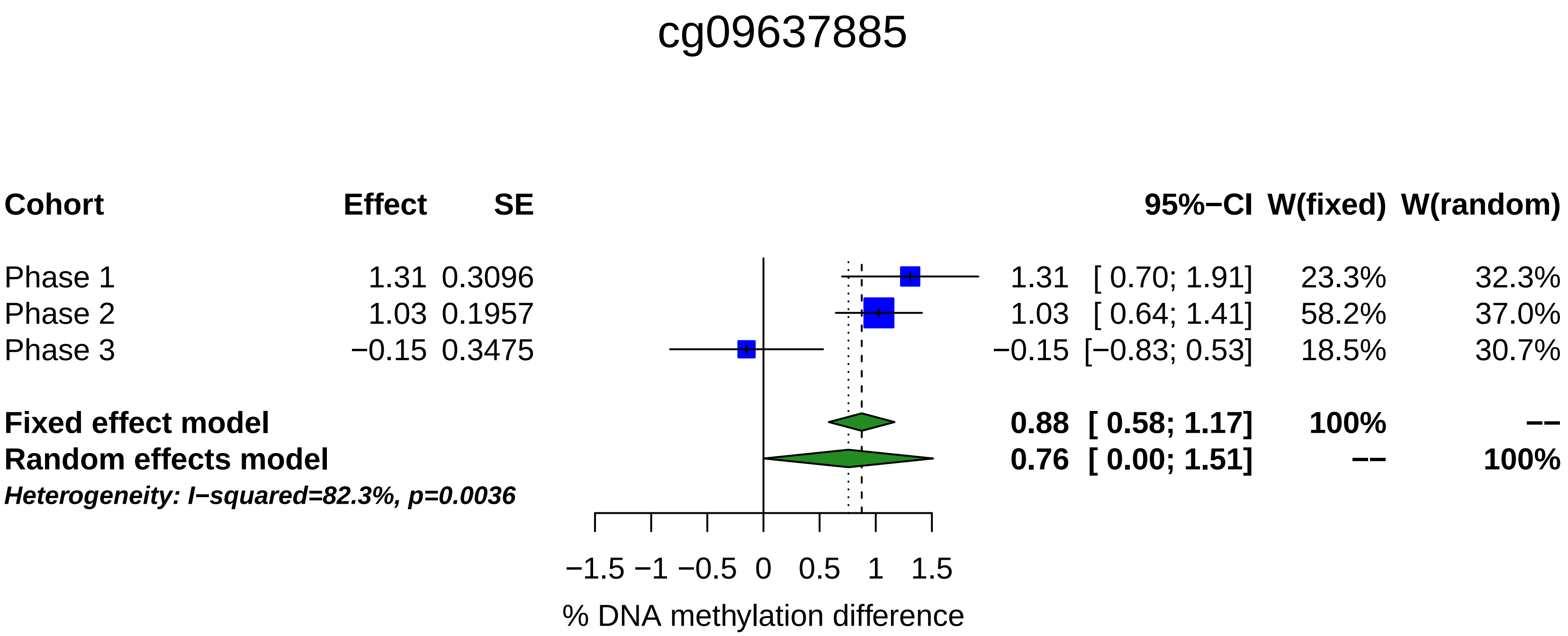
cg09637885
Cohort
Effect
SE
95%−CI
W(fixed)
W(random)
Phase 1 1.31 0.3096 1.31 [0.70; 1.91] 23.3% 32.3%
Phase 2 1.03 0.1957 1.03 [0.64; 1.41] 58.2% 37.0%
Phase 3 −0.15 0.3475 −0.15 [−0.83; 0.53] 18.5% 30.7%
Fixed effect model 0.88 [0.58; 1.17] 100% −−
Random effects model 0.76 [0.00; 1.51] −− 100%
Heterogeneity: I−squared=82.3%, p=0.0036
−1.5 −1 −0.5 0 0.5 1 1.5
% DNA methylation difference

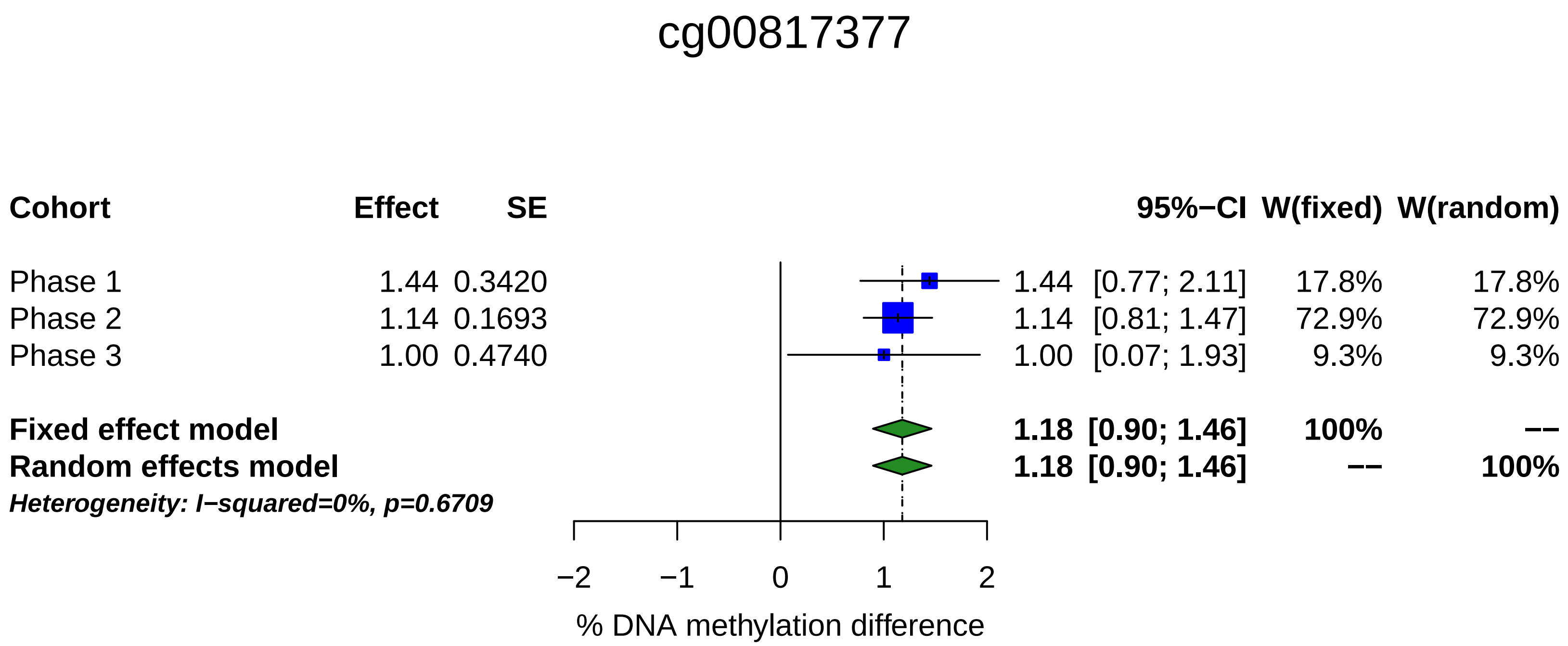
cg00817377
Cohort
Effect
SE
95%−CI
W(fixed)
W(random)
Phase 1
1.44 0.3420
1.44 [0.77; 2.11] 17.8% 17.8%
Phase 2
1.14 0.1693
1.14 [0.81; 1.47] 72.9% 72.9%
Phase 3
1.00 0.4740
1.00 [0.07; 1.93] 9.3% 9.3%
Fixed effect model
1.18 [0.90; 1.46] 100% −−
Random effects model
1.18 [0.90; 1.46] −− 100%
Heterogeneity: I−squared=0%, p=0.6709
−2
−1
0
1
2
% DNA methylation difference

cg22446998

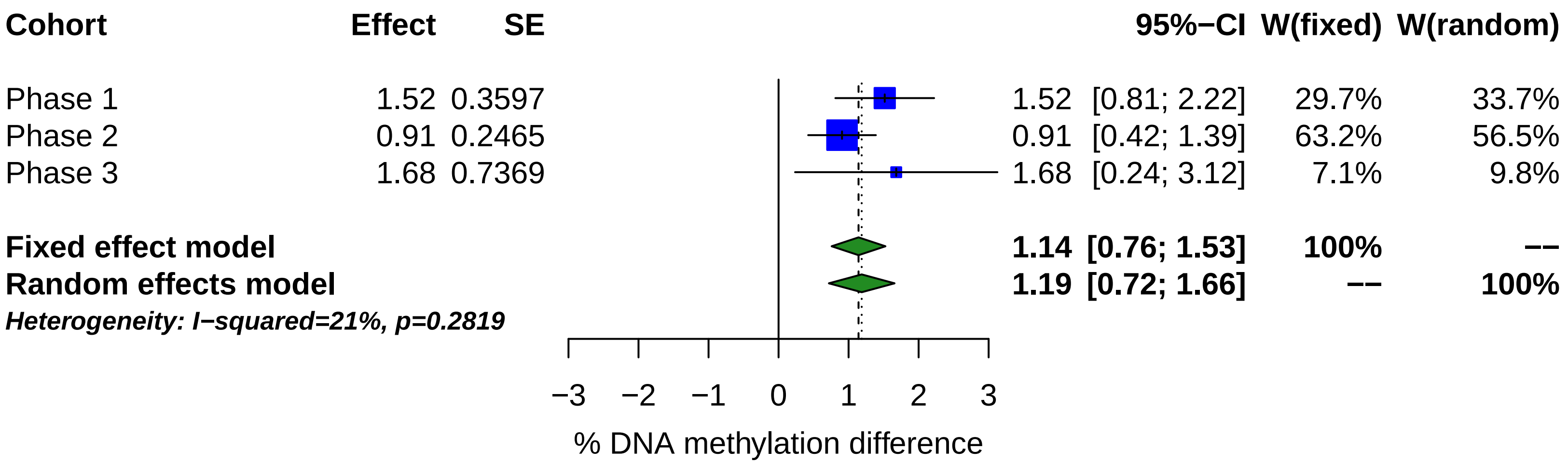
Cohort
Effect
SE
95%−CI
W(fixed)
W(random)
Phase 1 1.52 0.3597 1.52 [0.81; 2.22] 29.7% 33.7%
Phase 2 0.91 0.2465 0.91 [0.42; 1.39] 63.2% 56.5%
Phase 3 1.68 0.7369 1.68 [0.24; 3.12] 7.1% 9.8%
Fixed effect model 1.14 [0.76; 1.53] 100% −−
Random effects model 1.19 [0.72; 1.66] −− 100%
Heterogeneity: I−squared=21%, p=0.2819
−3
−2
−1
0
1
2
3
% DNA methylation difference

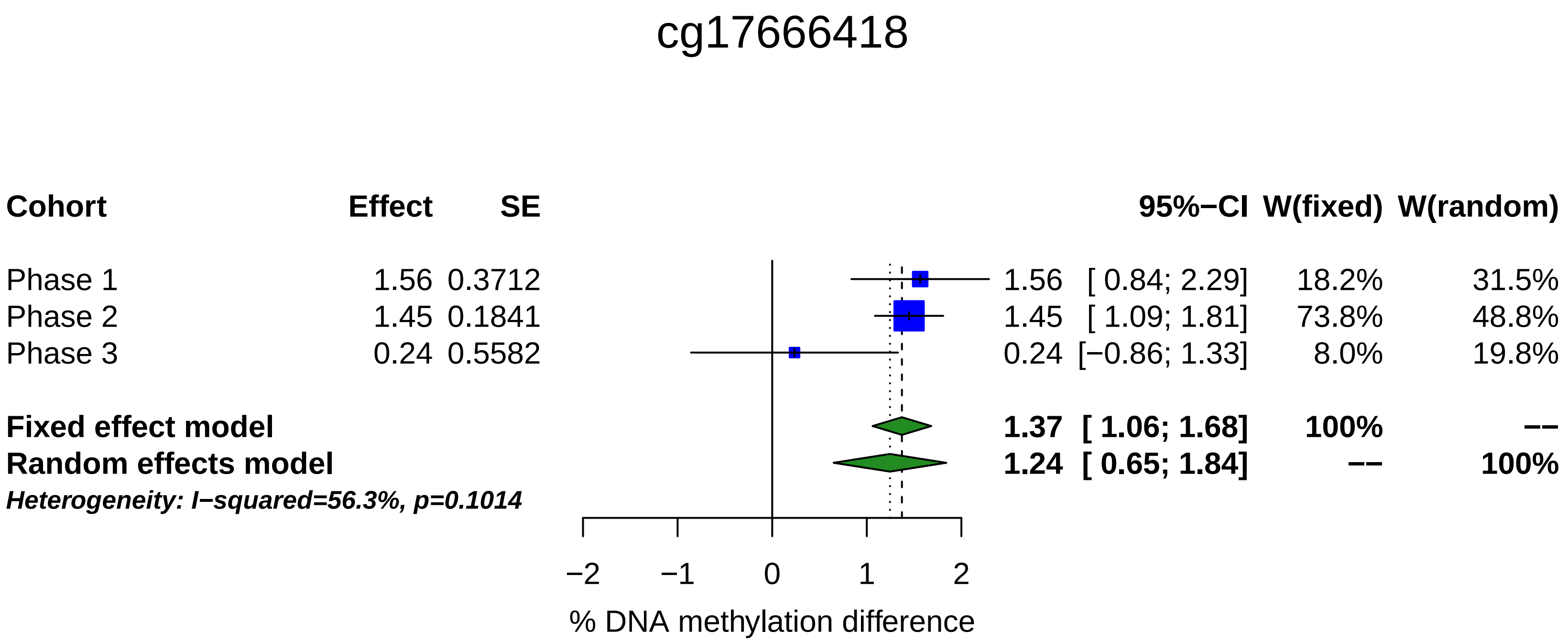
cg17666418
Cohort
Effect
SE
95%−CI
W(fixed)
W(random)
Phase 1 1.56 0.3712 1.56 [0.84; 2.29] 18.2% 31.5%
Phase 2 1.45 0.1841 1.45 [1.09; 1.81] 73.8% 48.8%
Phase 3 0.24 0.5582 0.24 [−0.86; 1.33] 8.0% 19.8%
Fixed effect model 1.37 [1.06; 1.68] 100% −−
Random effects model 1.24 [0.65; 1.84] −− 100%
Heterogeneity: I−squared=56.3%, p=0.1014
−2
−1
0
1
2
% DNA methylation difference

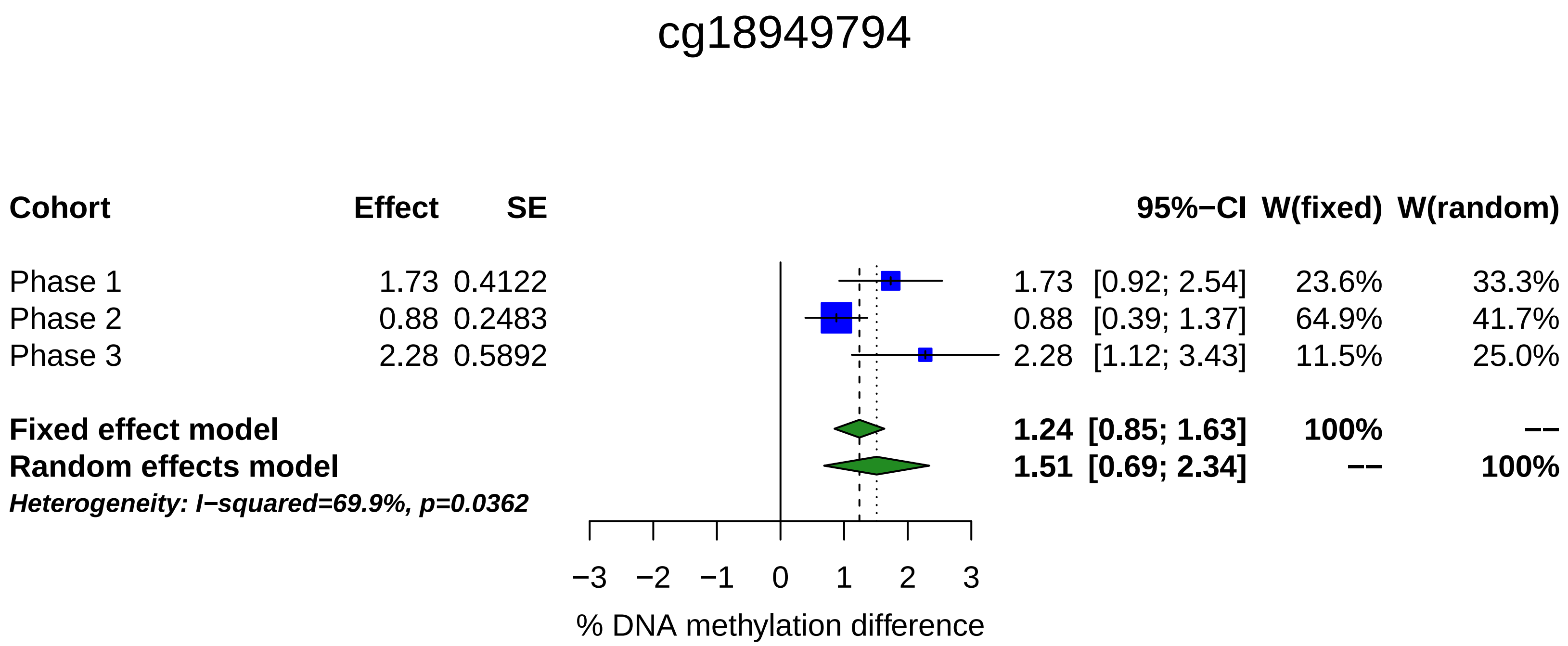
cg18949794
Cohort
Effect
SE
95%−CI
W(fixed)
W(random)
Phase 1 1.73 0.4122 1.73 [0.92; 2.54] 23.6% 33.3%
Phase 2 0.88 0.2483 0.88 [0.39; 1.37] 64.9% 41.7%
Phase 3 2.28 0.5892 2.28 [1.12; 3.43] 11.5% 25.0%
Fixed effect model 1.24 [0.85; 1.63] 100% −−
Random effects model 1.51 [0.69; 2.34] −− 100%
Heterogeneity: I−squared=69.9%, p=0.0362
−3
−2
−1
0
1
2
3
% DNA methylation difference

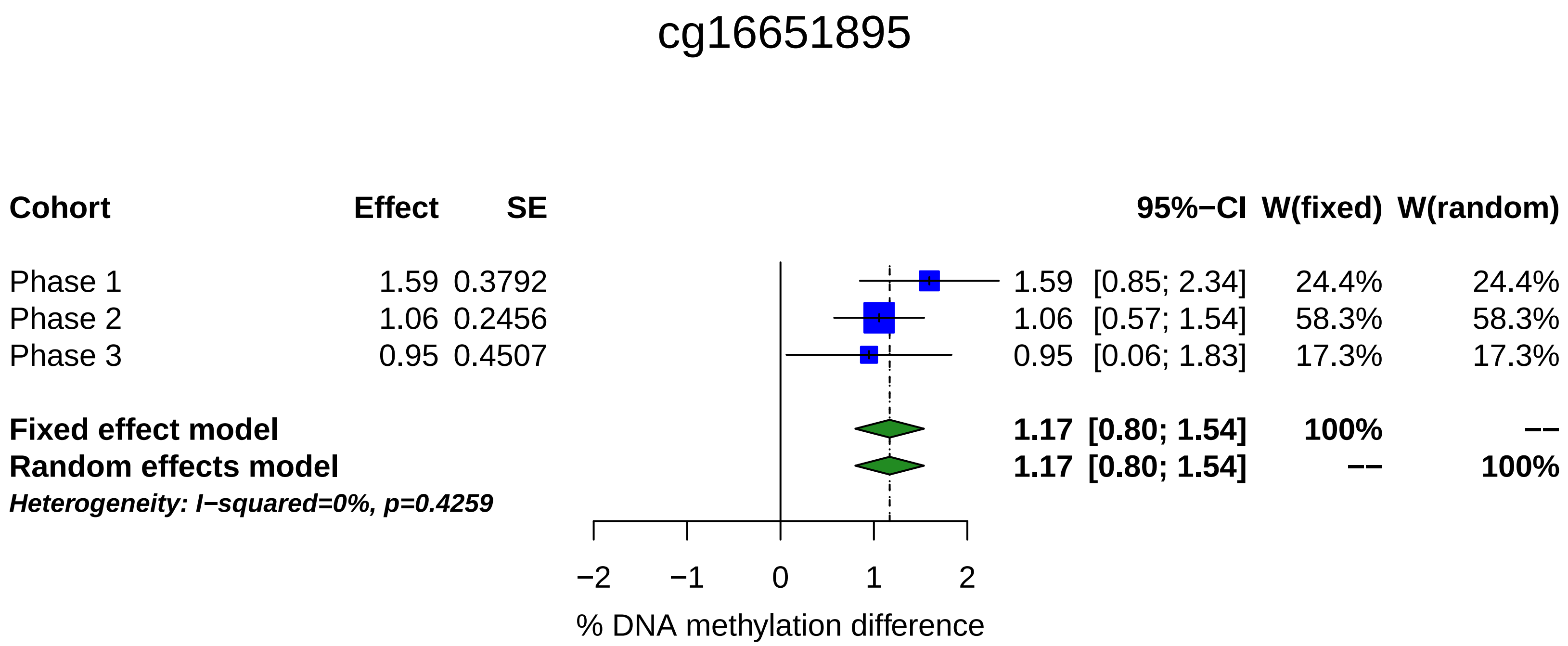
cg16651895
Cohort
Effect
SE
95%−CI
W(fixed)
W(random)
Phase 1 1.59 0.3792 1.59 [0.85; 2.34] 24.4% 24.4%
Phase 2 1.06 0.2456 1.06 [0.57; 1.54] 58.3% 58.3%
Phase 3 0.95 0.4507 0.95 [0.06; 1.83] 17.3% 17.3%
Fixed effect model 1.17 [0.80; 1.54] 100% −−
Random effects model 1.17 [0.80; 1.54] −− 100%
Heterogeneity: I−squared=0%, p=0.4259
−2
−1
0
1
2
% DNA methylation difference

cg25320169

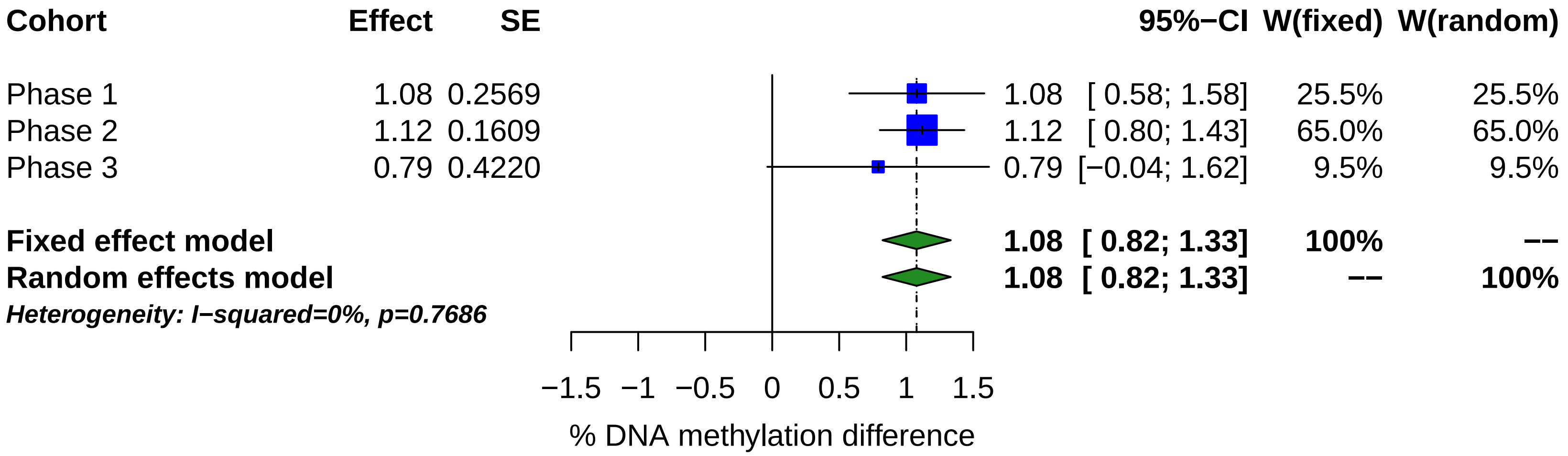
Cohort
Effect
SE
95%−CI
W(fixed)
W(random)
Phase 1
1.08
0.2569
1.08 [0.58; 1.58]
25.5%
25.5%
Phase 2
1.12
0.1609
1.12 [0.80; 1.43]
65.0%
65.0%
Phase 3
0.79
0.4220
0.79 [−0.04; 1.62]
9.5%
9.5%
Fixed effect model
1.08 [0.82; 1.33]
100%
−−
Random effects model
1.08 [0.82; 1.33]
−−
100%
Heterogeneity: I−squared=0%, p=0.7686
−1.5
−1
−0.5
0
0.5
1
1.5
% DNA methylation difference

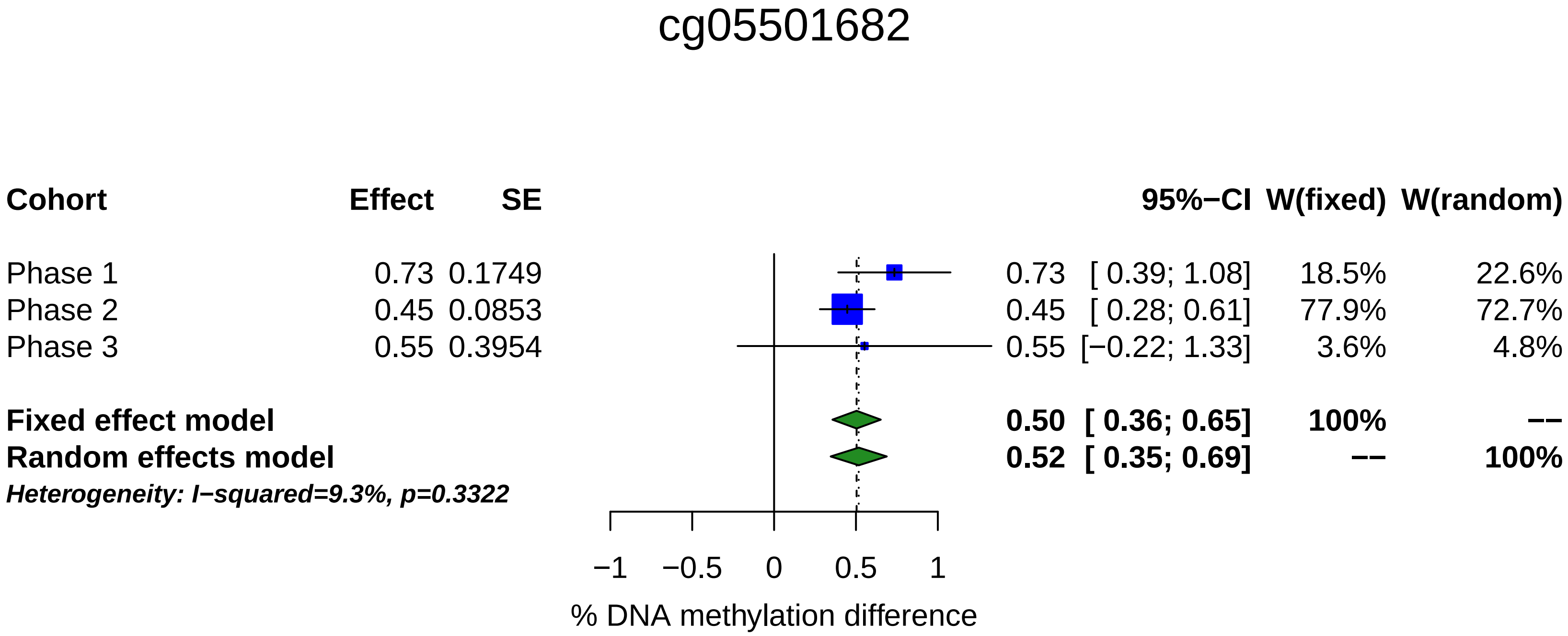
cg05501682
Cohort
Effect
SE
95%−CI
W(fixed)
W(random)
Phase 1
0.73
0.1749
0.73 [0.39; 1.08]
18.5%
22.6%
Phase 2
0.45
0.0853
0.45 [0.28; 0.61]
77.9%
72.7%
Phase 3
0.55
0.3954
0.55 [−0.22; 1.33]
3.6%
4.8%
Fixed effect model
0.50 [0.36; 0.65]
100%
−−
Random effects model
0.52 [0.35; 0.69]
−−
100%
Heterogeneity: I−squared=9.3%, p=0.3322
−1
−0.5
0
0.5
1
% DNA methylation difference

cg00676085

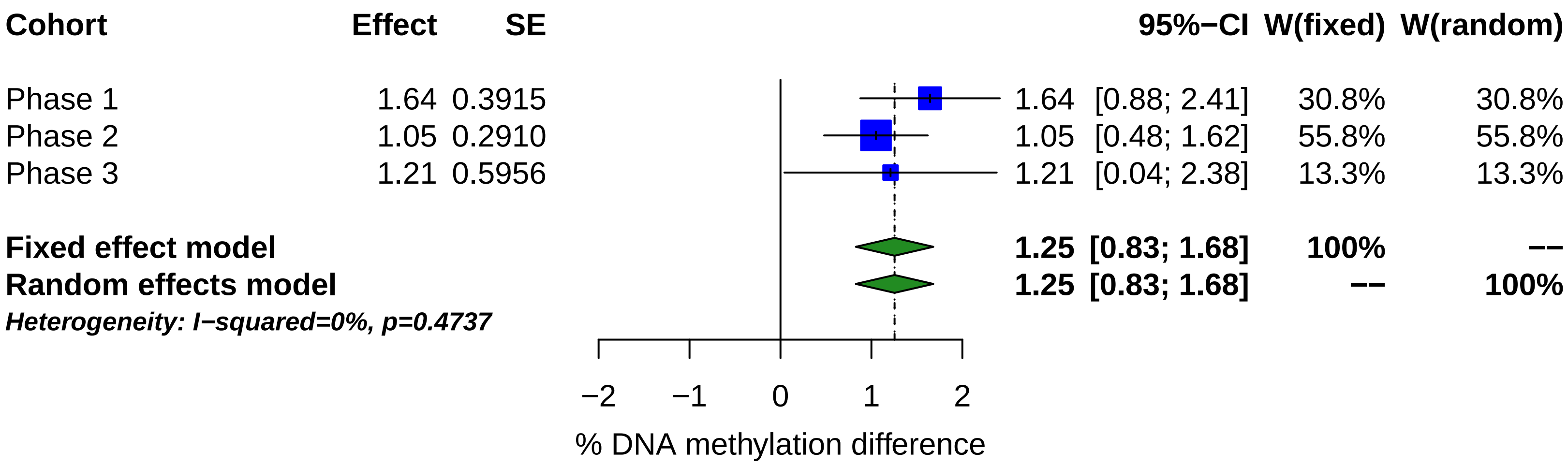
Cohort
Effect
SE
95%−CI
W(fixed)
W(random)
Phase 1 1.64 0.3915 1.64 [0.88; 2.41] 30.8% 30.8%
Phase 2 1.05 0.2910 1.05 [0.48; 1.62] 55.8% 55.8%
Phase 3 1.21 0.5956 1.21 [0.04; 2.38] 13.3% 13.3%
Fixed effect model 1.25 [0.83; 1.68] 100% −−
Random effects model 1.25 [0.83; 1.68] −− 100%
Heterogeneity: I−squared=0%, p=0.4737
−2
−1
0
1
2
% DNA methylation difference

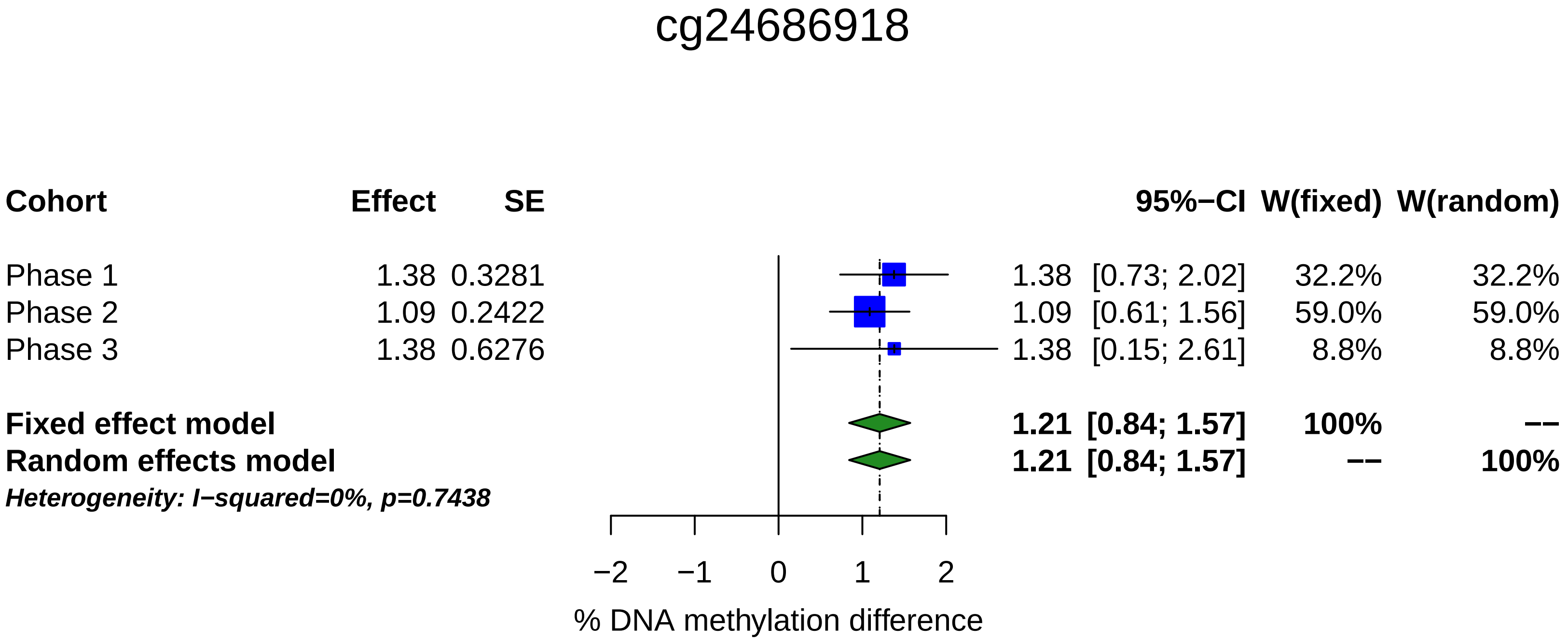
cg24686918
Cohort
Effect
SE
95%−CI
W(fixed)
W(random)
Phase 1 1.38 0.3281 1.38 [0.73; 2.02] 32.2% 32.2%
Phase 2 1.09 0.2422 1.09 [0.61; 1.56] 59.0% 59.0%
Phase 3 1.38 0.6276 1.38 [0.15; 2.61] 8.8% 8.8%
Fixed effect model 1.21 [0.84; 1.57] 100% −−
Random effects model 1.21 [0.84; 1.57] −− 100%
Heterogeneity: I−squared=0%, p=0.7438
−2
−1
0
1
2
% DNA methylation difference

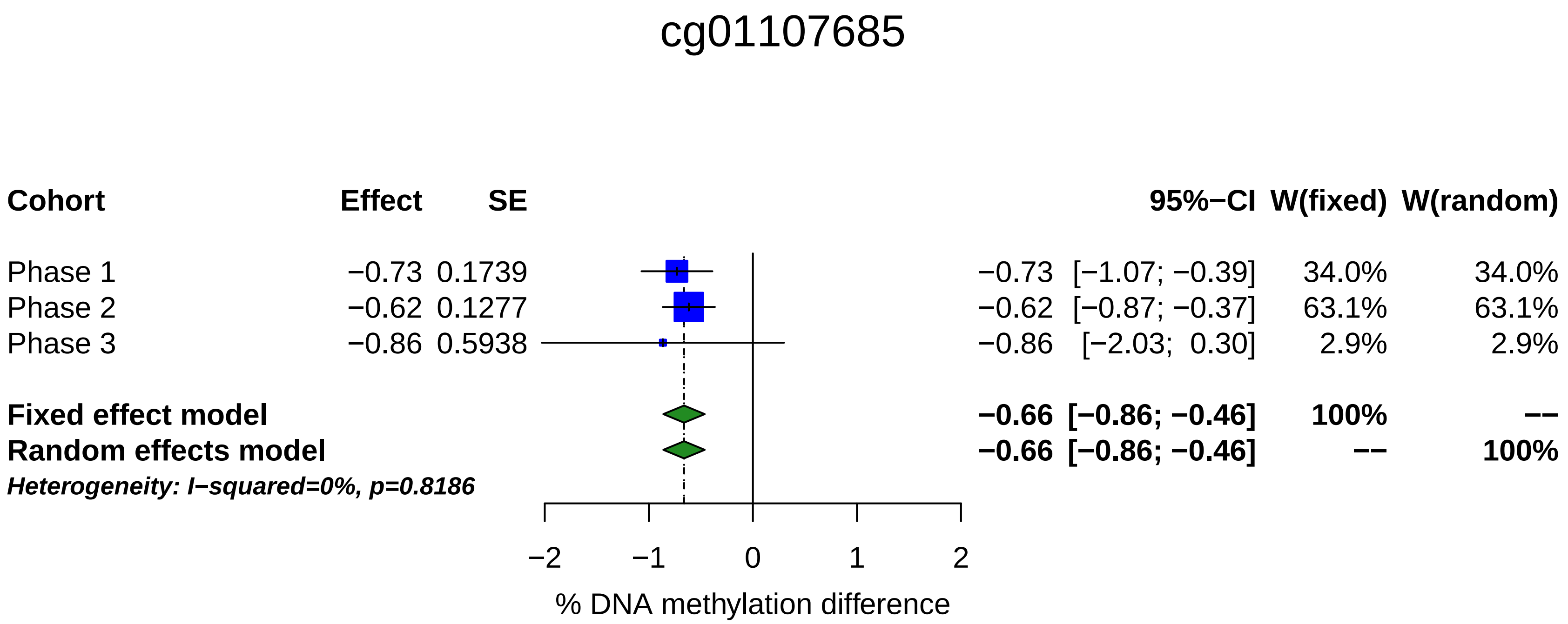
cg01107685
Cohort
Effect
SE
95%−CI
W(fixed)
W(random)
Phase 1
−0.73
0.1739
−0.73 [−1.07; −0.39]
34.0%
34.0%
Phase 2
−0.62
0.1277
−0.62 [−0.87; −0.37]
63.1%
63.1%
Phase 3
−0.86
0.5938
−0.86 [−2.03; 0.30]
2.9%
2.9%
Fixed effect model
−0.66 [−0.86; −0.46]
100%
−−
Random effects model
−0.66 [−0.86; −0.46]
−−
100%
Heterogeneity: I−squared=0%, p=0.8186
−2
−1
0
1
2
% DNA methylation difference

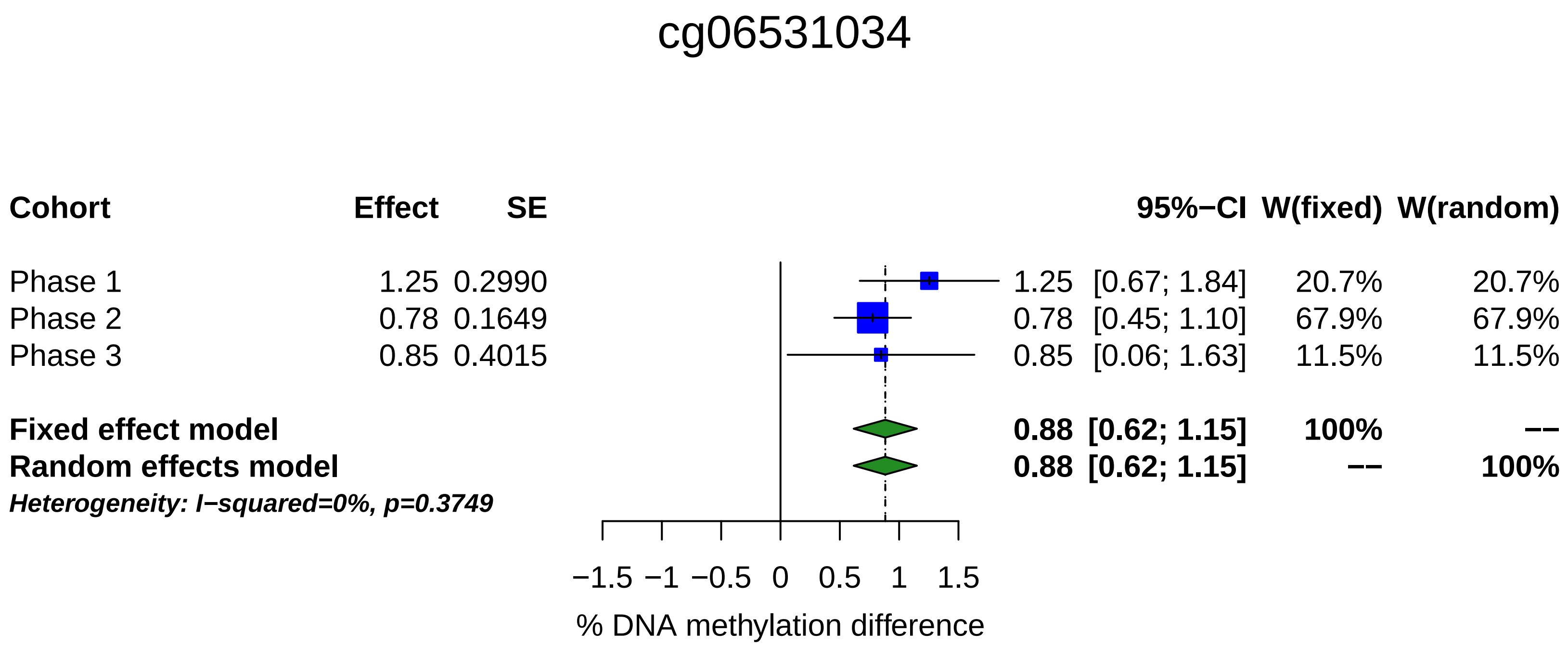
cg06531034
Cohort
Effect
SE
95%−CI
W(fixed)
W(random)
Phase 1 1.25 0.2990 1.25 [0.67; 1.84] 20.7% 20.7%
Phase 2 0.78 0.1649 0.78 [0.45; 1.10] 67.9% 67.9%
Phase 3 0.85 0.4015 0.85 [0.06; 1.63] 11.5% 11.5%
Fixed effect model 0.88 [0.62; 1.15] 100% −−
Random effects model 0.88 [0.62; 1.15] −− 100%
Heterogeneity: I−squared=0%, p=0.3749
−1.5
−1
−0.5
0
0.5
1
1.5
% DNA methylation difference

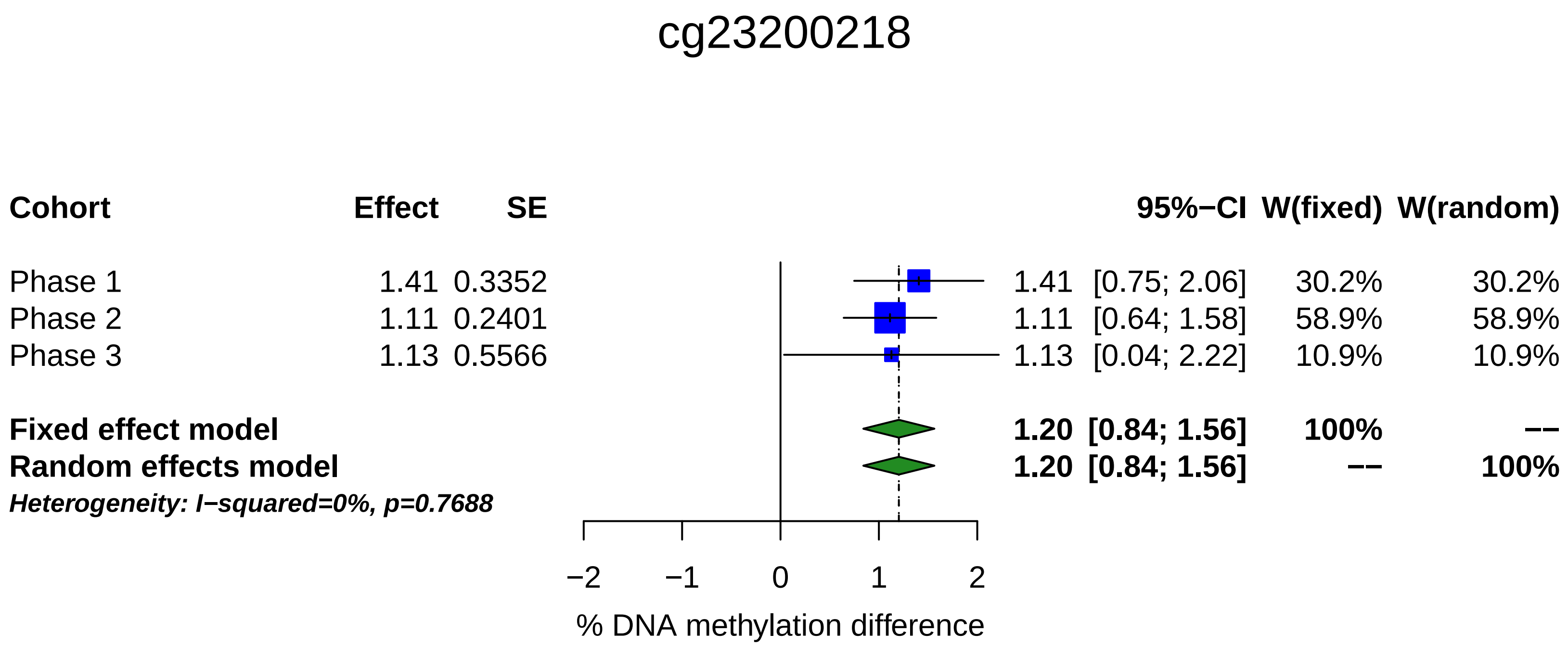
cg23200218
Cohort
Effect
SE
95%−CI
W(fixed)
W(random)
Phase 1 1.41 0.3352 1.41 [0.75; 2.06] 30.2% 30.2%
Phase 2 1.11 0.2401 1.11 [0.64; 1.58] 58.9% 58.9%
Phase 3 1.13 0.5566 1.13 [0.04; 2.22] 10.9% 10.9%
Fixed effect model 1.20 [0.84; 1.56] 100% −−
Random effects model 1.20 [0.84; 1.56] −− 100%
Heterogeneity: I−squared=0%, p=0.7688
−2
−1
0
1
2
% DNA methylation difference

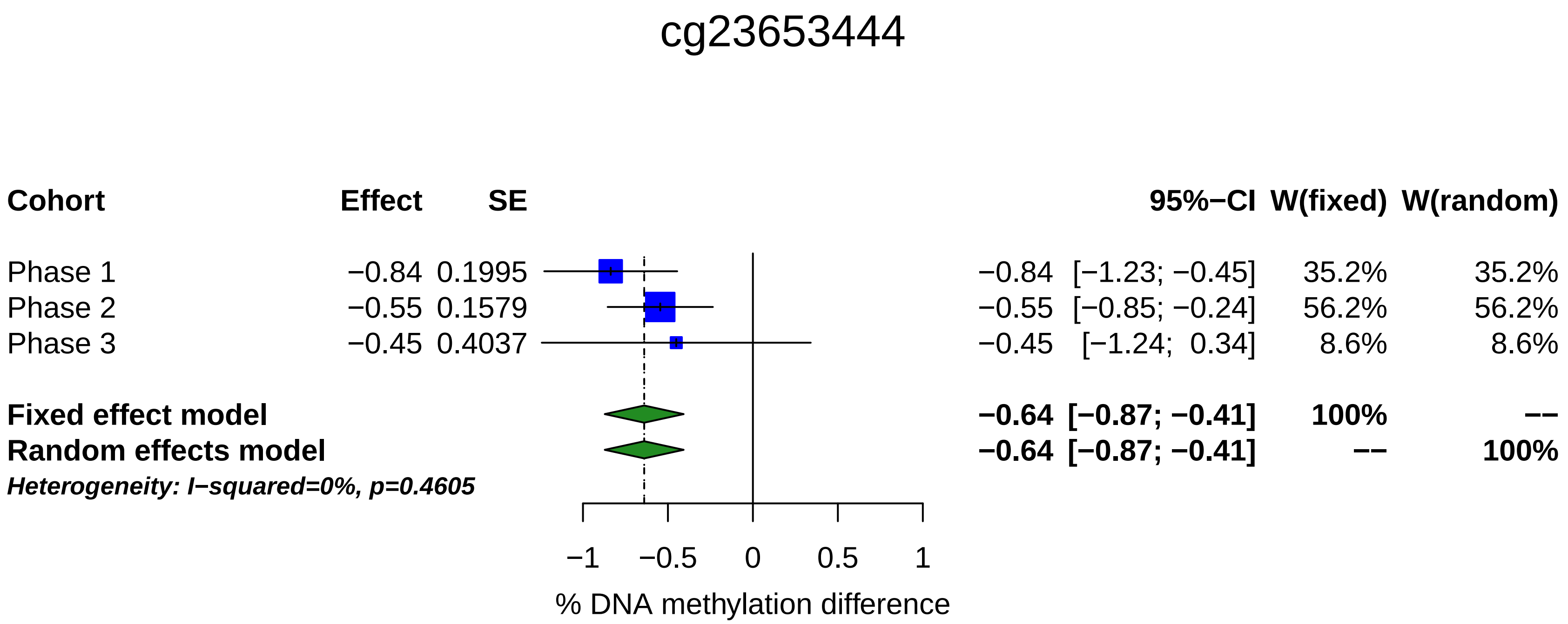

cg23653444
Cohort
Effect
SE
95%−CI
W(fixed)
W(random)
Phase 1 −0.84 0.1995 −0.84 [−1.23; −0.45] 35.2% 35.2%
Phase 2 −0.55 0.1579 −0.55 [−0.85; −0.24] 56.2% 56.2%
Phase 3 −0.45 0.4037 −0.45 [−1.24; 0.34] 8.6% 8.6%
Fixed effect model −0.64 [−0.87; −0.41] 100% −−
Random effects model −0.64 [−0.87; −0.41] −− 100%
Heterogeneity: I−squared=0%, p=0.4605
−1
−0.5
0
0.5
1
% DNA methylation difference

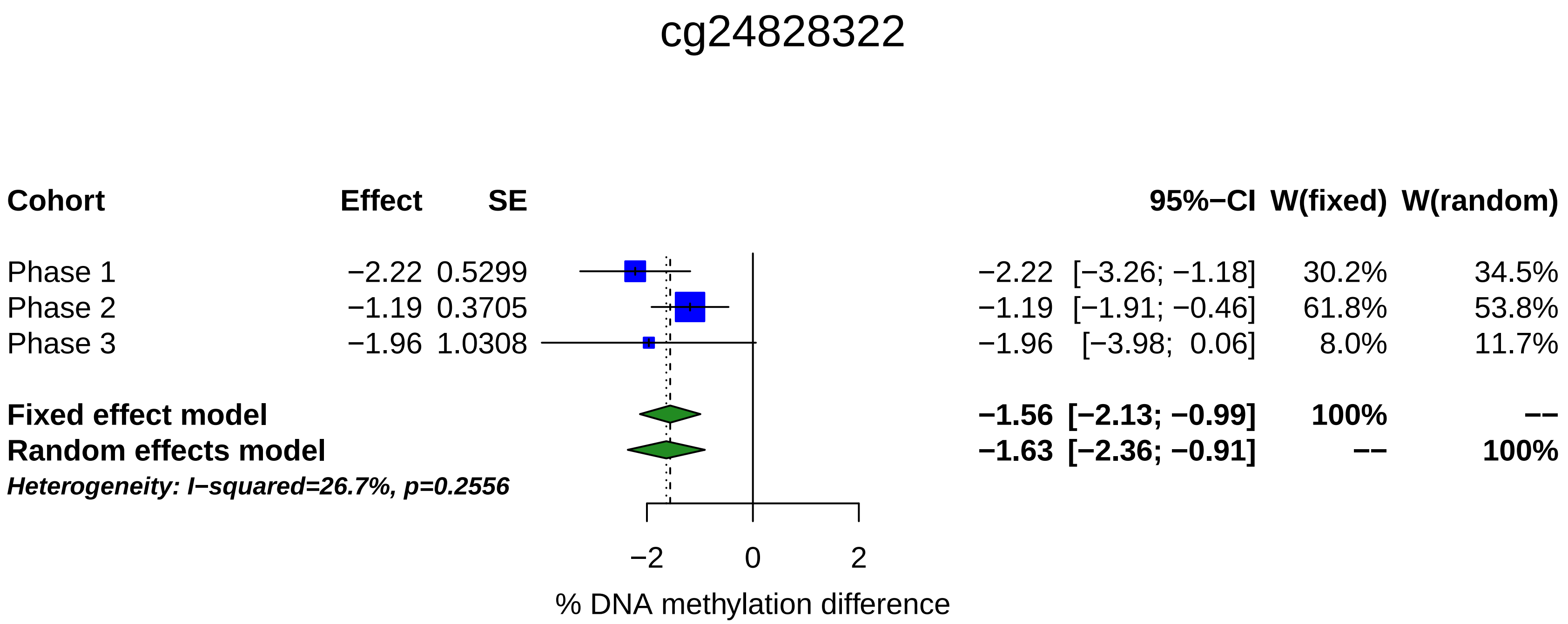
cg24828322
Cohort
Effect
SE
95%−CI
W(fixed)
W(random)
Phase 1 −2.22 0.5299 −2.22 [−3.26; −1.18] 30.2% 34.5%
Phase 2 −1.19 0.3705 −1.19 [−1.91; −0.46] 61.8% 53.8%
Phase 3 −1.96 1.0308 −1.96 [−3.98; 0.06] 8.0% 11.7%
Fixed effect model −1.56 [−2.13; −0.99] 100% −−
Random effects model −1.63 [−2.36; −0.91] −− 100%
Heterogeneity: I−squared=26.7%, p=0.2556
−2
0
2
% DNA methylation difference

cg20931134

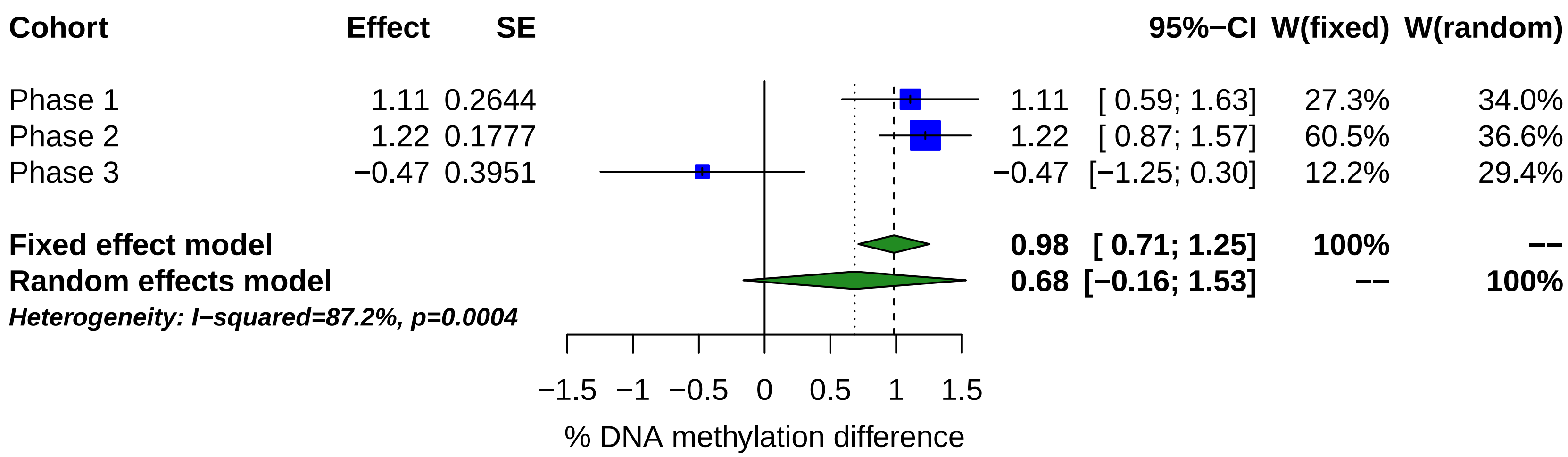

Cohort
Effect
SE
95%−CI
W(fixed)
W(random)
Phase 1 1.11 0.2644 1.11 [0.59; 1.63] 27.3% 34.0%
Phase 2 1.22 0.1777 1.22 [0.87; 1.57] 60.5% 36.6%
Phase 3 −0.47 0.3951 −0.47 [−1.25; 0.30] 12.2% 29.4%
Fixed effect model 0.98 [0.71; 1.25] 100% −−
Random effects model 0.68 [−0.16; 1.53] −− 100%
Heterogeneity: I−squared=87.2%, p=0.0004
−1.5
−1
−0.5
0
0.5
1
1.5
% DNA methylation difference

cg16780562

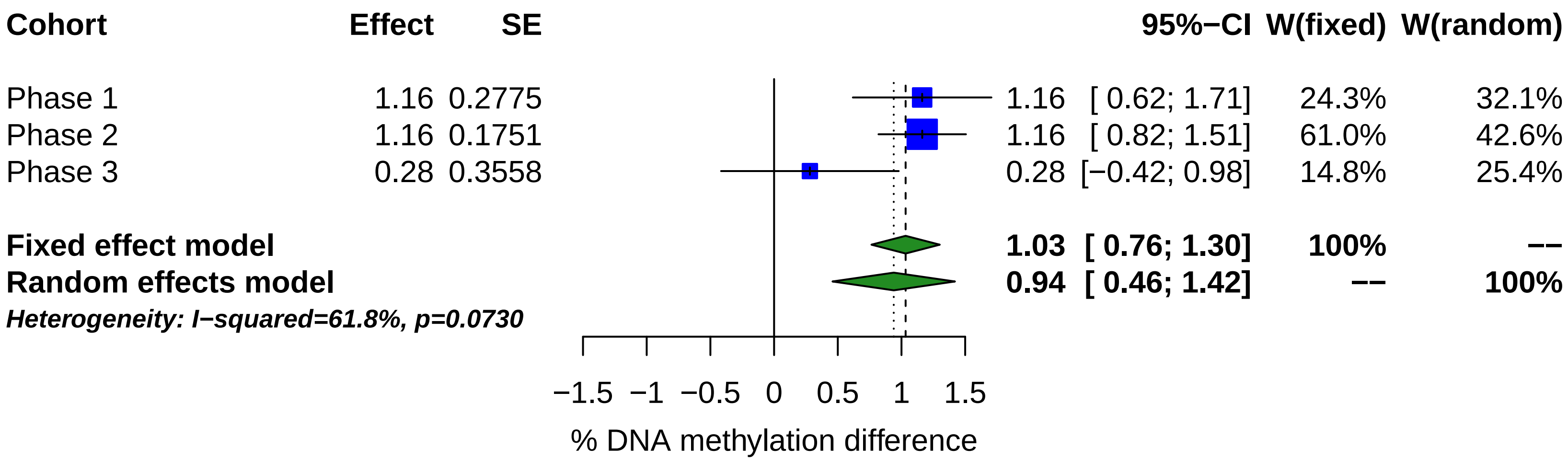

Cohort
Effect
SE
95%−CI
W(fixed)
W(random)
Phase 1
1.16
0.2775
1.16
[0.62; 1.71]
24.3%
32.1%
Phase 2
1.16
0.1751
1.16
[0.82; 1.51]
61.0%
42.6%
Phase 3
0.28
0.3558
0.28
[−0.42; 0.98]
14.8%
25.4%
Fixed effect model
1.03
[0.76; 1.30]
100%
−−
Random effects model
0.94
[0.46; 1.42]
−−
100%
Heterogeneity: I−squared=61.8%, p=0.0730
−1.5
−1
−0.5
0
0.5
1
1.5
% DNA methylation difference

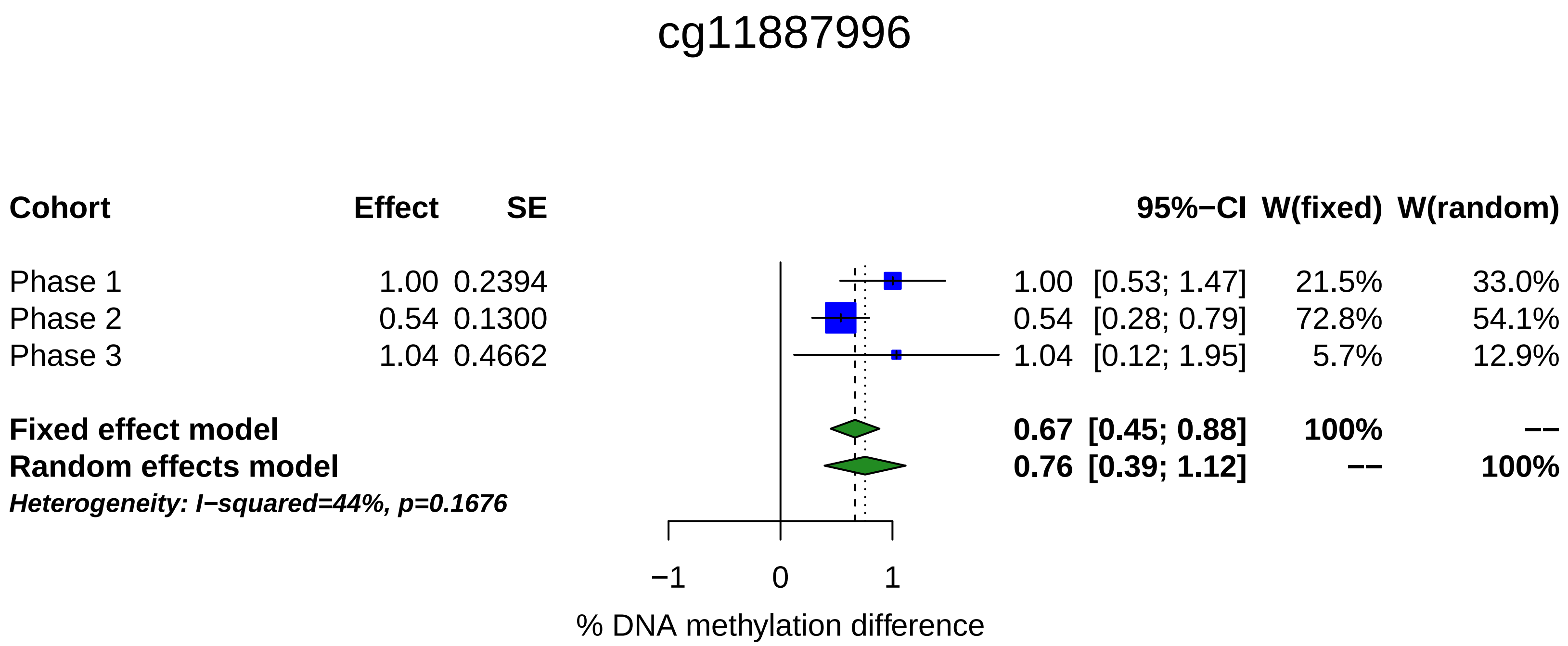
cg11887996
Cohort
Effect
SE
95%−CI
W(fixed)
W(random)
Phase 1 1.00 0.2394 1.00 [0.53; 1.47] 21.5% 33.0%
Phase 2 0.54 0.1300 0.54 [0.28; 0.79] 72.8% 54.1%
Phase 3 1.04 0.4662 1.04 [0.12; 1.95] 5.7% 12.9%
Fixed effect model 0.67 [0.45; 0.88] 100% −−
Random effects model 0.76 [0.39; 1.12] −− 100%
Heterogeneity: I−squared=44%, p=0.1676
−1
0
1
% DNA methylation difference

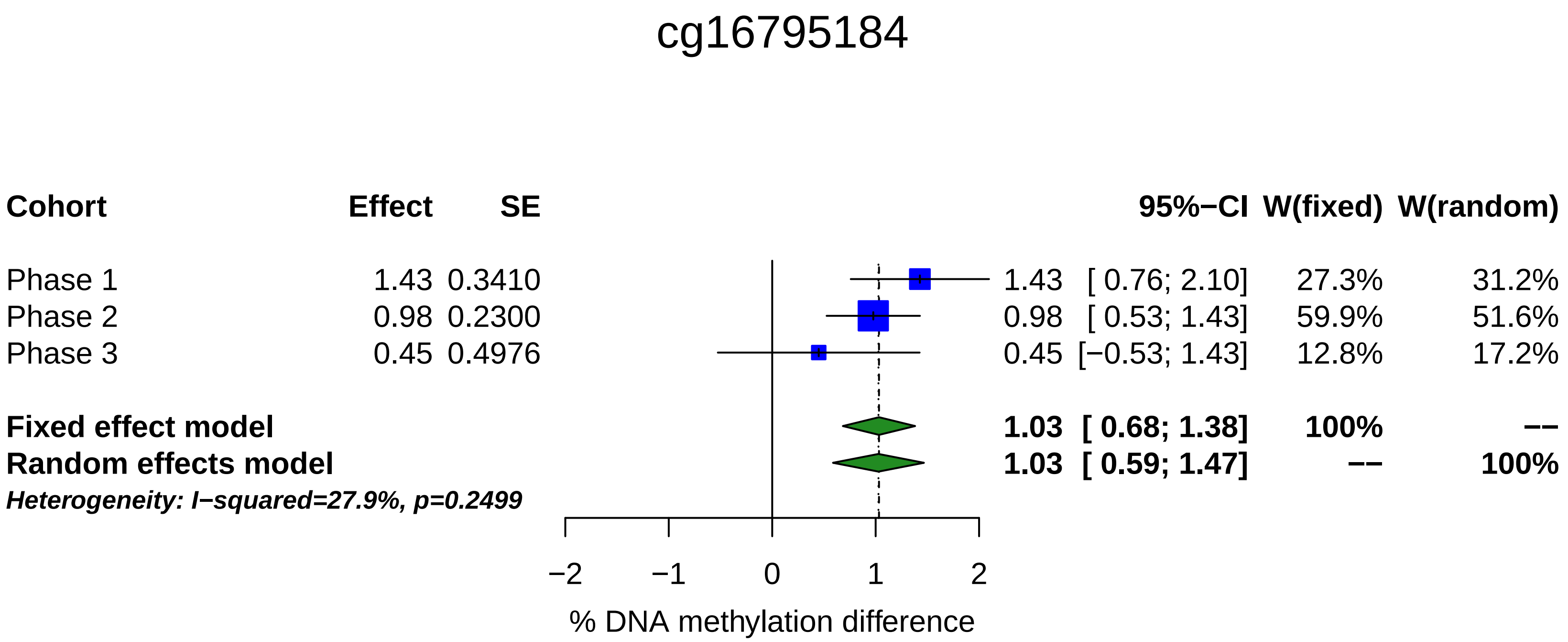
cg16795184
Cohort
Effect
SE
95%−CI
W(fixed)
W(random)
Phase 1
1.43
0.3410
1.43 [0.76; 2.10]
27.3%
31.2%
Phase 2
0.98
0.2300
0.98 [0.53; 1.43]
59.9%
51.6%
Phase 3
0.45
0.4976
0.45 [−0.53; 1.43]
12.8%
17.2%
Fixed effect model
1.03 [0.68; 1.38]
100%
−−
Random effects model
1.03 [0.59; 1.47]
−−
100%
Heterogeneity: I−squared=27.9%, p=0.2499
−2
−1
0
1
2
% DNA methylation difference

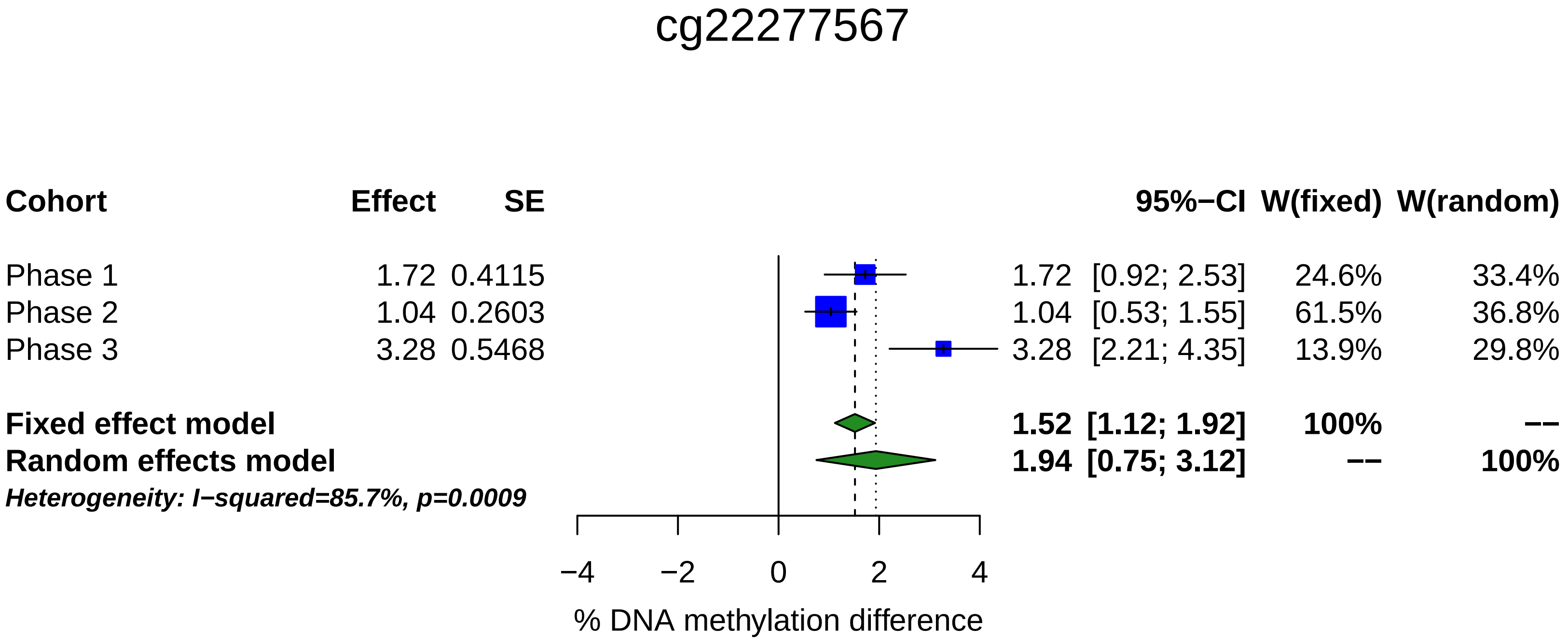
cg22277567
Cohort
Effect
SE
95%−CI
W(fixed)
W(random)
Phase 1 1.72 0.4115 1.72 [0.92; 2.53] 24.6% 33.4%
Phase 2 1.04 0.2603 1.04 [0.53; 1.55] 61.5% 36.8%
Phase 3 3.28 0.5468 3.28 [2.21; 4.35] 13.9% 29.8%
Fixed effect model 1.52 [1.12; 1.92] 100% −−
Random effects model 1.94 [0.75; 3.12] −− 100%
Heterogeneity: I−squared=85.7%, p=0.0009
−4
−2
0
2
4
% DNA methylation difference

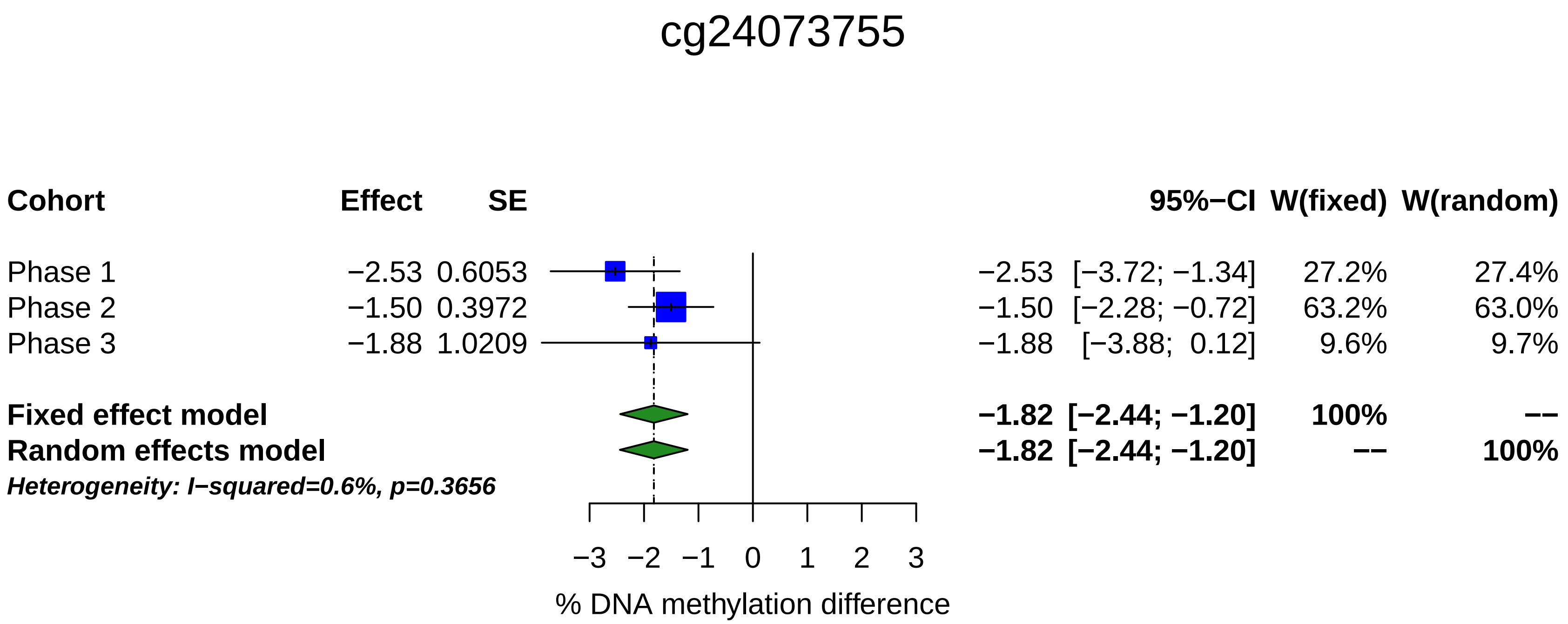
cg24073755
Cohort
Effect
SE
95%−CI
W(fixed)
W(random)
Phase 1 −2.53 0.6053 −2.53 [−3.72; −1.34] 27.2% 27.4%
Phase 2 −1.50 0.3972 −1.50 [−2.28; −0.72] 63.2% 63.0%
Phase 3 −1.88 1.0209 −1.88 [−3.88; 0.12] 9.6% 9.7%
Fixed effect model −1.82 [−2.44; −1.20] 100% −−
Random effects model −1.82 [−2.44; −1.20] −− 100%
Heterogeneity: I−squared=0.6%, p=0.3656
−3 −2 −1 0 1 2 3
% DNA methylation difference

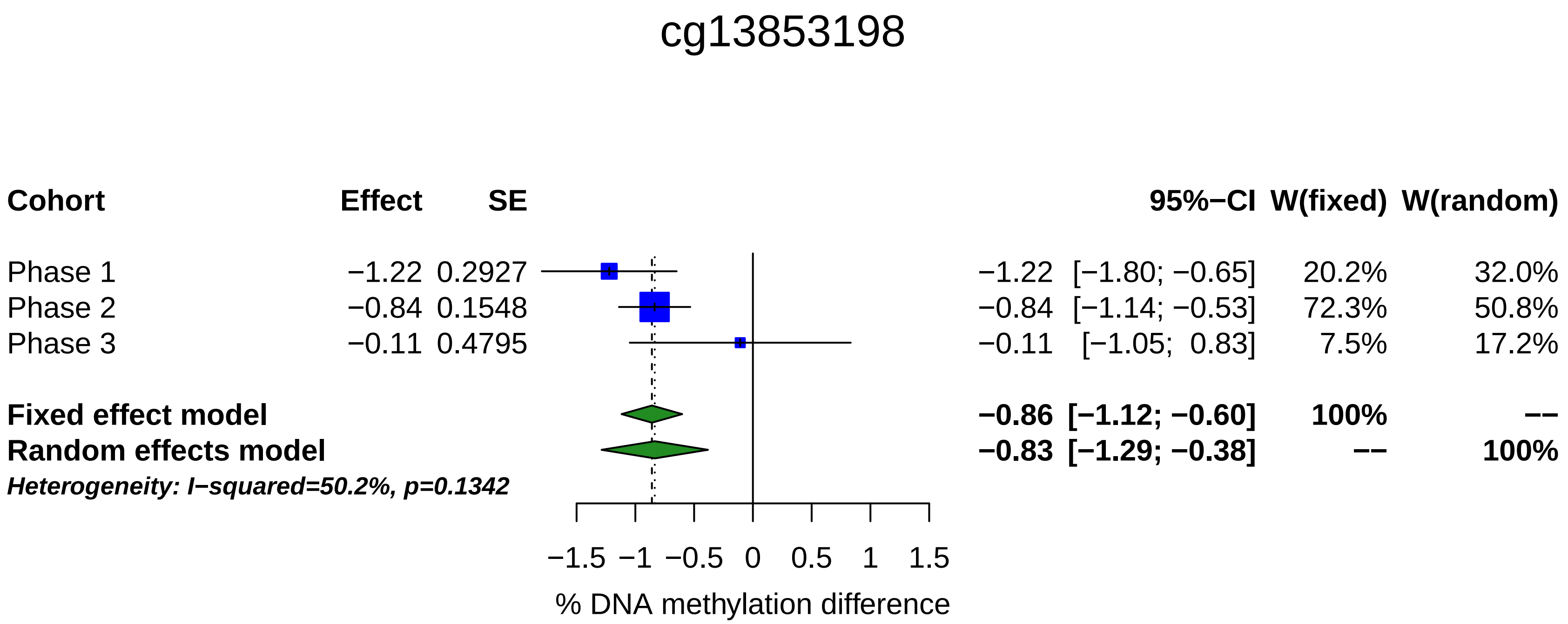
cg13853198
Cohort
Effect
SE
95%−CI
W(fixed)
W(random)
Phase 1 −1.22 0.2927 −1.22 [−1.80; −0.65] 20.2% 32.0%
Phase 2 −0.84 0.1548 −0.84 [−1.14; −0.53] 72.3% 50.8%
Phase 3 −0.11 0.4795 −0.11 [−1.05; 0.83] 7.5% 17.2%
Fixed effect model −0.86 [−1.12; −0.60] 100% −−
Random effects model −0.83 [−1.29; −0.38] −− 100%
Heterogeneity: I−squared=50.2%, p=0.1342
−1.5
−1
−0.5
0
0.5
1
1.5
% DNA methylation difference

cg12124767

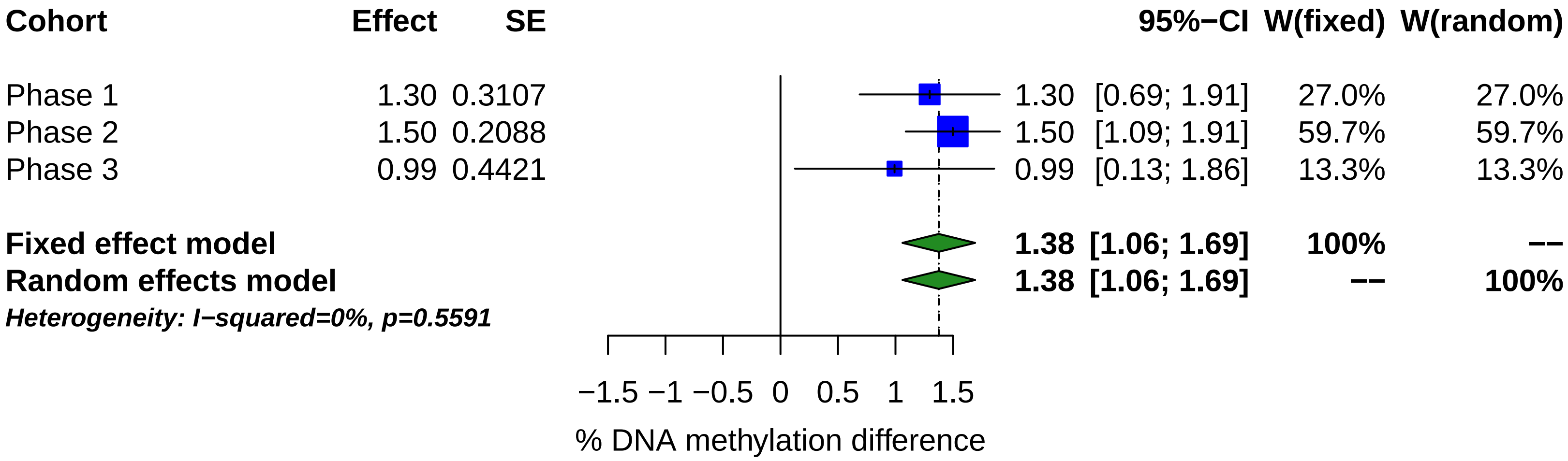

Cohort
Effect
SE
95%−CI
W(fixed)
W(random)
Phase 1 1.30 0.3107 1.30 [0.69; 1.91] 27.0% 27.0%
Phase 2 1.50 0.2088 1.50 [1.09; 1.91] 59.7% 59.7%
Phase 3 0.99 0.4421 0.99 [0.13; 1.86] 13.3% 13.3%
Fixed effect model 1.38 [1.06; 1.69] 100% −−
Random effects model 1.38 [1.06; 1.69] −− 100%
Heterogeneity: I−squared=0%, p=0.5591
−1.5 −1 −0.5 0 0.5 1 1.5
% DNA methylation difference

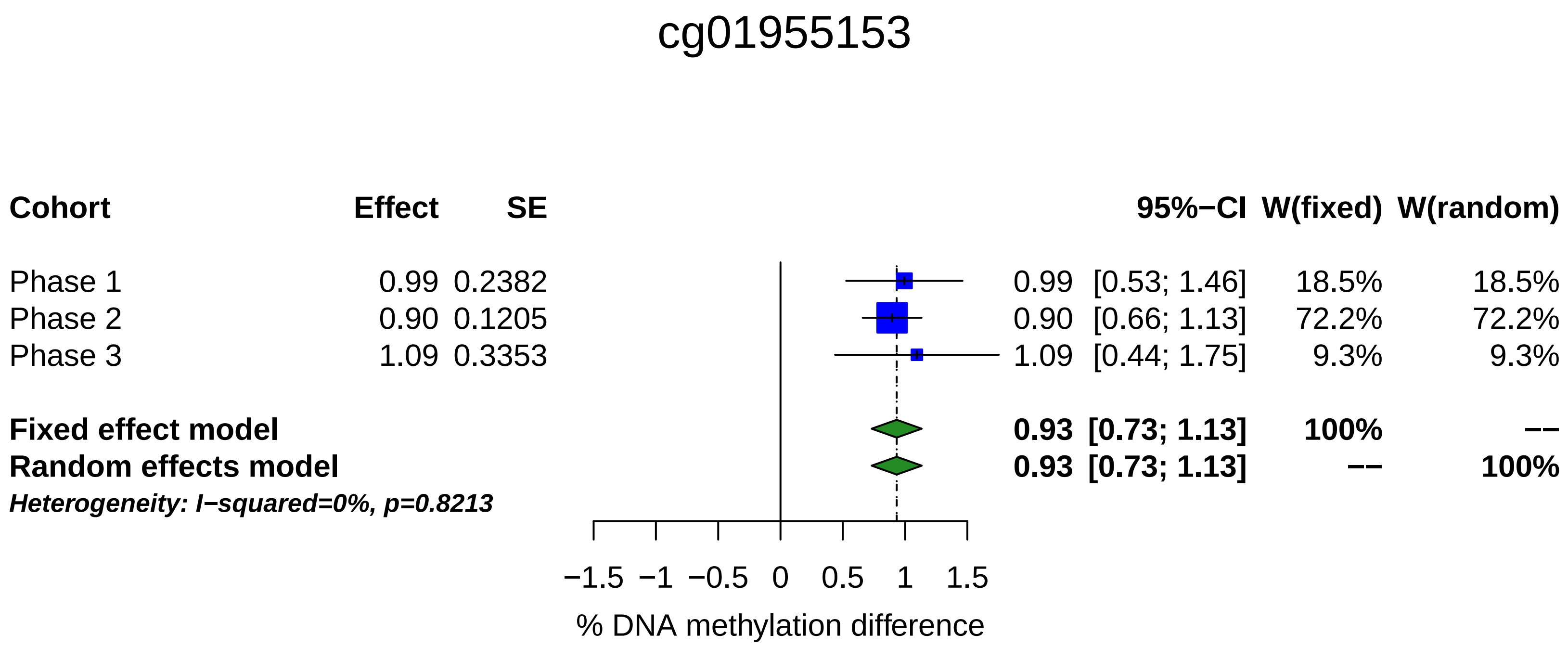

cg01955153
Cohort
Effect
SE
95%−CI
W(fixed)
W(random)
Phase 1 0.99 0.2382 0.99 [0.53; 1.46] 18.5% 18.5%
Phase 2 0.90 0.1205 0.90 [0.66; 1.13] 72.2% 72.2%
Phase 3 1.09 0.3353 1.09 [0.44; 1.75] 9.3% 9.3%
Fixed effect model 0.93 [0.73; 1.13] 100% −−
Random effects model 0.93 [0.73; 1.13] −− 100%
Heterogeneity: I−squared=0%, p=0.8213
−1.5 −1 −0.5 0 0.5 1 1.5
% DNA methylation difference

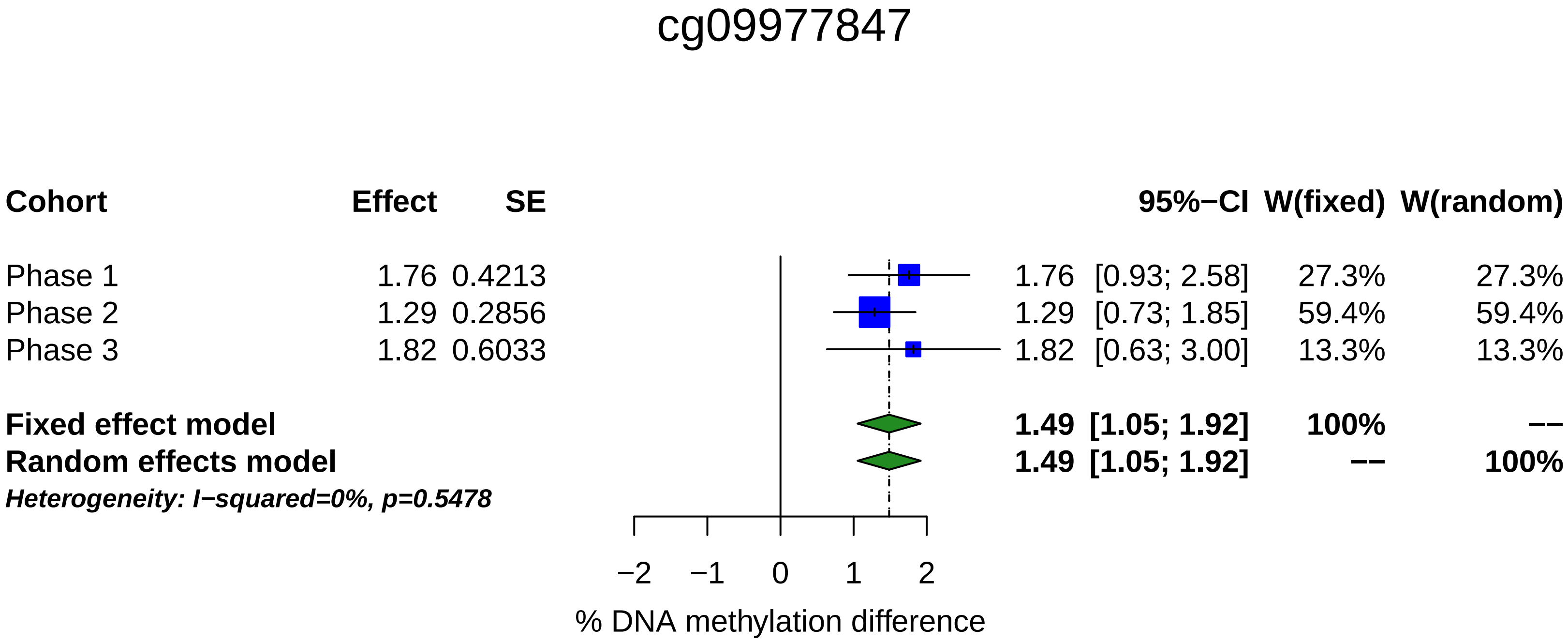
cg09977847
Cohort
Effect
SE
95%−CI
W(fixed)
W(random)
Phase 1 1.76 0.4213 1.76 [0.93; 2.58] 27.3% 27.3%
Phase 2 1.29 0.2856 1.29 [0.73; 1.85] 59.4% 59.4%
Phase 3 1.82 0.6033 1.82 [0.63; 3.00] 13.3% 13.3%
Fixed effect model 1.49 [1.05; 1.92] 100% −−
Random effects model 1.49 [1.05; 1.92] −− 100%
Heterogeneity: I−squared=0%, p=0.5478
−2
−1
0
1
2
% DNA methylation difference

cg14575983

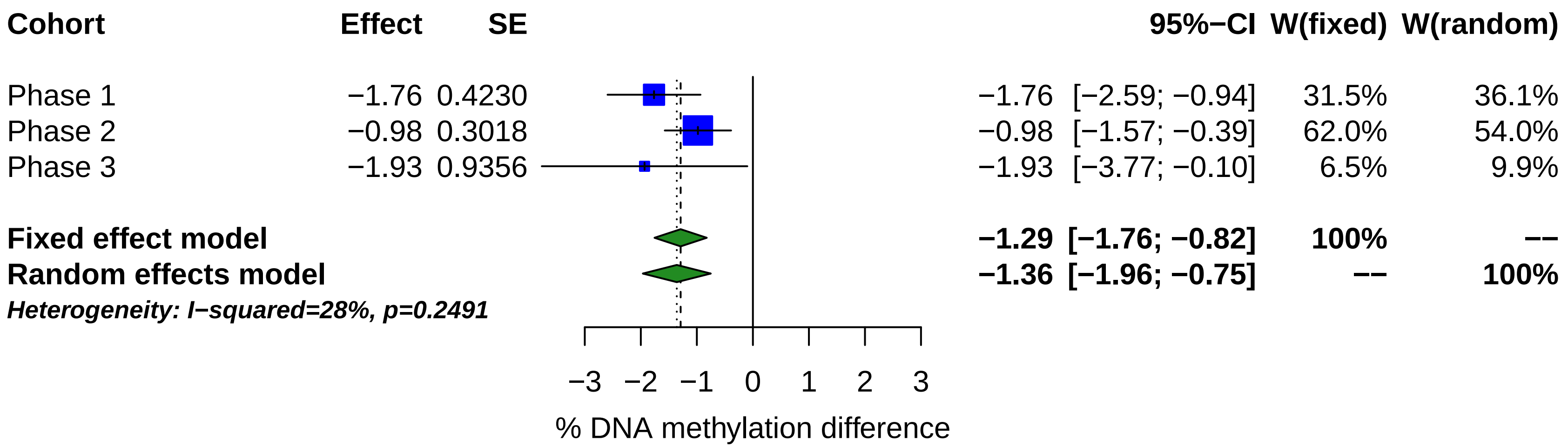
Cohort
Effect
SE
95%−CI
W(fixed)
W(random)
Phase 1 −1.76 0.4230 −1.76 [−2.59; −0.94] 31.5% 36.1%
Phase 2 −0.98 0.3018 −0.98 [−1.57; −0.39] 62.0% 54.0%
Phase 3 −1.93 0.9356 −1.93 [−3.77; −0.10] 6.5% 9.9%
Fixed effect model −1.29 [−1.76; −0.82] 100% −−
Random effects model −1.36 [−1.96; −0.75] −− 100%
Heterogeneity: I−squared=28%, p=0.2491
−3
−2
−1
0
1
2
3
% DNA methylation difference

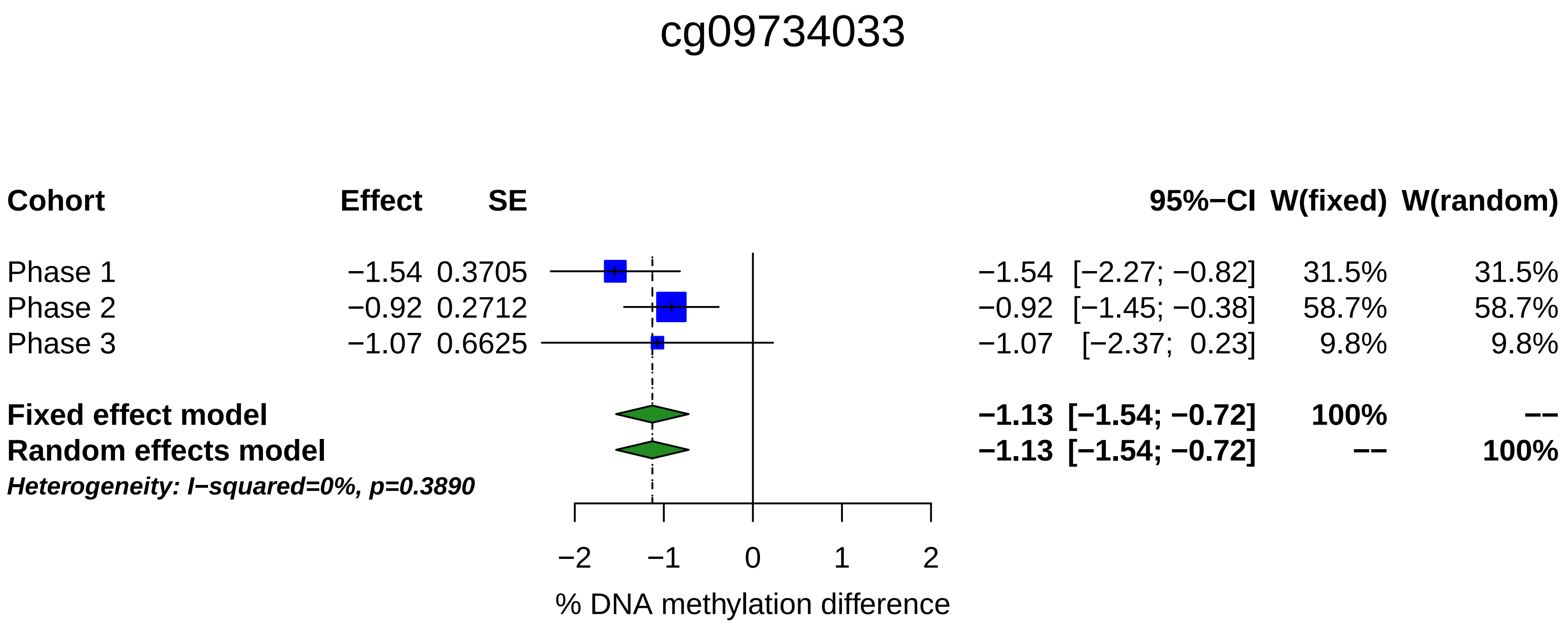
cg09734033
Cohort
Effect
SE
95%−CI
W(fixed)
W(random)
Phase 1 −1.54 0.3705 −1.54 [−2.27; −0.82] 31.5% 31.5%
Phase 2 −0.92 0.2712 −0.92 [−1.45; −0.38] 58.7% 58.7%
Phase 3 −1.07 0.6625 −1.07 [−2.37; 0.23] 9.8% 9.8%
Fixed effect model −1.13 [−1.54; −0.72] 100% −−
Random effects model −1.13 [−1.54; −0.72] −− 100%
Heterogeneity: I−squared=0%, p=0.3890
−2
−1
0
1
2
% DNA methylation difference

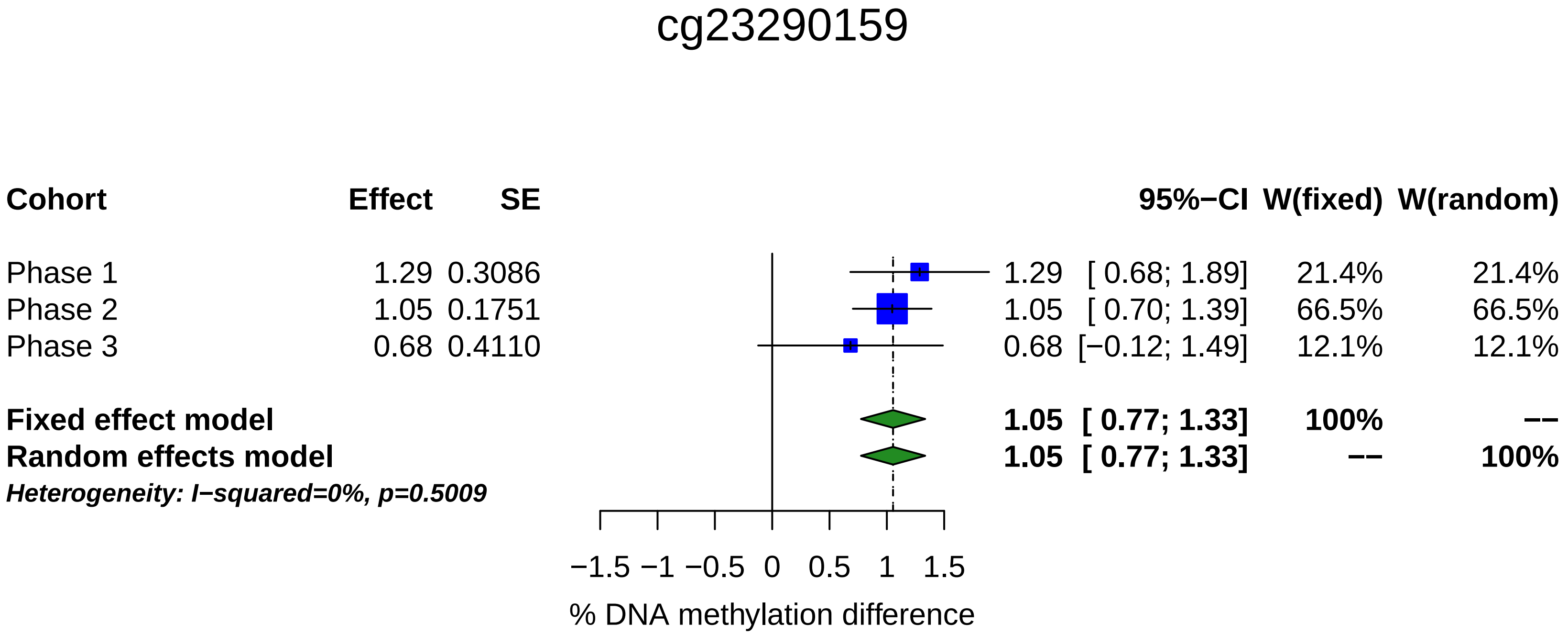
cg23290159
Cohort
Effect
SE
95%−CI
W(fixed)
W(random)
Phase 1 1.29 0.3086 1.29 [0.68; 1.89] 21.4% 21.4%
Phase 2 1.05 0.1751 1.05 [0.70; 1.39] 66.5% 66.5%
Phase 3 0.68 0.4110 0.68 [−0.12; 1.49] 12.1% 12.1%
Fixed effect model 1.05 [0.77; 1.33] 100% −−
Random effects model 1.05 [0.77; 1.33] −− 100%
Heterogeneity: I−squared=0%, p=0.5009
−1.5
−1
−0.5
0
0.5
1
1.5
% DNA methylation difference

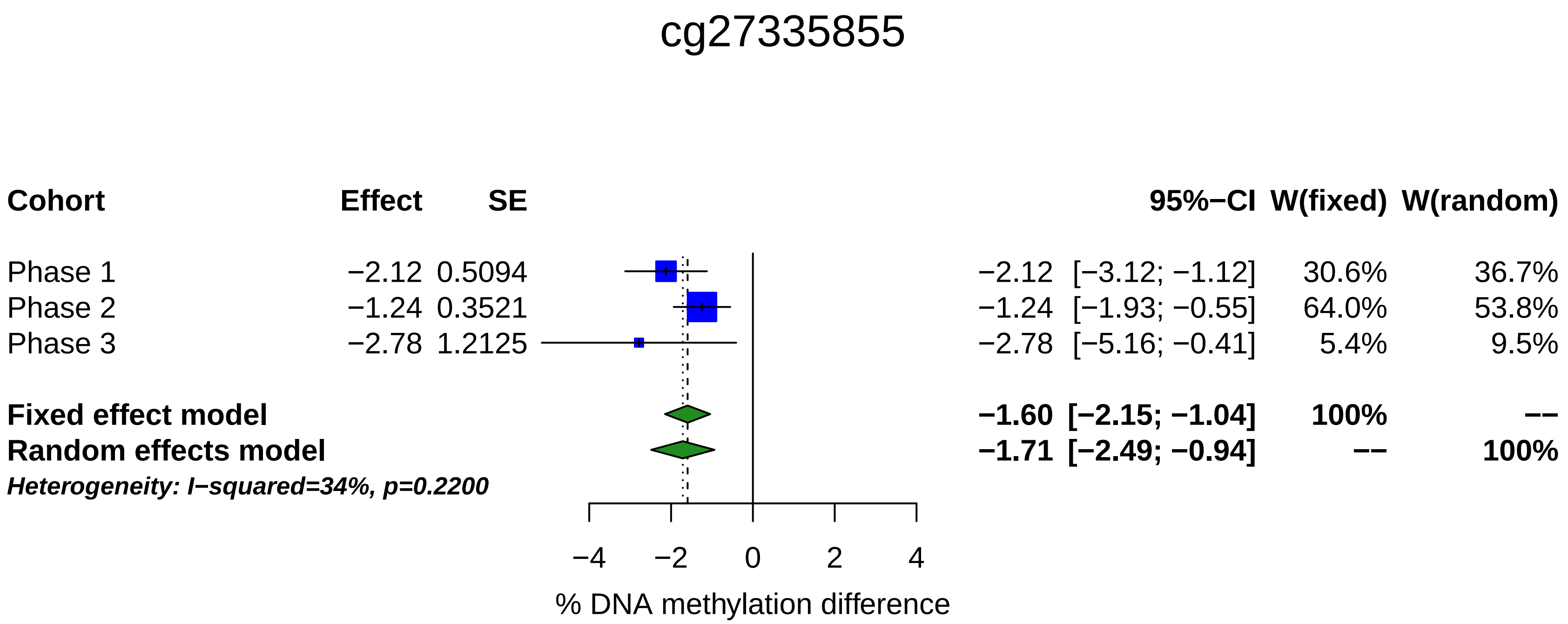
cg27335855
Cohort
Effect
SE
95%−CI
W(fixed)
W(random)
Phase 1 −2.12 0.5094 −2.12 [−3.12; −1.12] 30.6% 36.7%
Phase 2 −1.24 0.3521 −1.24 [−1.93; −0.55] 64.0% 53.8%
Phase 3 −2.78 1.2125 −2.78 [−5.16; −0.41] 5.4% 9.5%
Fixed effect model −1.60 [−2.15; −1.04] 100% −−
Random effects model −1.71 [−2.49; −0.94] −− 100%
Heterogeneity: I−squared=34%, p=0.2200
−4
−2
0
2
4
% DNA methylation difference

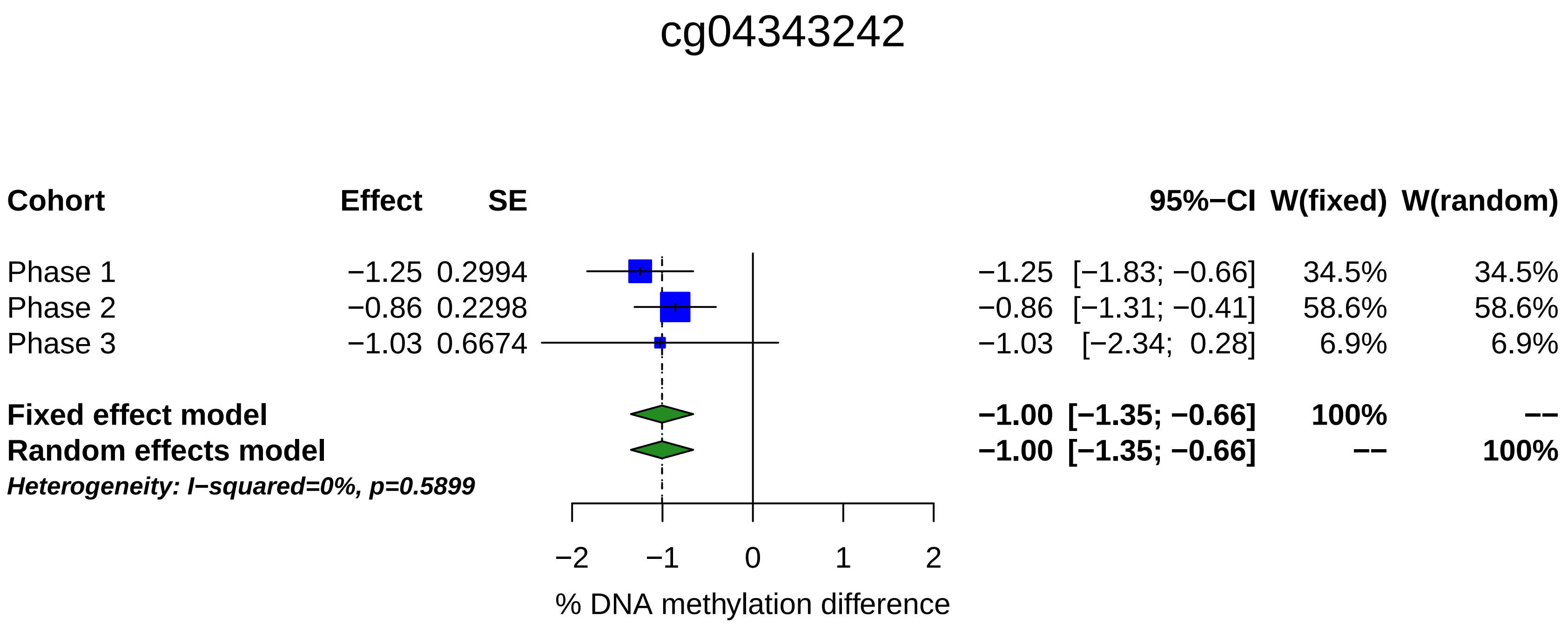
cg04343242
Cohort
Effect
SE
95%−CI
W(fixed)
W(random)
Phase 1 −1.25 0.2994 −1.25 [−1.83; −0.66] 34.5% 34.5%
Phase 2 −0.86 0.2298 −0.86 [−1.31; −0.41] 58.6% 58.6%
Phase 3 −1.03 0.6674 −1.03 [−2.34; 0.28] 6.9% 6.9%
Fixed effect model −1.00 [−1.35; −0.66] 100% −−
Random effects model −1.00 [−1.35; −0.66] −− 100%
Heterogeneity: I−squared=0%, p=0.5899
−2
−1
0
1
2
% DNA methylation difference

cg07504780

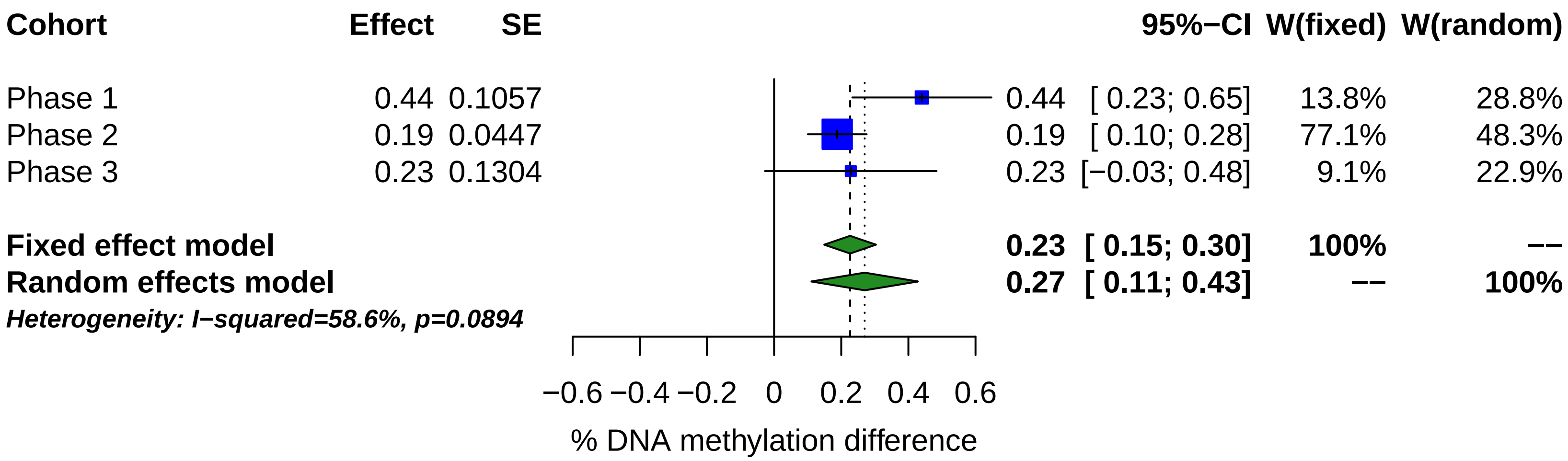

Cohort Effect SE 95%−CI W(fixed) W(random)
Phase 1 0.44 0.1057 0.44 [0.23; 0.65] 13.8% 28.8%
Phase 2 0.19 0.0447 0.19 [0.10; 0.28] 77.1% 48.3%
Phase 3 0.23 0.1304 0.23 [−0.03; 0.48] 9.1% 22.9%
Fixed effect model 0.23 [0.15; 0.30] 100% −−
Random effects model 0.27 [0.11; 0.43] −− 100%
Heterogeneity: I−squared=58.6%, p=0.0894
−0.6 −0.4 −0.2 0 0.2 0.4 0.6
% DNA methylation difference

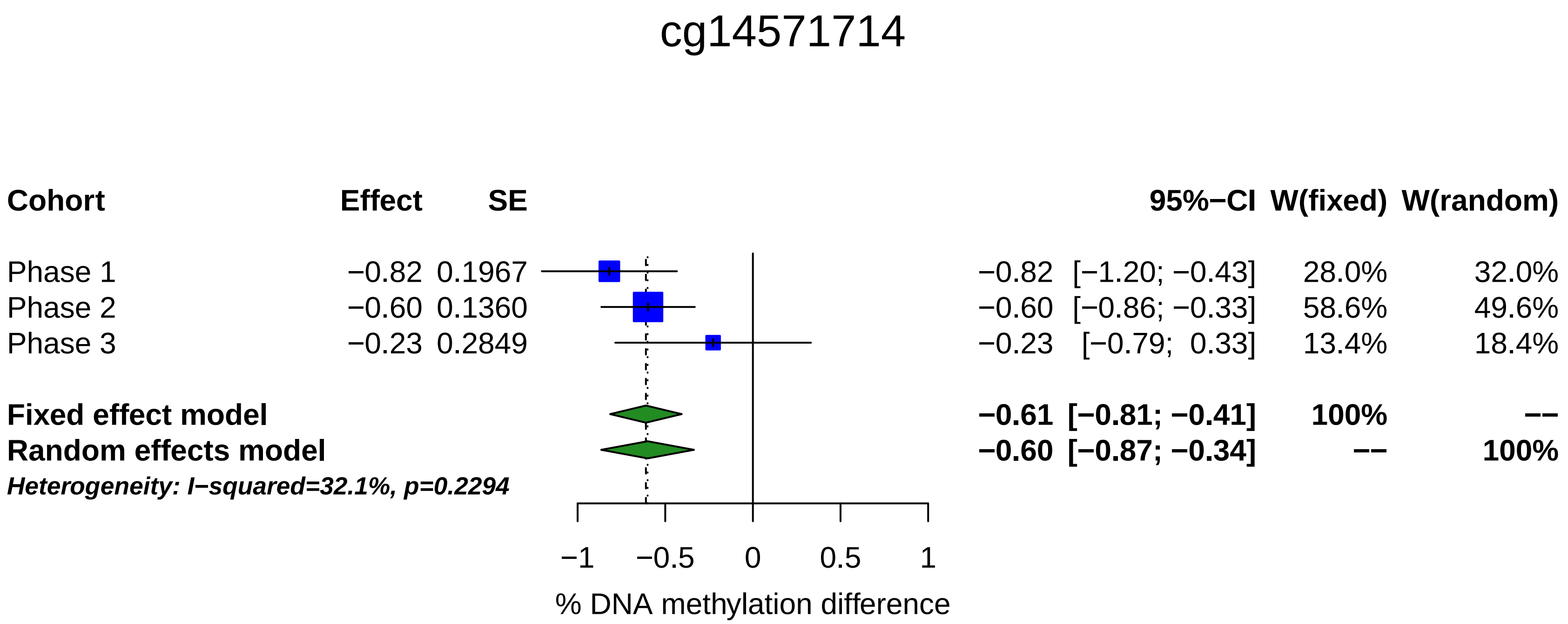
cg14571714
Cohort
Effect
SE
95%−CI
W(fixed)
W(random)
Phase 1 −0.82 0.1967 −0.82 [−1.20; −0.43] 28.0% 32.0%
Phase 2 −0.60 0.1360 −0.60 [−0.86; −0.33] 58.6% 49.6%
Phase 3 −0.23 0.2849 −0.23 [−0.79; 0.33] 13.4% 18.4%
Fixed effect model −0.61 [−0.81; −0.41] 100% −−
Random effects model −0.60 [−0.87; −0.34] −− 100%
Heterogeneity: I−squared=32.1%, p=0.2294
−1
−0.5
0
0.5
1
% DNA methylation difference

cg14084456

Cohort	Effect	SE	95%−CI	W(fixed)	W(random)
Phase 1	0.64	0.1531	0.64 [0.34; 0.94]	17.1%	28.3%
Phase 2	0.36	0.0719	0.36 [0.22; 0.50]	77.7%	60.8%
Phase 3	0.25	0.2791	0.25 [−0.29; 0.80]	5.2%	10.9%
**Fixed effect model**			**0.40 [0.28; 0.53]**	**100%**	**−−**
**Random effects model**			**0.43 [0.23; 0.62]**	**−−**	**100%**

Heterogeneity: I−squared=32.8%, p=0.2257

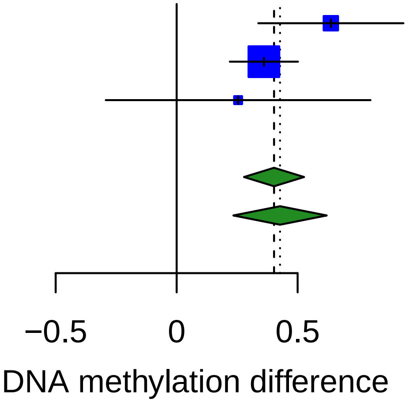

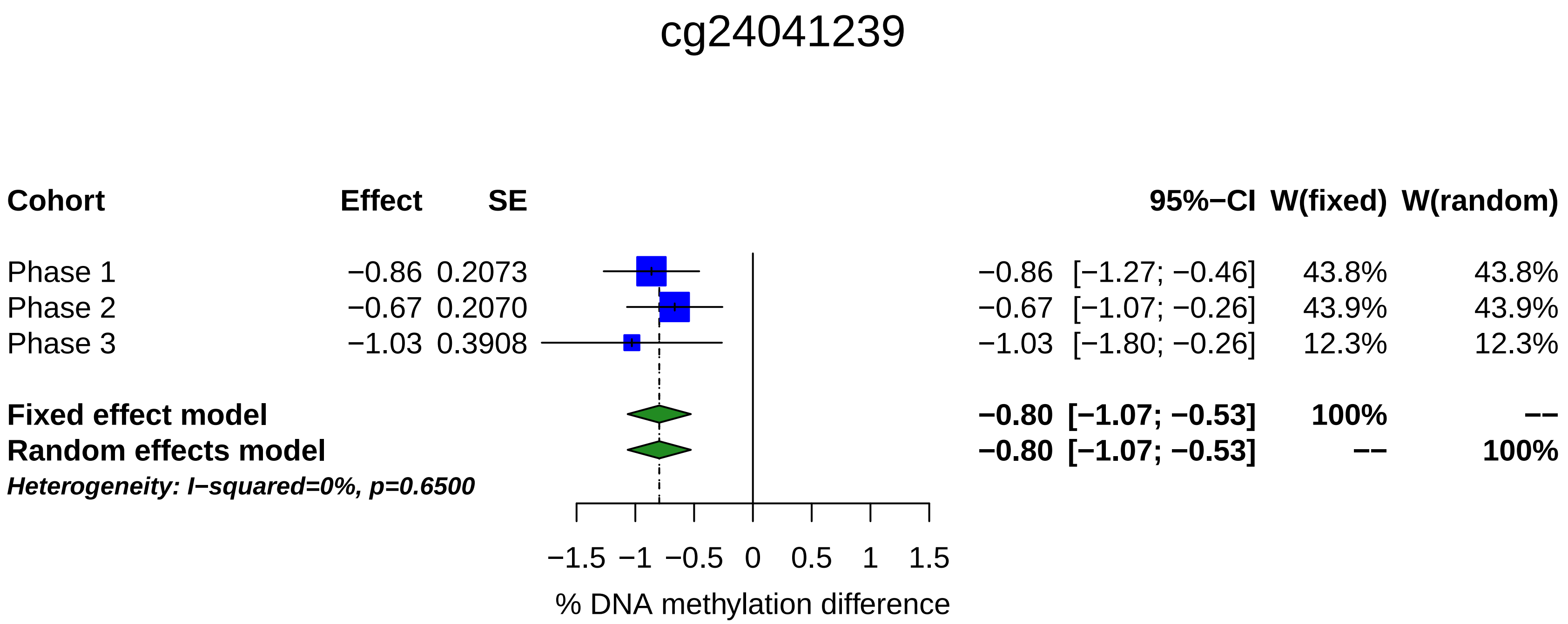

cg24041239
Cohort
Effect
SE
95%−CI
W(fixed)
W(random)
Phase 1 −0.86 0.2073 −0.86 [−1.27; −0.46] 43.8% 43.8%
Phase 2 −0.67 0.2070 −0.67 [−1.07; −0.26] 43.9% 43.9%
Phase 3 −1.03 0.3908 −1.03 [−1.80; −0.26] 12.3% 12.3%
Fixed effect model −0.80 [−1.07; −0.53] 100% −−
Random effects model −0.80 [−1.07; −0.53] −− 100%
Heterogeneity: I−squared=0%, p=0.6500
−1.5 −1 −0.5 0 0.5 1 1.5
% DNA methylation difference

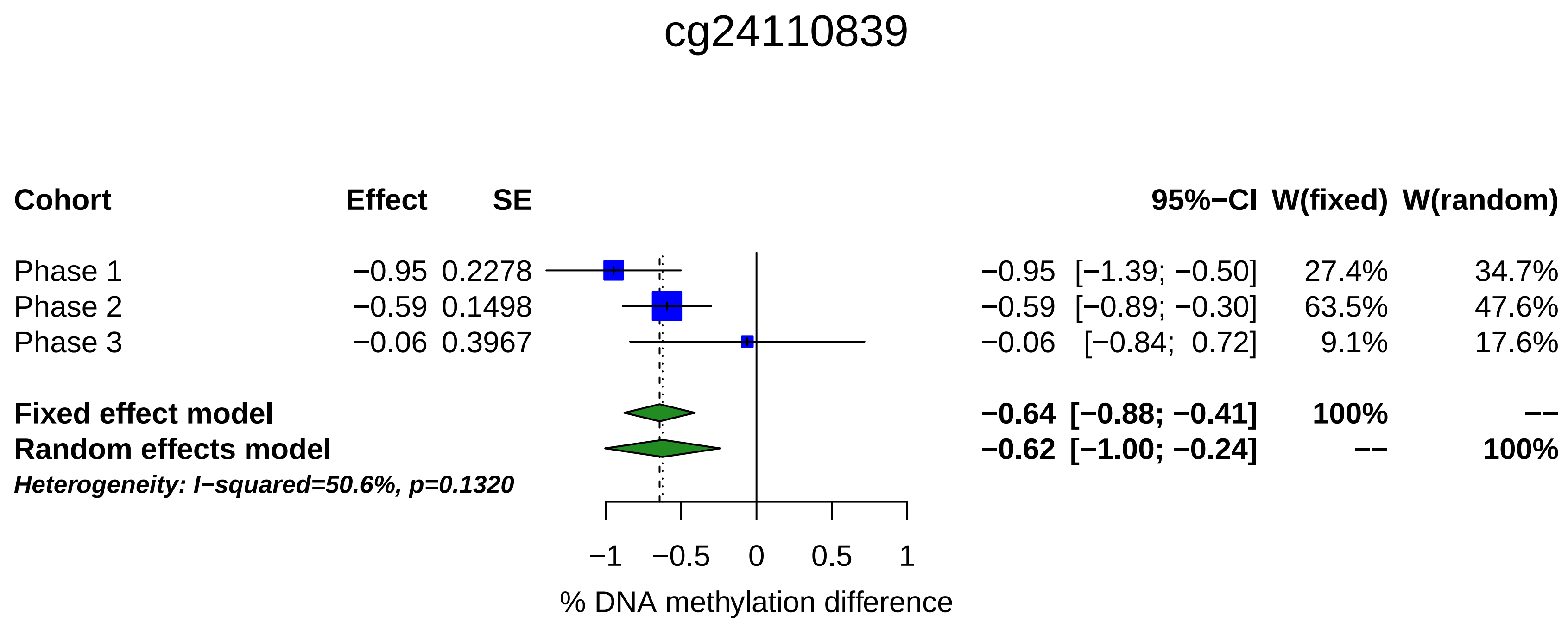

cg24110839
Cohort
Effect
SE
95%−CI
W(fixed)
W(random)
Phase 1 −0.95 0.2278 −0.95 [−1.39; −0.50] 27.4% 34.7%
Phase 2 −0.59 0.1498 −0.59 [−0.89; −0.30] 63.5% 47.6%
Phase 3 −0.06 0.3967 −0.06 [−0.84; 0.72] 9.1% 17.6%
Fixed effect model −0.64 [−0.88; −0.41] 100% −−
Random effects model −0.62 [−1.00; −0.24] −− 100%
Heterogeneity: I−squared=50.6%, p=0.1320
−1
−0.5
0
0.5
1
% DNA methylation difference

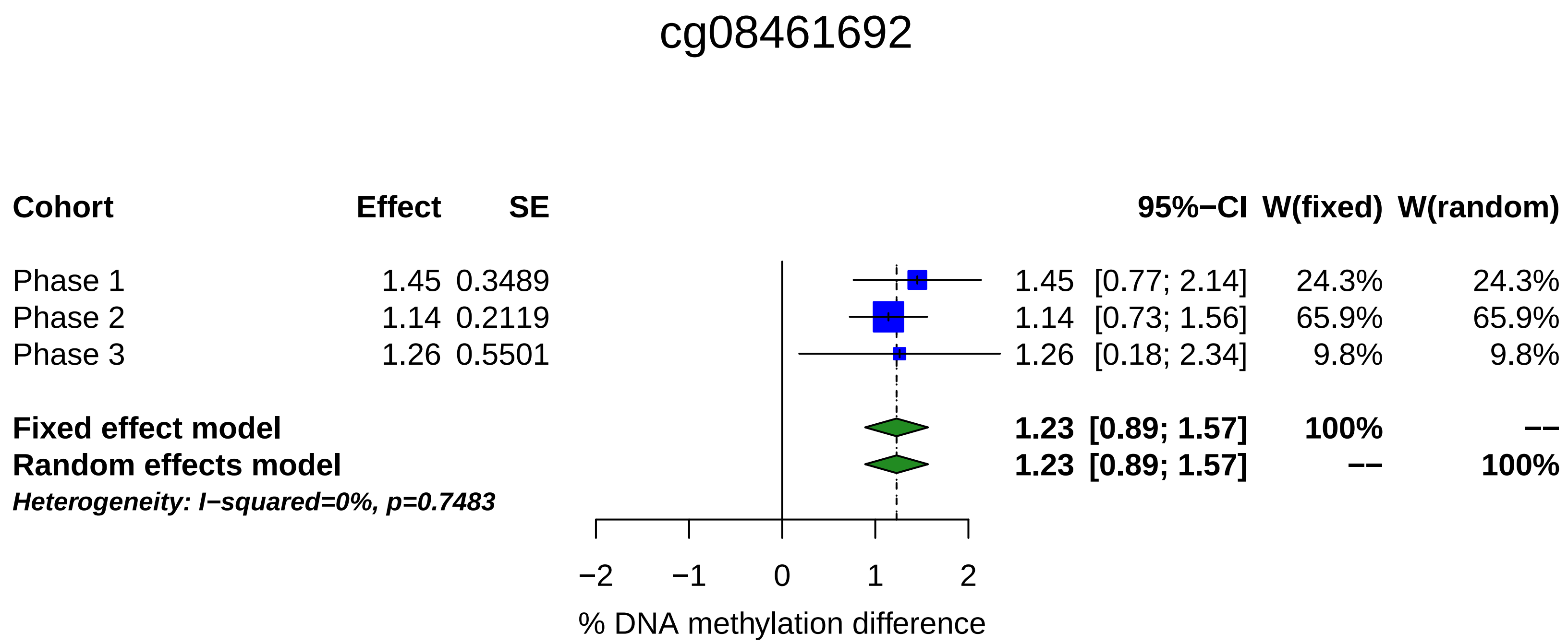
cg08461692
Cohort
Effect
SE
95%−CI
W(fixed)
W(random)
Phase 1
1.45
0.3489
1.45 [0.77; 2.14]
24.3%
24.3%
Phase 2
1.14
0.2119
1.14 [0.73; 1.56]
65.9%
65.9%
Phase 3
1.26
0.5501
1.26 [0.18; 2.34]
9.8%
9.8%
Fixed effect model
1.23 [0.89; 1.57]
100%
−−
Random effects model
1.23 [0.89; 1.57]
−−
100%
Heterogeneity: I−squared=0%, p=0.7483
−2
−1
0
1
2
% DNA methylation difference

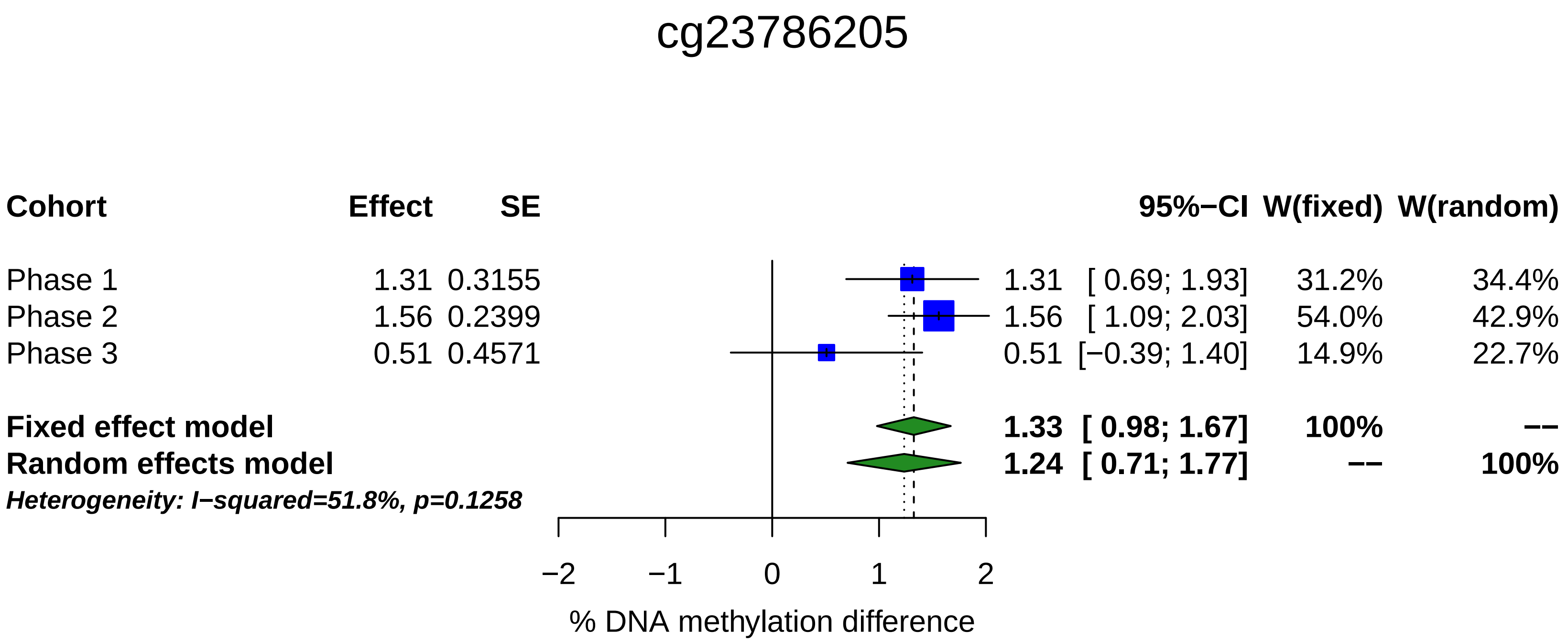
cg23786205
Cohort
Effect
SE
95%−CI
W(fixed)
W(random)
Phase 1 1.31 0.3155 1.31 [0.69; 1.93] 31.2% 34.4%
Phase 2 1.56 0.2399 1.56 [1.09; 2.03] 54.0% 42.9%
Phase 3 0.51 0.4571 0.51 [−0.39; 1.40] 14.9% 22.7%
Fixed effect model 1.33 [0.98; 1.67] 100% −−
Random effects model 1.24 [0.71; 1.77] −− 100%
Heterogeneity: I−squared=51.8%, p=0.1258
−2
−1
0
1
2
% DNA methylation difference

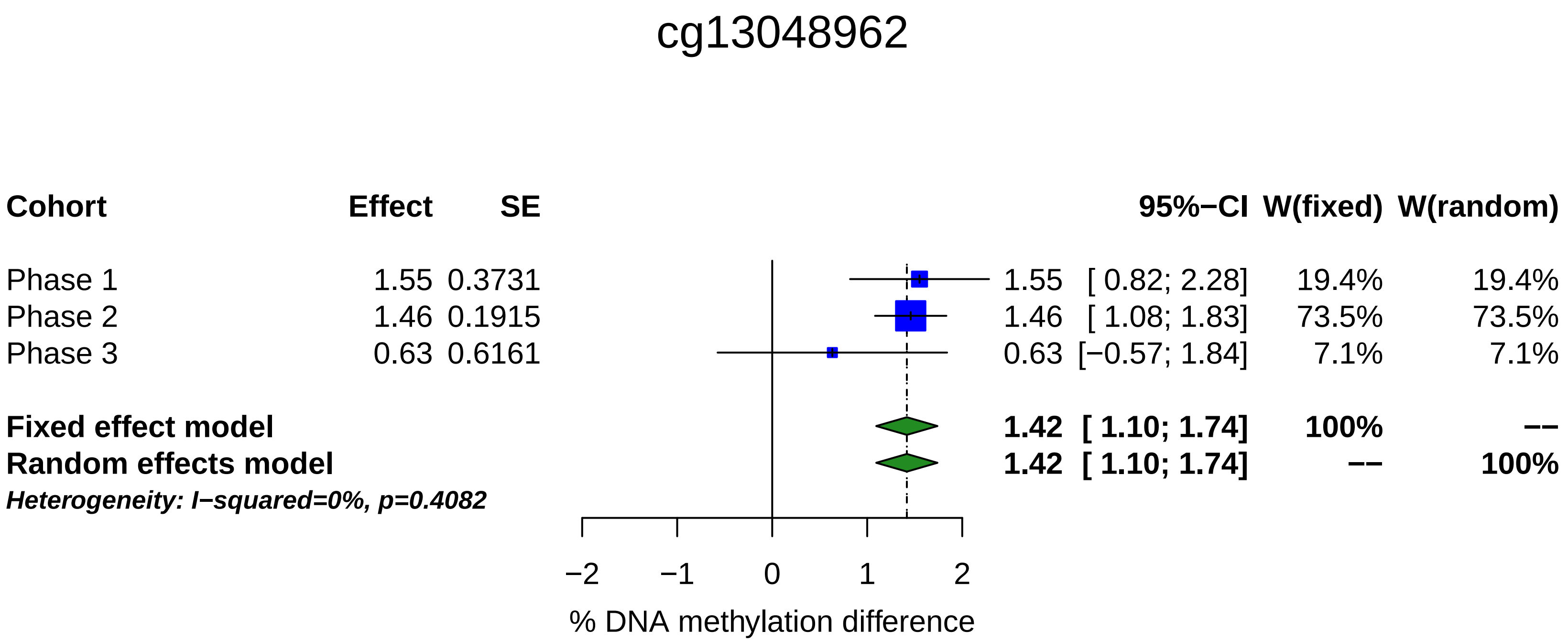
cg13048962
Cohort
Effect
SE
95%−CI
W(fixed)
W(random)
Phase 1 1.55 0.3731 1.55 [0.82; 2.28] 19.4% 19.4%
Phase 2 1.46 0.1915 1.46 [1.08; 1.83] 73.5% 73.5%
Phase 3 0.63 0.6161 0.63 [−0.57; 1.84] 7.1% 7.1%
Fixed effect model 1.42 [1.10; 1.74] 100% −−
Random effects model 1.42 [1.10; 1.74] −− 100%
Heterogeneity: I−squared=0%, p=0.4082
−2
−1
0
1
2
% DNA methylation difference

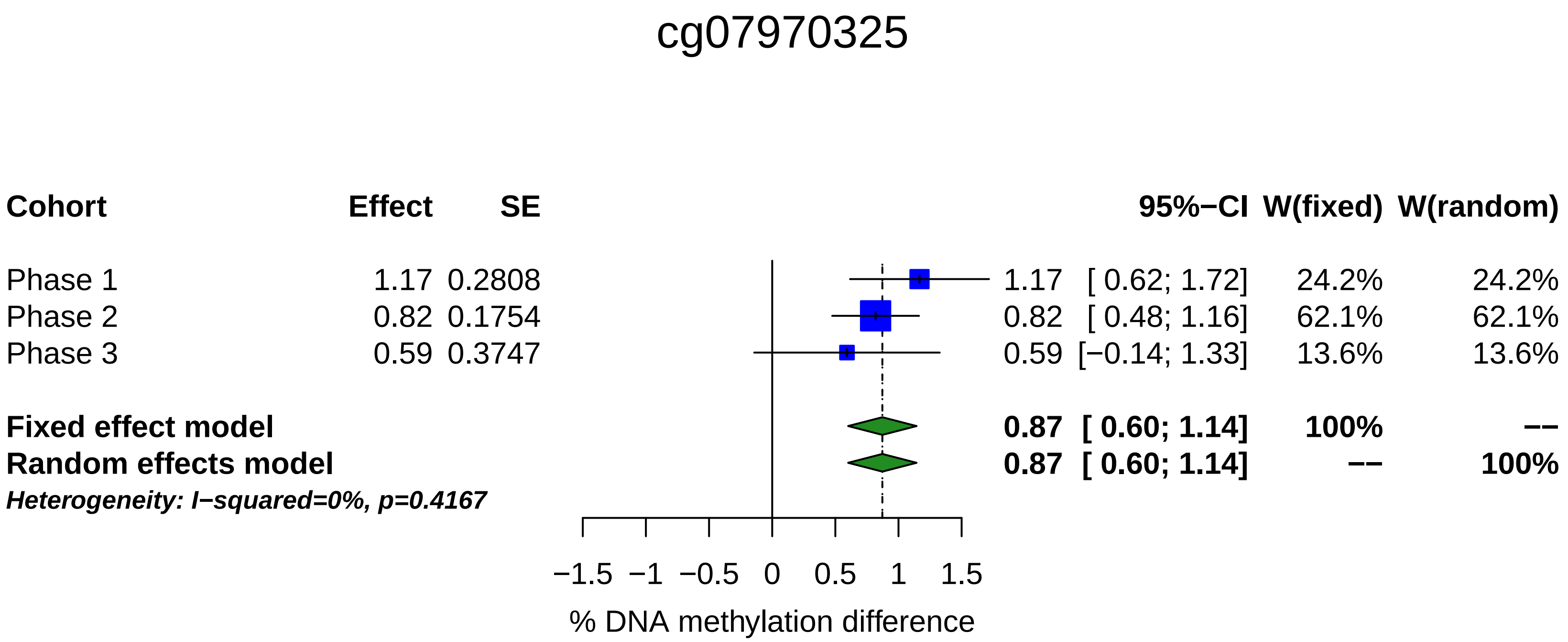
cg07970325
Cohort
Effect
SE
95%−CI
W(fixed)
W(random)
Phase 1 1.17 0.2808 1.17 [0.62; 1.72] 24.2% 24.2%
Phase 2 0.82 0.1754 0.82 [0.48; 1.16] 62.1% 62.1%
Phase 3 0.59 0.3747 0.59 [−0.14; 1.33] 13.6% 13.6%
Fixed effect model 0.87 [0.60; 1.14] 100% −−
Random effects model 0.87 [0.60; 1.14] −− 100%
Heterogeneity: I−squared=0%, p=0.4167
−1.5 −1 −0.5 0 0.5 1 1.5
% DNA methylation difference

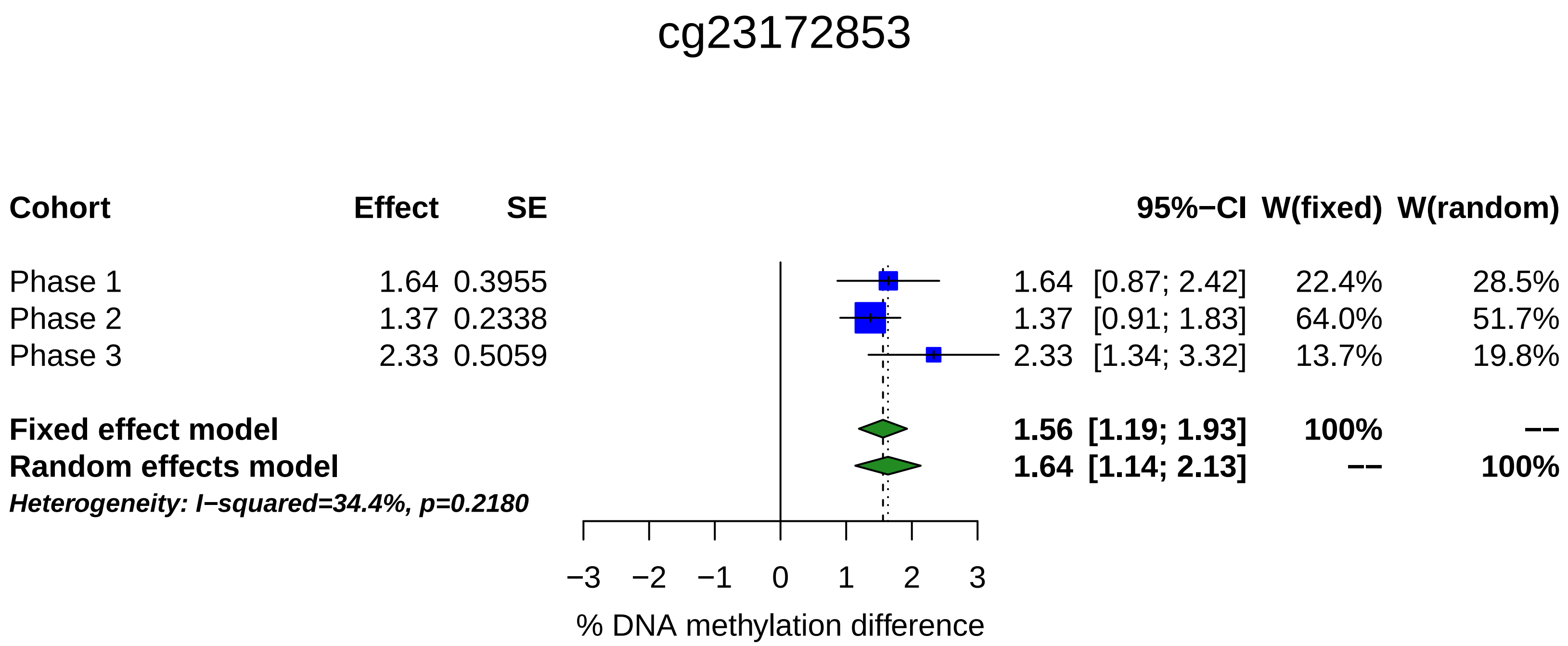
cg23172853
Cohort
Effect
SE
95%−CI
W(fixed)
W(random)
Phase 1 1.64 0.3955 1.64 [0.87; 2.42] 22.4% 28.5%
Phase 2 1.37 0.2338 1.37 [0.91; 1.83] 64.0% 51.7%
Phase 3 2.33 0.5059 2.33 [1.34; 3.32] 13.7% 19.8%
Fixed effect model 1.56 [1.19; 1.93] 100% −−
Random effects model 1.64 [1.14; 2.13] −− 100%
Heterogeneity: I−squared=34.4%, p=0.2180
−3
−2
−1
0
1
2
3
% DNA methylation difference

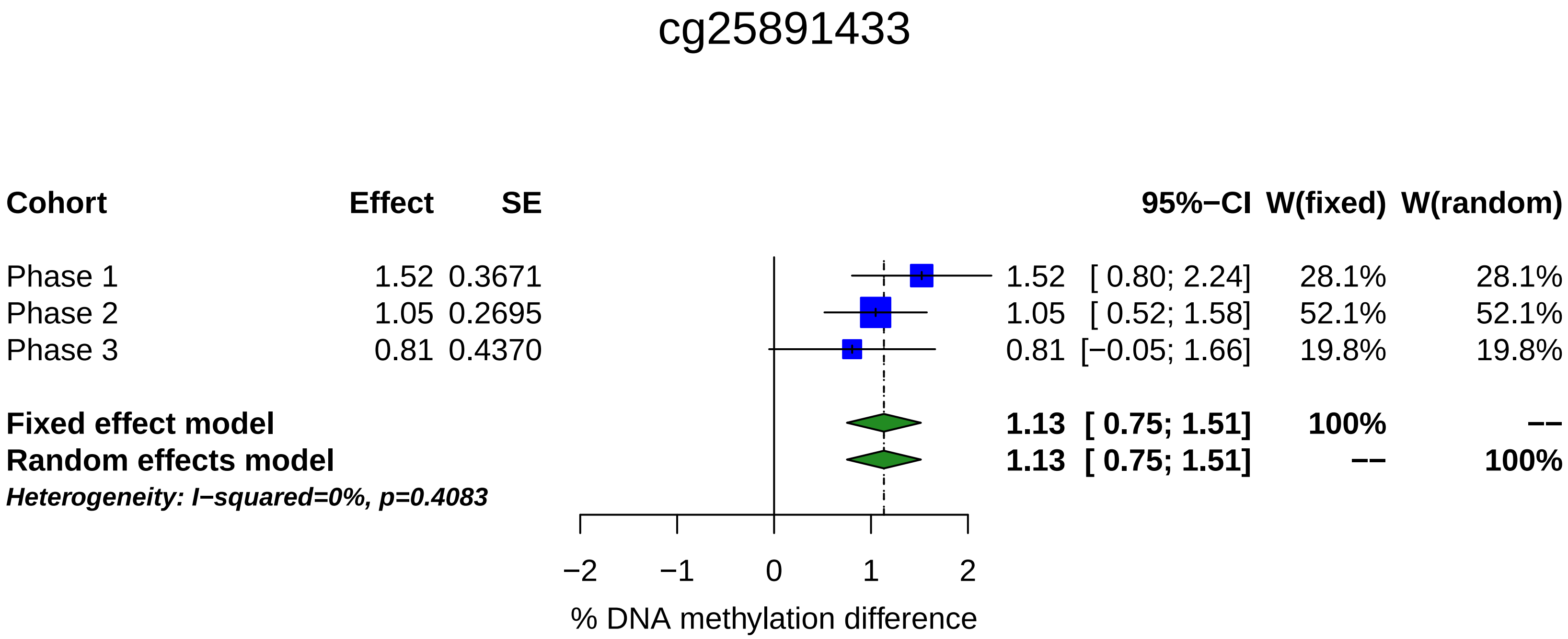
cg25891433
Cohort
Effect
SE
95%−CI
W(fixed)
W(random)
Phase 1 1.52 0.3671 1.52 [0.80; 2.24] 28.1% 28.1%
Phase 2 1.05 0.2695 1.05 [0.52; 1.58] 52.1% 52.1%
Phase 3 0.81 0.4370 0.81 [−0.05; 1.66] 19.8% 19.8%
Fixed effect model 1.13 [0.75; 1.51] 100% −−
Random effects model 1.13 [0.75; 1.51] −− 100%
Heterogeneity: I−squared=0%, p=0.4083
−2
−1
0
1
2
% DNA methylation difference

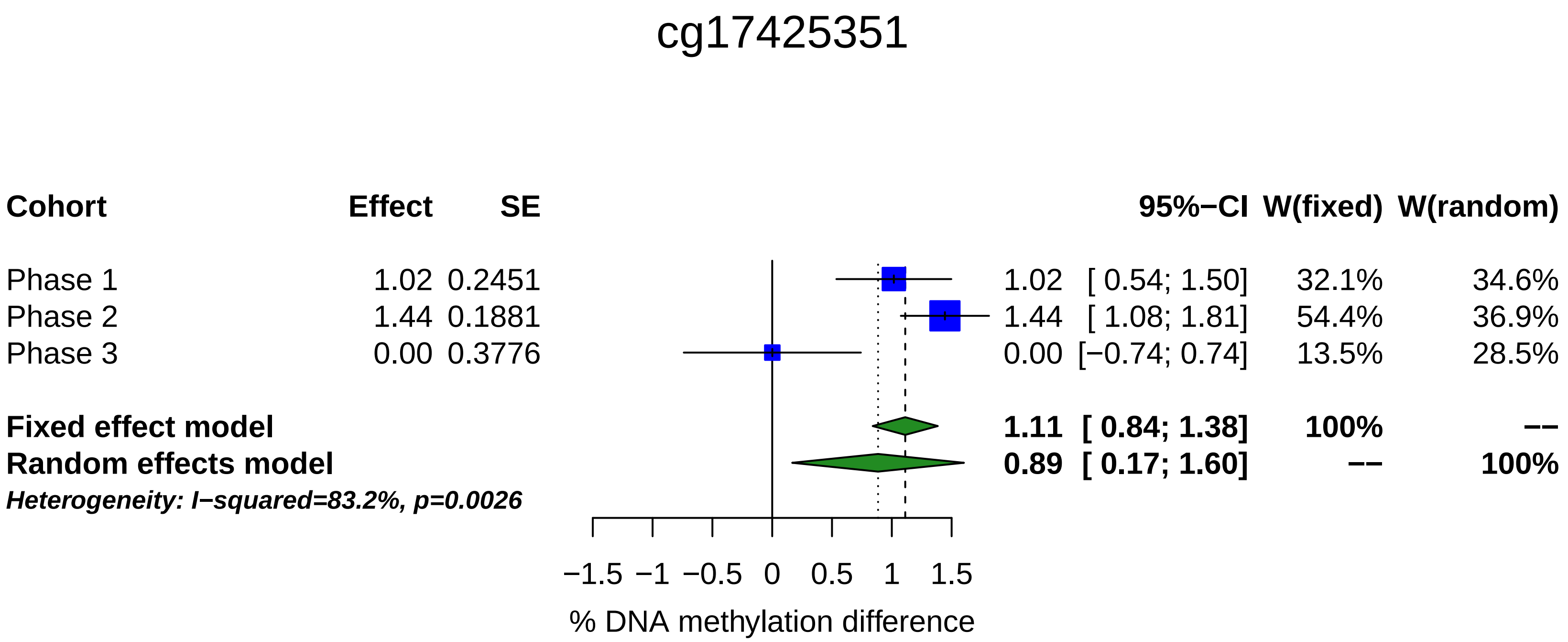
cg17425351
Cohort
Effect
SE
95%−CI
W(fixed)
W(random)
Phase 1 1.02 0.2451 1.02 [0.54; 1.50] 32.1% 34.6%
Phase 2 1.44 0.1881 1.44 [1.08; 1.81] 54.4% 36.9%
Phase 3 0.00 0.3776 0.00 [−0.74; 0.74] 13.5% 28.5%
Fixed effect model 1.11 [0.84; 1.38] 100% −−
Random effects model 0.89 [0.17; 1.60] −− 100%
Heterogeneity: I−squared=83.2%, p=0.0026
−1.5 −1 −0.5 0 0.5 1 1.5
% DNA methylation difference

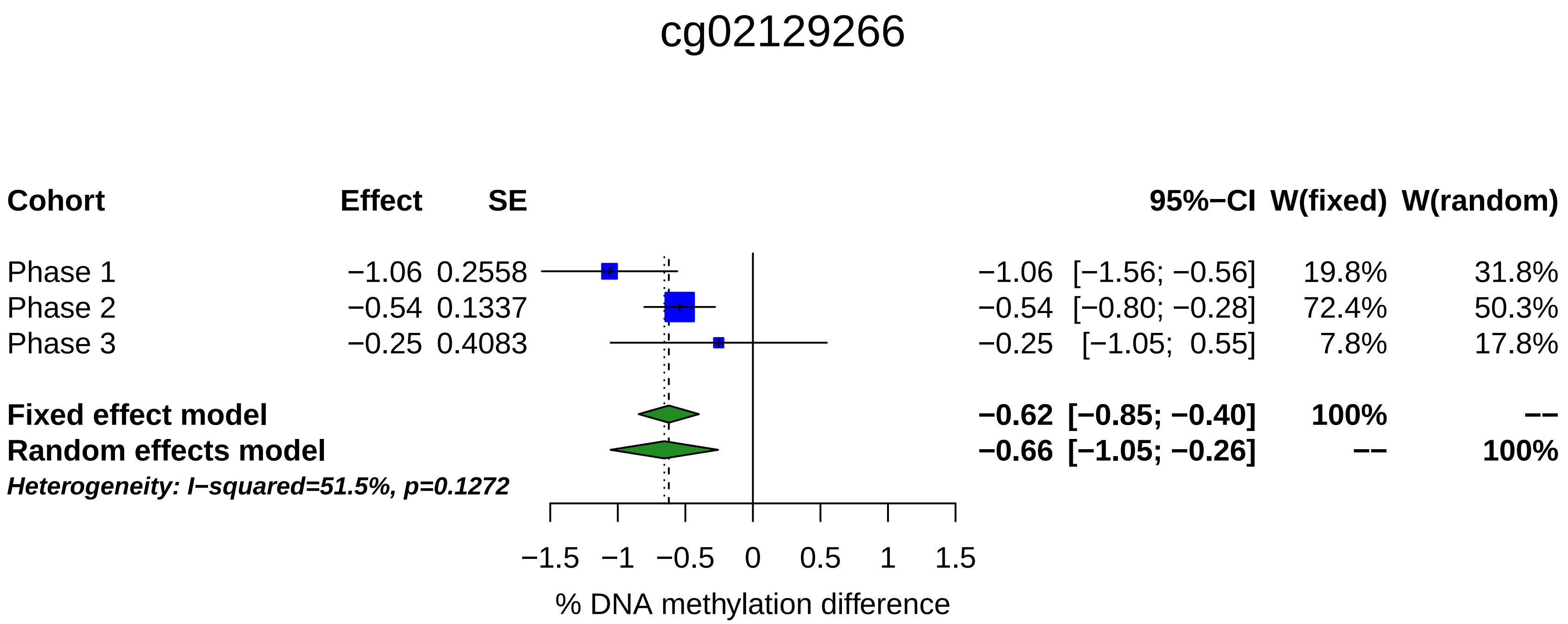
cg02129266
Cohort
Effect
SE
95%−CI
W(fixed)
W(random)
Phase 1 −1.06 0.2558 −1.06 [−1.56; −0.56] 19.8% 31.8%
Phase 2 −0.54 0.1337 −0.54 [−0.80; −0.28] 72.4% 50.3%
Phase 3 −0.25 0.4083 −0.25 [−1.05; 0.55] 7.8% 17.8%
Fixed effect model −0.62 [−0.85; −0.40] 100% −−
Random effects model −0.66 [−1.05; −0.26] −− 100%
Heterogeneity: I−squared=51.5%, p=0.1272
−1.5
−1
−0.5
0
0.5
1
1.5
% DNA methylation difference

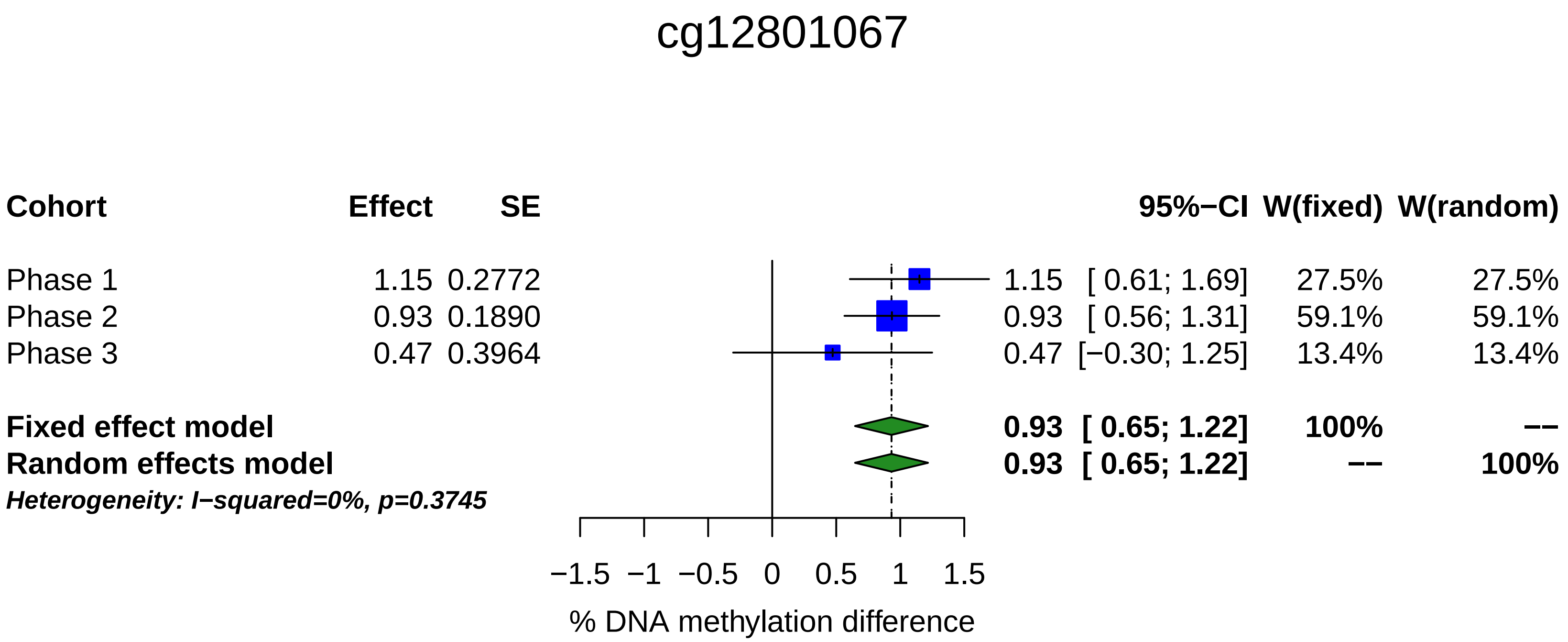
cg12801067
Cohort
Effect
SE
95%−CI
W(fixed)
W(random)
Phase 1
1.15
0.2772
1.15 [0.61; 1.69]
27.5%
27.5%
Phase 2
0.93
0.1890
0.93 [0.56; 1.31]
59.1%
59.1%
Phase 3
0.47
0.3964
0.47 [−0.30; 1.25]
13.4%
13.4%
Fixed effect model
0.93 [0.65; 1.22]
100%
−−
Random effects model
0.93 [0.65; 1.22]
−−
100%
Heterogeneity: I−squared=0%, p=0.3745
−1.5
−1
−0.5
0
0.5
1
1.5
% DNA methylation difference

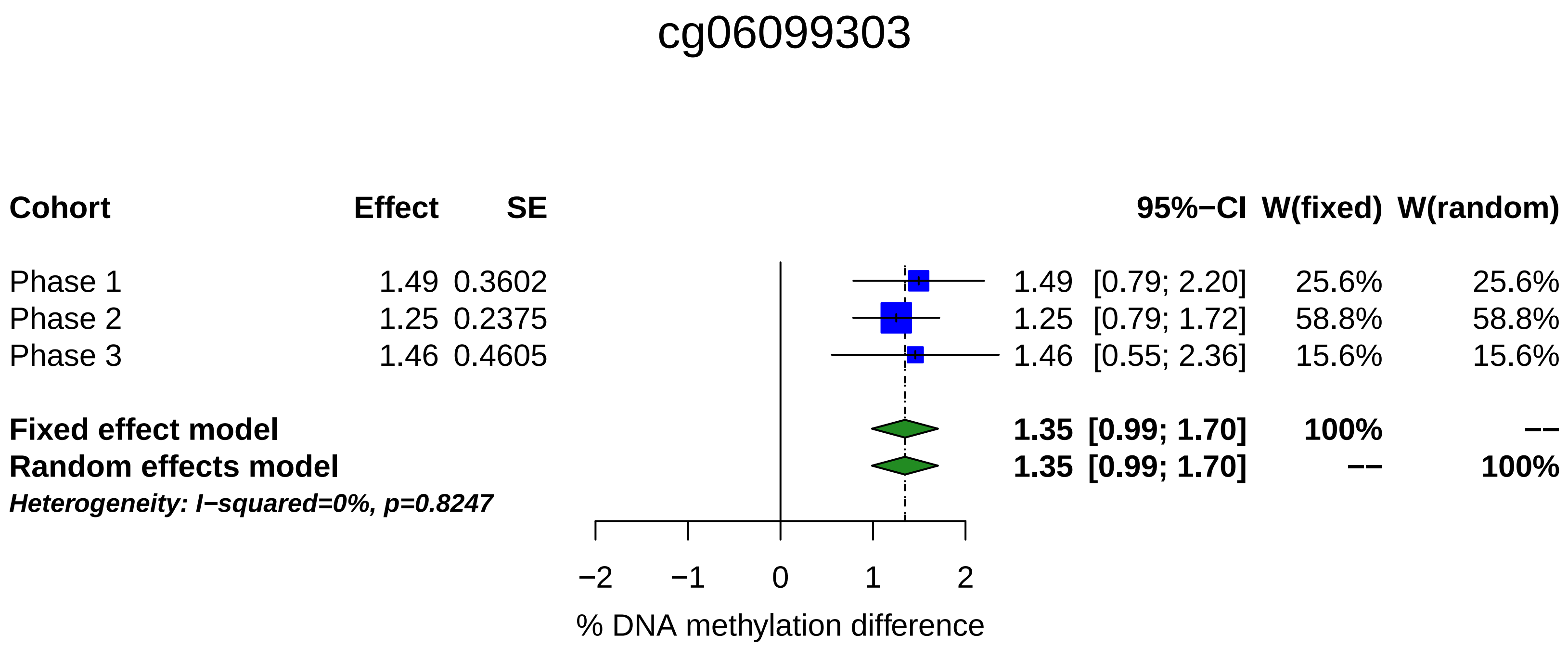
cg06099303
Cohort
Effect
SE
95%−CI
W(fixed)
W(random)
Phase 1 1.49 0.3602 1.49 [0.79; 2.20] 25.6% 25.6%
Phase 2 1.25 0.2375 1.25 [0.79; 1.72] 58.8% 58.8%
Phase 3 1.46 0.4605 1.46 [0.55; 2.36] 15.6% 15.6%
Fixed effect model 1.35 [0.99; 1.70] 100% −−
Random effects model 1.35 [0.99; 1.70] −− 100%
Heterogeneity: I−squared=0%, p=0.8247
−2
−1
0
1
2
% DNA methylation difference

cg23426156

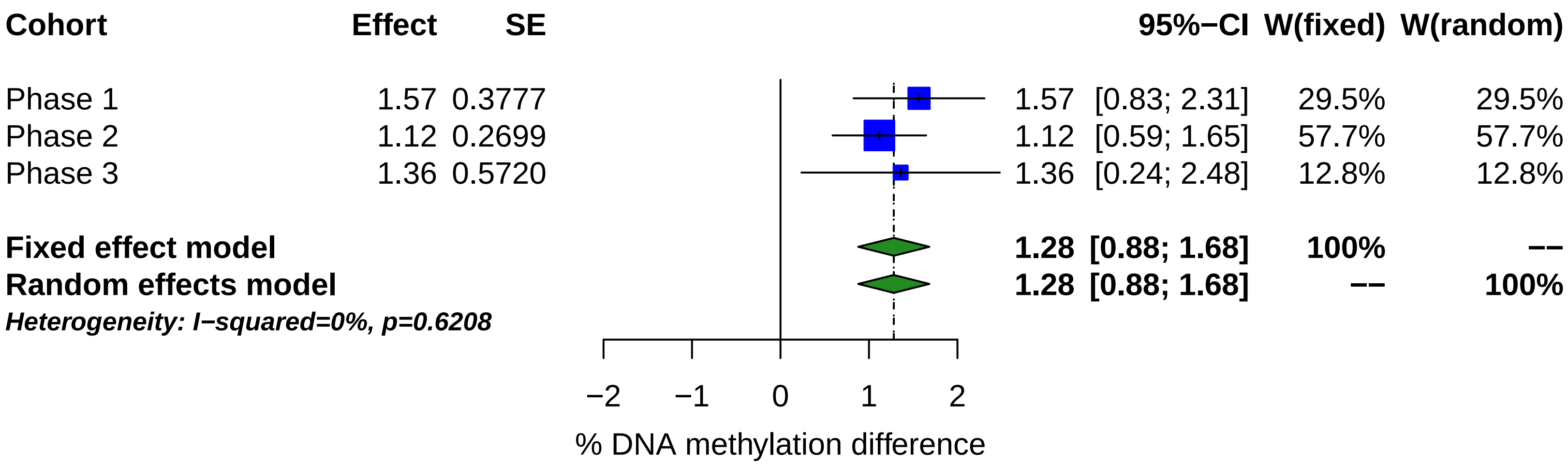
Cohort Effect SE 95%−CI W(fixed) W(random)
Phase 1 1.57 0.3777 1.57 [0.83; 2.31] 29.5% 29.5%
Phase 2 1.12 0.2699 1.12 [0.59; 1.65] 57.7% 57.7%
Phase 3 1.36 0.5720 1.36 [0.24; 2.48] 12.8% 12.8%
Fixed effect model 1.28 [0.88; 1.68] 100% −−
Random effects model 1.28 [0.88; 1.68] −− 100%
Heterogeneity: I−squared=0%, p=0.6208
−2 −1 0 1 2
% DNA methylation difference

cg04257163

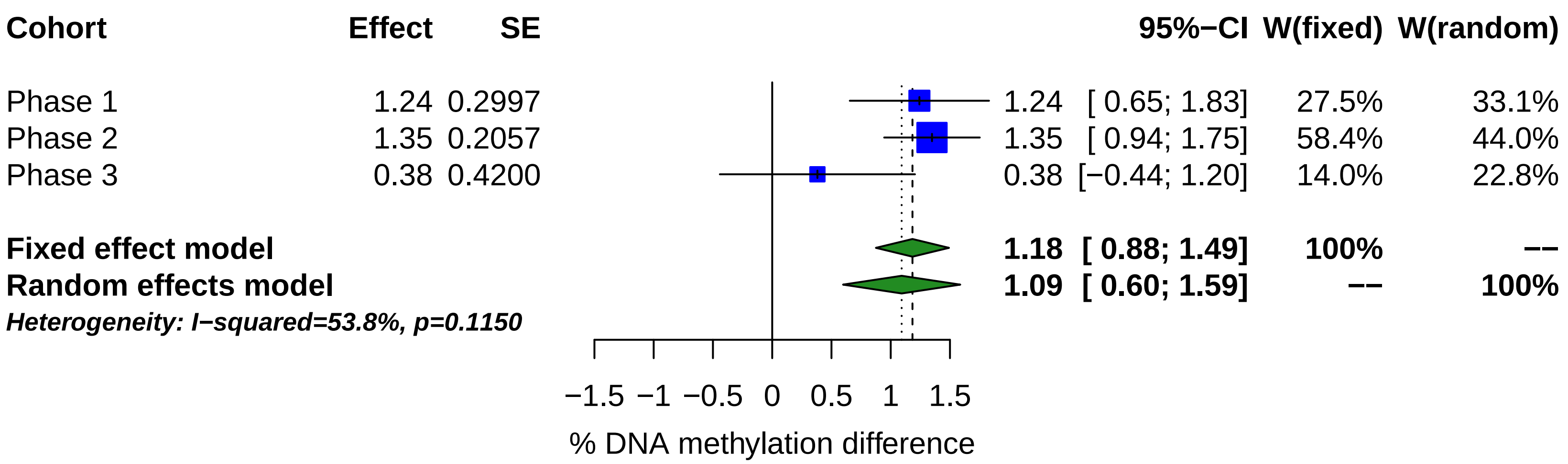

Cohort
Effect
SE
95%−CI
W(fixed)
W(random)
Phase 1 1.24 0.2997 1.24 [0.65; 1.83] 27.5% 33.1%
Phase 2 1.35 0.2057 1.35 [0.94; 1.75] 58.4% 44.0%
Phase 3 0.38 0.4200 0.38 [−0.44; 1.20] 14.0% 22.8%
Fixed effect model 1.18 [0.88; 1.49] 100% −−
Random effects model 1.09 [0.60; 1.59] −− 100%
Heterogeneity: I−squared=53.8%, p=0.1150
−1.5
−1
−0.5
0
0.5
1
1.5
% DNA methylation difference

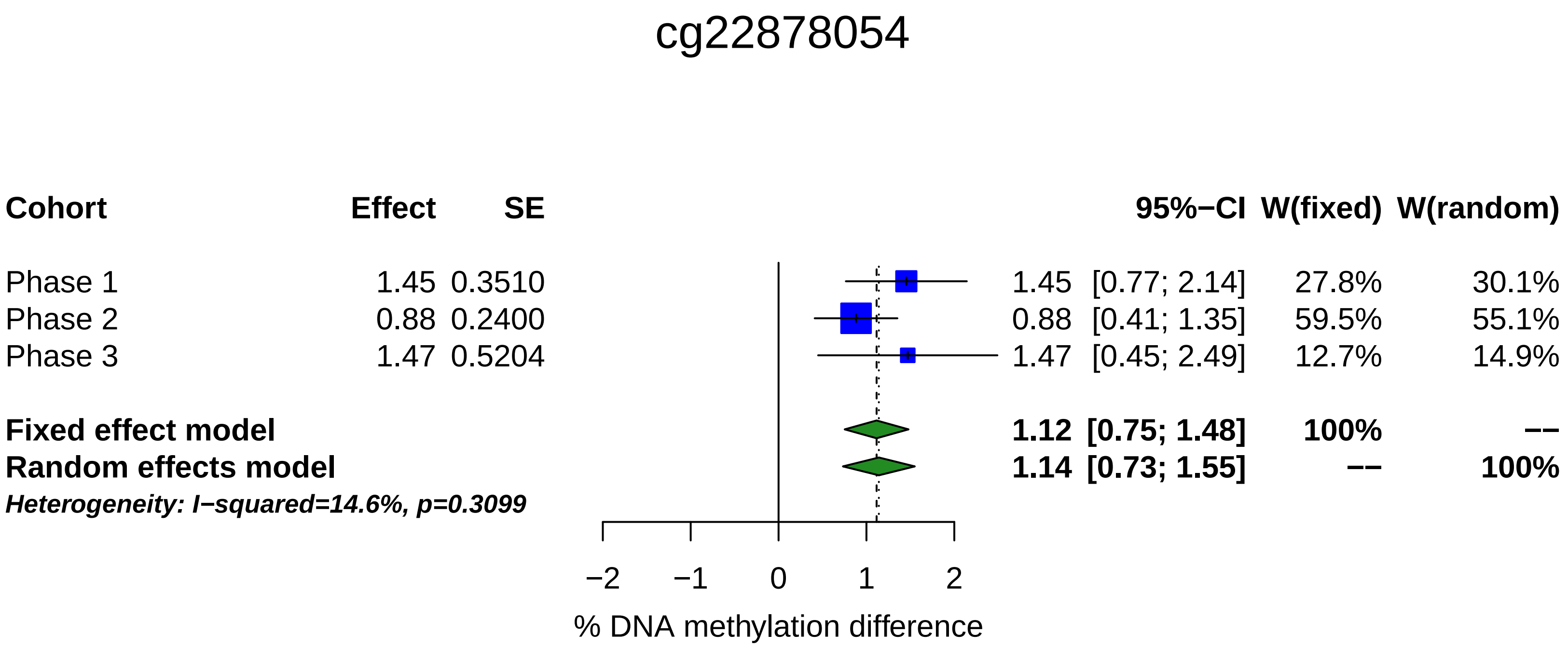
cg22878054
Cohort Effect SE 95%−CI W(fixed) W(random)
Phase 1 1.45 0.3510 1.45 [0.77; 2.14] 27.8% 30.1%
Phase 2 0.88 0.2400 0.88 [0.41; 1.35] 59.5% 55.1%
Phase 3 1.47 0.5204 1.47 [0.45; 2.49] 12.7% 14.9%
Fixed effect model 1.12 [0.75; 1.48] 100% −−
Random effects model 1.14 [0.73; 1.55] −− 100%
Heterogeneity: I−squared=14.6%, p=0.3099
−2 −1 0 1 2
% DNA methylation difference

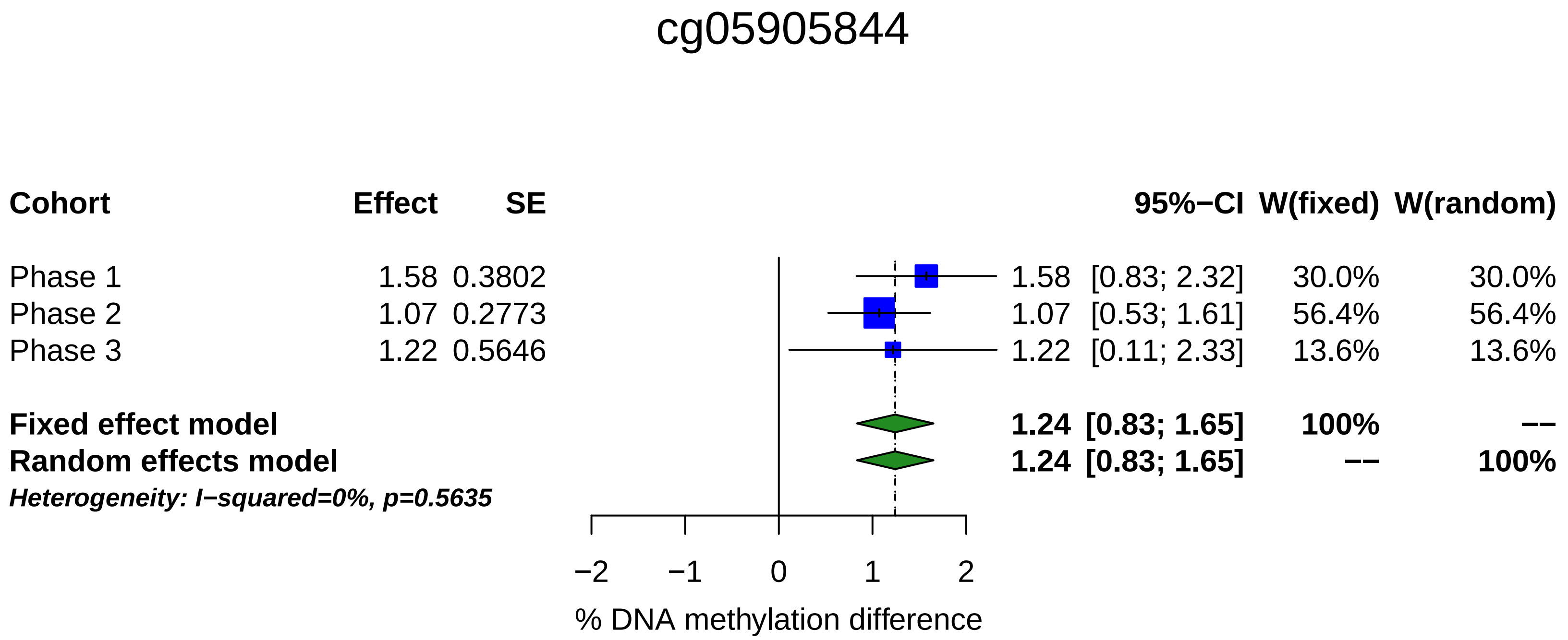
cg05905844
Cohort
Effect
SE
95%−CI
W(fixed)
W(random)
Phase 1 1.58 0.3802 1.58 [0.83; 2.32] 30.0% 30.0%
Phase 2 1.07 0.2773 1.07 [0.53; 1.61] 56.4% 56.4%
Phase 3 1.22 0.5646 1.22 [0.11; 2.33] 13.6% 13.6%
Fixed effect model 1.24 [0.83; 1.65] 100% −−
Random effects model 1.24 [0.83; 1.65] −− 100%
Heterogeneity: I−squared=0%, p=0.5635
−2
−1
0
1
2
% DNA methylation difference

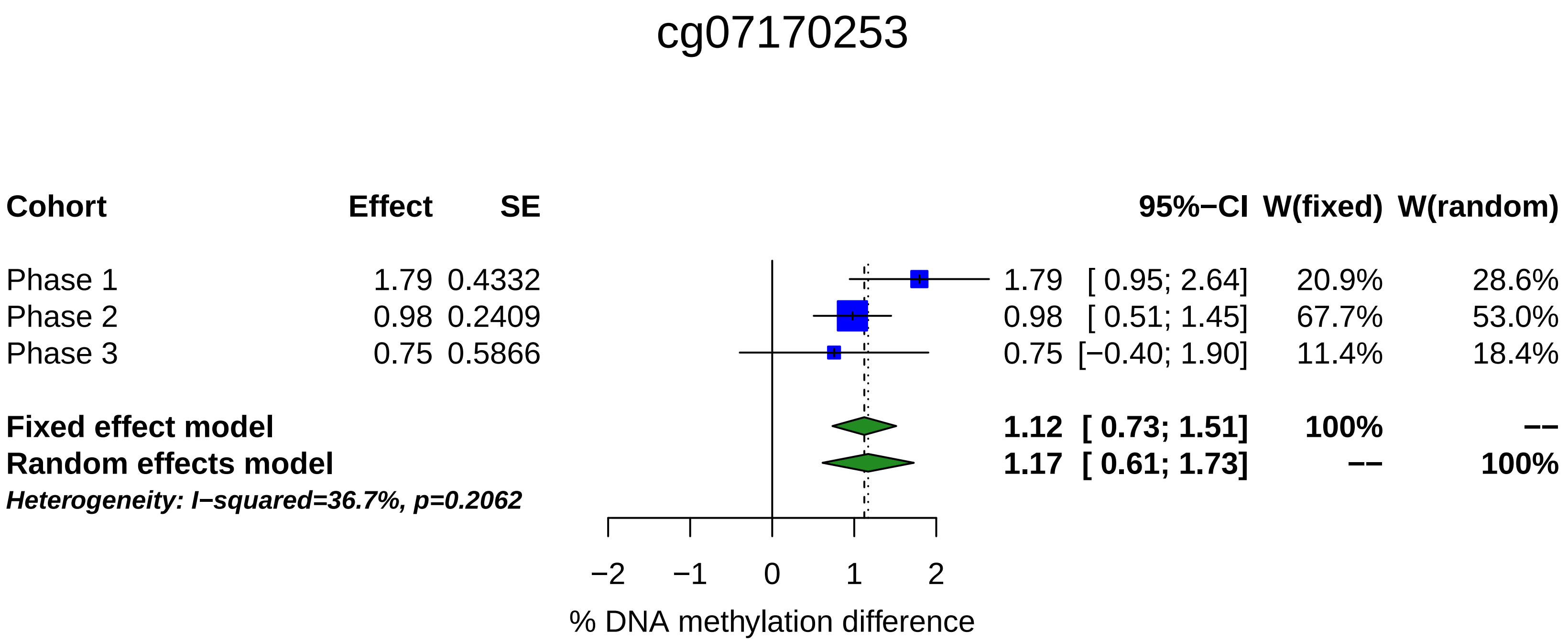
cg07170253
Cohort
Effect
SE
95%−CI
W(fixed)
W(random)
Phase 1 1.79 0.4332 1.79 [0.95; 2.64] 20.9% 28.6%
Phase 2 0.98 0.2409 0.98 [0.51; 1.45] 67.7% 53.0%
Phase 3 0.75 0.5866 0.75 [−0.40; 1.90] 11.4% 18.4%
Fixed effect model 1.12 [0.73; 1.51] 100% −−
Random effects model 1.17 [0.61; 1.73] −− 100%
Heterogeneity: I−squared=36.7%, p=0.2062
−2
−1
0
1
2
% DNA methylation difference

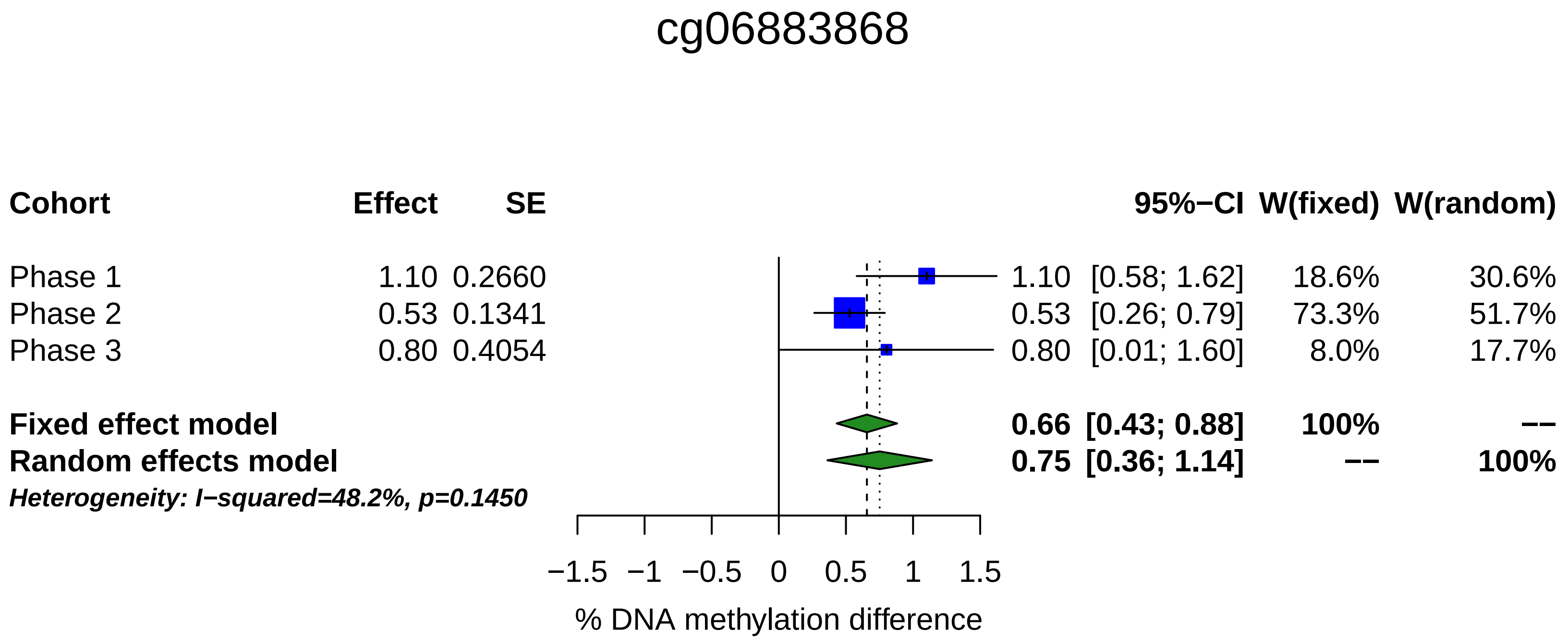
cg06883868
Cohort
Effect
SE
95%−CI
W(fixed)
W(random)
Phase 1 1.10 0.2660 1.10 [0.58; 1.62] 18.6% 30.6%
Phase 2 0.53 0.1341 0.53 [0.26; 0.79] 73.3% 51.7%
Phase 3 0.80 0.4054 0.80 [0.01; 1.60] 8.0% 17.7%
Fixed effect model 0.66 [0.43; 0.88] 100% −−
Random effects model 0.75 [0.36; 1.14] −− 100%
Heterogeneity: I−squared=48.2%, p=0.1450
−1.5
−1
−0.5
0
0.5
1
1.5
% DNA methylation difference

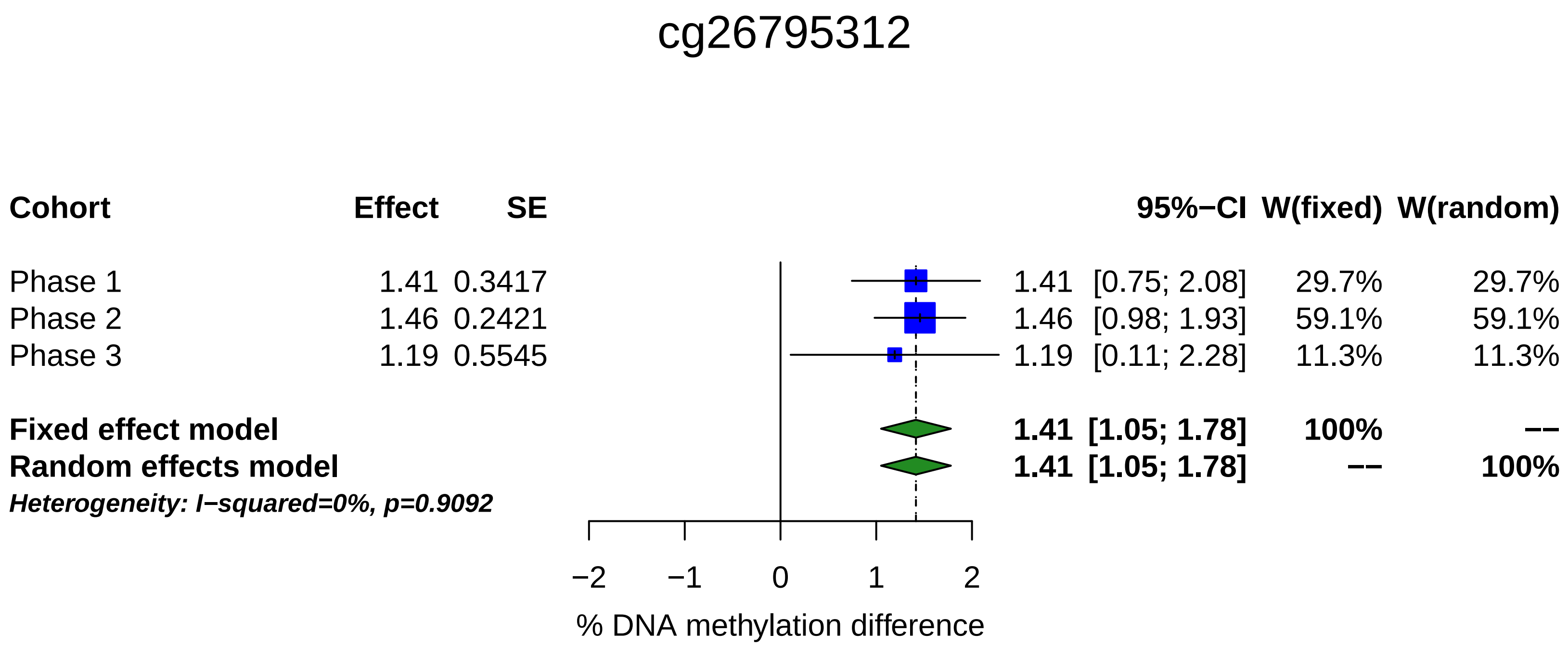
cg26795312
Cohort
Effect
SE
95%−CI
W(fixed)
W(random)
Phase 1 1.41 0.3417 1.41 [0.75; 2.08] 29.7% 29.7%
Phase 2 1.46 0.2421 1.46 [0.98; 1.93] 59.1% 59.1%
Phase 3 1.19 0.5545 1.19 [0.11; 2.28] 11.3% 11.3%
Fixed effect model 1.41 [1.05; 1.78] 100% −−
Random effects model 1.41 [1.05; 1.78] −− 100%
Heterogeneity: I−squared=0%, p=0.9092
−2
−1
0
1
2
% DNA methylation difference

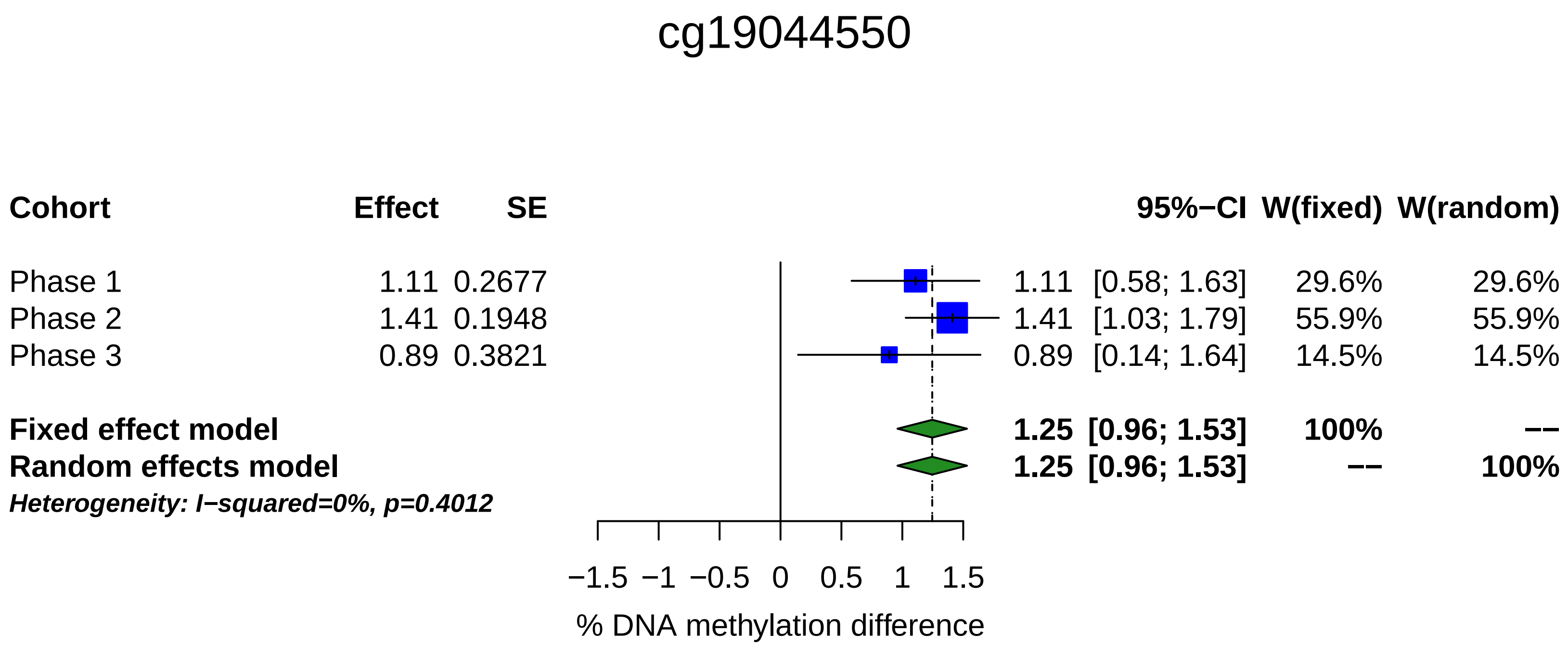
cg19044550
Cohort
Effect
SE
95%−CI
W(fixed)
W(random)
Phase 1 1.11 0.2677 1.11 [0.58; 1.63] 29.6% 29.6%
Phase 2 1.41 0.1948 1.41 [1.03; 1.79] 55.9% 55.9%
Phase 3 0.89 0.3821 0.89 [0.14; 1.64] 14.5% 14.5%
Fixed effect model 1.25 [0.96; 1.53] 100% −−
Random effects model 1.25 [0.96; 1.53] −− 100%
Heterogeneity: I−squared=0%, p=0.4012
−1.5 −1 −0.5 0 0.5 1 1.5
% DNA methylation difference

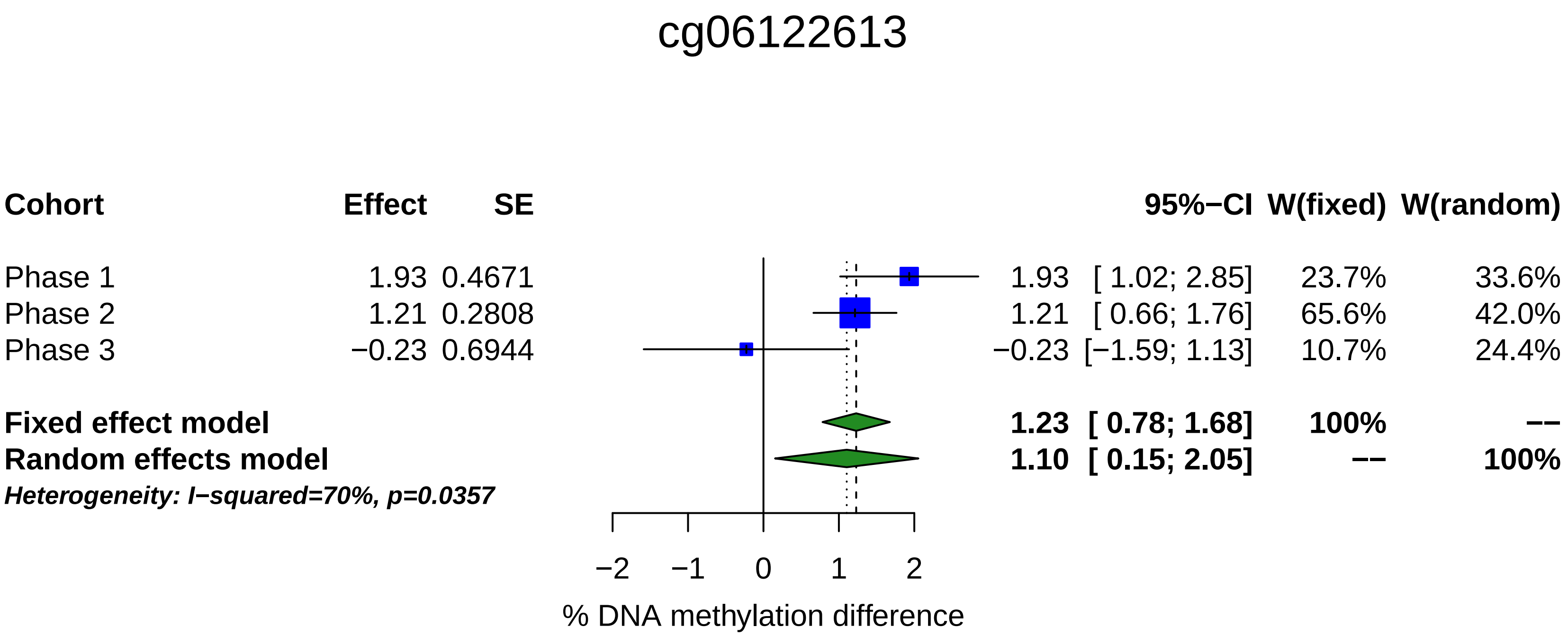

cg06122613
Cohort
Effect
SE
95%−CI
W(fixed)
W(random)
Phase 1 1.93 0.4671 1.93 [1.02; 2.85] 23.7% 33.6%
Phase 2 1.21 0.2808 1.21 [0.66; 1.76] 65.6% 42.0%
Phase 3 −0.23 0.6944 −0.23 [−1.59; 1.13] 10.7% 24.4%
Fixed effect model 1.23 [0.78; 1.68] 100% −−
Random effects model 1.10 [0.15; 2.05] −− 100%
Heterogeneity: I−squared=70%, p=0.0357
−2
−1
0
1
2
% DNA methylation difference

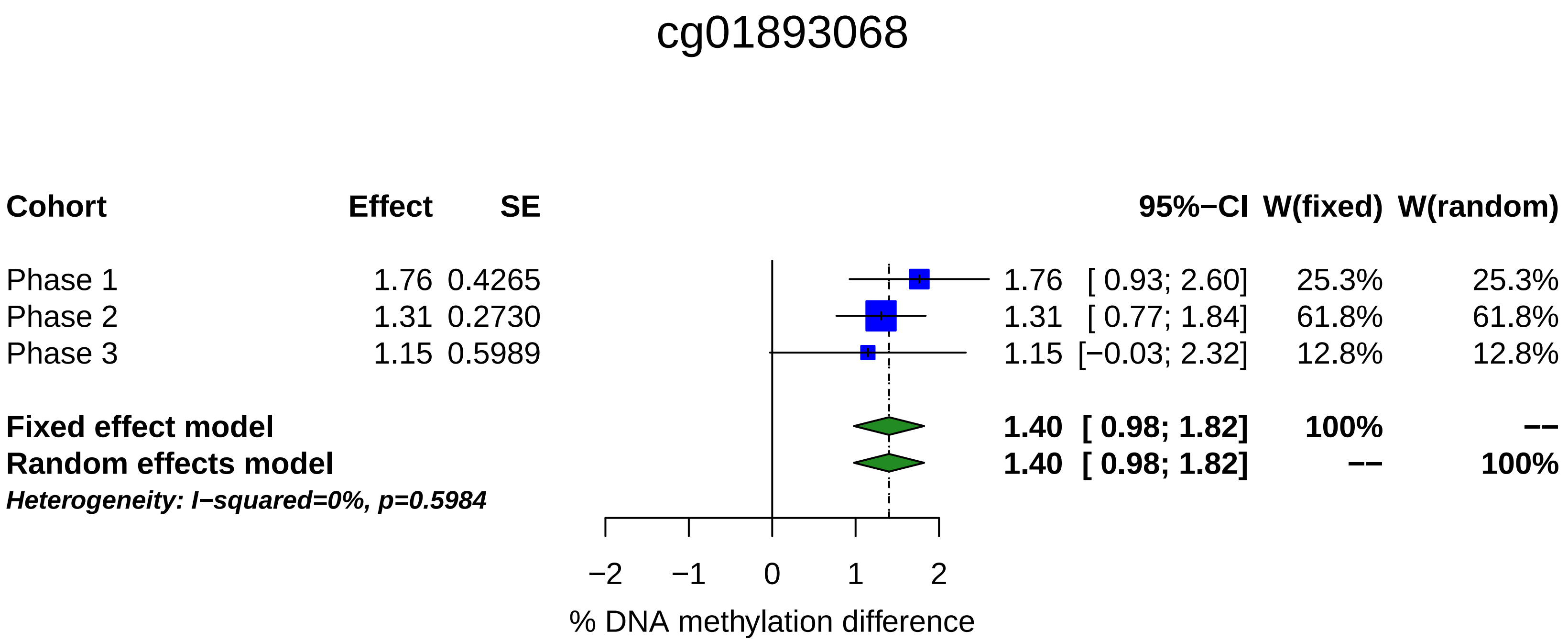
cg01893068
Cohort
Effect
SE
95%−CI
W(fixed)
W(random)
Phase 1 1.76 0.4265 1.76 [0.93; 2.60] 25.3% 25.3%
Phase 2 1.31 0.2730 1.31 [0.77; 1.84] 61.8% 61.8%
Phase 3 1.15 0.5989 1.15 [−0.03; 2.32] 12.8% 12.8%
Fixed effect model 1.40 [0.98; 1.82] 100% −−
Random effects model 1.40 [0.98; 1.82] −− 100%
Heterogeneity: I−squared=0%, p=0.5984
−2
−1
0
1
2
% DNA methylation difference

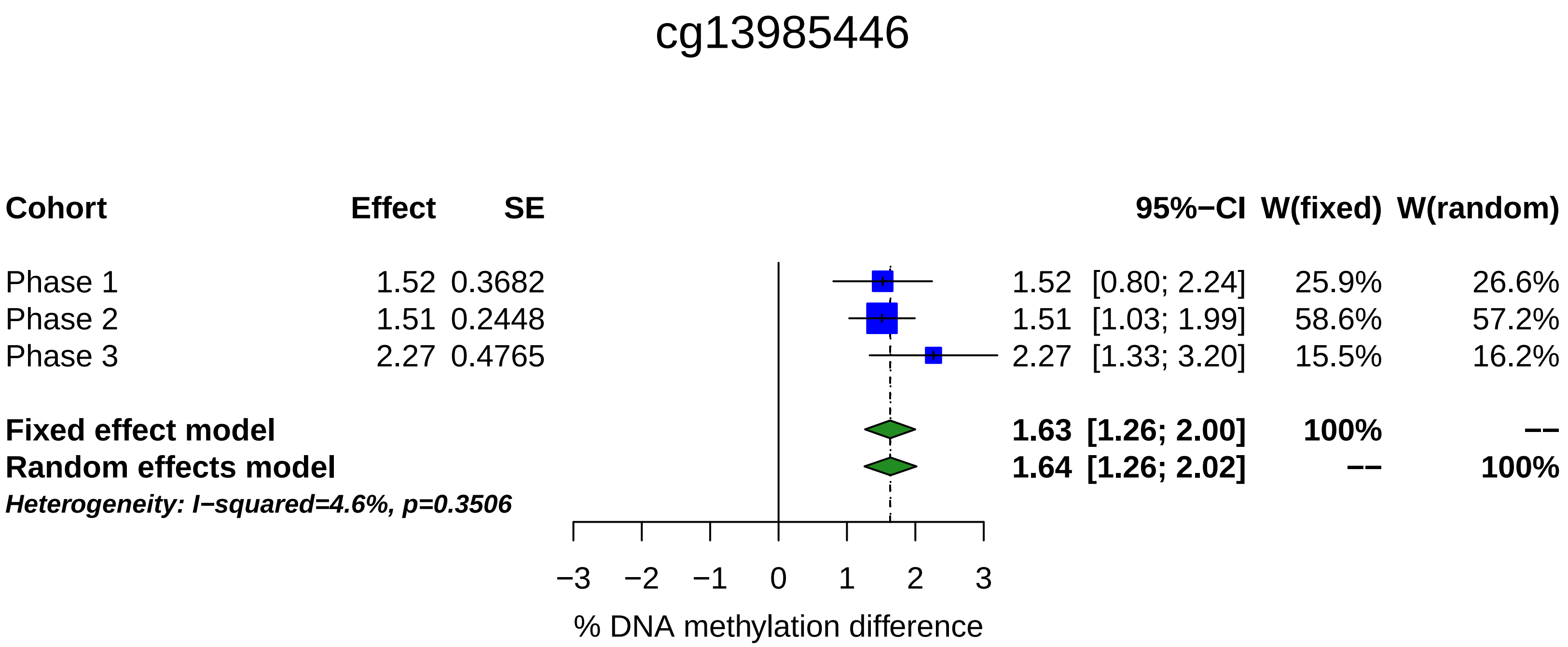

cg13985446
Cohort
Effect
SE
95%−CI
W(fixed)
W(random)
Phase 1
1.52
0.3682
1.52 [0.80; 2.24]
25.9%
26.6%
Phase 2
1.51
0.2448
1.51 [1.03; 1.99]
58.6%
57.2%
Phase 3
2.27
0.4765
2.27 [1.33; 3.20]
15.5%
16.2%
Fixed effect model
1.63 [1.26; 2.00]
100%
−−
Random effects model
1.64 [1.26; 2.02]
−−
100%
Heterogeneity: I−squared=4.6%, p=0.3506
−3
−2
−1
0
1
2
3
% DNA methylation difference

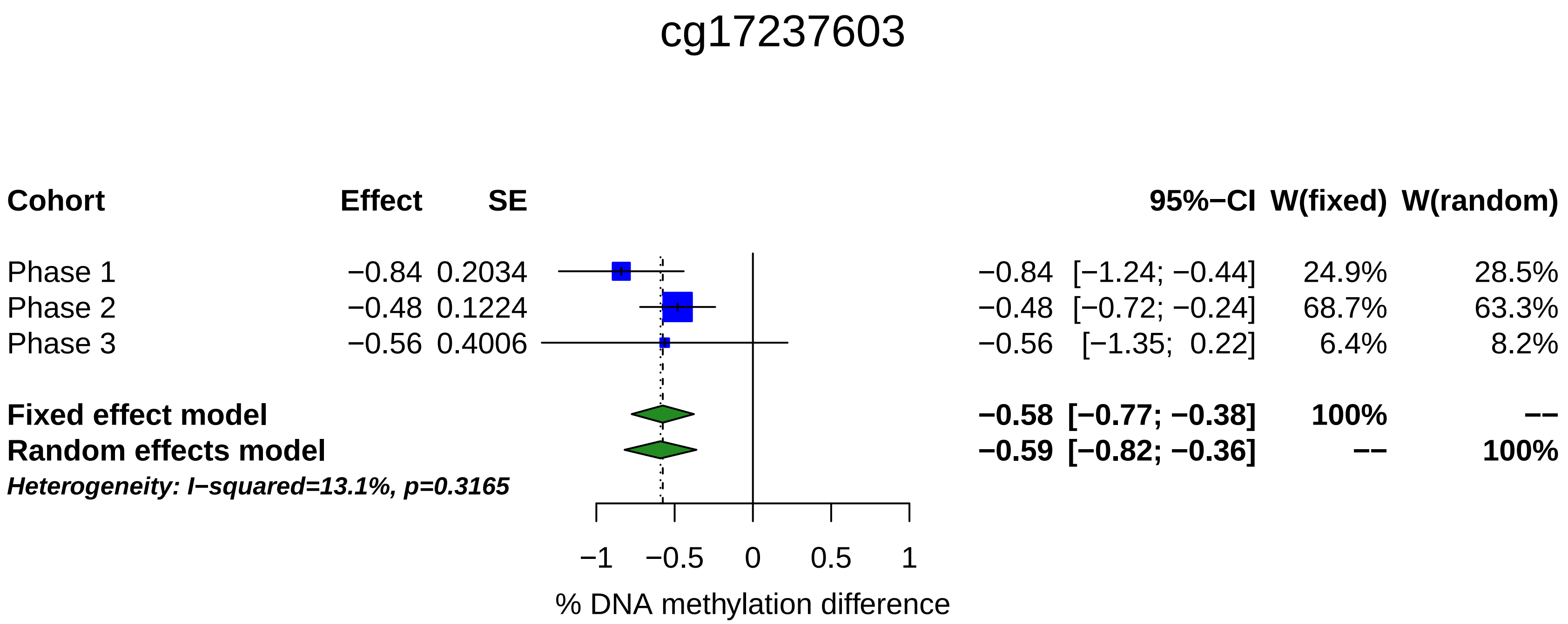
cg17237603
Cohort
Effect
SE
95%−CI
W(fixed)
W(random)
Phase 1 −0.84 0.2034 −0.84 [−1.24; −0.44] 24.9% 28.5%
Phase 2 −0.48 0.1224 −0.48 [−0.72; −0.24] 68.7% 63.3%
Phase 3 −0.56 0.4006 −0.56 [−1.35; 0.22] 6.4% 8.2%
Fixed effect model −0.58 [−0.77; −0.38] 100% −−
Random effects model −0.59 [−0.82; −0.36] −− 100%
Heterogeneity: I−squared=13.1%, p=0.3165
−1
−0.5
0
0.5
1
% DNA methylation difference

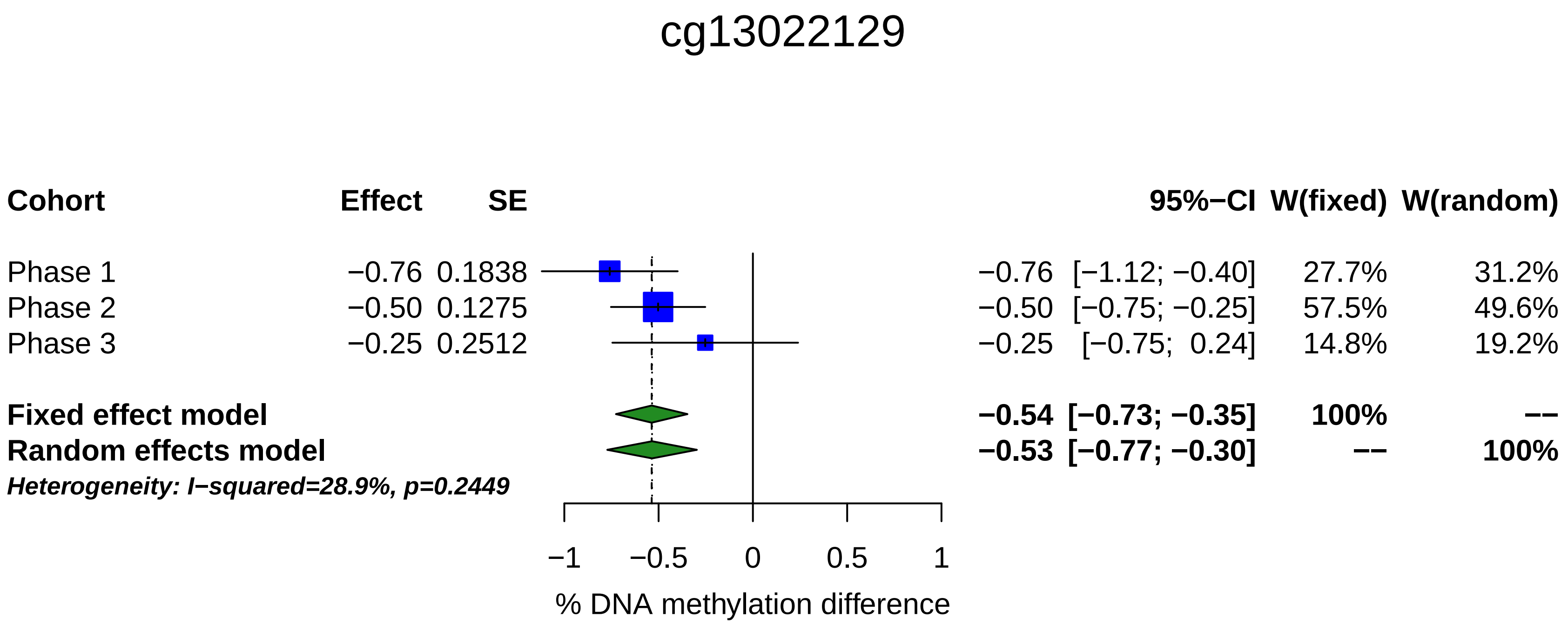
cg13022129
Cohort
Effect
SE
95%−CI
W(fixed)
W(random)
Phase 1 −0.76 0.1838 −0.76 [−1.12; −0.40] 27.7% 31.2%
Phase 2 −0.50 0.1275 −0.50 [−0.75; −0.25] 57.5% 49.6%
Phase 3 −0.25 0.2512 −0.25 [−0.75; 0.24] 14.8% 19.2%
Fixed effect model −0.54 [−0.73; −0.35] 100% −−
Random effects model −0.53 [−0.77; −0.30] −− 100%
Heterogeneity: I−squared=28.9%, p=0.2449
−1
−0.5
0
0.5
1
% DNA methylation difference

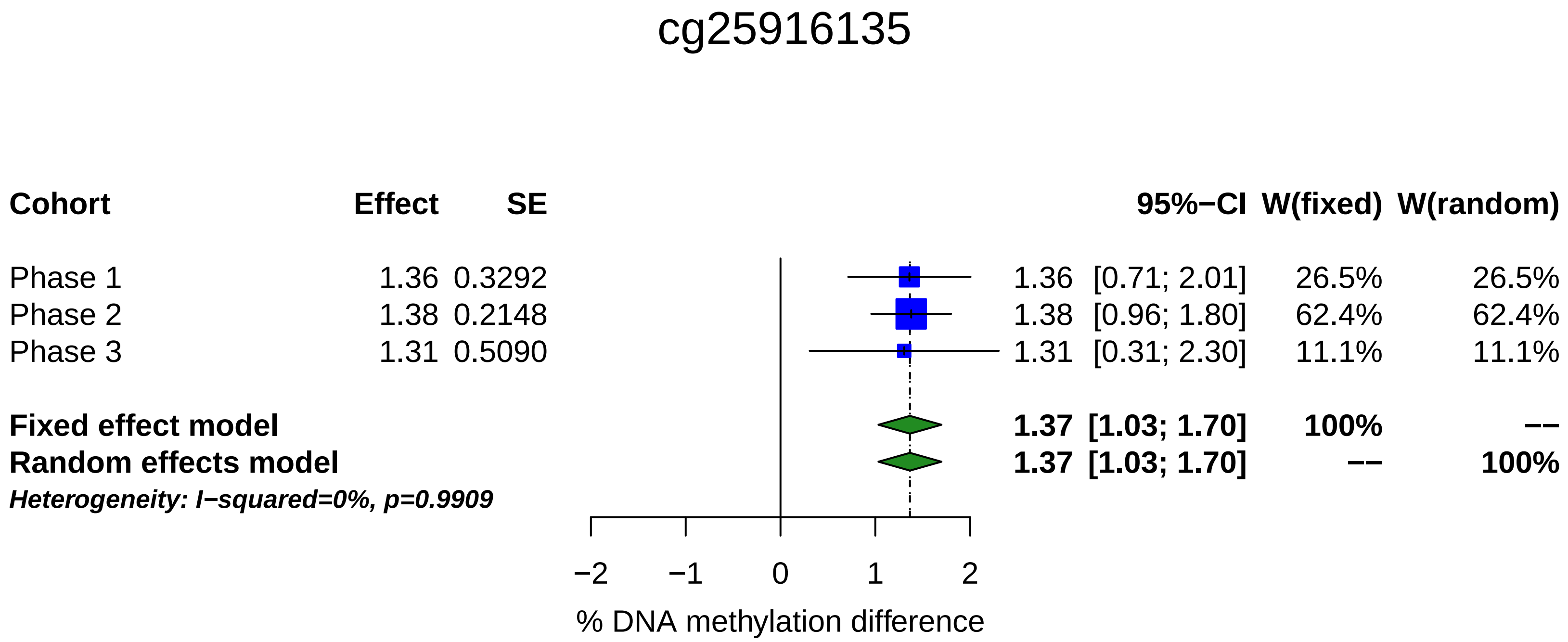
cg25916135
Cohort
Effect
SE
95%−CI
W(fixed)
W(random)
Phase 1
1.36 0.3292
1.36 [0.71; 2.01]
26.5%
26.5%
Phase 2
1.38 0.2148
1.38 [0.96; 1.80]
62.4%
62.4%
Phase 3
1.31 0.5090
1.31 [0.31; 2.30]
11.1%
11.1%
Fixed effect model
1.37 [1.03; 1.70]
100%
−−
Random effects model
1.37 [1.03; 1.70]
−−
100%
Heterogeneity: I−squared=0%, p=0.9909
−2
−1
0
1
2
% DNA methylation difference

cg23777479

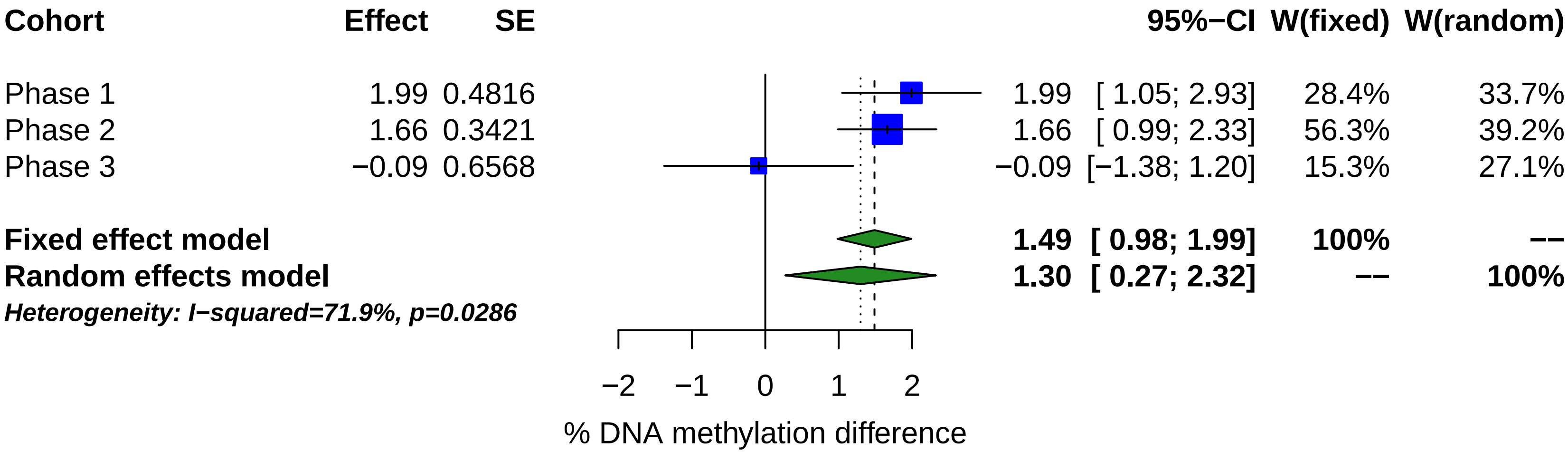
Cohort
Effect
SE
95%−CI
W(fixed)
W(random)
Phase 1
1.99
0.4816
1.99
[1.05; 2.93]
28.4%
33.7%
Phase 2
1.66
0.3421
1.66
[0.99; 2.33]
56.3%
39.2%
Phase 3
−0.09
0.6568
−0.09
[−1.38; 1.20]
15.3%
27.1%
Fixed effect model
1.49
[0.98; 1.99]
100%
−−
Random effects model
1.30
[0.27; 2.32]
−−
100%
Heterogeneity: I−squared=71.9%, p=0.0286
−2
−1
0
1
2
% DNA methylation difference

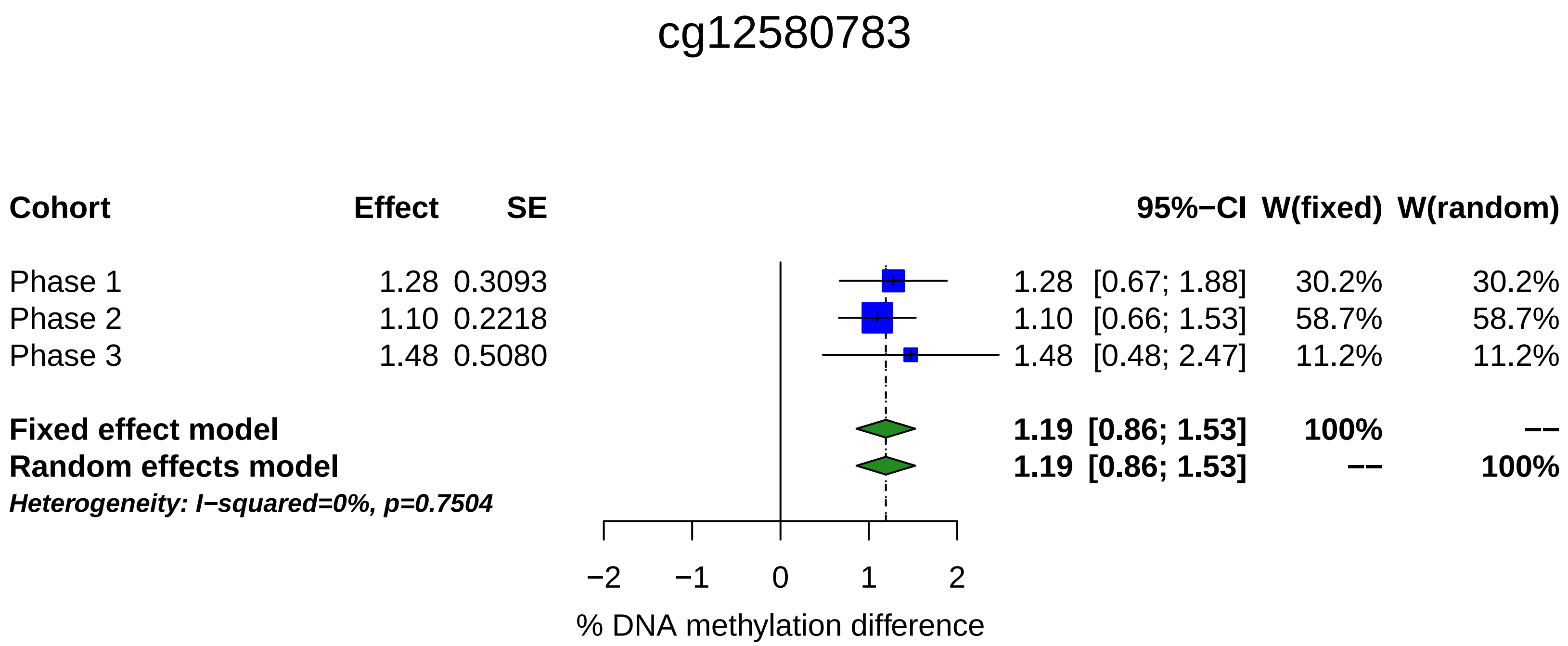
cg12580783
Cohort
Effect
SE
95%−CI
W(fixed)
W(random)
Phase 1 1.28 0.3093 1.28 [0.67; 1.88] 30.2% 30.2%
Phase 2 1.10 0.2218 1.10 [0.66; 1.53] 58.7% 58.7%
Phase 3 1.48 0.5080 1.48 [0.48; 2.47] 11.2% 11.2%
Fixed effect model 1.19 [0.86; 1.53] 100% −−
Random effects model 1.19 [0.86; 1.53] −− 100%
Heterogeneity: I−squared=0%, p=0.7504
−2
−1
0
1
2
% DNA methylation difference

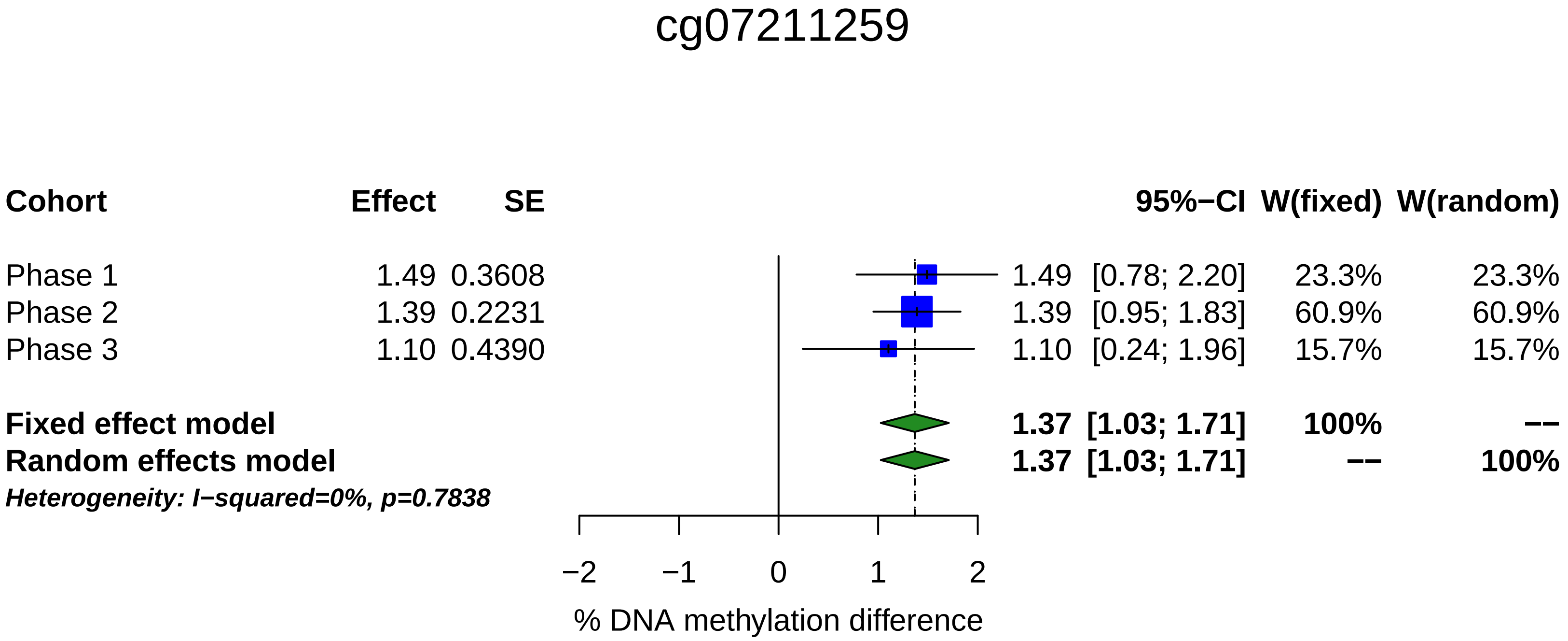
cg07211259
Cohort
Effect
SE
95%−CI
W(fixed)
W(random)
Phase 1
1.49
0.3608
1.49 [0.78; 2.20]
23.3%
23.3%
Phase 2
1.39
0.2231
1.39 [0.95; 1.83]
60.9%
60.9%
Phase 3
1.10
0.4390
1.10 [0.24; 1.96]
15.7%
15.7%
Fixed effect model
1.37 [1.03; 1.71]
100%
−−
Random effects model
1.37 [1.03; 1.71]
−−
100%
Heterogeneity: I−squared=0%, p=0.7838
−2
−1
0
1
2
% DNA methylation difference

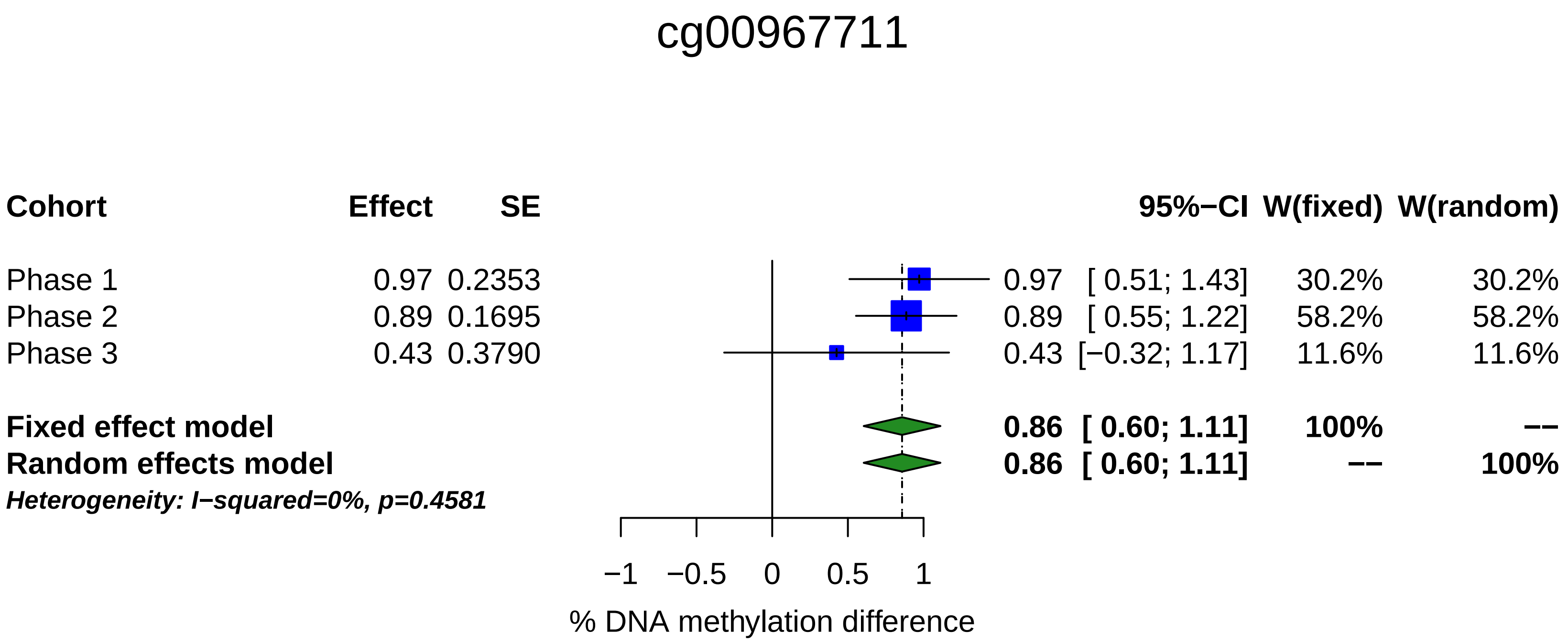
cg00967711
Cohort
Effect
SE
95%−CI
W(fixed)
W(random)
Phase 1
0.97
0.2353
0.97 [0.51; 1.43]
30.2%
30.2%
Phase 2
0.89
0.1695
0.89 [0.55; 1.22]
58.2%
58.2%
Phase 3
0.43
0.3790
0.43 [−0.32; 1.17]
11.6%
11.6%
Fixed effect model
0.86 [0.60; 1.11]
100%
−−
Random effects model
0.86 [0.60; 1.11]
−−
100%
Heterogeneity: I−squared=0%, p=0.4581
−1
−0.5
0
0.5
1
% DNA methylation difference

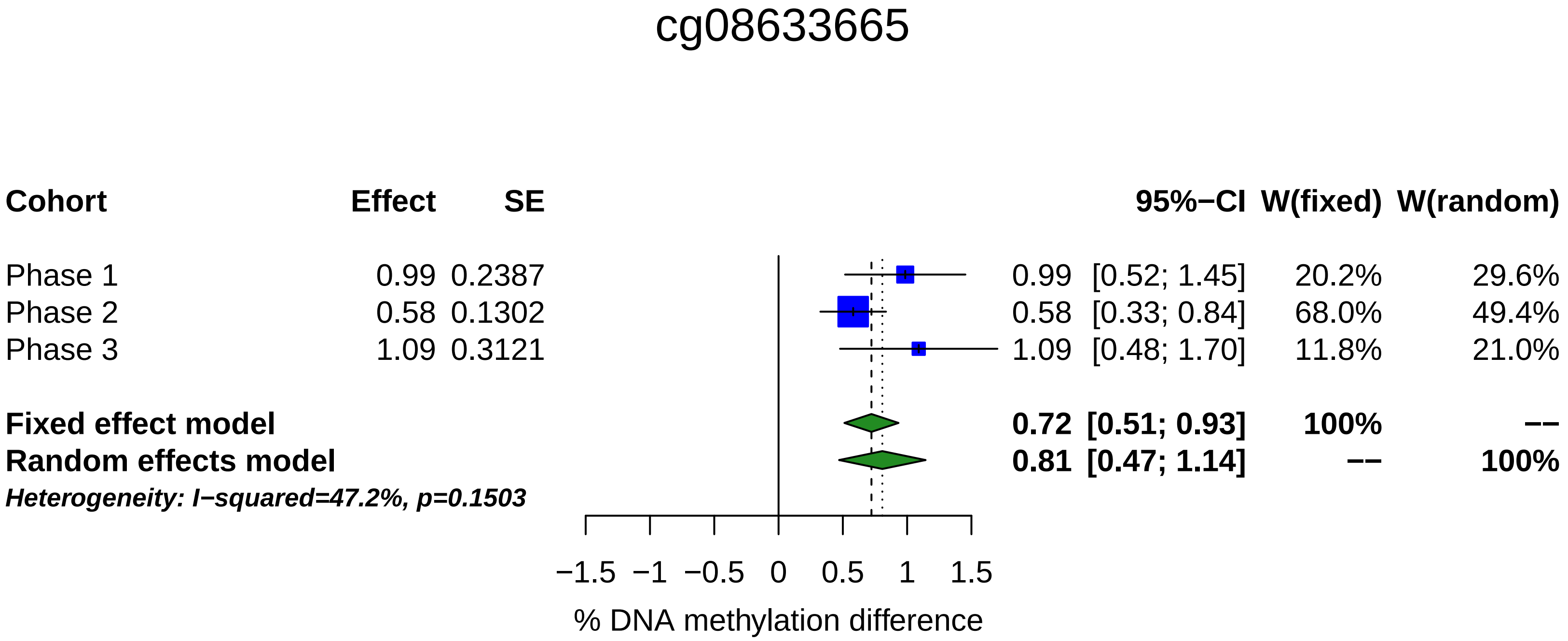
cg08633665
Cohort
Effect
SE
95%−CI
W(fixed)
W(random)
Phase 1 0.99 0.2387 0.99 [0.52; 1.45] 20.2% 29.6%
Phase 2 0.58 0.1302 0.58 [0.33; 0.84] 68.0% 49.4%
Phase 3 1.09 0.3121 1.09 [0.48; 1.70] 11.8% 21.0%
Fixed effect model 0.72 [0.51; 0.93] 100% −−
Random effects model 0.81 [0.47; 1.14] −− 100%
Heterogeneity: I−squared=47.2%, p=0.1503
−1.5
−1
−0.5
0
0.5
1
1.5
% DNA methylation difference

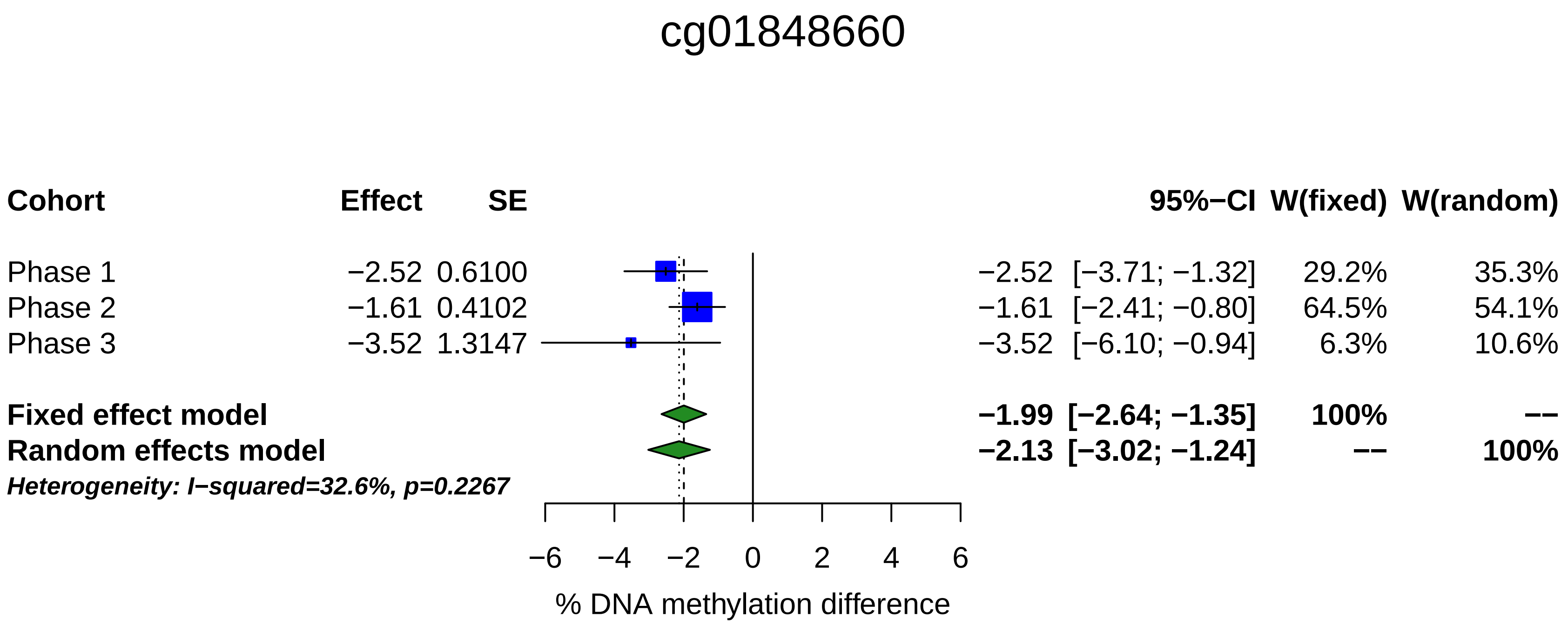
cg01848660
Cohort
Effect
SE
95%−CI
W(fixed)
W(random)
Phase 1 −2.52 0.6100 −2.52 [−3.71; −1.32] 29.2% 35.3%
Phase 2 −1.61 0.4102 −1.61 [−2.41; −0.80] 64.5% 54.1%
Phase 3 −3.52 1.3147 −3.52 [−6.10; −0.94] 6.3% 10.6%
Fixed effect model −1.99 [−2.64; −1.35] 100% −−
Random effects model −2.13 [−3.02; −1.24] −− 100%
Heterogeneity: I−squared=32.6%, p=0.2267
−6
−4
−2
0
2
4
6
% DNA methylation difference

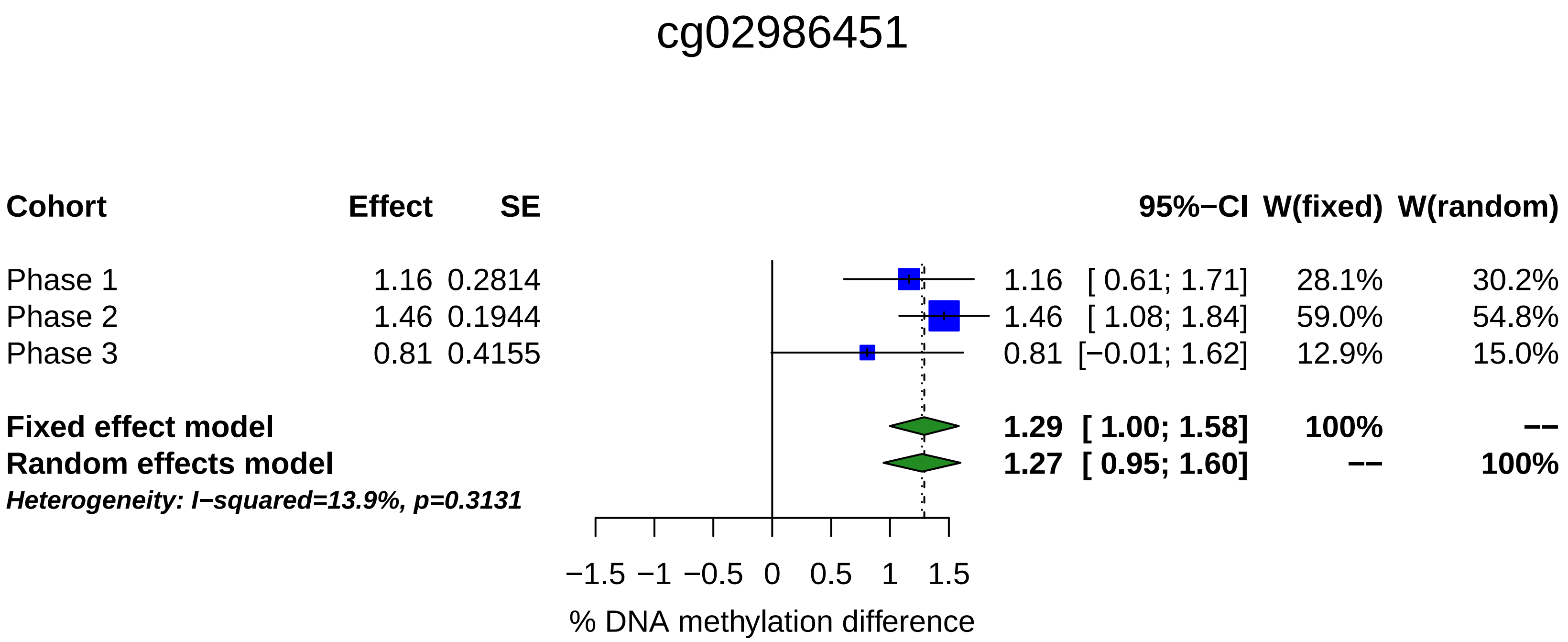
cg02986451
Cohort
Effect
SE
95%−CI
W(fixed)
W(random)
Phase 1 1.16 0.2814 1.16 [0.61; 1.71] 28.1% 30.2%
Phase 2 1.46 0.1944 1.46 [1.08; 1.84] 59.0% 54.8%
Phase 3 0.81 0.4155 0.81 [−0.01; 1.62] 12.9% 15.0%
Fixed effect model 1.29 [1.00; 1.58] 100% --
Random effects model 1.27 [0.95; 1.60] -- 100%
Heterogeneity: I−squared=13.9%, p=0.3131
−1.5 −1 −0.5 0 0.5 1 1.5
% DNA methylation difference

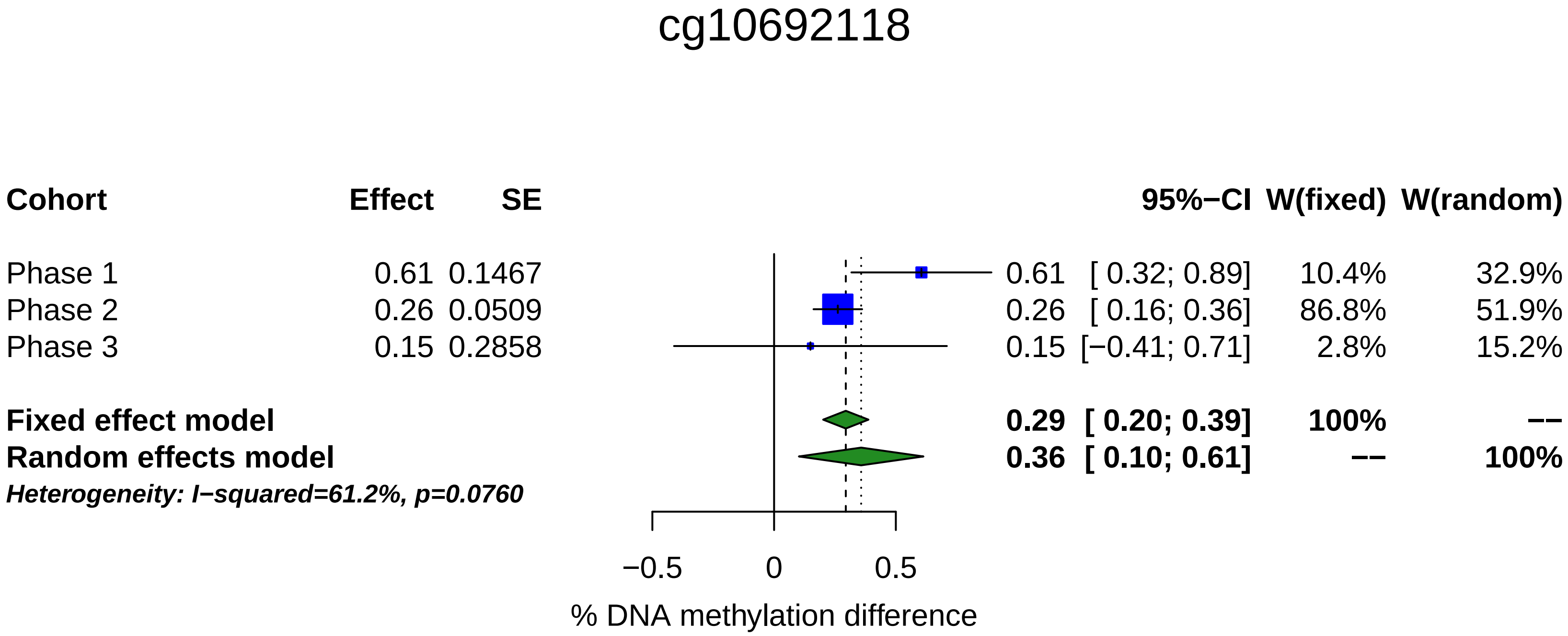
cg10692118
Cohort
Effect
SE
95%−CI
W(fixed)
W(random)
Phase 1 0.61 0.1467 0.61 [0.32; 0.89] 10.4% 32.9%
Phase 2 0.26 0.0509 0.26 [0.16; 0.36] 86.8% 51.9%
Phase 3 0.15 0.2858 0.15 [−0.41; 0.71] 2.8% 15.2%
Fixed effect model 0.29 [0.20; 0.39] 100% −−
Random effects model 0.36 [0.10; 0.61] −− 100%
Heterogeneity: I−squared=61.2%, p=0.0760
−0.5
0
0.5
% DNA methylation difference

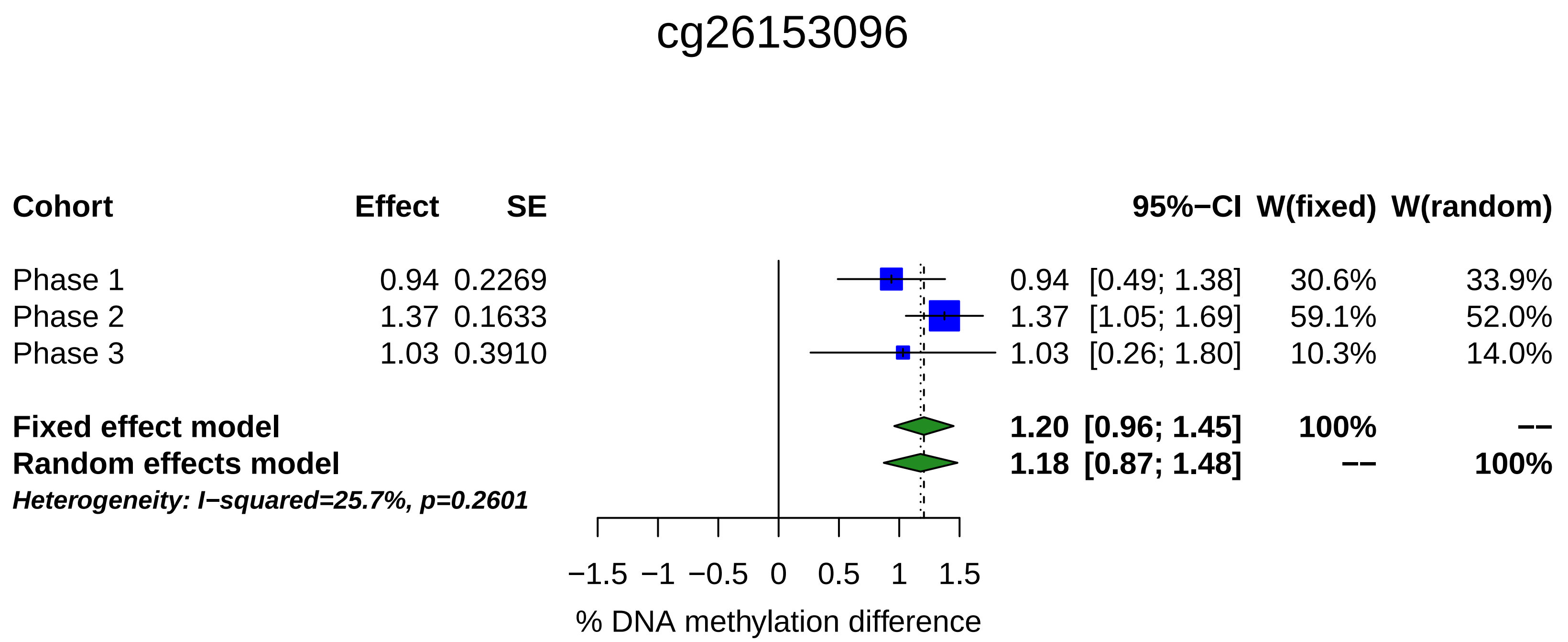
cg26153096
Cohort
Effect
SE
95%−CI
W(fixed)
W(random)
Phase 1
0.94
0.2269
0.94 [0.49; 1.38]
30.6%
33.9%
Phase 2
1.37
0.1633
1.37 [1.05; 1.69]
59.1%
52.0%
Phase 3
1.03
0.3910
1.03 [0.26; 1.80]
10.3%
14.0%
Fixed effect model
1.20 [0.96; 1.45]
100%
−−
Random effects model
1.18 [0.87; 1.48]
−−
100%
Heterogeneity: I−squared=25.7%, p=0.2601
−1.5
−1
−0.5
0
0.5
1
1.5
% DNA methylation difference

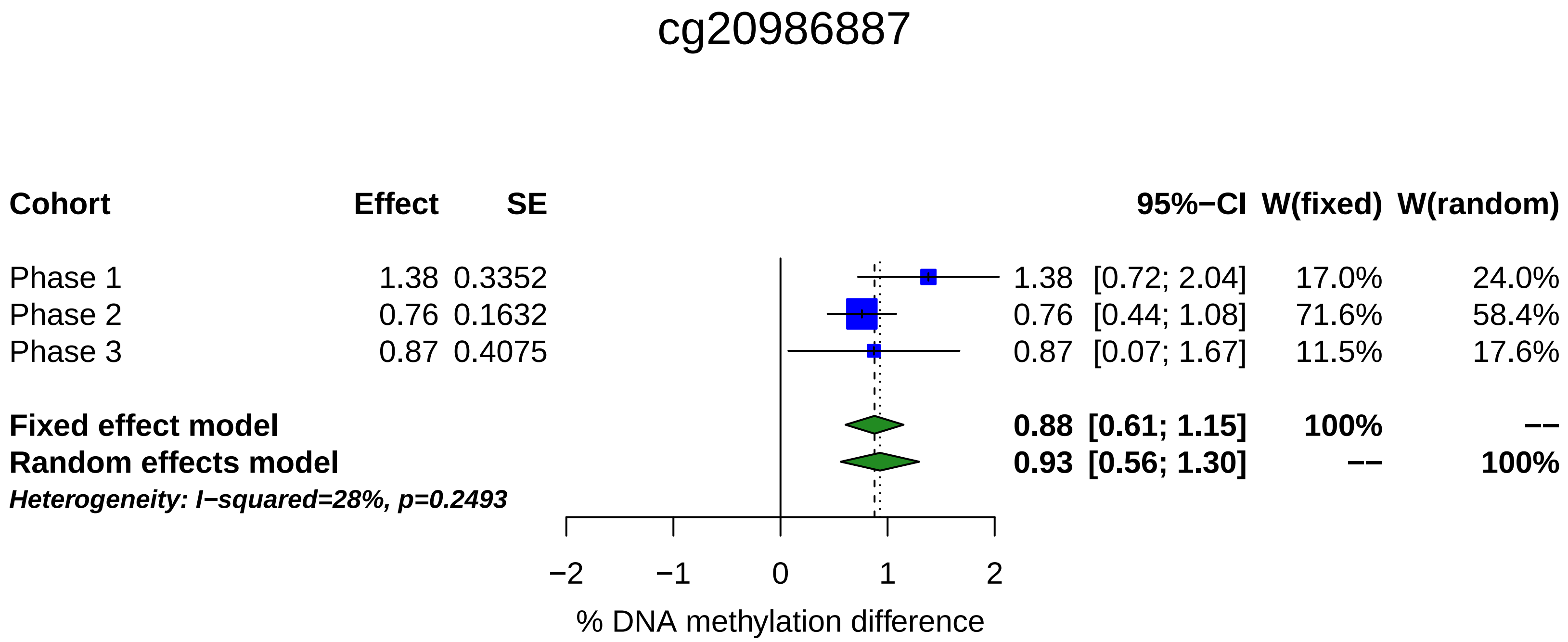
cg20986887
Cohort Effect SE 95%−CI W(fixed) W(random)
Phase 1 1.38 0.3352 1.38 [0.72; 2.04] 17.0% 24.0%
Phase 2 0.76 0.1632 0.76 [0.44; 1.08] 71.6% 58.4%
Phase 3 0.87 0.4075 0.87 [0.07; 1.67] 11.5% 17.6%
Fixed effect model 0.88 [0.61; 1.15] 100% −−
Random effects model 0.93 [0.56; 1.30] −− 100%
Heterogeneity: I−squared=28%, p=0.2493
−2 −1 0 1 2
% DNA methylation difference

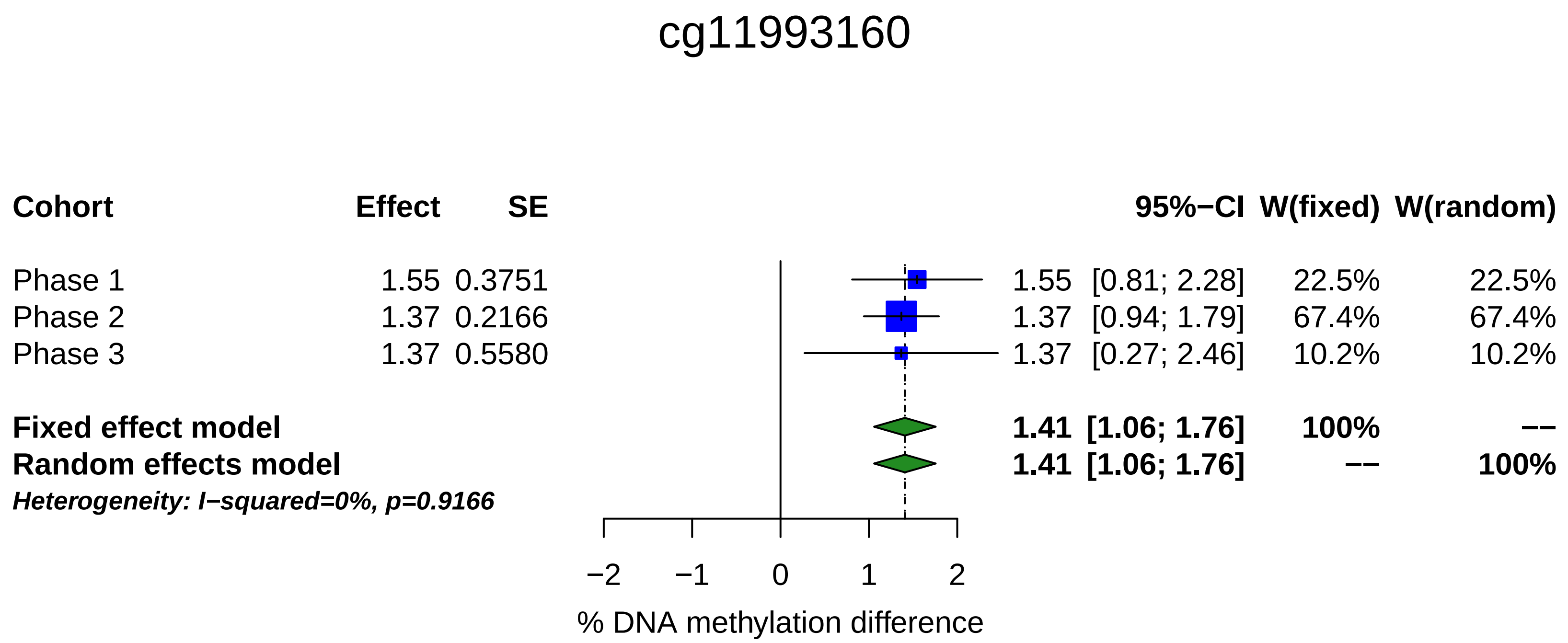
cg11993160
Cohort
Effect
SE
95%−CI
W(fixed)
W(random)
Phase 1 1.55 0.3751 1.55 [0.81; 2.28] 22.5% 22.5%
Phase 2 1.37 0.2166 1.37 [0.94; 1.79] 67.4% 67.4%
Phase 3 1.37 0.5580 1.37 [0.27; 2.46] 10.2% 10.2%
Fixed effect model 1.41 [1.06; 1.76] 100% −−
Random effects model 1.41 [1.06; 1.76] −− 100%
Heterogeneity: I−squared=0%, p=0.9166
−2
−1
0
1
2
% DNA methylation difference

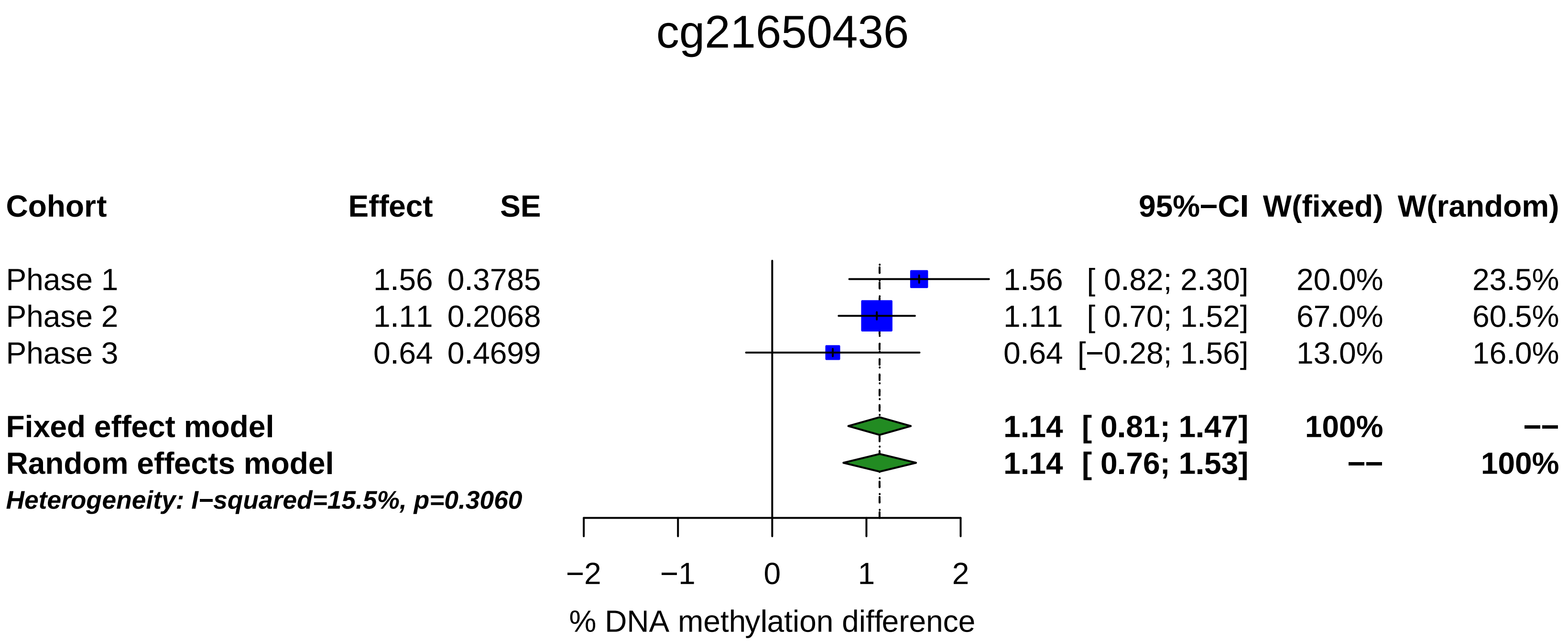
cg21650436
Cohort
Effect
SE
95%−CI
W(fixed)
W(random)
Phase 1 1.56 0.3785 1.56 [0.82; 2.30] 20.0% 23.5%
Phase 2 1.11 0.2068 1.11 [0.70; 1.52] 67.0% 60.5%
Phase 3 0.64 0.4699 0.64 [−0.28; 1.56] 13.0% 16.0%
Fixed effect model 1.14 [0.81; 1.47] 100% −−
Random effects model 1.14 [0.76; 1.53] −− 100%
Heterogeneity: I−squared=15.5%, p=0.3060
−2
−1
0
1
2
% DNA methylation difference

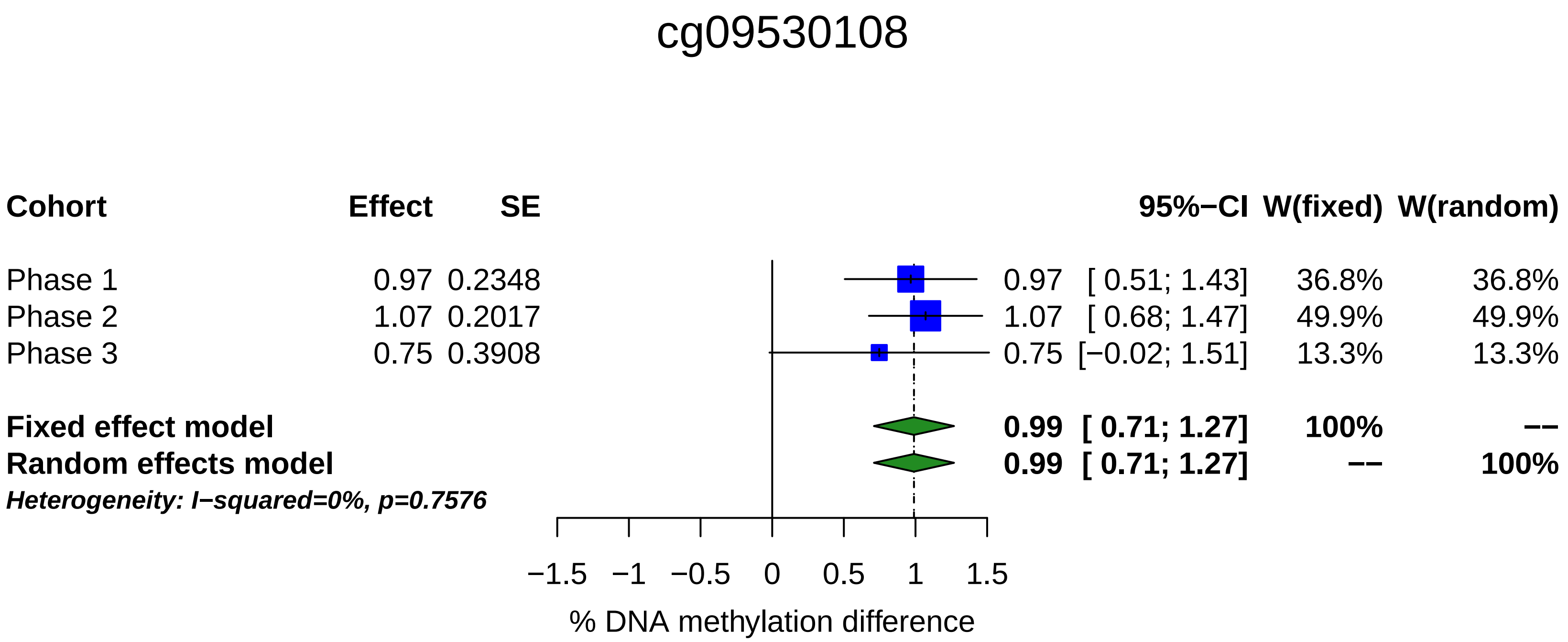
cg09530108
Cohort
Effect
SE
95%−CI
W(fixed)
W(random)
Phase 1 0.97 0.2348 0.97 [0.51; 1.43] 36.8% 36.8%
Phase 2 1.07 0.2017 1.07 [0.68; 1.47] 49.9% 49.9%
Phase 3 0.75 0.3908 0.75 [−0.02; 1.51] 13.3% 13.3%
Fixed effect model 0.99 [0.71; 1.27] 100% −−
Random effects model 0.99 [0.71; 1.27] −− 100%
Heterogeneity: I−squared=0%, p=0.7576
−1.5
−1
−0.5
0
0.5
1
1.5
% DNA methylation difference

cg13670091

Cohort	Effect	SE	95%−CI	W(fixed)	W(random)
Phase 1	1.34	0.3246	1.34 [0.70; 1.97]	28.1%	28.9%
Phase 2	0.82	0.2251	0.82 [0.38; 1.26]	58.4%	56.7%
Phase 3	0.67	0.4675	0.67 [−0.25; 1.59]	13.5%	14.4%
**Fixed effect model**			**0.94 [0.61; 1.28]**	**100%**	**−−**
**Random effects model**			**0.95 [0.59; 1.30]**	**−−**	**100%**

Heterogeneity: I−squared=5.7%, p=0.3463

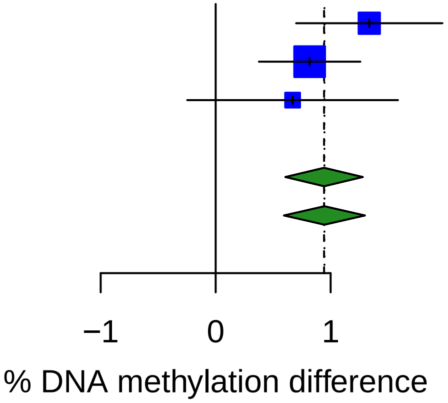

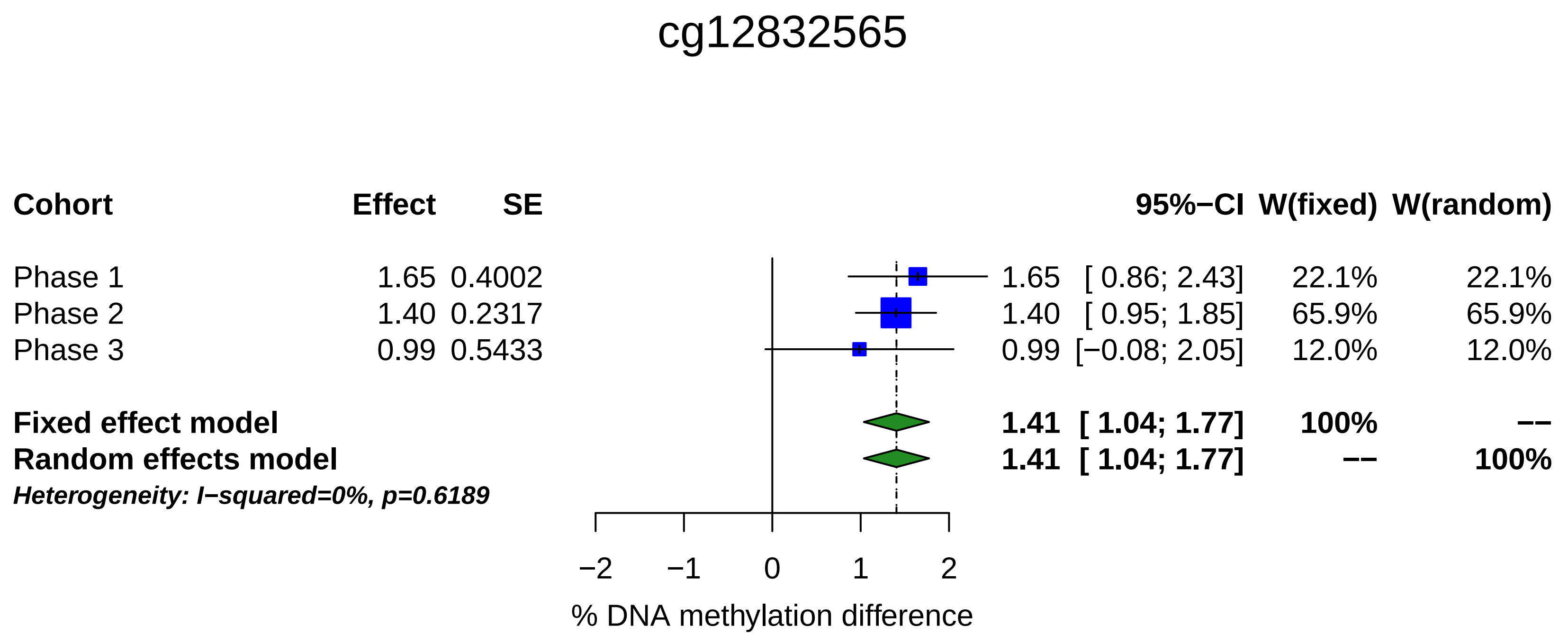
cg12832565
Cohort
Effect
SE
95%−CI
W(fixed)
W(random)
Phase 1 1.65 0.4002 1.65 [0.86; 2.43] 22.1% 22.1%
Phase 2 1.40 0.2317 1.40 [0.95; 1.85] 65.9% 65.9%
Phase 3 0.99 0.5433 0.99 [−0.08; 2.05] 12.0% 12.0%
Fixed effect model 1.41 [1.04; 1.77] 100% −−
Random effects model 1.41 [1.04; 1.77] −− 100%
Heterogeneity: I−squared=0%, p=0.6189
−2
−1
0
1
2
% DNA methylation difference

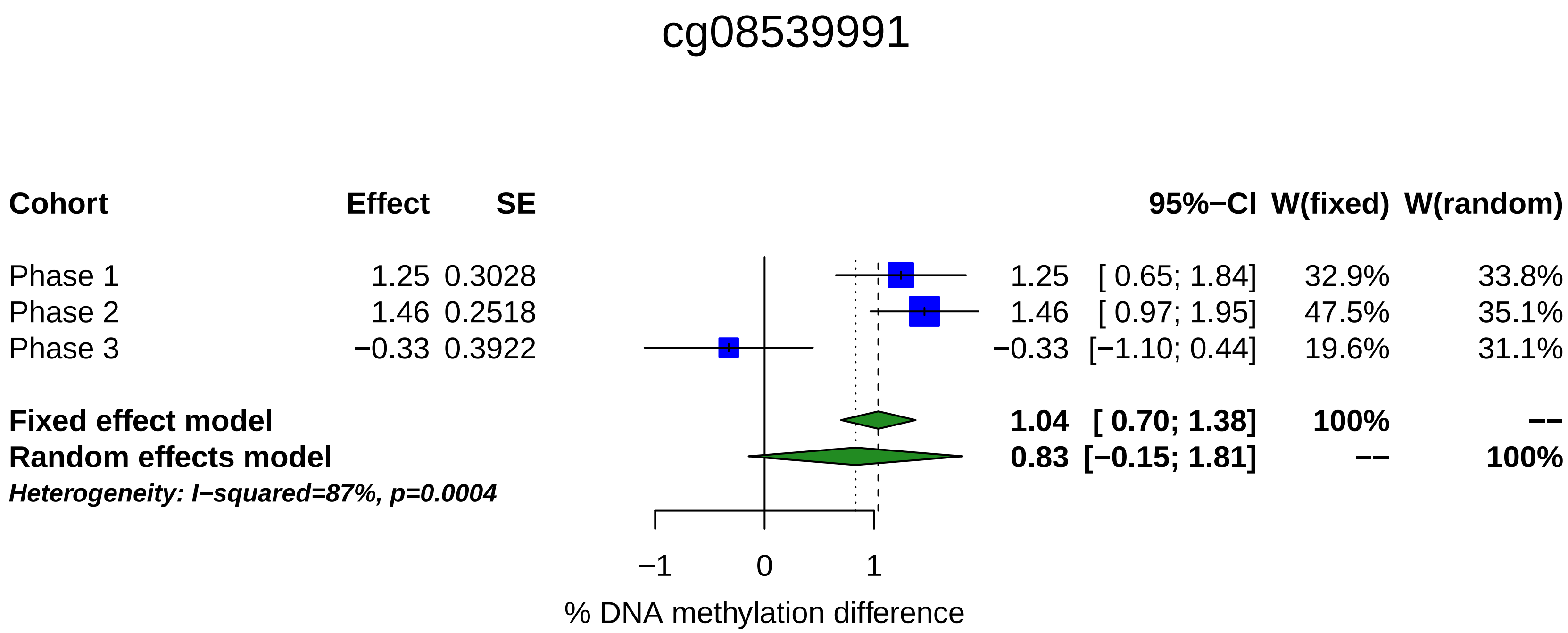
cg08539991
Cohort
Effect
SE
95%−CI
W(fixed)
W(random)
Phase 1 1.25 0.3028 1.25 [0.65; 1.84] 32.9% 33.8%
Phase 2 1.46 0.2518 1.46 [0.97; 1.95] 47.5% 35.1%
Phase 3 −0.33 0.3922 −0.33 [−1.10; 0.44] 19.6% 31.1%
Fixed effect model 1.04 [0.70; 1.38] 100% −−
Random effects model 0.83 [−0.15; 1.81] −− 100%
Heterogeneity: I−squared=87%, p=0.0004
−1
0
1
% DNA methylation difference

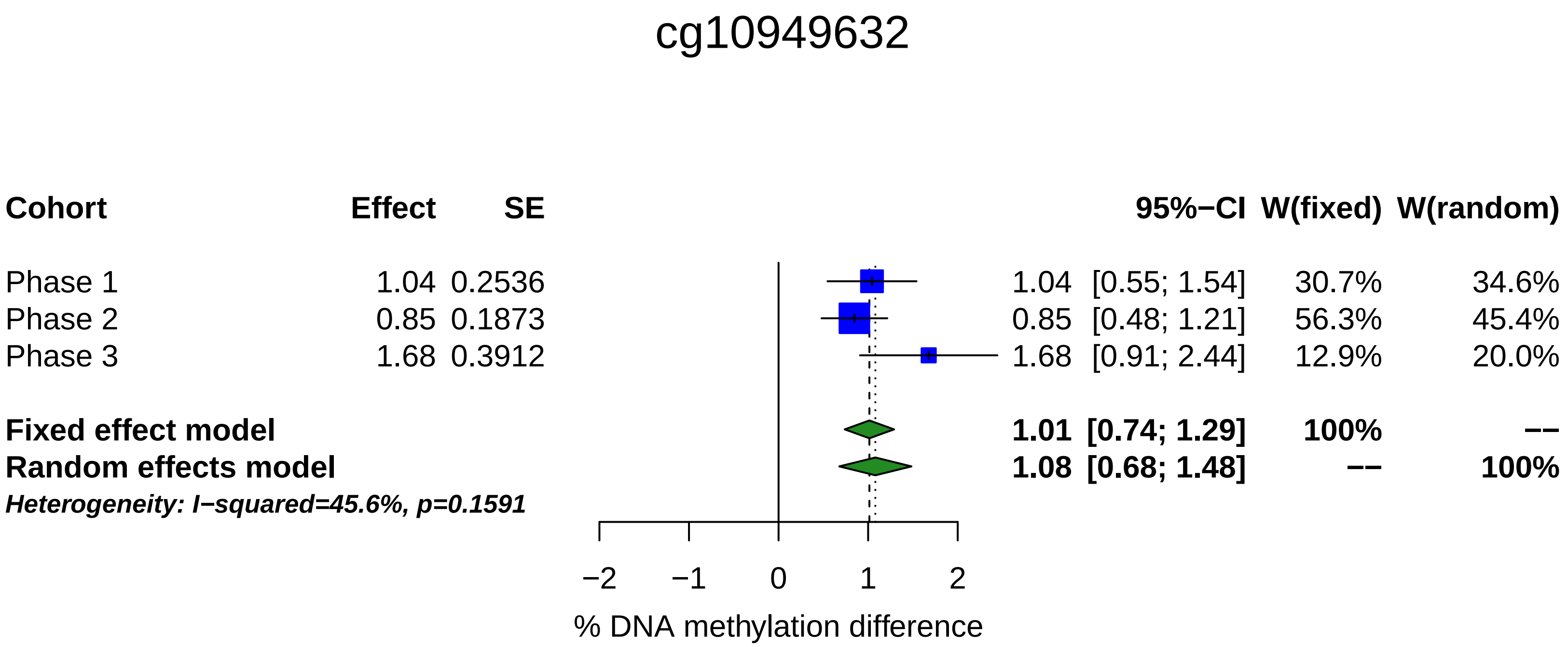
cg10949632
Cohort
Effect
SE
95%−CI
W(fixed)
W(random)
Phase 1 1.04 0.2536 1.04 [0.55; 1.54] 30.7% 34.6%
Phase 2 0.85 0.1873 0.85 [0.48; 1.21] 56.3% 45.4%
Phase 3 1.68 0.3912 1.68 [0.91; 2.44] 12.9% 20.0%
Fixed effect model 1.01 [0.74; 1.29] 100% −−
Random effects model 1.08 [0.68; 1.48] −− 100%
Heterogeneity: I−squared=45.6%, p=0.1591
−2
−1
0
1
2
% DNA methylation difference

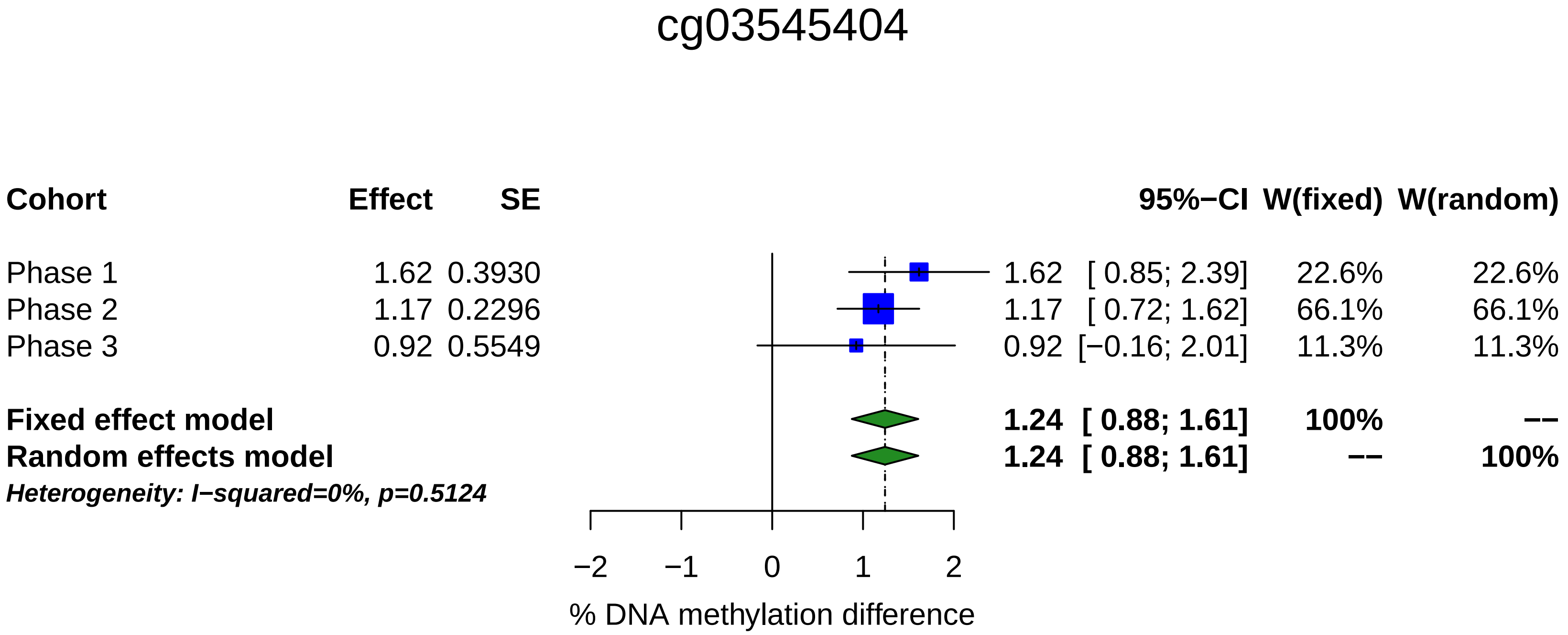
cg03545404
Cohort
Effect
SE
95%−CI
W(fixed)
W(random)
Phase 1 1.62 0.3930 1.62 [0.85; 2.39] 22.6% 22.6%
Phase 2 1.17 0.2296 1.17 [0.72; 1.62] 66.1% 66.1%
Phase 3 0.92 0.5549 0.92 [−0.16; 2.01] 11.3% 11.3%
Fixed effect model 1.24 [0.88; 1.61] 100% −−
Random effects model 1.24 [0.88; 1.61] −− 100%
Heterogeneity: I−squared=0%, p=0.5124
−2
−1
0
1
2
% DNA methylation difference

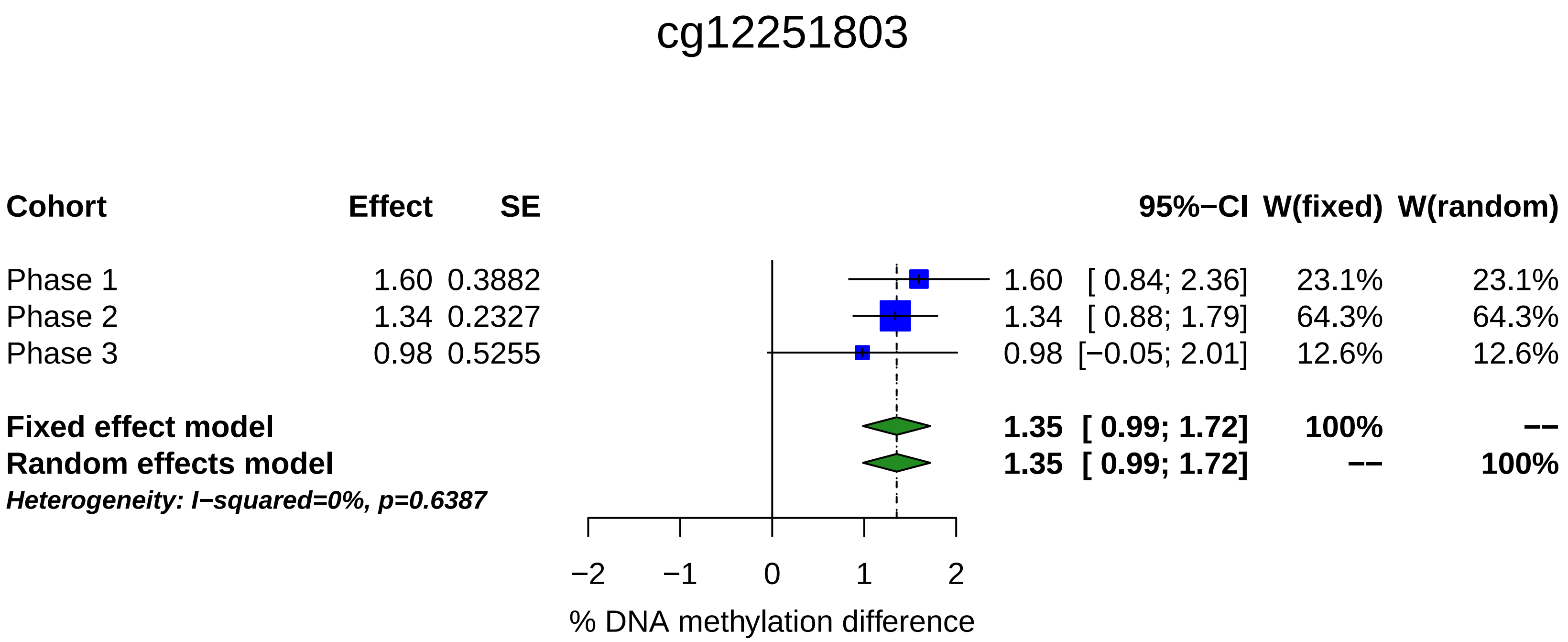
cg12251803
Cohort Effect SE 95%−CI W(fixed) W(random)
Phase 1 1.60 0.3882 1.60 [0.84; 2.36] 23.1% 23.1%
Phase 2 1.34 0.2327 1.34 [0.88; 1.79] 64.3% 64.3%
Phase 3 0.98 0.5255 0.98 [−0.05; 2.01] 12.6% 12.6%
Fixed effect model 1.35 [0.99; 1.72] 100% −−
Random effects model 1.35 [0.99; 1.72] −− 100%
Heterogeneity: I−squared=0%, p=0.6387
−2 −1 0 1 2
% DNA methylation difference

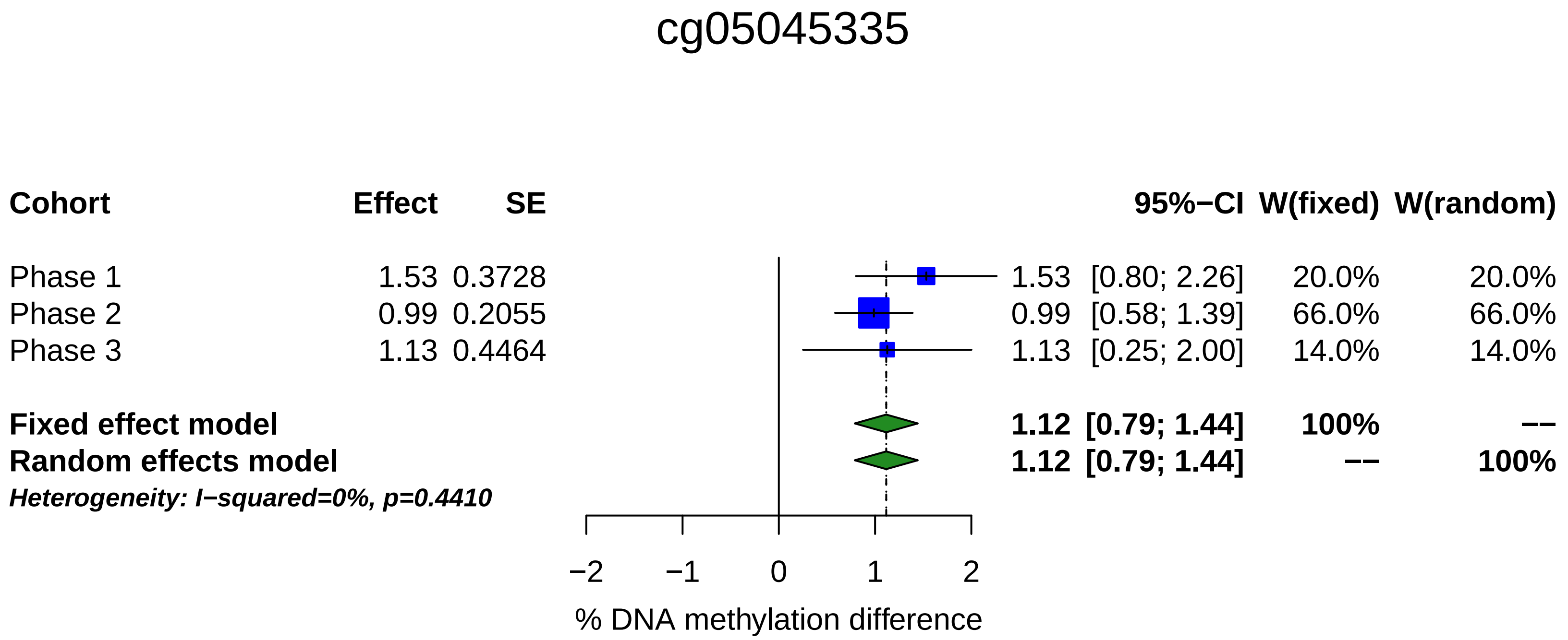
cg05045335
Cohort
Effect
SE
95%−CI
W(fixed)
W(random)
Phase 1 1.53 0.3728 1.53 [0.80; 2.26] 20.0% 20.0%
Phase 2 0.99 0.2055 0.99 [0.58; 1.39] 66.0% 66.0%
Phase 3 1.13 0.4464 1.13 [0.25; 2.00] 14.0% 14.0%
Fixed effect model 1.12 [0.79; 1.44] 100% −−
Random effects model 1.12 [0.79; 1.44] −− 100%
Heterogeneity: I−squared=0%, p=0.4410
−2
−1
0
1
2
% DNA methylation difference

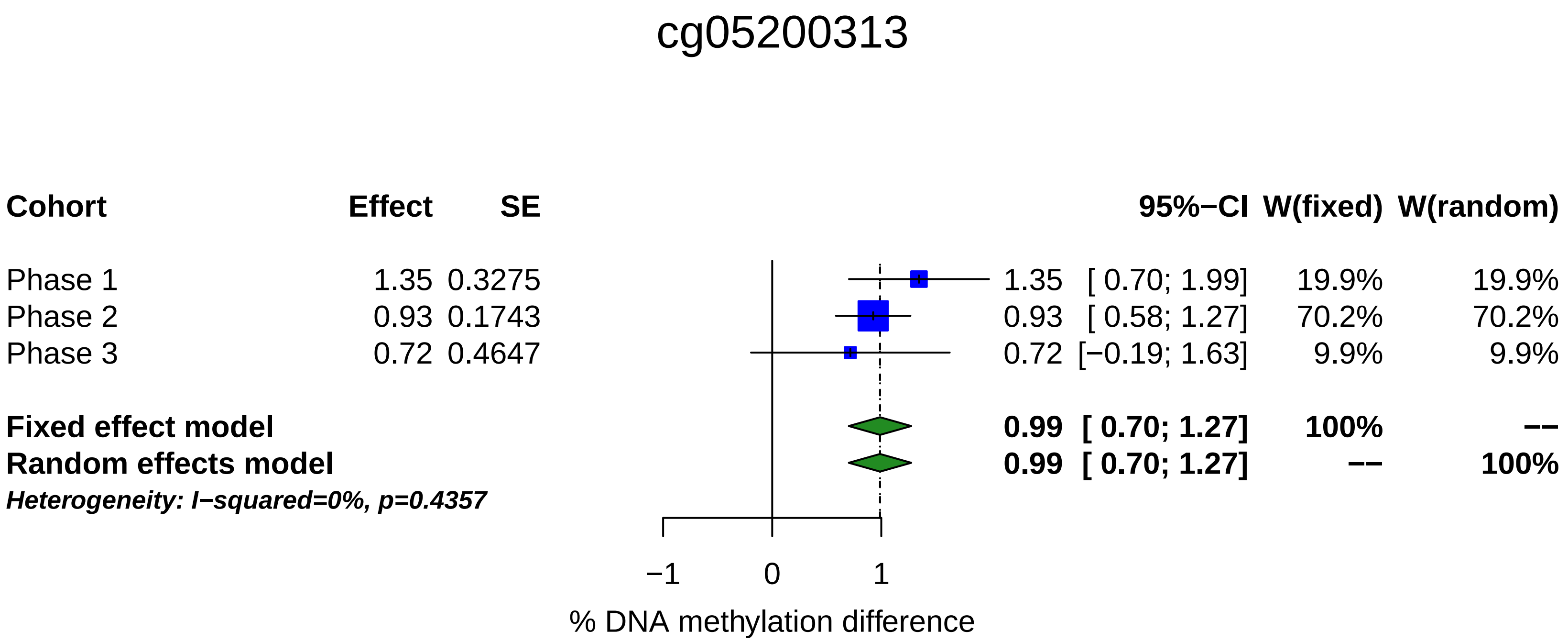
cg05200313
Cohort
Effect
SE
95%−CI
W(fixed)
W(random)
Phase 1
1.35
0.3275
1.35 [0.70; 1.99]
19.9%
19.9%
Phase 2
0.93
0.1743
0.93 [0.58; 1.27]
70.2%
70.2%
Phase 3
0.72
0.4647
0.72 [−0.19; 1.63]
9.9%
9.9%
Fixed effect model
0.99 [0.70; 1.27]
100%
−−
Random effects model
0.99 [0.70; 1.27]
−−
100%
Heterogeneity: I−squared=0%, p=0.4357
−1
0
1
% DNA methylation difference

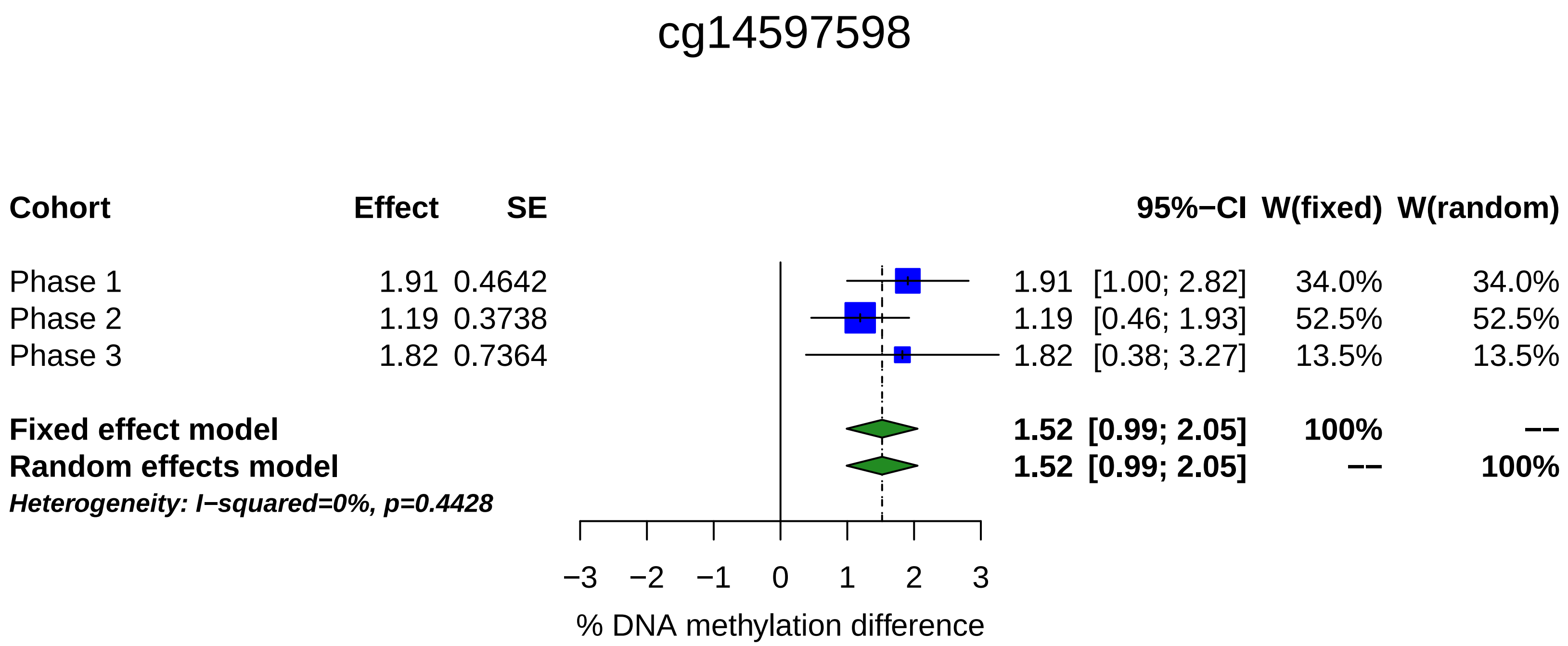
cg14597598
Cohort Effect SE 95%−CI W(fixed) W(random)
Phase 1 1.91 0.4642 1.91 [1.00; 2.82] 34.0% 34.0%
Phase 2 1.19 0.3738 1.19 [0.46; 1.93] 52.5% 52.5%
Phase 3 1.82 0.7364 1.82 [0.38; 3.27] 13.5% 13.5%
Fixed effect model 1.52 [0.99; 2.05] 100% −−
Random effects model 1.52 [0.99; 2.05] −− 100%
Heterogeneity: I−squared=0%, p=0.4428
−3 −2 −1 0 1 2 3
% DNA methylation difference

cg13793507

Cohort	Effect	SE	95%−CI	W(fixed)	W(random)
Phase 1	−0.93	0.2258	−0.93 [−1.37; −0.48]	36.9%	36.9%
Phase 2	−0.66	0.1837	−0.66 [−1.02; −0.30]	55.7%	55.7%
Phase 3	−0.88	0.5029	−0.88 [−1.87; 0.10]	7.4%	7.4%
**Fixed effect model**			**−0.78 [−1.05; −0.51]**	**100%**	**−−**
**Random effects model**			**−0.78 [−1.05; −0.51]**	**−−**	**100%**

Heterogeneity: I−squared=0%, p=0.6475

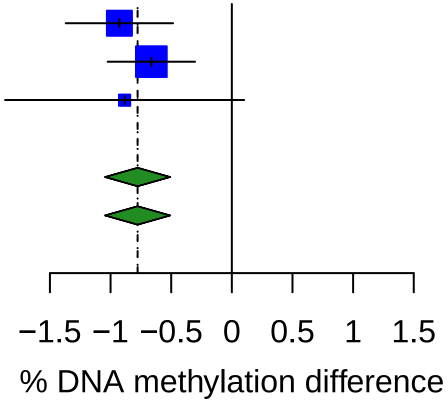

cg12083893

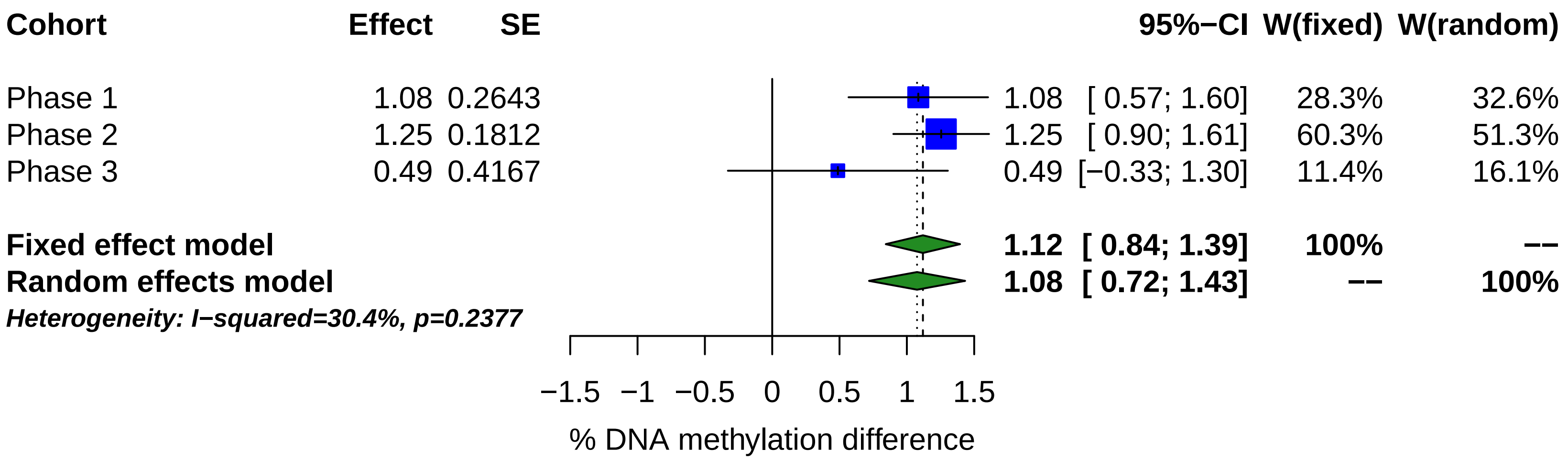
Cohort
Effect
SE
95%−CI
W(fixed)
W(random)
Phase 1
1.08
0.2643
1.08 [0.57; 1.60]
28.3%
32.6%
Phase 2
1.25
0.1812
1.25 [0.90; 1.61]
60.3%
51.3%
Phase 3
0.49
0.4167
0.49 [−0.33; 1.30]
11.4%
16.1%
Fixed effect model
1.12 [0.84; 1.39]
100%
−−
Random effects model
1.08 [0.72; 1.43]
−−
100%
Heterogeneity: I−squared=30.4%, p=0.2377
−1.5
−1
−0.5
0
0.5
1
1.5
% DNA methylation difference

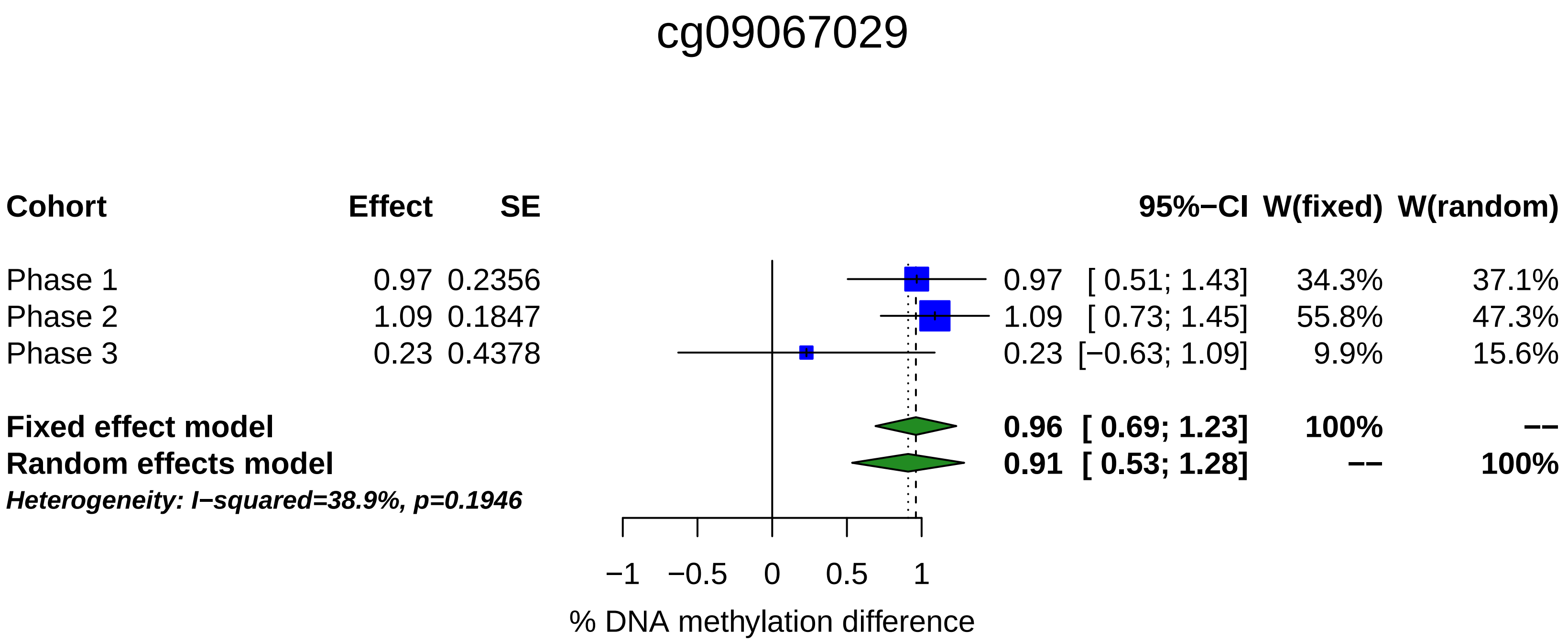
cg09067029
Cohort
Effect
SE
95%−CI
W(fixed)
W(random)
Phase 1 0.97 0.2356 0.97 [0.51; 1.43] 34.3% 37.1%
Phase 2 1.09 0.1847 1.09 [0.73; 1.45] 55.8% 47.3%
Phase 3 0.23 0.4378 0.23 [−0.63; 1.09] 9.9% 15.6%
Fixed effect model 0.96 [0.69; 1.23] 100% −−
Random effects model 0.91 [0.53; 1.28] −− 100%
Heterogeneity: I−squared=38.9%, p=0.1946
−1
−0.5
0
0.5
1
% DNA methylation difference

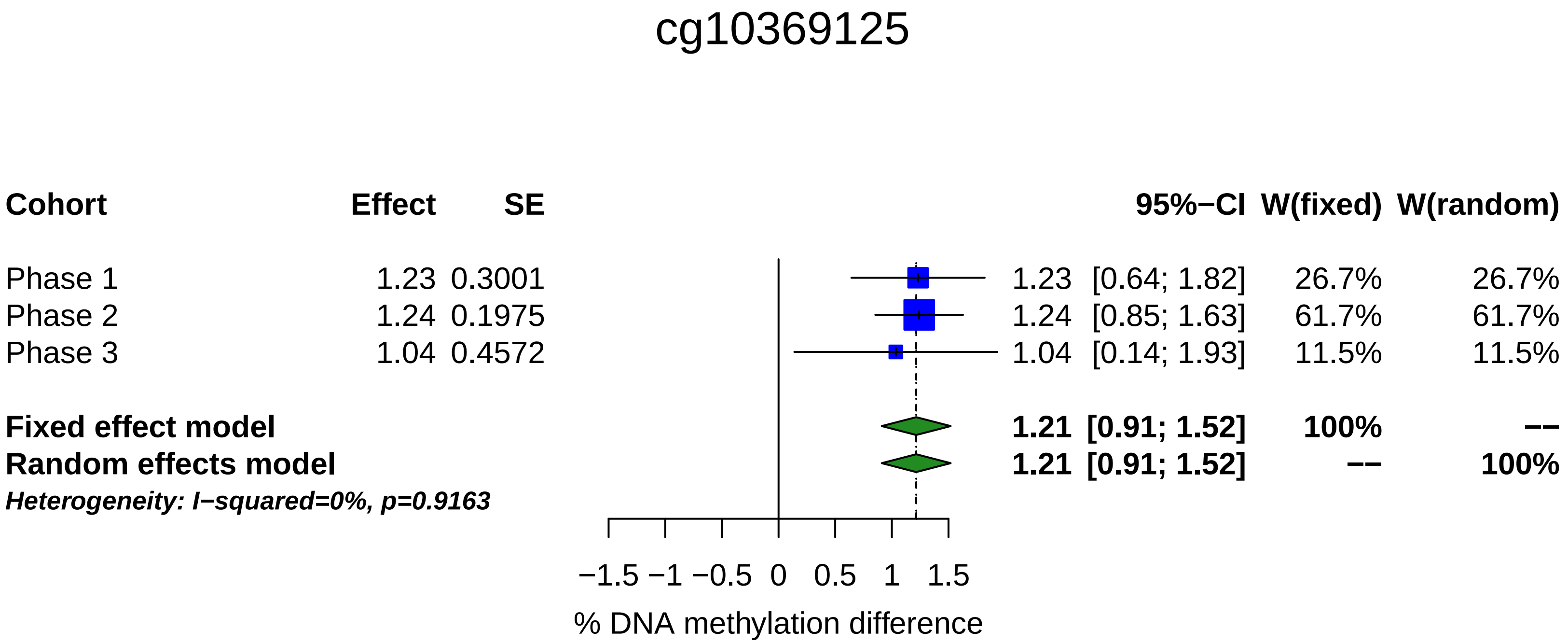
cg10369125
Cohort
Effect
SE
95%−CI
W(fixed)
W(random)
Phase 1 1.23 0.3001 1.23 [0.64; 1.82] 26.7% 26.7%
Phase 2 1.24 0.1975 1.24 [0.85; 1.63] 61.7% 61.7%
Phase 3 1.04 0.4572 1.04 [0.14; 1.93] 11.5% 11.5%
Fixed effect model 1.21 [0.91; 1.52] 100% −−
Random effects model 1.21 [0.91; 1.52] −− 100%
Heterogeneity: I−squared=0%, p=0.9163
−1.5 −1 −0.5 0 0.5 1 1.5
% DNA methylation difference

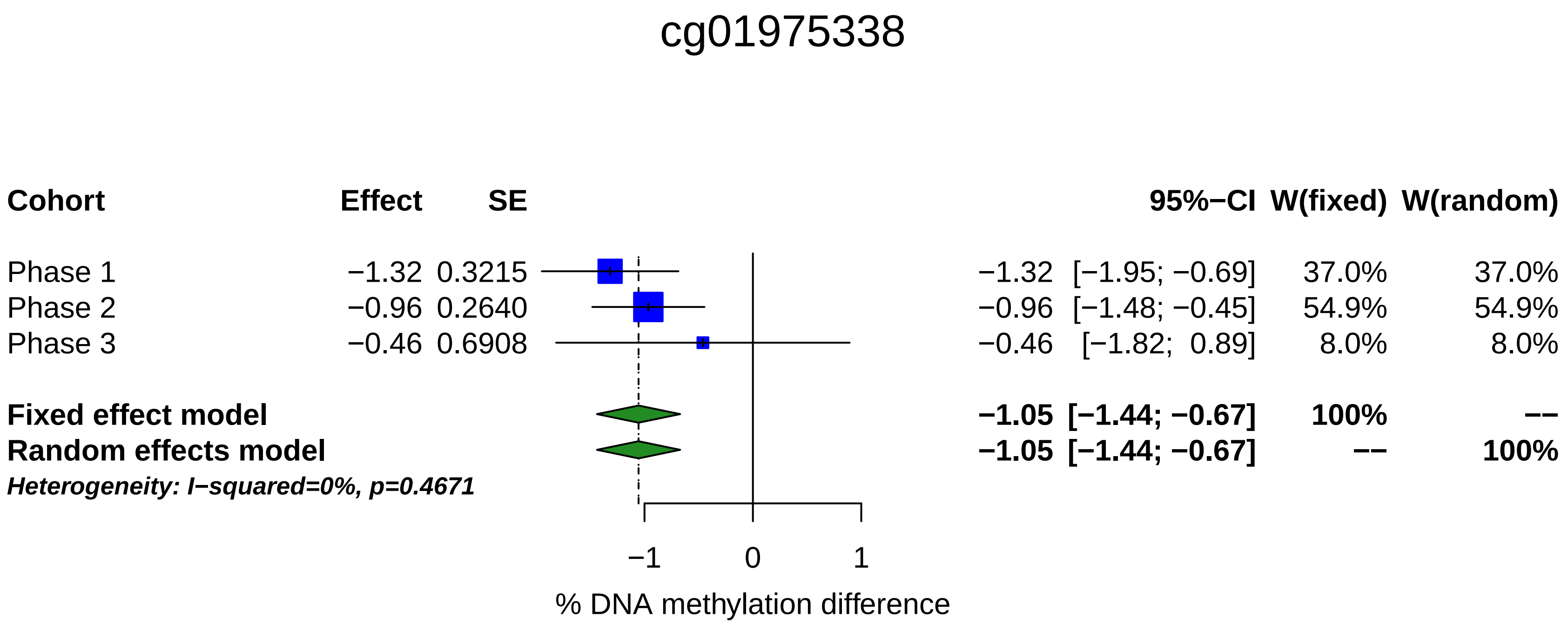
cg01975338
Cohort
Effect
SE
95%−CI
W(fixed)
W(random)
Phase 1 −1.32 0.3215 −1.32 [−1.95; −0.69] 37.0% 37.0%
Phase 2 −0.96 0.2640 −0.96 [−1.48; −0.45] 54.9% 54.9%
Phase 3 −0.46 0.6908 −0.46 [−1.82; 0.89] 8.0% 8.0%
Fixed effect model −1.05 [−1.44; −0.67] 100% −−
Random effects model −1.05 [−1.44; −0.67] −− 100%
Heterogeneity: I−squared=0%, p=0.4671
−1
0
1
% DNA methylation difference

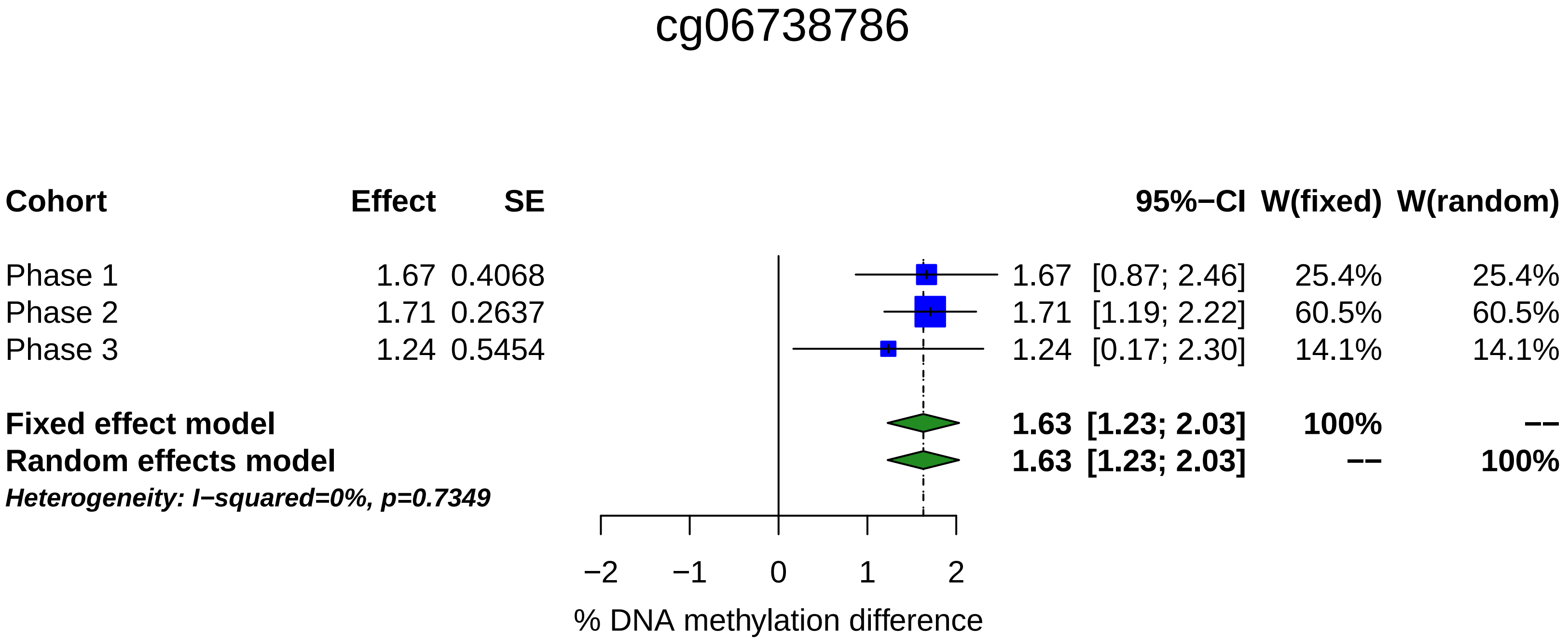
cg06738786
Cohort
Effect
SE
95%−CI
W(fixed)
W(random)
Phase 1
1.67
0.4068
1.67 [0.87; 2.46]
25.4%
25.4%
Phase 2
1.71
0.2637
1.71 [1.19; 2.22]
60.5%
60.5%
Phase 3
1.24
0.5454
1.24 [0.17; 2.30]
14.1%
14.1%
Fixed effect model
1.63 [1.23; 2.03]
100%
−−
Random effects model
1.63 [1.23; 2.03]
−−
100%
Heterogeneity: I−squared=0%, p=0.7349
−2
−1
0
1
2
% DNA methylation difference

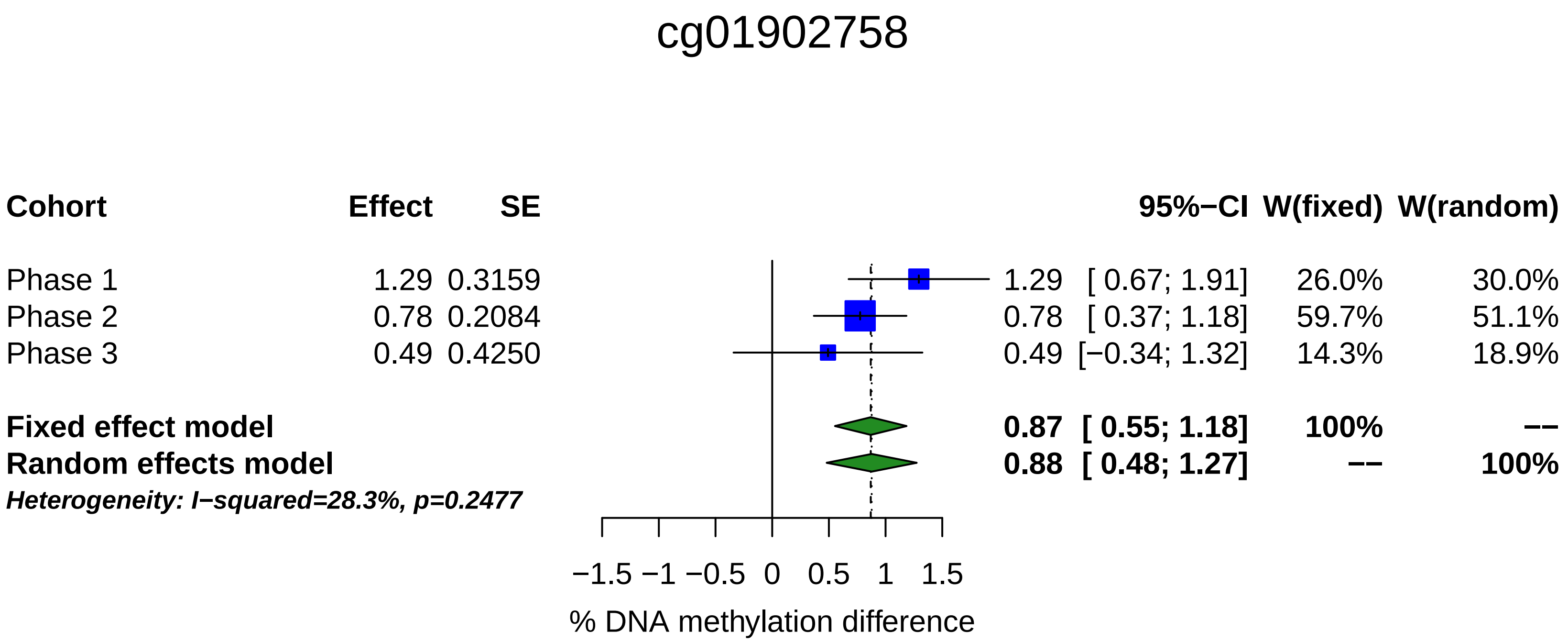
cg01902758
Cohort
Effect
SE
95%−CI
W(fixed)
W(random)
Phase 1 1.29 0.3159 1.29 [0.67; 1.91] 26.0% 30.0%
Phase 2 0.78 0.2084 0.78 [0.37; 1.18] 59.7% 51.1%
Phase 3 0.49 0.4250 0.49 [−0.34; 1.32] 14.3% 18.9%
Fixed effect model 0.87 [0.55; 1.18] 100% −−
Random effects model 0.88 [0.48; 1.27] −− 100%
Heterogeneity: I−squared=28.3%, p=0.2477
−1.5 −1 −0.5 0 0.5 1 1.5
% DNA methylation difference

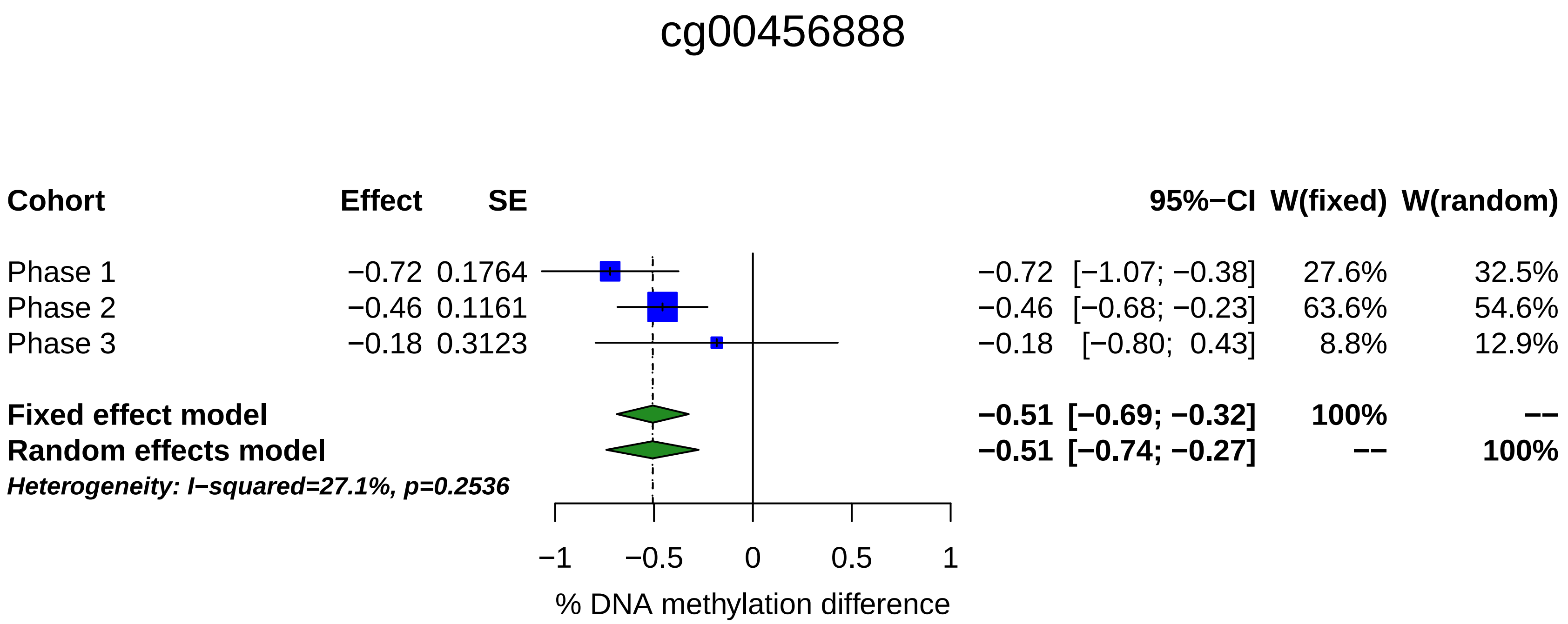
cg00456888
Cohort
Effect
SE
95%−CI
W(fixed)
W(random)
Phase 1 −0.72 0.1764 −0.72 [−1.07; −0.38] 27.6% 32.5%
Phase 2 −0.46 0.1161 −0.46 [−0.68; −0.23] 63.6% 54.6%
Phase 3 −0.18 0.3123 −0.18 [−0.80; 0.43] 8.8% 12.9%
Fixed effect model −0.51 [−0.69; −0.32] 100% −−
Random effects model −0.51 [−0.74; −0.27] −− 100%
Heterogeneity: I−squared=27.1%, p=0.2536
−1
−0.5
0
0.5
1
% DNA methylation difference
