# Supplementary material for: An integrated genetic-epigenetic analysis of schizophrenia: evidence for co-localization of genetic associations and differential DNA methylation
Source: Genome Biol. 2016 Aug 30;17(1):176. doi: 10.1186/s13059-016-1041-x (PMC5004279; doi:10.1186/s13059-016-1041-x)
Supplement: Additional file 4: — Examples of co-localization between variants associated with schizophrenia and DNA methylation. (PDF 335 kb) [file 13059_2016_1041_MOESM4_ESM.pdf]

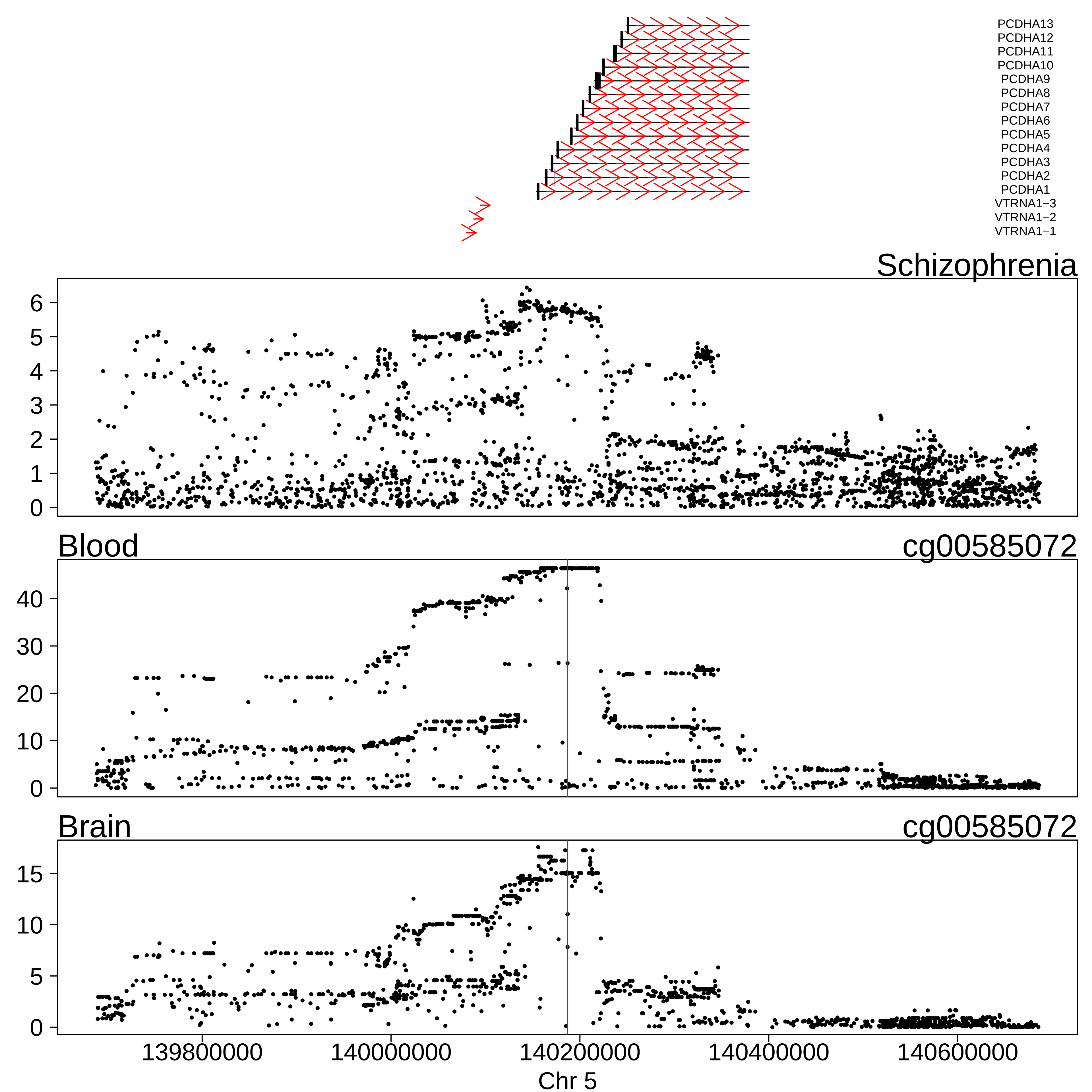
PCDHA13
PCDHA12
PCDHA11
PCDHA10
PCDHA9
PCDHA8
PCDHA7
PCDHA6
PCDHA5
PCDHA4
PCDHA3
PCDHA2
PCDHA1
VTRNA1−3
VTRNA1−2
VTRNA1−1
Schizophrenia
Blood
cg00585072
Brain
cg00585072
139800000
140000000
140200000
140400000
140600000
Chr 5

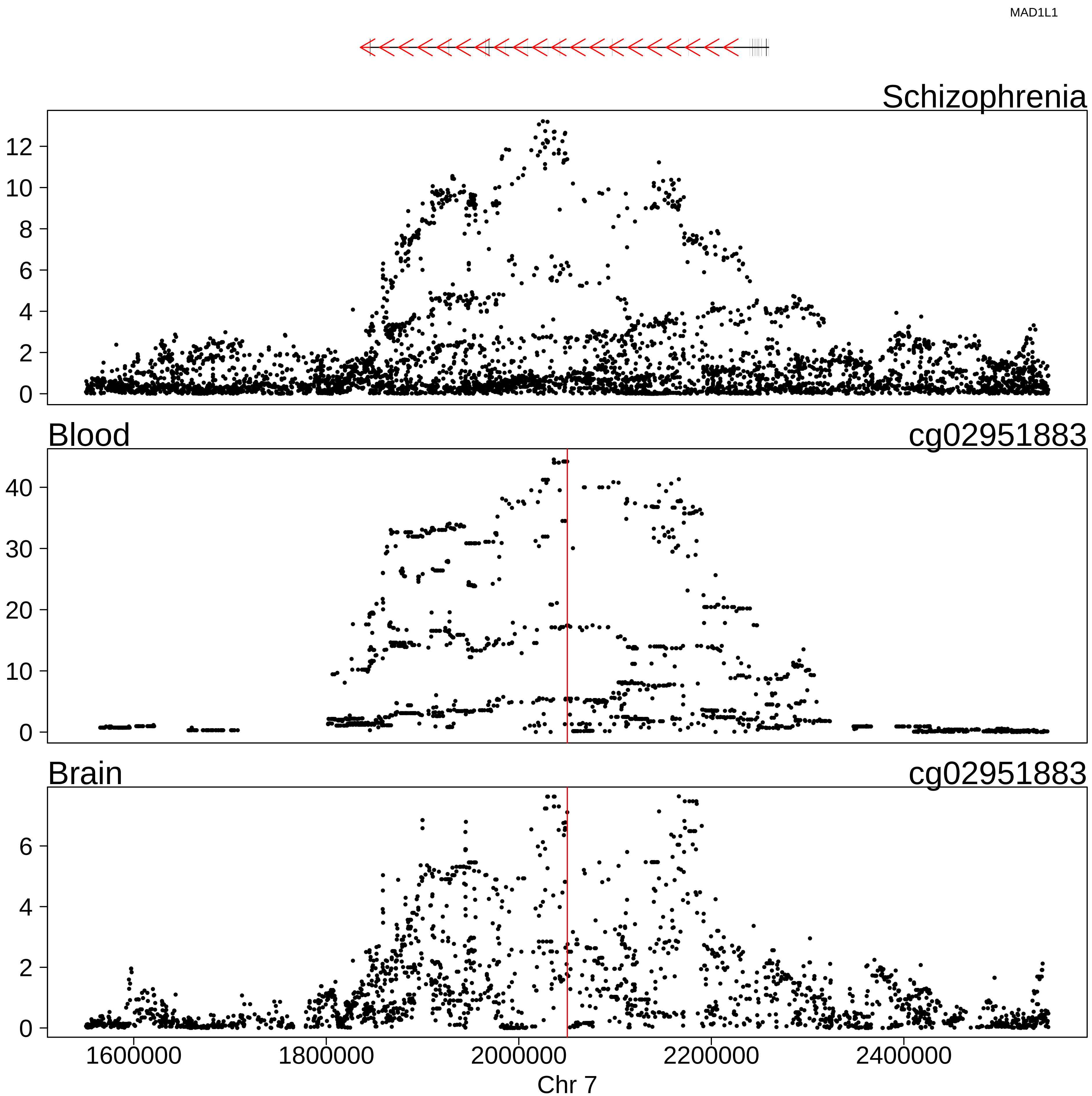

MAD1L1
Schizophrenia
0
2
4
6
8
10
12
Blood
cg02951883
0
10
20
30
40
Brain
cg02951883
0
2
4
6
1600000
1800000
2000000
2200000
2400000
Chr 7

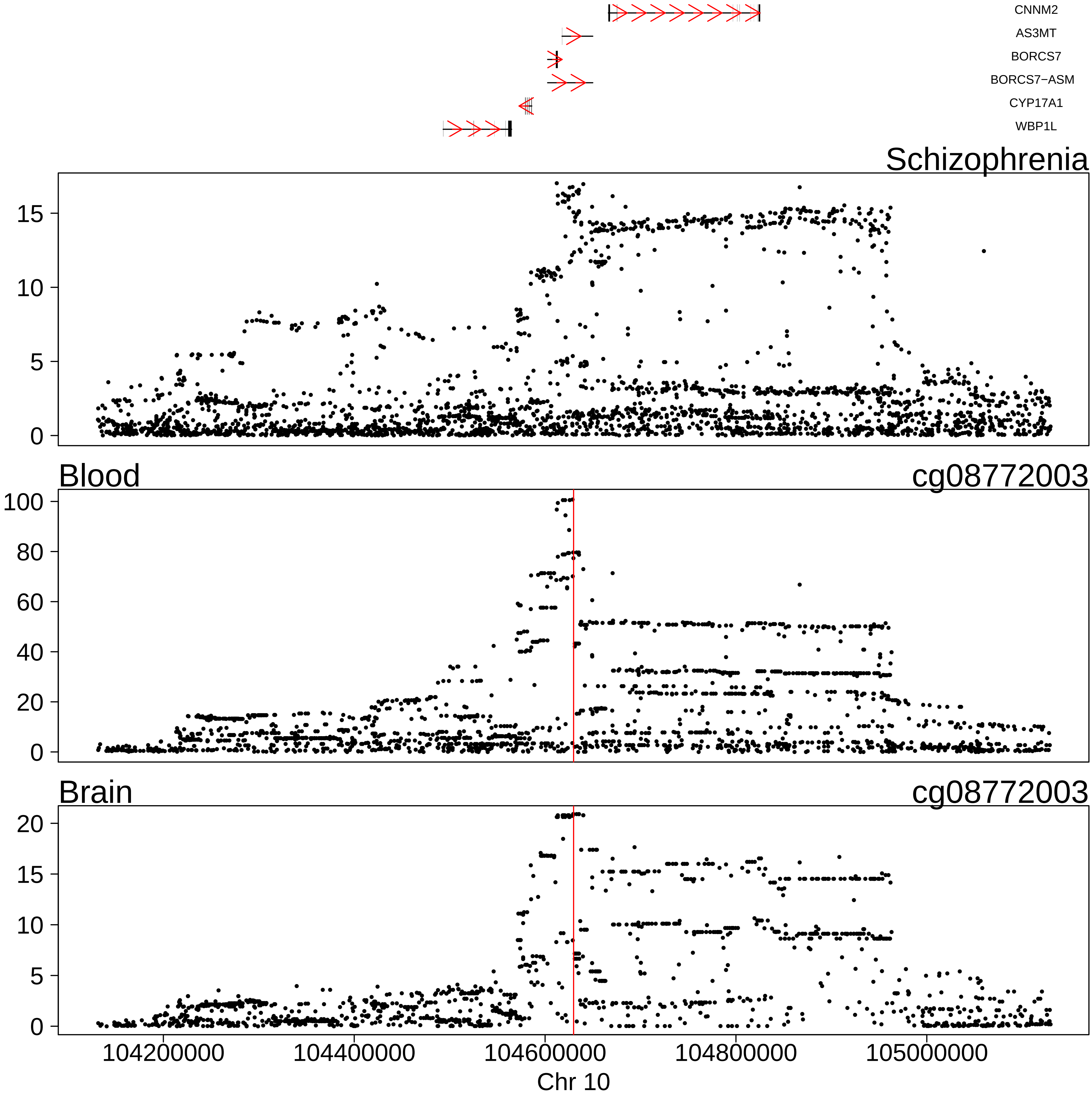
CNNM2
AS3MT
BORCS7
BORCS7−ASM
CYP17A1
WBP1L
Schizophrenia
Blood
cg08772003
Brain
cg08772003
104200000
104400000
104600000
104800000
105000000
Chr 10

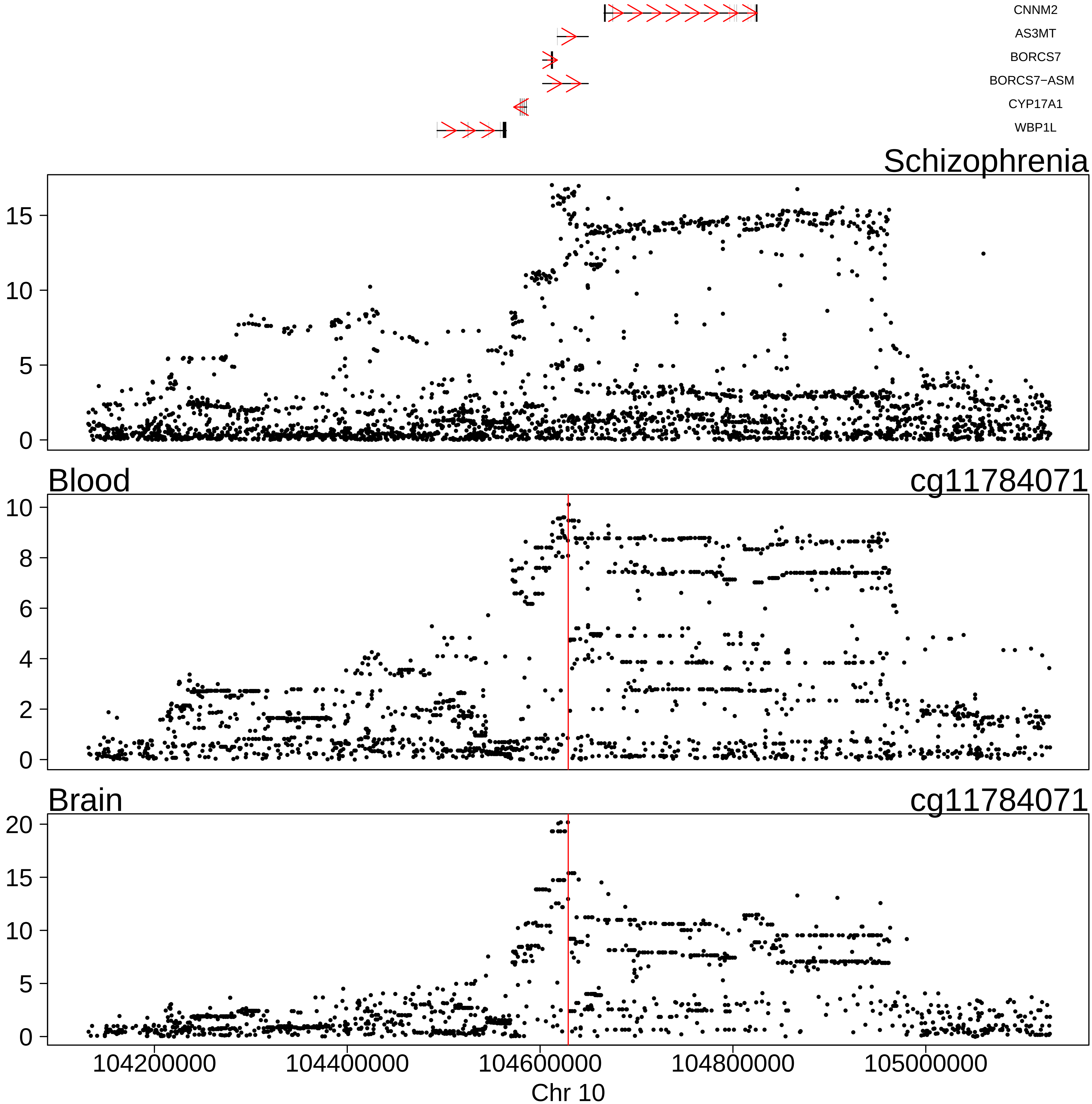
CNNM2
AS3MT
BORCS7
BORCS7−ASM
CYP17A1
WBP1L
Schizophrenia
Blood
cg11784071
Brain
cg11784071
104200000
104400000
104600000
104800000
105000000
Chr 10

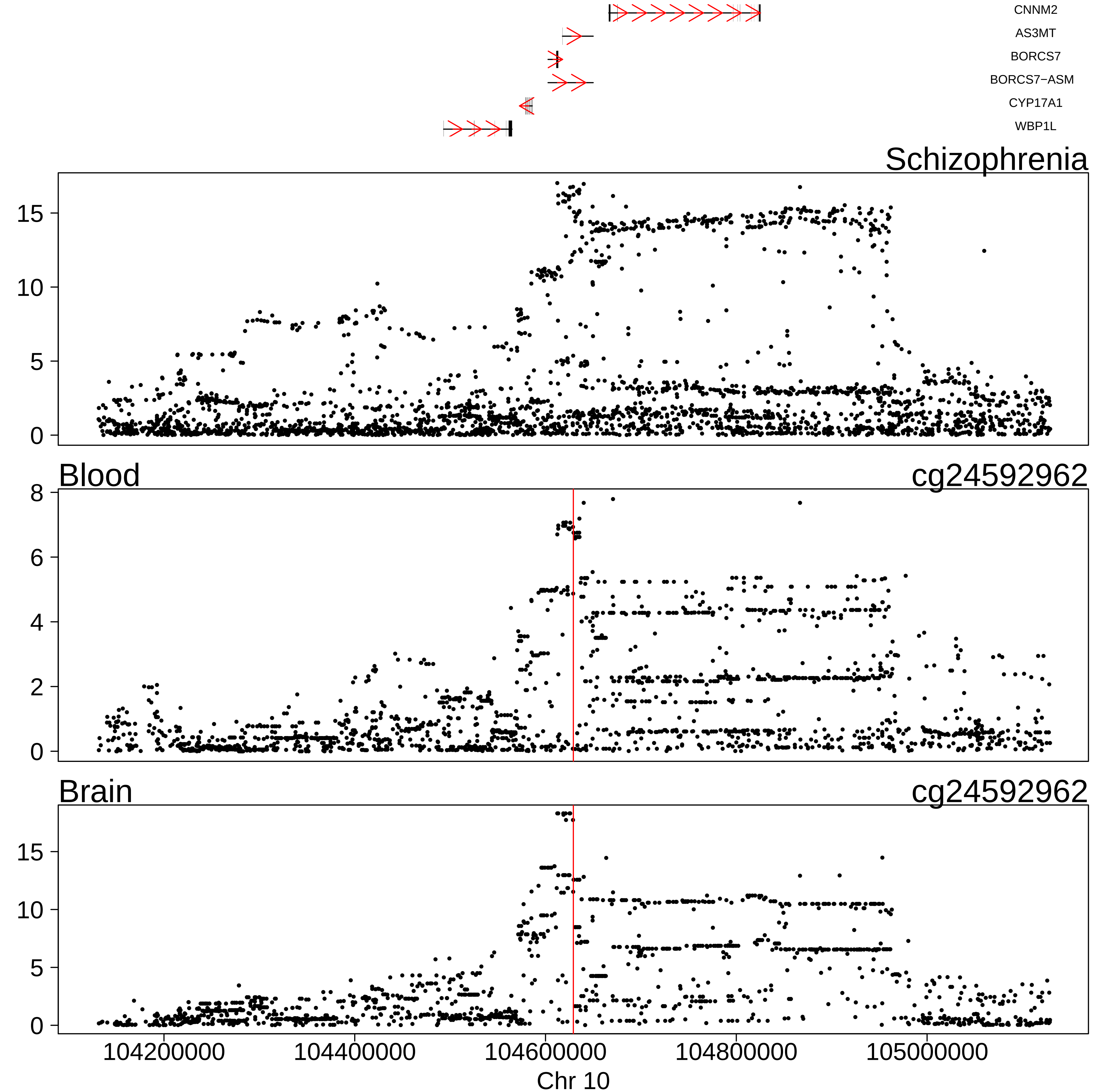

CNNM2
AS3MT
BORCS7
BORCS7−ASM
CYP17A1
WBP1L
Schizophrenia
Blood
cg24592962
Brain
cg24592962
104200000
104400000
104600000
104800000
105000000
Chr 10

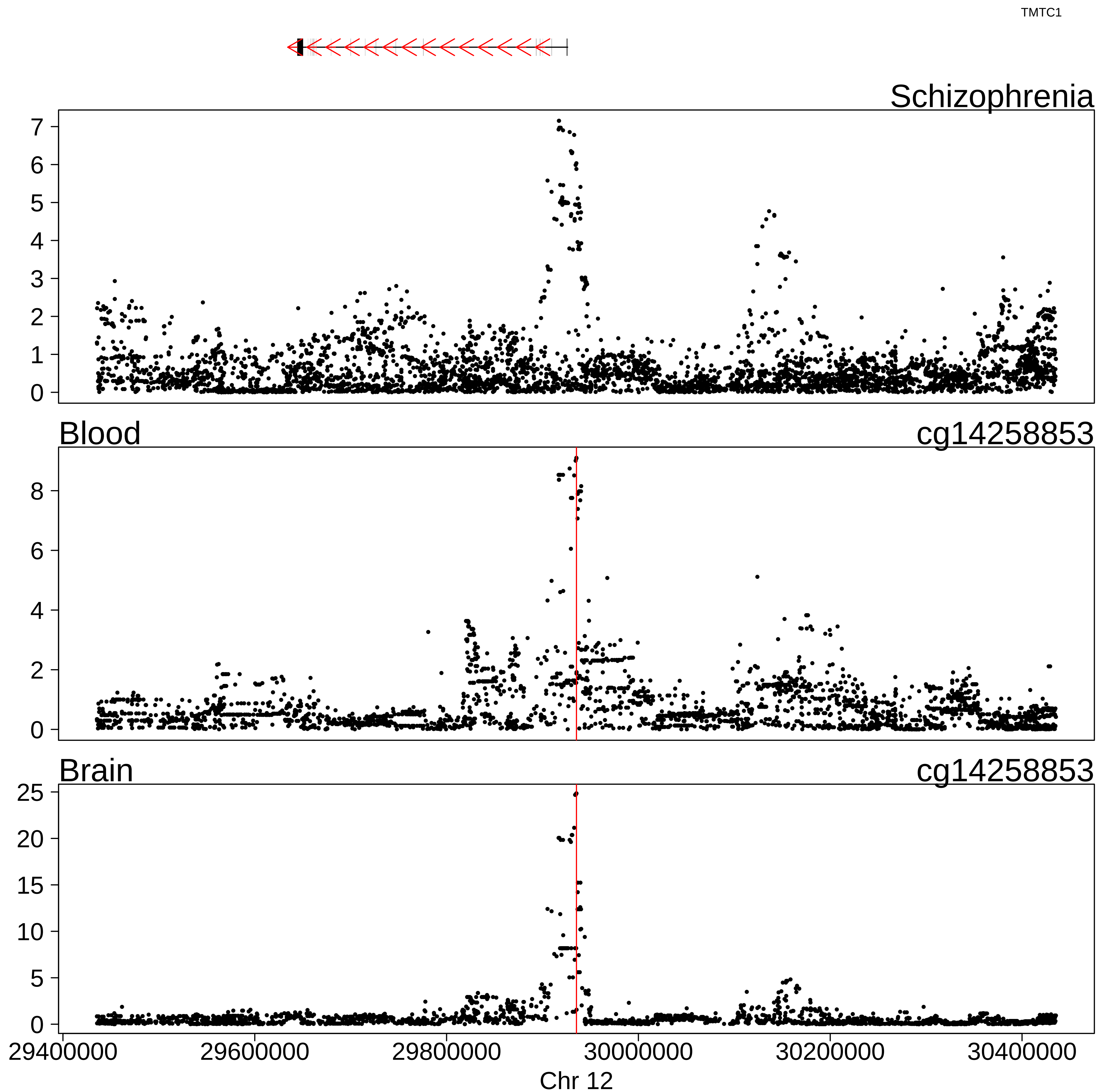
TMTC1
Schizophrenia
Blood
cg14258853
Brain
cg14258853
Chr 12
29400000
29600000
29800000
30000000
30200000
30400000

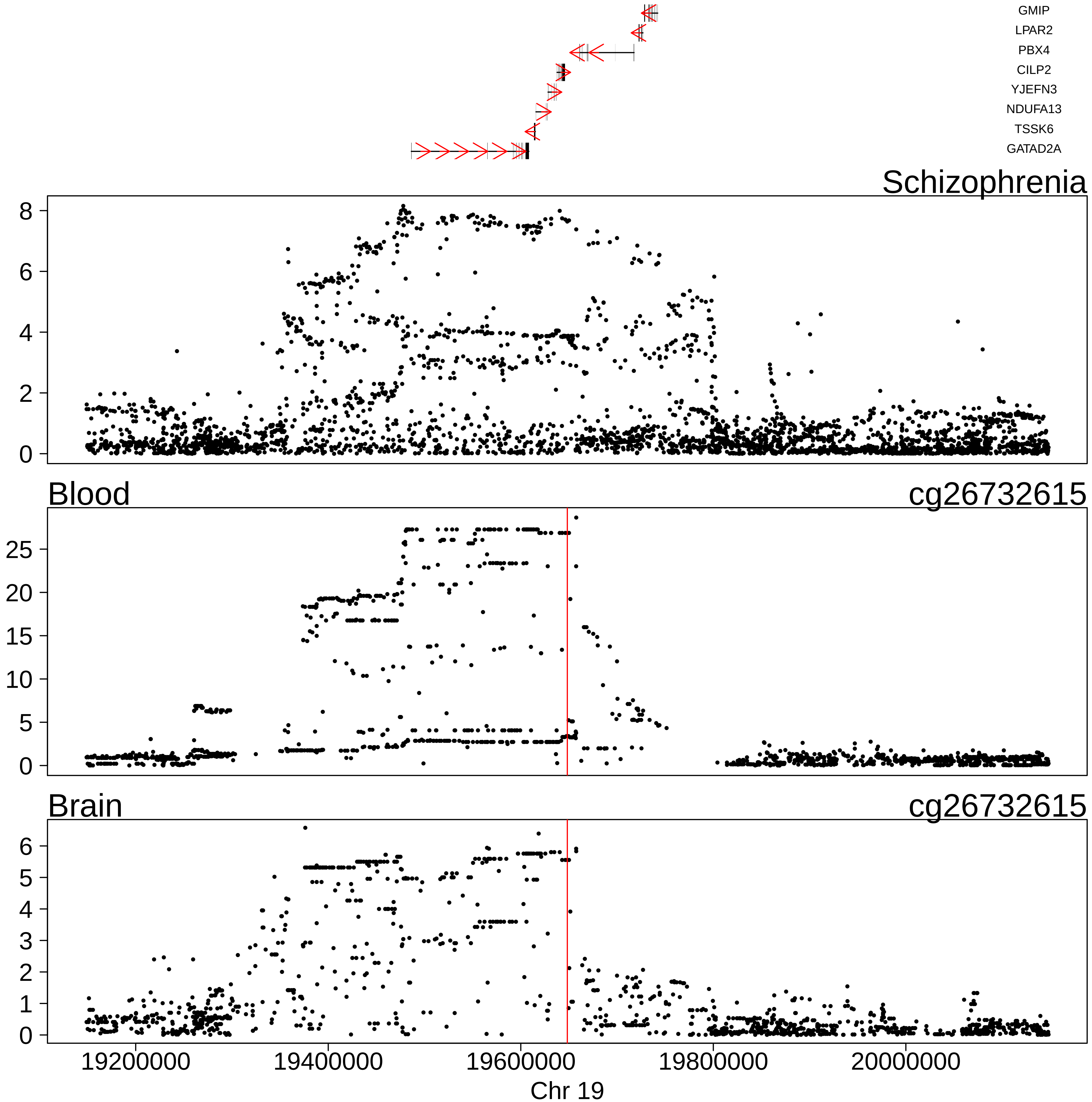

GMIP
LPAR2
PBX4
CILP2
YJEFN3
NDUFA13
TSSK6
GATAD2A
Schizophrenia
Blood
cg26732615
Brain
cg26732615
19200000
19400000
19600000
19800000
20000000
Chr 19
